# High-throughput autoantibody screening identifies differentially abundant autoantibodies in autism spectrum disorder

**DOI:** 10.3389/fnmol.2023.1222506

**Published:** 2023-10-16

**Authors:** Areej Mesleh, Hanan Ehtewish, Katie Lennard, Houari B. Abdesselem, Fouad Al-Shaban, Julie Decock, Nehad M. Alajez, Abdelilah Arredouani, Mohamed M. Emara, Omar Albagha, Lawrence W. Stanton, Sara A. Abdulla, Jonathan M. Blackburnand, Omar M. A. El-Agnaf

**Affiliations:** ^1^College of Health and Life Sciences, Hamad Bin Khalifa University, Qatar Foundation, Doha, Qatar; ^2^Neurological Disorders Research Center, Qatar Biomedical Research Institute, Hamad Bin Khalifa University, Qatar Foundation, Doha, Qatar; ^3^Sengenics Corporation, Level M, Plaza Zurich, Damansara Heights, Kuala Lumpur, Malaysia; ^4^Proteomics Core Facility, Qatar Biomedical Research Institute, Hamad Bin Khalifa University, Qatar Foundation, Doha, Qatar; ^5^Translational Cancer and Immunity Center, Qatar Biomedical Research Institute, Hamad Bin Khalifa University, Qatar Foundation, Doha, Qatar; ^6^Diabetes Research Center, Qatar Biomedical Research Institute, Hamad Bin Khalifa University, Doha, Qatar; ^7^Basic Medical Sciences Department, College of Medicine, Qatar University Health, Qatar University, Doha, Qatar; ^8^Department of Integrative Biomedical Sciences, Faculty of Health Sciences, University of Cape Town, Cape Town, South Africa; ^9^Institute of Infectious Disease and Molecular Medicine, Faculty of Health Sciences, University of Cape Town, Cape Town, South Africa

**Keywords:** autism spectrum disorder, autoantibodies, profiling, pathways, biomarker

## Abstract

**Introduction:**

Autism spectrum disorder (ASD) is a neurodevelopmental condition characterized by defects in two core domains, social/communication skills and restricted/repetitive behaviors or interests. There is no approved biomarker for ASD diagnosis, and the current diagnostic method is based on clinical manifestation, which tends to vary vastly between the affected individuals due to the heterogeneous nature of ASD. There is emerging evidence that supports the implication of the immune system in ASD, specifically autoimmunity; however, the role of autoantibodies in ASD children is not yet fully understood.

**Materials and methods:**

In this study, we screened serum samples from 93 cases with ASD and 28 healthy controls utilizing high-throughput KoRectly Expressed (KREX) i-Ome protein-array technology. Our goal was to identify autoantibodies with differential expressions in ASD and to gain insights into the biological significance of these autoantibodies in the context of ASD pathogenesis.

**Result:**

Our autoantibody expression analysis identified 29 differential autoantibodies in ASD, 4 of which were upregulated and 25 downregulated. Subsequently, gene ontology (GO) and network analysis showed that the proteins of these autoantibodies are expressed in the brain and involved in axonal guidance, chromatin binding, and multiple metabolic pathways. Correlation analysis revealed that these autoantibodies negatively correlate with the age of ASD subjects.

**Conclusion:**

This study explored autoantibody reactivity against self-antigens in ASD individuals' serum using a high-throughput assay. The identified autoantibodies were reactive against proteins involved in axonal guidance, synaptic function, amino acid metabolism, fatty acid metabolism, and chromatin binding.

## 1. Introduction

Autism spectrum disorder (ASD) is a term that encompasses a group of neurodevelopmental conditions. The common dominator of these conditions is a defect in two core domains, social/communication skills and restricted or repetitive behaviors (American Psychiatric Association, 2013). There is no approved biomarker for ASD diagnosis, and the current diagnostic method is based on clinical evaluation, which tends to vary greatly between the affected individuals. The advancement in high-throughput proteomics techniques, which made measuring multiple proteins simultaneously a reality, motivated scientists to undertake proteomics discovery studies using body fluids of ASD subjects in an effort to identify biomarkers for diagnosis and understanding of ASD pathogenesis (Voineagu et al., 2011; Saghazadeh et al., 2019; Hewitson et al., 2021). Many of these studies have identified dysregulation in various components of the immune system as they observed upregulation in pro-inflammatory markers, such as IFN-γ, TNF-α, IL-6, and IL-8, in the brain of ASD individuals at both the protein and mRNA levels (Voineagu et al., 2011; Saghazadeh et al., 2019), thus suggesting that pro-inflammation may play a pivotal role in ASD pathogenesis.

In addition, autoimmune abnormalities have been observed in ASD (reviewed by Edmiston et al., 2017). The notion that maternal autoantibodies may play a role in children's neurodevelopment arose when maternal IgG antibodies were found in the cerebrospinal fluid of fetuses and newborns, suggesting that maternal autoantibodies can pass through the blood–brain barrier (Adinolfi et al., 1976). Several studies further observed that maternal autoantibodies of ASD subjects are reactive toward fetal brain proteins (Braunschweig et al., 2008, 2013). Moreover, studies on the blood of ASD children found a significant increase of autoantibodies in ASD compared to healthy controls; these autoantibodies were specific for myelin basic protein (MBP), ribosomal P protein, and nuclear antigens (Singh et al., 1993; Mostafa et al., 2014, 2016). The majority of published studies have tested a limited number of autoantibodies in ASD, and, there is yet to be a study that utilizes high-throughput technology to test a large number of autoantibodies in ASD cases. Therefore, the overall landscape of ASD's autoantibody reactivity across a large spectrum of self-antigens is still unknown.

Autoantibodies originate from autoreactive B-cells. In a normal scenario, B-cells producing antibodies that bind with a high affinity to self-antigens are eliminated, while B-cells producing antibodies with medium-/low-binding affinity are more likely to escape the elimination processes (Ludwig et al., 2017). Many low-affinity autoantibodies have anti-inflammatory features depending on the antibody subclass (IgM) and the post-translational modifications it has undergone (glycosylation/sialylation) (Karsten et al., 2012; Mannoor et al., 2013). However, in many cases, autoantibodies against self-antigens can interfere with physiological processes and induce devastating life-long illnesses. For instance, autoantibodies may disrupt the physiological function of the cells by different mechanisms such as mimicking receptor function, inducing cell signaling and inflammatory processes, blocking neurotransmission, and inducing cell lysis (Ludwig et al., 2017).

Collectively, the studies mentioned above have presented emerging evidence linking immune system dysregulation to ASD. Nevertheless, the comprehensive autoantibody profile of individuals with ASD and the potential mechanisms through which these autoantibodies might influence ASD remain to be fully elucidated. Therefore, investigating the autoantibody profile of ASD individuals might provide insights into the autoimmune status of ASD. Herein, we screen for 1,623 autoantibodies using the serum of 93 ASD individuals and 28 healthy controls between the ages of 6 and 15 years. To the best of our knowledge, this is the first study that screens serum autoantibodies of ASD using high-throughput KoRectly Expressed (KREX) technology. The study aims to explore the autoreactivity of IgG antibodies against self-antigens in ASD individuals to identify potential autoantibodies that could underly ASD pathology.

## 2. Materials and methods

### 2.1. Study design and samples collection

A total of 121 serum samples were used in this study, comprising 93 samples from individuals with ASD and 28 samples from healthy controls (HCs). Non-fasting peripheral blood was collected on the day of the interview and processed to obtain serum, which was subsequently stored at −80°C until further analysis. All sample processing and storage procedures were conducted at Qatar Biomedical Research Institute (QBRI). The study was performed in accordance with the guidelines of the Declaration of Helsinki and was ethically approved (QBRI-IRB 2018-024). Written informed consent and assent were given to both parents and children, respectively, for all ASD individuals and HCs. ASD individuals were clinically diagnosed using the Diagnostic and Statistical Manual of Mental Disorders, Fifth Edition (DSM-5). The severity levels of ASD individuals were evaluated using the second edition of the Autism Diagnostic Observation Schedule (ADOS-2) test. In addition, the social and communication skills of HCs were evaluated using the Social Communication Questionnaire (SCQ). Table 1 summarizes the demographic information obtained from the participants.

**Table 1:** Study participant summary statistics.

	**ASD cases**	**Healthy controls**	**Statistical test**
Number of participants	*N* = 93	*N* = 28	-
Age (mean ± SD)	8.26 ± 2.29	11.29 ± 2.05	3.537 × 10^−8^
Gender (F/M)	20/73	14/14	0.008
DSM-5	3.78 ± 1.72	-	-
ADOS-2 scores (mean ± SD)	6.39 ± 1.47	-	-
SCQ	-	5.71 ± 3.35	-

### 2.2. Samples processing, assay description, and normalization

ASD cohort samples were processed at Qatar Biomedical Research Institute (QBRI). Samples were analyzed for antigen-specific autoantibodies using i-Ome protein arrays (Sengenics, Singapore), developed using KREX technology to provide a high-throughput immunoassay based on correctly folded, full-length, and functional recombinant human proteins (Blackburn et al., 2012; Adeola et al., 2016). The i-Ome arrays contain more than 1,600 human antigens, enriched for kinases, signaling molecules, cytokines, interleukins, chemokines, as well as known autoimmune antigens; the full list of proteins is provided in Supplementary Table S1. In addition, the array content is curated to represent the characteristics of protein subsets including both known and possible autoantigenic proteins across a range of disease areas (including immune-mediated diseases, malignancy, neurological disorders, endocrine disorders, and immunodeficiency). All proteins are of full length, with the exception of receptor tyrosine kinases, which are depicted as distinct cytosolic and extracellular domains.

The antigens were expressed in Sf9 insect cells, serving as fusions to a biotin carboxyl carrier protein (BCCP) domain through the utilization of a baculoviral system, a methodology previously described (Beeton-Kempen et al., 2014). The BCCP tag is endowed with biotinylation *in vivo* by host cell biotin ligase, onto a single specific lysine residue, positioned ~50 Angstroms away from the N- and C-termini of the tag. The biotin moiety serves as a site for connecting each recombinant protein to a streptavidin-coated microarray surface, as previously described (Beeton-Kempen et al., 2014). It is important to note that insect cells are well known for their capacity to bestow mammalian-like glycosylation as well as other PTMs onto recombinant proteins.

Samples were diluted in Serum Albumin Buffer (SAB) at optimized dilution 1:200. Samples and controls were randomized and added to the microarray slides for 1 h, and each slide contained antigens that were spotted in technical quadruplicate, and then IgG levels were then detected by a secondary Cy3-labeled anti-human IgG antibody. Slides were scanned at a fixed setting using an Agilent G4600AD fluorescence microarray scanner, generating a 16-bit TIFF file. A visual quality control check was conducted, and arrays showing spot merging or other artifacts were re-assayed. A GenePix Array List (GAL) file containing information regarding the location and identity of all probed spots was used to aid with image analysis. Automatic extraction and quantification of each spot were performed using GenePix Pro 7 software (Molecular Devices), generating the mean foreground and local background pixel intensities for each spot. Raw data were imported in R (version 4.0.5) and were analyzed using a Sengenics in-house normalization pipeline.

The data were log2 transformed and normalized by subtracting the mean background intensity from the mean foreground intensity to get the net intensity per spot in the relative fluorescence unit (RFU). Subsequently, antigen replicas with coefficient variation (CV%) > 20% were flagged and excluded. Thereafter, the generated dataset underwent negative control filtration (NCF), an approach for excluding non-specific autoantibody signals. In brief, NCF identifies a baseline low-level net intensity for the array using quadruplicate technical replicates of a negative control protein that does not react with sera. Individual antigens with intensity values that correlate closely with the negative control spots across all the samples possess no meaningful information related to the disease being studied and, therefore, are excluded from downstream analyses. No arbitrary cutoff value was used to eliminate non-reactive signals. Furthermore, Loess normalization was applied to the dataset using the normalizeCyclicLoess function in R, which normalizes each pair of columns in a matrix to each other. Then, combat correction, which utilizes the empirical Bayesian approach, was applied to adjust for batch effect when the potential cause of variation is known, as samples were processed in two different batches across 2021 and 2022. A *t*-distributed stochastic neighbor embedding (t-SNE) plot of the data before and after normalization is displayed in Supplementary Figure S1.

### 2.3. Autoantibody identification and pathway prediction

Differentially expressed autoantibodies were identified using the *Limma* (Linear Models for Microarray Data) package (version 3.46.0) in R. Age and sex were weighted as covariates in the model because they showed a significant difference between ASD cases and HC (Table 1). Supplementary Figure S2 illustrates that the dataset does not exhibit age- or sex-dependent clustering. Differentially expressed autoantibodies were considered significant if their unadjusted *p*-value ≤ 0.05, given that none of the autoantibodies withstood Benjamini–Hochberg (BH) multiple correction. Correlation analysis with the ADOS-2 score and age was performed using the cor.test function, and all the heatmaps were generated using the Heatmap function from the ComplexHeatmap package. All the analyses were conducted using R software (version 4.0.5).

Protein enrichment analysis was performed on the corresponding proteins of the differentially expressed autoantibodies using ShinyGO (version 0.77) (http://bioinformatics.sdstate.edu/go/) to detect overrepresentation in cellular components, molecular functions, and biological processes in addition to the KEGG database. All the enriched terms were tested using a Fisher exact test with an adjusted *p*-value (FDR < 0.05), and a minimum of two proteins were required in each enriched pathway. Protein–protein interactions (PPIs) and hub proteins of the differential autoantibodies were identified using STRING (version 11.5), and then the network files were uploaded to Cystoscape (version 3.9.1) for network visualization and hub proteins identification.

### 2.4. Sequence identity analysis

To assess cross-reactivity among proteins that express similar antigen epitopes and are highly correlated, we checked the correlation between the differentially expressed autoantibodies. The corresponding proteins of highly correlated autoantibodies (*R*^2^ > 0.7) were then aligned using the Uniport alignment tool. In addition, we used the BLAST tool to check protein isoforms with >50% identity and then performed correlation to rule out the possibility of cross-reactivity between the detected autoantibodies and the antigens available in the panel.

## 3. Results

### 3.1. Cohort characteristics

**Figure 1:**
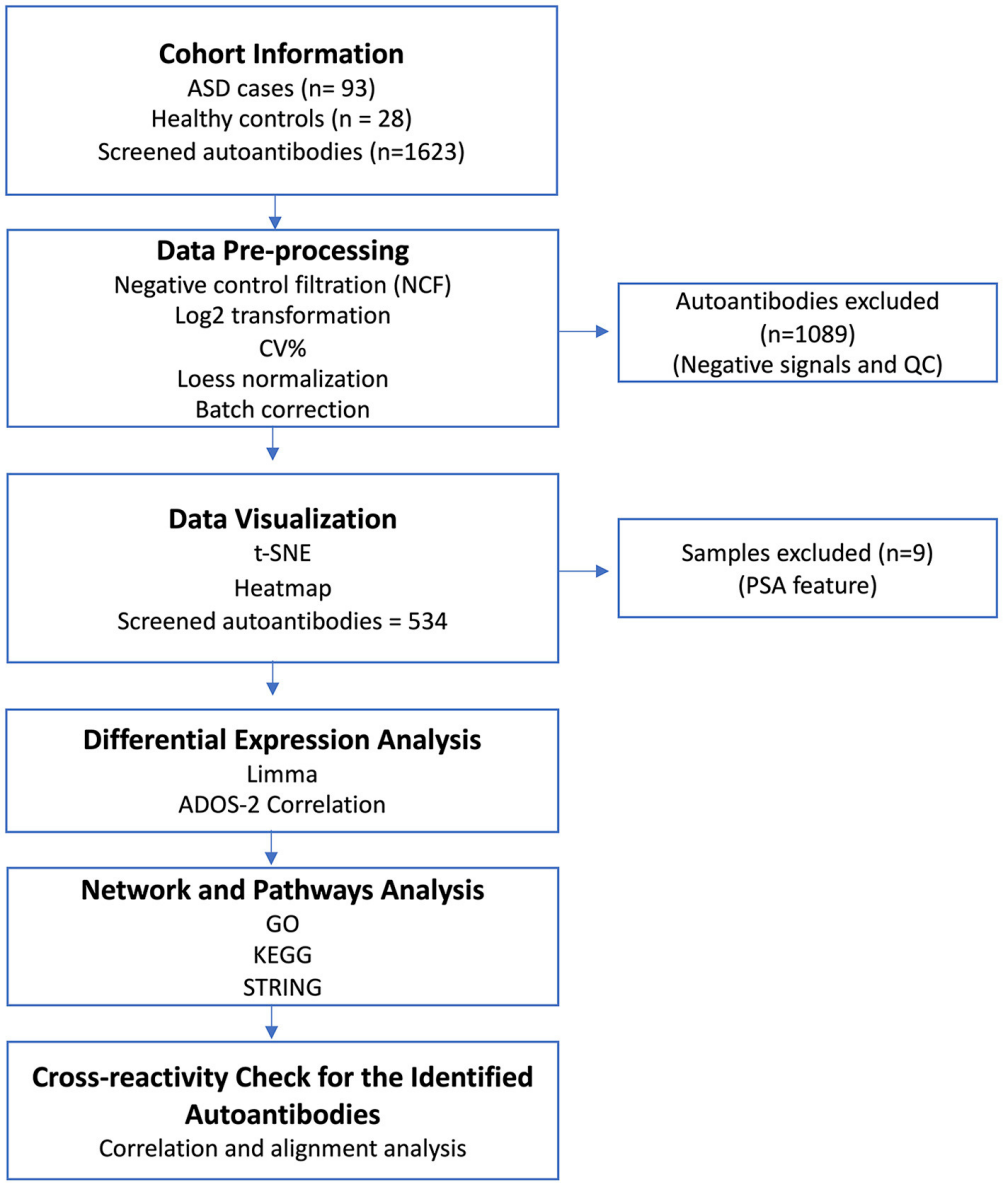
Study overview. Serum samples from ASD cases (*n* = 93) and healthy controls (HCs; *n* = 28) were collected along with the participants' demographic information. The serum samples were screened using Sengenics iOme arrays. Data pre-processing and QC analysis were performed on the generated data. Then, the generated data were visualized to detect sample outliers and batch effects. Different bioinformatic and statistical analysis approaches were deployed to analyze the dataset. Finally, a cross-reactivity check was performed on the potential autoantibodies to confirm the validity of their signals.

**Figure 2:**
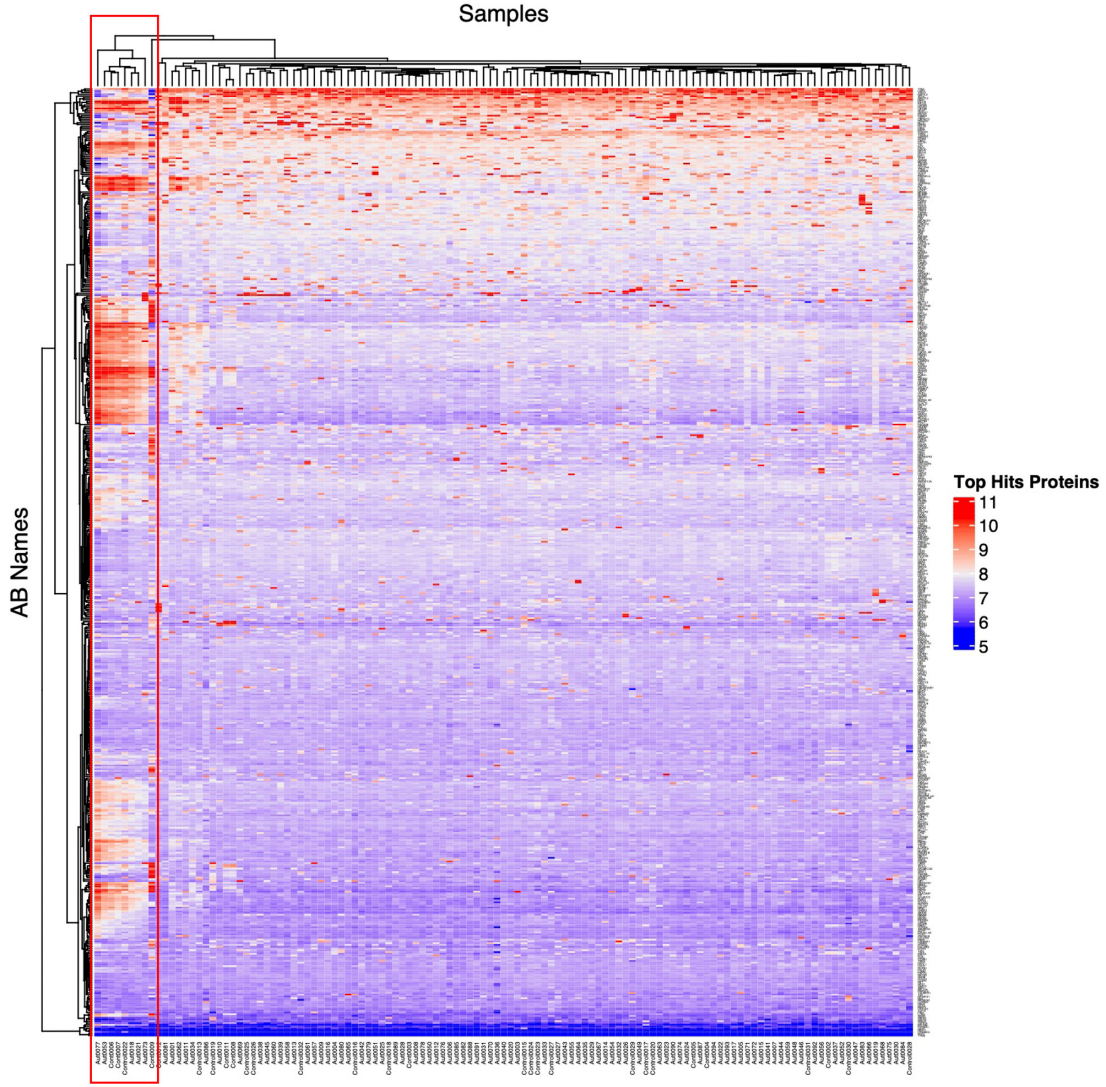
Unsupervised heatmap of ASD cases and HCs. This unsupervised heatmap was generated from all screened samples and autoantibodies (AB); the red box highlights the self-reactive polyspecific antibody (PSA) response in the samples. Any sample that exhibits this phenotype was excluded from downstream analysis.

An overview of the study design is illustrated in Figure 1. This study tested a total of 121 participants, i.e., ASD cases (*n* = 93) and HCs (*n* = 28), who ranged from 6 to 15 years of age. The average ages for ASD cases and HCs were 8.26 ± 2.29 and 11.29 ± 2.05, respectively. The male-to-female ratio in the HCs group was equal (50%); however, the majority of ASD cases (78.5%) were male subjects because the prevalence of ASD is higher in male subjects compared to female subjects, and it can reach up to 4:1 (Maenner et al., 2021), which is consistent with the male-to-female ratio in the ASD group. Both age and sex exhibited statistically significant differences between the groups (Table 1). All ASD cases were clinically diagnosed with ASD using DSM-5. Subsequently, the severity level of 50 ASD cases was assessed using the ADOS-2 score. The demographic information of ASD cases and HCs is summarized in Table 1. Upon visualizing the screened samples and autoantibodies on a heatmap, we identified a pattern of self-reactive polyspecific antibodies (PSA) in nine samples, as shown in Figure 2. This pattern occurs when the antibodies retain low-affinity characteristics with a spectrum of antigens, including self-antigens. This phenomenon happens because of some types of infections and autoimmune conditions (Wang et al., 1999). All samples that displayed PSA features were removed from downstream analysis.

### 3.2. Autoantibodies profiling identified up and downregulated humoral response to self-antigens in ASD

**Figure 3:**
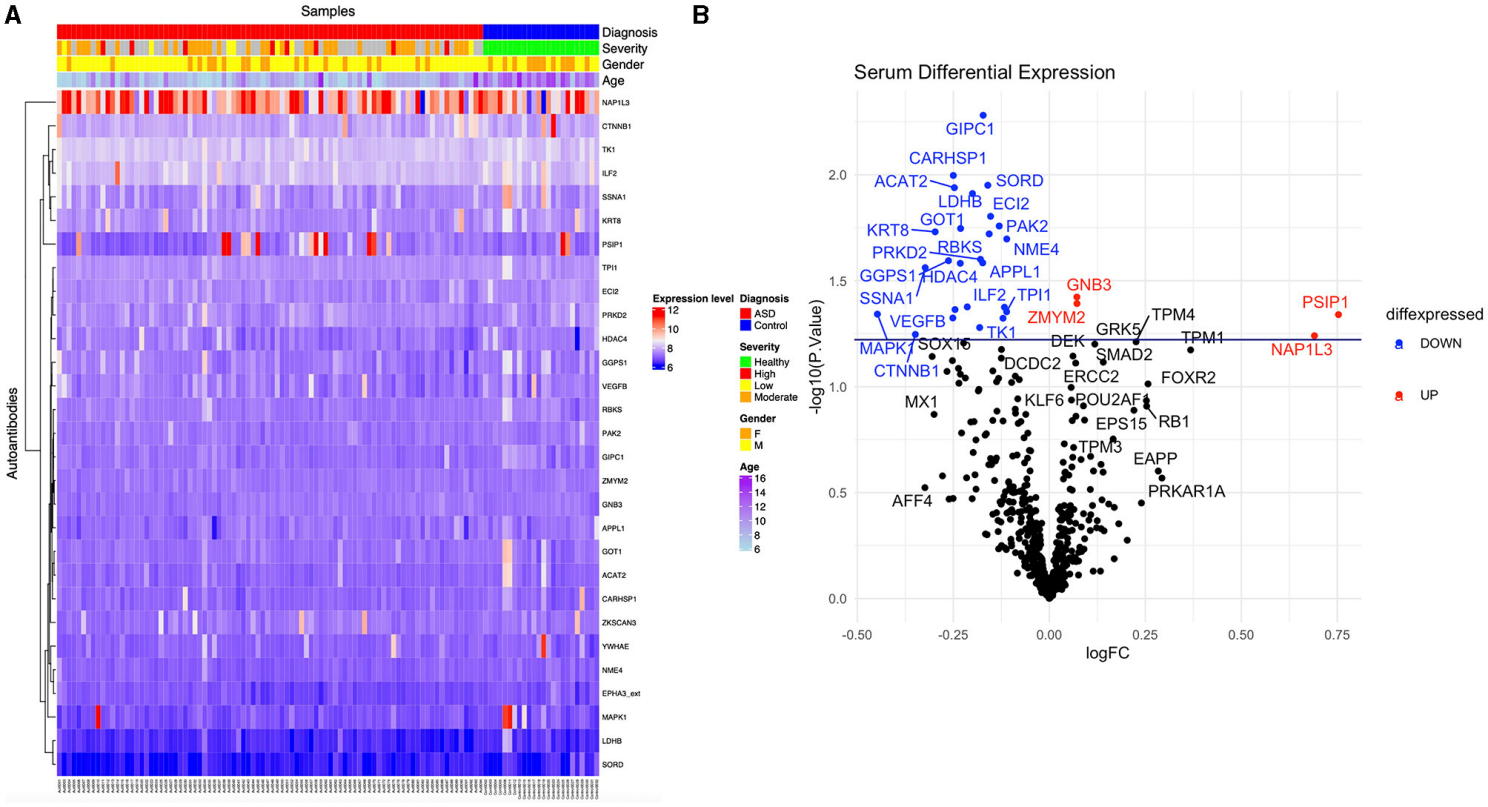
Differential expression of ASD autoantibodies. **(A)** Supervised heatmap of ASD cases and HCs using the differential autoantibodies (*p*-value ≤ 0.05), gender (male = yellow, female = orange), diagnosis (ASD = red, HCs = blue), severity (healthy = green, high = red, low = yellow, moderate = orange), and expression level (high = red, low=blue). **(B)** The volcano plot shows log2 fold change (*x*-axis) against Limma –log10 *p*-value (*y*-axis) for all the autoantibodies differentially expressed between HCs and ASD cases. The top significantly upregulated and downregulated proteins are labeled in red and blue, respectively.

**Figure 4:**
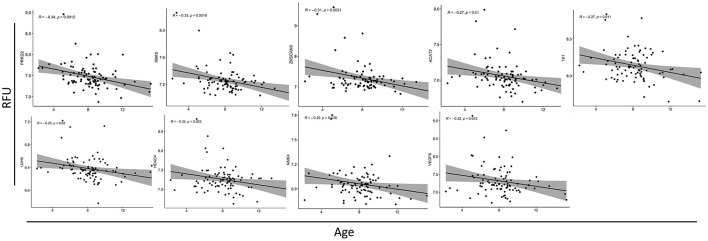
Correlation analysis between autoantibodies relative fluorescence unit (RFU) values and the age of ASD subjects. Scatterplots of all the significantly correlated autoantibodies. The correlation coefficients (*R*^2^) and associated *p*-values are shown in each figure (top left).

Autoantibody screening was performed against a total of 1,623 antigens, 534 of which were retained after filtration of antigens whose signals highly correlated across the cohort with the negative control probes (as described in Section 2). Blood profiling of the humoral response to self-antigens identified 29 autoantibodies with differential expression (unadjusted *p-value* ≤ 0.05) in ASD individuals compared to HCs (Table 2). The significant autoantibody expression levels are depicted on the heatmap (Figure 3A). The signals of four autoantibodies were increased (PSIP1, NAP1L3, GNB3, and ZMYM2), while those of 25 autoantibodies were decreased in ASD (CTNNB1, MAPK1, SSNA1, LDHB, GOT1, etc.; Figure 3B; Table 2). The top upregulated and downregulated autoantibodies are shown in boxplots (Supplementary Figures S3A, B). Furthermore, correlation analysis was performed on all detected autoantibodies and the ASD severity score (ADOS-2), a tool used to assess the severity level of ASD at different developmental stages. Out of all autoantibodies, 22 exhibited a significant weak correlation with the ADOS-2 score (Supplementary Table S2). However, only one protein (GOT1) from the differentially expressed autoantibodies list exhibited a significant weak positive correlation with the ADOS-2 score (Supplementary Figure S3C; Table 2). Moreover, correlation analysis was performed on the differential autoantibodies to examine the dynamic changes between the significant autoantibodies' concentration and age. Interestingly, 25 out of 29 autoantibodies negatively correlated with age, 9 of which were significantly correlated (Figure 4; Supplementary Table S2).

**Table 2:** Differentially expressed autoantibodies list.

**Protein symbol**	**Protein name**	**Fold change (FC)**	***Limma p-*value**	**Protein expression in the brain**	***p-*value, correlation coefficient**
ACAT2	Acetyl-CoA acetyltransferase 2	0.84	0.011	(+)	0.50, 0.096
APPL1	Adaptor protein, phosphotyrosine interacting with PH domain and leucine zipper 1	0.88	0.026	(+)	0.08, −0.24
CARHSP1	Calcium-regulated heat-stable protein 1	0.84	0.010	(+)	0.13, 0.21
CTNNB1	Catenin beta 1	0.78	0.056	Highest expression in Cereb^*^	0.82, 0.03
ECI2	Enoyl-CoA delta isomerase 2	0.89	0.015	(+)	0.93, 0.01
EPHA3_ext	EPH receptor A3	0.92	0.042	(+)	0.56, −0.08
GGPS1	Geranylgeranyl diphosphate synthase 1	0.83	0.025	Low expression in BG, Hipp, and CC^*^	0.53, −0.08
GIPC1	GAIP interacting protein C-terminus	0.88	0.005	(+)	0.95, −0.008
GNB3	Guanine nucleotide-binding protein G	1.05	0.037	(+)	0.62, −0.07
GOT1	Glutamic-oxaloacetic transaminase 1	0.85	0.017	(+)	0.04, 0.288
HDAC4	Histone deacetylase 4	0.85	0.026	(+)	0.07, −0.25
ILF2	Interleukin enhancer binding factor 2	0.86	0.042	(+)	0.97, 0.004
KRT8	Keratin 8	0.81	0.018	NA	0.84, −0.028
LDHB	Lactate dehydrogenase B	0.87	0.012	(+)	0.25, 0.165
MAPK1	Mitogen-activated protein kinase 1	0.73	0.045	(+)	0.28, −0.15
NAP1L3	Nucleosome assembly protein 1 like 3	1.61	0.057	RNA levels are high in the brain	0.39, −0.12
NME4	NME/NM23 nucleoside diphosphate kinase 4	0.92	0.020	(+)	0.21, 0.17
PAK2	P21 (RAC1)-activated kinase 2	0.91	0.017	Low expression in CC and Endoth^*^	0.99, −0.0005
PRKD2	Protein kinase D2	0.88	0.025	(+)	0.17, −0.19
PSIP1	PC4 and SRSF1 interacting protein 1	1.68	0.045	(+)	0.33, 0.13
RBKS	Ribokinase	0.89	0.019	Low expression in Cereb, BG, and Hipp^*^	0.31, 0.14
SORD	Sorbitol dehydrogenase	0.89	0.011	NA	0.44, 0.11
SSNA1	SS nuclear autoantigen 1	0.79	0.027	NA	0.88, 0.020
TK1	Thymidine kinase 1	0.91	0.047	Low expression in Hipp and CC^*^	0.66, 0.062
TPI1	Triosephosphate isomerase 1	0.92	0.044	(+)	0.75, 0.045
VEGFB	Vascular endothelial growth factor B	0.843	0.043	NA	0.23, −0.17
YWHAE	Tyrosine 3-monooxygenase/tryptophan 5-monooxygenase activation protein epsilon	0.88	0.052	(+)	0.30, 0.14
ZKSCAN3	Zinc finger with KRAB and SCAN domains 3	0.84	0.04	(+)	0.73, 0.048
ZMYM2	Zinc finger MYM-type containing 2	1.05	0.04	(+)	0.38, 0.12

^*^Cereb, cerebellum; BG, basal ganglia; Hippo, hippocampus; CC, cerebral cortex; Endoth., endothelium.

### 3.3. Gene ontology and network analysis revealed enrichment in autoantibodies against metabolic pathways and neuronal proteins

**Figure 5:**
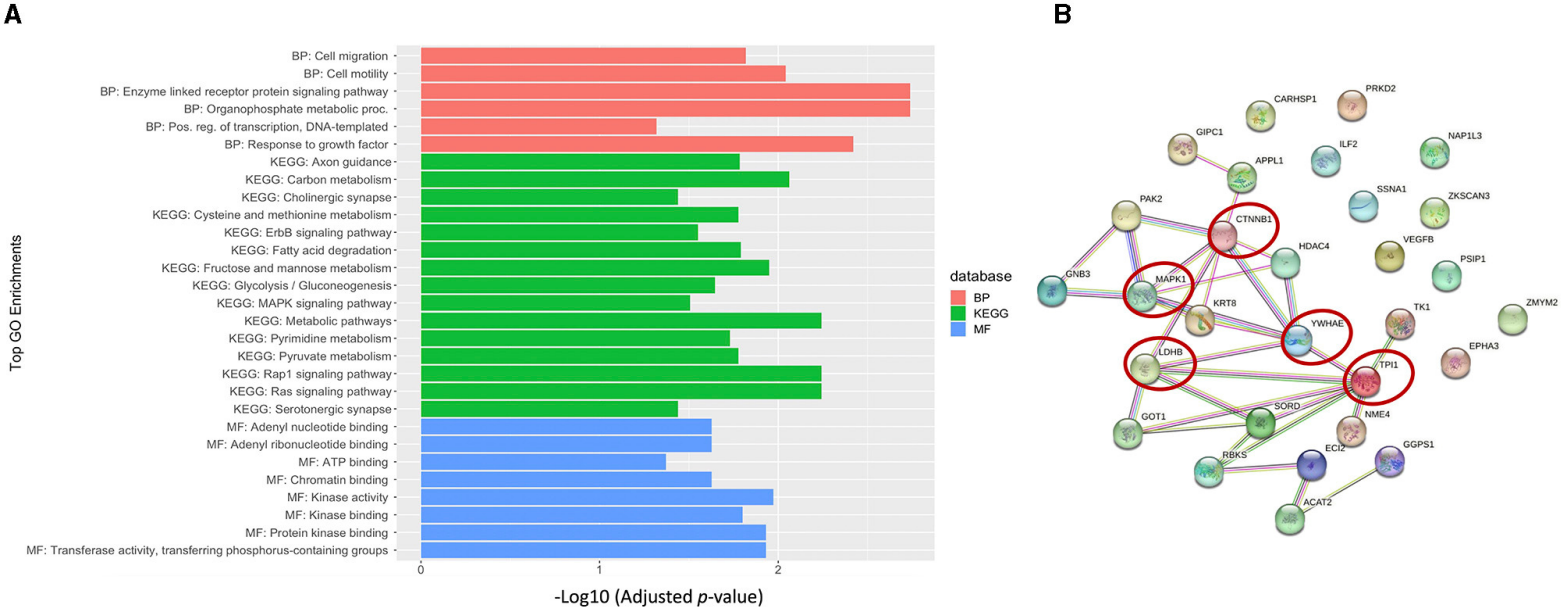
Biological relevance of the differential autoantibodies. **(A)** Top GO terms of the differential autoantibody's proteins; BP, biological process; MF, molecular function. **(B)** STRING protein-protein interaction (PPI) network and hub proteins list are highlighted with red circles.

To better understand the biological relevance between the identified autoantibodies, gene ontology (GO) enrichment analysis was performed on the corresponding proteins of those autoantibodies listed in Table 2. The results revealed that the majority of the autoantibodies were raised against proteins expressed in metabolic pathways. The KEGG database showed enrichment in multiple metabolic pathways, including carbon metabolism, glycolysis and gluconeogenesis, and fructose/mannose metabolism, in addition to pathways involved in amino acid metabolisms such as cysteine and methionine pathways. Nine corresponding proteins were involved in the metabolic pathways, and those proteins are NME4, TPI1, LDHB, GOT1, ACAT2, SORD, GGPS1, TK1, and RBKS. Another enriched category was signaling pathways such as Ras, Rap1, ErbB, and MAPK as autoantibodies against MAPK1, GNB3, VEGFB, PARK2, CTNNB2, and PRKD2 were differentially expressed in ASD. Furthermore, autoantibodies against neuronal processes such as axonal guidance were enriched, and included three corresponding proteins, EPHA3, MAPK1, and PAK2, as well as glutamatergic and cholinergic synapses, which included MAPK1 and GNB3 proteins. No enrichment was found at the specific cell component. However, enrichment in molecular functions was evident in adenyl nucleotide binding, kinases, phosphate group transfer, and chromatin binding. Biological processes included enrichment in enzyme-linked receptor protein signaling pathway, organophosphate metabolic process, cell motility, migration, and positive regulation of transcription (Figure 5A; Supplementary Table S3). Moreover, autistic disorder was among disease alliance enriched terms, and included MAPK1 and CTNNB1 proteins (FDR = 0.044). Protein–protein interaction (PPIs) network of the corresponding proteins is displayed in Figure 5B, along with the hub proteins.

### 3.4. Autoantibodies cross-reactivity check to ensure specific epitope recognition of the identified autoantibodies

**Figure 6:**
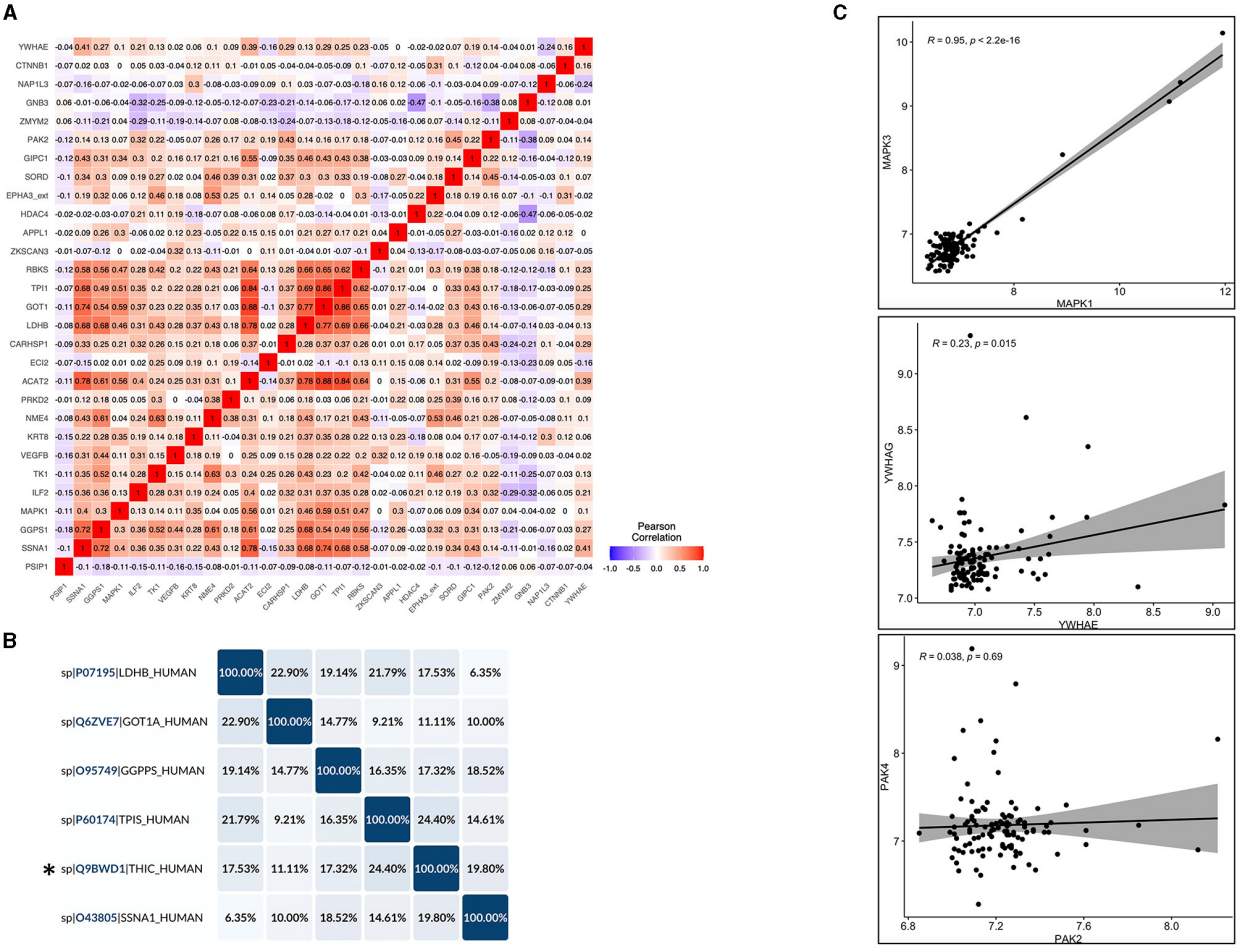
Cross-reactivity checks for the identified differential autoantibodies. **(A)** A correlation matrix of the differential autoantibodies using Pearson's correlation, red = high and blue = low. **(B)** Identity matrix, generated from aligning the corresponding protein sequences of the highly correlated autoantibodies (*R*^2^ ≥ 0.7) using Uniprot, *THIC corresponds to ACAT2. **(C)** Pearson correlation of the corresponding protein isoforms with >50% homology, correlation coefficients (*R*^2^), and associated *p*-values are shown in each figure (top left).

In order to check the cross-reactivity, correlation analysis was performed on the 29 autoantibodies identified by Limma analysis. Subsequently, sequence alignment and identity analysis were performed on the highly correlated autoantibodies (*R*^2^ > 0.7). The correlation matrix (Figure 6A) showed that ACAT2 correlated with TPI1, GOT1, and LDHB; SSNA1 correlated with GOT1, ACAT2, and GGPS1; LDHB correlated with GOT1 only; and GOT1 correlated with TPI1 only. Alignments and identity analysis of those protein sequences revealed that all proteins are <25% identical, and the fact that those proteins are not even isoforms indicates a low chance of cross-reactivity (Figure 6B; Supplementary Figure S4). Thus, this observed correlation might stem from a biological/functional relevance rather than antibody cross-reactivity *per-se*.

Another source of possible cross-reactivity is sequence homology between protein isoforms. Therefore, the Uniprot protein database was used to check sequence homology between the differential autoantibodies proteins (*n* = 29) and their isoforms that were also tested in the same iOme panel. Three proteins showed >50% homology with their isoforms in the iOme panel: MAPK1 and MAPK3 were 83% identical, and PAK2 and PAK4 and also YWHAE and YWHAG were ~64% identical. However, proteins with high homology were not found to be differentially expressed, and they exhibited a low correlation with their significant counterparts except for MAPK1 and MAPK3, which were highly correlated (*R*^2^ = 0.95), see Figure 6C. Therefore, based on the correlation and homology analysis findings, cross-reactivity cannot be ruled out for MAPK1 and MAPK3; however, protein co-expression might explain the high correlation. Overall, most of the identified autoantibodies are less likely to exhibit cross-reactivity except for MAPK1's autoantibody, which might cross-react with MAPK3. Although sequence similarity was excluded among the candidate antigens, conformational molecular mimicry cannot be excluded.

## 4. Discussion

Several studies have identified the presence of autoantibodies against fetal brain and neuronal proteins in ASD children and their mothers (Piras et al., 2014; Abou-Donia et al., 2019; Mazón-Cabrera et al., 2019; Ramirez-Celis et al., 2021). The implication of those autoantibodies and whether or not they interfere with normal developmental and biological processes are still unknown. In our study, we performed quantitative autoantibody profiling using 1,623 antigens for ASD subjects and healthy controls in an effort to identify differentially expressed autoantibodies and to elucidate the biological relevance of those autoantibodies in ASD. An aggregate of 534 autoantibodies were identified across the screened samples, 29 of which were differentially expressed in ASD (Figure 3; Table 2). The highest FC differences between ASD cases and HCs were observed in two proteins, PSIP1 (FC ~ 1.7) and NAP1L3 (FC ~ 1.6), which were upregulated in ASD. PSIP1 is a nuclear protein and a transcription coactivator that is involved in neurogenesis processes such as neuroepithelial cell differentiation (Chylack et al., 2004). Furthermore, NAP1L3 is also a nucleosome protein and a histone chaperone that plays a crucial role in epigenetic transcription regulation (Heshmati et al., 2018). Interestingly, both proteins have chromatin-binding abilities and were enriched in the chromatin-binding term in GO analysis. Moreover, other corresponding proteins also enriched in chromatin binding GO term are HDAC4, CTNNB1, and ZKSCAN3; however, unlike PSIP1 and NAP1L3, these proteins exhibited reduced expression in ASD compared to HCs. HDAC4 encodes for histone deacetylase 4, a protein involved in the deacetylation of the core histones, thus regulating transcription (Wang et al., 2014). Deletion in HDAC4 has been reported in ASD (Williams et al., 2010). CTNNB1 is a beta-catenin protein involved in the Wnt pathway in the neurons. Interestingly, according to the Simons Foundation Autism Research Initiative (SFARI) database, CTNNB1 nonsense and missense mutations have been reported in ASD subjects (O'Roak et al., 2012). No evidence has been reported on the relationship between ASD pathogenesis and ZKSCAN3.

Interestingly, differential levels of autoantibodies against proteins with neuronal-related function were also detected in our cohort. For instance, EPHA3, MAPK1, PAK2, and SSNA1 are important for the axonal guidance process during neurogenesis (Brown et al., 2000; Hammarlund et al., 2009; Ding et al., 2015; Lawrence et al., 2021). Moreover, MAPK1 is also involved in cholinergic and serotonergic synapse (as shown in our GO analysis). MAPK1 is highly expressed in the human brain and is involved in various processes such as synaptic plasticity, learning, and memory (Thomas and Huganir, 2004). Although we were not able to rule out the cross-reactivity between MAPK1 and MAPK3 due to their high correlation and homology, MAPK1 and MAPK3 are both as important in the MAPK/ERK cascade and participate in the same biological processes; thus, they might both correlate due to their biological relevance. Moreover, recent studies showed that MAPK1 and MAPK3 can generally compensate for each other in most tissues and have a very similar expression pattern (Upadhya et al., 2013; Frémin et al., 2015). MAPK1 has a critical role in multiple signaling pathways such as MAPK, ErbB, Ras, and Rap1 pathways, as shown in GO enrichment analysis (Figure 5A; Upadhya et al., 2013).

Another pathway of autoantibodies that was found to be differentially expressed in nine of its proteins is the metabolic pathway, which includes amino acids and fatty acid metabolism, gluconeogenesis, and glycolysis. Amongst these autoantibodies is one that corresponds to the LDHB protein. Autoantibodies against this protein have been reported to be upregulated in ASD children and their mothers (Braunschweig et al., 2013; Ramirez-Celis et al., 2021) contrary to our cohort, where it was found downregulated. This discrepancy could potentially stem from technical differences as the aforementioned studies utilized western blotting for detection, while our study employed iOme microarray. In addition, both western blotting and iOme microarray entail different normalization approaches. LDHB is an enzyme that catalyzes the conversion of lactate to pyruvate, a critical energy substrate. A recent study using an LDHB knock-out mouse model showed that LDHB deficiency triggers gliosis, neuroinflammation, and neurodegeneration in the brain (Park et al., 2022). Only one autoantibody, corresponding to GOT1 protein, exhibited a significant but weak correlation with the ADOS-2 scores. Our GO analysis revealed that GOT1 is involved in multiple metabolic processes, such as cysteine and methionine metabolism pathways. Paradoxically, our findings revealed a negative correlation between age and the majority of the identified autoantibodies. This finding suggests that there is an overall reduction in autoantibody signal as ASD children age increases. Moreover, our study could not confirm the source of the identified autoantibodies; therefore, more longitudinal studies are needed to validate and properly curate the origins and the dynamic changes of these autoantibodies.

Considering the existing reports on ASD, autoantibodies against glyceraldehyde-3phosphate dehydrogenase (GAPDH) have previously been identified (Mazón-Cabrera et al., 2023). Unfortunately, this specific autoantigen is not included in the Sengenics autoantigen panel. On the other hand, autoantibodies against stress-induced phosphoprotein 1 (STIP1) have been previously identified in ASD (Braunschweig et al., 2013), and the autoantigen is present in the Sengenics panel; however, our results revealed no statistically significant difference in its expression. It is crucial to highlight that autoantibodies targeting these proteins were primarily identified in mothers of ASD children rather than the children themselves. Another study highlighted the significance of GFAP and SNCA autoantibodies in ASD subjects (Abou-Donia et al., 2019). However, our study indicated that GFAP did not yield statistically significant results, and SNCA did not pass the NCF threshold, leading to its exclusion from the statistical analysis. These previous studies predominantly employed Western blotting for autoantibody detection, and this methodological disparity could potentially explain the challenges in reproducing these findings within our cohort.

It is noteworthy that the majority of the identified autoantibodies in this study were overall downregulated in ASD compared to HCs. The cause of this downregulation and how it might be involved in ASD pathogenesis requires further investigation. However, the overall downregulation of autoantibodies in ASD does not indicate an underlying autoimmune pathology. The presence of higher concentrations of autoantibodies does not necessarily signify pathology; on the contrary, they might serve a protective role (Shoenfeld and Toubi, 2005). For instance, autoantibodies against dsDNA were negatively correlated with lupus nephritis in systemic lupus erythematosus patients; in other words, the higher the antibody titer, the lower the severity of the disease (Witte, 2008). Furthermore, anti-TCR antibodies that circulate in myasthenia gravis patients' peripheral blood are reactive against a specific T-cell population that expresses the Vβ5.1 TCR gene. This gene is largely responsible for the production of anti–acetylcholine receptor antibodies in patients with myasthenia gravis, thus inhibiting the proliferation and IFN-γ production of those cells and reducing the severity of the condition (Jambou et al., 2003). Another example of the protective role of autoantibodies against self-antigens was tested in the Alzheimer's disease mouse model, where administrating antibodies against β-amyloid, a protein responsible for plaque formation in Alzheimer's disease, resulted in reduced amyloid plaque formation in the brain and attenuated other pathological features of Alzheimer's disease (Schenk et al., 1999). This hypothesis was observed in humans as naturally occurring autoantibodies against β-amyloid were reduced in the cerebrospinal fluid of Alzheimer's disease patients compared to healthy controls (Du et al., 2001).

The majority of the identified differentially expressed autoantibodies correspond to cytosol and nuclear proteins; hence, they are expressed intracellularly. It is important to mention that autoantibodies production is not limited to extracellular and cell-surface proteins, but rather, there have been some instances where autoantibodies are generated against intracellular proteins. For example, autoantibodies against double-strand DNA are known to be present in systematic lupus erythematosus disorder (Rahman and Isenberg, 2008); autoantibodies against SSA and SSB are known to be present in Sjögren's syndrome (Routsias and Tzioufas, 2010); and autoantibodies against myeloperoxidase (P-ANCA) and proteinase 3 (p-ANCA) are present in vasculitides (Chen and Kallenberg, 2010). The mechanism by which the immune system is exposed to those antigens is thought to be through apoptosis, where the intracellular content of the cell is expressed on the surface of the apoptotic body (Casciola-Rosen et al., 1999). Another possible mechanism is antigen mimicry; for example, the Ro60 antigen, recognized by anti-Ro60 in systematic lupus erythematosus, shares similarities with a ribonucleoprotein found in *Mycobacterium smegmatis* as well as with a specific amino acid sequence (58–72) in the Epstein–Barr nuclear antigen-I (EBNA-I), a latent viral protein expressed by Epstein–Barr virus (McClain et al., 2005). However, the exact mechanism of how this takes place in the context of ASD is unknown.

Other examples of autoantibody targets that have been reported to have a protective role are autoantibodies against chemokines and cytokines. These autoantibodies protect against disease models such as multiple sclerosis and rheumatoid arthritis, thus confirming the importance of beneficial autoimmunity in controlling harmful self-directed immune responses (Wildbaum et al., 2003). In addition, the presence of natural autoantibodies that arise with no prior exposure to antigen plays a crucial role in clearing apoptotic cell bodies by marking them for macrophage-mediated phagocytosis (Elkon and Casali, 2008). These natural autoantibodies recognize intracellular antigens such as nucleosomes and phosphatidylserine in the apoptotic cell body.

To the best of our knowledge, our study is the first to perform autoantibody profiling using a large number of antigens for ASD individuals. However, it is important to acknowledge that our study faced limitations, primarily related to sample size, as the number of the HCs group was smaller than that of the cases. Subsequent studies are needed to validate our findings through larger sample sizes and to explore the role of these autoantibodies in the pathogenesis of ASD. Although we excluded sequence similarity among the candidate antigens, the potential for conformational molecular mimicry remains. Furthermore, our study uncovered a negative correlation between the majority of the identified autoantibodies and the age of ASD individuals. As a result, future longitudinal studies should include samples from ASD children to better elucidate this correlation and to validate our findings.

## 5. Conclusion

In conclusion, this study aimed to explore autoantibody reactivity against self-antigens in the serum of ASD individuals using a high-throughput assay. We identified 29 autoantibodies that were differentially expressed in ASD. These differential autoantibodies were reactive against proteins involved in axonal guidance, synaptic function, amino acid metabolism, fatty acid metabolism, and chromatin binding. In addition, we detected an overall decline in autoantibody concentration as ASD children's ages increased. This study was limited by the imbalanced number of participants and the significant difference, in terms of age and sex, between the studied groups. Therefore, validating our findings using a different cohort with a larger sample size is warranted. Furthermore, additional longitudinal investigations are necessary to accurately ascertain the sources and evolving patterns of these autoantibodies.

## Data availability statement

The original contributions presented in the study are included in the article/Supplementary material, further inquiries can be directed to the corresponding author.

## Ethics statement

The studies involving humans were approved by Qatar Biomedical Research Institute (QBRI). The studies were conducted in accordance with the local legislation and institutional requirements. Written informed consent for participation in this study was provided by the participants' legal guardians/next of kin.

## Author contributions

AM: conceptualization, data curation, formal analysis, and writing—original draft. HE: data curation and formal analysis. KL: formal analysis. HA: project administration, resources, and writing—review and editing. FA-S: data curation (ADOS-2 scores) and writing—review and editing. JD, NA, ME, and OA: conceptualization and writing—review and editing. AA: conceptualization. LS: conceptualization, project coordination, and writing—review and editing. SA: project coordination. JB: writing—review and editing. OE-A: conceptualization, study design, supervision, and writing—review and editing. All authors have read and agreed to the published version of the manuscript.
