# Supplementary material for: High-throughput autoantibody screening identifies differentially abundant autoantibodies in autism spectrum disorder
Source: Front Mol Neurosci. 2023 Oct 16;16:1222506. doi: 10.3389/fnmol.2023.1222506 (PMC10613655; doi:10.3389/fnmol.2023.1222506)
Supplement: Supplementary file 1 [file Data_Sheet_1.ZIP › Supplmentary_Material_Updated/Table S1.pdf]

Protein Symbol	Protein Name	Other names	Uniprot Accession	ProteinAtlas	Protein Class	GenBank Accession	Associated Diseases	Open Target Link	PPI	Subcellular Localisation	Transmembrane	Druggable /Non-druggable	Ligand Links	i-Ome Array
A1BG	HUMAN Alpha-1B-glycoprotein	A1BG	P04217	ENSG0000(	Others	AC10642	Colorectal cancer, Breast carcinoma, Pancreatic carcinoma, Lung carcinoma, Cervical carcinoma, Non-small cell lung carcinoma, Down syndrome, Adenocarcinoma, Amyotrophic lateral sclerosis, Glioblastoma	https://www.targetvalidation.org/target/ENSG00000121410/asso ciations	CRISP3	Secreted		No		✓
AAK1	HUMAN AP2-associated protein kinase 1	DKFZp686K16132, KIAA1048	Q2M2I8	ENSG0000(	Kinase	AC136007	Astrocytoma, Ependymoma, Glioblastoma multiforme, Osteosarcoma, Breast carcinoma, Large cell lung carcinoma, Amyotrophic lateral sclerosis, Hepatitis C infection, Septicemia, Sepsis	https://www.targetvalidation.org/target/ENSG00000115977/asso ciations	CDK1 NUMB CDK5R1 AP2M1 AP1M1	Cell membrane		Yes	https://www.bindingdb.org/uniprot/Q2M2I8	✓
AARS	HUMAN Alanine--tRNA ligase, cytoplasmic	CMT2N, SYAC	P49588	ENSG0000(	Others	AC012184	Autosomal dominant Charcot-Marie-Tooth disease type 2N, Early infantile epileptic encephalopathy, Charcot-Marie-Tooth disease, Alopecia, Marie Unna hereditary hypotrichosis, Neuropathy, Skeletal muscle atrophy, Barth syndrome, Pili torti, Hypotrichosis simplex, Distal sensory impairment	https://www.targetvalidation.org/target/ENSG00000090861/asso ciations		Cytoplasm		Yes	https://www.bindingdb.org/uniprot/P49588	✓
ABCF1	HUMAN ATP-binding cassette sub-family F member 1	ABC50, EST123147	Q8NE71	ENSG0000(	Cancer,Cancer-Antigen	AL662800	Breast carcinoma, Acute myeloid leukemia, Dengue disease, Pancreatitis, Colorectal disease, Diabetes mellitus, Pneumonia, Ankylosing spondylitis, Schizophrenia, Thyroid associated opthalmopathy	https://www.targetvalidation.org/target/ENSG00000204574/asso ciations	CSNK2A1	Cytoplasm		No		✓
ABCF3	HUMAN ATP-binding cassette sub-family F member 3	EST201864	Q9NUQ8	ENSG0000(	Cancer,Cancer-Antigen	AK290106	Cervical carcinoma	https://www.targetvalidation.org/target/ENSG00000161204/asso ciations				No		✓
ABI1	HUMAN Abl interactor 1	ABI-1, E3B1, SSH3BP1	Q8IZP0	ENSG0000(	Cancer	AF540955	Adenocarcinoma, Melanoma, Cutaneous melanoma, Colonic neoplasm, Squamous cell carcinoma, Leukemia, Breast carcinoma, Ovarian serous adenocarcinoma, Gastric adenocarcinoma, Prostate carcinoma	https://www.targetvalidation.org/target/ENSG00000136754/asso ciations	NCKAP1 BAIAP2 CYFIP2 PIK3R3 PAK2 EPS8 SOS2 SOS1 ABL2 MAPK1 PIK3R1 MAPK3 NCF1 ITGA4 ABL1 PIK3R2 WASF2 SOS1 ABL1 CBL NCK2 NCKAP1 EPS8 ENAH CYFIP2	Cytoplasm		No		✓
ABL2	HUMAN Abelson tyrosine-protein kinase 2	ABLL, ARG	P42684	ENSG0000(	Kinase,Cancer	FJ542285	Lung carcinoma, Breast carcinoma, Melanoma, Small cell lung carcinoma, Prostate carcinoma, Renal carcinoma, Cardiomyopathy, Alzheimer's disease, Acute promyelocytic leukemia, Colorectal adenocarcinoma	https://www.targetvalidation.org/target/ENSG00000143322/asso ciations	CASP8 PRKG1 RIN1 CRK SRC BCR PDGFRB HCK ABL1 HCK DBN1 EGFR CTTN PSMA7 POLR2B NOD2 PDGFRB RAPH1 RIN1 ABI1 CRK SORBS2 HRAS IKBKB CDK7 ITGB1 WASL ARHGAP35 CAT ERBB3 ERBB2 ERBB4 LGALS3 CBL SIVA1	Cytoplasm, cytoskeleton		Yes	https://www.bindingdb.org/uniprot/P42684	✓
ABT1	HUMAN Activator of basal transcription 1	ABT1	Q9ULW3	ENSG0000(	Others	BC048812	Breast carcinoma, Gastric neoplasm	https://www.targetvalidation.org/target/ENSG00000146109/asso ciations	TBP	Nucleus		No		✓
ACAT2	HUMAN Acetyl-CoA acetyltransferase, cytosolic	THIC, ACTL	Q9BWD1	ENSG0000(	Cancer,Cancer-Antigen	AL135914	Breast carcinoma, Lung carcinoma, Large cell lung carcinoma, Pancreatic carcinoma, Melanoma, Muscle hypotonia, Chorea, Renal cell carcinoma, Idiopathic pulmonary fibrosis, Glioblastoma multiforme	https://www.targetvalidation.org/target/ENSG00000120437/asso ciations	CDX2 CSNK2A1	Cytoplasm		Yes	https://www.drugbank.ca/biodb/polypeptides/Q9BWD1	✓
ACPP	HUMAN Prostatic acid phosphatase	ACP-3, ACP3, PAP, PPAP	P15309	ENSG0000(	Cancer	X52174	Renal cell carcinoma, Li-Fraumeni carcinoma, Squamous cell carcinoma, Clear cell renal carcinoma, Psoriasis, Juvenile arthritis, Prostatitis, Prostate carcinoma, Esophageal squamous cell carcinoma, Glioma	https://www.targetvalidation.org/target/ENSG00000014257/asso ciations	AR ERBB2 ERBB2	Secreted		Yes	https://www.bindingdb.org/uniprot/P15309	✓

ACRBP	HUMAN Acrosin-binding protein	CT23, OY-TES-1, SP32	Q8NEB7	ENSG0000(	Others	NA	Azoospermia, Teratozoospermia, Ovarian carcinoma, Pancreatic carcinoma, Gastric carcinoma, Juvenile arthritis, Breast carcinoma, Epilepsy, Hepatocellular carcinoma, Squamous cell carcinoma	https://www.targetvalidation.org/target/ENSG00000111644/associations		Secreted		No		✓
ACSL6	HUMAN Long-chain-fatty-acid--CoA ligase 6	ACS2, FACL6, KIAA0837, LACS5	Q9UKU0	ENSG0000(	Cancer	DQ083031	Lung carcinoma, Melanoma, Kidney neoplasm, Colorectal adenocarcinoma, Basal cell carcinoma, Head and Neck squamous cell carcinoma, Esophageal carcinoma, Schizophrenia, Polycythemia Vera, Amphetamine-related disorder	https://www.targetvalidation.org/target/ENSG00000164398/associations	PHYH	Mitochondrion outer membrane	Transmembrane	No		✓
ACTA1	HUMAN Actin, alpha skeletal muscle	ACTA, NEM3, ACTS	P68133	ENSG0000(	Signalling	CR536516	Muscular disease, Congenital myopathy with excess of thin filaments, Congenital myopathy, Nemaline myopathy, Congenital fiber-type disproportion myopathy, Zebra body myopathy, Oculopharyngodistal myopathy, Becker muscular dystrophy, Congenital myasthenic syndrome, , Hyaline body myopathy	https://www.targetvalidation.org/target/ENSG00000143632/associations	TLN1 TTN TNNI3K MYLK PAK1 PRKCD PRKCE AIF1 SMTN CNN1 VASP IQGAP1 GLRX SPRR1A ADSS DNASE1 CFL1 S100A1 TNNI3 VCL ITGA2 PRKCA VAV1 DMD VIL1 PFN1 TPM3 GSN ISG15 TP53 GC SPTA1 EGFR ABL1 GAS7	Cytoplasm, cytoskeleton		Yes	https://www.drugbank.ca/biodb/polypeptides/P68133	✓
ACTB	HUMAN Actin, cytoplasmic 1	ACTB	P60709	ENSG0000(	Cancer,Cancer-Antigen	AY582799	Lissencephaly, Ptosis, Developmental malformations-deafness-dystonia, Baraitser-Winter syndrome, Auditory system disease, Dystonia, Pachygyria, Blepharoptosis, Melanoma, Sarcoma	https://www.targetvalidation.org/target/ENSG00000075624/associations	WASF2 CFL2 RUVBL1 RUVBL2 BAIAP2 WASF3 MRGBP TSC1 WASF1 LASP1 PRKCD ACTC1 ACTG1 RAC1 SYNJ2BP CLNS1A CAPZA1 HSD17B4 CCT4 MMP14 PCYT1A CSNK1A1 IQGAP1 MYH9 PFN2 CORO1A LMOD1 CTGF CFL1	Cytoplasm, cytoskeleton		No		✓
ACTL7B	HUMAN Actin-like protein 7B	Tact1, ACL7B	Q9Y614	ENSG0000(	Others	AL359692	Azoospermia, Endometrial neoplasm, Colorectal cancer, Familial dysautonomia, Colorectal cancer, Response to bronchodilator, Forced expiratory volume	https://www.targetvalidation.org/target/ENSG00000148156/associations		Cytoplasm, cytoskeleton		No		✓
ACTL8	HUMAN Actin-like protein 8	CT57	Q9H568	ENSG0000(	Others	BC028909	Malignant mesothelioma, Colorectal adenocarcinoma, Breast carcinoma, T-cell acute lymphoblastic leukemia, Glioblastoma, Diabetes mellitus, Autism	https://www.targetvalidation.org/target/ENSG00000117148/associations		Cytoplasm, cytoskeleton		No		✓
ACTR2	HUMAN Actin-related protein 2	ARP2	P61160	ENSG0000(	Kinase	AC007318	Melanoma, Lung carcinoma, Chronic obstructive pulmonary disease, Osteosarcoma, Wiskott-Aldrich syndrome, Schizophrenia, Prostate carcinoma, Forced expiratory volume, Response to bronchodilator	https://www.targetvalidation.org/target/ENSG00000138071/associations	WASF2 MAP3K14 ARPC1A WASF1 CTTN ACTC1 ACTR3 ARPC4 WAS BMP7 BMP2 GDF11 MED14 ARPC5 ARPC3 ARPC1B WASL ARPC1B ACVR1B NLRP2 IQGAP2 ARPC2 ARPC4 ACTR3 ARPC1A	Cytoplasm, cytoskeleton		Yes	https://www.drugbank.ca/biodb/polypeptides/P61160	✓
ACVR1	HUMAN Activin receptor type-1	ACVR1A, ACVRLK2, ALK2, SKR1	Q04771	ENSG0000(	Kinase,Cancer	NA	Melanoma, Fibrodysplasia ossificans progressiva, Lung carcinoma, Hepatocellular carcinoma, Colorectal adenocarcinoma, Myositis ossifican, Glioma, Astrocytoma, Clear cell renal carcinoma	https://www.targetvalidation.org/target/ENSG00000115170/associations	NUAK2 UHMK1 NEK8 AMHR2 BMPR2 ACVR2B PAK1 INHBC BMPR1A ACVR2A BMP7 BMP2 INHBB INHBA CDK14 SMAD6 SMAD7 IKBKB FKBP4 RPS6KA2 FANCL STAT1 RAB33B NKIRAS1 SMAD1 AJUBA SMAD5 SQSTM1 VDR	Membrane, Single-pass type I membrane protein	Transmembrane	Yes	https://www.bindingdb.org/uniprot/Q04771	✓
ACVR1C	HUMAN Activin receptor type-1C	ALK7	Q8NER5	ENSG0000(	Kinase	AF525681	Lung carcinoma, Large cell lung carcinoma, Breast carcinoma, Astrocytoma, Glioblastoma multiforme, Ovarian carcinoma, Epithelial ovarian cancer, Liver carcinoma	https://www.targetvalidation.org/target/ENSG00000123612/associations	NODAL TDGF1 NODAL INHBB	Membrane	Transmembrane	No		✓

ACVR2A	HUMAN Activin receptor type-2A	ACTRII, ACVR2	P27037	ENSG0000(	Kinase,Cancer,Cancer-Antigen	D31770	Colonic neoplasm, Hepatocellular carcinoma, Pancreatic carcinoma, Rectal adenocarcinoma, Colorectal adenocarcinoma, Melanoma, Lung carcinoma, Prostate carcinoma, Gastric adenocarcinoma, Colonic neoplasm	https://www.targetvalidation.org/target/ENSG00000121989/associations	ERBIN ACVR1 INHBC GDF5 ACVR1B BMP7 ENG TDGF1 INHBB INHBA TGFBR3 ACVR1B SYNJ2BP ENG ACVR1 ACVRL1 BMP2 MAGI2 GDF5 INHBA BMP7 GDF9 BMP6 IGSF1 INHBB INHBC	Membrane, Single-pass type I membrane protein	Transmembrane	Yes	https://www.bindingdb.org/uniprot/P27037	✓
ADAM2	HUMAN Disintegrin and metalloproteinase domain-containing protein 2	CT15, FTNB, PH-30b, PH30	Q99965	ENSG0000(	Others	AJ13005	Azoospermia, Breast carcinoma, Deafness-infertility syndrome, Male infertility withTeratozoospermia due to single gene mutation, Non-syndromic male infertility due to sperm motility disorder, Facioscapulohumeral muscular dystrophy, Renal carcinoma, Prostate carcinoma	https://www.targetvalidation.org/target/ENSG00000104755/associations	CD9 CLGN	Membrane, Single-pass type I membrane protein	Transmembrane	No		✓
ADAM29	HUMAN Disintegrin and metalloproteinase domain-containing protein 29	CT73, ADA29	Q9UKF5	ENSG0000(	Others	AF134708	Breast carcinoma, Azoospermia, Venous thromboembolism, Cognitive impairment, Chronic lymphocytic leukemia, Esophagus squamous cell carcinoma, Melanoma, Triple-negative breast cancer	https://www.targetvalidation.org/target/ENSG00000168594/associations		Membrane, Single-pass type I membrane protein	Transmembrane	No		✓
ADCK1	HUMAN Uncharacterized aarF domain-containing protein kinase 1	ADCK1	Q86TW2	ENSG0000(	Kinase	BC058906	Resting heart rate, Breast carcinoma, Multiple sclerosis, Ovarian carcinoma, Adenocarcinoma, Multiple sclerosis, Peripheral neuropathy, HIV-1 infection	https://www.targetvalidation.org/target/ENSG00000063761/associations	RAB1A	Secreted		No		✓
ADD1	HUMAN Alpha-adducin	ADDA	P35611	ENSG0000(	Signalling	AK295457	Hereditary spherocytosis, Hereditary elliptocytosis, Congenital dyserythropoietic anemia, Platelet stoage pool disease, Wiskott-Aldrich syndrome, Liver carcinoma, Myocardial Infarction, Cerebral Palsy, Type II diabetes mellitus, Heinz body anemia	https://www.targetvalidation.org/target/ENSG00000087274/associations	ROCK1 PRKCD PRKCZ CALM1 PRKX CDK2 RFX1 PRKACA PRKCA CDK1 ATP1A1 SPTA1 ROCK2 PRKY CHEK1 ID2 SMARCC1 ID3	Cytoplasm, cytoskeleton, Cell membrane, Peripheral membrane protein, Cytoplasmic side		No		✓
ADSL	HUMAN Adenylosuccinate lyase	AMPS, PUR8	P30566	ENSG0000(	Cancer,Cancer-Antigen	AF067854	Disorder of purine metabolism, Inborn errors of metabolism, Adenylosuccinate lyase deficiency, Autistic disorder, Hermansky-Pudlak syndrome, MELAS, Canavan disease, Mammographic density measurement, Dense area measurement	https://www.targetvalidation.org/target/ENSG00000239900/associations				No		✓
AFF4	HUMAN AF4/FMR2 family member 4	AF5Q31, MCEF	Q9UHB7	ENSG0000(	Transcription factor,Cancer	AC004500	Lung carcinoma, Prostate carcinoma, Melanoma, Cornelia de Lange syndrome, Breast carcinoma, CHOPS syndrome, Obesity, Oral squamous cell carcinoma, Gastric adenocarcinoma, Cutaneous melanoma	https://www.targetvalidation.org/target/ENSG00000072364/associations	SIAH1 CDK9	Nucleus		No		✓
AFP	HUMAN Alpha-fetoprotein	HPAFP	P02771	ENSG0000(	Cancer	BC027881	Hereditary persistance of alpha-fetoprotein, Congenital deficiency iom alpha-fetoprotein, Hepatocellular carcinoma, Primary ovarian insufficiency, Breast carcinoma, Liver cirrhosis, Gastric neoplasm, Liver carcinoma, Lung carcinoma, Inborn errors of metabolism	https://www.targetvalidation.org/target/ENSG00000081051/associations	ZFHX3 CASP3 CCR5	Secreted		No		✓
AGR2	HUMAN Anterior gradient protein 2 homolog	AG2, HAG-2, PDIA17, XAG-2	O95994	ENSG0000(	Others	AF115926	Breast carcinoma, Adenocarcinoma, Breast adenocarcinoma, Ovarian carcinoma, Renal cell carcinoma, Liver carcinoma, Pancreatic neoplasm, Gastrointestinal neoplasm, Ulcerative colitis, Peutz-Jeghers syndrome	https://www.targetvalidation.org/target/ENSG00000106541/associations		Secreted		No		✓

AHSG	HUMAN Alpha-2-HS-glycoprotein	A2HS, FETUA, HSGA	P02765	ENSG0000(	Cancer	AK292751	Colorectal cancer, Bone disease, Inflammatory bowel disease, Hypobetalipoproteinemia, Inborn errors of metabolism, Calcinosis, Metabolic syndrome X, Endometriosis, Alzheimer's disease, Type II diabetes mellitus	https://www.targetvalidation.org/target/ENSG00000145192/associations	INSR TLR4 DCN	Secreted		No		✓
AICDA	HUMAN Single-stranded DNA cytosine deaminase	AID, ARP2, CDA2, HIGM2	Q9GZX7	ENSG0000(	Others	AY536516	Primary immunodeficiency, Hyper-IgM syndrome without susceptibility to opportunistic infectious, Lymphoma, Hyper-IgM syndrome type 2, Agammaglobulinemia, Blast phase, Alzheimer's disease, Fanconi anemia, Activated PIK3-delta syndrome, Acute lymphoblastic leukemia	https://www.targetvalidation.org/target/ENSG00000111732/associations	PRKDC PSME3 KPNA1 RPA1 PRKACA	Nucleus		No		✓
AIF1	HUMAN Allograft inflammatory factor 1	AIF-1, Em:AF129756.17, IBA1, IRT-1	P55008	ENSG0000(	Others	Y14768	Autosomal thrombocytopenia with normal platelets, Autosomal dominant macrothrombocytopenia, MYH9-related disease, Li-Fraumeni syndrome, Autoimmune disease, Hyperalgesia, Lewy body disease, Rheumatoid arthritis, Type I diabetes mellitus, Arteriosclerosis	https://www.targetvalidation.org/target/ENSG00000204472/associations	LCP1 ACTA1	Cytoplasm, cytoskeleton		No		✓
AIFM2	HUMAN Apoptosis-inducing factor 2	AMID, FLJ14497, PRG3	Q9BRQ8	ENSG0000(	Others	AK127353	Response to bronchodilator, FEV/FEC ratio, Hepatitis C infection, Breast carcinoma, Pancreatic carcinoma, Ovarian carcinoma, Lung carcinoma, Pancreatic carcinoma, Cystic fibrosis, Multiple myeloma	https://www.targetvalidation.org/target/ENSG00000042286/associations	TP53	Cytoplasm, Mitochondrion outer membrane, Membrane	Transmembrane	No		✓
AIFM3	HUMAN Apoptosis-inducing factor 3	AIFL	Q96NN9	ENSG0000(	Others	AC002470	Astrocytoma, Medulloblastoma, 3-hydropropylmercapturic acid measurement, Glioblastoma multiforme, Primitive neuroectodermal tumor, Colonic neoplasm, Liver carcinoma, Ulcerative colitis, Crohn's disease, Cholangiocarcinoma	https://www.targetvalidation.org/target/ENSG00000183773/associations		Mitochondrion		No		✓
AIM2	HUMAN Interferon-inducible protein AIM2	PYHIN4	O14862	ENSG0000(	Interleukin-Interferon	BC010940	Psoriasis, Leucocyte count, Tongue squamous cell carcinoma, Breast carcinoma, Li-Fraumeni syndrome, Agammaglobulinemia, Psoriasis, Tongue squamous cell carcinoma, Colon carcinoma, Melanoma	https://www.targetvalidation.org/target/ENSG00000163568/associations	PYCARD POPDC3	Nucleus, Cytoplasm		No		✓
AK1	HUMAN Adenylate kinase isoenzyme 1	KAD1	P00568	ENSG0000(	Kinase	BC001116	Hemolytic anemia due to adenylate kinase deficiency, Friedreich ataxia, Vitamin B12-responsive methylmalonic acidemia, Methylmalonic acidemia with homocystinuria, Nervous system disease, Myocardial infarction, Schizophrenia, Homocystinuria without methylmalonic aciduria, Myocardial ischemia, Hemolytic anemia, Sepsis	https://www.targetvalidation.org/target/ENSG00000106992/associations	AKT1	Cytoplasm		Yes		✓
													https://www.drugbank.ca/biodb/polypeptides/P00568	
AK2	HUMAN Adenylate kinase 2, mitochondrial	KAD2, ADK2	P54819	ENSG0000(	Kinase	AB005621	Reticular Dysgenesis, Severe combined immunodeficiency, Breast carcinoma, Melanoma, Colorectal adenocarcinoma, Lung carcinoma, De Vaal's syndrome, Lung adenocarcinoma, Juvenile dermatomyositos	https://www.targetvalidation.org/target/ENSG00000004455/associations		Mitochondrion intermembrane space		Yes		✓
													https://www.bindingdb.org/uniprot/P54819	

AK3	HUMAN GTP:AMP phosphotransferase AK3, mitochondrial	AK3L1, AK6, AKL3L1, KAD3	Q9UIJ7	ENSG0000(	Kinase	AK001951	Platelet count, Atopic eczema, Pancreatic ductal adenocarcinoma, Melanoma, Hepatocellular carcinoma, Hemophilia A, Hemophilia B, Thrombophilia, Severe hereditary factor VIII deficiency disease, Osteosarcoma	https://www.targetvalidation.org/target/ENSG00000147853/associations		Mitochondrion matrix,	No		✓
AK4	HUMAN Adenylate kinase 4, mitochondrial	AK3, AK3L1	P27144	ENSG0000(	Kinase	AL356212, AC099680	Venous thromboembolism, Lung carcinoma, Colorectal adenocarcinoma, Pancreatic carcinoma, Glioma, Degenerative polyarthritis, Deficiency of butyryl-CoA dehydrogenase	https://www.targetvalidation.org/target/ENSG00000162433/associations		Mitochondrion matrix,	Yes		✓
												https://www.bindingdb.org/uniprot/P27144	
AK7	HUMAN Adenylate kinase 7	KAD7	Q96M32	ENSG0000(	Kinase	NA	Ciliopathy, Azoospermia, Breast carcinoma, Ependymoma, Primary cliliary dyskinesia, Kartagener Syndrome, Sepsis, Young syndrome, Huntington disease	https://www.targetvalidation.org/target/ENSG00000140057/associations		Cytoplasm, cytosol	No		✓
AK9	HUMAN Adenylate kinase 9	AKD1, AKD2, C6orf199, C6orf224, KAD9	Q5TCS8	ENSG0000(	Kinase,Cancer	AK131244	Breast carcinoma, Azoospermia, Melanoma, Chronic Hepatitis C infection, Neopharyngeal neoplasm, Squamous cell carcinoma, Acute lymphoblastic leukemia, Psoriasis	https://www.targetvalidation.org/target/ENSG00000155085/associations		Cytoplasm	No		✓
AKAP3	HUMAN A-kinase anchor protein 3	AKAP110, CT82, FSP95, SOB1	O75969	ENSG0000(	Kinase	AK292451	Azoospermia, Li-Fraumeni syndrome, Hepatocellular carcinoma, Breast carcinoma, Lung carcinoma,<br>Ovarian carcinoma, Colonic neoplasm, Primary ciliary dyskinesia	https://www.targetvalidation.org/target/ENSG00000111254/associations	PRKAR2A<br>SPA17	Cytoplasmic vesicle, secretory vesicle, acrosome	No		✓
AKAP4	HUMAN A-kinase anchor protein 4	AKAP82, CT99, Fsc1, hAKAP82, HI, p82	Q5JQC9	ENSG0000(	Kinase	BC126250	Azoospermia, Lung carcinoma, Adenocarcinoma, Teratozoospermia, Male infertility due to globozoospermia, Asthenozoospermia, Colon carcinoma, Multiple myeloma, Pituitary neoplasm, Lobular carcinoma	https://www.targetvalidation.org/target/ENSG00000147081/associations		Cell projection, cilium, flagellum	No		✓
AKT1	HUMAN RAC-alpha serine/threonine-protein kinase	AKT, PKB, PRKBA, RAC	P31749	ENSG0000(	Kinase,Cancer	AK314256	Colonic neoplasm, Hereditay breast cancer, Proteus syndrome, Overgrowth syndrome, Cowden syndrome, Adenocarcinoma, Ovarian carcinoma, Colorectal adenocarcinoma, Melanoma, Squamous cell carcinoma	https://www.targetvalidation.org/target/ENSG00000142208/associations	IKBKG<br>TRAF6<br>PIKFYVE<br>MAPK8IP1<br>GAB2<br>APPL1<br>TBK1<br>DAXX<br>METTL1<br>MAPKAP1<br>MAP3K5<br>TRIB3<br>CAMKK2<br>PAWR<br>CLIP3<br>MUL1<br>RHEBL1<br>PIK3C3<br>CAMKK1<br>RASGRP3<br>SH3RF1<br>PHLPP2<br>RICTOR<br>PDPK2P<br>THEM4<br>LRRK2<br>PLEKHO1<br>CRTC2<br>CCDC88A	Cytoplasm, Nucleus, Cell membrane,	Yes		✓
												https://www.bindingdb.org/uniprot/P31749	
AKT3	HUMAN RAC-gamma serine/threonine-protein kinase	PKBG, PRKBG, RAC-gamma	Q9Y243	ENSG0000(	Kinase,Cancer	AJ245709	Hemimegalencephaly, Megalencephaly-polymicrogyria-postaxial-polydactyly-hydrocephalus, Breast carcinoma, Hepatocellular carcinoma, Juvenile absence epilepsy, Seizures, Macrocephaly, Hepatocellular carcinoma, Progressive myoclonic epilepsy, Ovarian carcinoma	https://www.targetvalidation.org/target/ENSG00000117020/associations	TRAF6<br>ALK<br>AKT1S1<br>PHLPP2<br>PLEKHO1<br>HSPB2<br>PDK1<br>PRKCD<br>PRKCZ<br>PPP2CA<br>PPP2CB<br>PTEN<br>TCL1A<br>MTCP1<br>CASP3<br>TGFBR2<br>HSP90AB1<br>HSP90AA1<br>FAM107A<br>PHLPP1<br>PDPK1<br>HSPB6<br>NOS3<br>STK4<br>PRKCZ<br>CASP9<br>PRKCD<br>PRKCG<br>CASP3	Nucleus	Yes		✓
												https://www.bindingdb.org/uniprot/Q9Y243	
ALDOA	HUMAN Fructose-bisphosphate aldolase A	ALDA	P04075	ENSG0000(	Cancer,Cancer-Antigen	AK301993	Glycogen storage disease, Breast carcinoma, Melanoma, Ovarian carcinoma, Pancreatic carcinoma, Lung carcinoma, Glycogen storage disease due to aldolase A deficiency, Myocardial Ischemia, Normochromic anemia, Congenital Epicanthus, Osteosarcoma	https://www.targetvalidation.org/target/ENSG00000149925/associations	CASP1<br>ALDOC<br>ISG15<br>ALDOB<br>TUBB<br>TGM2<br>SNX9<br>ALDOB<br>ALDOC<br>GPD2	Cytoplasm, myofibril, sarcomere, I band, Cytoplasm, myofibril, sarcomere, M line,	Yes		✓
												https://www.drugbank.ca/biodb/polypeptides/P04075	

ALOX15	HUMAN Arachidonate 15-lipoxygenase	15-LOX-1, LOX15	P16050	ENSG0000(	Others	AK316126	Chronic myeloproliferative disorder, Essential thrombocythemia, Autoimmune lymphoproliferative syndrome, Hereditary thrombocytopenia with normal platelets, Prostate carcinoma, RAS-associated autoimmune leukoproliferative diseaese, Atherosclerosis, Glomerulonephritis, Asthma, Alzheimer's disease	https://www.targetvalidation.org/target/ENSG00000161905/associations		Cytoplasm, cytosol, Cell membrane, Peripheral membrane protein, Lipid droplet,	Yes		✓
												https://www.bindingdb.org/uniprot/P16050	
ALOX15B	HUMAN Arachidonate 15-lipoxygenase B	15-LOX-2, LX15B	O15296	ENSG0000(	Others	AF468052	Prostate carcinoma, Tongue squamous cell carcinoma, Lung carcinoma, Asthma, Esophageal squamous cell carcinoma, Narcolepsy, Atherosclerosis, Melanoma, Adrenal cortical adenoma, Epithelial ovarian cancer	https://www.targetvalidation.org/target/ENSG00000179593/associations		Nucleus	Yes		✓
												https://www.bindingdb.org/uniprot/O15296	
ALPK1	HUMAN Alpha-protein kinase 1	FLJ22670, KIAA1527, Lak	Q96QP1	ENSG0000(	Kinase	AC109347	Breast carcinoma, Epithelial neoplasm, Colorectal cancer, Obesity, Colorectal cancer, Adenocarcinoma, Maple syrup urine disease, Diabetes mellitus, Bile duct carcinoma, Chronic myeloid leukemia	https://www.targetvalidation.org/target/ENSG00000073331/associations			No		✓
ALX1	HUMAN ALX homeobox protein 1	CART1	Q15699	ENSG0000(	Transcription factor	NA	Frontonasal dysplasia-severe micropthalmia-severe facial clefting syndrome, Ovarian carcinoma, Delayed membranous cranial ossification, Isolated craniosynostosis, Lung carcinoma, Spina fibida, Microphthalmos, Uranostaphyloschisis, Prominent glabella, Meckel syndrome	https://www.targetvalidation.org/target/ENSG00000180318/associations	CREBBP ALX4	Nucleus	No		✓
AMD1	HUMAN S-adenosylmethionine decarboxylase proenzyme	SAMDC, DCAM	P17707	ENSG0000(	Signalling	AL365206, AL357515	Epithelial neoplasm, Astrocytoma, Glioblastoma multiforme, Prostate carcinoma, Lung carcinoma, Bipolar disorder, Breast carcinoma, Spina bifida, Colorectal cancer	https://www.targetvalidation.org/target/ENSG00000123505/associations			Yes		✓
												https://www.bindingdb.org/uniprot/P17707	
AMOTL2	HUMAN Angiomotin-like protein 2	LCCP, AMOL2	Q9Y2J4	ENSG0000(	Cancer,Cancer-Antigen	AK223598	Breast carcinoma, Glioma, Gliobalstoma multiforme, Melanoma, Wrinkly skin syndrome, Breast carcinoma, Ovarian carcinoma, Colon carcinoma, Atopy, Melanoma	https://www.targetvalidation.org/target/ENSG00000114019/associations	LATS2 MTOR FGFR1	Recycling endosome	No		✓
AMT	HUMAN Aminomethyltransferase, mitochondrial	GCST, NKH	P48728	ENSG0000(	Cancer,Cancer-Antigen	AK293481	Glycine encephalopathy, Glioma, Neural tube closure defect, Spina bifida-hypospadias, Unipolar depression, Epilepsy, Glycinuria, Hyperreflexia, Hyperglycinemia, Agenesis of corpus callosum	https://www.targetvalidation.org/target/ENSG00000145020/associations		Mitochondrion	Yes		✓
												https://www.drugbank.ca/biodb/polypeptides/P48728	
ANKRD13A	HUMAN Ankyrin repeat domain-containing protein 13A	ANKRD13, NY-REN-25, AN13A	Q8IZ07	ENSG0000(	Others	NA	Familial thrombocytosis, Autosomal recessive spondylocostal dysostosis, Essential thrombocythemia, Multiple synostoses syndrome, Polychemia, Jeune syndrome, Dubin-Johnson syndrome, Polycythemia cera, Thyroid ectopia, Zellweger syndrome	https://www.targetvalidation.org/target/ENSG00000076513/associations	TOLLIP	Cell membrane	No		✓
ANKRD45	HUMAN Ankyrin repeat domain-containing protein 45	ANR45	Q5TZF3	ENSG0000(	Others	CH471067	Ependymoma, Azoospermia, Melanoma, Central nervous system cancer, Li-Fraumeni syndrome, Ovarian carcinoma, Neuroblastoma, Colorectal adenocarcinoma, Squamous cell carcinoma	https://www.targetvalidation.org/target/ENSG00000183831/associations			No		✓

ANXA1	HUMAN Annexin A1	ANX1, LPC1	P04083	ENSG0000(	Signalling, Cancer	NA	Digestive system disease, Epithelial neoplasm, Ovarian carcinoma, Lung carcinoma, Central nervous system cancer, Ulcerative colitis, Breast carcinoma, Craniofacial Abnormalities, Inflammatory bowel disease	https://www.targetvalidation.org/target/ENSG00000135046/associations	IKBKG TRPM7 RIPK1 PRKCE PRKX PRKCI PRKCH PRKACA PRKCA SRC PRKCB PRKCG EGFR ABL1 PRKY TGM2 SRC KRT8 RELA IKBKG RIPK1 PLA2G4A FPR2 FPR3 FPR1 S100A11	Nucleus	Yes	https://www.drugbank.ca/biodb/polypeptides/P04083	✓
ANXA11	HUMAN Annexin A11	ANX11	P50995	ENSG0000(	Cancer,Cancer-Antigen	AJ278465	Sarcoidosis, Psoriasis, Ovarian carcinoma, Surfactant protein D measurement, Pancreatic carcinoma, Chronic obstructive pulmonary disease, Femur head necrosis, Celiac disease, Type I diabetes mellitus, Juvenile arthritis	https://www.targetvalidation.org/target/ENSG00000122359/associations	S100A6	Cytoplasm, Melanosome, Nucleus envelope, Nucleus, nucleoplasm, cytoskeleton, spindle,	No		✓
ANXA2	HUMAN Annexin A2	ANX2, ANX2L4, CAL1H, LIP2, LPC2D	P07355	ENSG0000(	Cancer	AC087385	Central nervous system cancer, Glioma, Astrocytoma, Hepatocellular carcinoma, Pancreatic carcinoma, Ependymoma, Osteoporosis, Squamous cell carcinoma, Acute myelocytic leukemia, Duchenne muscular dystrophy	https://www.targetvalidation.org/target/ENSG00000182718/associations	MAP3K4 SCYL2 PRKCZ PRKCE BMX PRKCA SRC CSF1R PRKCB ISG15 PRKCG APOH S100A10 STAT6 S100A4 CEACAM1 PHB PLG PLAT PCSK9	Secreted, extracellular space, extracellular matrix, basement membrane	Yes	https://www.drugbank.ca/biodb/polypeptides/P07355	✓
AP2M1	HUMAN AP-2 complex subunit mu	AP50, CLAPM1, mu2	Q96CW1	ENSG0000(	Cancer,Cancer-Antigen	AC131235	HIV infection, Ovarian carcinoma, Pancreatic carcinoma, Posterior cortical atrophy, Malignant peripheral nerve sheath tumor, Hepatitis C infection, Squamous cell carcinoma, Lung adenocarcinoma, Multiple myeloma, Acute quadriplegic myopathy	https://www.targetvalidation.org/target/ENSG00000161203/associations	RNF111 AAK1 RALBP1 KCNJ11 AP2S1 ADRA1B CD22 IFNAR1 CTLA4 FURIN CD3D AP2A1 AP2A2 TGOLN2 GAK EGFR ARRB2 DVL2 AP2B1 AP2A1	Cell membrane, Membrane, coated pit, Peripheral membrane protein, Cytoplasmic side,	No		✓
APCS	HUMAN Serum amyloid P-component	PTX2	P02743	ENSG0000(	Others	CR450313	Heart disease, AL amyloidosis, Amyloidosis, Common variable mmunodeficiency, Alport syndrome, Primary membranoproliferative glomerulonephritis, Immunodeficiency due to a complement cascade protein anomaly, Liver carcinoma, Atherosclerosis, Amyloid plaque	https://www.targetvalidation.org/target/ENSG00000132703/associations	CRP COL4A1 TG CALU LAMA1 C4BPA C1QA	Secreted	No		✓
APEX1	HUMAN DNA-(apurinic or apyrimidinic site) lyase	APE, APE-1, APEN, APEX, APX, HAP1, REF-1, REF1	P27695	ENSG0000(	Signalling, Cancer	S43127	Hepatocellular carcinoma, Sarcoma, Osteosarcoma, Gastric carcinoma, Systemic lupus erythematosus, Lung carcinoma, Familial Mediterranean fever, Melanoma, Liver carcinoma, Li-Fraumeni syndrome	https://www.targetvalidation.org/target/ENSG00000100823/associations	SIRT1 DKFZp686L02273 TRAF2 EP300 ERCC6 MDM2 CSNK2A1 STAT3 FEN1 GADD45A CSNK2A2 XRCC1 HNRNPL XRCC6 GZMA PCNA HSPA1A POLB GAPDH RECQL4 ARNT TP53 CCNO FOSL1 TRAF2 MUTYH TXN NME1 STAT3	Nucleus, nucleolus, Nucleus speckle, Endoplasmic reticulum, Cytoplasm,	Yes	https://www.bindingdb.org/uniprot/P27695	✓
API5	HUMAN Apoptosis inhibitor 5	AAC11, FIF, MIG8, XAGL	Q9BZZ5	ENSG0000(	Others	AF229254	Age-related nuclear cataract, Migraine disorder, Hepatocellular carcinoma, Parental longevity, Cervical carcinoma, Adenocarcinoma, Liver carcinoma, Lupus nephritis, Cervix carcinoma, Systemic lupus erythematosus	https://www.targetvalidation.org/target/ENSG00000166181/associations	FGF2 ACIN1	Nucleus	No		✓
APOBEC3G	HUMAN DNA dC->dU-editing enzyme APOBEC-3G	CEM15, MDS019, ABC3G	Q9HC16	ENSG0000(	Others	CR456472	HIV infection, Colonic neoplasm, Li-Fraumeni syndrome, Pancreatic carcinoma, Lymphoma, Immunologic deficiency syndrome, Retroviridae infection, Hepatitis C infection, Colorectal adenocarcinoma	https://www.targetvalidation.org/target/ENSG00000239713/associations	APOBEC3F PRKACA HDAC6	Cytoplasm, Nucleus, Cytoplasm, P-body,	Yes	https://www.bindingdb.org/uniprot/Q9HC16	✓

APP	HUMAN Amyloid beta A4 protein	AD1, A4	P05067	ENSG0000(	Others	M33112	Neurodegenerative disease, Tauopathy, Alzheimer's disease, Cardiovascular disease, Genetic disorder, AL amyloidosis, Amyloidosis, Dementia, Hereditary cerebral hemorrhage with amyloidosis, Vascular disease	https://www.targetvalidation.org/target/ENSG00000142192/associations	BACE2<br>CCL26<br>MAPK8IP1<br>PSENEN<br>TAB2<br>IFIH1<br>MAP3K5<br>DHX58<br>APH1A<br>TSLP<br>KAT5<br>NCSTN<br>APH1B<br>RASGRP3<br>PLA2G4D<br>CTF1<br>SNCB<br>NFKBIB<br>CASP8<br>MAPK7<br>CACNB1<br>CDK5<br>GRB2<br>FGF12<br>RAP1B<br>BACE1<br>CASP6<br>POLR2K<br>MAPK10	Membrane, Single-pass type I membrane protein, Membrane, clathrin-coated pit,	Transmembrane	Yes	https://www.bindingdb.org/uniprot/P05067	✓
APPL1	HUMAN DCC-interacting protein 13-alpha	APPL, DP13A, KIAA1428	Q9UKG1	ENSG0000(	Signalling	BC028599	Diabetes mellitus, MODY, Hepatocellular carcinoma, Osteosarcoma, Breast carcinoma, Obesity, Alzheimer's disease, Prostate carcinoma, Crohn's disease, Hereditary cerebral cavernous malformation	https://www.targetvalidation.org/target/ENSG00000157500/associations	AKT3<br>ADIPOR1<br>ADIPOR2<br>OCRL<br>AKT2<br>AKT1<br>PRKACA<br>PRKCA AR<br>EGFR<br>AKT1<br>PIK3CA<br>AKT2 AR<br>STK11<br>MTA2<br>RAB5A<br>DCC<br>RBBP7	Early endosome membrane		No		✓
AR	HUMAN Androgen receptor	AIS, DHTR, NR3C4, SMAX1, ANDR	P10275	ENSG0000(	Transcription factor,Cancer	M34233	Prostate carcinoma, Heart failure, Panhypopituitarism, Sexual dysfunction, Erectile dysfunction, Osteoporosis, Cryptorchidism, X-linked Bulbo-Spinal atrophy, Reifenstein syndrome, Androgen-Insensitivity syndrome	https://www.targetvalidation.org/target/ENSG00000169083/associations	PIAS3<br>NCOA3<br>NCOR2<br>KDM3A<br>RAD54L2<br>GRIP1<br>PA2G4<br>STUB1<br>ZMIZ1<br>VAV3<br>APPL1<br>DAXX<br>RNF14<br>PSMC3IP<br>PAK6<br>PATZ1<br>HIPK3<br>TBL1XR1<br>WDR77<br>PRMT1<br>RANBP9<br>RCHY1<br>NSD1<br>SIRT1<br>KAT5<br>KAT2B<br>CREBBP<br>APPBP2<br>GADD45GI<br>PBK IRF7	Nucleus		Yes	https://www.bindingdb.org/uniprot/P10275	✓
ARAF	HUMAN Serine/threonine-protein kinase A-Raf	ARAF1	P10398	ENSG0000(	Kinase	AB158254	Eye disease, Multiple endocrine neoplasia type 2, Bilateral striopallidodentate calcinosis, Hirschsprung disease, Adenocarcinoma, Melanoma, Langerhans-Cell histiocytosis, Atrial fibrillation, Glioma, Intrahepatic cholangiocarcinoma	https://www.targetvalidation.org/target/ENSG00000078061/associations	PAK1<br>MAP2K1<br>RRAS2<br>TIRAP<br>CDK7<br>MAP2K2<br>SRC<br>RRAS<br>PDGFRB<br>KRAS<br>HRAS<br>NRAS<br>YWHAG<br>SLC9A3R2<br>MCM7<br>MAP2K2<br>RRAS<br>MAPK1<br>MAP2K1<br>BRAF<br>HRAS<br>PKM<br>PIK3R1<br>BAD<br>CSNK2B<br>MAPK3<br>SRSF1<br>RRAS2			Yes	https://www.bindingdb.org/uniprot/P10398	✓
AREG	HUMAN Amphiregulin	AREGB, SDGF	P15514	ENSG0000(	Kinase,Cancer, Cytokine/Chemokine	BT019866	Colonic neoplasm, Colorectal adenocarcinoma, Breast carcinoma, Mammographic density measurement, Li-Fraumeni syndrome, Asthma, Squamous cell carcinoma, Juvenile arthritis, Pulmonary fibrosis, Ovarian neoplasm	https://www.targetvalidation.org/target/ENSG00000109321/associations	CCND3<br>SRC<br>EGFR<br>EGFR<br>MMP9<br>ERBB2<br>WT1<br>WDR92	Membrane, Single-pass membrane protein,	Transmembrane	Yes	https://www.bindingdb.org/uniprot/P15514	✓
ARHGDIA	HUMAN Rho GDP-dissociation inhibitor 1	GDIA1, RHOGDI, GDIR1	P52565	ENSG0000(	Others	X63863	Nephrotic syndrome, Glioma, Melanoma, Glioblastoma multiforme, Familial idiopathic steroid resistant nephrotic syndrome, Hypoalbuminemia, Large cell lung carcinoma, Astrocytoma, Squamous cell lung carcinoma, Anaplastic astrocytoma	https://www.targetvalidation.org/target/ENSG00000141522/associations	PAK1<br>RAC1<br>CASP3<br>RDX<br>CASP1<br>MSN<br>PRKACA<br>PRKCA<br>IL7R EZR<br>JUP SRC<br>RHOA<br>RAC2<br>CDC42<br>RAC1<br>XIAP<br>RHOB JUP<br>RNF128	Cytoplasm		Yes	https://www.bindingdb.org/uniprot/P52565	✓
ARHGDIB	HUMAN Rho GDP-dissociation inhibitor 2	GDIA2, GDID4, Ly-GDI, RAP1GN1	P52566	ENSG0000(	Others	AF498927	Ovarian carcinoma, Breast carcinoma, Malignant mesothelioma, Lung carcinoma, Li-Fraumeni syndrome	https://www.targetvalidation.org/target/ENSG00000111348/associations	SYK<br>CASP3<br>PRKCA<br>EZR RAC2<br>SRC<br>RHOA<br>CDC42<br>RAC1<br>VAV1	Cytoplasm, cytosol		No		✓
ARHGDIG	HUMAN Rho GDP-dissociation inhibitor 3	RHOGDI-3	Q99819	ENSG0000(	Others	AF498928	Central nervous system cancer, Ependymoma, Astrocytoma, Glioblastoma multiforme, Medulloblastoma, Breast carcinoma, Esophageal squamous cell carcinoma, Oligodendroglioma, Prostate carcinoma	https://www.targetvalidation.org/target/ENSG00000242173/associations	RAC1<br>RHOA<br>CDC42<br>RHOB	Cytoplasm,		No		✓
ARIH2	HUMAN E3 ubiquitin-protein ligase ARIH2	TRIAD1, ARI2	O95376	ENSG0000(	Cancer,Cancer-Antigen	BC000422	Self-reported educational attainment cancer, Atopic eczema, Cryptosporidiosis-chronic cholangitis-liver disease, Chronic granulomatous disease, Isolated agammaglobulinemia, Thymic aplasia, Hepatocellular adenoma, Porphyria cutanea tarda	https://www.targetvalidation.org/target/ENSG00000177479/associations	BUB1<br>TRIM24<br>GFI1<br>MDM2<br>UBE2L3	Nucleus		No		✓

ARL1	HUMAN ADP-ribosylation factor-like protein 1	ARFL1	P40616	ENSG0000(	Cancer,Cancer-Antigen	AK301701	Melanoma, Medulloblastoma, Prostate carcinoma, Crohn's disease, Dermatomyositis, Ovarian carcinoma, Breast carcinoma, Small cell lung carcinoma, Alzheimer's disease, Acute myeloid leukemia	https://www.targetvalidation.org/target/ENSG00000120805/associations	SYT1 MOGS MON2	Golgi apparatus membrane	Yes		✓
												https://www.drugbank.ca/biodb/polypeptides/P40616	
ARMC3	HUMAN Armadillo repeat-containing protein 3	CT81, FLJ32827	Q5W041	ENSG0000(	Others	AL139815, AC079269	Central nervous system cancer, Ependymoma, Azoospermia, Serum IgG glycosylation measurement, Nasopharyngeal neoplasm, Endometrial neoplasm, Pancreatic carcinoma, Lung carcinoma, Prostate carcinoma, Breast carcinoma	https://www.targetvalidation.org/target/ENSG00000165309/associations			No		✓
ARNT	HUMAN Aryl hydrocarbon receptor nuclear translocator	bHLHe2, HIF-1beta	P27540	ENSG0000(	Transcription factor,Cancer	AK293027	Kidney neoplasm, Cutaneous melanoma, Lung carcinoma, Esophageal carcinoma, Prostate carcinoma, Melanoma, Endometriosis, Precursor cell lymphoblastic leukemia lymphoma, Liver Cirrhosis, Anoxia	https://www.targetvalidation.org/target/ENSG00000143437/associations	HEY2 HIF1AN SENP6 EPAS1 CREBBP HIF1A EP300 CSNK2A1 UBE2I STAT1 AHR APEX1 TXN ESR1 HIF1A BRCA1 EP300 EGLN3 EGLN1 NCOA1 PTGES3 AHR TRIP11 SIM2	Nucleus	No		✓
ARRB1	HUMAN Beta-arrestin-1	ARR1	P49407	ENSG0000(	Signalling	AF084940	Prostate carcinoma, Li-Fraumeni syndrome, Familial short QT syndrome, King-Denborough syndrome, Fanconi anemia, Liver cirrhosis, Arteriovenous fistula, Cernunnos-XLF deficiency, Dyskeratosis congenita, MYH9-related disease	https://www.targetvalidation.org/target/ENSG00000137486/associations	TRAF6 FZD1 SMO MAPK14 MDM2 CLTC AP2B1 GNB1 AP2S1 CCR5 GNAQ NSF CSK TRHR GRK5 MAPK1 MAPK3 NFKBIA GRK2 CXCR2 SRC FGR CLTA HCK ADRB1 IGF1R ADRB2 JUN UBE4B DVL2 DVL1 ITCH CLTC AGTR1	Cytoplasm, Nucleus, Cell membrane, Membrane, clathrin-coated pit Cytoplasmic vesicle,	Yes		✓
												https://www.bindingdb.org/uniprot/P49407	
ARX	HUMAN Homeobox protein ARX	CT121, EIEE1, ISSX, MRX29, MRX32, MRX33, MRX36, MRX38, MRX43, MRX54, MRX76, MRX87, MRXS1, PRTS	Q96QS3	ENSG0000(	Transcription factor	NA	West syndrome, Partington syndrome, Infantile epileptic-dyskinetic encephalopathy, Lissencephaly, Spasticity-intellectual disability-X-linked epilepsy, Epilepsy syndrome, CLN1 disease, Ovarian carcinoma, Frontotemporal dementia, Adult neuronal ceroid lipofuscinosis	https://www.targetvalidation.org/target/ENSG00000004848/associations		Nucleus	No		✓
ASB1	HUMAN Ankyrin repeat and SOCS box protein 1	ASB-1	Q9Y576	ENSG0000(	Others	AB032972	Circadian rhythm, Osteosarcoma, Breast carcinoma, Forced expiratory volume, Pulmonary function measurement, Psoriasis, Atopic eczema, Melanoma, Pulmonary mucoepidermoid carcinoma, Ankylosing spondylitis	https://www.targetvalidation.org/target/ENSG00000065802/associations			No		✓
ASCL1	HUMAN Achaete-scute homolog 1	ASH1, bHLHa46, HASH1	P50553	ENSG0000(	Transcription factor	BC001638	Adenocarcinoma, Uterine fibroid, Glioma, Breast carcinoma, Juvenile amyotrophic lateral sclerosis, Strabismus, Epilepsy, Autonomic dysregulation, Congenital central hypoventilation, Astrocytoma	https://www.targetvalidation.org/target/ENSG00000139352/associations	DTX1 BMP2 CDK1 MEF2A	Nucleus	No		✓
ASNA1	HUMAN ATPase ASNA1	ARSA, TRC40, ASNA	O43681	ENSG0000(	Cancer,Cancer-Antigen	AY304483	Ovarian carcinoma, Melanoma, Adenoma, Breast carcinoma, Emery-Dreifuss muscular dystrophy, Parkinson's disease, Rheumatoid arthritis, Tibial muscular dystrophy, Congenital heart disease, Colorectal adenocarcinoma	https://www.targetvalidation.org/target/ENSG00000198356/associations	SEC61B	Cytoplasm, Endoplasmic reticulum, Nucleus, nucleolus,	Yes		✓
												https://www.drugbank.ca/biodb/polypeptides/O43681	

ASPSCR1	HUMAN Tether containing UBX domain for GLUT4	ASPL, RCC17, TUG, UBXD9, UBXN9, ASPC1	Q9BZE9	ENSG0000(	Cancer	AK290624	Adenocarcinoma, Melanoma, Renal carcinoma, Acute lymphoblastic leukemia, Oral squamous cell carcinoma, Bladder carcinoma, Colorectal adenocarcinoma, Sarcoma, Kidney neoplasm, Type II diabetes mellitus	https://www.targetvalidation.org/target/ENSG00000169696/associations	VCPKMT	Endomembrane system	No		✓
ATF1	HUMAN Cyclic AMP-dependent transcription factor ATF-1	TREB36	P18846	ENSG0000(	Transcription factor,Cancer	BC029619	Sarcoma, Melanoma, Breast carcinoma, Ewings sarcoma, Histiocytoma, Angiomatoid fibrous histiocytoma, Polycystic ovary syndrome, Colorectal adenocarcinoma, Retinal dystrophy, Hypertension	https://www.targetvalidation.org/target/ENSG00000123268/associations	HIPK2 CAMK1 CAMK2G SMAD4 PPP2R5C PRKCE CDK3 CSNK2A1 CSNK1A1 HNF1B CSNK2A2 PRKACA RPS6KA4 RPS6KA5 BRCA1 GOLPH3 SH3RF1 JUN NKX6-1 ATM CCDC65 IL10 ARNT2	Nucleus	Yes	https://www.bindingdb.org/uniprot/P18846	✓
ATF2	HUMAN Cyclic AMP-dependent transcription factor ATF-2	CRE-BP1, CREB2, HB16, TREB7	P15336	ENSG0000(	Transcription factor	DQ003037	Congenital Pulmonary alveolar Proteinosis, Congenital lobar Emphysema, Wiskott-Aldrich syndrome, Neonatal acute respiratory distress with surfactant metabolism deficiency, Lethal congenital contracture synfrome type 3, Familial primary pulmonary hypoplasia, Endotoxemia, Glaucoma, Lung neoplasm, Melanoma	https://www.targetvalidation.org/target/ENSG00000115966/associations	PLK3 MAPKAP1 VRK1 CREBBP JDP2 MAPKAPK5 IRF2BP1 MAPK14 MAPK11 SMAD4 ATM PRKCE SMAD3 CSNK2A1 MAPK10 MAPK12 BLVRA PRKX MAPK9 MAPK8 KRT2 MAPK1 MAPK3 CDK2 CSNK2A2 PRKACA PRKCA CREB1 RB1	Nucleus, Cytoplasm, Mitochondrion outer membrane,	Yes	https://www.drugbank.ca/biodb/polypeptides/P15336	✓
ATF3	HUMAN Cyclic AMP-dependent transcription factor ATF-3	ATF3	P18847	ENSG0000(	Transcription factor,Signalling	AB078027	Epithelial neoplasm, Melanoma, Lung carcinoma, Glioma, Wiskott-Aldrich syndrome, Hypospadia, Type II diabetes mellitus, Astrocytoma, Hermansky-Pudlak syndrome type 9, Cystinosis	https://www.targetvalidation.org/target/ENSG00000162772/associations	SMAD3 FGFR3 JUND ATF2 JUN TP53 ATF4 TP53 STAT1 NFKB1 JUN CGA MDM2 HDAC1 CREBBP PDX1	Nucleus	Yes	https://www.drugbank.ca/biodb/polypeptides/P18847	✓
ATF4	HUMAN Cyclic AMP-dependent transcription factor ATF-4	CREB-2, TAXREB67, TXREB	P18848	ENSG0000(	Transcription factor,Signalling	AL022312	Metabolic disease, Isolated anophthalmia-microphthalmia, Bone disease, Kenny-Caffey syndrome, Fibrochondrogenesis, Schizophrenia, Hypoxia, Necrosis, Bipolar disorder, Alzheimer's disease	https://www.targetvalidation.org/target/ENSG00000128272/associations	BTRC EIF2AK3 CREBZF EIF2A CCDC106 TRIB3 RPS6KA2 CEBPG RPS6KA3 DDIT3 ATF3 CEBPB RET DAPK3 NFE2L2 EP300 FUS ULK1 MAP1LC3B DDIT3 TRIB3 ATG5 CREBBP GPS2 LDOC1 FBXW11 NFE2L1 ABRAXAS2	Cytoplasm	Yes	https://www.drugbank.ca/biodb/polypeptides/P18848	✓
ATF5	HUMAN Cyclic AMP-dependent transcription factor ATF-5	ATF5	Q9Y2D1	ENSG0000(	Transcription factor	AK024402	Colorectal adenocarcinoma, Glioma, Cytomegalavirus infection, Hepatocellular carcinoma, Acute lymphoblastic leukemia, Glioblastoma, Hemophagocytic lymphohistiocytosis, Bipolar disorder, Schizophrenia, Asthma	https://www.targetvalidation.org/target/ENSG00000169136/associations	TRIB3 EP300 CDC34 CCND3	Cytoplasm	Yes	https://www.drugbank.ca/biodb/polypeptides/Q9Y2D1	✓
ATG12	HUMAN Ubiquitin-like protein ATG12	APG12, APG12L	O94817	ENSG0000(	Others	BC012266	Hepatocellular carcinoma, Lung disease, Melanoma, Leucocyte adhesion deficiency, Gastric carcinoma, Spinocerebellar ataxia type 7, HIV infection, Colorectal cancer, Multiple myeloma, Juvenile dermatomyositis	https://www.targetvalidation.org/target/ENSG00000145782/associations	ATG3 ATG5 ATG10 IFIH1 WDFY3 TECPR1 MAVS PTK2 DDX58 ATG7 DDX58 IFIH1 ATG16L1 MAVS ATG7 ATG10 ATG5 ATG16L2 WIPI1	Cytoplasm	No		✓
ATG7	HUMAN Ubiquitin-like modifier-activating enzyme ATG7	APG7L, GSA7	O95352	ENSG0000(	Cancer,Cancer-Antigen	AC022001	High density lipoprotein cholesterol measurement, Leukemia, Glioma, Asthma, Chronic myelogenous leukemia, Lethargy, Necrosis, Paraplegia, Myocardial infarction, Anemia	https://www.targetvalidation.org/target/ENSG00000197548/associations	TP63 GABARAPL1 FOXO1 HSF1 GABARAPL2 GABARAP ATG12 TP73 GABARAP EP300 MAP1LC3A MAP1LC3B ATG3 ATG12 ATG10 ATG5	Cytoplasm	Yes	https://www.bindingdb.org/uniprot/O95352	✓
ATIC	HUMAN Bifunctional purine biosynthesis protein PURH	AICARFT, IMPCHASE, PURH, PUR9	P31939	ENSG0000(	Cancer	AB062403	AICA-ribosiduria, Inborn errors of metabolism, Lymphoid neoplasm, Kidney neoplasm, Melanoma, Rheumatoid arthritis, Brachycephaly, Congenital blindness, Lung carcinoma, Colorectal adenocarcinoma	https://www.targetvalidation.org/target/ENSG00000138363/associations	ALK ISG15		Yes	https://www.bindingdb.org/uniprot/P31939	✓

ATP5B	HUMAN ATP synthase subunit beta, mitochondrial	ATPSB, ATPMB	P06576	ENSG0000(	Cancer	D00022	Tetatozoospermia, Ovarian carcinoma, Glioblastoma multiforme, Breast carcinoma, Polyp, Hypoxia, Renal fibrosis, Diabetic nephropathy, Spinocerebellar ataxia 17, Prostate carcinoma	https://www.targetvalidation.org/target/ENSG00000110955/associations	ATPAF2 ATPAF1 ATP5O ATP5C1 ATP5D ATP5A1 ATP5F1 IL7R ISG15 APOA1 ATP5A1 ATP5C1 ATP5D ATP5O ATP5F1 FIS1	Mitochondrion, Mitochondrion inner membrane,	Yes		✓
												https://www.drugbank.ca/biodb/polypeptides/P06576	
ATXN3	HUMAN Ataxin-3	ATXN3, MJD, MJD1, SCA3	P54252	ENSG0000(	Transcription factor	U64822	Autosomal dominant cerebellar ataxia, Spinocerebellar ataxia type 3, Neurodegenerative disease, Blackfan-Diamond anemia, Congenital dyserthropoietic anemia, Truncal ataxia, Bradykinesia, Machado-Joseph disease, Distal amyotrophy, Autosomal dominant cerebellar ataxia type 1	https://www.targetvalidation.org/target/ENSG00000066427/associations	CSNK2A1 GSK3B UBC PRKN HAP1 FOXO4 SQSTM1 CSNK2B PNKP CREBBP	Nucleus matrix	No		✓
AURKA	HUMAN Aurora kinase A	AIK, ARK1, AurA, BTAK, PPP1R47, STK15, STK6, STK7	O14965	ENSG0000(	Kinase,Cancer	AF011468	Neoplasm of mature T-cells or NK-cells, Lymphoid neoplasm, Melanoma, Hepatocellular carcinoma, Lung carcinoma, Aneuploidy, Neuroblastoma, Multiple myeloma, Fanconi anemia, Dyskeratosis congenita	https://www.targetvalidation.org/target/ENSG00000087586/associations	TPX2 AURKAIP1 FBXW7 BORA NUMA1 PAK1 PPP1CB PPP1CA PLK1 PRKX GSK3B STAT5A BRCA1 PPP1CC PRKACA TP53 PRKY PPP6C HDAC6 FAF1 STMN1 EGFR PRKACA PPP1CB TACC1 KIF11 BRCA1 BORA TP53	Cytoplasm, cytoskeleton, microtubule organizing center, centrosome	Yes		✓
												https://www.bindingdb.org/uniprot/O14965	
AURKB	HUMAN Aurora kinase B	Aik2, AIM-1, ARK2, AurB, IPL1, PPP1R48, STK12, STK5	Q96GD4	ENSG0000(	Kinase,Cancer	AF004022	Lung carcinoma, Central nervous system cancer, Leukemia, Multiple myeloma, Sarcoma, IGA Glomerulonephritis, Glioma, Endometrial neoplasm, Melanoma, Hepatocellular carcinoma	https://www.targetvalidation.org/target/ENSG00000178999/associations	AURKC INCENP CDCA8 STK4 FOXM1 CENPA CDKN2A PARP1 KIF20A BIRC5 CHEK1 GNAZ STMN1 PHB2 ATP2A2 PRKACA DES BIRC5 RACGAP1 HMGN2 TACC1 SUPT16H HDAC4 DDA1 RPA1 GFAP TP53 KRT8 RIF1 HIST3H3	Nucleus, Chromosome, Chromosome, centromere, Cytoplasm, cytoskeleton, spindle, Midbody,	Yes		✓
												https://www.bindingdb.org/uniprot/Q96GD4	
AXIN1	HUMAN Axin-1	AXIN	O15169	ENSG0000(	Kinase,Cancer	Z69667, AC005202, Z99754	Hepatocellular adenonoma, Caudal duplication, Breast carcinoma, Hepatocellular carcinoma, Oral cavity cancer, Liver carcinoma, Double ureter, Uterus bilocularis, Prostate carcinoma, Oral cavity cancer	https://www.targetvalidation.org/target/ENSG00000103126/associations	MAP3K4 TNKS2 ANAPC1 DVL3 AMER1 PPP2R5B PPP2R5A PPP2R5C CDK5 DAB2 PPP2CA SUMO1 GNAS PPP2CB PPP1CB PPP1CA GSK3B GSK3A CSNK1E CSNK1D CSNK1A1 TGFBR1 PPP1CC PPM1A CTNNB1 APC MYC APC2 TNKS LRP6	Cytoplasm	No		✓
AXL_int	HUMAN Tyrosine-protein kinase receptor UFO	UFO	P30530			AC011510							✓
BACH1	HUMAN Transcription regulator protein BACH1	BACH-1, BTBD24	O14867	ENSG0000(	Transcription factor	AF124731	Pancreatic carcinoma, Azoospermia, MODY, Herediatary coproporphyria, Pseudohypoaldosteronism type 1, Down syndrome, Breast carcinoma, Alzheimer's disease, Fanconi anemia, Epithelial ovarian cancer	https://www.targetvalidation.org/target/ENSG00000156273/associations	MAFK MAFG NFE2L2 HMMR NQO1 XPO1	Nucleus	No		✓
BAD	HUMAN Bcl2-associated agonist of cell death	BBC2, BCL2L8	Q92934	ENSG0000(	Cancer	AK291863	Familial hyperinsulinism, Exercise-induced hyperinsulinism, Dioxide-resistant focal hyperinsulinism due to SUR1 deficiency, Autosomal dominant hyperinsulinism due to Kir6.2 deficiency, Diazoxide-resistant focal hyperinsulinism due to Kir6.2 deficiency, Type II diabetes mellitus, Cardiomegaly, Prion disease, Pancreatic adenocarcinoma, Hepatocellular carcinoma	https://www.targetvalidation.org/target/ENSG00000002330/associations	RIPK3 AKT3 RPS6KA6 PAK5 PIM2 SGK494 BCL2L2 PIM3 BAK1 RPS6KA1 RPS6KA2 PRKD1 PDK1 PPP2R5C ATM MAP2K5 PAK1 PPP3CA MCL1 BCL2L1 BAX YWHAH PRKCQ PRKCE PPP2CA YWHAZ PPP2CB YWHAE PPP1CB	Mitochondrion outer membrane, Cytoplasm	Yes		✓
												https://www.bindingdb.org/uniprot/Q92934	
BAG3	HUMAN BAG family molecular chaperone regulator 3	BAG3	O95817	ENSG0000(	Cancer,Cancer-Antigen	AK291333	Familial dilated cardiomyopathy, Myofibrillar myopathy, Muscular dystrophy, Selcen type, Cardiomyopathy, Dilated cardiomyopathy, Familial isolated dilated cardiomyopathy, Desminopathy, Myofibrillar myopathy, Peripheral demyelinating neuropathy	https://www.targetvalidation.org/target/ENSG00000151929/associations	HSPB8 PRKCD IRAK1 MTOR BCL2 HSPA4	Nucleus	No		✓

BAG4	HUMAN BAG family molecular chaperone regulator 4	SODD	O95429	ENSG0000(	Others	AC084024	Bone developmental disease, Acute lymphoblastic leukemia, Breast carcinoma, Tuberculosis, Cutaneous T-cell lymphoma, Non-Hodgkin's lymphoma, Melanoma, Multiple sclerosis, Lung squamous cell carcinoma, Tuberculosis	https://www.targetvalidation.org/target/ENSG00000156735/associations	TNFRSF1A TNFRSF1A APOBEC1	Cytoplasm,	No		✓
BAGE2	HUMAN B melanoma antigen 2	BAGE2	Q86Y30	NA	Others	BC101133	tongue squamous cell carcinoma, cancer, prostate carcinoma, hepatocellular carcinoma, breast carcinoma, Down syndrome, multiple myeloma	https://www.targetvalidation.org/target/ENSG00000187172/associations		Secreted	No		✓
BANK1	HUMAN B-cell scaffold protein with ankyrin repeats	BANK, FLJ20706	Q8NDB2	ENSG0000(	Others	AK091523	Autoimmune disease, Systemic lupus erythematous, Inflammatory bowel disease, Crohn's disease, C-reactive protein measurement, Rheumatoid arthritis, Diffuse scleroderma, Type I diabetes mellitus, Antiphospholipid syndrome, Systemic scleroderma	https://www.targetvalidation.org/target/ENSG00000153064/associations	ITPR2 LYN		No		✓
BASP1	HUMAN Brain acid soluble protein 1	NAP22	P80723	ENSG0000(	Others	AK295995	Ovarian carcinoma, Breast carcinoma, Bipolar disorder, Glioma, Pancreatic ductal adenocarcinoma, Bipolar disorder, Myeloid leukemia, Mobius syndrome, Liver carcinoma, Crohn's disease	https://www.targetvalidation.org/target/ENSG00000176788/associations	RIPK3	Cell membrane, Lipid-anchor, Cell projection, growth cone,	No		✓
BATF	HUMAN Basic leucine zipper transcriptional factor ATF-like	B-ATF, BATF1, SFA-2	Q16520	ENSG0000(	Transcription factor	AC007182	Multiple sclerosis, Primary immunodeficeincy, Rheumatoid arthritis, Common variable immunodeficiency, Autoimmune lymphoproliferative syndrome, Pancreatic ductal adenocarcinoma, Myeloid leukemia	https://www.targetvalidation.org/target/ENSG00000156127/associations	GSK3B	Nucleus	No		✓
BCKDK	HUMAN [3-methyl-2-oxobutanoate dehydrogenase [lipoamide]] kinase, mitochondrial	BCKD	O14874	ENSG0000(	Kinase	AC135050	Autism-epilepsy syndrome due to branched chain ketoacid dehydrogenase kinase deficiency, Parkinson's disease, Autism, Sepsis, Breast carcinoma, Epilepsy, Seizures, Microcephaly	https://www.targetvalidation.org/target/ENSG00000103507/associations	TRAF4 STAT3 BCKDHA DBT BLOC1S5 DLD	Mitochondrion matrix, Mitochondrion	Yes	https://www.drugbank.ca/biodb/polypeptides/O14874	✓
BCL10	HUMAN B-cell lymphoma/leukemia 10	c-E10, CARMEN, CIPER, CLAP, mE10	O95999	ENSG0000(	Cancer	AF127386	Lymphoid neoplasm, Lymphoma, Primary immunodeficiency, MALT lymphoma, Neoplasm of mature B-cells, Gastric lymphoma, Hyperhidrosis disorder, Severe combined immunodeficiency	https://www.targetvalidation.org/target/ENSG00000142867/associations	IKBKG TRAF6 MALT1 CARD9 CARD11 CARD19 BIRC3 RELA RELB MAPK9 AKT1 BCL3 RIPK2 MAP3K7 CHUK IKBKB MAP2K7 SOCS3 TLR4 MAP3K7 PRKCZ CASP9 MALT1 RELA IKBKB IKBKG TRAF6 CHUK MAP2K7	Cytoplasm, perinuclear region	No		✓
BCL2A1	HUMAN Bcl-2-related protein A1	BCL2L5, BFL1, GRS, HBPA1, B2LA1	Q16548	ENSG0000(	Signalling, Cancer	AY234180	Autoimmune disease, Inflammatory bowel disease, Obesity, Psoriasis, Leukemia, Rheumatoid arthritis, Graft-vs-Host disease, Atopic dermatitis, Crohn's disease, Myelodysplastic syndrome	https://www.targetvalidation.org/target/ENSG00000140379/associations	BBC3 BBC3 BECN1 PMAIP1 BAX GRB2 BID BCL2L11 PMAIP1	Cytoplasm,	Yes	https://www.bindingdb.org/uniprot/Q16548	✓
BCL2L14	HUMAN Apoptosis facilitator Bcl-2-like protein 14	BCL-G, BCLG, BL2L14	Q9BZR8	ENSG0000(	Others	AK292995	Azoospermia, Hypertriglyceridemia, Psoriasis, Asthma, Lung carcinoma, Narcolepsy, Benign prostatic hyperplasia, Breast carcinoma	https://www.targetvalidation.org/target/ENSG00000121380/associations	COPS5 MELK BCL2L1 TP53	Cytoplasm, Cytoplasm, cytosol,	No		✓
BDNF	HUMAN Brain-derived neurotrophic factor	BDNF	P23560	ENSG0000(	Kinase,Signalling,Cancer	X60201	Anorexia nervosa, Bulimia nervosa, Ondine syndrome, Haddad syndrome, Obesity, Alzheimer's disease, Schizophrenia, Nerve Degeneration, MODY	https://www.targetvalidation.org/target/ENSG00000176697/associations	SORT1 NTRK2 POU4F3 MBTPS1 NTF4 NTF3 CPE NCAM1 ESR1 CHEK2 NTRK2 NOS3 NTF3 CADPS2	Secreted	Yes	https://www.drugbank.ca/biodb/polypeptides/P23560	✓

BID	HUMAN BH3-interacting domain death agonist	BID	P55957	ENSG0000(	Others	AF087891	Prion disease, Hereditary central diabetes insipidus, Fatal infantile cytochrome C oxidase deficiency, Lung adenocarcinoma, Leukemia, Psoriasis, Adenocarcinoma, Gastric neoplasm, Schizophrenia	https://www.targetvalidation.org/target/ENSG00000015475/associations	MTCH2<br>VRK1<br>CASP10<br>BCL2L2<br>VRK3<br>VRK2<br>TTBK2<br>TTBK1<br>CASP8<br>ATM FADD<br>MCL1<br>BCL2L1<br>BAX<br>CSNK1G2<br>CSNK2A1<br>CSNK2B<br>MAP2K6<br>CSNK1E<br>CSNK1A1<br>FASLG<br>MAPK8<br>CTSK<br>CASP2<br>CASP3<br>CASP1<br>CTSS FAS<br>CSNK2A2<br>CAPN2<br>BCL2	Cytoplasm		No		✓
BIRC2	HUMAN Baculoviral IAP repeat-containing protein 2	API1, c-IAP1, cIAP1, hiap-2, MIHB, RNF48	Q13490	ENSG0000(	Signalling, Cancer	AK303197	Acute myeloid leukemia, Teratozoospermia, Dermatomyositis, Malignant peripheral nerve sheath tumor, cowpox, HIV infection, Multiple myeloma, Squamous cell carcinoma, Lung neoplasm	https://www.targetvalidation.org/target/ENSG00000110330/associations	IKBKG<br>TRAF6<br>FBXO7<br>EIF2AK3<br>DIABLO<br>TOLLIP<br>TSGA10<br>MAP3K14<br>BIRC7<br>MAVS<br>XAF1<br>TRADD<br>RIPK1<br>BIRC3<br>TRAF3<br>TRAF2<br>XIAP<br>EIF4G2<br>CDC42<br>CSE1L<br>GAS1<br>CASP3<br>TNFRSF1<br>B<br>TNFRSF1<br>A GSPT1<br>HSP90B1<br>TNF<br>MAP4K4	Cytoplasm, Nucleus,		Yes	https://www.bindingdb.org/uniprot/Q13490	✓
BIRC3	HUMAN Baculoviral IAP repeat-containing protein 3	API2, c-IAP2, cIAP2, hiap-1, MALT2, MIHC, RNF49	Q13489	ENSG0000(	Cancer	AF070674	Adenocarcinoma, Colorectal carcinoma, Neoplasm of mature B-cells, Clear cell renal carcinoma, Lung carcinoma, Gastric adenocarcinoma, Ovarian serous adenocarcinoma, Hepatocellular carcinoma, Ocular sebaceous carcinoma	https://www.targetvalidation.org/target/ENSG00000023445/associations	TRAF6<br>EIF2AK3<br>DIABLO<br>COMMD1<br>MAVS<br>XAF1<br>RIPK1<br>BIRC2<br>TRAF3<br>TRAF1<br>TRAF2<br>E2F1<br>UBE2N<br>NFKB1<br>TNFRSF1<br>A TP53<br>USP19<br>HTRA2<br>RIPK2<br>CHUK<br>IKBKB<br>TRAF3<br>BIRC5<br>CASP9<br>MAP3K14<br>CASP3<br>CASP7<br>RIPK2<br>UBE2D1	Cytoplasm		Yes	https://www.bindingdb.org/uniprot/Q13489	✓
BIRC5	HUMAN Baculoviral IAP repeat-containing protein 5	API4, EPR-1, survivin	O15392	ENSG0000(	Signalling, Cancer	AY830084	Astrocytoma, Glioma, Melanoma, Central nervous system cancer, Lung carcinoma, Liver carcinoma, Squamous cell carcinoma, Adenocarcinoma, Hepatocellular carcinoma	https://www.targetvalidation.org/target/ENSG00000089685/associations	DIABLO<br>INCENP<br>MAP3K5<br>AURKB<br>CDCA8<br>BIRC3<br>XIAP<br>CSNK2A1<br>PLK1<br>PRKACA<br>CDK4 SP1<br>HSP90AA1<br>CDK1<br>TP53<br>CASP9<br>CASP3<br>CASP7<br>AURKB<br>DIABLO<br>BECN1<br>INCENP<br>CDCA8<br>XIAP<br>BIRC2	Cytoplasm		Yes	https://www.bindingdb.org/uniprot/O15392	✓
BIRC7	HUMAN Baculoviral IAP repeat-containing protein 7	KIAP, ML-IAP, mliap, RNF50	Q96CA5	ENSG0000(	Others	AF311388	Central nervous system cancer, Lymphoma, Non-Hodgkin's lymphoma, Glioma, Gastric carcinoma,<br>Renal cell carcinoma, Bladder neoplasm, Osteosarcoma	https://www.targetvalidation.org/target/ENSG00000101197/associations	DIABLO<br>CASP9<br>CASP9<br>TAB1<br>CASP3<br>CASP7<br>DIABLO<br>BIRC2	Nucleus		Yes	https://www.bindingdb.org/uniprot/Q96CA5	✓
BLK	HUMAN Tyrosine-protein kinase Blk	MGC10442	P51451	ENSG0000(	Kinase,Cancer	CH471157	Leukemia, Acute lymphoblastic leukemia, MODY, Chronic myelogenous leukemia, Autoimmune disease, Rheumatoid arthritis, Obesity, Type II diabetes mellitus, Systemic lupus erythematosus, Mucocutaneous lymph node syndrome	https://www.targetvalidation.org/target/ENSG00000136573/associations	PAX5<br>RUNX1<br>CD79B<br>PLCG2<br>CD79A<br>BCR<br>PLCG2<br>EGFR<br>CD79A<br>BCL2<br>GAB1 KIT<br>ERBB2<br>CBL LCP1<br>CEACAM1<br>ITGA4<br>FCGR2A<br>BCKDHA<br>F3 UBE3A<br>MYF5<br>FCGR2B<br>CD79B<br>MYL4<br>BCAS2<br>CTR9	Cell membrane		Yes	https://www.bindingdb.org/uniprot/P51451	✓
BLZF1	HUMAN Golgin-45	JEM-1, GO45	Q9H2G9	ENSG0000(	Transcription factor	AF272386	Asthma, Gastric carcinoma, Melanoma, Adenocarcinoma, Hepatitis C infection, Colorectal adenocarcinoma, Osteoporosis, X-linked agammaglobulinemia, Kartagener syndrome	https://www.targetvalidation.org/target/ENSG00000117475/associations		Golgi apparatus lumen, Nucleus, Cytoplasm,		No		✓
BMP7	HUMAN Bone morphogenetic protein 7	OP-1	P18075	ENSG0000(	Kinase,Signalling, Cytokine/Chemokine	AL157414	Isolated anophthalmia-microphthalmia, Genetic disorder, Joubert syndrome with orofaciodigital defect, Shwachman-Diamond syndrome, Abetalipoproteinemia, Pulmonary fibrosis, Endometriosis	https://www.targetvalidation.org/target/ENSG00000101144/associations	SOSTDC1<br>BMPR2<br>ACVR2B<br>NOG<br>ACVR1<br>BMPR1A<br>ACVR2A<br>BMPR1B<br>ACVR1<br>ACTR2<br>BMPR1A<br>BMPR2<br>ACVR2B<br>BMPR1B<br>ACVR2A<br>SOSTDC1<br>TWSG1<br>GDF7	Secreted		No		✓
BMPR1A	HUMAN Bone morphogenetic protein receptor type-1A	ACVRLK3, ALK3, CD292, BMR1A	P36894	ENSG0000(	Kinase,Cancer	NA	Generalized juvenile polyposis, Adnocarcinoma, Colonic neoplasm, Inherited cancer-predisposing syndrome, Squamous cell lung carcinoma	https://www.targetvalidation.org/target/ENSG00000107779/associations	SMURF1<br>SMURF2<br>GDF6<br>BMPR2<br>ACVR2B<br>NOG<br>BAMBI<br>GDF5<br>BMP6<br>BMP5<br>BMP7<br>BMP4<br>BMP2<br>CTDNEP1<br>BMP10<br>SMAD6<br>AXIN1<br>SMAD7<br>RUVBL1<br>TAB1<br>PRKCD<br>SMAD1<br>SMAD5<br>HOXC8<br>BMP4<br>ZMYND11<br>ACVR1<br>ZAP70<br>SF3B4	Membrane, Single-pass type I membrane protein,	Transmembrane	Yes	https://www.bindingdb.org/uniprot/P36894	✓

BMPR1B	HUMAN Bone morphogenetic protein receptor type-1B	ALK6, CDw293, BMR1B	O00238	ENSG0000(	Kinase	AK313642	Brachydactyly type A2, Acromesomelic dysplasia, Brachydactyly type A1, Clear cell renal carcinoma, Townes-Brocks syndrome, Grebe type chondrodysplasia, Ulnar hypoplasia, Multiple synostoses syndrome	https://www.targetvalidation.org/target/ENSG00000138696/associations	STK35<br>AMHR2<br>BMPR2<br>ACVR2B<br>NOG PAK1<br>BAMBI<br>ROR2<br>GDF5<br>TGFBR1<br>BMPR1A<br>BMP6<br>BMP7<br>BMP4<br>BMP2<br>CDK4<br>BMP15<br>OXSR1<br>CDK14<br>SMAD6<br>SMAD7<br>MDFI<br>PHB2<br>CTTN<br>NEUROD1<br>STUB1<br>FANCL<br>NKIRAS1<br>SMAD1<br>SMAD5	Cell membrane Membrane, Single-pass type I membrane protein,	Transmembrane	Yes	https://www.bindingdb.org/uniprot/O00238	✓
BMPR2	HUMAN Bone morphogenetic protein receptor type-2	BMPR-II, BMPR3, BRK-3, PPH1, T-ALK	Q13873	ENSG0000(	Kinase	U20165	Pulmonary venoocclusive disease, Idiopathic pulmonary arterial hypertension, Vascular disease, Hypertension, Liver cirrhosis, Familial primary pulmonary hypertension, Hyperandrogenism	https://www.targetvalidation.org/target/ENSG00000204217/associations	SMURF1<br>SMURF2<br>GDF6<br>NOG<br>MERTK<br>TWF1<br>CSNK2A1<br>PPP2CA<br>PPP2CB<br>GDF5<br>MAP3K8<br>TGFBR1<br>BMPR1A<br>BMP6<br>BMP7<br>BMP4<br>BMP2<br>PRKCB<br>MOS<br>BMP15<br>CTDNEP1<br>GDF9<br>BMPR1B<br>TUBB SRC<br>TRPC1<br>CTBP1<br>CRYAB<br>RABGGTA	Cell membrane, Single-pass type I membrane protein,	Transmembrane	Yes	https://www.bindingdb.org/uniprot/Q13873	✓
BMX	HUMAN Cytoplasmic tyrosine-protein kinase BMX	ETK, PSCTK3	P51813	ENSG0000(	Kinase	CH471074	Arthritis, Sepsis, Osteoarthritis, Lung carcinoma, Sjogren's syndrome, Liver carcinoma, Lymphoproliferative disorder, Breast carcinoma, Small cell lung carcinoma	https://www.targetvalidation.org/target/ENSG00000102010/associations	LIMK1<br>DYRK4<br>PAK1 ITK<br>BTK PTK2<br>RAP1A<br>BCAR1<br>TEC<br>CASP3<br>KDR<br>FGFR4<br>SRC PIM1<br>ERBB2<br>EGFR<br>CAV1<br>FGFR1<br>CTTN<br>STAT5A<br>TP53<br>BAK1<br>CASP3<br>STAT1<br>CRK MET<br>FAS GAB1<br>EIF4B<br>BCAR1<br>PAK1<br>HNRNPK<br>CBL<br>PPP1R13L<br>STAT3	Cytoplasm		Yes	https://www.bindingdb.org/uniprot/P51813	✓
BOLA3	HUMAN BolA-like protein 3	BOLA3	Q53S33	ENSG0000(	Others	CH471053	Fatal multiple mitochondrial dysfunction syndrome type 2, Fatal multiple mitochondrial dysfunction syndrome, Multiple myeloma, Malignant peripheral nerve sheath tumor, Meningococcal infection, Nonketotic hyperglycinemia, Glycine encephalopathy, Malignant peripheral nerve sheath tumor	https://www.targetvalidation.org/target/ENSG00000163170/associations		Mitochondrion		No		✓
BOP1	HUMAN Ribosome biogenesis protein BOP1	KIAA0124	Q14137	ENSG0000(	Cancer,Cancer-Antigen	BC001086	Breast carcinoma, Melanoma, Hepatocellular carcinoma, Ovarian carcinoma, T-cell acute lymphoblastic leukemia, Squamous cell carcinoma, Hypertension, Rectal carcinoma, Glioblastoma multiforme, Heredtary spastic paraplegia	https://www.targetvalidation.org/target/ENSG00000261236/associations	PES1	Nucleus, nucleolus		No		✓
BPI	HUMAN Bactericidal permeability-increasing protein	BPIFD1	P17213	ENSG0000(	Others	AL359555	Osteosarcoma, Azoospermia, Septic shock, Meningococcal infection, Mental or behavioural disorder, Ulcerative Ccolitis, Cystic fibrosis, Rheumatoid arthritis, Crohn's disease	https://www.targetvalidation.org/target/ENSG00000101425/associations	PRSS2	Secreted		Yes	https://www.drugbank.ca/biodb/polypeptides/P17213	✓
BPIFA1	HUMAN BPI fold-containing family A member 1	LUNX, NASG, PLUNC, SPLUNC1, SPURT	Q9NP55	ENSG0000(	Others	AB024937	X-linked mendelian susceptibility to mycobacterial diseases, Agammaglobulinemia, Isolated agammaglobulinemia, Immunodeficiency due to a complement cascade protein anomaly, Fetal alcohol spectrum disorder, Cystic Fibrosis, Adenocarcinoma, Liver carcinoma, Allergic rhinitis, Nasopharyngeal carcinoma	https://www.targetvalidation.org/target/ENSG00000198183/associations		Secreted		No		✓
BRAF	HUMAN Serine/threonine-protein kinase B-raf	BRAF1	P15056	ENSG0000(	Kinase,Cancer	AC006347	Colorectal neoplasm, Lymphoma, Lung carcinoma, Central nervous system cencer, Acute myeloid leukemia, Noonan syndrome, Craniopharyngioma, Colonic neoplasm, LEOPARD syndrome, Ductal adenocarcinoma	https://www.targetvalidation.org/target/ENSG00000157764/associations	AKT3<br>SGK2<br>SGK3<br>KSR1<br>RHEB<br>PDK1<br>PAK2<br>PAK1<br>YWHAH<br>MAP2K1<br>YWHAZ<br>RAP1A<br>YWHAG<br>RAP1B<br>PRKX<br>GSK3B<br>IQGAP1<br>MAP2K2<br>SFN<br>YWHAB<br>AKT2<br>AKT1<br>PEBP1<br>MAPK1<br>MAPK3<br>YWHAQ<br>PRKACA<br>PRKCA<br>ARAF	Nucleus		Yes	https://www.bindingdb.org/uniprot/P15056	✓

BRD2	HUMAN Bromodomain-containing protein 2	KIAA9001, RING3	P25440	ENSG0000	Cancer,Cancer-Antigen	Z96104	Astrocytoma, Glioblastoma multiforme, Berardinelli-Seip congenital lipodystrophy, Familial partial lipodystrophy, Perlman syndrome, Primary lipodystrophy, Spina bifida-hypospadias, Adenoma, Wolcott-Rallison syndrome	https://www.targetvalidation.org/target/ENSG00000204256/associations	RB1 E2F1 KDM5A RB1 RBL2 HIST1H4A; HIST1H4B; HIST1H4C; HIST1H4D; HIST1H4E; HIST1H4F; HIST1H4H; HIST1H4I; HIST1H4J; HIST1H4K; HIST1H4L; HIST2H4A; HIST2H4B; HIST4H4 BRD7 E2F2 HIST3H2BB	Nucleus	Yes	https://www.bindingdb.org/uniprot/P25440	✓
BRD3	HUMAN Bromodomain-containing protein 3	KIAA0043, RING3L	Q15059	ENSG0000	Kinase,Cancer	AL445931	Melanoma, Cutaneous melanoma, Squamous cell carcinoma, Gastric adenocarcinoma, Gastric intestinal type adenocarcinoma, Rheumatoid arthritis, Juvenile amyotrophic lateral sclerosis 4, Colorectal adenocarcinoma, Bladder carcinoma, Medulloblastoma	https://www.targetvalidation.org/target/ENSG00000169925/associations	BRD7	Nucleus	Yes	https://www.bindingdb.org/uniprot/Q15059	✓
BRD8	HUMAN Bromodomain-containing protein 8	SMAP, SMAP2, TrCP120	Q9H0E9	ENSG0000	Transcription factor	AC109442	Melanoma, Adenocarcinoma, Azoospermia, Teratozoospermia, Dermatomyositis, Endometriosis, Alzheimer's disease, Breast carcinoma, Ductal carcinoma, Colorectal adenocarcinoma	https://www.targetvalidation.org/target/ENSG00000112983/associations	TRRAP MORF4L1 ING3 MRGBP DMAP1 WEE1 PPARG YEATS4	Nucleus	Yes	https://www.bindingdb.org/uniprot/Q9H0E9	✓
BRDT	HUMAN Bromodomain testis-specific protein	BRD6, CT9	Q58F21	ENSG0000	Others	AK302758	Li-Fraumeni syndrome, Azoospermia, Congenital bilateral absence of vas deferens, Waardenburg syndrome, Primary ciliary dyskinesia, Male infertility with teratozoospermia due to single gene mutation, Non-syndromic male infertility due to sperm motility disorder, Male infertility with azoospermia or oligozoospermia due to single gene mutation, Deafness-infertility syndrome	https://www.targetvalidation.org/target/ENSG00000137948/associations	HIST1H4A; HIST1H4B; HIST1H4C; HIST1H4D; HIST1H4E; HIST1H4F; HIST1H4H; HIST1H4I; HIST1H4J; HIST1H4K; HIST1H4L; HIST2H4A; HIST2H4B; HIST4H4	Nucleus	Yes	https://www.bindingdb.org/uniprot/Q58F21	✓
BRSK1	HUMAN Serine/threonine-protein kinase BRSK1	KIAA1811	Q8TDC3	ENSG0000	Kinase	AF479826	Premature menopause, Pancreatic ductal adenocarcinoma, Peutz-Jeghers syndrome, Colorectal cancer, Primary ovarian insufficiency, Polyp, Melanoma, Alzheimer's disease, Breast carcinoma	https://www.targetvalidation.org/target/ENSG00000160469/associations	CAMKK1 STK11 PAK1 WEE1 CDC25C CDC25B PRKAA1 TUBG1	Cytoplasm	Yes	https://www.bindingdb.org/uniprot/Q8TDC3	✓
BRSK2	HUMAN Serine/threonine-protein kinase BRSK2	C11orf7, PEN11B, SADA, STK29, HUSSY-12	Q8IWQ3	ENSG0000	Kinase	AF533878	Li-Fraumeni syndrome, Ependymoma, Prostate carcinoma, Monoclonal gammopathy, Pancreatic ductal adenocarcinoma, Limbic encephalitis, Hepatocellular carcinoma	https://www.targetvalidation.org/target/ENSG00000174672/associations	STK11 VCP WEE1 PRKACA WEE1 COPS5 CDC25C CDC25B PRKAA1	Cytoplasm, cytoskeleton, microtubule organizing center, centrosome, perinuclear region, Endoplasmic reticulum,	Yes	https://www.bindingdb.org/uniprot/Q8IWQ3	✓
BTF3	HUMAN Transcription factor BTF3	BTF3a, BTF3b, NACB, OK/SW-cl.8	P20290	ENSG0000	Transcription factor	X74070	Teratozoospermia, Ovarian carcinoma, Melanoma, Multiple myeloma, Burkitts lymphoma, Myocardial infarction, Glioblastoma, Ataxia telangiectasia, Pancreatic ductal adenocarcinoma	https://www.targetvalidation.org/target/ENSG00000145741/associations	ESR1 NACA GAL EPHB2 CSNK2B	Cytoplasm	No		✓
BTG1	HUMAN Protein BTG1	BTG1	P62324	ENSG0000	Cancer	BC016759	Diffuse large B-cell lymphoma, Neoplasm of mature B-cells, Renal cell carcinoma, Squamous cell carcinoma, Cutaneous melanoma, Lymphoid neoplasm, Hepatocellular carcinoma, Multiple myeloma, Multiple sclerosis, Juvenile arthritis	https://www.targetvalidation.org/target/ENSG00000133639/associations	CDK2 CCNA2 PRMT1 HOXB9		No		✓
BTG3	HUMAN Protein BTG3	ANA, TOB55	Q14201	ENSG0000	Others	CH471079	Breast carcinoma, Pancreatic carcinoma, Prostate carcinoma, Melanoma, Hepatocellular carcinoma, Adenocarcinoma, Systemic lupus erythematosus, Gastric carcinoma, Hypertriglyceridemia, Ovarian carcinoma	https://www.targetvalidation.org/target/ENSG00000154640/associations	E2F1 BANP		No		✓

BTRC	HUMAN F-box/WD repeat-containing protein 1A	beta-TrCP1, betaTrCP, bTrCP, FBXW1A, Fwd1	Q9Y297	ENSG0000(	Signalling	GU727631	HIV infection, Balckfan-Diamond anemia, Hereditary elliptocytosis, Lamellar ichthyosis, Atypical hemolytic-uremic syndrome, Congenital dyserythropoietic anemia, Hereditary spherocytosis, Oligodactyly, Ectrodactyly	https://www.targetvalidation.org/target/ENSG00000166167/associations	FBXW11 IGF2BP1 RASSF1 CLSPN UHRF1 TRIB2 RASSF5 AMER1 FGD3 MAPK14 NFE2L2 NFKBIB CUL1 SMAD4 REST BST2 NFKB2 FGD1 SMAD3 SKP1 RBX1 VCP PSMD4 RCAN1 GSK3B HINT1 CDC34 CSNK1A1 CASP3 KDR	Cytoplasm	No		✓
BUB1	HUMAN Mitotic checkpoint serine/threonine-protein kinase BUB1	BUB1A, BUB1L, hBUB1	O43683	ENSG0000(	Kinase	AF047471	Glycogen storage disease, Glycogen storage disease due to aldolase A deficiency, Breast carcinoma, Lung carcinoma, Hepatocellular carcinoma, Micrognathism, Muscular dystrophy, Hypoplastic mandible condyle	https://www.targetvalidation.org/target/ENSG00000169679/associations	KNL1 ATM CDC20 PLK1 CDK1 APC BUB1B MAD1L1 LIG1 AP1B1 SMC1A CRK EIF4EBP1 ZFP36 CDC20 XRCC1 PPP2R5D SMARCA4 PLK1 NCL CDK1 MDM2 PPP1R13L SKP1 FZR1 MDM4 PPP1R8 HIST1H2AG; HIST1H2AI; HIST1H2A	Nucleus, Chromosome, centromere, kinetochore,	Yes	https://www.bindingdb.org/uniprot/O43683	✓
BUB1B	HUMAN Mitotic checkpoint serine/threonine-protein kinase BUB1 beta	Bub1A, BUBR1, MAD3L, SSK1	O60566	ENSG0000(	Kinase,Cancer	AF107297	Melanoma, Mosaic varigated aneuploidy syndrome, Lung carcinoma, Esophageal squamous cell carcinoma, Colorectal adenocarcinoma, Dandy-Walker syndrome, Rhabdomyosarcoma, Microcephaly	https://www.targetvalidation.org/target/ENSG00000156970/associations	FZR1 ANAPC4 ANAPC7 ANAPC1 KAT2B KNL1 MAD2L1 CDC16 CDC20 CDK5 PLK1 TTK CDC27 CDK1 BUB3 BUB1 TP73 MAP3K4 MAD2L1 PLK3 AP1B1 PARP1 CENPE CDC20 PTTG1 CDK1 ANAPC7 FZR1 BRCA2 SNCG	Cytoplasm, Nucleus, Chromosome, centromere, kinetochore, cytoskeleton, microtubule organizing center, centrosome,	No		✓
BUD31	HUMAN Protein BUD31 homolog	Cwc14, EDG-2, EDG2, fSAP17, G10, YCR063W	P41223	ENSG0000(	Transcription factor	AK316477	Ovarian carcinoma, Prostate carcinoma, Glioblastoma multiforme, Breast carcinoma, Hepatocellular carcinoma, Schistosomiasis, Inherited neuropathy, Osteosarcoma	https://www.targetvalidation.org/target/ENSG00000106245/associations	CDC42 PRPF19	Nucleus	No		✓
C1D	HUMAN Nuclear nucleic acid-binding protein C1D	LRP1, Rrp47, SUN-CoR, SUNCOR	Q13901	ENSG0000(	Transcription factor	CH471053	Restless leg syndrome, Age-related nuclear cataract, Systemic scleroderma, Breast carcinoma, Ovarian carcinoma, Alzheimer's disease, Paucibacillary leprosy, Senile Plaque, Prostate carcinoma, Dermatomyositis	https://www.targetvalidation.org/target/ENSG00000197223/associations	PRKDC TSNAX NR1D1	Nucleus	No		✓
C1orf174	HUMAN UPF0688 protein C1orf174	CA174	Q8IYL3	ENSG0000(	Others	CH471130	Multiple system atrophy, Migraine disorder, Breast carcinoma, Asthma, Response to diisocyanate, Hepatocellular carcinoma, Melanoma, Atopic eczema, Dermatomyositis, Pancreatic carcinoma	https://www.targetvalidation.org/target/ENSG00000198912/associations		Nucleus	No		✓
C1orf216	HUMAN UPF0500 protein C1orf216	CA216	Q8TAB5	ENSG0000(	Others	CH471059	Oligodenedroglioma, Ependymoma, Acute lymphoblastic leukemia, Astrocytoma, Medulloblastoma, Primitive neuroectodermal tumor, Squamous cell lung carcinoma, Breast carcinoma, Ulcerative colitis, Benign prostatic hyperplasia	https://www.targetvalidation.org/target/ENSG00000142686/associations			No		✓
C21orf2	HUMAN Protein C21orf2	LRRC76, YF5, CU002	O43822	ENSG0000(	Others	U84570	Cone rod dystrophy, Jeune syndrome, Axial spondylometaphyseal dysplasia, Ciliopathy, Li-Fraumeni syndrome, Retinal dystrophy, Friedreich ataxia, Retinal Dystrophy	https://www.targetvalidation.org/target/ENSG00000160226/associations		Mitochondrion	No		✓
C21orf33	HUMAN ES1 protein homolog, mitochondrial	ES1, GT335, HES1, KNPI	P30042	ENSG0000(	Others	U53007	Dementia, Lewy body dementia, Vascular dementia, Alzheimer's disease, Idiopathic pulmonary fibrosis, Down syndrome, Neuroblastoma, Endometrial carcinoma, Primary sclerosing cholangitis	https://www.targetvalidation.org/target/ENSG00000160221/associations		Mitochondrion	No		✓

C4BPA	HUMAN C4b-binding protein alpha chain	C4BP	P04003	ENSG0000(	Others	CH471100	Hepatocellular carcinoma, Lung carcinoma, Epithelial neoplasm, Colorectal cancer, Ovarian carcinoma, Prion disease, Hemolytic -Uremic syndrome, Creutzfeldt-Jakob disease, Schizophrenia	https://www.targetvalidation.org/target/ENSG00000123838/associations	BMPR2 APCS PROS1 LRP1 C4B; C4B_2	Secreted		No		✓
C6	HUMAN Complement component C6	C6	P13671	ENSG0000(	Cancer	AB126592	Immunodeficiency due to late component of complements deficiency, Immunodeficiency due to complement cascade protein anomaly, Central nervous system cancer, Colorectal cancer, Azoospermia, Psoriasis, Hepatocellular carcinoma	https://www.targetvalidation.org/target/ENSG00000039537/associations	C5 F2	Secreted		No		✓
C6orf106	HUMAN Uncharacterized protein C6orf106	CF106	Q9H6K1	ENSG0000(	Others	AL031577, AL451165	Azoospermia, Walker-Warburg syndrome, Postaxial acrofacial dysostosis, Waardenburg syndrome, High density lipoprotein cholesterol measurement, Hereditary hyperekplexia, Crohn's disease, Lung carcinoma, Tay-Sachs disease	https://www.targetvalidation.org/target/ENSG00000196821/associations	CDK2			No		✓
CA1	HUMAN Carbonic anhydrase 1	CA1	P00915	ENSG0000(	Signalling	NA	Epilepsy, Glaucoma, Brain disease, Obstructive sleep apnea, Heart disease, Altitude sickness, Pneumoconiosis, Colorectal cancer, Liver cirrhosis, Osteoarthritis	https://www.targetvalidation.org/target/ENSG00000133742/associations		Cytoplasm,		Yes		✓
													https://www.bindingdb.org/uniprot/P00915	
CA4	HUMAN Carbonic anhydrase 4	CAIV, Car4, RP17, CAH4	P22748	ENSG0000(	Others	AK289715	Retinal dystrophy, Retinitis pigmentosa, Epilepsy, Glaucoma, Prader-Willi syndrome, Obstructive sleep apnea, Migraine disorder, Altitude sickness, Obsessive-compulsive disorder, Anxiety disorder	https://www.targetvalidation.org/target/ENSG00000167434/associations	SLC4A4	Cell membrane, Lipid-anchor, GPI-anchor,		Yes		✓
													https://www.bindingdb.org/uniprot/P22748	
CA9	HUMAN Carbonic anhydrase 9	CAIX, MN, G250, CAH9	Q16790	ENSG0000(	Others	AL357874, AL133410	Renal carcinoma, Epithelial carcinoma, Breast carcinoma, Central nervous system cancer, Tongue squamous cell carcinoma, Hypoxia, Anaplasia, Anoxia, Head and Neck neoplasia, Medulloblastoma	https://www.targetvalidation.org/target/ENSG00000107159/associations	PRKACA	Nucleus	Transmembrane	Yes		✓
													https://www.bindingdb.org/uniprot/Q16790	
CAB39L	HUMAN Calcium-binding protein 39-like	bA103J18.3, FLJ12577, MO2L, CB39L	Q9H9S4	ENSG0000(	Cancer,Cancer-Antigen	BC010993	Blackfan-Diamond syndrome, Hereditary elliptocytosis, Beta-Thalassemia associated with another hemoglobin anomaly, Hereditary spherocytosis, Rh deficiency syndrome, Endometriosis, Acute monocytic leukemia, Glutathione synthetase deficiency, Azoospermia	https://www.targetvalidation.org/target/ENSG00000102547/associations				No		✓
CABYR	HUMAN Calcium-binding tyrosine phosphorylation-regulated protein	CBP86, CT88, FSP-2	O75952	ENSG0000(	Others	AF295039	Colonic neoplasm, Li-Fraumeni syndrome, Colorectal adenocarcinoma, Azoospermia, Malignant mesothelioma, Lung carcinoma, Cystic fibrosis, Ovarian carcinoma	https://www.targetvalidation.org/target/ENSG00000154040/associations	GSK3B	Cytoplasm, cytoskeleton, Cell projection, cilium, flagellum,		No		✓
CAGE1	HUMAN Cancer-associated gene 1 protein	CT3	Q8TC20	ENSG0000(	Cancer	BC026194	Azoospermia, Leukemia, Bladder carcinoma, Signet ring cell carcinoma, Rectum cancer, Hernia, Adenocarcinoma, Transitional cell carcinoma	https://www.targetvalidation.org/target/ENSG00000164304/associations				No		✓
CALM2	HUMAN Calmodulin-2	CAM2, CAMB	P0DP24	ENSG0000(	Signalling, Cancer	BT009916	Familial long QT syndrome, Prolonged QT interval, teratozoospermia, diphtheria, hearing loss, cancer	https://www.targetvalidation.org/target/ENSG00000143933/associations		Cytoplasm, cytoskeleton, spindle		No		✓

CALM3	HUMAN Calmodulin -3	CAKLML2, CAM3, CAMC, CAMIII	P0DP25	ENSG0000(	Signalling, Cancer	AC093503	Neoplasm, astrocytoma, glioblastoma multiforme, psoriasis, lung carcinoma, large cell lung carcinoma, oligodendroglioma	https://www.targetvalidation.org/target/ENSG00000160014/associations		Cytoplasm, cytoskeleton, spindle	No		✓
CALML4	HUMAN Calmodulin -like protein 4	NY-BR-20, CALL4	Q96GE6	ENSG0000(	Cancer,Cancer-Antigen	BX640817	Breast carcinoma, Reunion Island's Larsen syndrome, Inflammation	https://www.targetvalidation.org/target/ENSG00000129007/associations			No		✓
CALR	HUMAN Calreticulin	cC1qR, CRT, FLJ26680, RO, SSA	P27797	ENSG0000(	Cancer	AB451408	Myeloid neoplasm, Adenocarcinoma, Acute leukemia, Myelodysplastic syndrome, Essential thrombocythemia, Myelofibrosis, Thrombocytosis, Myeloproliferative disease, Melanoma, Polycythemia vera	https://www.targetvalidation.org/target/ENSG00000179218/associations	PDIA6<br>LRP1<br>TAP2<br>TAP1 B2M<br>ITGA3<br>TRIM21<br>IL7R PRF1<br>HLA-E<br>MBL2 AR<br>ITGA2B<br>THBS1<br>ITGAV<br>APP<br>NR3C1<br>APOB FGB<br>PLAT<br>GABARAP<br>TAPBP<br>NKX2-1<br>AR LPL<br>TAP1<br>PDIA3<br>P4HB<br>TAPBP<br>TAP2<br>FBN1<br>SLC6A4<br>PDIA2<br>SCARF1	Endoplasmic reticulum lumen	Yes	https://www.drugbank.ca/biodb/polypeptides/P27797	✓
CALR3	HUMAN Calreticulin -3	CRT2, CT93, FLJ25355, MGC26577	Q96L12	ENSG0000(	Others	CH471106	Hypertrophic cardiomyopathy, Rare familial disorderwith hypertrophic cardiomyopathy, Breast carcinoma, Lung carcinoma, Melanoma, Infertility, Cataract	https://www.targetvalidation.org/target/ENSG00000269058/associations		Endoplasmic reticulum lumen	No		✓
CALU	HUMAN Calumenin	CALU, Crocalbin	O43852	ENSG0000(	Cancer,Cancer-Antigen	HM002604	Self-reported educational attainment, Melanoma, Prostate carcinoma, Lung carcinoma, Psoriasis,<br>Calcinosis, Tumor necrosis, Colon carcinoma, Osteosarcoma, Warfarin sensitivity disorder	https://www.targetvalidation.org/target/ENSG00000128595/associations	PLK2<br>PLK3<br>CSNK2A1<br>APCS	Endoplasmic reticulum membrane	No		✓
CAMK1	HUMAN Calcium/calmodulin-dependent protein kinase type 1	CaMKI, KCC1A	Q14012	ENSG0000(	Kinase	NA	Leukemia, Acute myeloid leukemia, Diffuse large B-cell lymphoma, Hepatocellular carcinoma, Adrenocortical carcinoma, Burkitt lymphoma, Medulloblastoma, Sepsis	https://www.targetvalidation.org/target/ENSG00000134072/associations	CAMKK2<br>CAMK1G<br>CAMKK1<br>CAMK1D<br>CAMK4<br>PTK6<br>PPP2R5C<br>PPP2CA<br>PPP2CB<br>PPM1F<br>NUMB<br>CALM1<br>SYN1<br>EIF4G3<br>NOS1<br>MARK2<br>EP300<br>GAPDH<br>KRT18<br>CAMK2A<br>CREB1<br>CAMKK2<br>ATF1<br>CAMKK1<br>MYL9<br>ARHGEF7<br>HDAC5<br>MS4A2<br>RPS19	Cytoplasm	Yes	https://www.bindingdb.org/uniprot/Q14012	✓
CAMK1G	HUMAN Calcium/calmodulin-dependent protein kinase type 1G	CLICK3, VWS1, KCC1G	Q96NX5	ENSG0000(	Kinase	AL03754	Central nervous system cancer, Astrocytoma, Glioblastoma multiforme, Amyotrophic lateral sclerosis, Obesity, Cervical carcinoma, Papillary thyroid carcinoma, Benign prostatic hyperplasia, Van der Woude syndrome	https://www.targetvalidation.org/target/ENSG00000008118/associations	ARRB2<br>EIF4G3<br>EP300<br>CAMK1	Cytoplasm	Yes	https://www.bindingdb.org/uniprot/Q96NX5	✓
CAMK2A	HUMAN Calcium/calmodulin-dependent protein kinase type II subunit alpha	CAMKA, CaMKIINalpha, KIAA0968, KCC2A	Q9UQM7	ENSG0000(	Kinase	AC011372	Astrocytoma, Central nervous system cancer, Ependymoma, Primitive neuroectodermal tumor, Bipolar disorder, Unipolar depression, Alzheimer's disease, Hyperalgesia	https://www.targetvalidation.org/target/ENSG00000070808/associations	CABP1<br>RCHY1<br>MYRIP<br>CAMK4<br>SMAD2<br>NCAPH<br>CAMK1<br>GRIN2A<br>ACTN3<br>PLCB3<br>SMAD3<br>PPP2CA<br>PPP2CB<br>CALM1<br>PPP1CB<br>PPP1CA<br>PPM1F<br>PPP1CC<br>GRK5<br>EIF2AK2<br>RAF1<br>GNRH1<br>PLCB3<br>GRIN2B<br>STX3<br>RYR1<br>ACTN1<br>GRIN1<br>STMN1	Cell junction, synapse, presynaptic cell membrane	Yes	https://www.bindingdb.org/uniprot/Q9UQM7	✓
CAMK2B	HUMAN Calcium/calmodulin-dependent protein kinase type II subunit beta	CAM2, CAMK2, CAMKB, KCC2B	Q13554	ENSG0000(	Kinase,Signalling	AF083419	Central nervous system cancer, Astrocytoma, Osteoarthritis, Breast carcinoma, Glioblastoma multiforme, Metabolic syndrome, Hepatocellular carcinoma, Schizophrenia, Marijuana Abuse	https://www.targetvalidation.org/target/ENSG00000058404/associations	CDC20<br>PLCB3<br>PPP2CA<br>PPP2CB<br>CALM1<br>GNRH1<br>PLCB3<br>GRIN2B<br>STMN1<br>CTNNB1<br>CALM1<br>PRKACA<br>MAP3K7<br>CYLD<br>TRPC5<br>GSK3A<br>STAT1<br>GAPDH<br>SMAD2<br>ETS1<br>PRKAA1<br>ACTN4<br>DLGAP1<br>GRIN2A<br>ITPKA<br>CAMK2D<br>COL4A3<br>CAMK2N2	Cytoplasm, cytoskeleton	Yes	https://www.bindingdb.org/uniprot/Q13554	✓

CAMK2D	HUMAN Isoform Delta 6 of Calcium/calmodulin-dependent protein kinase type II subunit delta	CAMKD, KCC2D	Q13557	ENSG0000(	Kinase	AY987011	Chronic lymphocytic leukemia, Ovarian carcinoma, Lung carcinoma, Ishemia, Lymphoma, Osteosarcoma, Myocardial ischemia, Seizures, Epilepsy	https://www.targetvalidation.org/target/ENSG00000145349/associations	CAMK2B<br>PLCB3<br>CALM1<br>PLCB3<br>GRIN2B<br>ACTN2<br>RYR2<br>ATP2A2<br>CALM1<br>CTTN<br>PRKACA<br>FLNA<br>MAP3K7<br>PRKCD<br>CFL2<br>HDAC4<br>SMAD4<br>GFAP<br>PPP1R14A<br>STAT1<br>HSF1<br>KRT18<br>IKBKG<br>CREB1<br>VIM<br>CEBPB<br>PEA15<br>CEACAM1<br>KCND2	Cell membrane, sarcolemma	Yes	https://www.bindingdb.org/uniprot/Q13557	✓
CAMK2G	HUMAN Calcium/calmodulin-dependent protein kinase type II subunit gamma	CAMKG, CAMK, CAMK-II	Q13555	ENSG0000(	Kinase,Signalling	AL596247, AC022400, AL713896	Psoriasia, Psoriasis vulagris, Glioblastoma multiforme, Astrocytoma, Chronic lymphocytic leukemia, Myeloid leukemia, Anaplastic astrocytoma, Hypoplastic left heart syndrome, Type II diabetes mellitus	https://www.targetvalidation.org/target/ENSG00000148660/associations	PLCB3<br>PPP2CA<br>PPP2CB<br>CALM1<br>PPP1CB<br>PPP1CA<br>PPP1CC<br>MAP3K7<br>CTDSPL<br>PLCB3<br>GRIN2B<br>RYR1<br>ACTN2<br>EGFR<br>CALM1<br>PRKACA<br>FLNA<br>SYN1<br>MAP3K7<br>NCOR2<br>SMAD4<br>STAT1<br>SMAD3<br>ATF1<br>TIAM1<br>CAMKK1<br>PEA15<br>MYLK<br>HDAC5	Sarcoplasmic reticulum membrane	Yes	https://www.bindingdb.org/uniprot/Q13555	✓
CAMK4	HUMAN Calcium/calmodulin-dependent protein kinase type IV	CaMK-GR, CAMK, CAMKIV	Q16566	ENSG0000(	Kinase	CH471086	Central nervous system cancer, Astrocytoma, Azoospermia, Ependymoma, Li-Fraumeni syndrome, Systemic lupus erythematosus, Oligospermia, Narcolepsy	https://www.targetvalidation.org/target/ENSG00000152495/associations	CAMKK2<br>PPM1E<br>CAMKK1<br>CAMK1<br>PPP2R5C<br>PPP2CA<br>PPP2CB<br>CALM1<br>PPM1F<br>PLCB3<br>STMN1<br>PHB2<br>ATP2A2<br>CALM1<br>SYN1<br>NOS1<br>HDAC4<br>KRT8<br>EP300<br>GAPDH<br>RELA<br>HSF1<br>KRT18<br>PPP1R12A<br>PPP2R5D<br>CAMK2A<br>CREB1<br>HDAC8<br>CAMKK2	Cytoplasm, Nucleus,	Yes	https://www.bindingdb.org/uniprot/Q16566	✓
CAMKK1	HUMAN Calcium/calmodulin-dependent protein kinase kinase 1	CAMKKA	Q8N5S9	ENSG0000(	Kinase	BC031647	Breast carcinoma, Glioblastoma multiforme, Autism, Sepsis, Prostate carcinoma, Lung neoplasm, Schizophrenia	https://www.targetvalidation.org/target/ENSG00000004660/associations	STK17A<br>NRBP2<br>DYRK4<br>CAMKK2<br>AURKB<br>DYRK2<br>HIPK4<br>CAMK1D<br>HIPK1<br>MAP3K11<br>CAMK4<br>RPS6KA2<br>CAMK1<br>CAMK2G<br>PAK1<br>PRKCD<br>CDK5<br>CDK6<br>SRPK2<br>CALM1<br>PRKAA2<br>PLK1<br>PRKX<br>CDK7<br>CLK2<br>MAP3K8<br>AKT1<br>WEE1<br>RPS6KB1	Cytoplasm	Yes	https://www.bindingdb.org/uniprot/Q8N5S9	✓
CAMKK2	HUMAN Calcium/calmodulin-dependent protein kinase kinase 2	CAMKK, CAMKKB, KIAA0787	Q96RR4	ENSG0000(	Kinase	AF321385	Glioma, Berardinelli-Seip congenital lipodystrophy, Astrocytoma, Glioblastoma multiforme, Ependymoma	https://www.targetvalidation.org/target/ENSG00000110931/associations	DAPK2<br>STK17A<br>CAMK4<br>CAMK1<br>CDK5<br>CALM1<br>DAPK1<br>PLK1<br>GSK3B<br>GSK3A<br>PRKACA<br>STK17B<br>DAPK3<br>SGK1<br>PRKAA2<br>CALM1<br>PRKACA<br>PRKAG1<br>AKT1<br>PIK3C3<br>CAMKK1<br>PRKAA1<br>CAMK1<br>CAMK4<br>CAMK1D	Nucleus	Yes	https://www.bindingdb.org/uniprot/Q96RR4	✓
CAMKV	HUMAN CaM kinase-like vesicle-associated protein	MGC8407, VACAMKL	Q8NCB2	ENSG0000(	Kinase	AL136697	Central nervous system cancer, Glioblastoma, Astrocytoma, Reproductive behaviour measurement, Self-reported educational attainment, Schizophrenia, Ependymoma	https://www.targetvalidation.org/target/ENSG00000164076/associations	TCEA2	Cell membrane	No		✓
CAPG	HUMAN Macrophage-capping protein	AFCP, MCP	P40121	ENSG0000(	Others	CH471053	Chronic granulomatous disease, Agammaglobulinemia, Lung carcinoma, Leukocyte adhesion deficiency type I, Blau syndrome, Renal cell carcinoma, Lung adenocarcinoma, Keloid, Familial hypercholesterolemia	https://www.targetvalidation.org/target/ENSG00000042493/associations	NCAPH	Nucleus	No		✓
CARD8	HUMAN Caspase recruitment domain-containing protein 8	CARDINAL, KIAA0955, NDPP, TUCAN	Q9Y2G2	ENSG0000(	Transcription factor	AY026322	Neuroblastoma, Breast carcinoma, Cervical carcinoma, Melanoma, Gout, Rheumatoid arthritis, Crohn's disease, Alzheimer's disease	https://www.targetvalidation.org/target/ENSG00000105483/associations	NLRP2<br>NOD2<br>CARD6<br>NLRP3<br>RFWD2<br>CARD17<br>FHL2<br>PTCH1<br>CARD18<br>CASP9<br>CASP1<br>CASP1<br>CARD16<br>CASP9<br>IKBKG<br>APAF1	Cytoplasm	No		✓
CARD9	HUMAN Caspase recruitment domain-containing protein 9	CARD9	Q9H257	ENSG0000(	Others	AL592301	Chronic mucocutaneous candidosis, Autoimmune disease, Crohn's disease, Ulterative colitis, Primary immunodeficiency, IGA Glomerulonephritis, Arthritis	https://www.targetvalidation.org/target/ENSG00000187796/associations	NOD2<br>TRIM62<br>PRKCD<br>CSNK2A1<br>RIPK2<br>CSNK2A1<br>MAPK8<br>VHL<br>MAPK14<br>RIPK2<br>BCL10<br>RUBCN<br>CYBA<br>YWHAB<br>RAD50	Cytoplasm	No		✓

CARHSP1	HUMAN Calcium-regulated heat-stable protein 1	CRHSP-24, CHSP1	Q9Y2V2	ENSG0000	Cancer,Cancer-Antigen	CH471112	Psoriasis, Lung carcinoma, Melanoma, Rosacea, Vulvar intraepitelial neoplasia, Atherosclerosis, Arteriosclerosis, Psoriasis vulgaris	https://www.targetvalidation.org/target/ENSG00000153048/associations	SGK2<br>SGK3<br>DYRK2<br>STYX<br>RPS6KA1<br>AKT1<br>PRKCA<br>CDK1<br>SGK1	Cytoplasm	No		✓
CASP1	HUMAN Caspase-1	IL1BC,IL1BCE	P29466	ENSG0000	Cancer	U13698	Proastate carcinoma, Digestive system disease, Liver cirrhosis, Colonic neoplasm, Non-alcoholic steatohepatitis, Pneumonia, Obesity, Lung carcinoma, Hepatitis C infection	https://www.targetvalidation.org/target/ENSG00000137752/associations	CARD8<br>NOD1<br>PYCARD<br>NLRC4<br>NOD2<br>NLRP1<br>PYDC1<br>NLRP7<br>RFWD2<br>NLRP10<br>CASP12<br>CARD17<br>CARD16<br>IL18 PAK1<br>TIRAP<br>CARD18<br>SERPINB9<br>CASP4<br>PLA2G4A<br>CASP3<br>STAT1<br>CDK11B<br>GZMB<br>TP53<br>EGFR<br>DDX58<br>RIPK2<br>MEFV<br>EGFR IL18	Cytoplasm,	Yes	https://www.bindingdb.org/uniprot/P29466	✓
CASP10	HUMAN Caspase-10	MCH4	Q92851	ENSG0000	Cancer	AF111345	Gastric carcinoma, Autoimmunelymphoproliferative syndrome, Multiple sclerosis, Liver cirrhosis, Diabetes mellitus, Neoplasm of mature B-cells, Alcoholic hepatitis	https://www.targetvalidation.org/target/ENSG00000003400/associations	MAP3K14<br>DEDD2<br>CASP8<br>RIPK1<br>FADD<br>FASLG<br>CASP3<br>FAS GZMB<br>TP53<br>CFLAR<br>TNFRSF10B<br>MAP3K14<br>BID TP53<br>CASP3<br>CASP7<br>FAS FADD<br>FASLG<br>TNFRSF10B CASP8<br>RIPK1<br>TRAF1<br>DEDD2<br>RIOK3		Yes	https://www.bindingdb.org/uniprot/Q92851	✓
CASP3	HUMAN Caspase-3	apopain, CPP32, CPP32B, Yama	P42574	ENSG0000	Signalling, Cancer	AJ413269	Nervous system disease, Genetic disorder, Brain disease, Liver diseaase, Neoplasm, Autosomal recessive non-syndromic sensorineural deafness type DFNB, Autosomal dominant non-syndromic sensorineural deafness type DFNA, Brain ischemia, Type II diabetes mellitus	https://www.targetvalidation.org/target/ENSG00000164305/associations	STK24<br>AKT3<br>RNF7<br>BIRC6 SLK<br>GORASP1<br>BIRC7<br>BAD<br>MAP4K1<br>CASP10<br>TCP10L<br>COPS6<br>MAPK14<br>PKN2<br>PKN1<br>NFE2L2<br>HSPB2<br>ELAVL1<br>PPP2R5B<br>CASP8<br>HNRNPD<br>IL16<br>BIRC2<br>BIRC3<br>ROCK1<br>STK3<br>PAK2<br>NAIP STK4<br>RAD51<br>PRKCD	Cytoplasm,	Yes	https://www.bindingdb.org/uniprot/P42574	✓
CASP4	HUMAN Caspase-4	ICE(rel)II, ICH-2, TX	P49662	ENSG0000	Cancer	U28976	Pancreatic carcinoma, Liver cirrhosis, Schizophrenia, MODY, Chronic Hepatitis C infection, Lung carcinoma, Glioblastoma multiforme, Leigh syndrome, SHORT syndrome, Glioma	https://www.targetvalidation.org/target/ENSG00000196954/associations	APP<br>CASP1<br>IL18 NOD2<br>CASP3	Cytoplasm, cytosol	Yes	https://www.bindingdb.org/uniprot/P49662	✓
CASP6	HUMAN Caspase-6	MCH2	P55212	ENSG0000	Cancer	BC000305	Liver cirrhosis, Neurodegenerative disease, Diabetes mellitus, Ishemia, Chronic Hepatitis C infection, Adenocarcinoma, Gastric neoplasm, Colorectal cancer, Multiple myeloma	https://www.targetvalidation.org/target/ENSG00000138794/associations	CASP8<br>PRKCZ<br>XIAP<br>CASP9<br>DAPK1<br>CASP3<br>TP53<br>LMNA<br>NUAK1<br>TP73<br>NEDD4<br>APP<br>CASP3<br>HIPK2<br>NUMA1<br>KRT18<br>PSEN1<br>VIM TOP1<br>LMNA<br>CASP8<br>PSEN2<br>TFAP2A<br>PCYT1A<br>LMNB1<br>SATB1<br>PPL	Cytoplasm,	Yes	https://www.bindingdb.org/uniprot/P55212	✓
CASP9	HUMAN Caspase-9	APAF-3, ICE-LAP6, MCH6, PPP1R56	P55211	ENSG0000	Signalling, Cancer	AF093130	Liver cirrhosis, Non-alcoholic steatohepatitis, Hepatitis C infection, Keloid, Fibrosis, Brain ischemia, Peripheral neuropathy, Edema, Alcoholic hepatitis	https://www.targetvalidation.org/target/ENSG00000132906/associations	CARD8<br>AKT3<br>NOD1<br>DIABLO<br>BIRC6<br>NLRP1<br>BIRC8<br>BIRC7<br>CASP12<br>PDK1<br>CASP8<br>PTCH1<br>BIRC2<br>BIRC3<br>NAIP<br>BCL2L1<br>PRKCZ<br>MAP2K1<br>CYCS<br>XIAP<br>PPP1CA<br>CASP7<br>CASP3<br>MAP2K2<br>AKT2<br>AKT1<br>MAPK1<br>MAPK3<br>CAPN2		Yes	https://www.bindingdb.org/uniprot/P55211	✓
CASS4	HUMAN Cas scaffolding protein family member 4	C20orf32, HEFL, HEPL	Q9NQ75	ENSG0000	Others	AL121914	Alzheimer's disease, Li-Fraumeni syndrome, Sepsis, Neurodegenerative disease, Multiple sclerosis, Mycoses, Lung carcinoma, Epilepsy	https://www.targetvalidation.org/target/ENSG00000087589/associations		Cytoplasm, cytoskeleton	No		✓
CBFA2T3	HUMAN Protein CBFA2T3	MTG16, MTGR2, ZMYND4	O75081	ENSG0000	Cancer	AB013286	Melanoma, Dedifferentiated chondrosarcoma, Lung carcinoma, Gastric adenocarcinoma, Prostate carcinoma, Osteosarcoma, Ductal carcinoma, Colorectal adenocarcinoma, Hepatocellular carcinoma	https://www.targetvalidation.org/target/ENSG00000129993/associations	ERBB4<br>PRKACA<br>PRKAR2A<br>ZNF652<br>NCOR1<br>TAL1<br>ZBTB33<br>PLXNA3	Nucleus, nucleolus,	No		✓

CBFB	HUMAN Core-binding factor subunit beta	PEBP2B	Q13951	ENSG0000(	Cancer	CH471092	Adenocarcinoma, Breast carcinoma, Leukemia, Myeloid neoplasm, Bile duct carcinoma, Cleft palate, Gastric neoplasm, Acute leukemia, Prostate adenocarcinoma, Squamous cell lung carcinoma	https://www.targetvalidation.org/target/ENSG00000067955/associations	RUNX2 RUNX3 RUNX1	Nucleus	Yes	https://www.bindingdb.org/uniprot/Q13951	✓
CBLC	HUMAN E3 ubiquitin-protein ligase CBL-C	CBL-3, CBL-SL, RNF57	Q9ULV8	ENSG0000(	Cancer	BC028915	Prostate carcinoma, Lung carcinoma, Metastatic prostate cancer, Myeloproliferative disorder, Breast carcinoma, Methylmalonic acidemia with homocystinuria, Myeloproliferative disease, Chronic interstitial cystitis	https://www.targetvalidation.org/target/ENSG00000142273/associations	LRIG1 ARHGEF7 CRK SRC LYN EGFR TGFB1I1 INPPL1 EGFR FYN RET GRB2 SOS2 LRIG1		No		✓
CBX5	HUMAN Chromobox protein homolog 5	HP1, HP1-ALPHA, HP1Hs-alpha	P45973	ENSG0000(	Transcription factor,Cancer,Cancer-Antigen	CH471054	Adenocarcinoma, Gorlin syndrome, Breast carcinoma, Autosomal recessive spondylocostal dysostosis, Colonic neoplasm, Colon carcinoma, Campomelic dysplasia, Aicardi syndrome, Jacobsen syndrome	https://www.targetvalidation.org/target/ENSG00000094916/associations	HDAC5 AURKB TRIM28 H3F3A; H3F3B CSNK2A1 STAT5A RPSA SMC2 E2F6 SUV39H1 TRIM24 HDAC4 INCENP HIST1H3A; HIST1H3B; HIST1H3C; HIST1H3D; HIST1H3E; HIST1H3F; HIST1H3G ; HIST1H3H; HIST1H3I; HIST1H3J HDAC9 MECP2 BCL11B ATRX	Nucleus	No		✓
CCDC110	HUMAN Coiled-coil domain-containing protein 110	CT52, KM-HN-1, CC110	Q8TBZ0	ENSG0000(	Others	AK097605	Azoospermia, Teratozoospermia, Prostate carcinoma, Breast carcinoma, Colorectal adeocarcinoma, Diffuse large B-cell lymphoma, Melanoma, Pancreatic ductal adenocarcinoma	https://www.targetvalidation.org/target/ENSG00000168491/associations		Nucleus	No		✓
CCDC183	HUMAN Coiled-coil domain-containing protein 183	KIAA1984, CC183	Q5T5S1	ENSG0000(	Others	BC064582	Sepsis	https://www.targetvalidation.org/target/ENSG00000213213/associations			No		✓
CCDC33	HUMAN Coiled-coil domain-containing protein 33	CT61, FLJ32855, CCD33	Q8N5R6	ENSG0000(	Others	AC023300	Langer mesomelic dysplasia, Hypophosphatasia, Tibial hemimelia, Acheiropodia, Orofaciodigital syndrome, Lethal chondrodysplasia (Seller type), Orofaciodigital syndrome type 4, Seckel syndrome, Fibrochondrogenesis	https://www.targetvalidation.org/target/ENSG00000140481/associations			No		✓
CCDC36	HUMAN Interactor of HORMAD1 protein 1	CT74, IHO1	Q8IYA8	ENSG0000(	Others	BC036202	Li-Fraumeni syndrome, Self-repoerted educational attainment, Azoospermia, Hepatocellular carcinoma,	https://www.targetvalidation.org/target/ENSG00000173421/associations		Chromosome	No		✓
CCM2	HUMAN Isoform 2 of Cerebral cavernous malformations 2 protein	C7orf22, MGC4607, PP10187	Q9BSQ5	ENSG0000(	Cancer,Cancer-Antigen	AC013416	Hereditary cerebralcavernous malformation, Genetic cardiac anomaly, Vascular disease, Von Willebrand disease, Colobomatous microphthalmia, Supravalvular aortic stenosis, Metthew-Wood syndrome, Medulloblastoma, Neophronophthisis	https://www.targetvalidation.org/target/ENSG00000136280/associations	DYRK4 MAP3K3 STK25 PDCD10	Cytoplasm	No		✓
CCNA1	HUMAN Cyclin-A1	CCNA1	P78396	ENSG0000(	Kinase,Cancer	AK316392	Azoospermia, Kallmann syndrome, Tongue squamous cell carcinoma, Li-Fraumeni syndrome, Acute leukemia, Primary ovarian insufficiency, Acute myeloid leukemia, Cervix carcinoma, Deafness-infertiloty syndrome, Partial chromosome Y deletion	https://www.targetvalidation.org/target/ENSG00000133101/associations	ELAVL1 CDC20 RBL2 E2F1 CDKN1B CDKN1A CDC25A CDK2 CDK1 CDKN1B E2F1 MCM3 MCM6 RB1 SKP2 CDK2 CDC20 RBL1 MCM4 CDKN1A CDK1 FZR1 MCM2 HIST1H1A CDT1 ORC2 ORC1	Nucleus	Yes	https://www.bindingdb.org/uniprot/P78396	✓

CCNA2	HUMAN Cyclin-A2	CCN1, CCNA	P20248	ENSG0000(	Kinase,Cancer	AF518006	Lung carcinoma, Large cell lung carcinoma, Melanoma, Hepatocellular carcinoma, Acute lymphoblastic leukemia, Myocardial Infarction, Fibrosis, Gastric neoplasm, Schizophernia	https://www.targetvalidation.org/target/ENSG00000145386/associations	ANAPC4 ELAVL1 SKP2 CDC20 RBL2 ZBTB16 E2F1 CDK3 CDK7 CDKN1C TSC2 CDKN1B CDKN1A CDK2 TAF1 SP1 CDK1 RB1 E2F3 E2F1 TP53 FEN1 CDC6 RB1 KAT2B SKP2 CDK4 CDK2 E2F3 RBL1 TP73 CDK1 FZR1	Nucleus		Yes	https://www.bindingdb.org/uniprot/P20248	✓
CCNB1	HUMAN G2/mitotic-specific cyclin-B1	CCNB	P14635	ENSG0000(	Kinase,Signalling,Cancer	BT020128	Hepatocellular carcinoma, Lung carcinoma, Melanoma, Large cell lung carcinoma, Psoriasis, Polycystic ovary syndrome, Renal cell carcinoma, Colorectal adenocarcinoma	https://www.targetvalidation.org/target/ENSG00000134057/associations	FZR1 ANAPC4 NLK PLK3 PKMYT1 PBK AURKB TSC1 MAPK15 MAPK6 E2F4 ELAVL1 AGER E2F2 PTCH1 PIN1 MAPK7 CDC20 FOXM1 E2F1 CDK5 MAPK10 PLK1 CDK7 TSC2 MAPK9 MAPK8 CDKN1A TGFBR2 MAPK4	Cytoplasm, Nucleus, cytoskeleton, microtubule organizing center, centrosome,		Yes	https://www.bindingdb.org/uniprot/P14635	✓
CCNB1IP1	HUMAN E3 ubiquitin-protein ligase CCNB1IP1	C14orf18, HEI10	Q9NPC3	ENSG0000(	Cancer	BX161423	Melanoma, Lung carcinoma, Bile duct carcinoma, Gastric adenocarcinoma, Ovarian carcinoma, Head and Neck squamous cell carcinoma, Hepatocellular carcinoma, Rectal adenocarcinoma, Esophageal squamous cell carcinoma, Oral cavity cancer	https://www.targetvalidation.org/target/ENSG00000100814/associations	NF2	Nucleus, Chromosome,		No		✓
CCND1	HUMAN G1/S-specific cyclin-D1	BCL1, D11S287E, PRAD1, U21B31	P24385	ENSG0000(	Kinase,Cancer	M73554	Lung carcinoma, Breast carcinoma, Lymphoma , Neoplasma of mature B -cells, Plasma cell neoplasm, Multiple myeloma, AL amyloidosis, Colorectal adenocarcinoma, Primary systemic amyloidosis, Diffuse gastric adenocarcinoma	https://www.targetvalidation.org/target/ENSG00000110092/associations	NCOA3 DYRK1B BTRC EHMT1 TESK2 HDAC2 CDKN2A FBXW8 FBXO31 MAPK14 ELAVL1 HNRNPD DYRK1A CUL1 HDAC1 EP300 CDK6 BTG2 GTF2I SKP1 RBX1 PRKX GSK3B GSK3A CDKN1B CDKN2A STAT3 CDKN1A BRCA1	Nucleus		Yes	https://www.bindingdb.org/uniprot/P24385	✓
CCND2	HUMAN G1/S-specific cyclin-D2	CCND2	P30279	ENSG0000(	Kinase,Signalling,Cancer	BT019847	Lung carcinoma, Prostate carcinoma, Megalencephaly-polymicrogyria-postaxial polydactyly-hydrocephalus, Adenocarcinoma, Esophageal adenocarcinoma, Type II diabetes mellitus, Polymicrogyria, Telecanthus, Acute myelocytic leukemia, Cutaneous melanoma	https://www.targetvalidation.org/target/ENSG00000118971/associations	CDKN2A MAPK14 CDK5 CDK6 GSK3B CDKN1B CDKN1A CDK4 MYC CDKN1B CDK6 RB1 CDK4 CDK2 CDKN1A PCGF2	Nucleus		No		✓
CCNH	HUMAN Cyclin-H	CycH, p34, p37	P51946	ENSG0000(	Kinase,Signalling,Cancer	AF477979	HIV infection,Teratozoospermia, Schizophrenia, Ishemia, Unipolar depression, Glioma, AIDS, Esophageal squamous cell carcinoma	https://www.targetvalidation.org/target/ENSG00000134480/associations	MTA1 CSNK2A1 MNAT1 CDK7 CDK8 GTF2H1 CDK2 POLR2A CSNK2A2 ERCC3 ERCC2 RARA AR DAPK3 CDK7 POLR2A CDK2 RARA MNAT1 ERCC2 ERCC3 GTF2H1 GTF2E2	Nucleus		Yes	https://www.bindingdb.org/uniprot/P51946	✓
CCNI	HUMAN Cyclin-I	CCNI1	Q14094	ENSG0000(	Kinase,Cancer,Cancer-Antigen	AY207372	IGA Glomerulonephritis, Frasier syndrome, Atypical hemolytic-uremic syndrome, Lipoprotein glomerulopathy, Familial idiopathic steroid-resistant nephrotic syndrome, Dent disease type 1, Primary hyperoxaluria	https://www.targetvalidation.org/target/ENSG00000118816/associations				No		✓
CCR5	HUMAN C-C chemokine receptor type 5	CC-CKR-5, CD195, CKR-5, CKR5, CMKBR5, IDDM22	P51681	ENSG0000(	Others	U83326	HIV infection, AIDS, Vascular disease, Diabetes mellitus, Autoimmune disease, Endothelial dysfunction, Orchitis, Nephritis, West Nile fever, Schizophrenia	https://www.targetvalidation.org/target/ENSG00000160791/associations	CCL26 MKNK2 CCL13 CCL14 GRK1 PTK2B PTK2 CCL7 CCL8 CCL11 DNM2 GRK6 TXK CCR2 GRK3 GRK5 GRK4 PSMA5 GRK2 JAK1 PRKCA CCL3L1; CCL3L3 CCL5 CCL2 CCL4 CCL3 LCK AFP ORM1 CD4 JAK2 CCL16	Cell membrane	Transmembrane	Yes	https://www.bindingdb.org/uniprot/P51681	✓

CCT3	HUMAN T-complex protein 1 subunit gamma	TCPG, Cctg, TRIC5	P49368	ENSG0000(	Cancer,Cancer-Antigen	BC006501	Liver cirrhosis, Hepatocellular carcinoma, Ovarian carcinoma, Myotonic dystrophy, Breast carcinoma, Liver cirrhosis, Muscular dystrophy	https://www.targetvalidation.org/target/ENSG00000163468/associations	TBK1 MAP3K3 MAP3K1 STAT3 ISG15 RAF1	Cytoplasm,		Yes	https://www.drugbank.ca/biodb/polypeptides/P49368	✓
CCT5	HUMAN T-complex protein 1 subunit epsilon	CCTE, TCPE, KIAA0098	P48643	ENSG0000(	Cancer,Cancer-Antigen	AK302383	Hereditary sensory and autonomic neuropathy with spastic paralegia, Breast carcinoma, Melanoma, Psoriasis, Rheumatoid arthritis, Clonus, Spastic paraplegia, Atrophic spinal cord, Epilepsy, Glioblastoma multiforme	https://www.targetvalidation.org/target/ENSG00000150753/associations	RIPK3 TBK1 MAP3K3 IKBKE MAP3K1 IL7R THEG	Cytoplasm		No		✓
CD276	HUMAN CD276 antigen	B7-H3, B7H3, B7RP-2	Q5ZPR3	ENSG0000(	Others	AK074849	Hepatocellular carcinoma, Asthma, Pancreatic carcinoma, Serum Gamma-glutamyl transferase measurement, Lung disease, Rheumatoid arthritis, Systemic lupus erythematosus, Neuroblastoma, Breast carcinoma	https://www.targetvalidation.org/target/ENSG00000103855/associations		Membrane	Transmembrane	No		✓
CD5L	HUMAN CD5 antigen-like	API6, Spalpha	O43866	ENSG0000(	Others	AK29040	Hepatocellular carcinoma, Chronic granulomatous disease, Isolated agammaglobulinemia, Common variable immunodeficiency, Sanjad-Sakati syndrome, Obesity, Conn syndrome, Gitelman syndrome, Dyskeratosis congenita, Pachyonychia congenita	https://www.targetvalidation.org/target/ENSG00000073754/associations	CD40LG	Secreted		No		✓
CD96	HUMAN T-cell surface protein tactile	TACTILE,TACT	P40200	ENSG0000(	Others	NA	C syndrome, Melanoma, Ovarian cancer, Acute myeloid leukemia, Breast carcinoma, Lung carcinoma, Rheumatoid arthritis, Sjogren's syndrome	https://www.targetvalidation.org/target/ENSG00000153283/associations	PVR	Membrane, Single-pass type I membrane protein,	Transmembrane	No		✓
CDC20	HUMAN Cell division cycle protein 20 homolog	CDC20A, p55CDC	Q12834	ENSG0000(	Signalling, Cancer	BT007388	Lung carcinoma, Large cell lung carcinoma, Melanoma, Pancreatitis, Asparaginase-induced acute pancreatitis, Acute lymphoblastic leukemia, Lymphoma, Glioblastoma, T-cell leukemia	https://www.targetvalidation.org/target/ENSG00000117399/associations	AXIN2 ANAPC10 FBXO5 ANAPC2 ANAPC4 ANAPC5 ANAPC7 CDC23 ANAPC11 RASSF1 ANAPC1 CDC26 FBXO43 MAD2L1 CDC16 E2F1 CCNA1 PLK1 NEK2 TGFBR1 CDC27 CCNB1 CDH1 TP53 BUB1B BUB3 BUB1 AURKA UBE2C	Cytoplasm, cytoskeleton, microtubule organizing center, centrosome		No		✓
CDC25A	HUMAN M-phase inducer phosphatase 1	MPIP1	P30304	ENSG0000(	Signalling, Cancer	BC007401	Alzheimer's disease, Melanoma, Breast carcinoma, Hepatocellular carcinoma, Psoriasis, Large cell lung carcinoma, Squamous cell carcinoma, Azoospermia, Glioma, Glioblastoma multiforme	https://www.targetvalidation.org/target/ENSG00000164045/associations	BTRC CDC14A FBXW11 PLK3 MAP3K5 NEK11 USP17L2 MAPK14 RPS6KA1 CUL1 ROCK1 FOXM1 SMAD3 SKP1 GSK3B CSNK1E MAPKAPK 2 CSNK1A1 TGFBR2 MAPK3 CDK2 CCNE1 PRKCA CCNB1 PIM1 AR UBB CDK1 RAF1 MYC			Yes	https://www.bindingdb.org/uniprot/P30304	✓
CDC25C	HUMAN M-phase inducer phosphatase 3	MPIP3	P30307	ENSG0000(	Signalling, Cancer	CH471062	Alzheimer's disease, Schizophrenia, Hepatocellular carcinoma, Breast carcinoma, Acute lymphoblastic leukemia, Azoospermia, Prostate carcinoma, Bladder neoplasm, Central nervous system cancer, Vulvar carcinoma	https://www.targetvalidation.org/target/ENSG00000158402/associations	EGFR PLK3 BRSK1 BRSK2 MAPK14 PKN1 RPS6KA1 PIN1 FOXM1 YWHAH CSNK2A1 YWHAZ YWHAE PPP1CB PPP1CA MAPK10 PLK1 MAPKAPK 2 MAPK9 MAPK8 PPP1CC YWHAB MARK3 YWHAQ CDK2 CCNB1 PCNA CDK1 LCK TP53 CHEK2 MAP3K4	Nucleus		Yes	https://www.bindingdb.org/uniprot/P30307	✓
CDC42	HUMAN Cell division control protein 42 homolog	CDC42Hs, G25K	P60953	ENSG0000(	Signalling	AL121735	Glioma, Astrocytoma, Ependymoma, Lamellar ichthyosis, Angioma serpiginosum, Psoriasis, Alopecia, Hypotrichosis simplex, Glioblastoma multiforme, Schizophrenia	https://www.targetvalidation.org/target/ENSG00000070831/associations	PAK5 CDC42SE 1 PAK6 PARD6A ECT2 DEF6 RACGAP1 DOCK9 PARD6B PARD6G ARHGDIG DOCK7 MARK4 NGEF CDC42BP G MAP3K11 ITSN1 RALBP1 ARHGEF6 ARHGEF7 BIRC2 PAK2 PAK1 BNIP2 ARHGAP1 TNK2 PRKCZ	Cell membrane Cytoplasm, cytoskeleton, microtubule organizing center, centrosome, spindle, Midbody,		Yes	https://www.bindingdb.org/uniprot/P60953	✓

CDH19	HUMAN Cadherin-19	CAD19, CDH7L2, UNQ478, PRO941	Q9H159	ENSG0000(	Others	AC090393	Hepatocellular carcinoma, Colorectal adenocarcinoma, Lung adenocarcinoma, Alzheimer's disease, Melanoma, Head and Neck cancer, Rosacea, Psoriasis, Cervical carcinoma	https://www.targetvalidation.org/target/ENSG00000071991/associations		Cell membrane, Single-pass type I membrane protein,	Transmembrane	No		✓
CDK1	HUMAN Cyclin-dependent kinase 1	CDC2, CDC28A, CDKN1, P34CDC2	P06493	ENSG0000(	Kinase,Cancer	AK291939	Neoplasm of mature B-cells, Chronic lymphocytic leukemia, Lymphoid neoplasm, Cacaine dependence, Melanoma, Alzheimer's disease, Glioma, Lung carcinoma, Glioma, Central nervous system cancer	https://www.targetvalidation.org/target/ENSG00000170312/associations	ANAPC10<br>FZR1<br>FBXO5<br>ANAPC4<br>CDC23<br>ANAPC1<br>PKMYT1<br>CDK5RAP3 TSC1<br>E2F4<br>PTCH1<br>PIN1<br>CDC16<br>RBL2<br>DDR1<br>FOXM1<br>MYT1<br>E2F1<br>CCNA1<br>EIF4G2<br>CSNK2A1<br>PPP2CA<br>PPP2CB<br>CKS1B<br>BLM CDK7<br>GSK3B<br>TSC2<br>CDKN1B<br>MAP3K8	Nucleus, Cytoplasm, Mitochondrion, microtubule organizing center, centrosome, spindle,		Yes	https://www.bindingdb.org/uniprot/P06493	✓
CDK10	HUMAN Cyclin-dependent kinase 10	PISSLRE	Q15131	ENSG0000(	Kinase	AM392903	Melanoma, Lymphoma, Chronic lymphocytic leukemia, Small cell lung carcinoma, Mantle cell lymphoma, Keloid, Atopic eczema, Breast neoplasm, Ovarian carcinoma	https://www.targetvalidation.org/target/ENSG00000185324/associations	PIN1<br>STMN1<br>SRC SYN1<br>PTPN1<br>CSNK2A1<br>KIF11<br>TP53<br>MYOD1<br>MAP2K1<br>PPP1R1B<br>RB1 RBL2<br>PSEN1<br>PPP1CA<br>PAK1<br>LMNA<br>FZR1<br>ESR1<br>MAPT<br>CSNK2B<br>AMPH<br>GRIN2A<br>ETS2<br>RAB4A<br>CDK16<br>DAB1<br>LMNB1<br>NES<br>C1orf61			No		✓
CDK11B	HUMAN Cyclin-dependent kinase 11B	CDC2L1, CDK11-p110, CDK11-p58	P21127	ENSG0000(	Kinase	U04816	Lymphoma, Mantle cell lymphoma, Non-Hodgkin's lymphoma, Chronic lymphocytic leukemia, Small cell lung carcinoma, Neuroblastoma, Osteosarcoma, Melanoma, Breast carcinoma	https://www.targetvalidation.org/target/ENSG00000248333/associations	CDK11A<br>RANBP9<br>CDK7<br>CCND3<br>SRC<br>CHEK2<br>YWHAG<br>CASP1<br>SRC<br>CASP3<br>YWHAQ<br>EIF3F<br>TCEA2<br>PAK1<br>ESR1<br>CASP8<br>CCND3<br>YWHAE<br>YWHAB<br>CCNL2<br>URM1	Cytoplasm, Nucleus,		Yes	https://www.bindingdb.org/uniprot/P21127	✓
CDK14	HUMAN Cyclin-dependent kinase 14	PFTAIRE1, PFTK1	O94921	ENSG0000(	Kinase	AK295086	Breast carcinoma, Ovarian carcinoma, Esophageal squamous cell carcinoma, Colonic neoplasm, Small cell lung carcinoma, Mantle cell lymphoma, Glioma, Gastric carcinoma, Hepatocellular carcinoma, Osteosarcoma	https://www.targetvalidation.org/target/ENSG00000058091/associations	CCNY<br>LRP6<br>TGFBR1<br>TAGLN2<br>SMURF1<br>ACVR1<br>BMPR1B<br>PPP1R2<br>CCNY<br>SEPT8	Cell membrane, Peripheral membrane protein, Cytoplasm, Nucleus,		Yes	https://www.bindingdb.org/uniprot/O94921	✓
CDK16	HUMAN Cyclin-dependent kinase 16	PCTAIRE, PCTAIRE1, PCTK1	Q00536	ENSG0000(	Kinase	AK301832	Lymphoma, Male infertility with teratozoospermia due to single gene mutation, Schizophrenia, Peutz-Jeghers syndrome, Tuberculosis, Herpes simplex infection, Myotonic dystrophy, B-Cell lymphoma, Anaplastic thyroid carcinoma, Primary ciliary dyskinesia	https://www.targetvalidation.org/target/ENSG00000102225/associations	CDK12<br>CDK19<br>CDK20<br>CDK10<br>CDK13<br>ATM<br>CDK18<br>CDK17<br>CDK5<br>CDK6<br>CDK3<br>PRKX<br>CDK9<br>CDK7<br>CDK8<br>CDK2<br>PRKACA<br>CDK4<br>ARAF<br>CDK1<br>PRKY<br>YWHAG<br>STMN1<br>SRC SYN1<br>PTPN1<br>CSNK2A1<br>KIF11<br>TP53<br>MYOD1	Cytoplasm, Cytoplasmic vesicle, secretory vesicle, Cell membrane, Peripheral membrane protein, Cytoplasmic side, Cell junction, synapse, synaptosome		Yes	https://www.bindingdb.org/uniprot/Q00536	✓
CDK17	HUMAN Cyclin-dependent kinase 17	PCTAIRE2, PCTK2	Q00537	ENSG0000(	Kinase	AC125612	Venous thromboembolis, Lymphoma, Small cell lung carcinoma, Chronic lymphocytic leukemia, Neurofibromatosis 1, Mantle cell lymphoma, Glioblastoma multiforme, Dermamyositis, Multiple sclerosis, Gaucher disease type 3	https://www.targetvalidation.org/target/ENSG00000059758/associations	YWHAG<br>STMN1<br>SRC SYN1<br>PTPN1<br>CSNK2A1<br>KIF11<br>TP53<br>MYOD1<br>MAP2K1<br>PPP1R1B<br>RB1 RBL2<br>PSEN1<br>PPP1CA<br>PAK1<br>LMNA<br>FZR1<br>ESR1<br>MAPT<br>CSNK2B<br>TGFBR1<br>AMPH<br>GRIN2A<br>RAB4A<br>CDK16<br>DAB1<br>LMNB1<br>NES<br>TDRD7<br>C1orf61			Yes	https://www.bindingdb.org/uniprot/Q00537	✓
CDK18	HUMAN Isoform 2 of Cyclin-dependent kinase 18	PCTAIRE3, PCTK3	Q07002	ENSG0000(	Kinase	AL357131	Breast carcinoma, Lymphoma, Alzheimer's disease, Glioma, Mantle cell lymphoma, Melanoma, Pituitary cancer, Parkinson's disease, Chronic lymphocytic leukemia, Prostate carcinoma	https://www.targetvalidation.org/target/ENSG00000117266/associations	STMN1<br>MDFI SRC SYN1<br>PTPN1<br>TRIP10<br>CSNK2A1<br>KIF11<br>TP53<br>MYOD1<br>MAP2K1<br>PPP1R1B<br>RB1 RBL2<br>PSEN1<br>CCNK<br>PPP1CA<br>PAK1<br>LMNA<br>FZR1<br>ESR1<br>MAPT<br>CSNK2B<br>ZYX AMPH<br>GRIN2A<br>SEC23A<br>RAB4A<br>CDK16<br>DAB1<br>LMNB1<br>NES			Yes	https://www.bindingdb.org/uniprot/Q07002	✓

CDK19	HUMAN Cyclin-dependent kinase 19	CDC2L6, CDK11, KIAA1028	Q9BWU1	ENSG0000(	Kinase	AL512430, AL603914, Z84480	Lymhoma, Leukemia, Mantle cell lymphoma, Lung carcinoma, Breast carcinoma	https://www.targetvalidation.org/target/ENSG00000155111/associations	PTEN STMN1 SRC SYN1 PTPN1 CSNK2A1 KIF11 TP53 MYOD1 MAP2K1 PPP1R1B EIF3F RB1 RBL2 PSEN1 PPP1CA PAK1 LMNA FZR1 ESR1 MAPT CSNK2B PIK3CD AMPH GRIN2A RAB4A SRSF7 CDK16 DAB1 LMNB1 NES		Yes	https://www.bindingdb.org/uniprot/Q9BWU1	✓
CDK2	HUMAN Cyclin-dependent kinase 2	CDK2	P24941	ENSG0000(	Kinase,Cancer	AB012305	Chronic lymphocytic leukemia, Lymphoid neoplasm, Neoplasm of mature B-cells, Leukemia, Breast carcinoma, Medulloblastoma, Retinoblastoma, Glioma	https://www.targetvalidation.org/target/ENSG00000123374/associations	IKBKG FZR1 KAT2B ATRIP CDK20 SPDYA CDKN3 CUL1 SKP2 PRKAA1 PTP4A2 DDR1 UBXN1 CDK6 CCNA1 PPP2CA SKP1 PPP2CB CKS1B PSMA6 CCNH CDK9 CDK7 CDKN1C CDKN1B CDKN2A CDKN1A PPM1A AKT1	Cytoplasm, cytoskeleton, microtubule organizing center, centrosome, Nucleus, Cajal body, Cytoplasm, Endosome,	Yes	https://www.bindingdb.org/uniprot/P24941	✓
CDK20	HUMAN Cyclin-dependent kinase 20	CCRK, CDCH	Q8IZL9	ENSG0000(	Kinase	AF547664	Lymphoma, Chronic lymphocytic leukemia, Glioma, Mantle cell lymphoa, Glioblastoma multiforme, Spinocerebellar ataxia, Small cell lung carcinoma	https://www.targetvalidation.org/target/ENSG00000156345/associations	CDK2 STMN1 SRC SYN1 PTPN1 CSNK2A1 KIF11 TP53 MYOD1 MAP2K1 PPP1R1B RB1 RBL2 PSEN1 CDK2 PPP1CA PAK1 LMNA FZR1 ESR1 MAPT CSNK2B AMPH GRIN2A RAB4A ICK CDK16 DAB1 LMNB1 NES C1orf61	Nucleus	No		✓
CDK3	HUMAN Cyclin-dependent kinase 3	CDKN3	Q00526	ENSG0000(	Kinase	AY789470	Neoplasm, cancer, lymphoma, lung carcinoma, Mantle cell lymphoma, chronic lymphocytic leukemia	https://www.targetvalidation.org/target/ENSG00000250506/associations	CDKN1A STMN1 CTTN SRC SYN1 PTPN1 CDKN1B E2F1 CSNK2A1 LIG1 KIF11 TP53 CCNE1 MYOD1 MAP2K1 PPP1R1B JUN CCNA2 RB1 RBL2 PSEN1 E2F3 CDKN1A PPP1CA CKS1B CHEK1 PAK1 LMNA ATF1 FZR1 ESR1 MAPT		Yes	https://www.bindingdb.org/uniprot/Q00526	✓
CDK4	HUMAN Cyclin-dependent kinase 4	PSK-J3	P11802	ENSG0000(	Kinase,Cancer	U37022	Familial melanoma, Breast carcinoma, Lung carcinoma, Squamous cell carcinoma, Melanoma, Cutaneous melanoma, Prostate carcinoma, Pancreatic ductal adenocarcinoma, Bladder carcinoma, Colorectal adenocarcinoma	https://www.targetvalidation.org/target/ENSG00000135446/associations	SERTAD1 CDKN2A NFATC3 CDKN2D CDK7 CDKN1C CDKN1B CDKN2C CDKN2B CDKN2A CDKN1A BRCA1 CDC25A CCND3 CCND2 CCND1 CCNA2 RPA2 ETS1 SRC PCNA LYN YES1 MYC STMN1 CTTN LYN SRC SYN1 PTPN1 CDKN1B IRF3 MCM7 BIRC5	Cytoplasm, Nucleus, Membrane,	Yes	https://www.bindingdb.org/uniprot/P11802	✓
CDK5	HUMAN Cyclin-dependent-like kinase 5	PSSALRE	Q00535	ENSG0000(	Kinase	BT006680	Neuroegenerative disease, Leukemia, Tauopathy, Lissencephaly with cerebellar hypoplasia, Alzheimer's disease, Chronic lymphocytic leukemia, Arthrogryposis, Agyria, Hirsutism	https://www.targetvalidation.org/target/ENSG00000164885/associations	CSNK1G3 PPP1R1B CSNK1G1 VRK1 CSNK1A1L VRK3 VRK2 TTBK2 TTBK1 STX1A CDK5R1 CDK5R2 CSNK1G2 CSNK1E CSNK1D CSNK1A1 FYN ABL1 MAP2K7 STX3 DNM1 TRPV1 ACTN1 DBN1 DPYSL2 STMN1 CTNNB1 NOS3 CTTN DRD2 SRC	Cytoplasm, Cell membrane, Peripheral membrane protein, Perikaryon, Cell projection, lamellipodium	Yes	https://www.bindingdb.org/uniprot/Q00535	✓
CDK5R1	HUMAN Cyclin-dependent kinase 5 activator 1	Nck5a, p35nck5a, CD5R1, CDK5R	Q15078	ENSG0000(	Kinase	CH471147	Alzheimer's disease, Glioma, Chudley-McCullough syndrome, Astrocytoma, Psoriasis, Bilateral frontal polymicrogyria, Schizophrenia, Ganglioglioma	https://www.targetvalidation.org/target/ENSG00000176749/associations	CAMK2A SIK2 LMTK2 AATK TTBK1 AAK1 PAK1 CDK16 CDK5 PPP2CA PPP2CB STXBP1 CDK9 CTNNB1 CAPN3 CAPN2 CHN1 CAPN1 CDK5 PAK1 LMTK2 SUDS3 ODF1	Cyclin-dependent kinase 5 activator 1, p35, Cell membrane	Yes	https://www.bindingdb.org/uniprot/Q15078	✓
CDK7	HUMAN Cyclin-dependent kinase 7	CAK, CAK1, CDKN7, MO15, STK1	P50613	ENSG0000(	Kinase,Cancer	X77303	HIV infection, Lymphoma, Leukemia, Prostate carcinoma, Cystic fibrosis, Small cell lung carcinoma, Mantle cell lymphoma, Chronic lymphoblastic leukemia	https://www.targetvalidation.org/target/ENSG00000134058/associations	NEK6 GTF2H4 GTF2H3 GTF2H2 MNAT1 CCNH ABL2 PRKCI GTF2H1 ERCC5 CDK2 POLR2A ERCC3 ERCC2 RPA2 CDK1 STMN1 CTTN SRC SYN1 PTPN1 MCM7 E2F1 CSNK2A1 NEUROD1 KIF11 SMAD4 BRCA1 TP53 CCNH	Nucleus, Cytoplasm, Cytoplasm, perinuclear region,	Yes	https://www.bindingdb.org/uniprot/P50613	✓

CDK8	HUMAN Cyclin-dependent kinase 8	K35	P49336	ENSG0000(	Kinase	CH471075	Pituitary gland adenoma, Sarcoma, Leukemia, Breast carcinoma, Type II diabetes mellitus, Lymphoma, Ductal carcinoma, Colorectal cancer, Melanoma, Polyploidy	https://www.targetvalidation.org/target/ENSG00000132964/associations	CREBBP MAML1 STMN1 SRC SYN1 PTPN1 E2F1 CSNK2A1 KIF11 TP53 CCNH MYOD1 MAP2K1 SMAD3 PPP1R1B RB1 SMAD1 MAML1 RBL2 NOTCH1 PSEN1 POLR2A PPP1CA PAK1 LMNA FZR1 ESR1 MAPT CSNK2B AMPH	Nucleus		Yes		✓
													https://www.bindingdb.org/uniprot/P49336	
CDK9	HUMAN Cyclin-dependent kinase 9	C-2k, CDC2L4, PITALRE, TAK	P50750	ENSG0000(	Kinase	BT019903	Neoplasm of mature B-cells, Leukemia, Chronic lymphocytic leukemia, HIV infection, Prostate carcinoma, Neuroblastoma, Liver carcinoma, Mantle cell lymphoma, Melanoma, Cystic fibrosis	https://www.targetvalidation.org/target/ENSG00000136807/associations	CTDP1 AURKB KAT2B CAMK1D EP300 PRKCD PPP2CA PPP2CB PPP1CA PPP1CC PPM1A GTF2F1 AKT1 CDK2 POLR2A AR PPM1B BRD4 CCNT2 CCNT1 CUL1 STMN1 SRC SYN1 PTPN1 APC CSNK2A1 NEUROD1 KIF11 BORA MYBL2	Nucleus, Cytoplasm, Nucleus, PML body,		Yes		✓
													https://www.bindingdb.org/uniprot/P50750	
CDKN1A	HUMAN Cyclin-dependent kinase inhibitor 1	CAP20, CDKN1, CIP1, p21CIP1, p21Cip1/Waf1, SDI1, WAF1, CDN1A, MDA6, PIC1	P38936	ENSG0000(	Kinase,Signalling,Cancer	U03106	Heart function measurement, QRS duration, Epithelial neoplasm, Alopecia, Autoimmune disease, Colorectal cancer, Colonic neoplasm, Dyskeratosis congenita, Melanoma, Head and Neck squamous cell carcinoma	https://www.targetvalidation.org/target/ENSG00000124762/associations	DYRK1B ING1 NLK BCCIP ZBTB4 PIM2 DTL TSG101 MAP3K5 EP400 DDB2 MAPK15 GFI1B MAPK6 MAPK14 ELAVL1 PDK1 CUL4A CUL1 NEUROD1 HDAC1 ATR SMAD4 SKP2 TRIM28 MAPK7 ZBTB17 FOXO1 EP300 E2F1 MDM2	Cytoplasm, Nucleus,		Yes		✓
													https://www.bindingdb.org/uniprot/P38936	
CDKN2A	HUMAN Cyclin-dependent kinase inhibitor 2A	ARF, CDK4I, CDKN2, CMM2, INK4, INK4a, MLM, MTS1, p14ARF, p16, p16INK4a, p19Arf	P42771	ENSG0000(	Kinase,Cancer	AF527803	Sarcoma, Head and Neck neoplasia, Pancreatic neoplasm, Lung carcinoma, Familial melanoma, Glioma, Squamous cell carcinoma, Renal cell carcinoma, Melanoma, Lymphoid neoplasm	https://www.targetvalidation.org/target/ENSG00000147889/associations	AURKB E2F2 ATR ZBTB17 E2F1 MDM2 CDK6 PSME3 MAPK10 DAPK1 MAPK8 BMI1 PSMC3 CDK4 NPM1 TP53 MYC IKBKB E2F3 CDC7 E2F1 TP53 CDK6 AURKB CDK4 HUWE1 CDK2 E2F3 ATR MDM2 BCL2L1 CCND1 MYC KAT5	Cytoplasm		No		✓
CDKN2B	HUMAN Cyclin-dependent kinase 4 inhibitor B	CDK4I, INK4B, MTS2, p15INK4b, CDN2B	P42772	ENSG0000(	Kinase,Signalling	CR536529	Brain aneurysm, Central nervous system disorder, Glaucoma, Myocardial infarction, Diabetes mellitus, Glioma, Hereditary melanoma, Coronary artery disease, Multiple endocrine neoplasia type 1, Aortic aneurysm	https://www.targetvalidation.org/target/ENSG00000147883/associations	ZBTB17 CDK6 CDK8 CDK4 PIAS2 CDK6 CDK4	Cytoplasm		No		✓
CDKN2C	HUMAN Cyclin-dependent kinase 4 inhibitor C	INK4C, CDN2C, CDKN6	P42773	ENSG0000(	Kinase,Signalling,Cancer	AY094608	Glioma, Melanoma, Multiple myeloma, Brain stem glioblastoma, Kidney neoplasm, Acromegaly, Parathyroid hyperplasia, Colorectal adenocarcinoma, Breast carcinoma, Astrocytoma	https://www.targetvalidation.org/target/ENSG00000123080/associations	KMT2A CDK6 CDK4 TP53 CDK6 CDK4			No		✓
CDKN2D	HUMAN Cyclin-dependent kinase 4 inhibitor D	INK4D, CDN2D	P55273	ENSG0000(	Kinase,Signalling	AF061327	Kallmann syndrome, Glioma, Astrocytoma, Neuroblastoma, Autosomal dominant non-syndromic sensorineural deafness type DFNA, Autosomal recessive non-syndromic sensorineural deafness type DFNB, Xerosis, Melanoma, Melanocytic nevus, Lung carcinoma	https://www.targetvalidation.org/target/ENSG00000129355/associations	CDK6 MAP2K6 CDKN1A CDK2 PRKACA PRKCA CDK4 CDK6 CDK4	Nucleus		No		✓
CDO1	HUMAN Cysteine dioxygenase type 1	CDO1	Q16878	ENSG0000(	Others	D85777	Li-Fraumeni syndrome, Colorectal cancer, Lung carcinoma, Campomelic dysplasia, Acrodysostosis, Sezary syndrome, Mycosis fungoides, Pfeiffer syndrome	https://www.targetvalidation.org/target/ENSG00000129596/associations	SHH			Yes		✓
													https://www.drugbank.ca/biodb/polypeptides/Q16878	
CEACAM1	HUMAN Carcinoembryonic antigen-related cell adhesion molecule 1	BGP, BGP1, CD66a, CEAM1	P13688	ENSG0000(	Others	X16356	Squamous cell carcinoma, Melanoma, Prostate carcinoma, Esophageal squamous cell carcinoma, Lung carcinoma, Septic shock, Adenocarcinoma, Bladder neoplasm, Autoimmune disease, Colonic neoplasm	https://www.targetvalidation.org/target/ENSG00000079385/associations	CAMK2D PTPN11 MAP3K10 BLK PXN SHC1 PTPN6 FLNA SRC ANXA2 INSR ITGB3 EGFR PXN SHC1 PTPN6 HAVCR2 CEACAM8	Cell membrane	Transmembrane	Yes		✓
													https://www.drugbank.ca/biodb/polypeptides/P13688	

CEACAM7	HUMAN Carcinoembryonic antigen-related cell adhesion molecule 7	CGM2, CEAM7	Q14002	ENSG0000(	Kinase	AF006623	Esophageal squamous cell carcinoma, Lung carcinoma, Colorectal cancer, Colonic neoplasm, Lung adenocarcinoma, Breast carcinoma, Crohn's disease, Psoriasis, Rosacea, Pancreatic ductal adenocarcinoma	https://www.targetvalidation.org/target/ENSG00000007306/associations		Cell membrane		No		✓
CEACAM8	HUMAN Carcinoembryonic antigen-related cell adhesion molecule 8	CD66b, CGM6, CEAM8	P31997	ENSG0000(	Others	AC004558	Osteosarcoma, MALT lymphoma, Septic shock, Sepsis, Lung carcinoma, Meningococcal infection, Aplastic anemia, Paroxysmal nocturnal hemoglobinuria, Tuberculosis, Chronic myelogenous leukemia	https://www.targetvalidation.org/target/ENSG00000124469/associations	CEACAM1 ITGAM CEACAM6	Cell membrane, Lipid-anchor, GPI-anchor,		No		✓
CEBPG	HUMAN CCAAT/enhancer-binding protein gamma	GPE1BP, IG/EBP-1	P53567	ENSG0000(	Transcription factor,Cancer,Cancer-Antigen	BT019820	Breast carcinoma, Melanoma, Lung carcinoma, Famililal hemophagocytic lymphohistiocytosis, Wiskott-Aldrich syndrome, Bronchogenic carcinoma, Acute myelocytic leukemia, Lymphoblastic leukemia, Glioblastoma multiforme, X-linked lymphoproliferative disease, Central nervous system cancer	https://www.targetvalidation.org/target/ENSG00000153879/associations	CEBPE FOS ATF4 DDIT3	Nucleus		No		✓
CEP55	HUMAN Centrosomal protein of 55 kDa	C10orf3, CT111, FLJ10540	Q53EZ4	ENSG0000(	Others	AK315536	Glioma, Central nervous system cancer, Lung carcinoma, Melanoma, Colonic neoplasm, Medulloblastoma, Acute lymphoblastic leukemia, Adenocarcinoma, Pancreatic carcinoma, Polycystic ovary syndrome	https://www.targetvalidation.org/target/ENSG00000138180/associations	PLK1 MAPK1 CDK1	Cytoplasm, cytoskeleton, microtubule organizing center, centrosome, centriole		No		✓
CERS2	HUMAN Ceramide synthase 2	LASS2, SP260, TMSG1	Q96G23	ENSG0000(	Transcription factor	CH471121	Rhegmatogenous retinal detachment, Chronic kidney failure, Prostate carcinoma, Breast carcinoma, Bladder carcinoma, Urate measurement, Hepatocellular carcinoma, Progressive myoclonic epilepsy, Serum creatinine measurement, Glomerular filtration rate	https://www.targetvalidation.org/target/ENSG00000143418/associations		Nucleus membrane	Transmembrane	No		✓
CFL1	HUMAN Cofilin-1	CFL, COF1	P23528	ENSG0000(	Cancer,Cancer-Antigen	BT006846	Isolated spina bifida, Breast carcinoma, Gastric carcinoma, Keratoconus, Asthma, Neuralgia, Neural tube cloasure defect, Keratoconus, Mammary neoplasm, Spina bifida	https://www.targetvalidation.org/target/ENSG00000172757/associations	TESK2 PDXP SSH1 SSH3 NRK SSH2 TESK1 PRKD1 ILK PPP2R5C YWHAH ACTA1 ACTC1 ACTG1 ACTA2 ACTB LIMK2 LIMK1 PPP1CC PRKCA IL7R SRC ISG15 ATP1A1 ACTB LIMK1 ACTC1 TPI1 ACTG1 ACTA1 SSH1 CLCN5	Nucleus matrix		Yes	https://www.drugbank.ca/biodb/polypeptides/P23528	✓
CHEK1	HUMAN Serine/threonine-protein kinase Chk1	CHK1	O14757	ENSG0000(	Kinase,Cancer	JF289264	Melanoma, Medulloblastoma, Colorectal cancer, Small cell lung carcinoma, Lymphoid neoplasm, Glioma, Glioblstoma multiforme, Large cell lung carcinoma, Psoriasis, Polyploidy	https://www.targetvalidation.org/target/ENSG00000149554/associations	CLSPN TESK2 CDK5RAP3 ATRIP HIPK1 DDB1 PDK1 ATR BIRC2 ATM RAD51 E2F1 CDK3 XIAP CSNK2A1 CSNK2B PPP2CA YWHAZ PPP2CB IRAK1 MAP3K8 BRCA1 AKT1 CDK2 RPA2 CDK1 TP53 CHEK2 NUAK1 PPM1D	Nucleus, Cytoplasm, cytoskeleton, microtubule organizing center, centrosome,		Yes	https://www.bindingdb.org/uniprot/O14757	✓
CHEK2	HUMAN Serine/threonine-protein kinase Chk2	CDS1, CHK2, HuCds1, RAD53	O96017	ENSG0000(	Kinase,Cancer	AY551295	Epithelial neoplasm, Hereditary breast cancer, Prostate adenocarcinoma, Li-Fraumeni syndrome, Lung carcinoma, Osteosarcoma, Squamous cell carcinoma, Lymphoid neoplasm, Esophageal carcinoma, Colonic neoplasm	https://www.targetvalidation.org/target/ENSG00000183765/associations	MAP3K20 PLK3 TERF2 MDC1 ATR ATM E2F1 MDM2 PRKDC CSNK2B PPP2CA PPP2CB PLK1 KPNA2 CDK9 BRCA1 TTK MARK3 EIF2AK2 UBR5 RNF8 NBN PPM1D CHEK1 CDC7 TERF2 E2F1 LIG1 MUS81 MSH2 VHL BRCA1 PLK3 KPNA2	Nucleus,		Yes	https://www.bindingdb.org/uniprot/O96017	✓

CHI3L1	HUMAN Chitinase-3-like protein 1	GP39, YKL40	P36222	ENSG0000(	Cancer	AK312911	Asthma, Central nervous system cancer, Breast carcinoma, Glioma, Astrocytoma, Crohn's disease, Schizophrenia, Glioblastoma, Degenerative polyarthritis	https://www.targetvalidation.org/target/ENSG00000133048/associations		Secreted, extracellular space		No				✓
CHIC2	HUMAN Cysteine-rich hydrophobic domain-containing protein 2	BTL	Q9UKJ5	ENSG0000(	Cancer	BC034691	Lung carcinoma, Breast ductal adenocarcinoma, Leukemia, Myeloid neoplasm, Melanoma, Acute myelocytic leukemia, Eosinophilia, Systemic mastocytosis, Squamous cell lung carcinoma, Lung adenocarcinoma	https://www.targetvalidation.org/target/ENSG00000109220/associations		Cell membrane		No				✓
CHMP3	HUMAN Charged multivesicular body protein 3	CGI-149, NEDF, VPS24	Q9Y3E7	ENSG0000(	Cancer,Cancer-Antigen	AK290725	Lung carcinoma, Lung adenocarcinoma, Pancreatic carcinoma, HIV infection, Herpes simplex infection, Down syndrome, Prostate carcinoma, Amyotrophic lateral sclerosis, , Breast carcinoma, Tuberculosis	https://www.targetvalidation.org/target/ENSG00000115561/associations	VPS4A CHMP6 IGFBP7 STAMBP	Cytoplasm, cytosol, Membrane, Lipid-anchor, Endosome, Late endosome membrane		No				✓
CHN1	HUMAN N-chimaerin	ARHGAP2, CHN, DURS2	P15882	ENSG0000(	Cancer	AK289941	Duane retraction syndrome, Central nervous system cancer, Force expiratory volume, Glioblastoma multiforme, Astrocytoma, Polycythemia vera, Duane retraction syndrome type 2, Strabismus, Familial LCAT deficiency, Amyotrophic lateral sclerosis	https://www.targetvalidation.org/target/ENSG00000128656/associations	ACVR1 RAC1 TGFBR1 ERBB2 BMPR1B RAC1 CDK5R1 GRIN2A			No				✓
CHST4	HUMAN Carbohydrate sulfotransferase 4	HEC-GLCNAC-6-ST, LSST	Q8NCG5	ENSG0000(	Others	AK074746	Hepatocellular carcinoma, Pancreatic adenocarcinoma, Prostate carcinoma, Colorectal adenocarcinoma, Hyper-IgM syndrome type 3, Mucinous adenocarcinoma, Adenocarcinoma, Acute promyelocytic leukemia, Intestinal volvulus	https://www.targetvalidation.org/target/ENSG00000140835/associations	CD34	Golgi apparatus membrane	Transmembrane	No				✓
CIDEB	HUMAN Cell death activator CIDE-B	CIDEB	Q9UHD4	ENSG0000(	Others	AK000387	Obesity due to congenital leptin deficiency, Berardinelli-Seip congenital lipodystrophy, Hypertriglyceridemia, Colorectal cancer, Hepatocellular carcinoma, Prostate neoplasm, Pancreatic ductal adenocarcinoma	https://www.targetvalidation.org/target/ENSG00000136305/associations	CIDEA			No				✓
CIDEC	HUMAN Cell death activator CIDE-3	CIDE-3, FLJ20871, Fsp27	Q96AQ7	ENSG0000(	Others	AK024524	Familial partial lipodystrophy, Lipodystrophy, Mandibuloacral dysplasia, Lung carcinoma, Familial partial lipodystrophy, Dunnigan type, Steatohepatitis, Barth syndrome, Alagille syndrome, Fanconi anemia	https://www.targetvalidation.org/target/ENSG00000187288/associations		Nucleus Endoplasmic reticulum, Lipid droplet,		No				✓
CISH	HUMAN Cytokine-inducible SH2-containing protein	CIS, CIS-1, G18, SOCS	Q9NSE2	ENSG0000(	Kinase,Signalling	AK313850	Cervical carcinoma, Breast carcinoma, Autoimmune lymphoproliferative syndrome, Common variable immunodeficiency, Wiskott-Aldrich syndrome, Tuberculosis, Liver carcinoma, Astrocytoma, Chronic myeloproliferative disorder	https://www.targetvalidation.org/target/ENSG00000114737/associations	ELOB ELOC PRKCQ NFKBIA EPOR PRKCA IL2RB GHR KIT PRKCB ERBB2 EGFR ABL1 EPOR STAT5A LEPR PRKCQ KIT BCL2L11 PRLR GHR CSF2RB IL2RB			No				✓

CITED1	HUMAN Cbp/p300-interacting transactivator 1	MSG1, CITE1	Q99966	ENSG0000(	Transcription factor	AL135749	Breast carcinoma, Melanoma, Papillary thyroid carcinoma, Wilms tumor, Colorectal cancer, Nephroblastoma, Embryonal neoplasm, Hepatoblastoma, Central nervous system cancer, Glioblastoma multiforme	https://www.targetvalidation.org/target/ENSG00000125931/associations	CREBBP SMAD4 EP300 RELA HSPA8 HSPA8 SMAD4	Nucleus	No		✓
CKB	HUMAN Creatine kinase B-type	CKBB, KCRB	P12277	ENSG0000(	Kinase,Cancer	M16364	Prostate carcinoma, Li-Fraumeni syndrome, Melanoma, Seizures, Lung adenocarcinoma, Breast carcinoma, Diffuse large B-cell lymphoma, Schizophrenia, Left ventricular hypertrophy, Colorectal cancer	https://www.targetvalidation.org/target/ENSG00000166165/associations	CKM ASB9	Cytoplasm,	Yes	https://www.drugbank.ca/biodb/polypeptides/P12277	✓
CKM	HUMAN Creatine kinase M-type	CKMM, KCRM	P06732	ENSG0000(	Kinase	AY585238	Creatine kinase measurement, Response to statin, Li-Fraumeni syndrome, Corticosteroid-binding globulin deficiency, Psoriasis, Glycogen storage disease due to muscle and heart glycogen synthase deficiency, Myotonic dystrophy, Muscular dystrophy, Pyruvate dehydrogenase deficiency, Becker muscular dystrophy	https://www.targetvalidation.org/target/ENSG00000104879/associations	MYOM2 CKB	Cytoplasm,	Yes	https://www.bindingdb.org/uniprot/P06732	✓
CKMT2	HUMAN Creatine kinase S-type, mitochondrial	SMTCK, KCRS	P17540	ENSG0000(	Kinase	NA	Colorectal cancer, Psoriasis, Gastric carcinoma, Clear cell renal carcinoma, Werner syndrome, Gastric cardia carcinoma, Acute myelocytic leukemia	https://www.targetvalidation.org/target/ENSG00000131730/associations		Mitochondrion inner membrane, Peripheral membrane protein, Intermembrane side,	Yes	https://www.drugbank.ca/biodb/polypeptides/P17540	✓
CKS1B	HUMAN Cyclin-dependent kinases regulatory subunit 1	CKS1, ckshs1, PNAS-143, PNAS- 16	P61024	ENSG0000(	Kinase,Cancer	BT007196	Multiple myeloma, Leukemia, Hepatocellular carcinoma, Lung carcinoma, Monoclonal gammopathy, Breast carcinoma, Mammary neoplasm, Squamous cell carcinoma, Gastric cardia carcinoma, Melanoma, Renal cell carcinoma	https://www.targetvalidation.org/target/ENSG00000173207/associations	CUL1 SKP2 CDK3 SKP1 CDK2 CDK1 CUL1 CDKN1B SKP2 CDK2 CDK1 SKP1		Yes	https://www.drugbank.ca/biodb/polypeptides/P61024	✓
CLK1	HUMAN Dual specificity protein kinase CLK1	CLK1	P49759	ENSG0000(	Kinase	AK294295	Pseudohypoaldosteronism type 2, Kenny-Caffey syndrome, Familial isolated hypoparathyroidism, Lung carcinoma, Albright hereditary osteodystrophy, Ovarian carcinoma, Osteocraniostenosis, Autosomal dominant hypocalcemia, Distal renal tubular acidosis, Large cell lung carcinoma	https://www.targetvalidation.org/target/ENSG00000013441/associations	CAMKK1 ISG15 YWHAG PTPN1 TRIM28 ABL1 PPP2R5D PIAS4 PPP1R13L MDM4 SRSF1 SRPK1 MBP CSN1S1 RBM17 SNRNP70 PRPF4 LUC7L3 PPIG SRSF6 UBL5 GOLM1	Nucleus,	Yes	https://www.bindingdb.org/uniprot/P49759	✓
CLK2	HUMAN Dual specificity protein kinase CLK2	clk2	P49760	ENSG0000(	Kinase	AL713999	Breast carcinoma, Glioblastoma multiforme, Alzheimer's disease, Pancreatic carcinoma, Prostate carcinoma, Obesity, Mental retardation	https://www.targetvalidation.org/target/ENSG00000176444/associations	AKT1 YWHAG PRKACA PTPN1 ARID3A RPA1 TRIM28 MATR3 MTA1 ZFP36 MDM2 PPP1R13L BAD CAMKK1 XPO1 KHDRBS1 NFATC4 EIF2B5 SUB1 CLK3 URI1 ZMYM3 U2AF1 SAFB2 HLCS PRDM10 GPBP1L1	Nucleus, Nucleus speckle,	Yes	https://www.bindingdb.org/uniprot/P49760	✓
CLK3	HUMAN Dual specificity protein kinase CLK3	clk3	P49761	ENSG0000(	Kinase	AC100835	Bladder carcinoma, Melanoma, Central nervous system cancer, Breast carcinoma, Glioblastoma multiforme, Pancreatic carcinoma, Neurodegenerative disease	https://www.targetvalidation.org/target/ENSG00000179335/associations	CLK2 MAPK1 CHEK1 YWHAG PRKCD PPP2R5D PPP1R13L SRSF1 ANKS1A RSRP1 HLCS	Nucleus, Cytoplasm, Nucleus speckle,	Yes	https://www.bindingdb.org/uniprot/P49761	✓
CLUAP1	HUMAN Clusterin-associated protein 1	KIAA0643, CLUA1	Q96AJ1	ENSG0000(	Cancer,Cancer-Antigen	AK023359	Leber congenital amaurosis, Osteosarcoma, Breast carcinoma, Colonic neoplasm, Lung carcinoma, Squamous cell carcinoma, Retinopathy, Psoriasis, Hepatocellular carcinoma, Breast adenocarcinoma	https://www.targetvalidation.org/target/ENSG00000103351/associations	CLU	Cell projection, cilium	No		✓

CMC4	HUMAN Cx9C motif -containing protein 4	MTCP1, MTCP1NB, P8MTCP1	P56277	ENSG0000(	Signalling	CH471172	Breast carcinoma, Gastric adenocarcinoma, Cutaneous melanoma, Ataxia telangiectasia, T-cell leukemia, Bladder carcinoma, Colorectal adenocarcinoma, Squamous cell carcinoma, Prostate adenocarcinoma, Ovarian serous adenocarcinoma	https://www.targetvalidation.org/target/ENSG00000182712/associations	AKT2	Mitochondrion	No		✓
CMPK1	HUMAN UMP-CMP kinase	CMK, UCK, UMK, UMPK, CMPK, UMP-CMPK	P30085	ENSG0000(	Kinase	AF087865	Breast carcinoma, Pancreatic ductal adenocarcinoma, Diffuse large B-cell lymphoma, Atopic eczema, Multiple myeloma, Acute megakaryocytic leukemia, Down syndrome, Small cell lung carcinoma, Glioblastoma multiforme, HPV infection	https://www.targetvalidation.org/target/ENSG00000162368/associations		Nucleus	Yes		✓
												https://www.drugbank.ca/biodb/polypeptides/P30085	
CNN1	HUMAN Calponin-1	Sm-Calp, SMCC	P51911	ENSG0000(	Signalling, Cancer	AK300837	Lung adenocarcinoma, Breast carcinoma, Ovarian carcinoma, Li-Fraumeni syndrome, Metastatic prostate cancer, Liver carcinoma, Colonic neoplasm, Colorectal cancer, Hereditary arterial and articular multiple calcification syndrome, Fibrodysplasia ossidicans progreesiva	https://www.targetvalidation.org/target/ENSG00000130176/associations	PRKCE FYN ACTA1		No		✓
CNOT2	HUMAN CCR4-NOT transcription complex subunit 2	CDC36, NOT2, NOT2H	Q9NZN8	ENSG0000(	Transcription factor	AK000662	Glioblastoma multiforme, Astrocytoma, Amyotrophic lateral sclerosis, Ovarian carcinoma, Teratozoospermia, Obesity, Lung carcinoma, Pancreatic carcinoma	https://www.targetvalidation.org/target/ENSG00000111596/associations	ATR CNOT1	Cytoplasm	No		✓
CNOT8	HUMAN CCR4-NOT transcription complex subunit 8	CAF1, CALIF, hCAF1, POP2	Q9UFF9	ENSG0000(	Transcription factor	AL122045	Melanoma, Glioblastoma multiforme, Lung carcinoma, Atopic eczema, Medulloblastoma, Breast carcinoma, Psoriasis, Crohn's disease	https://www.targetvalidation.org/target/ENSG00000155508/associations	PUM1 CCR4 STAT1 PYCARD	Cytoplasm	No		✓
CNOT9	HUMAN CCR4-NOT transcription complex subunit 9	CNOT9, RCD1, RQCD1	Q92600	ENSG0000(	Others	CH471063	Melanoma, Breast neoplasm, Cutaneous melanoma, Lung carcinoma, Small cell lung carcinoma, Retinal dystrophy, Steinert myotonic dystrophy, Multiple myeloma, Pulmonary tuberculosis, Celiac disease	https://www.targetvalidation.org/target/ENSG00000144580/associations	MAP3K3 SOS1 MYB ZNF335	Nucleus	No		✓
CNRIP1	HUMAN CB1 cannabinoid receptor-interacting protein 1	C2orf32, CRIP1a, CRIP1b, DKFZP566K1924, CNRP1	Q96F85	ENSG0000(	Others	CH471053	Lung carcinoma, Breast carcinoma, Ovarian carcinoma, Neuroblastoma, Pancreatic carcinoma, Large cell lung carcinoma, Crohn's disease, Duchenne muscular dystrophy, Multiple sclerosis, Gastric carcinoma	https://www.targetvalidation.org/target/ENSG00000119865/associations			No		✓
COL4A3BP	HUMAN Collagen type IV alpha-3-binding protein	CERT, GPBP, STARD11, C43BP	Q9Y5P4	ENSG0000(	Kinase	AY453385	Autosomal dominant non-syndromic intellectual disability, Severe congenital neutropenia, Autosomal dominant severe congenital neutropenia, Agammaglobulinemia, Isolated agammaglobulinemia, Congenital Epicanthus, Hermansky-pudlak syndrome, Barth syndrome, Blackfan-Diamond anemia, Shwachman-Diamond syndrome	https://www.targetvalidation.org/target/ENSG00000113163/associations	PPM1L PRKD1 CSNK1G2 BLVRA PPM1L COL4A3	Cytoplasm	Yes		✓
												https://www.bindingdb.org/uniprot/Q9Y5P4	
COMMD3	HUMAN COMM domain-containing protein 3	BUP, C10orf8	Q9UBI1	ENSG0000(	Others	AL158211	Lymphoma, Metastatic prostate cancer, Ovarian carcinoma, Multiple myeloma, Obesity, B-Cell lymphoma, Cervical carcinoma, Schizophrenia, Unipolar depression, Bipolar disorder	https://www.targetvalidation.org/target/ENSG00000148444/associations		Cytoplasm	No		✓
COPG2	HUMAN Coatomer subunit gamma-2	2-COP	Q9UBF2	ENSG0000(	Cancer,Cancer-Antigen	AK291409	Autism, Tuberculosis, Cystic fibrosis, Silver-Russell syndrome, Breast carcinoma, Rheumatoid arthritis	https://www.targetvalidation.org/target/ENSG00000158623/associations		Cytoplasm, cytosol	No		✓

COPS6	HUMAN COP9 signalosome complex subunit 6	CSN6, HVIP	Q7L5N1	ENSG0000(	Cancer,Cancer-Antigen	U70735	Cervical carcinoma, Breast carcinoma, Neoplasm of mature B-cells, Glioblastoma multiforme, Colonic neoplasm, HIV infection, Lymphoma, Psoriasis, Pancreatic carcinoma, Colorectal cancer	https://www.targetvalidation.org/target/ENSG00000168090/associations	BTRC NOD1 COPS3 NOD2 COPS4 TRIB3 STK40 NEDD8 CASP8 CUL1 SKP2 GPS1 SKP1 RBX1 COPS2 CASP3 AKT1 MAPK1 NR3C1 CASP3 COPS5 CASP8 NOD1 COPS8 COPS7A COPS7B	Nucleus, Cytoplasm,		No		✓
COQ8A	HUMAN Atypical kinase COQ8A, mitochondrial	ADCK3, CABC1, PP265	Q8NI60	NA	Kinase	AK074693	Autosomal recessive ataxia due to ubiquinone deficiency, Coenzyme Q10 deficiency, Nystagmus, Melanoma, Osteosarcoma, Cerebellar ataxia, Seizures, Osteosarcoma	https://www.targetvalidation.org/target/ENSG00000163050/associations	IRAK1 RABAC1	Mitochondrion	Transmembrane	Yes		✓
													https://www.bindingdb.org/uniprot/Q8NI60	
COQ8B	HUMAN Atypical kinase COQ8B, mitochondrial	COQ8B	Q96D53	NA	Kinase	NA	Nephrotic synfrome, Familial idiopathic steroid-resistant nephrotic syndrome, Seizures, Retinitis pigmentosa, Renal glomerular disease, Kidney disease	https://www.targetvalidation.org/target/ENSG00000123815/associations		Mitochondrion membrane	Transmembrane	Yes		✓
													https://www.bindingdb.org/uniprot/Q96D53	
COTL1	HUMAN Coactosin-like protein	CLP	Q14019	ENSG0000(	Others	CH471114	Adenocarcinoma, Colorectal adenocarcinoma, Lung carcinoma, Astrocytoma, Psoriasis, Mammary neoplasm, Rheumatoid arthritis, Acute lymphoblastic leukemia, Renal cell carcinoma, Duchenne muscular dystrophy	https://www.targetvalidation.org/target/ENSG00000103187/associations	ACTA1 ACTB IL7R ALOX5	Cytoplasm		No		✓
COX6B2	HUMAN Cytochrome c oxidase subunit 6B2	CT59, CX6B2	Q6YFQ2	ENSG0000(	Others	BC100899	Colorectal adenocarcinoma, Azoospermia, Pulmonary mucoepidermoid carcinoma	https://www.targetvalidation.org/target/ENSG00000160471/associations		Mitochondrion intermembrane space		No		✓
COX6C	HUMAN Cytochrome c oxidase subunit 6C	COX6C	P09669	ENSG0000(	Cancer	AK311791	Melanoma, Colonic neoplasm, Lung adenocarcinoma, Breast carcinoma, Rectal adenocarcinoma, Prostate neoplasm, Esophageal squamous cell carcinoma, Small cell lung carcinoma, Hepatocellular carcinoma, Cutaneous melanoma	https://www.targetvalidation.org/target/ENSG00000164919/associations		Mitochondrion inner membrane,	Transmembrane	Yes		✓
													https://www.drugbank.ca/biodb/polypeptides/P09669	
CPA4	HUMAN Carboxypeptidase A4	CPA3, CBPA4, UNQ694/PRO1339	Q9UI42	ENSG0000(	Others	AC024085	Breast carcinoma, Lung carcinoma, Non-small cell lung carcinoma, Colorectal adenocarcinoma, Ovarian carcinoma, Prostate neoplasm, Psoriasis, Uterine fibroid, Lung adenocarcinoma, Liver cirrhosis	https://www.targetvalidation.org/target/ENSG00000128510/associations		Secreted		No		✓
CPM	HUMAN Carboxypeptidase M	CPM	P14384	ENSG0000(	Others	AK313180	Breast carcinoma, Colorectal cancer, Lung carcinoma, Li-Fraumeni syndrome, Ovarian carcinoma, Endometriosis, Silicosis, Unipolar depression, Inflammation, Large cell lung carcinoma	https://www.targetvalidation.org/target/ENSG00000135678/associations	EGF HBA1; HBA2	Cell membrane		Yes		✓
													https://www.bindingdb.org/uniprot/P14384	
CPXCR1	HUMAN CPX chromosomal region candidate gene 1 protein	CT77, CPXCR	Q8N123	ENSG0000(	Others	AL031116	Anti-neutrophil antibody associated vasculitis, Azoospermia, Teratozoospermia, Uterine fibroid, Gastric neoplasm, X-linked cleft palate	https://www.targetvalidation.org/target/ENSG00000147183/associations				No		✓
CRADD	HUMAN Death domain-containing protein CRADD	RAIDD	P78560	ENSG0000(	Cancer	AC012085	Autosomal recessive non-syndromic intellectual disability, Breast carcinoma, Lymphoma, Body height, Rheumatoid arthritis, Renal cell carcinoma, Narcolepsy, Osteosarcoma, Down syndrome, Lissencephaly	https://www.targetvalidation.org/target/ENSG00000169372/associations	PIDD1 RIPK1 CASP2 TRIM24 CASP2 CASP8 RIPK1	Cytoplasm		No		✓

CREB1	HUMAN Cyclic AMP - responsive element-binding protein 1	CREB1	P16220	ENSG0000(	Transcription factor,Cancer	M34356	Adenocarcinoma, Prostate carcinoma, Lymphoma, Prostate carcinoma, Oral cavity cancer, Melanoma, Gastric adenocarcinoma, Hepatocellular carcinoma, Colorectal adenocarcinoma, Bladder transitional cell carcinoma	https://www.targetvalidation.org/target/ENSG00000118260/associations	KCNIP3 AKT3 CAMK2A RPS6KA6 SGK2 HIPK2 SGK494 AURKB SGK3 KAT5 CREBBP CAMKK1 TSSK4 CRTC2 CAMK4 MAPK14 SMAD2 RPS6KA1 RPS6KA2 PRKD1 CAMK1 PRKG1 DYRK1A CAMK2D ATR SMAD4 PPP2R5C ATM PRKG2	Nucleus	Yes	https://www.drugbank.ca/biodb/polypeptides/P16220	✓
CREB5	HUMAN Cyclic AMP - responsive element-binding protein 5	CREBPA	Q02930	ENSG0000(	Transcription factor	L05913	Breast carcinoma, Li-Fraumeni syndrome, Glioma, Glioblastoma multiforme, Hepatocellular carcinoma, Osteosarcoma, Folding of antihelix, Diabetic retinopathy, Hypertriglyceridemia, Colorectal cancer	https://www.targetvalidation.org/target/ENSG00000146592/associations	ATF2 JUN	Nucleus	No		✓
CRISP2	HUMAN Cysteine-rich secretory protein 2	CT36, GAPDL5, TPX1, CRIS2	P16562	ENSG0000(	Others	AY292862	Azoospermia, Esophageal squamous cell carcinoma, Osteosarcoma, Psoriasis, Medulloblastoma, Melanoma, Chronic lymphocytic leukemia, Pancreatic carcinoma	https://www.targetvalidation.org/target/ENSG00000124490/associations		Secreted	No		✓
CROT	HUMAN Peroxisomal carnitine O-octanoyltransferase	COT, OCTC	Q9UKG9	ENSG0000(	Kinase	CH236949	Malignant mesothelioma, Breast carcinoma, Adenocarcinoma, Breast adenocarcinoma, Ovarian carcinoma	https://www.targetvalidation.org/target/ENSG00000005469/associations		Peroxisome	Yes	https://www.bindingdb.org/uniprot/Q9UKG9	✓
CRX	HUMAN Cone-rod homeobox protein	CORD2, CRD, LCA7, OTX3	O43186	ENSG0000(	Transcription factor	BC016664	Retinal dystrophy, Keratoconus, Leber congenital amaurosis, Cone rod dystrophy, Retinitis pigmentosa, Severe visual impairment, Bietti crystalline dystrophy, Achromatopsia, MRCS syndrome, Late-onset retinal degeneration	https://www.targetvalidation.org/target/ENSG00000105392/associations	SP4 PDC SP1 ATXN7 BANF1 NR2E3 NRL RAX2	Nucleus	No		✓
CRYAB	HUMAN Alpha-crystallin B chain	CRYA2, HSPB5	P02511	ENSG0000(	Cancer,Cancer-Antigen	AK314029	Myofibrillar myopathy, Cardiomyopathy, Alpha-crystallinopathy, Familial dilated cardiomyopathy, Zonular cataract, Posterior polar cataract, Fatal infantile hypertonic myofibrillar myopathy, Desminopathy, Breast carcinoma, Cystic fibrosis	https://www.targetvalidation.org/target/ENSG00000109846/associations	HSPB8 TTN MAPK14 BMPR2 SRC CRYAA AVP PSIP1 MAF CDH16 TP53 APP CASP3 HSPB2 SNCA BCL2L1 BAX HSPB1 VEGFA PSMA3 FCGR2A TMEM109 CRYGD LBH CRYGS	Cytoplasm	No		✓
CSAG1	HUMAN Putative chondrosarcoma-associated gene 1 protein	CSAGE, CT24.1	Q6PB30	ENSG0000(	Others	BC059947	Li-Fraumeni syndrome, Hepatocellular carcinoma, Tongue squamous cell carcinoma, Prostate carcinoma, Breast carcinoma, Melanoma, Chondrosarcoma	https://www.targetvalidation.org/target/ENSG00000198930/associations			No		✓
CSAG2	HUMAN Chondrosarcoma-associated gene 2/3 protein	CSAG2	Q9Y5P2	NA	Others	BC007228	Lung carcinoma, Non-small cell lung carcinoma, Psoriasis, Ovarian carcinoma, Tongue squamous cell carcinoma, Central nervous system cancer, Melanoma, Renal cell carcinoma, Cutaneous lupus erythematosus, Azoospermia	https://www.targetvalidation.org/target/ENSG00000268916/associations			No		✓
CSK	HUMAN Tyrosine-protein kinase CSK	CSK	P41240	ENSG0000(	Kinase	CR541960	Diastolic blood pressure, Systolic blood pressure, Mean arterial pressure, Frontal encephalocele, Ovarian carcinoma, Hyperalgesia, Cystic Fibrosis, Hepatitis C infection, Systemic lupus erythematosus, Hypertension	https://www.targetvalidation.org/target/ENSG00000103653/associations	PAG1 DOK1 CREBBP DAG1 PRKCD PTK2 CAV1 DAB2 PXN AXL CD247 PRKACA PECAM1 SRC IGF1R FYN LCK INSR EGFR IKBKB YES1 HCK ITCH GJA1 CAV1 CTNNB1 EGFR CTTN LYN PRKACA LCK SRC ARRB1 FYN XRCC5	Cytoplasm	Yes	https://www.bindingdb.org/uniprot/P41240	✓

CSNK1D	HUMAN Casein kinase I isoform delta	CKID, CKIdelta, HCKID, KC1D	P48730	ENSG0000(	Kinase	AK291758	Familial advanced sleep-phase syndrome, Osteosarcoma, Lung carcinoma, Polyp, Septic shock, Bipolar disorder, Schizophrenia, Unipolar depression, Attention deficit hyperactivity disorder, Migraine disorder	https://www.targetvalidation.org/target/ENSG00000141551/associations	CSNK1E LRP6 AXIN1 GJA1 CTNNB1 WWTR1 CDK5 CDH1 HIF1A APC YAP1 TP53 BACE1 PPP1R14A YWHAQ NCOA3 LRP6 GSK3B CSNK1E PER1 PER2 DVL1 SNCA LEF1 MDM2 PER3 PPP1R13L MAPT AXIN1 UHRF1	Cytoplasm, Nucleus, cytoskeleton, microtubule organizing center, centrosome, perinuclear region, Cell membrane, spindle, Golgi apparatus,	Yes	https://www.bindingdb.org/uniprot/P48730	✓
CSNK1G1	HUMAN Casein kinase I isoform gamma-1	CK1gamma1, KC1G1	Q9HCP0	ENSG0000(	Kinase	CH471082	Lung carcinoma, Adenocarcinoma, Pancreatic ductal adenocarcinoma, Gastric carcinoma, Epilepsy, Melanoma, Colorectal adenocarcinoma, Diffuse large B-cell lymphoma, Ovarian carcinoma	https://www.targetvalidation.org/target/ENSG00000169118/associations	CASKIN1 AMER1 APBA1 CSNK1E APC LRP6 DVL1 ATP2B1 STX3 CTNNB1 CDK5 APC FOXO1 APBA1 TP53 PPP1R14A YWHAQ LRP6 CSNK1E DVL1 MAPT NFATC1 PSEN2 TBR1 CASKIN1 ANK1 SERPINB2 NFATC3 SLC18A2 PAXBP1	Cytoplasm,	Yes	https://www.bindingdb.org/uniprot/Q9HCP0	✓
CSNK1G2	HUMAN Casein kinase I isoform gamma-2	CK1g2, KC1G2	P78368	ENSG0000(	Kinase	AB451278	Melanoma, Breast carcinoma, Febrile Seizures, Psoriasis, Rheumatoid arthritis, Febrile convulsions, Hepatitis C infection, Liposarcoma, Follicular thyroid adenoma, Papillary thyroid carcinoma	https://www.targetvalidation.org/target/ENSG00000133275/associations	CSNK1E APC BMPR1B CTNNB1 CDK5 APC BID TP53 PPP1R14A SMAD3 YWHAQ MTA1 BECN1 CSNK1E PER1 NCK1 ZYX PSEN2 COL4A3BP NFATC3 CC2D1A SLC18A2 APPBP2	Cytoplasm	Yes	https://www.bindingdb.org/uniprot/P78368	✓
CSNK2A1	HUMAN Casein kinase II subunit alpha	CSK21, CK2A1	P68400	ENSG0000(	Kinase,Cancer	X70251	Achondrogenesis, Gaucher disease, Free sialic acid storage disease, Achondrogenesis, Schizophrenia, Anoxia, Myocardial infarction, Trisomy 13, Lethal multiple pterygium syndrome, Greenberg dysplasia	https://www.targetvalidation.org/target/ENSG00000101266/associations	FAF1 HSPB8 CDK12 CARD9 CDK19 CDK20 MAPK14 CDK10 NOLC1 CDK13 PIN1 CDK18 PRKCZ CDK17 CDK16 CDK5 CDK6 CDK3 CSNK2B CDK9 CDK7 GSK3A CDK8 CTNNB1 AKT1 MAPK1 NFKBIA APC CDK2 UBA1	Nucleus	Yes	https://www.bindingdb.org/uniprot/P68400	✓
CSNK2A2	HUMAN Casein kinase II subunit alpha'	CSK22, CK2A2	P19784	ENSG0000(	Kinase,Cancer	NA	Male infertility with teratozoospermia due to single gene mutation, Male infertility due to globozoospermia, Male infertility with azoospermia or oligozoospermia due to single gene mutation, Non-syndromic male infertility due to sperm motility disorder, Telomere length, Partial androgen insensitivity syndrome, Obesity due to CEP19 deficiency, Partial chromosome Y deletion, Young syndrome, Primary ciliary dyskinesia	https://www.targetvalidation.org/target/ENSG00000070770/associations	MAPK14 CSNK2A1 CSNK2B GSK3B PTEN FAF1 CABP1 CAV1 CTNNB1 AQP4 CALM1 CDH1 ARRB2 PTPN1 PACS1 MAX CSNK2A1 LIG1 BID FGF2 TP53 CCNH HSP90AA1 HSP90AB1 STX1A TNF HDAC2 SMC3 PPP1R1B JUN SET		Yes	https://www.bindingdb.org/uniprot/P19784	✓
CSRNP1	HUMAN Cysteine/serine-rich nuclear protein 1	AXUD1, TAIP3, URAX1, CSRN1	Q96S65	ENSG0000(	Transcription factor	BC038949	Breast carcinoma, Paragraph delayed recall mesureament, Memory performance, Glioma, Chronic lymphocytic leukemia, Dilated cardiomyopathy, Campomelic dysplasia, Hypertrihlyceridemia, Lung carcinoma, Nijmegen breakage syndrome	https://www.targetvalidation.org/target/ENSG00000144655/associations		Nucleus	No		✓
CST4	HUMAN Cystatin-S	CYTS	P01036	ENSG0000(	Others	AL359433	Lung carcinoma, Breast carcinoma, Prostate carcinoma, Metastatic prostate cancer, Pancreatic ductal adenocarcinoma, Bacterial vaginosis, Breast adenocarcinoma, Bronchiectasis, Colorectal cancer, Melanoma	https://www.targetvalidation.org/target/ENSG00000101441/associations	CSNK2A1	Secreted	No		✓
CSTB	HUMAN Cystatin-B	CST6, EPM1, STFB	P04080	ENSG0000(	Signalling, Cancer	AP001752	Epilepsy syndrome, Progressive myoclonic epilepsy, Unverricht-Lundborg disease, Squamous cell carcinoma, Neurodegenerative disease, Meningioma, Myoclonus, Dysarthria, Esophagus squamous cell carcinoma, Tonic-clonic seizures	https://www.targetvalidation.org/target/ENSG00000160213/associations	CTSD	Cytoplasm	No		✓
CT45A1	HUMAN Cancer/testis antigen family 45 member A1	CT45-1, CT45.1, CT451	Q5HYN5	ENSG0000(	Cancer	NA	Li-Fraumeni syndrome, Gastric carcinoma, Breast carcinoma, Hepatocellular carcinoma, Adenocarcinoma, Ovary neoplasm	https://www.targetvalidation.org/target/ENSG00000268940/associations			No		✓

CT47A1	HUMAN Cancer/testis antigen 47A	CT47.1, CT47A	Q5JQC4	ENSG0000(	Transcription factor,Cancer	AL670379	Lung carcinoma, Breast carcinoma, Colorectal cancer, Esophageal squamous cell carcinoma, Facioscapulohumeral dystrophy, Small cell lung carcinoma	https://www.targetvalidation.org/target/ENSG00000226685/associations				No		✓
CT55	HUMAN Cancer/testis antigen 55	CXorf48	Q8WUE5	ENSG0000(	Cancer	AL670455	Breast Carcinoma, Breast neoplasm, Familial prostate cancer, Head and Neck squamous cell carcinoma, Astrocytoma, Ovarian carcinoma, Fanconi anemia	NA				No		✓
CT62	HUMAN Cancer/testis antigen 62	CT62	P0C5K7	ENSG0000(	Cancer	NA	Azoospermia, Breast carcinoma, Psoriasis, Cervical carcinoma	https://www.targetvalidation.org/target/ENSG00000225362/associations				No		✓
CT83	HUMAN Kita-kyushu lung cancer antigen 1	CXorf61, KKLC1	Q5H943	ENSG0000(	Cancer	NA	Azoospermia, Breast carcinoma, Ovarian carcinoma, Medulloblastoma, Pancreatic ductal adenocarcinoma, Adenocarcinoma, Breast neoplasm, Squamous cell carcinoma, Melanoma, Gastric carcinoma	https://www.targetvalidation.org/target/ENSG00000204019/associations		Cell membrane	Transmembrane	No		✓
CTAG1A	HUMAN Cancer/testis antigen 1	NY-ESO-1, CTAG, CTAG1, LAGE2, LAGE2A, CTG1B,NY-ESO-1 ORF2	P78358	ENSG0000(	Cancer	AJ275977	Liposarcoma, Gastric carcinoma, Round cell liposarcoma, Glioma, Testicular germ cell tumor, Melanoma, Non-small cell lung carcinoma, Esing sarcoma, Multiple myeloma, Esophageal squamous cell carcinoma	https://www.targetvalidation.org/target/ENSG00000268651/associations		Cytoplasm		No		✓
CTAG2	HUMAN Cancer/testis antigen 2	CAMEL, CT6.2a, CT6.2b, ESO2, LAGE-1, LAGE-1a, LAGE-1b, LAGE1, MGC138724, MGC3803	O75638	ENSG0000(	Cancer,Cancer-Antigen	AJ223041	Central nervous system cancer, Lung carcinoma, Melanoma, Breast carcinoma, Hepatocellular carcinoma, Multiple myeloma, Non-small cell lung carcinoma, Bladder carcinoma	https://www.targetvalidation.org/target/ENSG00000126890/associations				No		✓
CTAGE1	HUMAN cTAGE family member 2	CT21.2, cTAGE-1, cTAGE-2	Q96RT6	ENSG0000(	Others	NA	T-cell cutaneous lymphoma, Tarsal-carpal coalition syndrome	NA		Membrane	Transmembrane	No		✓
CTAGE5	HUMAN cTAGE family member 5	MEA6, MGEA, MEA11, MGEA11, MGEA6, CTGE5	O15320	ENSG0000(	Others	AF338234	Breast carcinoma, T-cell acute lymphoblastic leukemia, Gastric carcinoma, Hepatocellular carcinoma, Leukemia, Meningioma, Liver carcinoma	https://www.targetvalidation.org/target/ENSG00000150527/associations		Endoplasmic reticulum membrane	Transmembrane	No		✓
CTBP2	HUMAN C-terminal-binding protein 2	CTBP2	P56545	ENSG0000(	Others	AK290390	Prostate carcinoma, Body mass index, Age at menarche, Prostate carcinoma, Glioma, Hepatocellular carcinoma, Dermatomyositis, Lung carcinoma	https://www.targetvalidation.org/target/ENSG00000175029/associations	HIPK2 ZBP1 RBBP8 CDKN2A MECOM CTBP1 ZEB1 DVL2 CTBP1 EP300 HIPK2 KLF8 MDM2 KCNIP3 ZFPM1	Nucleus		Yes	https://www.bindingdb.org/uniprot/P56545	✓
CTCFL	HUMAN Transcriptional repressor CTCFL	BORIS, CT27, dJ579F20.2	Q8NI51	ENSG0000(	Transcription factor	DQ778124	Kallmann syndrome, Normosmic congenital hypogonadotropic hypogonadism, Li-Fraumeni syndrome, Deafness-infetility syndrome, 46-XY disorder of sex development due to isolated 17,20 lyase deficiency, Azoospermia, Ovarian carcinoma, Testicular germ cell tumor, 46-XX ovotesticular disorder of sex development	https://www.targetvalidation.org/target/ENSG00000124092/associations	SP1	Cytoplasm, Nucleus,		No		✓
CTNNA2	HUMAN Catenin alpha-2	CAPR, CTNA2	P26232	ENSG0000(	Others	AK295493	Body mass index, Femoral neck bone mineral density, Hepatocellular carcinoma, Bipolar disorder, Dysequilibrium syndrome, Walker-Warburg syndrome, Orthostatic hypotension, Revesz syndrome, Muscle-eye-brain disease, Pomtocerebellar hypoplasia type 2	https://www.targetvalidation.org/target/ENSG00000066032/associations	JUP YAP1 CTNNBIP1 ZNF639	Cell membrane		No		✓

CTNNB1	HUMAN Catenin beta-1	armadillo, CTNNB, PRO2286, OK/SW-cl.35	P35222	ENSG0000(	Cancer	AY463360	Epithelial neoplasm, Benign skin appendage neoplasm, Endometrial carcinoma, Brain disease, Colonic neoplasm, Medulloblastoma, Papillary carcinoma, Pilomatrixoma, Liver carcinoma, Melanoma, Embryonla neoplasm	https://www.targetvalidation.org/target/ENSG00000168036/associations	CSNK1G3 CBY1 AXIN2 BTRC CAMK2A FBXW11 LEF1 RABAC1 NLK CTNNBIP1 TCF7L2 CSNK1G1 CHD8 SMURF2 SOX17 PTPN23 ANAPC1 VRK1 PKP4 ERBIN DVL3 KAT2B KAT2A CREBBP PTPRU MAML1 CSNK1A1L VRK3 SIAH1	Cytoplasm		Yes	https://www.bindingdb.org/uniprot/P35222	✓
CTNNBL1	HUMAN Beta-catenin-like protein 1	C20orf33, NYD-SP19	Q8WYA6	ENSG0000(	Cancer	AK074663	Obesity, Asthma, Morbid obesity, Bone density, Psoriasis, Ovarian neoplasm, Colorectal neoplasm, Papillary carcinoma, Melanoma, Colonic neoplasm	https://www.targetvalidation.org/target/ENSG00000132792/associations	CDC5L CALCOCO1	Nucleus, Cytoplasm		No		✓
CTSD	HUMAN Cathepsin D	CLN10, CPSD	P07339	ENSG0000(	Cancer	CR456947	Neurodegenerative disease, Retinal dystrophy, Neuronal ceroid lipofuscinosis, Juvenile neuronal ceroid lipofuscinosis, Congenital neuronal ceroid lipofuscinosis, Liver carcinoma, Degenerative polyarthritis, CLN10 disease, Adult neuronal ceroid lipofuscinosis, Heart disease	https://www.targetvalidation.org/target/ENSG00000117984/associations	TP53 CSTB ESR1 FN1 CD4 CST3 APP INS IGF2R CTSB CCL20 CCL21 PSAP BGLAP SERPINA4	Lysosome, Melanosome, Secreted, extracellular space,		Yes	https://www.bindingdb.org/uniprot/P07339	✓
CTSE	HUMAN Cathepsin E	CTSE	P14091	ENSG0000(	Others	AJ250717	Adenocarcinoma, Colonic neoplasm, Adenocarcinoma, Epidermolytic palmoplantar keratoderma, Atopic eczema, Pityriasis rubra pilaris, Mal de Meleda, Hyper-IgM syndrome type 3, Erythropoietic protoporphyria, Omenn syndrome	https://www.targetvalidation.org/target/ENSG00000196188/associations	INS A2M EDN3	Endosome		Yes	https://www.bindingdb.org/uniprot/P14091	✓
CTTN	HUMAN Src substrate cortactin	EMS1, SRC8	Q14247	ENSG0000(	Transcription factor,Cancer	CH471076	46-XY complete gonadal dysgenesis, 46-XX testicular disorder of sex development, 46-XY partial gonadal dysgenesis, Breast carcinoma, Bardet-Biedl syndrome, Leukoplakia, Squamous cell carcinoma, Juvenile arthritis, Chronic granulomatous disease, Melanoma	https://www.targetvalidation.org/target/ENSG00000085733/associations	STK38L CTNND2 HDAC6 DYRK4 CDKL3 SCYL2 STYK1 DDR2 MYLK RPS6KA2 STK38 PRKD1 PTK2B CAMK2D PAK2 PAK1 TNK2 DNM1 CDK5 CDK3 ARHGAP8 ACTA1 BMX CDK7 DNM2 GSK3B PXN	Cytoplasm, cytoskeleton		No		✓
CUL4A	HUMAN Cullin-4A	CUL4A	Q13619	ENSG0000(	Others	AL136221	Male infertility with teratozoospermia due to single gene mutation, Kallmann syndrome, COFS syndrome, Cockayne syndrome, Primary ciliary dyskinesia, Stevens-Johnson syndrome, Proximal myotonic myopathy, Young syndrome, Premature aging, Hepatocellular carcinoma	https://www.targetvalidation.org/target/ENSG00000139842/associations	CSNK1A1 MAP2K4 SYK DTL CAND1 DDB1 NEDD8 RBX1 TSC2 CDKN1B HOXA9 APC CHEK1 ERCC8 TSC2 PCNA CDKN1A DDB2 DDIT4 DTL DDB1 TSC1 FBXW5 DCAF16 DCAF5 DCAF15 DCAF4 DCAF1 DCAF10 DCAF8 DCAF12 DCAF6 DCAF11			No		✓
CUL5	HUMAN Cullin-5	VACM-1	Q93034	ENSG0000(	Cancer	AK292575	HIV infection, Lung carcinoma, Breast carcinoma, Melanoma, Endometrial neoplasm, Endometrial neoplasm, Chronic lymphocytic leukemia, Glioma, Mammary neoplasm	https://www.targetvalidation.org/target/ENSG00000166266/associations	ELOB ELOC CUL3 RBX1 PRKX TGFBR1 MAPK1 APC PRKACA PRKY SOCS3 SOCS2 RBX1 ELOB ELOC RNF7 UBE2F			No		✓
CWC27	HUMAN Peptidyl-prolyl cis-trans isomerase CWC27 homolog	NY-CO-10, SDCCAG-10, SDCCAG10	Q6UX04	ENSG0000(	Others	AF039693	Bladder carcinoma, Inguinal hernia, Breast carcinoma, Ovarian carcinoma, Osteonecrosis, Brachtdactyly, Glioblastoma multiforme, Medulloblastoma, Retinal dystrophy, Melanoma	https://www.targetvalidation.org/target/ENSG00000153015/associations				No		✓
CXCR2	HUMAN C-X-C chemokine receptor type 2	CD182, CMKAR2, IL8RB	P25025	ENSG0000(	Cancer	L19593	Autoimmune disease, Diabetes mellitus, Type I diabetes mellitus, Pneumonia, Ulcerative colitis, Sepsis, Hypersensitivity, Juvenile arthritis, Mastitis, Nephritos	https://www.targetvalidation.org/target/ENSG00000180871/associations	DNAJC9 CXCL6 PPP2CA CXCL5 PPP2R1A CXCR1 CDK2 CXCL3 CXCL2 CXCL8 CXCL1 PPBP SLC9A3R1 GNAI2 GNAI3 ARRB1 CXCL8 CXCL2 GPRASP1 GTF3A IQGAP1 ST13 BRF1 LASP1 GRIA1	Cell membrane, Multi-pass membrane protein,	Transmembrane	Yes	https://www.bindingdb.org/uniprot/P25025	✓

CXCR4	HUMAN C-X-C chemokine receptor type 4	CD184, D2S201E, fusin, HM89, HSY3RR, LESTR, NPY3R, NPYR, NPYY3R	P61073	ENSG00000121966-CXCR4	Cancer, Cytokine/Chemokine	L06797	Primary immunodeficiency, WHIM syndrome, Colorectal adenocarcinoma, HIV infection, Diabetes mellitus, Mammary neoplasm, Rheumatoid arthritis, Neuralgia, Warts, Colonic neoplasm	https://www.targetvalidation.org/target/ENSG00000121966/associations	KLF2 ITCH PTPN11 PRKCD PTK2 CD164 GNAI1 PPIA USP14 JAK3 CCR5 CXCL12 GRK6 CCR2 GRK3 ARRB2 SDC4 PTPN6 MAPK1 DPP4 YY1 JAK1 VAV1 PIM1 PTPRC NPM1 CD74 CD4 CXCL14 FLOT1 JAK2 HSPA8	Cell membrane, Multi-pass membrane protein, Cell junction, Early endosome, Late endosome, Lysosome,	Transmembrane	Yes	https://www.bindingdb.org/uniprot/P61073	✓
CXCR6	HUMAN C-X-C chemokine receptor type 6	BONZO, CD186, STRL33, TYMSTR	O00574	ENSG0000(	Others	Y13248	Cervical carcinoma, Central nervous system cancer, Medulloblastoma, Familial adenomatous polyposis, Psoriasis, HIV infection, Acquired immunodeficiency syndrome, Pneumocystis jiroveci pneumonia, Kaposi sarcoma	https://www.targetvalidation.org/target/ENSG00000172215/associations	CXCL16	Cell membrane, Multi-pass membrane protein,	Transmembrane	No		✓
CXXC1	HUMAN CXXC-type zinc finger protein 1	CFP1, CGBP, hCGBP, HsT2645, PCCX1, PHF18, SPP1, ZCGPC1	Q9P0U4	ENSG0000(	Transcription factor	AK314886	Breast carcinoma, Rett syndrome, Autism, Psoriasis, Seizures, Gastric carcinoma, Autistic disorder, Osteoarthritis, Chronic lymphocytic leukemia, ICF syndrome	https://www.targetvalidation.org/target/ENSG00000154832/associations	SETD1A	Nucleus speckle		No		✓
CYCS	HUMAN Cytochrome c	CYC	P99999	ENSG0000(	Others	AL713681	Hereditary thrombocytopenia with normal platelets, Autosomal thrombocytopenia with normal platelets, Thrombocytopenia, Genetic disorder, Neoplasm, Methylmalonic acidemia, Chloracne, Brain ischemia, Transient ischemic attack, Epithelial neoplasm	https://www.targetvalidation.org/target/ENSG00000172115/associations	TP53AIP1 NLRP1 PINK1 COX4I2 BAD BAK1 CASP8 BCL2L1 BAX BID CASP9 DAP3 GSK3B VDAC2 CASP2 SNCA UQCRC1 POR HSPA8 BCL2 FDX1 CYC1 HSPB1 TP53 CYB5A APAF1 CASP9 BAX APAF1 HSPB1	Mitochondrion intermembrane space,		Yes	https://www.drugbank.ca/biodb/polypeptides/P99999	✓
CYLD	HUMAN Ubiquitin carboxyl-terminal hydrolase CYLD	CYLD1, KIAA0849, HSPC057	Q9NQC7	ENSG0000(	Cancer	AF161542	Brooke-Spiegler syndrome, Familial multiple trichoepithelioma, Familial cylindromatosis, Colonic neoplasm, Lymphoid neoplasm, Eccrine dermal cylindroma, Head and Neck neoplasia, Kidney neoplasm, Pancreatic ductal adenocarcinoma, Renal cell carcinoma	https://www.targetvalidation.org/target/ENSG00000083799/associations	CUL9 IKBKG TRAF6 TBK1 TRAIP ITCH RNF31 MIB2 MAVS TUBA1A MAPRE1 IKBKE CAMK2B SQSTM1 TRAF3 TRAF2 PLK1 AKT1 DDX58 CHUK IKBKB TRPA1 DDX58 TRAF2 MAVS IKBKG TBK1 BCL3 TRAF6 RIPK1	Cytoplasm, perinuclear region, cytoskeleton, Cell membrane, Peripheral membrane protein, Cytoplasmic side, microtubule organizing center, centrosome		No		✓
CYP1B1	HUMAN Cytochrome P450 1B1	CP1B, GLC3A, CP1B1	Q16678	ENSG0000(	Others	BT019979	Peters anomaly, Primary glaucoma, Congenital glaucoma, Glaucoma, Open-angle glaucoma, Hydrophthalmos, Juvenile glaucoma, Leukemia, Lymphoma, Acute myeloid leukemia	https://www.targetvalidation.org/target/ENSG00000138061/associations		Endoplasmic reticulum membrane, Peripheral membrane protein, Microsome membrane, Peripheral membrane protein, Mitochondrion		Yes	https://www.bindingdb.org/uniprot/Q16678	✓
DAPK1	HUMAN Death-associated protein kinase 1	DAPK	P53355	ENSG0000(	Kinase,Cancer	DQ436495	Lung carcinoma, Li-Fraumeni syndrome, Colorectal adenocarcinoma, Senior-Loken syndrome, Neurodegenerative disease, Renal cell carcinoma, Acute myelocytic leukemia, Large cell lung carcinoma, Dent disease	https://www.targetvalidation.org/target/ENSG00000196730/associations	IKBKG RPS6KA6 DAPK2 STK17A SGK494 NEO1 UNC5B MIB1 SMAD2 RPS6KA1 RPS6KA2 SMAD4 SMAD3 CALM1 RPS6KA3 TSC2 MAP1B MAPK1 MAPK3 SRC PTPRF HSP90AA1 TP53 STK17B RPS6KA4 RPS6KA5 PDCD6 DAPK3 TPM1	Cytoplasm, Cytoplasm, cytoskeleton,		Yes	https://www.bindingdb.org/uniprot/P53355	✓
DAPK2	HUMAN Death-associated protein kinase 2	DRP-1	Q9UIK4	ENSG0000(	Kinase	AC015914	Lung carcinoma, Lung adenocarcinoma, Osteosarcoma, Breast carcinoma, Non-small cell lung carcinoma, Atopic eczema, Bladder neoplasm, Gastric neoplasm, Alzheimer's disease, Cystic fibrosis	https://www.targetvalidation.org/target/ENSG00000035664/associations	CAMK2A KLF6 AURKB E2F1 CTNNB1 EPO STX1A DAPK1 MYL1 CAMKK2 TGFBR1 MLC1	Cytoplasm, Cytoplasmic vesicle, autophagosome lumen,		Yes	https://www.bindingdb.org/uniprot/Q9UIK4	✓
DBNL	HUMAN Drebrin-like protein	HIP-55, SH3P7	Q9UJU6	ENSG0000(	Others	AF151364	Severe combined immunodeficiency, Common variable immunodeficiency, Hyper-IgM syndrome with susceptibility to opportunistic infections, Agammaglobulinemia, Hodgkin's lymphoma, Bipolar disorder, Asthma, Lymphangioleiomyomatosis, Wiskott-Aldrich syndrome	https://www.targetvalidation.org/target/ENSG00000136279/associations	MAP4K1 SYK ZAP70 CDK2 IL7R MAP4K1 ZAP70 SYK	Cytoplasm, cytoskeleton		No		✓

DCAF12	HUMAN DDB1- and CUL4-associated factor 12	DCA12, KIAA1892, TCC52, WDR40A	Q5T6F0	ENSG0000(	Others	AK292935	Lung carcinoma, Small cell lung carcinoma, Breast carcinoma, Sepsis, Hepatocellular carcinoma, Cholangiocarcinoma, Obesity, Gastric carcinoma, Lung adenocarcinoma, Pancreatic carcinoma	https://www.targetvalidation.org/target/ENSG00000198876/associations	CUL4A TGFBR1 BMPR1B	Cytoplasm		No		✓
DCBID2	HUMAN Discoidin, CUB and LCCL domain-containing protein 2	CLCP1, ESDN, DCBD2	Q96PD2	ENSG0000(	Others	CH471052	Respiratory system disease, Lung carcinoma, Small cell lung carcinoma, Methylmalonic acidemia with homo cystinuria, Glycogen storage disease due to glucose 6-phosphatase deficiency, Gastric neoplasm, Polycystic ovary syndrome, Ovarian carcinoma, Colorectal cancer, Prostate carcinoma	https://www.targetvalidation.org/target/ENSG00000057019/associations		Membrane	Transmembrane	No		✓
DCDC2	HUMAN Doublecortin domain-containing protein 2	DCDC2A, KIAA1154, NPHP19, RU2	Q9UHG0	ENSG0000(	Others	AL133043	Nephronophthisis, Autosomal recessive non-syndromic sensorineural deafness type DFNB, Dyslexia, Ciliopathy, Deafness, Schizophrenia, Cholestasis, Autosomal recessive predisposition, Liver fibrosis, Breast carcinoma	https://www.targetvalidation.org/target/ENSG00000146038/associations		Cell projection, cilium		No		✓
DCLK1	HUMAN Serine/threonine-protein kinase DCLK1	DCAMKL1, DCDC3A, KIAA0369	O15075	ENSG0000(	Kinase	AK295777	Optic cup area measurement, Optic disc size measurement, Pancreatic adenocarcinoma, Glioma, Renal cell carcinoma, Bipolar disorder, Colorectal carcinoma, Breast carcinoma, Malignant mesothelioma, Walker-Warburg syndrome	https://www.targetvalidation.org/target/ENSG00000133083/associations	MAPK1 CAMKK1 PRKACB			Yes		✓
													https://www.bindingdb.org/uniprot/O15075	
DCLK2	HUMAN Serine/threonine-protein kinase DCLK2	DCAMKL2, DCDC3, DCDC3B, DCK2	Q8N568	ENSG0000(	Kinase	AC108934	Coronary artery disease, Prostate carcinoma, Myocardial infarction, Diffuse large B-cell lymphoma, Tongue squamous cell carcinoma, Autism spectrum disorder symptom, Bladder carcinoma, Medulloblastoma, Adenocarcinoma, Seizures	https://www.targetvalidation.org/target/ENSG00000170390/associations		Cytoplasm, cytoskeleton,		Yes		✓
													https://www.bindingdb.org/uniprot/Q8N568	
DCT	HUMAN L-dopachrome tautomerase	TYRP2	P40126	ENSG0000(	Others	DQ902581	Melanoma, Response to bronchodilator, Vitiligo, Li-Fraumeni syndrome, Psoriasis, Intervertebral disk displacement, Hypopigmentation disorder, Glioblastoma multiforme, Retinoblastoma	https://www.targetvalidation.org/target/ENSG00000080166/associations		Melanosome membrane	Transmembrane	No		✓
DDB1	HUMAN DNA damage-binding protein 1	DDB1	Q16531	ENSG0000(	Signalling	AJ002955	T-cell acute lymphoblastic leukemia, Melanoma, Hepatitis, Breast carcinoma, Cockayne syndrome, Gastric neoplasm, Ovarian carcinoma, Xeroderma pigmentosum, Multiple myeloma, Vitelliform macular dystrophy	https://www.targetvalidation.org/target/ENSG00000167986/associations	MAP3K3 DDB2 CUL4B CUL4A MAP3K1 PRKCZ XPC RBX1 APC PRKACA CHEK1 E2F1 CUL4A STAT1 ERCC8 XPC DDB2 DDIT4 CHEK1 RASSF1 SIRT7 CDT1 DCAF1 DCAF8 CRBN	Cytoplasm, Nucleus,		No		✓
DDB2	HUMAN DNA damage-binding protein 2	DDBB, FLJ34321, UV-DDB2, XPE	Q92466	ENSG0000(	Cancer	AB107039	Xeroderma pigmentosum, Xeroderma pigmentosum complementation group E, Bladder carcinoma, Prostate adenocarcinoma, Kidney neoplasm, Melanoma, Poikiloderma, Adenocarcinoma, Squamous cell carcinoma, Diffuse large B-cell lymphoma	https://www.targetvalidation.org/target/ENSG00000134574/associations	USP24 PAQR3 MAPK14 DDB1 CUL4B CUL4A HDAC1 XPC E2F1 RBX1 XPA XRCC5 ISG15 TP53 ABL1 E2F1 XPA XPC CDKN1A DDB1	Nucleus		No		✓

DDIT3	HUMAN DNA damage-inducible transcript 3 protein	CHOP, CHOP10, GADD153	P35638	ENSG0000(	Transcription factor,Cancer	AY880949	Sarcoma, Liposarcoma, Adenocarcinoma, Colorectal adenocarcinoma, Prostate carcinoma, Obesity, Type I diabetes mellitus, Ocular hypertension, Melanoma, Colonic neoplasm	https://www.targetvalidation.org/target/ENSG00000175197/associations	TRIB3 MAPK14 CEBPE EP300 CSNK2A1 CEBPG CEBPD CSNK2A2 ATF4 PRKCA ATF2 ATF4 BCL2 FOSL1 MAP1LC3B JUND ITGAM BCL2L11 HDAC1 CEBPB ATG5 RPS3A	Cytoplasm, Nucleus,		No		✓
DDR1	HUMAN Epithelial discoidin domain-containing receptor 1	CAK, CD167, EDDR1, NEP, NTRK4, PTK3A, RTK6, TRKE	Q08345	ENSG0000(	Others	L20817	Familial idiopathic steroid-resistant nephrotic syndrome, Gastric carcinoma, Dent disease, Primary hyperoxaluria, Alport syndrome, Schizophrenia, Lipoprotein glomerulopathy, Bipolar disorder, Cystinuria, Focal segmental glomerulosclerosis	https://www.targetvalidation.org/target/ENSG00000204580/associations	NEK6 TNK2 CDK4 TP53 COL1A1 SNAPIN RGS2 PHB2 PLCG1 SHC1 CDK2 PTPN11 CDK1 WWC1 FRS2 NCK2 TTR COL2A1 COL5A2 SNRNP40 COL3A1 BEND5 TM4SF1 COL11A1	Cell membrane, Single-pass type I membrane protein, Cell membrane, Single-pass type I membrane protein, Secreted, Cell membrane, Single-pass type I membrane protein,	Transmembrane	Yes	https://www.bindingdb.org/uniprot/Q08345	✓
DDR1_ext	HUMAN Epithelial discoidin domain-containing receptor 1	CAK, CD167, EDDR1, NEP, NTRK4, PTK3A, RTK6, TRKE	Q08345	ENSG0000(	Others	L20817	Familial idiopathic steroid-resistant nephrotic syndrome, Gastric carcinoma, Dent disease, Primary hyperoxaluria, Alport syndrome, Schizophrenia, Lipoprotein glomerulopathy, Bipolar disorder, Cystinuria, Focal segmental glomerulosclerosis	https://www.targetvalidation.org/target/ENSG00000204580/associations	NEK6 TNK2 CDK4 TP53 COL1A1 SNAPIN RGS2 PHB2 PLCG1 SHC1 CDK2 PTPN11 CDK1 WWC1 FRS2 NCK2 TTR COL2A1 COL5A2 SNRNP40 COL3A1 BEND5 TM4SF1 COL11A1	Cell membrane, Single-pass type I membrane protein, Cell membrane, Single-pass type I membrane protein, Secreted, Cell membrane, Single-pass type I membrane protein,	Transmembrane	Yes	https://www.bindingdb.org/uniprot/Q08345	✓
DDR1_int	HUMAN Epithelial discoidin domain-containing receptor 1	CAK, CD167, EDDR1, NEP, NTRK4, PTK3A, RTK6, TRKE	Q08345	ENSG0000(	Others	L20817	Familial idiopathic steroid-resistant nephrotic syndrome, Gastric carcinoma, Dent disease, Primary hyperoxaluria, Alport syndrome, Schizophrenia, Lipoprotein glomerulopathy, Bipolar disorder, Cystinuria, Focal segmental glomerulosclerosis	https://www.targetvalidation.org/target/ENSG00000204580/associations	NEK6 TNK2 CDK4 TP53 COL1A1 SNAPIN RGS2 PHB2 PLCG1 SHC1 CDK2 PTPN11 CDK1 WWC1 FRS2 NCK2 TTR COL2A1 COL5A2 SNRNP40 COL3A1 BEND5 TM4SF1 COL11A1	Cell membrane, Single-pass type I membrane protein, Cell membrane, Single-pass type I membrane protein, Secreted, Cell membrane, Single-pass type I membrane protein,	Transmembrane	Yes	https://www.bindingdb.org/uniprot/Q08345	✓
DDX43	HUMAN Probable ATP-dependent RNA helicase DDX43	CT13, DKFZp434H2114, HAGE	Q9NXZ2	ENSG0000(	Others	AC019205	Li-Fraumeni syndrome, Breast carcinoma, Azoospermia, Cystic fibrosis, Ovarian carcinoma, Candidiasis, Lung carcinoma, Leukemia, Melanoma, Uveal melanoma	https://www.targetvalidation.org/target/ENSG00000080007/associations				No		✓
DDX5	HUMAN Probable ATP-dependent RNA helicase DDX5	G17P1, HLR1, p68	P17844	ENSG0000(	Cancer,Cancer-Antigen	AK297192	Adenocarcinoma, Lung carcinoma, Esophageal carcinoma, Squamous cell carcinoma, Kidney neoplasm, Endometriosis, Chronic Hepatitis C, Liver cirrhosis, Colonic neoplasm, Bladder carcinoma	https://www.targetvalidation.org/target/ENSG00000108654/associations	RIPK3 MAP3K14 MAPK14 EP300 PRKCE MAPKAPK2 MAPK1 PRKCA PRKCB PRKCG ABL1 PIAS1 AKAP8 MAP3K7 DDX3X CALM1 TP53 SMAD3 AR HDAC1 DDX17	Nucleus, nucleolus		No		✓
DDX53	HUMAN Probable ATP-dependent RNA helicase DDX53	CAGE, CT26	Q86TM3	ENSG0000(	Others	NA	Li-Fraumeni syndrome, 3-hydroxy-1-methylpropylmercapturic acid measurement, Azoospermia, Colonic neoplasm, Breast carcinoma, Liver carcinoma, Colorectal carcinoma, Gastric neoplasm, Lung carcinoma, Adenocarcinoma	https://www.targetvalidation.org/target/ENSG00000184735/associations		Nucleus		No		✓
DDX55	HUMAN ATP-dependent RNA helicase DDX55	KIAA1595	Q8NHQ9	ENSG0000(	Others	BC035911	Body height, Breast carcinoma, Ovarian carcinoma	https://www.targetvalidation.org/target/ENSG00000111364/associations				No		✓
DEDD	HUMAN Death effector domain-containing protein	CASP8IP1, DEDD1, DEFT, FLDED1, KE05	O75618	ENSG0000(	Others	AJ010973	Breast carcinoma, Bladder carcinoma, Molluscum contagiosum, Colonic neoplasm, Diabetes mellitus, Rheumatoid arthritis, Systemic lupus erythematosus, Type II diabetes mellitus, Meningococcal infection, Kaposi's sarcoma	https://www.targetvalidation.org/target/ENSG00000158796/associations	CASP8 FADD SMAD3 CASP8	Cytoplasm, Nucleus, nucleolus		No		✓

DEK	HUMAN Protein DEK	D6S231E	P35659	ENSG0000(	Cancer	AL031774	Adenocarcinoma, Melanoma, Colonic neoplasm, Adenocarcinoma, Bile duct carcinoma, Retinoblastoma, Bladder neoplasm, Acute myelocytic leukemia, Oral squamous cell carcinoma, Prostate carcinoma	https://www.targetvalidation.org/target/ENSG00000124795/associations	AURKB CSNK2A1 HLA-A HIST1H4A; HIST1H4B; HIST1H4C; HIST1H4D; HIST1H4E; HIST1H4F; HIST1H4H; HIST1H4I; HIST1H4J; HIST1H4K; HIST1H4L; HIST2H4A; HIST2H4B; HIST4H4 SRRM1 ZIK1	Nucleus	No		✓
DFFA	HUMAN DNA fragmentation factor subunit alpha	DFF-45, DFF1, DFF45, ICAD	O00273	ENSG0000(	Others	BT006980	Stroke, Colonic neoplasm, Coronory artery disease, Polyp, Lymphoma, Hepatitis C infection, Central neuroblastoma, Breast carcinoma, Colonic neoplasm, Psoriasis	https://www.targetvalidation.org/target/ENSG00000160049/associations	CASP7 CASP3 GZMB DFFB DFFB CASP3	Cytoplasm,	No		✓
DGKA	HUMAN Diacylglycerol kinase alpha	DAGK, DAGK1, DGK-alpha	P23743	ENSG0000(	Kinase	AF064769	Male infertility with teratozoospermia due to single gene mutation, Male infertility with azoospermia or oligozoospermia due to single gene mutation, Autoimmune lymphoproliferative syndrome, Deafness-infertility syndrome, Fibrosis, Melanoma, Juvenile periodontitis, Uterine corpus cancer, Glioblastoma, Breast neoplasm	https://www.targetvalidation.org/target/ENSG00000065357/associations	SRC LCK IL21		Yes		✓
												https://www.drugbank.ca/biodb/polypeptides/P23743	
DGUOK	HUMAN Deoxyguanosine kinase, mitochondrial	dGK	Q16854	ENSG0000(	Kinase	CH471053	Mitochondrial disease, Mitochondrial myopathy, Mitochondrial DNA depletion syndrome, Portal hypertension, Adult-onset multiple mitochondrial DNA deletion syndrome due to DGUOK deficiency, Deoxyguanosine kinase deficiency, Encephalopathy	https://www.targetvalidation.org/target/ENSG00000114956/associations	PRKCA	Mitochondrion,	Yes		✓
												https://www.bindingdb.org/uniprot/Q16854	
DKK1	HUMAN Dickkopf-related protein 1	DKK-1, SK	O94907	ENSG0000(	Others	AF261158, AF261157	Breast carcinoma, Lung carcinoma, Multiple myeloma, Lymphoma, Ovarian carcinoma, Osteoporosis, Gait abnormality, Astrocytoma, Melanoma, Li-Fraumeni syndrome	https://www.targetvalidation.org/target/ENSG00000107984/associations	CTNNB1 TP53 LRP6 LRP5 CTNNB1 LRP6 LRP5 KREMEN2 KREMEN1	Secreted	Yes		✓
												https://www.bindingdb.org/uniprot/O94907	
DKKL1	HUMAN Dickkopf-like protein 1	CT34, SGY-1	Q9UK85	ENSG0000(	Others	BC030581	Multiple sclerosis, Azoospermia, Non-syndromic male infertility due to sperm motility disorder, Male infertility with azoospermia or oligozoospermia due to single gene mutation, X-linked intellectual disability-macrocephaly-macroorchidism, Deafness-infertility syndrome, Cryptorchidism, Sertoli cell-only syndrome	https://www.targetvalidation.org/target/ENSG00000104901/associations		Secreted	No		✓
DLX1	HUMAN Homeobox protein DLX-1	DLX1	P56177	ENSG0000(	Transcription factor	CH471058	Prostate carcinoma, Tongue squamous cell carcinoma, Autosomal dominant non-syndromic sensorineural deafness type DFNA, Nervous system disease, Proximal symphalangism, Schizophrenia, Bipolar disorder, Astrocytoma, Thickened earlobes - conductive deafness, Deafness-ear malformation-facial palsy	https://www.targetvalidation.org/target/ENSG00000144355/associations	SMAD4	Nucleus	No		✓
DLX3	HUMAN Homeobox protein DLX-3	DLX3	O60479	ENSG0000(	Transcription factor	CH471109	Ectodermal dysplasia syndrome, Tricho-dento-osseous syndrome, Amelogenesis imperfecta, Hypomaturation-hypoplastic amelogenesis imperfecta with taurodontism, Breast carcinoma, Colorectal adenocarcinoma, Taurodontism, Osteosclerosis, Turridolichocephaly, Li-Fraumeni syndrome	https://www.targetvalidation.org/target/ENSG00000064195/associations	PRKACA PRKCA RUNX2	Nucleus	No		✓
DLX4	HUMAN Homeobox protein DLX-4	BP1, DLX7, DLX8, DLX9	Q92988	ENSG0000(	Transcription factor	AF452638	Orofacial clefting syndrome, Ovarian carcinoma, Leukemia, Acute myeloid leukemia, Neoplasm of mature B-cells, Sarcoma, Leukemia, Thalassemia, Diabetes mellitus, AIDS	https://www.targetvalidation.org/target/ENSG00000108813/associations		Nucleus	No		✓

DMAP1	HUMAN DNA methyltransferase 1-associated protein 1	DNMAP1, DNMTAP1, EAF2, FLJ11543, KIAA1425, MEAF2, SWC4	Q9NPF5	ENSG0000(	Transcription factor	AL136657	Gastric carcinoma, Obesity, Polycystic ovary syndrome, Melanoma, Colorectal adenocarcinoma, Myotonic dystrophy	https://www.targetvalidation.org/target/ENSG00000178028/associations	TRRAP RUVBL1 RUVBL2 MORF4L1 ING3 MRGBP RNF2 RGS6 BMI1 CDK2 ACTL6A URI1 ING1 EP400 DAXX VPS72 EPC1 YEATS4 BRD8	Nucleus, Cytoplasm,		No		✓
DMPK	HUMAN Myotonin-protein kinase	DM, DM1, DM1PK, DMK, MDPK, MT-PK	Q09013	ENSG0000(	Kinase,Cancer,Cancer-Antigen	L08835	Muscular dystrophy, Myotonic dystrophy, Heart disease, Steinert myotonic dystrophy, Cardiomyopathy, DPM3-CDG, Hypogonadism, Atrial stand still, Familial isolated dilated cardiomyopathy, Alpha-crystallinopathy	https://www.targetvalidation.org/target/ENSG00000104936/associations	PLN RAF1 GABARAP RAF1 HSPB2 PPP1R12A SRF TGFB1I1 ZYX ATXN1 PLN ATN1 FXYD1 CELF1 UBQLN4	Endoplasmic reticulum membrane	Transmembrane	Yes	https://www.bindingdb.org/uniprot/Q09013	✓
DMRTB1	HUMAN Doublesex- and mab-3-related transcription factor B1	DMRTB	Q96MA1	ENSG0000(	Transcription factor	AJ291671	Li-Fraumeni syndrome, Azoospermia, Colorectal adenocarcinoma, Chronic obstructive pulmonary disease, Trypanosoma cruzi seropositivity	https://www.targetvalidation.org/target/ENSG00000143006/associations		Nucleus		No		✓
DMRTC2	HUMAN Doublesex- and mab-3-related transcription factor C2	DMRTD	Q8IXT2	ENSG0000(	Transcription factor	AJ291669	Kallmann syndrome, Male infertility with azoospermia or oligozoospermia due to single gene mutation, Normosmic congenital hypogonadotropic hypogonadism, Male infertility with teratozoospermia due to single gene mutation, Non-syndromic male infertility due to sperm motility disorder, Young syndrome, Isolated follicle stimulating hormone deficiency, Primary ovarian insufficiency, 46-XY complete gonadal dysgenesis	https://www.targetvalidation.org/target/ENSG00000142025/associations		Nucleus		No		✓
DNAJA1	HUMAN DnaJ homolog subfamily A member 1	DNAJ2, dj-2, hdj-2, HSJ2, HSPF4, DNJA1	P31689	ENSG0000(	Cancer,Cancer-Antigen	BT007292	Kallmann syndrome, Normosmic congenital hypogonadotropic hypogonadism, Male infertility with azoospermia or oligozoospermia due to single gene mutation, Melanoma, Deafness-infertility syndrome, Oculopharyngeal muscular dystrophy, Spinocerebellar ataxia type 7, Malignant pleural mesothelioma, Huntington disease, Pancreatic carcinoma	https://www.targetvalidation.org/target/ENSG00000086061/associations	RIPK3 MAP3K3 MAP3K14 RIPK1 MAP3K1 HSPA8 TXN MAP3K7	Membrane		Yes	https://www.bindingdb.org/uniprot/P31689	✓
DNAJA2	HUMAN DnaJ homolog subfamily A member 2	CPR3, DNJ3, HIRIP4	O60884	ENSG0000(	Cancer,Cancer-Antigen	AK313031	Glioblastoma multiforme, Breast carcinoma, Acute lymphoblastic leukemia, Melanoma, Medulloblastoma, Rheumatoid arthritis, Huntington disease, Ovarian carcinoma, Parkinson's disease, Psoriasis	https://www.targetvalidation.org/target/ENSG00000069345/associations	HSPA8 HSPA1A	Membrane		No		✓
DNAJB1	HUMAN DnaJ homolog subfamily B member 1	DNAJ1, HDJ1, Hsp40, HSPF1	P25685	ENSG0000(	Signalling	AK301817	Lung carcinoma, Adenocarcinoma, Liver disease, Melanoma, Breast carcinoma, Fibrolamellar carcinoma, Bipolar disorder, Fibrolamellar hepatocellular carcinoma, Schizophrenia, Herpes simplex infection	https://www.targetvalidation.org/target/ENSG00000132002/associations	MAP3K3 MAPKAPK5 HSPA4 GLB1 HSPA1A ISG15 HSF1 MDM2 HSPA4 ANK2	Cytoplasm		No		✓
DNM1L	HUMAN Dynamin-1-like protein	DRP1, DVLP, DYMPLE, HDYNIV, DLP1	O00429	ENSG0000(	Others	AF151685	Lethal encephalopathy due to mitochondrial and peroxisomal fission defect, Heart disease, Cardiomyopathy, Familial dilated cardiomyopathy, Familial isolated dilated cardiomyopathy, Lactic acidosis, Optic atrophy, Horizontal nystagmus, Hyaline body myopathy, Cardiomyopathy	https://www.targetvalidation.org/target/ENSG00000087470/associations	FIS1 MFF CAMK1 PPP3CA PRKCD MAP3K10 CDK5 PRKX GSK3B PPP3CC CDK2 PRKACA PPP3CB CDK1 PRKY FZR1 LRRK2	Cytoplasm, cytosol, Golgi apparatus, Endomembrane system, Peripheral membrane protein, Mitochondrion outer membrane		No		✓
DNM2	HUMAN Dynamin-2	CMT2M, CMTDI1, CMTDIB, DI-CMTB, DYN2	P50570	ENSG0000(	Cancer	AC007229	Genetic disorder, Muscular disease, Congenital myopathy, Ptosis, Centronuclear myopathy, Blepharoptosis, Congenital pes cavus, Charcot-Marie-Tooth disease, Autosomal dominant centronuclear myopathy, Melanoma	https://www.targetvalidation.org/target/ENSG00000079805/associations	SNX9 PPP3CA PTK2 AMPH PPP3CC KDR CDK2 NME2 PPP3CB SRC HCK CDK1 LCK ABL1 PSTPIP1 CTTN SORBS1 MYO1C GRB2 PTK2 GJB2 CCR5 KDR PFN1 VAV1 PACSIN2 SH2D3A AHI1	Cytoplasm, Cytoplasm, cytoskeleton, Cell junction		Yes	https://www.bindingdb.org/uniprot/P50570	✓

DNMT3L	HUMAN DNA (cytosine-5)-methyltransferase 3-like	DNM3L	Q9UJW3	ENSG0000(	Transcription factor	AP001060	Male infertility with azoospermia or oligozoospermia due to single gene mutation, Normosmic congenital hypogonadotropic hypogonadism, Kallmann syndrome, 46-XX testicular disorder of sex development, Partial chromosome Y deletion, Schizophrenia, Hypergonadotropic hypogonadism - cataract syndrome, 46-XY complete gonadal dysgenesis, Severe congenital hypochromic anemia with ringed sideroblasts, Female infertility due to zona pellucida defect	https://www.targetvalidation.org/target/ENSG00000142182/associations	DNMT3B HELLS HDAC1 DNMT3A	Nucleus		Yes	https://www.bindingdb.org/uniprot/Q9UJW3	✓
DNTT	HUMAN DNA nucleotidylexotransferase	TDT	P04053	ENSG0000(	Others	AK223317	Acute lymphoblastic leukemia, T-cell acute lymphoblastic leukemia, Sarcoma, Osteosarcoma, Neoplasm of mature B-cells	https://www.targetvalidation.org/target/ENSG00000107447/associations	DNTTIP1 XRCC6 PCNA PRPF19	Nucleus		Yes	https://www.bindingdb.org/uniprot/P04053	✓
DPF2	HUMAN Zinc finger protein ubi-d4	BAF45d, REQ, ubid4, REQU	Q92785	ENSG0000(	Transcription factor	AY220877	Lung carcinoma, Colorectal adenocarcinoma, Multiple myeloma, Leukemia, Astrocytoma, Tuberculosis, Steinert myotonic dystrophy, Mesothelioma, Tuberculosis	https://www.targetvalidation.org/target/ENSG00000133884/associations	RELB CDK2	Nucleus, Cytoplasm,		No		✓
DPPA2	HUMAN Developmental pluripotency-associated protein 2	CT100, PESCRG1	Q7Z7J5	ENSG0000(	Others	NA	Medulloblastoma, Renal cell carcinoma, Non-small cell lung carcinoma, Colorectal cancer, Cervical carcinoma, Azoospermia, Lung neoplasm, Multiple myeloma	https://www.targetvalidation.org/target/ENSG00000163530/associations		Nucleus		No		✓
DR1	HUMAN Protein Dr1	NC2-BETA, NC2B	Q01658	ENSG0000(	Signalling	BC002809	Glioblastoma multiforme, Atopic eczema, Ovarian carcinoma, Medulloblastoma, Psoriasis, Alzheimer's disease, Schizophrenia, Rheumatoid arthritis, Lung carcinoma, Cystic fibrosis	https://www.targetvalidation.org/target/ENSG00000117505/associations	NFIL3 DRAP1	Nucleus		No		✓
DRD2	HUMAN D(2) dopamine receptor	DRD2	P14416	ENSG0000(	Others	X51362	Mental or behavioural disorder, Schizophrenia, Neurodegenerative disease, Anorexia nervosa, Tourette syndrome, Myoclonic dystonia, Parkinson's disease, Fibromyalgia, Acromegaly, Unipolar depression	https://www.targetvalidation.org/target/ENSG00000149295/associations	CDK5 CALM1 GNAZ GNAI2 GNAI3 TRPC1 SLC6A3 PAWR SSTR5 GNAO1 ADORA2A GNB5	Cell membrane	Transmembrane	Yes	https://www.bindingdb.org/uniprot/P14416	✓
DSTYK	HUMAN Dual serine/threonine and tyrosine protein kinase	KIAA0472, RIP5, RIPK5, SGK496, HDCMD38P, DUSTY	Q6XUX3	ENSG0000(	Kinase,Cancer,Cancer-Antigen	AC093422	Genetic renal or urinary tract malformation, Melanoma, Malignant Mesothelioma, Prostate carcinoma, Hypertriglyceridemia, Micrognathism, Retrognathia, Hypoplastic mandible condyle, Psoriasis, Atopic eczema	https://www.targetvalidation.org/target/ENSG00000133059/associations		Cytoplasm		Yes	https://www.bindingdb.org/uniprot/Q6XUX3	✓
DTYMK	HUMAN Thymidylate kinase	CDC8, TMPK, TYMK, KTHY	P23919	ENSG0000(	Kinase	BC001827	Breast carcinoma, Lung carcinoma, Large cell lung carcinoma, Pancreatic carcinoma, Melanoma, Colonic neoplasm, Central neuroblastoma, HIV infection, Hepatocellular carcinoma, Acute lymphoblastic leukemia	https://www.targetvalidation.org/target/ENSG00000168393/associations				Yes	https://www.bindingdb.org/uniprot/P23919	✓
DUSP10	HUMAN Dual specificity protein phosphatase 10	MKP5, DUS10	Q9Y6W6	ENSG0000(	Kinase	AL590966	Keloid, Colorectal cancer, Response to bronchodilator, Glioma, Chronic obstructive pulmonary disease, Breast carcinoma, Progressive supranuclear palsy, Pancreatic adenocarcinoma, Melanoma, Medulloblastoma	https://www.targetvalidation.org/target/ENSG00000143507/associations	MAPK14 MAPK11 MAPK10 MAPK9 MAPK8 MTOR TGFBR1 MAPK3 MAPK8 MAPK9 MAPK14 MAPK10 MAPK11 MAPK3	Cytoplasm, Nucleus,		Yes	https://www.bindingdb.org/uniprot/Q9Y6W6	✓

DUSP11	HUMAN RNA/RNP complex-1-interacting phosphatase	DUS11, PIR1,	O75319	ENSG0000(	Others	AC092653	Glioblastoma multiforme, Lung carcinoma, Melanoma, Osteosarcoma, Contagious pustular dermatitis, Colon carcinoma, Sjogren's syndrome, Shigellosis, Gastric carcinoma, Colorectal adenocarcinoma, Diffuse large B-cell lymphoma	https://www.targetvalidation.org/target/ENSG00000144048/associations	MBP	Nucleus	No		✓
DUSP12	HUMAN Dual specificity protein phosphatase 12	YVH1, DUS12	Q9UNI6	ENSG0000(	Cancer,Cancer-Antigen	BC006286	Diabetes mellitus, Type II diabetes mellitus, Neuroblastoma, Cardiac hypertrophy, Fibrosis, Sarcoma, Polycystic ovary syndrome, Obesity, Gastric carcinoma, Chronic myelogenous leukemia	https://www.targetvalidation.org/target/ENSG00000081721/associations	GCK	Nucleus	No		✓
DUSP13	HUMAN Dual specificity protein phosphatase 13 isoform B	BEDP, DUSP13A, DUSP13B, TMDP, DS13B	Q9UII6	ENSG0000(	Others	AL392111	Neuroblastoma, Central neuroblastoma	NA	MBP		No		✓
DUSP14	HUMAN Dual specificity protein phosphatase 14	MKP-L, MKP6	O95147	ENSG0000(	Signalling, Cancer	BC001894	Tuberculosis, Colorectal cancer, Lung carcinoma, Breast carcinoma, Melanoma, Encephalomyelitis, Colonic neoplasm, Spina bifida	https://www.targetvalidation.org/target/ENSG00000276023/associations	MAPK9 MAPK14 TRAF2 MAPK3		Yes	https://www.bindingdb.org/uniprot/O95147	✓
DUSP18	HUMAN Dual specificity protein phosphatase 18	DUSP20, LMWDSP20	Q8NEJ0	ENSG0000(	Others	AK056074	Hepatocellular carcinoma, Pancreatic ductal adenocarcinoma, Sepsis, Chronic myelogenous leukemia	https://www.targetvalidation.org/target/ENSG00000167065/associations	MAPK8	Cytoplasm	No		✓
DUSP4	HUMAN Dual specificity protein phosphatase 4	HVH2, MKP-2, VH2	Q13115	ENSG0000(	Cancer	AL137704	Breast carcinoma, Ovarian carcinoma, Neoplasm of mature B-cells, Agammaglobulinemia, Cervical carcinoma, Juvenile arthritis, Depressive disorder, Hypohidrotic ectodermal dysplasia, Ulcerative colitis, Tuberculosis	https://www.targetvalidation.org/target/ENSG00000120875/associations	MAPK14 MAPK9 MAPK8 MAPK1 MAPK3 EGR1 MAPK8 MAPK1 MAPK9 MAPK14 MAPK3	Nucleus	Yes	https://www.bindingdb.org/uniprot/Q13115	✓
DUSP6	HUMAN Dual specificity protein phosphatase 6	MKP-3, PYST1	Q16828	ENSG0000(	Kinase,Signalling	AB013602	Congenital hypogonadotropic hypogonadism, Isolated congenital hypogonadotropic hypogonadism, Normosmic congenital hypogonadotropic hypogonadism, Kallmann syndrome, Breast carcinoma, Prostate carcinoma, Metastatic prostate cancer, Cystic fibrosis, Pancreatic carcinoma, Lung carcinoma	https://www.targetvalidation.org/target/ENSG00000139318/associations	MAPK6 ZBTB16 MAPK1 MAPK3 FOXO1 MAPK1 MAPK6 MAPK3	Cytoplasm	Yes	https://www.bindingdb.org/uniprot/Q16828	✓
DVL2	HUMAN Segment polarity protein dishevelled homolog DVL-2	DVL2	O14641	ENSG0000(	Signalling	CH471108	Autosomal recessive spondylocostal dysostosis, Carpenter syndrome, Robinow syndrome, Renal-hepatic-pancreatic dysplasia, Polysyndactyly - cardiac malformation, Macham syndrome, Neural tube defect, Colobomatous microphthalmia, Familial caudal dysgenesis, Matthew-Wood syndrome	https://www.targetvalidation.org/target/ENSG00000004975/associations	DAAM1 ANKRD6 FZD4 DACT1 PRICKLE1 AP2M1 RUNX2 BAMBI DAB2 PLK1 TSC2 CSNK1E ARRB1 CSNK1D RYK ARRB2 SDC4 APC CSNK2A2 PRKCA SRC PRKCB PRKCG GABARAP LRP6 PRKCB PRKCA RHOA ARRB1 ARRB2	Cell membrane	Yes	https://www.bindingdb.org/uniprot/O14641	✓
DXO	HUMAN Decapping and exoribonuclease protein	DOM3Z, DOM3L, NG6	O77932	ENSG0000(	Others	AL049547	Ovarian carcinoma, Sepsis, Alzheimer's disease, Chronic lymphocytic leukemia	https://www.targetvalidation.org/target/ENSG00000204348/associations		Nucleus	No		✓
DYNC1LI2	HUMAN Cytoplasmic dynein 1 light intermediate chain 2	DNCLI2, LIC2	O43237	ENSG0000(	Others	AC018557	Juvenile dermatomyositis, Acute quadriplegic myopathy, Breast adenocarcinoma, Pancreatic ductal adenocarcinoma, Pancreatic carcinoma	https://www.targetvalidation.org/target/ENSG00000135720/associations		Cytoplasm, cytoskeleton	No		✓
DYRK2	HUMAN Dual specificity tyrosine-phosphorylation-regulated kinase 2	DYRK2	Q92630	ENSG0000(	Kinase,Cancer	BC005809	Infant expressive language ability, Lung carcinoma, Osteosarcoma, Glioblastoma multiforme, Acute lymphoblastic leukemia, Bladder carcinoma, Breast carcinoma, Sepsis	https://www.targetvalidation.org/target/ENSG00000127334/associations	ATM MAP3K10 DPYSL2 TP53 CDK6 SIAH2 HIST3H3 JUN HSF1 EIF4EBP1 ZFP36 TBK1 MDM2 CBL STAT3 CRMP1 MAPT CAMKK1 CARHSP1 H3F3A; H3F3B RGS14 PPP1R8 CSDC2 SDC2 DPYSL3 TRIM24 DCX KATNA1 HIST2H2B	Cytoplasm, Nucleus,	Yes	https://www.bindingdb.org/uniprot/Q92630	✓

DYRK3	HUMAN Dual specificity tyrosine-phosphorylation-regulated kinase 3	REDK	O43781	ENSG0000(	Kinase	AF327561	Blackfan-Diamond anemia, Azoospermia, Congenital dyserythropoietic anemia, Congenital dyserythropoietic anemia type I, Hereditary methemoglobinemia, Anemia, Glutathione synthetase deficiency, Non-syndromic congenital cataract, Pyruvate kinase hyperactivity	https://www.targetvalidation.org/target/ENSG00000143479/associations	LIG1 XPA HCLS1 TERF2IP CREB1 AKT1S1 H3F3A; H3F3B RBM17 SFMBT1 NOC2L	Nucleus	Yes	https://www.bindingdb.org/uniprot/O43781	✓
DYRK4	HUMAN Dual specificity tyrosine-phosphorylation-regulated kinase 4	DYRK4	Q9NR20	ENSG0000(	Kinase	BC031244	Allergic rhinitis, Hepatocellular carcinoma, Sepsis, Small cell lung carcinoma, Breast carcinoma, Colorectal adenocarcinoma, Non-small cell lung carcinoma	https://www.targetvalidation.org/target/ENSG00000010219/associations	CTTN NPM1 RAD23B HCLS1 CBL CAMKK1 EIF4ENIF1 YWHAH ZYX BMX STIP1 ABI2 DGCR8 CCM2 CC2D1A CSDE1 AGGF1 SRA1 SART3 PAXBP1	Cytoplasm	Yes	https://www.bindingdb.org/uniprot/Q9NR20	✓
E2F6	HUMAN Transcription factor E2F6	E2F-6	O75461	ENSG0000(	Transcription factor	AY083997	Autosomal recessive spondylocostal dysostosis, Spondylocamptodactyly syndrome, Orofaciodigital syndrome type 11, Dilated cardiomyopathy, Breast carcinoma, Hodgkin's disease, Osteosarcoma, Lung carcinoma	https://www.targetvalidation.org/target/ENSG00000169016/associations	E2F1 CHEK1 E2F1 MAX BRCA1 BMI1 CBX5 RBBP8 UXT RING1 EZH2 TFDP1 RYBP PCGF2 TFDP2 SIN3B EPC1	Nucleus	No		✓
E6	HPV16 Protein E6	VE6	P03126	NA	Others	NA	Creutzfeldt-Jakob disease	NA	NA	Host cytoplasm, Host nucleus	Yes	https://www.bindingdb.org/uniprot/P03126	✓
E7	HPV16 Protein E7	VE7	P03129	NA	Others	AF003023	Hypertensive disease, Breast Carcinoma, Cerebral Infarction, Cerebral Ischemia	NA	NA	Host cytoplasm, Host nucleus	No		✓
EAPP	HUMAN E2F-associated phosphoprotein	BM036, C14orf11, FLJ20578	Q56P03	ENSG0000(	Others	AF217512	Glioblastoma multiforme, Atopic eczema, Breast carcinoma, Breast adenocarcinoma, Colitis, Colonic neoplasm, Ulcerative colitis, Ovarian carcinoma	https://www.targetvalidation.org/target/ENSG00000129518/associations		Cytoplasm, Nucleus,	No		✓
ECI2	HUMAN Enoyl-CoA delta isomerase 2, mitochondrial	DRS1, HCA88, PECI	O75521	ENSG0000(	Cancer,Cancer-Antigen	AL033383	Colorectal adenocarcinoma, Malignant mesothelioma, Aplastic anemia, Myeloid leukemia, Paroxysmal nocturnal hemoglobinuria, Autoimmune diabetes, Myelodysplastic syndrome, Gastric carcinoma, Omenn syndrome, Melanoma	https://www.targetvalidation.org/target/ENSG00000198721/associations		Mitochondrion, Peroxisome matrix,	Yes	https://www.drugbank.ca/biodb/polypeptides/O75521	✓
EEF1A1	HUMAN Elongation factor 1-alpha 1	EEF1A, EF1A, LENG7	P68104	ENSG0000(	Signalling, Cancer	AY043301	Hereditary spastic paraplegia, Breast carcinoma, Ovarian carcinoma, Hereditary spastic paraplegia, Pancreatic carcinoma, Mycoplasma Infection, Prostate carcinoma, Colonic neoplasm	https://www.targetvalidation.org/target/ENSG00000156508/associations	XPO5 MAP3K3 MAP3K14 PASK SMAD2 IKBKE PRKCD RPL18A PRKCE RPL24 RPL10A RPL30 RPS4X RPL7A RPS11 RPS13 RPS23 RPS14 RPS8 RPS3A ACTB RPS5 RPL5 TGFBR1 RPL4 RPL12 EEF1G EEF1B2 RPL17	Cytoplasm	Yes	https://www.bindingdb.org/uniprot/P68104	✓
EEF1A2	HUMAN Elongation factor 1-alpha 2	EEF1AL, STN, STNL	Q05639	ENSG0000(	Others	AL121829	Early infantile epileptic encephalopathy, Mental retardation, Nervous system disease, Brain disease, Epilepsy, Muscle hypotonia, Seizures, Gastric neoplasm, Renal cell carcinoma, Lung carcinoma	https://www.targetvalidation.org/target/ENSG00000101210/associations	RIPK3 MAP3K3 MAP3K1 MAPK8 MAP3K8 BRAF CHRM4 ISG15 CHUK LRRK2	Nucleus	Yes	https://www.bindingdb.org/uniprot/Q05639	✓
EEF1D	HUMAN Elongation factor 1-delta	EF-1D, NY-CO-4	P29692	ENSG0000(	Cancer,Cancer-Antigen	AC067930	Melanoma, Astrocytoma, Oligodendroglioma, Lung carcinoma, Medulloblastoma, Spinal muscular atrophy, Oral squamous cell carcinoma	https://www.targetvalidation.org/target/ENSG00000104529/associations	RIPK3 RPS6KA1 CSNK2A1 PRKAA2 EEF1G VARS EEF1B2 PRKCA TPT1 CDK1 OXSR1 STK16 PDPK1 SIAH1 KTN1 EEF1G EEF1B2	Nucleus	No		✓
EEF1G	HUMAN Elongation factor 1-gamma	EF1G, PRO1608	P26641	ENSG0000(	Cancer,Cancer-Antigen	AP001363	Adenocarcinoma, Colorectal adenocarcinoma, Lung carcinoma, Pancreatic carcinoma, Gastric carcinoma, Anaplastic large cell lymphoma	https://www.targetvalidation.org/target/ENSG00000254772/associations	TRIB3 EEF1D EEF1B2 ISG15 EEF1A1 EEF1D EEF1B2 CARS		No		✓

EEF2K	HUMAN Eukaryotic elongation factor 2 kinase	eEF-2K	O00418	ENSG0000(	Kinase	NA	Primary ovarian insufficiency, Perrault syndrome, Mayer-Rokitansky-Küster-Hauser syndrome, Kallmann syndrome, Polycystic ovary syndrome, Lethal omphalocele-cleft palate syndrome, 46-XX testicular disorder of sex development, Normosmic congenital hypogonadotropic hypogonadism, 46-XY complete gonadal dysgenesis	https://www.targetvalidation.org/target/ENSG00000103319/associations	FBXW11 RPS6KB2 TRPM7 MAPKAPK5 MAPKAPK3 MAPK14 MAPK11 RPS6KA1 RPS6KA2 PRKAA1 CALM1 PRKAA2 MAPK12 MAPKAPK2 MAPK9 MAPK8 MTOR RPS6KB1 PRKACA CDK1 MAPK13 PRKAA2 MAPK8 MAPK9 TNF MAPK14 MAPKAPK3 MAPK13			Yes	https://www.bindingdb.org/uniprot/O00418	✓
EFHD2	HUMAN EF-hand domain-containing protein D2	MGC4342	Q96C19	ENSG0000(	Others	BC014923	Melanoma, Lung carcinoma, Ovarian carcinoma, Oligodendroglioma, Colorectal cancer, Schizophrenia, Rosacea, Squamous cell carcinoma, Acute monocytic leukemia	https://www.targetvalidation.org/target/ENSG00000142634/associations	CDK2 CDK1	Membrane raft		No		✓
EFS	HUMAN Embryonal Fyn-associated substrate	CASS3, hEFS	O43281	ENSG0000(	Signalling	AK314221	Li-Fraumeni syndrome, Primary immunodeficiency, Common variable immunodeficiency, Hyper-IgM syndrome with susceptibility to opportunistic infections, Wiskott-Aldrich syndrome, Hyper-IgM syndrome type 2, Hyper-IgM syndrome type 3, Hyper-IgM syndrome type 5, Atopic eczema	https://www.targetvalidation.org/target/ENSG00000100842/associations	PTK2B PTK2 SRC FYN IWS1			No		✓
EGFR_ext	HUMAN Epidermal growth factor receptor	ERBB, ERBB1, HER1	P00533	ENSG0000(	Cancer	U48723	Epithelial neoplasm, Breast carcinoma, Digestive system disease, Thyroid disease, Head and Neck squamous cell carcinoma, Adenocarcinoma, Bronchioloalveolar adenocarcinoma, Glioblastoma, Lung adenocarcinoma, Colonic neoplasm	https://www.targetvalidation.org/target/ENSG00000146648/associations	EPN1 SNX9 CAMK2A CBLC VAV3 ERRFI1 HDAC6 SMURF2 SH3GL2 MAP3K14 GPER1 HBEGF LRIG1 AP2M1 SH3KBP1 MAP4K1 PTPRU SOCS4 PDCD6IP CMTM8 EPGN PKN3 MAPK14 PKN1 MAPK11 ERBB4 PTPRK PRKD1 PTK2B	Cell membrane, Single-pass type I membrane protein, Endoplasmic reticulum membrane, Golgi apparatus membrane, Nucleus membrane, Endosome, Endosome membrane, Nucleus,	Transmembrane	Yes	https://www.bindingdb.org/uniprot/P00533	✓
EGFR_int	HUMAN Epidermal growth factor receptor	ERBB, ERBB1, HER1	P00533	ENSG0000(	Cancer	U48723	Epithelial neoplasm, Breast carcinoma, Digestive system disease, Thyroid disease, Head and Neck squamous cell carcinoma, Adenocarcinoma, Bronchioloalveolar adenocarcinoma, Glioblastoma, Lung adenocarcinoma, Colonic neoplasm	https://www.targetvalidation.org/target/ENSG00000146648/associations	EPN1 SNX9 CAMK2A CBLC VAV3 ERRFI1 HDAC6 SMURF2 SH3GL2 MAP3K14 GPER1 HBEGF LRIG1 AP2M1 SH3KBP1 MAP4K1 PTPRU SOCS4 PDCD6IP CMTM8 EPGN PKN3 MAPK14 PKN1 MAPK11 ERBB4 PTPRK PRKD1 PTK2B	Cell membrane, Single-pass type I membrane protein, Endoplasmic reticulum membrane, Golgi apparatus membrane, Nucleus membrane, Endosome, Endosome membrane, Nucleus,	Transmembrane	Yes	https://www.bindingdb.org/uniprot/P00533	✓
EGR2	HUMAN E3 SUMO-protein ligase EGR2	KROX20	P11161	ENSG0000(	Transcription factor,Signalling	AK312813	Hereditary motor and sensory neuropathy, Charcot-Marie-Tooth disease, Charcot-Marie-Tooth disease type 1, Charcot-Marie-Tooth disease type 1D, Dejerine-Sottas syndrome, Congenital hypomyelinating neuropathy, Schizophrenia, Rett syndrome, Cockayne syndrome, Hereditary neuropathy with liability to pressure palsies	https://www.targetvalidation.org/target/ENSG00000122877/associations	WWP2 HCFC1 UBE2I	Nucleus		No		✓
EHF	HUMAN ETS homologous factor	ESE3, ESE3B, ESEJ	Q9NZC4	ENSG0000(	Transcription factor	AF212848	Li-Fraumeni syndrome, Adenocarcinoma, Colorectal adenocarcinoma, Clear cell renal carcinoma, Lung carcinoma, Abdominal Aortic aneurysm, Cystic fibrosis, Systemic lupus erythematosus	https://www.targetvalidation.org/target/ENSG00000135373/associations	ELF3	Nucleus		No		✓
EHMT2	HUMAN Histone-lysine N-methyltransferase EHMT2	BAT8, C6orf30, Em:AF134726.3, G9A, KMT1C, NG36/G9a	Q96KQ7	ENSG0000(	Cancer,Cancer-Antigen	AF134726	Breast carcinoma, Inflammation, Prostate carcinoma, Melanoma, Leukemia, Vesicular stomatitis, Oral mucositis	https://www.targetvalidation.org/target/ENSG00000204371/associations	KAT2B RICTOR CBX3 ZNF200 CBX1 PRDM1 BANF1 DNMT1 TP53 ERCC6 DNMT3B DNMT3A HDAC1 UHRF1 ACIN1 HIST1H3A; HIST1H3B; HIST1H3C; HIST1H3D; HIST1H3E; HIST1H3F; HIST1H3G; HIST1H3H; HIST1H3I; HIST1H3J HIST2H3A; HIST2H3C; HIST2H3D	Nucleus		Yes	https://www.bindingdb.org/uniprot/Q96KQ7	✓

EIF4A2	HUMAN Eukaryotic initiation factor 4A-II	DDX2B, EIF4A, EIF4F	Q14240	ENSG0000(	Cancer	CH471052	Lung carcinoma, Melanoma, Breast carcinoma, Esophageal squamous cell carcinoma, Bladder carcinoma, Schizophrenia, Gastric adenocarcinoma, Non-small cell lung carcinoma, Papillary renal cell carcinoma, Cutaneous melanoma	https://www.targetvalidation.org/target/ENSG00000156976/associations	EIF3L EIF3K EIF3M EIF3A EIF4A1 MAP2K4 EIF4E EIF3H EIF3D EIF4G3 EIF4G1 EIF4E EIF4A1 EIF4G2 PDCD4 PABPC1		No		✓
EIF4A3	HUMAN Eukaryotic initiation factor 4A-III	DDX48, EIF4AIII, KIAA0111	P38919	ENSG0000(	Others	CR456750	Richieri Costa-Pereira syndrome, Myocardial infarction, Lung adenocarcinoma, Pancreatic carcinoma, Gastric carcinoma, Pierre Robin syndrome, Microretrognathia	https://www.targetvalidation.org/target/ENSG00000141543/associations	RBM8A SMG1 UPF1 RNPS1 CDK5 MAGOH WEE1 CDK2 PRKCA CDK4 CDKL5 CASC3 SMG1 UPF1 PDCD4 RBM8A RNPS1 CASC3 MAGOH CWC22 UPF3B NOM1	Nucleus, Nucleus speckle, Cytoplasm,	No		✓
EIF4E	HUMAN Eukaryotic translation initiation factor 4E	EIF4E1, EIF4EL1, EIF4F	P06730	ENSG0000(	Signalling, Cancer	AC093836	Autism spectrum disorder, Autism, Breast carcinoma, Leukemia, Myxoid Liposarcoma, Vascular dementia, Sepsis, Myocardial ischemia, Melanoma	https://www.targetvalidation.org/target/ENSG00000151247/associations	EIF4ENIF1 MKNK2 CARD14 MKNK1 ANKHD1 MAPKAPK 5 ELAVL1 EIF4A2 HNRNPD EIF4EBP2 EIF4EBP1 EIF4G1 EIF4G2 PPP2CA EIF4A1 EIF3B WEE1 PML EIF4B VCL PRKCA PRKCB PRKCG MYC EIF4EBP3 DDX3X EIF4G3 SMAD4 EIF4G1 SMAD3	Cytoplasm, P-body	Yes	https://www.bindingdb.org/uniprot/P06730	✓
EIF4EBP1	HUMAN Eukaryotic translation initiation factor 4E-binding protein 1	4E-BP1, PHAS-I	Q13541	ENSG0000(	Signalling, Cancer	CR456769	Familial hyperinsulinism, Central nervous system cancer, Berardinelli-Seip congenital lipodystrophy, Glioblastoma multiforme, Autosomal recessive hyperinsulinism due to Kir6.2 deficiency, HIV infection, Myocardial ischemia, Obesity due to congenital leptin deficiency	https://www.targetvalidation.org/target/ENSG00000187840/associations	DYRK2 RHEBL1 RPTOR MAPKAPK 5 MAPK14 ATM CSNK2A1 PPP2CA RORC GSK3B CSNK1E CASP3 MTOR MAP3K8 AKT1 MAPK1 MAPK3 RPS6KB1 CSNK2A2 EIF4E CDK1 RPS6KA5 EIF4E2 BUB1 EIF4E RPTOR MTOR EIF4E2 LRRK2		No		✓
ELF1	HUMAN ETS-related transcription factor Elf-1	ELF1	P32519	ENSG0000(	Transcription factor	CH471075	Systemic lupus erythematosus, Inflammatory bowel disease, Crohn's disease, Astrocytoma, Glioblastoma multiforme, Biliary cirrhosis, Liver carcinoma, Hyper-IgM syndrome with susceptibility to opportunistic infection, Hyper-IgM syndrome type 3, Melanoma	https://www.targetvalidation.org/target/ENSG00000120690/associations	PPP2CA PPP2CB NFYB RB1 NFATC1 NFKB1 JUN JUND FOS	Nucleus	No		✓
ELF2	HUMAN ETS-related transcription factor Elf-2	NERF, NERF-1A, NERF-1B, NERF-2	Q15723	ENSG0000(	Transcription factor	AF256222	Lung carcinoma, Breast carcinoma, Non-small cell lung carcinoma, Melanoma, Osteosarcoma, Juvenile arthritis, Hepatocellular carcinoma, Adenocarcinoma, Leukemia, Adenoma	https://www.targetvalidation.org/target/ENSG00000109381/associations	LMO2	Nucleus	No		✓
ELF4	HUMAN ETS-related transcription factor Elf-4	ELFR, MEF	Q99607	ENSG0000(	Transcription factor,Cancer	AF256222	Adenocarcinoma, Bladder carcinoma, Lung carcinoma, Melanoma, Colorectal adenocarcinoma, Familial mediterranean fever, Lymphoma, Bile duct carcinoma, Oral squamous cell carcinoma, Clear cell renal carcinoma	https://www.targetvalidation.org/target/ENSG00000102034/associations	TBK1 RUNX1 SUMO1 SUMO2 MAPK8 PML CDK2 NPM1 RELA MAVS TBK1 TMEM173	Nucleus, PML body	No		✓
ELF5	HUMAN ETS-related transcription factor Elf-5	ELF5	Q9UKW6	ENSG0000(	Transcription factor	AF115403	Breast carcinoma, Kidney disease, Renal carcinoma, Clear cell renal carcinoma, Chronic interstitial cytitis, Asthma, Prostate carcinoma, Ovarain carcinoma, Li-Fraumeni syndrome, Colorectal cancer	https://www.targetvalidation.org/target/ENSG00000135374/associations		Nucleus	No		✓

ELK1	HUMAN ETS domain-containing protein Elk-1	ELK1	P19419	ENSG0000(	Transcription factor	AB016193	Male infertility with teratozoospermia due to single gene mutation, Non-syndromic male infertility due to sperm motility disorder, Male infertility with azoospermia or oligozoospermia due to single gene mutation, Deafness-infertility syndrome, Male infertility due to large-headed multiflagellar polyploid spermatozoa, Breast carcinoma, Lung carcinoma, Diffuse large B-cell lymphoma, Transient ischemic attack	https://www.targetvalidation.org/target/ENSG00000126767/associations	TAOK2 CREBBP MAPK14 SNCB MAPK11 PRPF4B PPP3CA MAP2K1 GRB2 MAPK10 PPP3CC MAP2K3 MAPK9 MAPK8 MAPK1 MAPK3 PPP3CB APP FOS PIAS2 STK16 RPS6KA5 PRPF4 ID2 TCF7 ID1 MAPK8 MAPK1 APP EP300 MAPK14 FOS SRF	Nucleus		No		✓
ELK3	HUMAN ETS domain-containing protein Elk-3	ERP, NET, SAP2	P41970	ENSG0000(	Transcription factor,Signalling,Cancer	CH471054	Acute lymphoblastic leukemia, B-cell acute lymphoblastic leukemia, Arrhythmogenic right ventricular dysplasia, Congenital pulmonary lymphangiectasia, Neonatal acute respiratory distress with surfactant metabolism deficiency, Mammary neoplasm, Congenital chylothorax, Bundle branch block, Glioma, Astrocytoma, Familial progressive cardiac conduction defect	https://www.targetvalidation.org/target/ENSG00000111145/associations	MAPK14 CTBP1 MAPK10 MAPK9 MAPK8 EPHB2 TCF3 SRC PIAS1 ID2	Nucleus		No		✓
ELL3	HUMAN RNA polymerase II elongation factor ELL3	FLJ22637	Q9HB65	ENSG0000(	Transcription factor	AK126384	Breast neoplasm, Breast carcinoma	NA		Nucleus		No		✓
ELMOD1	HUMAN ELMO domain-containing protein 1	ELMOD1	Q8N336	ENSG0000(	Others	CH471065	Li-Fraumeni syndrome, Central nervous system cancer, Glioma, Medulloblastoma, Renal cell carcinoma, Narcolepsy, LCAT deficiency, Ependymoma, Melanoma, Atopic eczema	https://www.targetvalidation.org/target/ENSG00000110675/associations				No		✓
ELMOD3	HUMAN ELMO domain-containing protein 3	DFNB88, RBED1, RBM29, PP4068	Q96FG2	ENSG0000(	Others	AF258573	Autosomal recessive non-syndromic sensorineural deafness type DFNB, Hearing loss, Deafness, Ductal carcinoma in situ, Invasive ductal carcinoma, Diffuse large B-cell lymphoma	https://www.targetvalidation.org/target/ENSG00000115459/associations		Cell projection, stereocilium		No		✓
ELOA	HUMAN Transcription elongation factor B polypeptide 3	SIII, TCEB3, MSTP059	Q14241	ENSG0000(	Transcription factor	CH471134	HIV infection, Psoriasis, Medeira-Dennis-Donnai syndrome, CHILD syndrome, X-linked lethal multiple pterygium syndrome, Von Hippel-Lindau syndrome, Pheochromocytoma, Hemangioblastoma, Ccockayne syndrome, Meningeal tuberculosis	https://www.targetvalidation.org/target/ENSG00000011007/associations	ELOB ELOC RAP1A	Nucleus		No		✓
ELOVL4	HUMAN Elongation of very long chain fatty acids protein 4	SCA34, STGD3	Q9GZR5	ENSG0000(	Others	AK055277	Retinal dystrophy, Autosomal dominant cerebellar ataxia, Spinocerebellar ataxia type 34, Stargardt disease, Congenital ichthyosis - intellectual disability - spastic quadriplegia, Macular degeneration, Xerosis, Dysdiadochokinesis, Papule, Retinitis pigmentosa	https://www.targetvalidation.org/target/ENSG00000118402/associations		Endoplasmic reticulum membrane	Transmembrane	Yes		✓
													https://www.drugbank.ca/biodb/polypeptides/Q9GZR5	
ENO1	HUMAN Alpha-enolase	ENO1L1, MBPB1, MPB1, ENOA	P06733	ENSG0000(	Signalling, Cancer	X84907	Asthma, Autoimmune disease, Arthritis, Lung carcinoma, Glioma, Rheumatoid arthritis, Liver carcinoma, Astrocytoma, Alzheimer's disease, Duchenne muscular dystrophy	https://www.targetvalidation.org/target/ENSG00000074800/associations	STK17A GRK5 CASP1 IL7R SRC YES1 ISG15 PLG RPS6KA4 RIPK2 MAP3K7 HDAC1 IVNS1ABP TRAPPC2	Cytoplasm		Yes		✓
													https://www.bindingdb.org/uniprot/P06733	
ENO2	HUMAN Gamma-enolase	ENO2	P09104	ENSG0000(	Signalling, Cancer	M22349	Epithelial neoplasm, Renal carcinoma, Clear cell renal carcinoma, Breast carcinoma, Alzheimer's disease, Schizophrenia, Lung carcinoma, Ovarian carcinoma, Tauopathy, Creutzfeldt Jacob disease	https://www.targetvalidation.org/target/ENSG00000111674/associations	IL7R ENO3 ENO3	Cytoplasm		Yes		✓
													https://www.bindingdb.org/uniprot/P09104	
EPAS1	HUMAN Endothelial PAS domain-containing protein 1	bHLHe73, HIF2A, HLF, MOP2, PASD2	Q99814	ENSG0000(	Transcription factor,Cancer	NA	Polycythemia, Secondary polycythemia, Autosomal dominant secondary polycythemia, Kidney neoplasm, Lung carcinoma, Melanoma, Adenocarcinoma, Esophageal squamous cell carcinoma, Renal cell carcinoma, Prostat carcinoma	https://www.targetvalidation.org/target/ENSG00000116016/associations	HIF1AN EGLN3 EGLN1 EGLN2 PRKD1 PDK1 EP300 SMAD3 VHL F12 PDPK1 SIRT1 ARNT ARNT2 DROSHA KLHL20	Nucleus		Yes		✓
													https://www.bindingdb.org/uniprot/Q99814	

EPCAM	HUMAN Epithelial cell adhesion molecule	17-1A, 323/A3, CD326, CO-17A, EGP-2, EGP34, EGP40, Ep -CAM, ESA, GA733-2, HEA125, KS1/4, KSA, Ly74, M4S1, MH99, MIC18, MK -1, MOC31, TACST-1, TACSTD1, TROP1	P16422	ENSG0000(	Cancer	M32306	Colonic neoplasm, Lynch syndrome, Epithelial neoplasm, Intestinal epithelial dysplasia, Bladder carcinoma, Hereditary nonpolyposis colorectal cancer, Gastrointestinal hemorrhage, Breast carcinoma, Large cell lung carcinoma	https://www.targetvalidation.org/target/ENSG00000119888/asso ciations	CLDN7	Lateral cell membrane	Transmem brane	Yes		✓
													https://www.drugbank.ca/biodb/polypeptides/P16422	
EPHA10	HUMAN Ephrin type -A receptor 10	EPHAA	Q5JZY3	ENSG0000(	Others	BC067734	Thyroid neoplasm, Thyroid carcinoma, Lung carcinoma, Prostate carcinoma, Pancreatic carcinoma, Non-small cell lung carcinoma, Breast carcinoma, Gastrointestinal stromal tumor, Glioblastoma multiforme, Verrucous carcinoma	https://www.targetvalidation.org/target/ENSG00000183317/asso ciations		Cell membrane	Transmem brane	No		✓
EPHA2	HUMAN Ephrin type -A receptor 2	ECK	P29317	ENSG0000(	Cancer	CH471167	Non-syndromic congenital cataract, Posterior polar cataract, Total congenital cataract, Leukemia, Thyroid carcinoma, Acute lymphoblastic leukemia, Lung carcinoma, Melanoma, Pulverulent cataract, Noonan syndrome	https://www.targetvalidation.org/target/ENSG00000142627/asso ciations	PTK2 EFNA5 EFNA4 EFNA3 AKT1 ACP1 CBL EFNA1 SRC TP53 EGFR EFNA2 EGFR SRC PIK3R2 SHC1 PTPN11 PIK3R1 GRB2 CBL PTK2 TIAM1 EFNA5 RASA1 CDH5 EFNA2 EFNA4 EFNA3 ACP1 EFNA1 TNFAIP1 CLDN4	Cell membrane	Transmem brane	Yes		✓
													https://www.bindingdb.org/uniprot/P29317	
EPHA3_ext	HUMAN Ephrin type -A receptor 3	ETK, ETK1, HEK, TYRO4	P29320			AF213460								✓
EPHB3_int	HUMAN Ephrin type -B receptor 3	ETK2, HEK2, TYRO6	P54753			BC052968								✓
EPHB4	HUMAN Ephrin type -B receptor 4	HTK, Tyro11	P54760	ENSG0000(	Cancer,Cancer-Antigen	EU826609	Thyroid neoplasm, Thyroid carcinoma, Lung carcinoma, Non-small cell lung carcinoma, Inflammatory bowel disease, Pancreatic carcinoma, Central nervous system cancer, Glioblastoma multiforme, Ulcerative colitis, Crohn's disease	https://www.targetvalidation.org/target/ENSG00000196411/asso ciations	EFNB1 EFNB2 KPNA2 GRIN1 EFNB2 SDC3	Cell membrane	Transmem brane	Yes		✓
													https://www.bindingdb.org/uniprot/P54760	
EPPIN	HUMAN Eppin	CT71, EPPIN2, SPINLW1, WAP7, WFDC7	O95925	ENSG0000(	Others	AL118493	Prostate intraepithelial neoplasia, Male infertility, Azoospermia, Hyperglycemia, Obesity, Glioblastoma multiforme	https://www.targetvalidation.org/target/ENSG00000249139/asso ciations		Secreted, Cell surface,		No		✓
EPS15	HUMAN Epidermal growth factor receptor substrate 15	AF-1P, MLLT5	P42566	ENSG0000(	Cancer	DQ367924	Listeriosis, Lymphoid neoplasm, Lung carcinoma, Kidney neoplasm, Colorectal adenocarcinoma, Amyloidosis, Squamous cell lung carcinoma, Hepatocellular carcinoma, Melanoma, Oral cavity cancer	https://www.targetvalidation.org/target/ENSG00000085832/asso ciations	EPN1 ITSN2 USP9X STON2 MAPK14 ITSN1 PTPN3 GJA1 TFAP2A EGFR AP2A1 PRKN SYNJ1 HGS EGFR DAB2 NEDD4 GRB2 UBQLN1 HGS AGFG1 EPN1 REPS2 SPG20	Cytoplasm, Cell membrane, Peripheral membrane protein, Cytoplasmic side, Membrane, clathrin-coated pit,		No		✓
ERBB2	HUMAN Receptor tyrosine-protein kinase erbB-2	CD340, HER2, NEU, NGL	P04626	ENSG0000(	Kinase,Cancer	M12036	Digestive system disease, Epithelial neoplasm, Breast carcinoma, Thyroid neoplasm, Upper aerodigestive tract neoplasm, Adenocarcinoma, Gastric carcinoma, Renal cell carcinoma, Head and Neck squamous cell carcinoma, Non-small cell lung carcinoma	https://www.targetvalidation.org/target/ENSG00000141736/asso ciations	CAMK2A PICK1 GDF15 TP53RK ERBIN LRIG1 DLG3 ELAVL1 DLG2 ERBB4 PAK1 PRKAA1 PTPN11 PTK2 NRG1 DLG4 GRB2 DUSP3 BLK PTPN9 ABL2 CSK CTNNA1 CBL ERBB3 PRKACA PRKCA FER AREG ACPP JUP CDH1 FGR	Cell membrane, Single-pass type I membrane protein, Cytoplasm, perinuclear region, Nucleus,	Transmem brane	Yes		✓
													https://www.bindingdb.org/uniprot/P04626	
ERBB3_ext	HUMAN Receptor tyrosine-protein kinase erbB-3	HER3, LCCS2	P21860	ENSG0000(	Kinase,Cancer	BT007226	Lethal congenital contracture syndrome, Lung carcinoma, Thyroid neoplasm, Renal cell carcinoma, Diabetes mellitus, Type I diabetes mellitus, Prostate carcinoma, Clear cell renal carcinoma, Non-small cell lung carcinoma, Melanoma	https://www.targetvalidation.org/target/ENSG00000065361/asso ciations	RNF41 LRIG1 ERBB4 PTK6 PAK1 PTK2 NRG1 CDK5 GRB2 NEDD4 ABL2 CSK FER FGFR1 AR HCK ERBB2 EGFR ABL1 RASA4 EREG NRG2 FGFR1 EGFR SRC PIK3R2 PLCG1 JAK2 SH2D1A CRKL SHC1	Cell membrane, Single-pass type I membrane protein, Secreted,	Transmem brane	Yes		✓
													https://www.bindingdb.org/uniprot/P21860	

ERBB3_int	HUMAN Receptor tyrosine-protein kinase erbB-3	HER3, LCCS2	P21860	ENSG0000[illegible]	Kinase,Cancer	BT007226	Lethal congenital contracture syndrome, Lung carcinoma, Thyroid neoplasm, Renal cell carcinoma, Diabetes mellitus, Type I diabetes mellitus, Prostate carcinoma, Clear cell renal carcinoma, Non-small cell lung carcinoma, Melanoma	https://www.targetvalidation.org/target/ENSG00000065361/associations	RNF41 LRIG1 ERBB4 PTK6 PAK1 PTK2 NRG1 CDK5 GRB2 NEDD4 ABL2 CSK FER FGFR1 AR HCK ERBB2 EGFR ABL1 RASA4 EREG NRG2 FGFR1 EGFR SRC PIK3R2 PLCG1 JAK2 SH2D1A CRKL SHC1	Cell membrane, Single-pass type I membrane protein, Secreted,	Transmembrane	Yes	https://www.bindingdb.org/uniprot/P21860	✓
ERCC2	HUMAN TFIIH basal transcription factor complex helicase XPD subunit	TFIIH, XPD, XPDC	P18074	ENSG0000[illegible]	Transcription factor,Cancer	AY092780	Premature aging, Xeroderma pigmentosum, Xeroderma pigmentosum-Cockayne syndrome complex, Xeroderma pigmentosum complementation group D, Trichothiodystrophy, COFS syndrome, HIV infection, Squamous cell carcinoma, Esophageal squamous cell carcinoma, Bladder carcinoma	https://www.targetvalidation.org/target/ENSG00000104884/associations	GTF2H3 GTF2H2 MNAT1 CCNH CDK7 GTF2H1 ERCC5 ERCC3 CDK1 TP53 CCNH CDK7 ERCC5 RAD51 MNAT1 ERCC3 GTF2H1 GTF2H3 GTF2H2	Nucleus		No		✓
ERCC3	HUMAN TFIIH basal transcription factor complex helicase XPB subunit	BTF2, GTF2H, RAD25, TFIIH, XPB	P19447	ENSG0000[illegible]	Transcription factor,Cancer	CH471103	Xeroderma pigmentosum, Trichothiodystrophy, Premature aging, Xeroderma pigmentosum-Cockayne syndrome complex, HIV infection, Glioma, Astrocytoma, Oral squamous cell carcinoma, Clear cell renal carcinoma, Gastric adenocarcinoma	https://www.targetvalidation.org/target/ENSG00000163161/associations	GTF2H4 GTF2H5 GTF2H3 GTF2H2 XPC CSNK2A1 RAD23B MNAT1 CCNH CDK7 GTF2H1 ERCC5 ERCC2 BCR CCNH RAD23B CDK7 ERCC5 XPC MNAT1 PSMC5 ERCC2 GTF2H1 GTF2E1 GTF2E2 GTF2H3 GTF2H2 GTF2H4	Nucleus		No		✓
ERCC5	HUMAN DNA repair protein complementing XP-G cells	ERCM2, XPGC	P28715	ENSG0000[illegible]	Cancer	AF255436	Premature aging, Xeroderma pigmentosum, Xeroderma pigmentosum-Cockayne syndrome complex, Xeroderma pigmentosum complementation group G, COFS syndrome, Cerebrooculofacioskeletal Syndrome 1, Melanoma, Deafness, Arthrogryposis, Esophageal carcinoma	https://www.targetvalidation.org/target/ENSG00000134899/associations	GTF2H4 GTF2H2 ERCC6 CDK7 GTF2H1 GADD45A ERCC3 ERCC2 EP300 CDK7 ERCC6 PCNA ERCC2 ERCC3 GTF2H1 NTHL1 GTF2H2 GTF2H4	Nucleus		Yes	https://www.bindingdb.org/uniprot/P28715	✓
ERG	HUMAN Transcriptional regulator ERG	ERG	P11308	ENSG0000[illegible]	Transcription factor,Cancer	AY204742	Sarcoma, Ewings sarcoma, Prostate carcinoma, Acute lymphoblastic leukemia, Squamous cell carcinoma, Ewings sarcoma-primitive neuroectodermal tumor, Bladder carcinoma, Pancreatic ductal adenocarcinoma, Gastric adenocarcinoma, Lung carcinoma	https://www.targetvalidation.org/target/ENSG00000157554/associations	CSNK1A1 MAPK1 HSPA8 AR LMO2	Nucleus		Yes	https://www.bindingdb.org/uniprot/P11308	✓
ESR2	HUMAN Estrogen receptor beta	ESTRB, NR3A2	Q92731	ENSG0000[illegible]	Transcription factor,Cancer	AF061054	Breast carcinoma, Digestive system disease, Osteoporosis, Schizophrenia, Unipolar depression, Mammary neoplasm, Endometriosis, Hepatocellular carcinoma, Turner syndrome, Cardiac arrhythmia	https://www.targetvalidation.org/target/ENSG00000140009/associations	NCOR2 MKNK2 NCOA7 MAPK6 NCOA1 MAPK11 NR0B2 RPS6KA1 UBE3A CALM1 AKT1 SRC RARA LCK ESR1 OXT ABL1 TRIM24 MAPK11 ESR1 RARA NR0B2 WIPI1	Nucleus		Yes	https://www.bindingdb.org/uniprot/Q92731	✓
ESRRG	HUMAN Estrogen-related receptor gamma	NR3B3, ERR3, ERRG2, KIAA0832	P62508	ENSG0000[illegible]	Transcription factor	AY388456	Response to bronchodilator, Romano-Ward syndrome, Clear cell renal carcinoma, Brugada syndrome, Nephronophthisis, Adenocarcinoma, Anoxia, Arrhythmogenic right ventricular dysplasia, Familial short QT syndrome, Atrial fibrillation	https://www.targetvalidation.org/target/ENSG00000196482/associations	NR0B2 CALM1 ESRRA PPARGC1A NCOA1	Nucleus		Yes	https://www.bindingdb.org/uniprot/P62508	✓
ETNK2	HUMAN Ethanolamine kinase 2	EKI2, HMFT1716	Q9NVF9	ENSG0000[illegible]	Kinase	AL592146	Li-Fraumeni syndrome, Periodontitis, Colorectal adenocarcinoma, Clear cell renal carcinoma, Pituitary cancer, Hypertensive disease, Breast carcinoma, Nephrosclerosis, Ductal adenocarcinoma, Isolated scaphocephaly	https://www.targetvalidation.org/target/ENSG00000143845/associations				No		✓

ETS2	HUMAN Protein C-ets-2	ETS2	P15036	ENSG0000(	Transcription factor,Signalling	BT006838	Lung carcinoma, Leukemia, Large cell lung carcinoma, Noonan syndrome, Chronic lymphocytic leukemia, LEOPARD syndrome, Craniofacial Abnormality, Down syndrome, Cardiofaciocutaneous syndrome, Psoriasis	https://www.targetvalidation.org/target/ENSG00000157557/associations	CAMK2A CDK10 MAP3K1 PLK1 ETS1 SRC JUN TP53 JUN SMARCA4 ZMYND11 ETS1 POU5F1	Nucleus,		No		✓
ETV4	HUMAN ETS translocation variant 4	E1A-F, E1AF, PEA3	P43268	ENSG0000(	Transcription factor,Cancer	AK299019	Adenocarcinoma, Prostate carcinoma, Melanoma, Ovarian serous adenocarcinoma, Colorectal adenocarcinoma, Ewings sarcoma, Esophageal adenocarcinoma, Oral squamous cell carcinoma, Hepatocellular carcinoma, Colonic neoplasm	https://www.targetvalidation.org/target/ENSG00000175832/associations	STK11 SRF EP300 SMAD2	Nucleus,		No		✓
ETV6	HUMAN Transcription factor ETV6	TEL	P41212	ENSG0000(	Transcription factor,Cancer	BC043399	Lymphoid neoplasm, Hematological system disease, Acute myeloid leukemia, Autosomal thrombocytopenia with normal platelets, Papillary thyroid carcinoma, Thrombocytopenia, Precursor cell lymphoblastic leukemia lymphoma, Adenocarcinoma, Myeloid neoplasm, Colorectal adenocarcinoma	https://www.targetvalidation.org/target/ENSG00000139083/associations	PCSK7 MAPK14 TRAF3 MAPK3 NCOR1 SIN3A FLI1 SUMO1 KAT5 UBE2I FBXL6 ETV7	Nucleus,		Yes		✓
													https://www.bindingdb.org/uniprot/P41212	
ETV7	HUMAN Transcription factor ETV7	TEL-2, TEL2	Q9Y603	ENSG0000(	Transcription factor	AF218235	Li-Fraumeni syndrome, Asthma, Hepatitis C infection, Ankylosing spondylitis, Psoriasis, Myeloproliferative disease, Multiple myeloma, Sepsis, Colorectal adenocarcinoma, Lung adenocarcinoma	https://www.targetvalidation.org/target/ENSG00000010030/associations	ETV6	Nucleus,		No		✓
EWSR1	HUMAN RNA-binding protein EWS	EWS	Q01844	ENSG0000(	Transcription factor,Cancer	CR456490	Sarcoma, Ewings sarcoma, Histiocytoma, Prostate carcinoma, Melanoma, Primitive neuroectodermal tumor, Extraskeletal myxoid chondrosarcoma, Desmoplastic small round cell tumor, Askin tumor, Head and Neck neoplasia	https://www.targetvalidation.org/target/ENSG00000182944/associations	EIF2AK4 PRMT8 SF1 PTK2B ATM BTK PRKCE FLI1 POLR2G MAPKAPK2 MATK TAF1 PRKCA SRC SNRPC PRKCB JUN PRKCG BARD1 CREBBP DHX9 POU4F1 POU5F1	Nucleus		No		✓
EXOSC10	HUMAN Exosome component 10	PM/Scl-100, PMSCL, PMSCL2, RRP6, EXOSX	Q01780	ENSG0000(	Others	AL109811	Systemic scleroderma, Myositis, Polymyositis, Breast carcinoma, Interstitial lung disease, Overlap syndrome, Werner syndrome, Dermatomyositis, Progressive supranuclear palsy, Pancreatic carcinoma	https://www.targetvalidation.org/target/ENSG00000171824/associations	MAP3K14 LRP1	Cytoplasm, Nucleus, nucleolus, Nucleus,		No		✓
EXOSC4	HUMAN Exosome complex component RRP41	p12A, RRP41, RRP41A, Rrp41p, SKI6, EXOS4	Q9NPD3	ENSG0000(	Cancer,Cancer-Antigen	BC002777	Sepsis, Diabetes mellitus, Coronary heart disease, Medulloblastoma, Diabetic nephropathy, Rectum cancer, Breast carcinoma, Metabolic syndrome	https://www.targetvalidation.org/target/ENSG00000178896/associations	NEK1 EXOSC2 TRIM24	Cytoplasm		No		✓
EXOSC8	HUMAN Exosome complex component RRP43	OIP2, RRP43, Rrp43p	Q96B26	ENSG0000(	Others	AF025438	Pontocerebellar hypoplasia type 1C, Breast carcinoma, Colorectal cancer, Melanoma, Ovarian carcinoma, Spastic tetraparesis, Proximal spinal muscular atrophy, Acute lymphoblastic leukemia	https://www.targetvalidation.org/target/ENSG00000120699/associations	EXOSC6 HTD2 NIP7 EXOSC5	Cytoplasm		No		✓
EXT1	HUMAN Exostosin-1	LGCR, LGS, ttv	Q16394	ENSG0000(	Cancer	BC001174	Congenital disorder of glycosylation, Multiple osteochondromas, Hereditary multiple exostoses, Melanoma, Lymphoid neoplasm, Chondrosarcoma, Autistic disorder, Langer-Giedion syndrome, Mental retardation	https://www.targetvalidation.org/target/ENSG00000182197/associations	EXT2	Endoplasmic reticulum membrane	Transmembrane	No		✓

EXT2	HUMAN Exostosin-2	SOTV	Q93063	ENSG0000(	Cancer	U72263	Hereditary multiple exostoses, Congenital disorder of glycosylation, Multiple osteochondromas, Gastric intestinal type adenocarcinoma, Melanoma, Cutaneous melanoma, Exostoses, Seizures, Chondrosarcoma, Chromosome 11p11.2 deletion syndrome	https://www.targetvalidation.org/target/ENSG00000151348/associations	EXT1	Endoplasmic reticulum membrane	Transmembrane	No		✓
EZH2	HUMAN Histone-lysine N-methyltransferase EZH2	ENX-1, EZH2, KMT6	Q15910	ENSG0000(	Signalling, Cancer	AK092676	Overgrowth syndrome, Weaver syndrome, Myeloid neoplasm, Colonic neoplasm, Kidney neoplasm, Lung carcinoma, Acute leukemia, Renal cell carcinoma, Breast carcinoma, Head and Neck squamous cell carcinoma	https://www.targetvalidation.org/target/ENSG00000106462/associations	BTRC HDAC2 MAPK14 SUZ12 E2F2 HDAC1 ATM RBL2 RELB E2F1 ATRX STAT3 CTNNB1 AKT1 MAPK1 CDK2 VAV1 CDK1 SNAI1 E2F6 JAK2 PHF1 BRCA1 HIST3H3 AR EED TRIM25 HSPB1 HIST1H3A; HIST1H3B; HIST1H3C; HIST1H3D;	Nucleus		Yes	https://www.bindingdb.org/uniprot/Q15910	✓
EZR	HUMAN Ezrin	VIL2, EZRI	P15311	ENSG0000(	Cancer,Cancer-Antigen	AL589931	Epithelial neoplasm, Adenocarcinoma, Lung carcinoma, Melanoma, Oral cavity cancer, Prostate carcinoma, Gastric adenocarcinoma, Leukemia, Bladder carcinoma, Cutaneous melanoma	https://www.targetvalidation.org/target/ENSG00000092820/associations	STK26 TSC1 PALLD SCYL3 LRRK2 ROCK1 FADD PTK2 PRKCQ CDK5 ACTB SYK PRKCI MAP3K8 RDX NF2 SDC2 L1CAM AKT2 MSN FAS GRK2 VCAM1 PRKACA PRKCA IL7R FER PRKAR2A SRC SP1 CAPN1 LCK ICAM1 ISG15 EGFR	Apical cell membrane		Yes	https://www.bindingdb.org/uniprot/P15311	✓
FADD	HUMAN FAS-associated death domain protein	GIG3, MORT1	Q13158	ENSG0000(	Signalling, Cancer	AK291005	FADD-related immunodeficiency, Melanoma, Lung carcinoma, Breast carcinoma, Glioma, Cerebral atrophy, Non-small cell lung carcinoma, Obesity, Hepatocellular carcinoma, Glioblastoma multiforme	https://www.targetvalidation.org/target/ENSG00000168040/associations	FAF1 PIDD1 HIPK3 ATG5 DUSP26 MYD88 CASP10 TANK MAVS TRADD CASP8 RIPK1 MAP3K1 TRAF2 PTPN13 PRKCZ CSNK2B RAP1A DAPK1 PLK1 IRAK1 DAP3 TNFSF10 CSNK1A1 FASLG STAT1 MAPK1 FAS RPS6KB1			No		✓
FAF1	HUMAN FAS-associated factor 1	CGI-03, hFAF1, HFAF1s, UBXD12, UBXN3A	Q9UNN5	ENSG0000(	Cancer,Cancer-Antigen	AJ271408	Diabetes mellitus, Type II diabetes mellitus, Ischemic stroke, Large artery stroke, Gastric carcinoma, Crohn's disease, Alzheimer's disease, Sarcoma	https://www.targetvalidation.org/target/ENSG00000185104/associations	FBXW11 NLRP3 CUL1 KRR1 FADD CSNK2A1 RAP1A VCP CTNNB1 HSPA4 MAPK3 FAS CSNK2A2 UBC AURKA IKBKB IPO5 TRPV1 CSNK2A1 RELA FAS FADD SKP1 NLRP3 CSNK2B CASP8	Nucleus		No		✓
FAIM	HUMAN Fas apoptotic inhibitory molecule 1	FAIM1	Q9NVQ4	ENSG0000(	Others	AC020890	Inborn errors of metabolism, Progressive familial intrahepatic cholestasis, Breast carcinoma, Hyper-IgM syndrome with susceptibility to opportunistic infections, Tyrosinemia type 1, ALG8-CDG, Congenital bile acid synthesis defect type 4, Erythropoietic protoporphyria, Melanoma, Phosphoenolpyruvate carboxykinase deficiency	https://www.targetvalidation.org/target/ENSG00000158234/associations		Cytoplasm		No		✓
FAM133A	HUMAN Protein FAM133A	F133A	Q8N9E0	ENSG0000(	Others	BC113385	Tongue squamous cell carcinoma, Hepatocellular carcinoma, Breast carcinoma, Central nervous system cancer, Ovarian carcinoma, Medulloblastoma, Lung carcinoma, Lung adenocarcinoma	https://www.targetvalidation.org/target/ENSG00000179083/associations				No		✓
FAM46D	HUMAN Protein FAM46D	TENT5D	Q8NEK8	ENSG0000(	Others	AL591431	Li-Fraumeni syndrome, Azoospermia, Multiple myeloma	https://www.targetvalidation.org/target/ENSG00000174016/associations				No		✓
FAM50B	HUMAN Protein FAM50B	X5L	Q9Y247	ENSG0000(	Cancer,Cancer-Antigen	CH471087	Response to bronchodilator, FEV/FEC ratio, Forced expiratory volume, Colorectal adenocarcinoma, Astrocytoma, Seminoma, Glioblastoma multiforme, Germ cell tumor, Pancreatic adenocarcinoma, Oligodendroglioma	https://www.targetvalidation.org/target/ENSG00000145945/associations				No		✓

FANCC	HUMAN Fanconi anemia group C protein	FA3, FAC, FACC	Q00597	ENSG0000(	Cancer	AY220878	Inherited cancer-predisposing syndrome, Fanconi anemia, Melanoma, Lung carcinoma, Pancytopenia, Acquired scoliosis, Colorectal adenocarcinoma, Acute myeloid leukemia, Oral squamous cell carcinoma, Squamous cell carcinoma	https://www.targetvalidation.org/target/ENSG00000158169/associations	FANCL FANCF FANCE FANCD2 CENPS FANCM BLM POR HSP90B1 GSTP1 HSP90AA1 CDK1 TP53 FANCA FANCG BLM FANCM FANCL STAT1 EIF2AK2 FANCE FANCG FANCD2 FANCA FANCF FAAP20 ZBTB32	Nucleus, Cytoplasm,		No		✓
FAP	HUMAN Prolyl endopeptidase FAP	SEPR	Q12884	ENSG0000(	Cancer	AC007750	Epithelial neoplasm, Prostate carcinoma, Proriasis, Vitiligo, Inflammatory bowel disease, Adenomatous polyposis coli, Colorectal cancer, Ulcerative colitis, Malignant mesothelioma, Lung carcinoma	https://www.targetvalidation.org/target/ENSG00000078098/associations	PLAUR DPP4	Prolyl endopeptidase FAP, Cell surface	Transmembrane	Yes	https://www.bindingdb.org/uniprot/Q12884	✓
FAS	HUMAN Tumor necrosis factor receptor superfamily member 6	APO-1, APT1, CD95, FAS1, TNFRSF6, TNR6	P25445	ENSG0000(	Cancer	X83491	Immune system disease, Primary immunodeficiency, Autoimmune lymphoproliferative syndrome, Melanoma, Lymphoid neoplasm, Retinal detachment, Galucoma, Acute myelocytic leukemia, Breast ductal adenocarcinoma, Cutaneous melanoma	https://www.targetvalidation.org/target/ENSG00000026103/associations	FAF1 DAXX BABAM2 HIPK3 MAP3K5 CASP10 CASP8 RIPK1 FADD PTPN13 BTK RAP1A BMX FASLG STAT1 TTK AKT1 YY1 LGALS3 PRKCA EZR LGALS1 MET FYN LCK TP53 EGFR NOL3 CFLAR TP73 PTPN13 FAF1	Cell membrane, Single-pass type I membrane protein, Secreted, Secreted, Secreted, Secreted, Secreted,	Transmembrane	No		✓
FASTK	HUMAN Fas-activated serine/threonine kinase	FAST	Q14296	ENSG0000(	Kinase	AC010973	Agammaglobulinemia, Isolated agammaglobulinemia, Hyper-IgM syndrome with susceptibility to opportunistic infections, Autosomal dominant severe congenital neutropenia, Autosomal agammaglobulinemia, Alpha-Thalassemia, Chronic granulomatous disease, WHIM syndrome, Transcobalamin deficiency	https://www.targetvalidation.org/target/ENSG00000164896/associations	MDFI TIA1 CBL PITX1 CALCOCO2 WWP1 SF1 RBPMS	Mitochondrion matrix		No		✓
FASTKD1	HUMAN FAST kinase domain-containing protein 1, mitochondrial	KIAA1800	Q53R41	ENSG0000(	Kinase	AC093899	Breast carcinoma, Breast adenocarcinoma, Non-small cell lung carcinoma, Melanoma, Nasopharyngeal neoplasm, Glioblastoma multiforme, Endometrial neoplasm, Juvenile dermatomyositis, Acute myeloid leukemia, Prostate adenocarcinoma	https://www.targetvalidation.org/target/ENSG00000138399/associations		Mitochondrion		No		✓
FBXO9	HUMAN F-box only protein 9	FBX9, NY-REN-57, VCIA1	Q9UK97	ENSG0000(	Cancer,Cancer-Antigen	AK021559	Coronary artery disease - hyperlipidemia - hypertension - diabetes - osteoporosis, Large cell lung carcinoma, Familial hypercholesterolemia, Cholesterol-ester transfer protein deficiency, LCAT deficiency, Homozygous familial hypercholesterolemia, Johanson-Blizzard syndrome, Hyperlipoproteinemia type 5, Niemann-Pick disease type B, Acute lymphoblastic leukemia	https://www.targetvalidation.org/target/ENSG00000112146/associations		Cytoplasm		No		✓
FEN1	HUMAN Flap endonuclease 1	FEN-1, MF1, RAD2	P39748	ENSG0000(	Signalling, Cancer	AC004770	Phospholipid measurement, Fatty acid measurement, Trans fatty acid measurement, Inflammatory bowel disease, Platelet count, Huntington disease, Familial primary pulmonary hypoplasia, Lethal Larsen-like syndrome, Pancreatic carcinoma, Breast carcinoma	https://www.targetvalidation.org/target/ENSG00000168496/associations	WRN EP300 BLM CDKN1A RPA1 APC CDK2 CCNA2 PCNA HNRNPA1 CDK1 RECQL4 RAD1 CHEK1 PRMT5 MUS81 APEX1 PCNA	Nucleus, nucleolus, Nucleus, nucleoplasm,		Yes	https://www.bindingdb.org/uniprot/P39748	✓
FES	HUMAN Tyrosine-protein kinase Fes/Fps	FPS	P07332	ENSG0000(	Kinase,Cancer	AY513656	Melanoma, Kidney neoplasm, Cutaneous melanoma, Bladder carcinoma, Prostate carcinoma, Myeloid leukemia, Sarcoma, Colorectal neoplasm, Gastric adenocarcinoma, Lymphoid neoplasm	https://www.targetvalidation.org/target/ENSG00000182511/associations	DOK1 JAK3 CSF2RB PIK3R1 JAK1 EZR BCR EGFR JAK2 JAK1 BCR TRIM28 IRS2 IRS1 GAB1 IL4R BCAR1 PIK3R1 STAT3 JAK3 RASA1 CSF2RB DOK1 NSF ACHE AGA KRTAP4-12 DPYSL5 RASA3 PLXNA1 HSH2D	Cytoplasm, cytosol, cytoskeleton, Cell membrane, Peripheral membrane protein, Cytoplasmic side, Cytoplasmic vesicle, Golgi apparatus, Cell junction, focal adhesion,		Yes	https://www.bindingdb.org/uniprot/P07332	✓

FGF1	HUMAN Fibroblast growth factor 1	AFGF, ECGF, ECGF-beta, ECGFA, ECGFB, FGF-alpha, FGFA, GLIO703, HBGF1	P05230	ENSG0000(	Kinase,Signalling,Cancer, Cytokine/Chemokine	X51943	Endometrial neoplasm, Acrocephalosyndactylia, Epithelial neoplasm, Hepatocellular carcinoma, Acrophalosyndactylia, Ovarian neoplasm, Mesothelioma, Clear cell renal carcinoma, Renal cell carcinoma, Tongue squamous cell carcinoma	https://www.targetvalidation.org/target/ENSG00000113578/associations	FGFBP1 CSNK2A1 FGFR3 FGFR4 FGFR2 CSNK2A2 FGFR1 FIBP FGFR1 FGFR2 FGFR3 HSPA9 FGFR4	Secreted, Cytoplasm, cell cortex, cytosol, Nucleus,		Yes	https://www.bindingdb.org/uniprot/P05230	✓
FGF13	HUMAN Fibroblast growth factor 13	FGF2, FHF2	Q92913	ENSG0000(	Kinase,Signalling	AK297545	Astrocytoma, Glioblastoma multiforme, Ependymoma, Oligodendroglioma, Medulloblastoma, Pancreatic neoplasm, Alzheimer's disease, Central neuroblastoma, Epilepsy, Borjeson-Forssman-Lehmann syndrome	https://www.targetvalidation.org/target/ENSG00000129682/associations	SCN3A SCN2A SCN1A SCN4A SCN5A SCN11A SCN9A SCN10A SCN8A	Cell projection, filopodium		No		✓
FGFR1	HUMAN Fibroblast growth factor receptor 1	BFGFR, CD331, CEK, FLG, FLT2, FGFBR, HBGFR	P11362	ENSG0000(	Cancer	M34186	Osteoglophonic dwarfism, Depressive disorder, Kallmann syndrome, Cleft lip, Cleft palate, Septo-optic dysplasia, Interfrontal craniofaciosynostosis, Encephalocraniocutaneous lipomatosis, Congenital hypogonadotropic hypogonadism, Syndromic craniosynostosis	https://www.targetvalidation.org/target/ENSG00000077782/associations	FGF23 FRS2 MAPK14 SHB RPS6KA1 SOS1 PRKCE E2F1 GRB2 FGF8 EPHA4 BMX RPS6KA3 CTNNB1 FGF9 MAPK1 MAPK3 ERBB3 FGF7 NCAM1 FGF5 FGF3 FGF6 GZMB FGF2 HTR1A FGF4 MMP2 FGF1	Cell membrane, Single-pass type I membrane protein, Nucleus, Cytoplasm, cytosol, Cytoplasmic vesicle,	Transmembrane	Yes	https://www.bindingdb.org/uniprot/P11362	✓
FGFR1_ext	HUMAN Fibroblast growth factor receptor 1	BFGFR, CD331, CEK, FLG, FLT2, FGFBR, HBGFR	P11362	ENSG0000(	Cancer	M34186	Osteoglophonic dwarfism, Depressive disorder, Kallmann syndrome, Cleft lip, Cleft palate, Septo-optic dysplasia, Interfrontal craniofaciosynostosis, Encephalocraniocutaneous lipomatosis, Congenital hypogonadotropic hypogonadism, Syndromic craniosynostosis	https://www.targetvalidation.org/target/ENSG00000077782/associations	FGF23 FRS2 MAPK14 SHB RPS6KA1 SOS1 PRKCE E2F1 GRB2 FGF8 EPHA4 BMX RPS6KA3 CTNNB1 FGF9 MAPK1 MAPK3 ERBB3 FGF7 NCAM1 FGF5 FGF3 FGF6 GZMB FGF2 HTR1A FGF4 MMP2 FGF1	Cell membrane, Single-pass type I membrane protein, Nucleus, Cytoplasm, cytosol, Cytoplasmic vesicle,	Transmembrane	Yes	https://www.bindingdb.org/uniprot/P11362	✓
FGFR1_int	HUMAN Fibroblast growth factor receptor 1	BFGFR, CD331, CEK, FLG, FLT2, FGFBR, HBGFR	P11362	ENSG0000(	Cancer	M34186	Osteoglophonic dwarfism, Depressive disorder, Kallmann syndrome, Cleft lip, Cleft palate, Septo-optic dysplasia, Interfrontal craniofaciosynostosis, Encephalocraniocutaneous lipomatosis, Congenital hypogonadotropic hypogonadism, Syndromic craniosynostosis	https://www.targetvalidation.org/target/ENSG00000077782/associations	FGF23 FRS2 MAPK14 SHB RPS6KA1 SOS1 PRKCE E2F1 GRB2 FGF8 EPHA4 BMX RPS6KA3 CTNNB1 FGF9 MAPK1 MAPK3 ERBB3 FGF7 NCAM1 FGF5 FGF3 FGF6 GZMB FGF2 HTR1A FGF4 MMP2 FGF1	Cell membrane, Single-pass type I membrane protein, Nucleus, Cytoplasm, cytosol, Cytoplasmic vesicle,	Transmembrane	Yes	https://www.bindingdb.org/uniprot/P11362	✓
FGFR1OP	HUMAN FGFR1 oncogene partner	FOP, FR1OP	O95684	ENSG0000(	Cancer	Z94721	Myeloid neoplasm, Leukemia, Acute lymphoblastic leukemia, Myelodysplastic syndrome, Melanoma, Lung neoplasm, Vitiligo, Ulcerative colitis, Crohn's disease, Colorectal adenocarcinoma	https://www.targetvalidation.org/target/ENSG00000213066/associations	PCM1 PPP2R1A BMPR1B MAPRE1 CEP290 OFD1 CEP135	Cytoplasm, cytoskeleton, microtubule organizing center, centrosome		No		✓
FGFR2	HUMAN Fibroblast growth factor receptor 2	BEK, CD332, KSAM, KGFR	P21802	ENSG0000(	Cancer, Kinase	M35718	Ovarian neoplasm, Craniosynostosis, Colonic neoplasm, Endometrial neoplasm, Bent bone dysplasia, Jackson-Weiss syndrome, Autosomal dominant Antley-Bixler syndrome, Lacrimoauriculodentodigital syndrome, Breast carcinoma, Lung carcinoma	https://www.targetvalidation.org/target/ENSG00000066468/associations	FGF22 FGF23 PRKCE GRB2 PTEN FGF8 EPHA4 DUSP3 NEDD4 FGF9 CBL FGF7 SRC FGF5 FGF3 FGF6 FGF2 FGF4 FGF1 PAK4 FGF18 FGF17 FGF10 HGS PTEN CTTN SRC PLCG1 TCF3 FGF8 SHC1 FGF2 CRK YWHAZ	Cell membrane, Single-pass type I membrane protein, Golgi apparatus, Cytoplasmic vesicle,	Transmembrane	Yes	https://www.bindingdb.org/uniprot/P21802	✓
FGFR2_ext	HUMAN Fibroblast growth factor receptor 2	BEK, CD332, KSAM, KGFR	P21802	ENSG0000(	Cancer, Kinase	M35718	Ovarian neoplasm, Craniosynostosis, Colonic neoplasm, Endometrial neoplasm, Bent bone dysplasia, Jackson-Weiss syndrome, Autosomal dominant Antley-Bixler syndrome, Lacrimoauriculodentodigital syndrome, Breast carcinoma, Lung carcinoma	https://www.targetvalidation.org/target/ENSG00000066468/associations	FGF22 FGF23 PRKCE GRB2 PTEN FGF8 EPHA4 DUSP3 NEDD4 FGF9 CBL FGF7 SRC FGF5 FGF3 FGF6 FGF2 FGF4 FGF1 PAK4 FGF18 FGF17 FGF10 HGS PTEN CTTN SRC PLCG1 TCF3 FGF8 SHC1 FGF2 CRK YWHAZ	Cell membrane, Single-pass type I membrane protein, Golgi apparatus, Cytoplasmic vesicle,	Transmembrane	Yes	https://www.bindingdb.org/uniprot/P21802	✓

FGFR2_int	HUMAN Fibroblast growth factor receptor 2	BEK, CD332, KSAM, KGFR	P21802	ENSG0000(	Cancer, Kinase	M35718	Ovarian neoplasm, Craniosynostosis, Colonic neoplasm, Endometrial neoplasm, Bent bone dysplasia, Jackson-Weiss syndrome, Autosomal dominant Antley-Bixler syndrome, Lacrimoauriculodentodigital syndrome, Breast carcinoma, Lung carcinoma	https://www.targetvalidation.org/target/ENSG00000066468/associations	FGF22<br>FGF23<br>PRKCE<br>GRB2<br>PTEN<br>FGF8<br>EPHA4<br>DUSP3<br>NEDD4<br>FGF9 CBL<br>FGF7 SRC<br>FGF5<br>FGF3<br>FGF6<br>FGF2<br>FGF4<br>FGF1<br>PAK4<br>FGF18<br>FGF17<br>FGF10<br>HGS PTEN<br>CTTN SRC<br>PLCG1<br>TCF3<br>FGF8<br>SHC1<br>FGF2 CRK<br>YWHAZ	Cell membrane, Single-pass type I membrane protein, Golgi apparatus, Cytoplasmic vesicle,	Transmembrane	Yes	https://www.bindingdb.org/uniprot/P21802	✓
FGFR4_ext	HUMAN Fibroblast growth factor receptor 4	CD334, JTK2	P22455	ENSG0000(	Cancer	Y13901	Prostate carcinoma, Prostate adenocarcinoma, Hepatocellular carcinoma, Familial prostate cancer, Rhabdomyosarcoma, Adult respiratory distress syndrome, Adenoid cystic carcinoma, Idiopathic pulmonary fibrosis, Non-small cell lung carcinoma, Sarcoma	https://www.targetvalidation.org/target/ENSG00000160867/associations	FGF8<br>EPHA4<br>FGF9 CBL<br>FGF7<br>CDH2<br>FGF5<br>FGF3<br>FGF6<br>FGF2<br>FGF4<br>FGF1<br>FGF19<br>FGF18<br>FGF17<br>CTTN<br>PLCG1<br>MAPK8<br>FGF8<br>FGF2<br>STAT1<br>HCLS1<br>RPS6KA3<br>CBL<br>PPP1R13L<br>STAT3<br>CDH2<br>FGF3<br>FGF9<br>FGF1 BMX	Cell membrane, Single-pass type I membrane protein, Endosome, Endoplasmic reticulum,	Transmembrane	Yes	https://www.bindingdb.org/uniprot/P22455	✓
FGFR4_int	HUMAN Fibroblast growth factor receptor 4	CD334, JTK2	P22455	ENSG0000(	Cancer	Y13901	Prostate carcinoma, Prostate adenocarcinoma, Hepatocellular carcinoma, Familial prostate cancer, Rhabdomyosarcoma, Adult respiratory distress syndrome, Adenoid cystic carcinoma, Idiopathic pulmonary fibrosis, Non-small cell lung carcinoma, Sarcoma	https://www.targetvalidation.org/target/ENSG00000160867/associations	FGF8<br>EPHA4<br>FGF9 CBL<br>FGF7<br>CDH2<br>FGF5<br>FGF3<br>FGF6<br>FGF2<br>FGF4<br>FGF1<br>FGF19<br>FGF18<br>FGF17<br>CTTN<br>PLCG1<br>MAPK8<br>FGF8<br>FGF2<br>STAT1<br>HCLS1<br>RPS6KA3<br>CBL<br>PPP1R13L<br>STAT3<br>CDH2<br>FGF3<br>FGF9<br>FGF1 BMX	Cell membrane, Single-pass type I membrane protein, Endosome, Endoplasmic reticulum,	Transmembrane	Yes	https://www.bindingdb.org/uniprot/P22455	✓
FH	HUMAN Fumarate hydratase, mitochondrial	FUMH	P07954	ENSG0000(	Cancer	AK312415	Fumaric aciduria, Inherited cancer-predisposing syndrome, Melanoma, Kidney neoplasm, Hereditary leiomyomatosis, Fumarase deficiency, Cutaneous leiomyoma, Polycythemia, Muscle hypotonia, Polymicrogyria	https://www.targetvalidation.org/target/ENSG00000091483/associations	PRKDC C3<br>MDH2	Mitochondrion, Cytoplasm,		No		✓
FIP1L1	HUMAN Pre-mRNA 3'-end-processing factor FIP1	FIP1, RHE	Q6UN15	ENSG0000(	Cancer	AC058822	Colorectal adenocarcinoma, Melanoma, Breast carcinoma, Esophageal squamous cell carcinoma, Sarcoma, Hypereosinophilic syndrome, Chronic eosinophilic leukemia, Prostate carcinoma, Clear cell renal carcinoma, Lung adenocarcinoma	https://www.targetvalidation.org/target/ENSG00000145216/associations	STK17A<br>SGK3<br>REG3A<br>AKT1<br>PRKACA<br>SRC<br>PAPOLA	Nucleus		No		✓
FKBP1B	HUMAN Peptidyl-prolyl cis-trans isomerase FKBP1B	FKBP12.6, FKBP1L, FKBP9, OTK4, FKB1B	P68106	ENSG0000(	Signalling	S69800	Familial atrial fibrillation, Cardiomyopathy, Cardiac arrhythmia, Arrhythmogenic right ventricular dysplasia, Atrial conduction disease, Genetic cardiac anomaly, Left ventricular noncompaction, Brugada syndrome, Atypical progressive supranuclear palsy, Prostate carcinoma	https://www.targetvalidation.org/target/ENSG00000119782/associations	TGFBR1<br>RYR2	Cytoplasm		Yes	https://www.bindingdb.org/uniprot/P68106	✓
FKBP3	HUMAN Peptidyl-prolyl cis-trans isomerase FKBP3	FKBP-25, PPIase	Q00688	ENSG0000(	Cancer,Cancer-Antigen	BT006904	Breast carcinoma, Ovarian carcinoma, Teratozoospermia, Acute quadriplegic myopathy, Fibrosis, Lymphoma, Phyllodes tumor, Pulmonary mucoepidermoid carcinoma, Malaria	https://www.targetvalidation.org/target/ENSG00000100442/associations	CSNK2A1<br>CSNK2A2	Nucleus		Yes	https://www.bindingdb.org/uniprot/Q00688	✓
FKBP5	HUMAN Peptidyl-prolyl cis-trans isomerase FKBP5	FKBP51, FKBP54, P54, PPIase, Ptg-10	Q13451	ENSG0000(	Cancer	AB209893	Li-Fraumeni syndrome, Lung carcinoma, Large cell lung carcinoma, Breat carcinoma, Glioma, Unipolar depression, Bipolar disorder, Melanoma, Astrocytoma, Sepsis	https://www.targetvalidation.org/target/ENSG00000096060/associations	IKBKE<br>MAP3K1<br>IL15 AR<br>HSP90AA1<br>MAP3K7<br>CHUK<br>IKBKB<br>MAP3K7<br>MAP3K1<br>NR3C1<br>IKBKE	Cytoplasm, Nucleus		Yes	https://www.bindingdb.org/uniprot/Q13451	✓

FLCN	HUMAN Folliculin	BHD	Q8NFG4	ENSG00000	Cancer	AC055811	Inherited cancer-predisposing syndrome, Birt-Hogg-Dubé syndrome, Fibrofolliculoma, Renal carcinoma, Colorectal adenocarcinoma, Familial spontaneous pneumothorax, Renal cell carcinoma, Hepatocellular carcinoma, Melanoma, Breast carcinoma	https://www.targetvalidation.org/target/ENSG00000154803/associations	PRKAA1	Cytoplasm		No		✓
FLI1	HUMAN Friend leukemia integration 1 transcription factor	EWSR2, SIC-1	Q01543	ENSG00000	Kinase,Transcription factor,Cancer	M93255	Sarcoma, Ewings sarcoma, Melanoma, Kidney neoplasm, Lung carcinoma, Squamous cell lung carcinoma, Lymphoma, Prostate carcinoma, Colorectal adenocarcinoma, Kidney neoplasm	https://www.targetvalidation.org/target/ENSG00000151702/associations	HDAC1 KLF1 EP300 PRKCD ETV6 TGFBR2 GATA1 EWSR1 BCL2 KAT2B KLF1	Nucleus		No		✓
FLT1	HUMAN Vascular endothelial growth factor receptor 1	FLT, FRT, VEGFR1, VGFR1	P17948	ENSG00000	Kinase,Cancer	EU826561	Renal carcinoma, Colorectal cancer, Thyroid carcinoma, Idiopathic pulmonary fibrosis, Clear cell renal carcinoma, Liver cirrhosis, Cerebral hemorrhage, Gastrointestinal stromal tumor, Ovarian neoplasm, Pancreatic carcinoma	https://www.targetvalidation.org/target/ENSG00000102755/associations	ATR PTPRJ PTPN11 PTK2 VEGFB PGF CSNK1A1 NEDD4 STAT1 STAT3 KDR CBL VEGFA YES1 CTNNB1 PLCG2 FYN PLCG1 SHC1 STAT1 CRK EGR1 PTPN11 PIK3R1 GRB2 STAT3 PTK2 NCK1 KDR VEGFA PTPRB VEGFB	Cell membrane, Single-pass type I membrane protein, Endosome,	Transmembrane	Yes	https://www.bindingdb.org/uniprot/P17948	✓
FMNL2	HUMAN Formin-like protein 2	FHOD2, KIAA1902	Q96PY5	ENSG00000	Transcription factor	AC093794	Crigler-Najjar syndrome, Colorectal adenocarcinoma, Asthma, Genetic disorder, Metabolic disease, Autistic disorder, Hereditary fructose intolerance, Omodysplasia, Acheiropodia, Richieri Costa-Pereira syndrome, Progressive familial intrahepatic cholestasis	https://www.targetvalidation.org/target/ENSG00000157827/associations	PRKCA	Cytoplasm		No		✓
FMR1NB	HUMAN Fragile X mental retardation 1 neighbor protein	CT37, NY-SAR-35	Q8N0W7	ENSG00000	Others	CH471171	Li-Fraumeni syndrome, Anti-neutrophil antibody associated vasculitis, Azoospermia, Colorectal adenocarcinoma, Medulloblastoma, Pancreatic ductal adenocarcinoma	https://www.targetvalidation.org/target/ENSG00000176988/associations		Membrane	Transmembrane	No		✓
FN3K	HUMAN Fructosamine-3-kinase	FN3K	Q9H479	ENSG00000	Kinase	NA	Kidney disease, Kidney stone, Diabetes mellitus, Colonic neoplasm, Endothelial dysfunction, Type II diabetes mellitus, Colorectal cancer, Breast carcinoma, Stroke, Human Herpesvirus 8 infection	https://www.targetvalidation.org/target/ENSG00000167363/associations				No		✓
FOLH1	HUMAN Glutamate carboxypeptidase 2	FOLH, FGCP, GCPII, NAALAD1, NAALAdase1, PSM, PSMA, GIG27	Q04609	ENSG00000	Signalling, Cancer	AF176574	Inflammatory bowel disease, Crohn's disease, Hyperplasia, Prostate carcinoma, Lung carcinoma, Schizophrenia, Colorectal cancer, Neural tube defect, Hepatocellular carcinoma, Benign prostatic hyperplasia	https://www.targetvalidation.org/target/ENSG00000086205/associations		Cell membrane	Transmembrane	Yes	https://www.bindingdb.org/uniprot/Q04609	✓
FOS	HUMAN Proto-oncogene c-Fos	AP-1, c-fos	P01100	ENSG00000	Transcription factor,Cancer	AK097379	Inflammatory bowel disease, Prostate carcinoma, Osteopetrosis, Leokocyte adhesion deficiency, Goldblatt syndrome, Hyperalgesia, Endometriosis, Crohn's disease, Hepatocellular carcinoma, Infantile osteopetrosis with neuroaxonal dysplasia	https://www.targetvalidation.org/target/ENSG00000170345/associations	RPS6KA6 NLK SGK494 SIRT1 MAPK15 MAPK6 MAPK14 NTRK3 USF2 NCOA1 ELAVL1 TRIP6 RPS6KA1 RPS6KA2 SMAD4 MAPK7 NFATC3 EP300 RELA CSNK2A1 SUMO1 SUMO2 SUMO3 MAPK10 RPS6KA3 MAPK9 MAPK8 STAT1 ELF1	Nucleus, Endoplasmic reticulum, Cytoplasm, cytosol,		Yes	https://www.bindingdb.org/uniprot/P01100	✓
FOSL1	HUMAN Fos-related antigen 1	fra-1	P15407	ENSG00000	Transcription factor,Signalling,Cancer	CR542257	Squamous cell carcinoma, Central nervous system cancer, Psoriasis, Colorectal cancer, Malaria, Mammary neoplasm, Adenocarcinoma, Glioma, Parasitic infection, Lung carcinoma, Lung adenocarcinoma	https://www.targetvalidation.org/target/ENSG00000175592/associations	PRKCQ GSK3B MAPK8 DDIT3 APEX1 JUNB FOSL2 JUN EP300 JUN PSMC3	Nucleus		No		✓

FOSL2	HUMAN Fos-related antigen 2	FLJ23306, FRA2	P15408	ENSG0000(	Transcription factor	AK315415	Inflammatory bowel disease, Central nervous system cancer, Seckel syndrome, Torg-Winchester syndrome, Glioblastoma multiforme, Juvenile arthritis, Idiopathic pulmonary fibrosis, Astrocytoma, Leukemia, Glioma	https://www.targetvalidation.org/target/ENSG00000075426/associations	JUND JUN JUN JUNB FOSL1	Nucleus	No		✓
FOXA3	HUMAN Hepatocyte nuclear factor 3-gamma	HNF3G	P55318	ENSG0000(	Transcription factor	AK223473	Colorectal adenocarcinoma, Li-Fraumeni syndrome, Asthma, Hypoglycemia, Melanoma, Glioblastoma multiforme, Lung adenocarcinoma, Lung carcinoma, Chronic obstructive pulmonary disease	https://www.targetvalidation.org/target/ENSG00000170608/associations		Nucleus	No		✓
FOXI1	HUMAN Forkhead box protein I1	FKHL10, FREAC6, HFH-3, HNF-3	Q12951	ENSG0000(	Transcription factor	U13224	Pendred's syndrome, Li-Fraumeni syndrome, Male infertility with teratozoospermia due to single gene mutation, Male infertility due to globozoospermia, Distal renal tubular acidosis, Goiter, Vestibular dysfunction, Cochlear malformation, Branchio-otic syndrome, Deafness-infertility syndrome	https://www.targetvalidation.org/target/ENSG00000168269/associations	FGF3 PAX8 SIX1	Nucleus	No		✓
FOXN2	HUMAN Forkhead box protein N2	HTLF	P32314	ENSG0000(	Transcription factor	NA	Lung carcinoma, Breast carcinoma, Juvenile dermatomyositis, Pancreatic ductal adenocarcinoma, Endometrial neoplasm, Asthma, Hepatocellular carcinoma, Diffuse large B-cell lymphoma, Sjogren's syndrome, Ovarian carcinoma	https://www.targetvalidation.org/target/ENSG00000170802/associations		Nucleus	No		✓
FOXR2	HUMAN Forkhead box protein R2	FOXN6, MGC21658	Q6PJQ5	ENSG0000(	Transcription factor	NA	Tongue squamous cell carcinoma, Medulloblastoma, Prostate carcinoma, Melanoma, Central nervous system neoplasm, Nerve sheath tumor, Breast carcinoma, Hepatocellular carcinoma, Ewings sarcoma, Neuroblastoma	https://www.targetvalidation.org/target/ENSG00000189299/associations		Nucleus	No		✓
FOXS1	HUMAN Forkhead box protein S1	FKHL18, FREAC10	O43638	ENSG0000(	Transcription factor	AF042831	Lung carcinoma, Squamous cell lung carcinoma, Breast carcinoma, Medulloblastoma, Amyotrophic lateral sclerosis, Cystic fibrosis, Exstrophy-epispadias complex	https://www.targetvalidation.org/target/ENSG00000179772/associations		Nucleus	No		✓
FRK	HUMAN Tyrosine-protein kinase FRK	GTK, PTK5, RAK	P42685	ENSG0000(	Kinase	AL021451	Leukemia, Acute lymphoblastic leukemia, Chronic myelogenous leukemia, Metastatic colorectal cancer, Hepatocellular carcinoma, Colorectal cancer, Melanoma, Prostate carcinoma, Impaired glucose tolerance, Alzheimer's disease	https://www.targetvalidation.org/target/ENSG00000111816/associations	STYK1 STAT3 PTEN BRCA1 RB1 GTF2I CBL PPP1R8	Cytoplasm	Yes		✓
												https://www.bindingdb.org/uniprot/P42685	
FSCN1	HUMAN Fascin	FLJ38511, p55, SNL	Q16658	ENSG0000(	Cancer	AK316607	Epithelial neoplasm, Lymphoid neoplasm, Breast carcinoma, Pancreatic carcinoma, Colonic neoplasm, Adenocarcinoma, Glioma, Bladder neoplasm, Adenoma, Lung carcinoma	https://www.targetvalidation.org/target/ENSG00000075618/associations	PRKCD PRKCE ACTC1 GSK3B CTNNB1 PRKCA NGFR PRKCB PRKCG MAP1LC3A PLXNB3	Cytoplasm, cytosol	No		✓
FTH1	HUMAN Ferritin heavy chain	FTH, FTHL6, PIG15, FRIH, OK/SW-cl.84	P02794	ENSG0000(	Cancer	X03487,X03488	FTH1-related iron overload, Autosomal recessive bestrophinopathy, Phospholipid measurement, Breast carcinoma, Hemoglobin E-Beta-Thalassemia, Keloid, Malignant mesothelioma, Glioma, Hemochromatosis type 2, GRACILE syndrome	https://www.targetvalidation.org/target/ENSG00000167996/associations	TSC1 MAX CXCR4 UBC CSF3R TSC1		Yes		✓
												https://www.drugbank.ca/biodb/polypeptides/P02794	

FTHL17	HUMAN Ferritin heavy polypeptide-like 17	CT38, FHL17	Q9BXU8	ENSG0000(	Others	BC100768	Li-Fraumeni syndrome, Breast carcinoma, Azoospermia, Melanoma, Glioblastoma multiforme, Multiple sclerosis, Adenocarcinoma, Colorectal adenoma	https://www.targetvalidation.org/target/ENSG00000132446/associations				No		✓
FTSJ3	HUMAN pre-rRNA processing protein FTSJ3	SPB1	Q8IY81	ENSG0000(	Cancer,Cancer-Antigen	AC015651	Medulloblastoma, Breast carcinoma, Gastric carcinoma, Pancreatic ductal adenocarcinoma, Hepatocellular carcinoma, Guillain-Barre syndrome, Ovarian neoplasm, Group B Streptococcal Infection	https://www.targetvalidation.org/target/ENSG00000108592/associations		Nucleus, nucleolus		No		✓
FUS	HUMAN RNA-binding protein FUS	ALS6, FUS1, hnRNP-P2, HNRNPP2, TLS	P35637	ENSG0000(	Transcription factor,Cancer	X71428	Nervous system disease, Neurodegenerative disease, Motor neuron disease, Amyotrophic lateral sclerosis, Brain disease, Myxoid liposarcoma, Sarcoma, Adenocarcinoma, Essential tremor, Histiocytoma	https://www.targetvalidation.org/target/ENSG00000089280/associations	SF1 ATM PRKDC ATF4 SPI1 FYN PRKCB SRSF10 MYO6 BCL2 RELA BCL2L11 BCL2L1 TARDBP PA2G4 DROSHA TNPO1	Nucleus		No		✓
FYN	HUMAN Tyrosine-protein kinase Fyn	SLK, SYN	P06241	ENSG0000(	Kinase	AB451293	Leukemia, Chronic myelogenous leukemia, Acute lymphoblastic leukemia, HIV infection, Pancreatic carcinoma, Schizophrenia, Bipolar disorder, Polycythemia vera, Lung carcinoma, Breast carcinoma	https://www.targetvalidation.org/target/ENSG00000010810/associations	CBLC PAG1 GP6 PTPN18 SMO DOK4 NTRK2 DLG2 PTK2B DAG1 GRB10 CBLB PTPRJ TNK2 SOS1 KHDRBS1 PRKCD PRKCZ PTK2 PRKCQ CAV1 TEK PRKCE CDK5 DLG4 TUBA1B RACK1 PTPN5 EPHA4 EPHB3 PXN	Cytoplasm, Nucleus, Cell membrane,		Yes	https://www.bindingdb.org/uniprot/P06241	✓
FZD1	HUMAN Frizzled-1	DKFZp564G072	Q9UP38	ENSG0000(	Cancer	CH236949	Primary ovarian insufficiency, Dental caries, Mayer-Rokitansky-Küster-Hauser syndrome, Perrault syndrome, 46-XY complete gonadal dysgenesis, Ovarian neoplasm, Idiopathic pulmonary fibrosis, Breast neoplasm, Lynch syndrome	https://www.targetvalidation.org/target/ENSG00000157240/associations	WNT6 MACROD1 LRP1 WNT7B WNT3A WNT3 ARRB1 WNT5A WNT2 WNT1 LRP6 DVL1 WNT10B ARRB1 LRP6 WNT1 WNT2 DVL1 WNT5A WNT10B WNT3A WNT6 WNT3 WNT7B	Membrane, Multi-pass membrane protein, Cell membrane	Transmembrane	No		✓
G6PD	HUMAN Glucose-6-phosphate 1-dehydrogenase	G6PD1	P11413	ENSG0000(	Signalling, Cancer	M19866	Abnormality of blood and blood-forming tissues, Anemia, Hematological system disease, Genetic disorder, Hemoglobinopathy, Hemolytic anemia, Neonatal jaundice, Favism, Hematocrit, Congenital nonspherocytic hemolytic anemia	https://www.targetvalidation.org/target/ENSG00000160211/associations	PRKCD SRC HSPB1 SIRT2			Yes	https://www.bindingdb.org/uniprot/P11413	✓
GADD45G	HUMAN Growth arrest and DNA damage-inducible protein GADD45 gamma	CR6, DDIT2	O95257	ENSG0000(	Signalling, Cancer	AF087883	Fibrinogen measurement, 46-XY complete gonadal dysgenesis, 46-XX testicular disorder of sex development, 46-XY partial gonadal dysgenesis, Breast carcinoma, Liver carcinoma, Pituitary neoplasm, Epithelial ovarian cancer, Wiskott-Aldrich syndrome, SERKAL syndrome	https://www.targetvalidation.org/target/ENSG00000130222/associations	MAP3K4 IKBKG CDK1 TRIM24 MAP3K4 PCNA CDK1 CCNB1			No		✓
GAGE1	HUMAN G antigen 1	CT4.1	Q13065	ENSG0000(	Others	AC142496	Neoplasm, cancer, carcinoma, hyperplasia, Epstein-Barr virus infection, melanoma, ovarian carcinoma, nodular goiter	https://www.targetvalidation.org/target/ENSG00000205777/associations				No		✓
GAGE2	HUMAN G antigen 2B/2C	GAG2B, GAGE2B, GAGE2C	Q13066	NA	Others	BC069309	NA	NA				No		✓
GALK1	HUMAN Galactokinase	GALK	P51570	ENSG0000(	Kinase	AK314890	Inborn errors of metabolism, Disorder of galactose metabolism, Galactosemia, Galactokinase deficiency, Cataract, Galactosuria, Prolonged neonatal jaundice, Breast carcinoma, Autosomal recessive predisposition, Hypergalactosemia	https://www.targetvalidation.org/target/ENSG00000108479/associations				Yes	https://www.bindingdb.org/uniprot/P51570	✓

GAPDH	HUMAN Glyceraldehyde-3-phosphate dehydrogenase	GAPD, CDABP0047, OK/SW-cl.12	P04406	ENSG0000(	Others	J02642	Autosomal dominant secondary polycythemia, Glutathione synthetase deficiency, Glutathione synthetase deficiency without 5-oxoprolinuria, Hereditary spherocytosis, Blackfan-Diamond anemia, Delta-beta-thalassemia, Cytomegalovirus infection, Acute coronary syndrome, Necrosis, Hypoxia, Osteoporosis	https://www.targetvalidation.org/target/ENSG00000111640/associations	TBK1 MAP3K3 MAP3K14 SCYL2 CAMK4 PTK2B CAMK1 CAMK2B PRKCD RAB2A PRKCI GLRX AKT2 CASP1 IL7R SRC APP PGK1 EGFR PRKN RIPK2 XPO1 PLD2 ACTB SIRT1 EP300 AKT1 APEX1 SIAH1 TRAP1 AR SNCA	Cytoplasm, cytosol	Yes	https://www.bindingdb.org/uniprot/P04406	✓
GAS2	HUMAN Growth arrest-specific protein 2	GAS2	O43903	ENSG0000(	Others	AC006299	Neuroblastoma, Hepatocellular carcinoma, Lung carcinoma, Melanoma, Hepatitis C infection, Adenoid cystic carcinoma, Salivary gland neoplasm, Medulloblastoma, Lung adenocarcinoma, Colorectal adenocarcinoma	https://www.targetvalidation.org/target/ENSG00000148935/associations	CASP7 CASP3 CAPN2	Cytoplasm, cytoskeleton	No		✓
GAS7	HUMAN Growth arrest-specific protein 7	KIAA0394	O60861	ENSG0000(	Cancer	BT006891	Lung carcinoma, Melanoma, Colonic neoplasm, Cutaneous melanoma, Obesity, Ulcerative colitis, Intracranial hemorrhage, Esophageal adenocarcinoma, Prostate carcinoma, Papillary renal cell carcinoma	https://www.targetvalidation.org/target/ENSG00000007237/associations	ACTA1 WASF1	Cytoplasm	No		✓
GATA1	HUMAN Erythroid transcription factor	ERYF1, GATA-1, GF1, NF-E1	P15976	ENSG0000(	Transcription factor,Cancer	BC009797	Hematological system disease, Congenital dyserythropoietic anemia, Thrombocytopenia with congenital dyserythropoietic anemia, Hemoglobinopathy, Genetic disorder, Down syndrome, Myeloproliferative disorder, Lymphoid neoplasm, Beta-Thalassemia- X-linked thrombocytopenia	https://www.targetvalidation.org/target/ENSG00000102145/associations	HEY1 PAK5 ZNF521 CREBBP PDK1 EP300 CASP3 STAT3 AKT1 MAPK1 MAPK3 SPI1 UBC HSPB1 ZBTB16 FLI1 YY1 SPI1 STAT3 RUNX1 HEY1 MED1 ZFPM1	Nucleus,	No		✓
GATA3	HUMAN Trans-acting T-cell-specific transcription factor GATA-3	HDR	P23771	ENSG0000(	Transcription factor,Signalling,Cancer	M69106	Hypoparathyroidism-deafness-renal disease, Epithelial neoplasm, Lymphoid neoplasm, Acute lymphoblastic leukemia, Breast carcinoma, Barakat syndrome, Renal dysplasia, Adenocarcinoma, Colonic neoplasm, Esophageal squamous cell carcinoma	https://www.targetvalidation.org/target/ENSG00000107485/associations	ASH2L SIX1 NFATC2 SMAD3 STAT6 CDK2 PRKACA MYB SP1 ESR1 NEUROD1 KPNA2 FBXW7 SMAD3 CEBPA IL10 FGF10 CEBPB SOX4 SATB1 ZFPM2	Nucleus,	No		✓
GATAD2A	HUMAN Transcriptional repressor p66-alpha	Hp66alpha	Q86YP4	ENSG0000(	Transcription factor	AC092067	Schizophrenia, LDL cholesterol change measurement, Response to fenofibrate, Astrocytoma, Psoriasis, Type II diabetes mellitus, Breast carcinoma, Pancreatic ductal adenocarcinoma, Retinoblastoma, Acute myeloid leukemia	https://www.targetvalidation.org/target/ENSG00000167491/associations	HDAC2 HDAC1 CDK2 PAK4 CHD4 MBD2 CHD3 MTA2 MBD3 RBBP4 MTA3 RBBP7	Nucleus speckle	No		✓
GCLC	HUMAN Glutamate--cysteine ligase catalytic subunit	GCS, GLCL, GLCLC	P48506	ENSG0000(	Signalling	BC022487	Inborn errors of metabolism, Gamma-glutamylcysteine synthetase deficiency, Hemolytic anemia, Progressive familial intrahepatic cholestasis, Hyperbiliverdinemia, Schizophrenia, Myocardial infarction, Hereditary hemorrhagic telangiectasia, Biliary atresia, Congenital bile acid synthesis defect type 1	https://www.targetvalidation.org/target/ENSG00000001084/associations	PSIP1 GCLM		Yes	https://www.bindingdb.org/uniprot/P48506	✓
GDF15	HUMAN Growth/differentiation factor 15	MIC-1, MIC1, NAG-1, PDF, PLAB, PTGFB	Q99988	ENSG0000(	Kinase,Cancer, Cytokine/Chemokine	AF003934	Melanoma, Central nervous system cancer, Li-Fraumeni syndrome, Pancreatic ductal adenocarcinoma, Osteosarcoma, Left ventricular hypertrophy, Breast carcinoma, Lung carcinoma, Large cell lung carcinoma, Glioma	https://www.targetvalidation.org/target/ENSG00000130513/associations	SP1 TP53 ERBB2	Secreted	No		✓

GEM	HUMAN GTP-binding protein GEM	KIR	P55040	ENSG0000(	Kinase,Signalling	CH471060	Breast carcinoma, Acute myeloid leukemia, Dengue disease, Pancreatitis, Colorectal disease, Diabetes mellitus, Autoimmune disease, Multiple sclerosis, Tongue squamous cell carcinoma, Ovarian carcinoma, Glioma	https://www.targetvalidation.org/target/ENSG00000164949/associations	ATM PRKCZ CACNB1	Cell membrane, Peripheral membrane protein, Cytoplasmic side,	No		✓
GFAP	HUMAN Glial fibrillary acidic protein	FLJ45472	P14136	ENSG0000(	Cancer,Cancer-Antigen	AK128790	Alexander Disease, Epilepsy, Hyperalgesia, Schizophrenia, Bipolar Disorder, Major Depressive Disorder, Gliosis, Amyotrophic Lateral Sclerosis, Parkinson's disease, Nervous system disorder	https://www.targetvalidation.org/target/ENSG00000131095/associations	CAMK2A AURKB CAMK2D ROCK1 PRKCE GSK3B S100A1 PRKACA PRKCA SRC CDK1 PRKCB PRKCG ROCK2 PSEN1 COL16A1	Cytoplasm	No		✓
GGPS1	HUMAN Geranylgeranyl pyrophosphate synthase	GGPPS	O95749	ENSG0000(	Others	AB016043	Liver Cirrhosis, Liver neoplasm, Mumps, Liver and Intrahepatic Biliary Tract Carcinoma, Liver carcinoma, Familial partial lipodystrophy, Mandibuloacral dysplasia, Ataxia-telangiectasia, Wolfram syndrome, Hyperinsulinism	https://www.targetvalidation.org/target/ENSG00000152904/associations	SEPHS1	Cytoplasm,	Yes	https://www.bindingdb.org/uniprot/O95749	✓
GIPC1	HUMAN PDZ domain-containing protein GIPC1	C19orf3, GIPC, GLUT1CBP, Hs.6454, NIP, RGS19IP1, SEMCAP, SYNECTIN, TIP-2	O14908	ENSG0000(	Cancer,Cancer-Antigen	AC012318	Familial idiopathic steroid-resistant nephrotic syndrome, Focal segmental glomerulosclerosis, Atypical hemolytic-uremic syndrome, Fibronectin glomerulopathy, Primary membranoproliferative glomerulonephritis, Mammary neoplasm, Pancreatic neoplasm, Ovarian neoplasm	https://www.targetvalidation.org/target/ENSG00000123159/associations	NTRK2 TGFBR3 PRKCA BCR IGF1R NTRK1 NRP1 ACTN1 MYO6 TGFBR3 TYRP1 ITGA5 RGS19 ADRB1 ITGA6 TPBG PLEKHG5 SEMA4C	Cytoplasm, Membrane, Peripheral membrane protein,	No		✓
GK	HUMAN Glycerol kinase	GKD	P32189	ENSG0000(	Kinase	AJ252550	Glycerol kinase deficiency, Isolated glycerol kinase deficiency, Familial hyperinsulinism, Navajo neurohepatopathy, Congenital isolated hyperinsulinism, MPI-CDG, Mental retardation, Adrenal gland hypofunctionHereditary fructose intolerance, Athyreosis	https://www.targetvalidation.org/target/ENSG00000198814/associations	RXRA NR4A1	Mitochondrion outer membrane, Peripheral membrane protein, Cytoplasmic side, Cytoplasm,	Yes	https://www.bindingdb.org/uniprot/P32189	✓
GK2	HUMAN Glycerol kinase 2	GKP2, GKTA	Q14410	ENSG0000(	Kinase	BC029820	Azoospermia, Abnormality of refraction, Triglyceride measurement, Teratozoospermia, Alzheimer's disease, Stroke, Cervical carcinoma, Abnormality of refraction, Leigh syndrome, Gastric carcinoma	https://www.targetvalidation.org/target/ENSG00000196475/associations		Mitochondrion outer membrane	No		✓
GLRX3	HUMAN Glutaredoxin-3	bA500G10.4, GLRX4, GRX3, GRX4, PICOT, TXNL2	O76003	ENSG0000(	Kinase	AK022131	Thomsen and Becker disease, Alcohol dependence, Cardiomegaly, Colorectal Neoplasms, Colon carcinoma, Lung carcinoma, Kidney neoplasm	https://www.targetvalidation.org/target/ENSG00000108010/associations	PRKD2 PRKCZ PRKCQ	Cytoplasm, cytosol	No		✓
GLS2	HUMAN Glutaminase liver isoform, mitochondrial	GA, GLS, GLSL	Q9UI32	ENSG0000(	Others	AF223944	Schizophrenia, Dementia, Liver carcinoma, Breast Carcinoma, Hepatic Encephalopathy, Glioblastoma, Glioma, Astrocytoma, Hepatocellular carcinoma, Medulloblastoma	https://www.targetvalidation.org/target/ENSG00000135423/associations	SNTA1 TP53 CASK TAX1BP3	Mitochondrion	Yes	https://www.drugbank.ca/biodb/polypeptides/Q9UI32	✓
GMEB1	HUMAN Glucocorticoid modulatory element-binding protein 1	P96PIF	Q9Y692	ENSG0000(	Transcription factor	AK000892	Ischemia, Breast carcinoma, Ovarian carcinoma, Neuroblastoma, Acute myeloid leukemia, Chronic lymphocytic leukemia, Lung carcinoma, Gastric carcinoma	https://www.targetvalidation.org/target/ENSG00000162419/associations	GMEB2 NCOA2	Nucleus, Cytoplasm,	No		✓
GMEB2	HUMAN Glucocorticoid modulatory element-binding protein 2	KIAA1269, P79PIF, PIF79	Q9UKD1	ENSG0000(	Transcription factor	AL353715, AL121907	Chronic lymphocytic leukemia, Melanoma, Breast carcinoma, Hepatocellular carcinoma	https://www.targetvalidation.org/target/ENSG00000101216/associations	GMEB1	Nucleus, Cytoplasm,	No		✓

GMPS	HUMAN GMP synthase [glutamine-hydrolyzing]	GMPS	P49915	ENSG0000(	Cancer	AK315832	Adenocarcinoma, Head and Neck squamous cell carcinoma, Colorectal adenocarcinoma, Acute myelocytic leukemia, Schizophrenia, Autoimmune diseases, African trypanosomiasis, Tuberculosis, Precursor cell lymphoblastic lymphoma, Luminal B breast carcinoma	https://www.targetvalidation.org/target/ENSG00000163655/associations	CDK2<br>TRIM21<br>CDK1<br>TP53<br>USP7	Cytoplasm,	Yes	https://www.drugbank.ca/biodb/polypeptides/P49915	✓
GNA15	HUMAN Guanine nucleotide-binding protein subunit alpha-15	GNA16	P30679	ENSG0000(	Signalling	AF493904	Prostate carcinoma, Li-Fraumeni syndrome, Acute leukemia, Low grade B-cell lymphoma, Pancreatic carcinoma, Neuroendocrine tumor, Plasmacytoma, Acute myelocytic leukemia, Non-Hodgkin lymphoma	https://www.targetvalidation.org/target/ENSG00000060558/associations	PRKCE<br>F2R<br>PRKCA<br>SRC<br>CHRM2<br>ADRB2<br>PRKCB<br>PRKCG<br>OPRD1<br>LTB4R		Yes	https://www.bindingdb.org/uniprot/P30679	✓
GNAI2	HUMAN Guanine nucleotide-binding protein G(i) subunit alpha-2	GNAI2B	P04899	ENSG0000(	Signalling	AY677118	Hyper-IgM syndrome with susceptibility to opportunistic infections, Arrhythmogenic right ventricular dysplasia, Inflammatory bowel disease, Hyper-IgM syndrome type 3, Genetic cardiac anomaly, Familial ventricular tachycardia, Sudden cardiac death, Bradycardia, Paroxysmal ventricular tachycardia, Abnormality of metabolism/homeostasis	https://www.targetvalidation.org/target/ENSG00000114353/associations	GNB4<br>SMO<br>MAP3K3<br>TTC1<br>RNF2<br>PRKCE<br>GPSM2<br>GNB1<br>CCR5<br>RGS19<br>MTNR1A<br>CXCR5<br>CXCR2<br>CXCR1<br>DRD2<br>CXCL8<br>IGF1R<br>EGFR<br>CHRM1<br>MDFI<br>TRPC4<br>F2R<br>ADCY1<br>TTC1<br>EDNRA<br>OPRD1<br>C5AR1<br>ADORA1<br>LPAR3	Cytoplasm	No		✓
GNAI3	HUMAN Guanine nucleotide-binding protein G(k) subunit alpha	87U6	P08754	ENSG0000(	Signalling	J03198	Auriculocondylar syndrome, Obesity, Achromatopsia, Autosomal recessive spondylocostal dysostosis, Auriculo-condylar syndrome, Big calvaria, Hypoplasia of condylar head of mandible, Small condylar head of mandible	https://www.targetvalidation.org/target/ENSG00000065135/associations	RIPK3<br>GNB4<br>SMO<br>MAP3K3<br>GPSM1<br>CCDC88A<br>GRM7<br>GNG3<br>GNAI1<br>GNB2<br>GNB1<br>CCR5<br>RGS3<br>RGS19<br>MTNR1A<br>BDKRB1<br>RGS2<br>OPRL1<br>OPRK1<br>OPRD1<br>DRD3<br>OPRM1<br>CNR2<br>SSTR2<br>SSTR1<br>ADORA1<br>PTAFR<br>CXCR2<br>DRD1	Cytoplasm	Yes	https://www.bindingdb.org/uniprot/P08754	✓
GNAO1	HUMAN Guanine nucleotide-binding protein G(o) subunit alpha	G-ALPHA-o, GNAO	P09471	ENSG0000(	Signalling	AF493894	Early infantile epileptic encephalopathy, Li-Fraumeni syndrome, Schizophrenia, Epileptic encephalopathy, Hypertonic Seizures, Delayed myelination, Cerebral atrophy, Supratentorial atrophy	https://www.targetvalidation.org/target/ENSG00000087258/associations	GABBR1<br>GRM7<br>RGS19<br>OPRL1<br>OPRK1<br>OPRD1<br>DRD3<br>OPRM1<br>CNR2<br>SSTR2<br>SSTR1<br>PTAFR<br>CNR1<br>DRD2<br>ADRA2A<br>CHRM2<br>APP<br>RGS16<br>GRM6<br>ZBTB16<br>ADCY1<br>OPRD1<br>CACNA1B<br>SCN8A<br>KLHL3<br>ADRA2C<br>RGS1<br>S1PR5	Cell membrane	Yes	https://www.bindingdb.org/uniprot/P09471	✓
GNAZ	HUMAN Guanine nucleotide-binding protein G(z) subunit alpha	GNAZ	P19086	ENSG0000(	Signalling	CR456495	Prostate carcinoma, Von Willebrand disease, Hermansky-Pudlak syndrome, Rare hemorrhagic disorder due to a platelet receptor defect, Bipolar disorder, Melanoma, Night blindness, congenital stationary	https://www.targetvalidation.org/target/ENSG00000128266/associations	AURKB<br>PAK1<br>PRKCD<br>PRKCE<br>SYN1<br>PRKCA<br>DRD2<br>HTR1A<br>PRKCB<br>PRKCG<br>RGS20<br>ADCY5<br>ADCY1<br>NMT1<br>RAP1GAP<br>DRD5	Membrane, Lipid-anchor,	No		✓
GNB1	HUMAN Guanine nucleotide-binding protein G(I)/G(S)/G(T) subunit beta-1	GBB1	P62873	ENSG0000(	Signalling, Cancer	CR456784	Autosomal dominant non-syndromic intellectual disability, Seizures, Mental retardation, Growth delay, Nystagmus, Anxiety disorders, Depressive disorder, Schizophrenia, Asthma, Breast Carcinoma, Acute lymphocytic leukemia	https://www.targetvalidation.org/target/ENSG00000078369/associations	RIPK3<br>HDAC5<br>PHPT1<br>FZD3 SMO<br>MAP3K3<br>GNA12<br>GNGT1<br>GNAI1<br>GNAS<br>GNG2<br>ARRB1<br>KCNJ3<br>MTOR<br>GNA11<br>GRK2<br>NME2<br>TBXA2R<br>MCF2<br>GNAI3<br>ADRB2<br>GNAI2<br>ESR1<br>ADCY5<br>RACK1<br>GNAI2<br>PLCB2<br>GNAI3<br>PLCB1<br>ARRB1		No		✓
GNB3	HUMAN Guanine nucleotide-binding protein G(I)/G(S)/G(T) subunit beta-3	GBB3	P16520	ENSG0000(	Signalling	Y12050	Retinal dystrophy, Congenital stationary night blindness, Obesity, Autosomal dominant optic atrophy (classic type), Hypertensive disease, Schizophrenia, Coronary artery disease, Major depressive disorder, Mental depression, Essential hypertension, Bipolar disorder	https://www.targetvalidation.org/target/ENSG00000111664/associations	FZD3 SMO<br>PTGER3<br>TGFBR1<br>PLCG1<br>PIK3CA<br>SMO<br>GNG4<br>GNGT1<br>FZD3<br>GNG3<br>GNG12<br>GNG2<br>GNG13<br>GNG5<br>GNG7<br>GNG8<br>GNG10		No		✓
GNG11	HUMAN Guanine nucleotide-binding protein G(I)/G(S)/G(O) subunit gamma-11	GNGT11, GBG11	P61952	ENSG0000(	Signalling	BC009709	Resting heart rate, Lung carcinoma, Breast carcinoma, Lung adenocarcinoma, Lung adenocarcinoma, Juvenile arthritis	https://www.targetvalidation.org/target/ENSG00000127920/associations	GNB1	Cell membrane	No		✓

GNG2	HUMAN Guanine nucleotide-binding protein G(I)/G(S)/G(O) subunit gamma-2	GBG2	P59768	ENSG0000(	Signalling	BC020774	FEV/FEC ratio, Response to bronchodilator, Skin pigmentation, Melanoma	https://www.targetvalidation.org/target/ENSG00000186469/associations	GNB4 SMO BTK GNAI1 GNB2 GNB1 GNB3 CHRM2 CACNA1H GNB5 RACK1 PLCG1 PIK3CA SMO GNAI1 GNB1	Cell membrane		Yes		✓
													https://www.drugbank.ca/biodb/polypeptides/P59768	
GNG3	HUMAN Guanine nucleotide-binding protein G(I)/G(S)/G(O) subunit gamma-3	GNGT3	P63215	ENSG0000(	Signalling	AF493871	Central nervous system cancer, Astrocytoma, Glioblastoma multiforme, Ependymoma, Oligodendroglioma, Seizures	https://www.targetvalidation.org/target/ENSG00000162188/associations	GNB4 SMO GNB2 GNB1 GNB3 GNAI3 GNB5 GNAI3 PLCG1 PIK3CA SMO	Cell membrane		No		✓
GNG4	HUMAN Guanine nucleotide-binding protein G(I)/G(S)/G(O) subunit gamma-4	GNGT3, GBG4	P50150	ENSG0000(	Signalling	NA	Lung carcinoma, Hepatocellular carcinoma, Colorectal adenocarcinoma, Renal cell carcinoma	https://www.targetvalidation.org/target/ENSG00000168243/associations	GOPC GNB4 GNB2 GNB1 GNB3 RAF1 GNB5	Cell membrane		No		✓
GNGT2	HUMAN Guanine nucleotide-binding protein G(I)/G(S)/G(O) subunit gamma-T2	GNG8, GNGT8, GNG9	O14610	ENSG0000(	Signalling	AK312839	Sepsis, Breast adenocarcinoma, Colorectal adenocarcinoma, Ovarian carcinoma, Osteosarcoma, Coronary heart disease, Type II diabetes mellitus	https://www.targetvalidation.org/target/ENSG00000167083/associations	GNB2 GNB1 KCNJ4 GNB2	Cell membrane		No		✓
GOT1	HUMAN Aspartate aminotransferase, cytoplasmic	AATC	P17174	ENSG0000(	Cancer,Cancer-Antigen	AK301916	Astrocytoma, Glioblastoma multiforme, Medulloblastoma, Severe acute respiratory syndrome, Impaired glucose tolerance, Periodontal diseases, Biliary cirrhosis, Metabolic syndrome X	https://www.targetvalidation.org/target/ENSG00000120053/associations		Cytoplasm		Yes		✓
													https://www.drugbank.ca/biodb/polypeptides/P17174	
GP2	HUMAN Pancreatic secretory granule membrane major glycoprotein GP2	GP2	P55259	ENSG0000(	Others	AB035541	Lung carcinoma, Type I diabetes mellitus, Chronic pancreatitis, Ulcerative colitis, Crohn's disease, Inflammatory bowel diseases, Epithelial ovarian cancer, Idiopathic chronic pancreatitis, Hyperuricemic nephropathy, Ovarian Carcinoma	https://www.targetvalidation.org/target/ENSG00000169347/associations	ANXA4 YES1 AMY2A	Cell membrane		No		✓
GPA33	HUMAN Cell surface A33 antigen	A33	Q99795	ENSG0000(	Cancer	CH471067	Colonic neoplasm, Colorectal adenocarcinoma, Hypertensive disease, Osteoporosis, Colon carcinoma, Adenoma, Adenoma, Adenomatous polyps	https://www.targetvalidation.org/target/ENSG00000143167/associations		Membrane, Single-pass type I membrane protein,	Transmembrane	No		✓
GPAT2	HUMAN Glycerol-3-phosphate acyltransferase 2, mitochondrial	CT123	Q6NUI2	ENSG0000(	Others	BC068596	Li-Fraumeni syndrome, Tongue squamous cell carcinoma, Breast neoplasm, Melanoma, Breast carcinoma	https://www.targetvalidation.org/target/ENSG00000186281/associations		Mitochondrion outer membrane	Transmembrane	No		✓
GPATCH2	HUMAN G patch domain-containing protein 2	GPATC2, GPTC2	Q9NW75	ENSG0000(	Others	CH471100	Visceral adipose tissue measurement, Breast carcinoma, Resting heart rate, Large cell lung carcinoma, renal cell carcinoma, Breast neoplasm	https://www.targetvalidation.org/target/ENSG00000092978/associations		Nucleus speckle		No		✓
GPC3	HUMAN Glypican-3	DGSX, OCI-5, SDYS, SGB, SGBS, SGBS1	P51654	ENSG0000(	Cancer	L47176	Overgrowth syndrome, Simpson-Golabi-Behmel syndrome, Epithelial neoplasm, Adenocarcinoma, Lung carcinoma, Kidney neoplasm, Melanoma, Accessory nipple, Mesothelioma, Ventricular septal defects	https://www.targetvalidation.org/target/ENSG00000147257/associations	FGF2 IGF2 DPP4	Cell membrane		No		✓
GPHN	HUMAN Gephyrin	KIAA1385, GEPH, GPH	Q9NQX3	ENSG0000(	Cancer	AB037806	Encephalopathy due to sulfite oxidase deficiency, Sulfite oxidase deficiency due to molybdenum cofactor deficiency, Sulfite oxidase deficiency due to molybdenum cofactor deficiency type C, Hereditary hyperexplexia, Epilepsy, Hyperexplexia, Polymicrogyria, Hyperreflexia	https://www.targetvalidation.org/target/ENSG00000171723/associations	GABARAPL3 PIN1 TUBA4A DYNLL1 GSK3B GLRB MTOR ARHGEF9 MTOR TUBA1A GLRA1 GABARAPL3	Cell junction, synapse		Yes		✓
													https://www.drugbank.ca/biodb/polypeptides/Q9NQX3	

GPX1	HUMAN Glutathione peroxidase 1	GPX1	P07203	ENSG0000(	Cancer	X13710	Self-reported educational attainment, Hypertensive disease, Hyperthyroidism, Mammary neoplasm, Type II diabetes mellitus, Schizophrenia, Squamous cell carcinoma, Bladder neoplasm, Dilated cardiomyopathy	https://www.targetvalidation.org/target/ENSG00000233276/associations	ABL2 TP53 ABL1 SELENBP 1	Cytoplasm,	Yes	https://www.bindingdb.org/uniprot/P07203	✓
GRAP2	HUMAN GRB2-related adapter protein 2	GADS, GRBLG, GrbX, Grf40, Mona	O75791	ENSG0000(	Kinase	AF236119	Breast carcinoma, Severe combined immunodeficiency, Liver carcinoma, Prostate carcinoma, Rheumatoid arthritis, Hepatitis C infection, Thyroid Neoplasm, Non-small cell lung carcinoma	https://www.targetvalidation.org/target/ENSG00000100351/associations	MAP4K1 LAX1 LCP2 SHC1 CBL CD28 KIT CSF1R ERBB2 LAT GAB1 GAB2 LCP2	Nucleus	No		✓
GRB10	HUMAN Growth factor receptor-bound protein 10	GRBIR, KIAA0207	Q13322	ENSG0000(	Signalling, Cancer	AF001534	Fasting blood glucose measurement, Response to bronchodilator, Schizophrenia, Squamous cell carcinoma, Russell-Silver syndrome, Type II diabetes mellitus, Retinoblastoma, CREST syndrome, Somatotropin deficiency, Ewings sarcoma	https://www.targetvalidation.org/target/ENSG00000106070/associations	MAP3K14 MAP2K1 EPHB1 TEC MTOR KDR AKT1 MAPK1 MAPK3 SRC BCR GHR KIT PDGFRB IGF1R RET FYN INSR RAF1 EGFR JAK2 SRC FYN RAF1 IGF1R NEDD4 AKT1 INSR PIK3CA ABL1 KIT KDR DOK1	Cytoplasm	Yes	https://www.bindingdb.org/uniprot/Q13322	✓
GRB2	HUMAN Growth factor receptor-bound protein 2	NCKAP2	P62993	ENSG0000(	Kinase,Signalling,Cancer	AF063618	Listeriosis, Achondrogenesis, Hypochondrogenesis, Schizophrenia, Alzheimer's disease, Breast carcinoma, Fibroid tumor, Hepatitis C infection, Central neuroblastoma	https://www.targetvalidation.org/target/ENSG00000177885/associations	MAP4K5 IRS2 GAB2 ALK CBLC VAV3 ERRFI1 SH2B1 LY9 LAT2 SH3KBP1 MAP4K1 USP6NL WASF1 SHC3 BLNK FRS2 TUBA1A SHC4 MAPK14 RPS6KA1 ERBB4 PTK2B IRF5 GAB1 ADAM15 MAP3K1 CBLB PAK1 LCP2 MERTK PNRC1	Nucleus	Yes	https://www.bindingdb.org/uniprot/P62993	✓
GRB7	HUMAN Growth factor receptor-bound protein 7	GRB7	Q14451	ENSG0000(	Cancer,Cancer-Antigen	AF274875	Breast carcinoma, Squamous cell carcinoma, Adenocarcinoma, Testicular germ cell tumor, Lung neoplasm, Prostatic neoplasm, Renal cell carcinoma, Chronic lymphocytic leukemia, Liver carcinoma	https://www.targetvalidation.org/target/ENSG00000141738/associations	MAP3K14 PTPN11 PTK2 CAV1 TEK CALM1 EPHB1 ERBB3 KIT PDGFRB MET RET INSR ERBB2 EGFR HAX1 CAV1 INSR KIT RND1	Cytoplasm	Yes	https://www.bindingdb.org/uniprot/Q14451	✓
GRK2	HUMAN Beta-adrenergic receptor kinase 1	BARK1, GRK2, ARBK1	P25098	ENSG0000(	Kinase	EU326303	Heart disease, Congenital left-sided heart lesions, Matthew-Wood synfrome, Left-ventricular noncompaction, Congenital alveolar capillary dysplasia, Rheumatoid arthritis, Cardiomegaly, Cardiomyopathy, CHILD syndrome, Lethal faciocardiomelic dysplasia	https://www.targetvalidation.org/search?src=q:P25098	NLK MAPK15 MAPK6 MAPK7 PRKCD ROR2 MDM2 GNB1 CALM1 MAPK10 PRKX CCR5 ARRB1 MAPK9 MAPK8 MAPK4 MAPK1 MAPK3 CDK2 CAPN2 PRKACA PRKCA SRC ADRB2 TUBB PRKCG EGF PRKY PRKCB HDAC6	Cytoplasm	Yes	https://www.bindingdb.org/uniprot/P25098	✓
GRK3	HUMAN Beta-adrenergic receptor kinase 2	ADRBK2, BARK2, ARBK2	P35626	ENSG0000(	Kinase	NA	Astrocytoma, Central nervous system cancer, Breast carcinoma, Glioblastoma multiforme, Colorectal adenocarcinoma, Azoospermia, Ependymoma, Schizophrenia, Hypothyroidism, WHIM syndrome	https://www.targetvalidation.org/target/ENSG00000100077/associations	AGTR1 OPRM1 BDKRB2 CXCR4 CCR5 ADRB2 TBXA2R CCR4 SSTR2 MC4R CHRM2 GIT1 OPRL1 XK		Yes	https://www.bindingdb.org/uniprot/P35626	✓
GRK5	HUMAN G protein-coupled receptor kinase 5	GPRK5	P34947	ENSG0000(	Kinase,Cancer,Cancer-Antigen	AL583824, AL355273, AL355861	Diabetes mellitus, Colorectal adenocarcinoma, Colonic neoplasm, Major depressive disorder, Parkinson's disease, Asthma, Narcolepsy, Takotsubo cardiomyopathy, Hyperphenylalaninemia, Malignant mesothelioma	https://www.targetvalidation.org/target/ENSG00000198873/associations	MAST1 MAP3K14 ARRB1 CAV1 AGTR1 ARRB1 TP53 HSP90AA1 LRP6 BDKRB2 CAMK2A ENO1 SNCA CCR5 RGCC ADRB2 SNCG ZYX TBXA2R SNCB PTPN7 ST13 MC4R CHRM2 GIT1 DLGAP2 PINX1 AVPR2 RHO TACR1	Cytoplasm, Nucleus, Cell membrane, Peripheral membrane protein,	Yes	https://www.bindingdb.org/uniprot/P34947	✓

GRK6	HUMAN G protein-coupled receptor kinase 6	GPRK6	P43250	ENSG00000	Kinase	AC145098	Coronary artery calcification, Rheumatoid arthritis, Schizophrenia, Parkinson's disease, Neoplasm metastasis, Melanoma, Medulloblastoma, Dyskinetic syndrome	https://www.targetvalidation.org/target/ENSG00000198055/associations	MAPK1 HSP90AA1 SLC9A3R1 SLC9A3R1 PRKCD IGF1R BDKRB2 SNCA CXCR4 CCR5 ADRB2 PRSS23 TBXA2R NF2 FSHR EDNRA MC4R CHRM2 GIT1 EDNRB MC1R LTB4R RHO CHRM3 RCVRN	Membrane, Lipid-anchor,		Yes	https://www.bindingdb.org/uniprot/P43250	✓
GRM3	HUMAN Metabotropic glutamate receptor 3	GPRC1C, mGlu3, MGLUR3	Q14832	ENSG00000	Others	AC005009	Mental or behavioural disorder, Schizophrenia, Central nervous system cancer, Bipolar Disorder, Chronic alcoholic intoxification, Melanoma, Gliosis	https://www.targetvalidation.org/target/ENSG00000198822/associations	PPM1A	Cell membrane	Transmembrane	Yes	https://www.bindingdb.org/uniprot/Q14832	✓
GSK3B	HUMAN Glycogen synthase kinase-3 beta	GSK3B	P49841	ENSG00000	Kinase	BC000251	Leukemia, Depressive disorder, Bipolar disorder, Alzheimer's disease, Myocardial infarction, Schizophrenia, Hypertensive disease, Prostatic neoplasm, HIV infection, Osteosarcoma	https://www.targetvalidation.org/target/ENSG00000082701/associations	MAP4K5 AXIN2 AKT3 RPS6KA6 SGK2 SGK494 SGK3 FRAT1 TSC1 MARK2 AMER1 MAPK14 STK11 RPS6KA1 RPS6KA2 PIN1 ILK PPP3CA PRKCD PRKCZ MAP2K1 PPP2CA CDC42 PRKX RPS6KA3 TSC2 CSNK1D CSNK1A1 PPP3CC CTNNB1	Cytoplasm Nucleus, Cell membrane,		Yes	https://www.bindingdb.org/uniprot/P49841	✓
GSTT1	HUMAN Glutathione S-transferase theta-1	GSTT1	P30711	NA	Cancer,Cancer-Antigen	AB057594	Lung neoplasm, Chromosome Aberrations, Hypertensive disease, Prostatic neoplasm, Kidney neoplasm, Renal cell carcinoma, Schizophrenia, Skin neoplasm	NA		Cytoplasm,		Yes	https://www.drugbank.ca/biodb/polypeptides/P30711	✓
GTF2A2	HUMAN Transcription initiation factor IIA subunit 2	TFIIA, T2AG, TF2A2	P52657	ENSG00000	Transcription factor	BT007362	HIV infection, Teratozoospermia, Breast carcinoma, Malignant peripheral nerve sheath tumor, Multiple myeloma, Embryonal carcinoma, Prostate adenocarcinoma, Breast adenocarcinoma	https://www.targetvalidation.org/target/ENSG00000140307/associations	RBPJ GTF2E1 TBP TAF4 TBP GTF2A1 GTF2A1L	Nucleus,		No		✓
GTF2B	HUMAN Transcription initiation factor IIB	TFIIB, TF2B	Q00403	ENSG00000	Transcription factor	AB451296	HIV infection, Glioblastoma multiforme, Autoimmune Diseases, Melanoma, Multiple sclerosis, Osteoporosis, Non-small cell lung carcinoma, Tumor Angiogenesis	https://www.targetvalidation.org/target/ENSG00000137947/associations	GTF2H4 TBPL1 POLR2J GTF2F1 POLR2A TAF1 TBP POLR2E CREB1 GTF2F2 THRA NR2F1 ESR1 TAF1 RELA POLR2A TCEA2 GTF2F1 GTF2E1 GTF2E2 TAF9 TAF11	Nucleus,		No		✓
GTF2E2	HUMAN Transcription initiation factor IIE subunit beta	TF2E2, T2EB	P29084	ENSG00000	Transcription factor	CH471080	Trichothiodystrophy, HIV infection, Hydroxy-leucine measurement, Ulcerative colitis	https://www.targetvalidation.org/target/ENSG00000197265/associations	GTF2B MNAT1 CCNH GTF2F1 GTF2H1 GTF2E1 POLR2A XPA TBP ERCC3 GTF2F2 SUPT16H GTF2A1 GTF2H1	Nucleus		No		✓
GTF2F1	HUMAN General transcription factor IIF subunit 1	RAP74, TFIIF, T2FA	P35269	ENSG00000	Transcription factor	AK315240	HIV infection, Influenza infection, Exstrophy-epispadias complex, Dermatomyositis, Systemic lupus erythematosus, Rheumatoid arthritis, Mixed-connective tissue disease, Breast neoplasm, Epithelial ovarian cancer	https://www.targetvalidation.org/target/ENSG00000125651/associations	CTDP1 GTF2H4 GTF2B GTF2A1 POLR2J CDK9 GTF2E1 CDK2 POLR2A TAF1 GTF2F2 SRF AR CDK1 MYC TAF1 POLR2A TBP AR CDK9 PSMC2 RARG GTF2B CTDSP1 CTDP1 GTF2A1 GTF2E1 GTF2E2 GTF2F2 GTF2H4 TAF11 POLR2J	Nucleus,		No		✓
GTF2F2	HUMAN General transcription factor IIF subunit 2	RAP30, TFIIF	P13984	ENSG00000	Transcription factor	BT019525	HIV infection, Influenza infection, Juvenile dermatomyositis, Hepatitis, Breast carcinoma, Anaplastic oligodendroglioma, Melanoma, Epithelial ovarian cancer	https://www.targetvalidation.org/target/ENSG00000188342/associations	POLR2G POLR2J GTF2F1 POLR2B POLR2A POLR2E AR POLR2B POLR2A TBP AR POLR2E GTF2B GTF2F1 GTF2E1 GTF2E2 TAF5 POLR2J POLR2G	Nucleus		No		✓
GTF2H1	HUMAN General transcription factor IIH subunit 1	BTF2, TFIIH	P32780	ENSG00000	Transcription factor,Cancer	AY163770	HIV infection, Melanoma, Xeroderma pigmentosum, Trichothiodystrophy, Osteitis deformans, Herpes simplex infections, Amyotrophic lateral sclerosis, Narcolepsy, Multiple sclerosis	https://www.targetvalidation.org/target/ENSG00000110768/associations	ERCC4 GTF2H4 GTF2H3 GTF2H2 XPC HNRNPU MNAT1 CCNH CDK7 GTF2E2 ERCC5 POLR2A XPA ERCC3 ERCC2 AR TP53 E2F1 ERCC4 CCNH XPA CDK7 POLR2A ERCC5 XPC AR MNAT1 ERCC2 ERCC3 GTF2E1 GTF2E2 GTF2H3 GTF2H2	Nucleus,		No		✓

GTF2H2C	HUMAN General transcription factor IIH subunit 2-like protein	T2H2L, GTF2H2D	Q6P1K8	ENSG0000(	Transcription factor	BC064557	Lung carcinoma, Colorectal adenocarcinoma, Large cell lung carcinoma, Melanoma, Pancreatic carcinoma, Astrocytoma, Oligodendroglioma, Ependymoma, Adrenocortical carcinoma, Pancreatic carcinoma	https://www.targetvalidation.org/target/ENSG00000183474/associations		Nucleus	No		✓
GTPBP1	HUMAN GTP-binding protein 1	GP1	O00178	ENSG0000(	Others	U87964	Lung adenocarcinoma, Asthma, Psoriasis, Autoimmune disease, Thrombophilia, Protein C deficiency, Liver carcinoma, Ischemia, Primary atiphospholipid syndrome, Borna disease	https://www.targetvalidation.org/target/ENSG00000100226/associations	IRF5 LRP1	Cytoplasm	No		✓
GUK1	HUMAN Guanylate kinase	KGUA, GMK	Q16774	ENSG0000(	Kinase	AL359510	Breast carcinoma, Ovarian carcinoma, Glioma, Astrocytoma, Osteosarcoma, Coronary heart disease, Pituitary adenoma, Glioblastoma multiforme, Adenoma	https://www.targetvalidation.org/target/ENSG00000143774/associations			Yes	https://www.drugbank.ca/biodb/polypeptides/Q16774	✓
H2AFY	HUMAN Core histone macro-H2A.1	macroH2A1.2, H2AY	O75367	ENSG0000(	Cancer,Cancer-Antigen	AK023409	Phosphoenolpyruvate carboxykinase deficiency, Bladder carcinoma, Familial partial lipodystrophy, Melanoma, Autistic disorder, Steatohepatitis, Hepatocarcinogenesis, Breast carcinoma, Colon carcinoma	https://www.targetvalidation.org/target/ENSG00000113648/associations	SPOP PARP1 ERBB2 ATF2 TNKS	Nucleus	No		✓
HAGH	HUMAN Hydroxyacylglutathione hydrolase, mitochondrial	GLO2, HAGH1	Q16775	ENSG0000(	Cancer,Cancer-Antigen	AE006639	Sarcoma, Osteosarcoma, Leukemia, Melanoma, Schizophrenia, Prostatic neoplasm, Breast carcinoma, Autism spectrum disorder, Renal carcinoma, Adenocarcinoma	https://www.targetvalidation.org/target/ENSG00000063854/associations		Mitochondrion matrix,	Yes	https://www.bindingdb.org/uniprot/Q16775	✓
HAGHL	HUMAN Hydroxyacylglutathione hydrolase-like protein	HAGHL	Q6PII5	ENSG0000(	Cancer,Cancer-Antigen	CH471112	Breast carcinoma, Diffuse large B-cell lymphoma, Sjogren's syndrome, Melanoma, Prostate carcinoma	https://www.targetvalidation.org/target/ENSG00000103253/associations			No		✓
HAND1	HUMAN Heart- and neural crest derivatives-expressed protein 1	bHLHa27, eHand, Hxt, Thing1	O96004	ENSG0000(	Transcription factor	NA	QRS duration measurement, Lymphoma, Malignant mesothelioma, Heart septal defects, Congenital heart disease, Tetralogy of Fallot, Gastric carcinoma, Congenital abnormality, Dilated cardiomyopathy, Congestive heart failure	https://www.targetvalidation.org/target/ENSG00000113196/associations	PRKCE PRKX PRKACA PRKCA CDK1 PRKCB PRKCG PRKY MEF2A FHL2 ACHE HAND2	Nucleus, nucleoplasm	No		✓
HBG1	HUMAN Hemoglobin subunit gamma-1	HBG-T2	P69891	ENSG0000(	Signalling	V00514	Sickle cell anemia, Osteosarcoma, Mental retardation, Hemoglobin F disease, Anemia, Delta-Beta Thalassemia, Iron-Refractory Iron Deficiency Anemia, Splenomegaly	https://www.targetvalidation.org/target/ENSG00000213934/associations			No		✓
HCFC2	HUMAN Host cell factor 2	HCF-2	Q9Y5Z7	ENSG0000(	Transcription factor	AC084359	Azoospermia, Breast carcinoma, Melanoma, Anaplastic large cell lymphoma, Anaplastic oligodendroglioma, Liver neoplasms, Hepatocarcinogenesis	https://www.targetvalidation.org/target/ENSG00000111727/associations	ASH2L WDR5	Cytoplasm	No		✓
HCLS1	HUMAN Hematopoietic lineage cell-specific protein	CTTNL, HS1	P14317	ENSG0000(	Transcription factor	AK312750	Li-Fraumeni syndrome, Malignant mesothelioma, Tongue squamous cell carcinoma, Rheumatoid arthritis, lupus erythematosus, Neuroblastoma, Glioma, Melanoma, Diabetes mellitus	https://www.targetvalidation.org/target/ENSG00000180353/associations	DYRK1B DYRK4 MAP4K1 HIPK4 PDIK1L TNNI3K CDK5 CSNK2A1 GSK3B SYK WEE1 TYK2 FGFR4 SRC CD79A FGFR1 FGR LYN FYN ABL1 DYRK3 LYN SRC CASP3 WAS HS1BP3	Membrane	No		✓

HDAC1	HUMAN Histone deacetylase 1	GON-10, HD1, RPD3L1	Q13547	ENSG0000(	Transcription factor	NA	Lymphoid neoplasm, Neoplasm of mature B-cells, Lymphoma, Non-Hodgkin's lymphoma, Myelodysplastic syndrome, Cutaneous T-cell lymphoma, Schizophrenia, Squamous cell carcinoma, Endometriosis, Familial primary pulmonary hypertension	https://www.targetvalidation.org/target/ENSG00000116478/associations	TRAF6 SENP1 TAB2 SPHK2 SMARCAD1 NKX3-1 SIN3A EHMT2 CREBBP ZNF420 CDKN2A PIAS4 BCOR KCTD11 HIF1A E2F4 MECOM CTBP1 ATM TRIM28 RUNX1T1 PPARD CREM TFAP4 RUNX1 E2F1 NFKB2 CSNK2A1 UBE2I	Nucleus	Yes	https://www.bindingdb.org/uniprot/Q13547	✓
HDAC3	HUMAN Histone deacetylase 3	HD3, RPD3, RPD3-2	O15379	ENSG0000(	Others	AF005482	Lymphoid neoplasm, Neoplasm of mature B-cells, Lymphoma, Schizophrenia, Erectile dysfunction, Fibrosis, Liver carcinoma, Glioma, Colonic Neoplasm	https://www.targetvalidation.org/target/ENSG00000171720/associations	NCOR2 HDAC5 TAB2 HDAC10 HDAC7 BCOR MAPK11 HDAC1 CSNK2A1 PPP4C NRIP1 CCND1 BCL3 MYC NCOR1 AKAP8 HR CHUK XPO1 SRC NCOR2 HIF1A E2F1 HDAC4 NFE2L2 EP300 STAT1 MAFK NFKB1 JUN TRIM28 YY1 RELA	Nucleus	Yes	https://www.bindingdb.org/uniprot/O15379	✓
HDAC4	HUMAN Histone deacetylase 4	HD4, HDAC4, KIAA0288	P56524	ENSG0000(	Transcription factor	AC062017	Lymphoid neoplasm, Neoplasm of mature B-cells, Multiple myeloma, Myelodysplastic syndrome, 2q37 microdeletion syndrome, Cutaneous T-cell lymphoma, Non-Hodgkin's lymphoma, Brachydactyly, Schizophrenia, Obesity	https://www.targetvalidation.org/target/ENSG00000068024/associations	NCOR2 HDAC5 HDAC9 AURKB CAMKK1 MARK2 CAMK4 CAMK2D PRKAA1 TP53BP1 MEF2C RELA PPP2CA UBE2I SUMO1 SIK1 NR1H2 CSRP3 GSK3B RANBP2 CBX5 YWHAB MAPK1 MAPK3 NFKBIA YY1 ATF2 SRF SP1 ESR1 NUP155	Nucleus, Cytoplasm,	Yes	https://www.bindingdb.org/uniprot/P56524	✓
HES6	HUMAN Transcription cofactor HES-6	bHLHb41	Q96HZ4	ENSG0000(	Transcription factor	AK293111	Glioma, Medulloblastoma, Oligodendroglioma, Prostate carcinoma, Breast carcinoma, Colon carcinoma, Ovarian neoplasm, Cirrhosis	https://www.targetvalidation.org/target/ENSG00000144485/associations	HES1 TLE1 CSNK2A1 HES1	Nucleus	No		✓
HEXIM1	HUMAN Protein HEXIM1	CLP-1, EDG1, HIS1, MAQ1, HEXI1	O94992	ENSG0000(	Transcription factor	NA	Familial dilated cardiomyopathy, Muscular disease, Familial isolated dilated cardiomyopathy, Left ventricular noncompaction, Familial restrictive cardiomyopathy, Desminopathy, Aneurysm, Leukemogenesis, Breast Carcinoma, Muscle degeneration	https://www.targetvalidation.org/target/ENSG00000186834/associations	CDK9 PRKCA CCNT1 RELA	Nucleus	No		✓
HEYL	HUMAN Hairy/enhancer-of-split related with YRPW motif-like protein	bHLHb33, HESR3, HEY3	Q9NQ87	ENSG0000(	Transcription factor	BC006087	Narcolepsy with cataplexy, Walker-Warburg syndrome, Autism, Brittle cornea syndrome, Niemann-Pick disease type C, Mammary neoplasm, Prostate carcinoma	https://www.targetvalidation.org/target/ENSG00000163909/associations	HIPK3 SKP1	Nucleus	No		✓
HIF1A	HUMAN Hypoxia-inducible factor 1-alpha	bHLHe78, HIF-1alpha, HIF1, MOP1, PASD8	Q16665	ENSG0000(	Transcription factor,Signalling,Cancer	FJ790247	Adenocarcinoma, Clear cell renal carcinoma, Melanoma, Colorectal adenocarcinoma, Lung carcinoma, Colonic neoplasm, Cutaneous melanoma, Brain ischemia, Mammary neoplasm, Glioblastoma	https://www.targetvalidation.org/target/ENSG00000100644/associations	RWDD3 SENP1 HIF1AN EGLN3 SENP3 PLK3 EGLN1 NAA11 EGLN2 COPS5 CREBBP RPTOR MAPK11 ELOB ELOC MVP CUL2 HDAC1 OS9 MTA1 ATM EP300 MDM2 CSNK2A1 RACK1 ARRB1 CSNK1D NOTCH1 STAT1 NAA10 STAT3	Cytoplasm	Yes	https://www.bindingdb.org/uniprot/Q16665	✓
HIF3A	HUMAN Hypoxia-inducible factor 3-alpha	bHLHe17, IPAS, MOP7, PASD7	Q9Y2N7	ENSG0000(	Transcription factor	AB295039	Li-Fraumeni syndrome, Malignant mesothelioma, Lung carcinoma, Pulmonary hypertension, Pediatric obesity, Sarcoma, Renal cell carcinoma, Steroid sulfatase deficiency disease	https://www.targetvalidation.org/target/ENSG00000124440/associations	HIF1A	Nucleus	No		✓
HINFP	HUMAN Histone H4 transcription factor	DKFZP434F162, HiNF-P, MIZF, ZNF743	Q9BQA5	ENSG0000(	Transcription factor	AP003391	Breast adenocarcinoma, Melanoma, Ataxia-telangiectasia, Prostatic neoplasm, Neuroblastoma, Cervical carcinoma	https://www.targetvalidation.org/target/ENSG00000172273/associations	NPAT PRKDC MBD2 ATM HIST1H4A; HIST1H4B; HIST1H4C; HIST1H4D; HIST1H4E; HIST1H4F; HIST1H4H; HIST1H4I; HIST1H4J; HIST1H4K; HIST1H4L; HIST2H4A; HIST2H4B; HIST4H4 CKS2	Nucleus	No		✓

HIPK1	HUMAN Homeodomain-interacting protein kinase 1	KIAA0630, MYAK, NBAK2	Q86Z02	ENSG0000(	Kinase	AL731797	Azoospermia, Osteosarcoma, Breast carcinoma, Osteocarcinoma, Acute lymphoblastic leukemia, Colorectal carcinoma, Colorectal cancer, Melanoma, Psoriasis, Pancreatic carcinoma	https://www.targetvalidation.org/target/ENSG00000163349/associations	PARK7 PRKACA PRKCD TP53 RPS6KA2 DAXX NKX2-5 MTA1 CHEK1 CAMKK1 SKIL SNIP1 PAGE4 PRPF40A HOXD4	Nucleus, Cytoplasm,	Yes	https://www.bindingdb.org/uniprot/Q86Z02	✓
HIPK4	HUMAN Homeodomain-interacting protein kinase 4	FLJ32818	Q8NE63	ENSG0000(	Kinase	NA	Central nervous system cance, Autism	https://www.targetvalidation.org/target/ENSG00000160396/associations	PRKACA TP53 RPS6KA2 HCLS1 CAMKK1 OPTN ARAP1 ANKS1A GPBP1	Cytoplasm	Yes	https://www.bindingdb.org/uniprot/Q8NE63	✓
HIST1H1C	HUMAN Histone H1.2	H1.2, H1c, H1F2, H1s-1	P16403	ENSG0000(	Others	AB451259	Breast carcinoma, pancreatic carcinoma, Squamous cell carcinoma, Neuroblastoma, Pituitary cancer, Spontaneous abortion, Parkinson's disease, Acute myelocytic leukemia, Mammary Neoplasm	https://www.targetvalidation.org/target/ENSG00000187837/associations	PRKDC CSNK1D CDK1 CHEK2 CDK2	Nucleus, Chromosome,	No		✓
HIST1H4A	HUMAN Histone H4	H4FA, H4FI, H4FG, H4FB, H4FC, H4FJ, H4FH, H4FM, H4FE, H4FD, H4FK	P62805	ENSG0000(	Cancer	X60481	Breast carcinoma, Lung carcinoma, Melanoma, Glioblastoma, Lung adenocarcinoma, Liver carcinoma, Esophageal squamous cell carcinoma, Mammary neoplasm, Central serous chorioretinopathy, Colorectal cancer, Refractory cancer, Idiopathic pulmonary fibrosis	https://www.targetvalidation.org/target/ENSG00000273542/associations	NCOA3 ASF1A HDAC5 PADI4 HDAC6 ASF1B PHPT1 KMT5A KAT8 HDAC8 HINFP MAP3K3 KAT5 KAT2B HDAC2 MAML1 DTX3L KMT5C BRDT KMT5B HIST2H2AC NCOA1 NCOA2 HDAC1 MAP3K1 PAK2 PAK1 EP300 HDAC4	Nucleus, Chromosome,	Yes	https://www.bindingdb.org/uniprot/P62805	✓
HK2	HUMAN Hexokinase-2	HXK2	P52789	ENSG0000(	Kinase,Cancer	AY623118	Renal cell carcinoma, Clear cell renal carcinoma, Glioma, Hepatocellular carcinoma, Central nervous system cancer, Ovarian carcinoma, Psoriasis, Obesity, Myocardial ischemia, Type II diabetes nellitus	https://www.targetvalidation.org/target/ENSG00000159399/associations	PRKCE	Mitochondrion outer membrane	Yes	https://www.bindingdb.org/uniprot/P52789	✓
HLX	HUMAN H2.0-like homeobox protein	HB24, HLX1	Q14774	ENSG0000(	Transcription factor,Cancer,Cancer-Antigen	AB208812	Clear cell renal carcinoma, Malignant mesothelioma, Heart failure, Asthma, Myeloid leukemia, Precursor cell lymphoblastic leukemia lymphoma, Congenital diaphragmatic hernia, Acute leukemia, Systemic lupus eryhtematosus	https://www.targetvalidation.org/target/ENSG00000136630/		Nucleus	No		✓
HMG20B	HUMAN SWI/SNF-related matrix-associated actin-dependent regulator of chromatin subfamily E member 1-related	BRAF35, HMGX2, HMGXB2, SMARCE1r	Q9P0W2	ENSG0000(	Transcription factor,Cancer,Cancer-Antigen	AF288679	Melanoma, Astrocytoma, Glioblastoma multiforme, Lung carcinoma, Glioma, Lung neoplasm, Lung carcinoma, Ovarian carcinoma, Chronic lymphocytic leukemia	https://www.targetvalidation.org/target/ENSG00000064961/associations	BRCA2 KIF4A	Nucleus, Chromosome,	No		✓
HMGB2	HUMAN High mobility group protein B2	HMG2	P26583	ENSG0000(	Transcription factor	AK311864	Melanoma, Breast carcinoma, Glioma, Lung carcinoma, Large cell lung carcinoma, Medulloblastoma, Liver carcinoma, Obesity, Oligospermia, Azoospermia, Degenerative polyarthritis	https://www.targetvalidation.org/target/ENSG00000164104/associations	PRKDC CSNK1A1 GZMA HMGB1 SET RAG1 SYVN1 NSG1	Nucleus	No		✓
HMGN5	HUMAN High mobility group nucleosome-binding domain-containing protein 5	NSBP1	P82970	ENSG0000(	Transcription factor	AL391294	Breast carcinoma, Lung carcinoma, Primary immunodeficiency, Prostate carcinoma, Osteosarcoma, Neoplasm metastasis, Bladder carcinoma, Large cell lung carcinoma, Wiskott-Aldrich syndrome, Glioma	https://www.targetvalidation.org/target/ENSG00000198157/associations	AURKB CDK2 CDK1	Nucleus	No		✓
HNF1B	HUMAN Hepatocyte nuclear factor 1-beta	HNF1beta, LFB3, MODY5, TCF2, VHNF1	P35680	ENSG0000(	Transcription factor	HM116556	Renal cysts and diabetes syndrome, Familial prostate cancer, Chromophobe renal cell carcinoma, Diabetes mellitus, Gout, Renal Cell Carcinoma, Multicystic Dysplastic Kidney	https://www.targetvalidation.org/target/ENSG00000275410/associations	HNF1A CREB1 HDAC1 ATF1 CREBBP	Nucleus,	No		✓

HNRNPA2B1	HUMAN Heterogeneous nuclear ribonucleoproteins A2/B1	HNRPA2B1, ROA2	P22626	ENSG0000(	Cancer	D28877	Inclusion body myopathy with Paget disease of bone and frontotemporal dementia, Adenocarcinoma, Breast carcinoma, Colorectal adenocarcinoma, Lung carcinoma, Kidney neoplasm, Myopathy, Muscular Dystrophy, Myositis	https://www.targetvalidation.org/target/ENSG00000122566/associations	PRMT1 CSNK2A1 HNRNPL SNRNP70 ISG15 CHEK1 VHL SET RBM3	Nucleus, nucleoplasm	No	✓
HNRNPUL1	HUMAN Heterogeneous nuclear ribonucleoprotein U-like protein 1	E1B-AP5, E1BAP5, HNRPUL1, HNRL1	Q9BUJ2	ENSG0000(	Cancer,Cancer-Antigen	AK127057	Large cell lung carcinoma, Lung adenocarcinoma, Renal cell carcinoma, Breast carcinoma, Psoriasis, Myocardial infarction, Coronary heart disease, Familial hypercholesterolemia, Adenovirus infections, Metabolic syndrome X	https://www.targetvalidation.org/target/ENSG00000105323/associations	RICTOR PRMT2 MAPK9 CDK2 TP53	Nucleus	No	✓
HOMER2	HUMAN Isoform 2 of Homer protein homolog 2	Cupidin, HOMER-2, HOMER-2A, HOMER-2B	Q9NSB8	ENSG0000(	Others	Y19026	Autosomal dominant non-syndromic sensorineural deafness type DFNA, Asperger syndrome, Renal cell carcinoma, Breast carcinoma, Schizophrenia, Cocaine-related disorders, Alzheimer's Disease, Ischemic stroke, Familial multiple trichoepitheliomata	https://www.targetvalidation.org/target/ENSG00000103942/associations	SHANK1 DBN1 ITPR1 GRM1 DNM1 UBXN1 GRIA1 DNM1 RYR1 GRM1 SHANK1 GRIA1	Cytoplasm	No	✓
HOOK1	HUMAN Protein Hook homolog 1	HK1	Q9UJC3	ENSG0000(	Kinase,Cancer,Cancer-Antigen		Azoospermia, Breast carcinoma, Astrocytoma, Glioblastoma multiforme, Ependymoma, Lung carcinoma, Alzheimer's disease, Senile plaques, Juvenile neuronal ceroid lipofuscinosis	https://www.targetvalidation.org/target/ENSG00000134709/associations	ANK3	Cytoplasm	No	✓
HOPX	HUMAN Homeodomain-only protein	HOD, HOP, LAGY, NECC1, OB1	Q9BPY8	ENSG0000(	Transcription factor	AF492678	Squamous cell carcinoma, Esophageal squamous cell carcinoma, Romano-ward syndrome, Atria fibrillation, Dilated cardiomyopathy with ataxia, Glioblastoma, Lung carcinoma, Gastric carcinoma, Heart failure, Brugada syndrome	https://www.targetvalidation.org/target/ENSG00000171476/associations	HDAC2 SRF EPC1	Nucleus	No	✓
HORMAD1	HUMAN HORMA domain-containing protein 1	HORM1, CT46,	Q86X24	ENSG0000(	Others	AK097390	Tongue squamous cell carcinoma, Lung carcinoma, Lung adenocarcinoma, Young syndrome, Kallmann syndrome, Primary ovarian insufficiency, Deafness-infertility syndrome, Breast carcinoma, Melanoma	https://www.targetvalidation.org/target/ENSG00000143452/associations		Nucleus	No	✓
HOXA1	HUMAN Homeobox protein Hox-A1	HOX1, HOX1F	P49639	ENSG0000(	Transcription factor	CH236948	Bosley-Salih-Alorainy syndrome, Athabaskan brainstem dysgenesis syndrome, Autistic Disorder, craniofacial abnormalities, Abnormal ocular motility, Congenital heart defects, Brainstem abnormalities	https://www.targetvalidation.org/target/ENSG00000105991/associations	PKNOX1	Nucleus,	No	✓
HOXA9	HUMAN Homeobox protein Hox-A9	HOX1, HOX1G	P31269	ENSG0000(	Kinase,Transcription factor,Cancer	AC004080	Prostate carcinom, Colorectal adenocarcinoma, Breast ductal adenocarcinoma, Cutaneous melanoma, Acute myelocytic leukemia, Bladder neoplasm, Melanoma, Leukemia, Chronic myeloid leukemia	https://www.targetvalidation.org/target/ENSG00000078399/associations	SMAD4 KMT2A PRMT5 SMAD4 CUL4A TRIP6	Nucleus,	No	✓
HOXB13	HUMAN Homeobox protein Hox-B13	HXB13	Q92826	ENSG0000(	Transcription factor	AK313266	Prostate adenocarcinoma, Adenocarcinoma, Li-Fraumeni syndrome, Central nervous system cancer, Glioblastoma miltiforme, Familial prostate carcinoma, Neoplasm invasiveness, Breast carcinoma	https://www.targetvalidation.org/target/ENSG00000159184/associations		Nucleus,	No	✓

HOXB6	HUMAN Homeobox protein Hox-B6	HOX2, HOX2B	P17509	ENSG0000(	Transcription factor	AF287967	Central nervous system cancer, Tongue squamous cell carcinoma, Autosomal recessive spondylocostal dysostosis, Lymphoma, Hypospadias, Neurofibromatosis 1, Pancreatic intraepithelial neoplasia, Rhabdomyosarcoma, Pancreatic carcinoma, Acute myelocytic leukemia	https://www.targetvalidation.org/target/ENSG00000108511/associations	CSNK2B PKNOX1 CREBBP	Nucleus,		No		✓
HOXB7	HUMAN Homeobox protein Hox-B7	HOX2, HOX2C	P09629	ENSG0000(	Transcription factor	AC103702	Prostate carcinoma, Tongue squamous cell carcinoma, Central nervous system cancer, Greenberg dysplasia, Ovarian carcinoma, Thoracolaryngopelvic dysplasia, Rosacea, Hirschsprung Disease, Bile duct neoplasm, Glioblastoma	https://www.targetvalidation.org/target/ENSG00000260027/associations	CSNK2A1 CSK CSNK2A2 PRKDC PKNOX1 NFKBIA CREBBP	Nucleus,		No		✓
HOXC10	HUMAN Homeobox protein Hox-C10	HOX3I	Q9NYD6	ENSG0000(	Transcription factor	X99685	Tongue squamous cell carcinoma, Central nervous system cancer, Short rib-polydactyly syndrome, Verma-Naumoff type, Fibrochondrogenesis, Metatropic dysplasia, Colorectal adenocarcinoma, Aortic valve insufficiency, Thyroid carcinoma, Obesity	https://www.targetvalidation.org/target/ENSG00000180818/associations	CDC27 EGFR	Nucleus,		No		✓
HOXC8	HUMAN Homeobox protein Hox-C8	HOX3A	P31273	ENSG0000(	Transcription factor	BC053898	Central nervous system cancer, Cervical carcinoma, Waardenburg syndrome type 1, Cervical carcinoma, Prostate neoplasm, Breast carcinoma, Mammary neoplasm, Thoracolaryngopelvic dysplasia, Multiple synostoses syndrome, Orofaciodigital syndrome type 11	https://www.targetvalidation.org/target/ENSG00000037965/associations	HELLS SMAD1 KMT2A BMPR1A SMAD6 MEN1 HOMEZ	Nucleus,		No		✓
HPCAL1	HUMAN Hippocalcin-like protein 1	BDR1, HLP2, VILIP-3	P37235	ENSG0000(	Others	BC017482	Melanoma, Ovarian carcinoma, Colorectal adenocarcinoma, Pituitary cancer, Lung carcinoma, Breast carcinoma, Clear cell renal carcinoma, Cystic fibrosis, Oligodendroglioma, Astrocytoma, Congenital central hypoventilation	https://www.targetvalidation.org/target/ENSG00000115756/associations	IKBKG SLC34A1	Membrane		No		✓
HPRT1	HUMAN Hypoxanthine-guanine phosphoribosyltransferase	HGPRT, HPRT	P00492	ENSG0000(	Signalling	BT019350	Inborn errors of metabolism, Disorder of purine metabolism, Lesch-Nyhan syndrome, Hypoxanthine guanine phosphoribosyltranferase partial deficiency, MODY, Type I diabetes mellitus, Hyperuricemia, Gout, Choreoathetosis, Nephrolithiasis	https://www.targetvalidation.org/target/ENSG00000165704/associations		Cytoplasm,		Yes	https://www.bindingdb.org/uniprot/P00492	✓
HRAS	HUMAN GTPase HRas	HRAS1, RASH	P01112	ENSG0000(	Cancer	CR536579	Costello syndrome, Epithelial neoplasm, Nevus sebaceous, Papilloma, Thyroid neoplasm, Bladder neoplasm, Melanocytic nevus, Liver carcinoma, Squamous cell carcinoma, Noonan syndrome	https://www.targetvalidation.org/target/ENSG00000174775/associations	TRAF6 RAPGEF2 ZDHHC9 SHOC2 PLCE1 TTC1 ERBIN RAPGEF5 RAPGEF6 RASGRP4 RASGEF1A RASGEF1C RASIP1 RIN1 DGKZ TIAM1 RASGEF1B SOS2 SOS1 PTPN11 PRKCZ MAP2K1 GPSM2 GRB2 IRAK1 GNAQ GSK3B FNTB	Cell membrane, Lipid-anchor, Cytoplasmic side, Golgi apparatus, Golgi apparatus membrane,		Yes	https://www.bindingdb.org/uniprot/P01112	✓
HRH1	HUMAN Histamine H1 receptor	HRH1	P35367	ENSG0000(	Others	D28481	Allergic rhinitis, Asthma, Eczema, Nasal obstruction, Cardiac arrhythmia, Parkinson's disease, Vasomotor rhinitis, Fibromyalgia, Atopic eczema, Schizophrenia, Pruritus	https://www.targetvalidation.org/target/ENSG00000196639/associations	CAMK2A PRKG1 CSNK2A1 PRKX GRK2 PRKACA PRKCA PRKY	Cell membrane	Transmembrane	Yes	https://www.bindingdb.org/uniprot/P35367	✓

HRH2	HUMAN Histamine H2 receptor	H2R, HH2R	P25021	ENSG0000(	Others	AB041384	Arthralgia, Myalgia, Pneumonia, Schizophrenia, Gastrointestinal hemmorhage, Gastroesophageal reflux disease, Pneumonia, Peptic ulcer, Coronary stenosis, Hyperemia	https://www.targetvalidation.org/target/ENSG00000113749/associations		Cell membrane, Multi-pass membrane protein,	Transmembrane	Yes	https://www.bindingdb.org/uniprot/P25021	✓
HSBP1	HUMAN Heat shock factor-binding protein 1	HSBP1	O75506	ENSG0000(	Transcription factor	BC007515	Teratozoospermia, Breast carcinoma, Sarcoma, Medulloblastoma, Psoriasis, Mammary neoplasm, Rheumatoid arthritis, Hearing loss, Microcephaly, Oral squamous cell carcinoma	https://www.targetvalidation.org/target/ENSG00000230989/associations	HSF1	Nucleus,		No		✓
HSF1	HUMAN Heat shock factor protein 1	HSTF1	Q00613	ENSG0000(	Transcription factor,Cancer	AC110280	Leukemia, Ischemia, Neoplasm of mature B-cells, Chronic lymphocytic leukemia, Mental depression, Cardiomegaly, Cryptorchidism, Heat stroke, Heart failure, Transient ischemic attack	https://www.targetvalidation.org/target/ENSG00000185122/associations	CAMK2A NLK NEK6 DYRK2 MAPK15 MAPK6 CAMK4 CAMK2D MAPK7 PRKCQ PRKDC CSNK2A1 YWHAE MAPK10 DAPK1 PLK1 SMARCA4 PPT1 GSK3B GSK3A MAPKAPK 2 MAPK9 MAPK8 MTOR STAT1 MAP2K2 MAPK4 MAPK1 MAPK3 DNAJB1	Nucleus		Yes	https://www.bindingdb.org/uniprot/Q00613	✓
HSFY1	HUMAN Heat shock transcription factor, Y-linked	HSF2L, HSFY	Q96LI6	ENSG0000(	Transcription factor	AK058182	Azoospermia, Sertoli cell-only syndrome, Male infertility, Oligospermia	https://www.targetvalidation.org/target/ENSG00000169953/associations		Nucleus		No		✓
HSP90AA1	HUMAN Heat shock protein HSP 90-alpha	HSPC1, HSPCA, HSP90A,	P07900	ENSG0000(	Cancer	AJ890083	Diphtheria, HIV infections, Mammary neoplasm, Ki-1+ Anaplastic large cell lymphoma, Contact dermatitis, Unipolar depression, Bladder carcinoma, Melanoma, Prostate carcinoma, Influenza infection	https://www.targetvalidation.org/target/ENSG00000080824/associations	RIPK3 SUGT1 ALK TBK1 HDAC6 EIF2AK3 EIF2AK1 MAP3K3 DNAJC7 MAP3K14 NLRP3 PNCK CDC37 PTGES3 IKBKE RIPK1 MAP3K1 PPARA PRKDC CSNK2A1 YWHAE CALM1 IRAK1 ZAP70 PAFAH1B1 MAP3K8 STAT3 KDR AHR GRK5	Cytoplasm		Yes	https://www.bindingdb.org/uniprot/P07900	✓
HSP90AB1	HUMAN Heat shock protein HSP 90-beta	HSPC2, HSPCB, HSP90B	P08238	ENSG0000(	Kinase,Cancer	AL136543	Diphtheria, Melanoma, Adenocarcinoma, Gastrointestinal stromal tumor, Kidney neoplasm, Lung carcinoma, HIV infections, Pulmonary fibrosis, Asthma, Breast carcinoma	https://www.targetvalidation.org/target/ENSG00000096384/associations	HSPA4 RIPK3 TBK1 PLK3 MAP3K3 MAP3K14 AURKB PNCK IKBKE RIPK1 MAP3K1 CSNK2A1 MAP3K8 STAT3 AKT2 AKT1 CSNK2A2 SRC CDK4 HSP90AA1 CDK1 TP53 NR3C1 ESR1 AHSA1 CHUK IKBKB CHEK1 APAF1 FKBP4 NOS3 STUB1	Cytoplasm		Yes	https://www.bindingdb.org/uniprot/P08238	✓
HSPA1A	HUMAN Heat shock 70 kDa protein 1A	HSP72, HSPA1, HSX70	P0DMV8	ENSG00000204389-HSI		AF134726	Influenza infection, neoplasm, breast carcinoma, ovarian carcinoma, hepatocellular carcinoma, prostate carcinoma, metastatic prostate cancer, adenocarcinoma, breast carcinoma	https://www.targetvalidation.org/target/ENSG00000204389/associations	HSPBP1 NEK6 BAG1 MAP3K5 TTC1 DNAJA3 CDK9 EIF2AK2 PRKACA PRKCA HSPA8 KRT18 APOB MOS AIFM1 DNAJA2 TRIM24 APAF1 RHOA AIFM1 NOD2 TP53 DNAJB1 DCLRE1B SOX9 BCL2 HSF1	Cytoplasm		Yes	https://www.bindingdb.org/uniprot/P0DMV8	✓
HSPA5	HUMAN 78 kDa glucose-regulated protein	BiP, GRP78	P11021	ENSG0000(	Others	AF216292	Melanoma, Renal cell carcinoma, Heart disease, LCAT deficiency, Nephronophthisis, Liver carcinoma, Obesity, Myocardial infarction, Asbestosis, Respiratory Hypersensitivity	https://www.targetvalidation.org/target/ENSG00000044574/associations	NR3C1 RIPK3 TBK1 SEC63 EIF2AK3 MAP3K3 MAP3K14 RIPK1 MAP3K1 E2F1 MAP3K8 IL7R CLU RPN1 IGHM TG LDLR F8 ERN1 AIRE MAP3K7 IKBKB EIF2AK3 ATF6 CASP7 ERN2 PAK2 ERN1 SEC61A1 ERP29 PCSK7 AMFR SIL1 PRNP	Endoplasmic reticulum lumen		Yes	https://www.bindingdb.org/uniprot/P11021	✓
HSPB8	HUMAN Heat shock protein beta-8	CMT2L, E2IG1, H11, HSP22, HspB8	Q9UJY1	ENSG0000(	Kinase	AL136936	Charcot-Marie-Tooth disease, Distal hereditary motor neuropathy, Autosomal dominant distal hereditary motor neuropathy, Distal hereditary motor neuropathy type 2, Neurodegenerative disease, Curvature of spine, Distal sensory impairment, Areflexia of lower limbs	https://www.targetvalidation.org/target/ENSG00000152137/associations	PRKCE ZAP70 MAPK3 PRKACA PRKCA PRKCB PRKCG CSNK2A1 CRYAB HSPB2 HSPB6 HSPB1 MAPK3 BAG3 LNX1 HSPB7 HSPB3	Cytoplasm		No		✓
HSPB9	HUMAN Heat shock protein beta-9	CT51	Q9BQS6	ENSG0000(	Others	BC093991	Azoospermia, Lung carcinoma, Malignant neoplasm of testis	https://www.targetvalidation.org/target/ENSG00000260325/associations	DYNLT1	Cytoplasm		No		✓

HSPD1	HUMAN 60 kDa heat shock protein, mitochondrial	HSP60, SPG13, CH60	P10809	ENSG0000(	Cancer,Cancer-Antigen	DQ217936	Hereditary spastic paraplegia, Autosomal dominant spastic paraplegia type 13, Leukodystrophy, Spastic paraplegia, Acute coronary syndrome, HIV infection, Colorectal neoplasm, Tuberculosis, Adenocarcinoma, Monoparesis on leg , Sphincter disturbances	https://www.targetvalidation.org/target/ENSG00000144381/associations	TLR1 HSPE1 PRKACA IL7R HSP90AA1 ISG15 ESR1 CA2 DHFR IKBKB PRNP SIRT3 BAK1 CASP3 BAX ISG15 CHUK RASA1 TMEM11 CLU MFHAS1	Mitochondrion matrix,		No		✓
HSPE1	HUMAN 10 kDa heat shock protein, mitochondrial	CPN10, EPF, HSP10	P61604	ENSG0000(	Cancer	AK312104	Breast carcinoma, Abortion, Cardiomyopathy, Hereditary spastic paraplegia, neoplasm metastasis, Colorectal neoplasm, Tuberculosis, Myobacterium infection, Central neuroblastoma	https://www.targetvalidation.org/target/ENSG00000115541/associations	TRIM24 HSPD1	Mitochondrion matrix,		No		✓
HTATIP2	HUMAN Oxidoreductase HTATIP2	CC3, TIP30, HTAI2	Q9BUP3	ENSG0000(	Transcription factor	AK292092	Li-Fraumeni syndrome, Breast carcinoma, Central nervous system cancer, Gestational trophoblastic neoplasm, Synovial sarcoma, Leiomyosarcoma, Multiple sclerosis, Hepatocarcinogenesis, Non-small cell lung carcinoma, Liver carcinoma	https://www.targetvalidation.org/target/ENSG00000109854/associations	ETS1 TP53 NCOA5	Cytoplasm		No		✓
HTR1D	HUMAN 5-hydroxytryptamine receptor 1D	5-HT1D, HTR1DA, HTRL	P28221	ENSG0000(	Others	BT007027	Migraine disorder, Schizophrenia, Autistic disorder, Depressive disorder, Hypotension, Obsessive-compulsive disorder, Anorexia nervosa, Attention deficit hyperactivity disorder, Chronic obstructive pulmonary disease, Weight gain adverse event	https://www.targetvalidation.org/target/ENSG00000179546/associations		Cell membrane	Transmembrane	Yes	https://www.bindingdb.org/uniprot/P28221	✓
HTR2B	HUMAN 5-hydroxytryptamine receptor 2B	5-HT(2B), 5-HT2B	P41595	ENSG0000(	Others	AY136751	Migraine disorder, Hemorrhage, Schizophrenia, Cardiomegaly, Pulmonary Hypertension, Hypersensitivity, Irritable Bowel Syndrome, Heart failure, Heart valve disease, Atopic eczema	https://www.targetvalidation.org/target/ENSG00000135914/associations	GNAQ GNA11	Cell membrane	Transmembrane	Yes	https://www.bindingdb.org/uniprot/P41595	✓
HUS1	HUMAN Checkpoint protein HUS1	HUS1	O60921	ENSG0000(	Signalling	AY294117	Neural tube closure defect, Isolated spina bifida, Spina bifida-hypospadias, Fanconi anemia, Seckel syndrome, Amyotrophic lateral sclerosis, Breast carcinoma, Lung neoplasm, Papillary thyroid carcinoma	https://www.targetvalidation.org/target/ENSG00000136273/associations	RAD9A RAD9B YWHAZ RPA1 RPA2 RAD17 RPA1 RAD17 PCNA RAD9A RAD1 RPA2	Nucleus		No		✓
ID1	HUMAN DNA-binding protein inhibitor ID-1	bHLHb24, dJ857M17.1.2	P41134	ENSG0000(	Transcription factor,Signalling,Cancer	S78986	Lung carcinoma, Squamous cell carcinoma, Hepatocellular carcinoma, Gaucher disease, Type II diabetes mellitus, Myocardial ischemia, Cholestasis, Prostatic neoplasm, Mammary neoplasm, Thyroid neoplasm, Melanoma	https://www.targetvalidation.org/target/ENSG00000125968/associations	HES1 ELK4 ELK1 TCF3 MYOD1 CASK TCF3 MYOD1 PSMD4 ATOH1 TCF4 IFI16 CASK MYOG MYF5 TCF12 MYF6	Cytoplasm Nucleus,		No		✓
ID2	HUMAN DNA-binding protein inhibitor ID-2	bHLHb26, GIG8	Q02363	ENSG0000(	Transcription factor,Signalling,Cancer	NA	Breast carcinoma, Lung carcinoma, Acute myelocytic leukemia, Dermatologic disorder, Thyroid disease, Small cell lung carcinoma, Arsenic poisoining, Neuroblastoma, Hodgkin's disease, T-cell lymphoma	https://www.targetvalidation.org/target/ENSG00000115738/associations	HES1 ZBTB16 ELK3 ADD1 ELK4 CDK2 ELK1 TCF3 TCF4 MYOD1 TP53 MSC PKD2 TCF3 PAX2 MYOD1 RB1 RBL2 PAX8 RBL1 PAX5 ATOH1 FZR1 TCF4 IFI16 MYOG MYF5 ASB4 TCF12 MYF6	Cytoplasm		No		✓

IDI1	HUMAN Isopentenyl-diphosphate Delta-isomerase 1	IDI1, IPPI1	Q13907	ENSG0000(	Cancer,Cancer-Antigen	AF271724	Large cell lung carcinoma, Breast carcinoma, Colorectal adenocarcinoma, Pancreatic carcinoma, Hepatocellular carcinoma, Glioblastoma multiforme, Endometrial neoplasm, Acute lymphoblastic leukemia, Alzheimer's disease	https://www.targetvalidation.org/target/ENSG00000067064/associations	CHEK1	Peroxisome		Yes		✓
													https://www.drugbank.ca/biodb/polypeptides/Q13907	
IDO1	HUMAN Indoleamine 2,3-dioxygenase 1	IDO, INDO, I23O1	P14902	ENSG0000(	Signalling, Cancer	M86473	Spondyloarthropathy, Inflammatory skin disease, Central nervous system cancer, Ulcerative colitis, Renal carcinoma, Hypertriglyceridemia, Autoimmune disease, Endometriosis, Chronic depression, Mammary neoplasm	https://www.targetvalidation.org/target/ENSG00000131203/associations	SOCS3	Cytoplasm, cytosol		Yes		✓
													https://www.bindingdb.org/uniprot/P14902	
IFI16	HUMAN Gamma-interferon-inducible protein 16	IFNGIP1, IFI16	Q16666	ENSG0000(	Transcription factor,Cancer,Cancer-Antigen	AY138863	Breast carcinoma, Autoimmune disease, Systemic lupus erythematosus, Squamous cell carcinoma, Herpes simplex infection, Ovarian neoplasm, Prostate neoplasm, Lung adenocarcinoma, Pancreatic adenocarcinoma, Cytomegalovirus infection, Juvenile dermatomyositis	https://www.targetvalidation.org/target/ENSG00000163565/associations	PYCARD TMEM173 TP53BP1 ID3 ID2 CSNK2A1 GSK3B ID1 BRCA1 CASP1 CSNK2A2 AR SP1 TP53 TMEM173 MB21D1	Nucleus, Cytoplasm,		No		✓
IFI35	HUMAN Interferon-induced 35 kDa protein	IFP35, IN35	P80217	ENSG0000(	Interleukin-Interferon	L78833	Hepatitis C infection, Psoriasis, Asthma, Colorectal adenocarcinoma, Multiple sclerosis, Spontaneous abortion, Ovarian neoplasm, Rheumatoid arthritis, Vesicular stomatitis, Influenza	https://www.targetvalidation.org/target/ENSG00000068079/associations	NMI	Nucleus,		No		✓
IFIT1	HUMAN Interferon-induced protein with tetratricopeptide repeats 1	G10P1, GARG-16, IFI56, IFNAI1	P09914	ENSG0000(	Interleukin-Interferon	AK314588	Breast carcinoma, Hepatitis C infection, Li-Fraumeni syndrome, Asthma, Colorectal carcinoma, Endometriosis, Ovarian neoplasm, Ebola hemorrhagic fever, Myelodysplastic syndrome, Systemic lupus erythemetosus	https://www.targetvalidation.org/target/ENSG00000185745/associations	TMEM173 IRF3 ISG15 EIF3E	Cytoplasm		No		✓
IFIT5	HUMAN Interferon-induced protein with tetratricopeptide repeats 5	RI58	Q13325	ENSG0000(	Interleukin-Interferon	CR457031	Lung carcinoma, Sepsis, Lung adenocarcinoma, Endometrial neoplasm, Asthma, Dermatomyositis, Influenza, Lymphocytosis, Psoriasis, Juvenile dermatomyositis	https://www.targetvalidation.org/target/ENSG00000152778/associations	ISG15	Cell projection, ruffle membrane		No		✓
IFITM1	HUMAN Interferon-induced transmembrane protein 1	9-27, CD225, IFI17, IFM1	P13164	ENSG0000(	Interleukin-Interferon	AK290480	Lung carcinoma, Lung adenocarcinoma, Atopy, Wiskott-Aldrich syndrome, Asthma, Colonic neoplasm, Esophageal neoplasm, Hepatitis C infection, Ovarian neoplasm, Colorectal cancer, Colorectal carcinoma	https://www.targetvalidation.org/target/ENSG00000185885/associations	CR2 CD81	Cell membrane	Transmembrane	No		✓
IGF2	HUMAN Insulin-like growth factor II	PP1446, IGF-II	P01344	ENSG0000(	Cancer	X03425	Silver-Russell syndrome, Liver carcinoma, Nephroblastoma, Adrenocortical carcinoma, Hepatoblastoma, Congenital hemihypertrophy, Alzheimer's disease, Beckwith-Wiedemann Syndrome, Obesity, Meckel syndrome	https://www.targetvalidation.org/target/ENSG00000167244/associations	GPC3 IGFBP4 EGR1 IGF2R IGF1R INSR PLG IGFBP4 IGF1R IGFBP1 INSR IGFBP3 IGF2R VTN IGFBP5 IGFBP2 IGFBP6 PCSK4	Secreted		No		✓
IGF2BP1	HUMAN Insulin-like growth factor 2 mRNA-binding protein 1	IF2B1, CRDBP, VICKZ1, ZBP1	Q9NZI8	ENSG0000(	Others	AC105030	Odontogenesis, Tooth eruption, Melanoma, Meningioma, Mammary Neoplasm, Lung Neoplasm, ovarian neoplasm, Lymphoma, Atherosclerosis, Obesity	https://www.targetvalidation.org/target/ENSG00000159217/associations	BIRC2 CTNNB1 ELAVL4 BTRC	Nucleus, Cytoplasm, perinuclear region, Cell projection, lamellipodium, Cell projection, dendrite		No		✓

IGF2BP3	HUMAN Insulin-like growth factor 2 mRNA-binding protein 3	IMP-3, IMP3, IF2B3, KOC1, VICKZ3	O00425	ENSG0000(	Others	BX648488	Breast carcinoma, Central nervous system cancer, Glioma, Hepatocellular carcinoma, Peritoneal neoplasm, Malignant mesothelioma, Neuroendocrine carcinoma, Liver carcinoma, Osteosarcoma, Endometrioid carcinoma	https://www.targetvalidation.org/target/ENSG00000136231/associations		Nucleus, Cytoplasm,		No		✓
IGHG1	HUMAN Immunoglobulin heavy constant gamma 1	IGHG1	P01857	ENSG0000(	Others	NA	Amyloidosis, Primary systemic amyloidosis, AL amyloidosis, Polycystic ovary syndrome, Chronic Lymphocytic leukemia, Myositis, Sarcoidosis, Crohn's disease, Polymyositis, Sjogren's syndrome	https://www.targetvalidation.org/target/ENSG00000211896/associations	IGKC IGLV2-8 FCGR3B FCGR3A TRIM21 FCGR2B IGLC1 SWAP70	Secreted		Yes	https://www.drugbank.ca/biodb/polypeptides/P01857	✓
IGSF11	HUMAN Immunoglobulin superfamily member 11	BT-IgSF, VSIG3, CXADRL1	Q5DX21	ENSG0000(	Others	AC012032	Clear cell renal carcinoma, Lung carcinoma, Melanoma, Adrenocortical carcinoma, Parkinson's disease, Gastric carcinoma, Liver carcinoma, Malignant neoplasm of testis, Neuroblastoma	https://www.targetvalidation.org/target/ENSG00000211896/associations		Cell membrane	Transmembrane	No		✓
IKBKB	HUMAN Inhibitor of nuclear factor kappa-B kinase subunit beta	IKK-beta, IKK2, IKKB, NFKBIKB	O14920	ENSG0000(	Kinase,Cancer	AK302723	Primary immunodeficiency due to a defect in adaptive immunity, Severe combined immunodeficiency, Severe combined immunodeficiency due to IKK2 deficiency, Immunologic deficiency syndrome, Chronic diarrhea, Infantile onset, Respiratory tract infection, Agammaglobulinemia, Autosomal recessive predisposition, Alzheimer's disease	https://www.targetvalidation.org/target/ENSG00000104365/associations	NCOA3 IKBKG TRAF6 NOD1 PYCARD TBK1 STAP2 NLRP2 NOD2 CUEDC2 MLST8 MAP3K3 MAP3K14 PPM1M DNAJA3 TSC1 PYDC1 TRPC4AP ERC1 TICAM1 MAVS CDC37 NFKBIB CASP8 IKBKE KEAP1 RIPK1 SQSTM1 MAP3K1	Cytoplasm		Yes	https://www.bindingdb.org/uniprot/O14920	✓
IKBKE	HUMAN Inhibitor of nuclear factor kappa-B kinase subunit epsilon	IKK-i, IKKE, KIAA0151	Q14164	ENSG0000(	Kinase	DQ667176	Primary ciliary dyskinesia, Familial hyperinsulinism, Non-acquired isolated growth hormone deficiency, Isolated growth hormone deficiency type III, Isolated agammaglobulinemia, Unipolar depression, Rheumatoid arthritis, Vesicular stomatitis, Degenerative polyarthritis, Squamous cell carcinoma, Breast carcinoma	https://www.targetvalidation.org/target/ENSG00000263528/associations	IKBKG MAFB TBK1 AZI2 TRIM6 IFIH1 SIKE1 DHX58 IRF7 TANK CISD2 TICAM1 TMEM173 KTN1 MAVS CDC37 IRF3 FKBP5 TRAF3 CSNK2A1 TNFAIP3 TRIM27 HSP90AB1 HSP90AA1 DDX58 TRAF3IP2 CHUK IKBKB DDX3X TBKBP1 CUL1	Cytoplasm		Yes	https://www.bindingdb.org/uniprot/Q14164	✓
IKZF1	HUMAN DNA-binding protein Ikaros	IK1, IKAROS, LYF1, ZNFN1A1	Q13422	ENSG0000(	Transcription factor,Cancer	AK303586	Primary immunodeficiency, Pancytopenia due to IKZF1 mutations, Acute lymphoblastic leukemia, Precursor cell lmphoblastic leukemia lymphoma, Systemic lupus erythematosus, Nikolsky sign, Skin bulla, Stevens-Johnsons syndrome, Precursor B-Cell lymphoblastic leukemia-lymphoma, Melanoma	https://www.targetvalidation.org/target/ENSG00000185811/associations	IKZF2 BTK CSNK2A1 CSNK2B PPP1CB PPP1CA SYK PPP1CC IKZF3 IKZF4 CRBN	Nucleus		No		✓
IKZF3	HUMAN Zinc finger protein Aiolos	Aiolos, ZNFN1A3	Q9UKT9	ENSG0000(	Transcription factor	AJ292567	Inflammatory bowel disease, Ulcerative colitis, Crohn's disease, Biliary liver cirrhosis, Asthma, Rheumatoid arthritis, Chronic lymphocytic leukemia, Systemic lupus erythematosus, Colorectal cancer metastatic, Multiple myeloma	https://www.targetvalidation.org/target/ENSG00000161405/associations	FOXP3 IKZF1 BCL2L1 GRB2 HRAS	Nucleus, Cytoplasm,		No		✓
IL13RA1	HUMAN Interleukin-13 receptor subunit alpha-1	CD213a1, IL13Ra, II13R1, IL13R	P78552	ENSG0000(	Interleukin-Interferon	U81379	Central nervous system cancer, Melanoma, Glioma, Asthma, Astrocytoma, Atopic dermatitis, Viral bronchitis, Secretory diarrhea, Respiratory syncytial virus infection, Psoriasis	https://www.targetvalidation.org/target/ENSG00000131724/associations	TRAF3IP1 IL13 IL2RG TYK2 IL4R IL4 JAK2 JAK2 STAT6 IL4R TYK2 IL2RG	Membrane, Single-pass type I membrane protein,	Transmembrane	No		✓
IL13RA2	HUMAN Interleukin-13 receptor subunit alpha-2	CD213a2, CT19, IL-13R	Q14627	ENSG0000(	Cancer,Interleukin-Interferon	AK291957	Breast carcinoma, Li-Fraumeni syndrome, Glioma, Astrocytoma, Systemic scleroderma, Asthma, Psoriasis, Glioblastoma, Ovarian neoplasm, Infection of amniotic sac and membrane	https://www.targetvalidation.org/target/ENSG00000123496/associations	IL13 IL4	Membrane, Single-pass type I membrane protein,	Transmembrane	Yes	https://www.drugbank.ca/biodb/polypeptides/Q14627	✓

IL18	HUMAN Interleukin-18	IGIF, IL-18, IL-1g, IL1F4	Q14116	ENSG0000	Signalling, Cancer,Interleukin-Interferon, Cytokine/Chemokine	AY044641	Metabolic syndrome X, Rheumatoid arthritis, Sarcoidosis, Neoplasm metastasis, Immediate hypersensitivity, Brain ischemia, Schizophrenia, Prostatic neoplasm, Cardiomegaly, Hypersensitivity	https://www.targetvalidation.org/target/ENSG00000150782/associations	CASP5 CASP4 CASP3 CASP1 PRTN3 IL18BP CASP1 PRTN3 IL18R1 IL18BP IL18RAP	Secreted		No		✓
IL1A	HUMAN Interleukin-1 alpha	IL-1A, IL1, IL1-ALPHA, IL1F1	P01583	ENSG0000	Kinase,Transcription factor,Cancer,Interleukin-Interferon, Cytokine/Chemokine	X56086	Heat stroke, Inflammation, Schizophrenia, Dermatomyositis, Chronic alcoholic intoxication, Dermatologic disorder, Anthracosis, Chorioamnionitis, Brain ischemia, Mental depression	https://www.targetvalidation.org/target/ENSG00000115008/associations	CASP1 IL1R2 IL1R1 CAPN1 NFKBIE HAX1 IL1R2 IL1R1 S100A13 NDN IL1RAP	Secreted		Yes		✓
													https://www.drugbank.ca/biodb/polypeptides/P01583	
IL1B	HUMAN Interleukin-1 beta	IL-1B, IL1-BETA, IL1F2	P01584	ENSG0000	Cancer,Interleukin-Interferon, Cytokine/Chemokine	X04500	Alzheimer's disease, Inflammation, Degenerative polyarthritis, Rheumatoid arthritis, Gastric neoplasm, Myocardial infarction, Hypertensive disease, Pulmonary fibrosis, Colitis, Hyperalgesia	https://www.targetvalidation.org/target/ENSG00000125538/associations	IL1RAP NLRP7 NLRP10 CASP5 CASP1 IL1R2 IL1R1 GZMA A2M IL1R2 HMGB1 IL1R1 MMP2 ADRB2 UMOD ALPP TRIM16L IL1RAP	Cytoplasm, cytosol		Yes		✓
													https://www.bindingdb.org/uniprot/P01584	
IL1F5	HUMAN Interleukin-36 receptor antagonist protein	IL36RN, FIL1D, IL1HY1, IL1L1, IL1RP3	Q9UBH0			AJ271338								✓
IL1RN	HUMAN Interleukin-1 receptor antagonist protein	ICIL-1RA, IL-1RN, IL1F3, IL1RA	P18510	ENSG0000	Cancer,Interleukin-Interferon, Cytokine/Chemokine	X64532	Sterile multifocal osteomyelitis with periostitis and pustulosis, Diabetic nephropathy, Gastric neoplasm, Anorexia nervosa, Hyperalgesia, Rheumatoid arthritis, Inflammation, Asthma, Cerebrovascular accident, Brain ischemia	https://www.targetvalidation.org/target/ENSG00000136689/associations	IL1R2 IL1R1 IL1R2 IL1R1	Secreted, Cytoplasm,		Yes		✓
													https://www.drugbank.ca/biodb/polypeptides/P18510	
IL21R	HUMAN Interleukin-21 receptor	CD360	Q9HBE5	ENSG0000	Cancer,Interleukin-Interferon	AY064474	Primary immunodeficiency, Systemic lupus erythematosus, Liver cirrhosis, Recurrent sinusitis, Immunologic deficiency syndrome, Pneumonia, Liver Failure, Melanoma, Ovarian serous adenocarcinoma, Rectal adenocarcinoma	https://www.targetvalidation.org/target/ENSG00000103522/associations	IL21 JAK1 JAK1 STAT3 JAK3	Membrane, Single-pass type I membrane protein,	Transmembrane	No		✓
IL24	HUMAN Interleukin-24	IL-24, MAD7, ST16	Q13007	ENSG0000	Cancer,Interleukin-Interferon, Cytokine/Chemokine	AY237723	Li-Fraumeni syndrome, Lung carcinoma, Pancreatic neoplasm, Major depressive disorder, Mammary neoplasm, Prostatic neoplasm, Spontaneous abortion, Unipolar depression, Periodontal disease, Melanoma	https://www.targetvalidation.org/target/ENSG00000162892/associations	IFNB1 IL20RA IL22RA1 IL20RB	Secreted		No		✓
IL37	HUMAN Interleukin-37	FIL1, FIL1(ZETA), FIL1Z, IL-1F7, IL-1H4, IL-1RP1, IL1F7	Q9NZH6	ENSG0000	Interleukin-Interferon, Cytokine/Chemokine	AF251119	Ankylosing spondylitis, Rheumatoid arthritis, Autoimmune diseases, Systemic lupus erythematosus, Inflammatory bowel disease, Ulcerative colitis, Crohn's disease, Psoriasis, Spondylarthritis, Inflammatory disorder	https://www.targetvalidation.org/target/ENSG00000125571/associations	SMAD3 CASP1	Cytoplasm, cytosol		No		✓
ILF2	HUMAN Interleukin enhancer-binding factor 2	NF45	Q12905	ENSG0000	Kinase,Transcription factor,Interleukin-Interferon	AL713889	Neoplasm of mature B-cells, Multiple myeloma, Hepatocellular carcinoma, HIV Infection, Cervix carcinoma, Pancreatic ductal adenocarcinoma, Multiple malignancy, Papilloma, Dengue Fever, Melanoma	https://www.targetvalidation.org/target/ENSG00000143621/associations	MAP3K14 PRKDC ILF3	Nucleus, nucleolus, Cytoplasm, Nucleus,		No		✓

ILK	HUMAN Integrin-linked protein kinase	ILK	Q13418	ENSG0000(	Kinase,Cancer	AJ404847	Exstrophy-epispadias complex, Transient ischemic attack, Epilepsy, Colonic polyp, Malignant mesothelioma, Cocaine-related disorder, Thoracic disease, Ovarian neoplasm, Melanoma, Renal cell carcinoma	https://www.targetvalidation.org/target/ENSG00000166333/associations	PARVB ILKAP PDK1 PAK1 TMSB4X GSK3B PXN AKT1 SHC1 PRKACA PRKCA ITGB1 ITGB3 PDPK1 CAV1 PRKACA PXN GSK3A PPP1R14A RICTOR AKT1 GSK3B ITGB2 ITGB1 PPP1R12A CFL1 PDPK1 CASP8 NCK2 ARHGEF7	Cell junction, focal adhesion	Yes	https://www.bindingdb.org/uniprot/Q13418	✓
IMP3	HUMAN U3 small nucleolar ribonucleoprotein protein IMP3	BRMS2, C15orf12, FLJ10968, MRPS4	Q9NV31	ENSG0000(	Others	AK057531	Breast carcinoma, Glioblastoma multiforme, Lung carcinoma, Psoriasis, Acute myeloid leukemia, Osteosarcoma of bone, Triple negative breast neoplasm, Secondary malignant neoplasm of lymph node, Invasive cancer, Tumor initiation	https://www.targetvalidation.org/target/ENSG00000177971/associations	IMP4 MPHOSPH10	Nucleus, nucleolus	No		✓
IMPA1	HUMAN Inositol monophosphatase 1	IMPA	P29218	ENSG0000(	Others	AK297078	Bipolar disorder, Psychosis, Osteosarcoma, HIV infection, Ovarian carcinoma, Pseudomonas infection, Colon carcinoma, Seizures, Stroke, Thyroid carcinoma	https://www.targetvalidation.org/target/ENSG00000133731/associations	CALB1 S100B	Cytoplasm	Yes	https://www.drugbank.ca/biodb/polypeptides/P29218	✓
IMPDH1	HUMAN Inosine-5'-monophosphate dehydrogenase 1	LCA11, RP10,	P20839	ENSG0000(	Signalling	AK122994	Retinal dystrophy, Retinitis pigmentosa, Leber congenital amaurosis, Myasthenia gravis, Systemic lupus erythematosus, Uveitis, Ischemia reperfusion injury, IGA Glomerulonephritis, Cauda equina syndrome, Chronic kidney disease	https://www.targetvalidation.org/target/ENSG00000106348/associations		Cytoplasm	Yes	https://www.bindingdb.org/uniprot/P20839	✓
INA	HUMAN Alpha-internexin	NEF5, NF-66, AINX	Q16352	ENSG0000(	Others	BC006359	Li-Fraumeni syndrome, Glioma, Neurodegenerative disorder, Multiple myeloma, Neuroblastoma, Schizophrenia, Oligodendroglioma, Frontotemporal dementia, Amyotrophic lateral sclerosis, Prostate carcinoma	https://www.targetvalidation.org/target/ENSG00000148798/associations	ESR1		No		✓
ING4	HUMAN Inhibitor of growth protein 4	my036, p29ING4	Q9UNL4	ENSG0000(	Transcription factor	AB197697	Leukocyte adhesion deficiency, Glioblastoma, Mammary neoplasm, Adenocarcinoma, Tumor angiogenesis, Breast carcinoma, Neoplasm metastasis, Melanoma, Lung carcinoma, Idiopathic pulmonary fibrosis	https://www.targetvalidation.org/target/ENSG00000111653/associations	EGLN1 HNRNPD PPFIA1 RELA HIST1H3A; HIST1H3B; HIST1H3C; HIST1H3D; HIST1H3E; HIST1H3F; HIST1H3G ; HIST1H3H; HIST1H3I; HIST1H3J GADD45A TP53 KAT7	Nucleus	No		✓
INHBA	HUMAN Inhibin beta A chain	INHBA	P08476	ENSG0000(	Kinase,Signalling,Cancer, Cytokine/Chemokine	X57579	Squamous cell carcinoma, Esophageal squamous cell carcinoma, Pre-eclampsia, Ovarian adenocarcinoma, Fibroid tumor, Uterine neoplasm, Polycystic ovary syndrome, Anoxia, Clear cell carcinoma, Myocardial infarction	https://www.targetvalidation.org/target/ENSG00000122641/associations	ACVR2B ACVR1 ACVR1B ACVR2A FST FSTL3 ACVR1B ACVR1 ACVR2B ACVR2A IGFBP7 CHRDL2	Secreted	No		✓
INPP1	HUMAN Inositol polyphosphate 1-phosphatase	INPP	P49441	ENSG0000(	Others	NA	Breast carcinoma, Achondrogenesis, Brachyolmia, Bipolar disorder, Autistic disorder, Type II diabetes mellitus, Colorectal carcinoma, Post-traumatic stress disorder, Colorectal cancer, Lepidic predominant adenocarcinoma	https://www.targetvalidation.org/target/ENSG00000151689/associations			No		✓

INPP5B	HUMAN Type II inositol 1,4,5-trisphosphate 5-phosphatase	I5P2	P32019	ENSG0000(	Kinase	AL603790, AL929472	Male infertility with teratozoospermia due to single gene mutation, Male infertility with azoospermia or oligozoospermia due to single gene mutation, Non-syndromic male infertility due to sperm motility disorder, Male infertility due to large-headed multiflagellar polyploid spermatozoa, Deafness-infertility syndrome, Oculocerebrorenal syndrome, Atrophy of testis, Primary ciliary dyskinesia, Partial chromosome Y deletion, Ciliopathy	https://www.targetvalidation.org/target/ENSG00000204084/associations		Cytoplasm, cytosol	No		✓
IP6K1	HUMAN Inositol hexakisphosphate kinase 1	IHPK1, KIAA0263	Q92551	ENSG0000(	Kinase	AC099668	Self-reported educational attainment, Male infertility with azoospermia or oligozoospermia due to single gene mutation, Male infertility with teratozoospermia due to single gene mutation, Non-syndromic male infertility due to sperm motility disorder, Type II diabetes mellitus, Crohn's disease, Intraepithelial neoplasia, Invasive carcinoma, Breast carcinoma, Kallmann syndrome	https://www.targetvalidation.org/target/ENSG00000176095/associations		Cytoplasm	No		✓
IP6K2	HUMAN Inositol hexakisphosphate kinase 2	IHPK2	Q9UHH9	ENSG0000(	Kinase	AK304714	Self-reported educational attainment, Huntington disease, Ovarian carcinoma, Epithelial ovarian cancer, Colorectal carcinoma, Multiple malignancy, Invasive carcinoma, Colorectal cancer, Melanoma, Pancreatic ductal adenocarcinoma	https://www.targetvalidation.org/target/ENSG00000068745/associations	CSNK2A1 GZMB HSP90AA1 TP53 GZMB TRAF2	Nucleus	No		✓
IPMK	HUMAN Inositol polyphosphate multikinase	IPMK	Q8NFU5	ENSG0000(	Kinase	NA	Inflammatory bowel disease, Crohn's disease, Neural tube closure defect, Isolated spina bifida, Carcinoid tumor, Carcinoid tumor no ICD-O subtype, Glioblastoma multiforme, Neural tube closure defect, Breast carcinoma, Coronary artery calcification	https://www.targetvalidation.org/target/ENSG00000151151/associations		Nucleus	No		✓
IRAK1	HUMAN Interleukin-1 receptor-associated kinase 1	IRAK	P51617	ENSG0000(	Kinase,Transcription factor,Interleukin-Interferon	DQ054788	Systemic lupus erythematosus, Rheumatoid arthritis, Coronary artery disease, Brain hypoxia-ischemia, Cognition disorder, Systemic lupus erythematosus, Sepsis, Prostate neoplasm, Atherosclerosis, Gastric neoplasm	https://www.targetvalidation.org/target/ENSG00000184216/associations	IKBKG IRAK3 TRAF6 TAB2 IRAK4 IL1RAP PELI2 UNC93B1 TOLLIP TRAF4 MYD88 PELI1 IRF7 PELI3 UNC5CL TICAM2 TMEM173 SARM1 SIGIRR IRAK1BP1 CDC37 MAPK14 NTRK3 TAB1 TRIP6 IRF4 PDK1 PIN1 SQSTM1 FADD	Cytoplasm	Yes	https://www.bindingdb.org/uniprot/P51617	✓
IRAK4	HUMAN Interleukin-1 receptor-associated kinase 4	NY-REN-64	Q9NWZ3	ENSG0000(	Kinase,Transcription factor,Cancer,Cancer-Antigen,Interleukin-Interferon	AY340965	Primary immunodeficiency, Immunodeficiency due to interleukin-1 receptor-associated kinase-4 deficiency, Lymphoma, IRAK4 Deficiency, Immunologic deficiency syndrome, Prostatic neoplasm, Recurrent streptococcus pneumoniae infection, Sepsis, Recurrent staphylococcal infection, Autosomal recessive predisposition	https://www.targetvalidation.org/target/ENSG00000198001/associations	IRAK3 TRAF6 MYD88 PELI1 UNC5CL TICAM2 IL1RL1 TIRAP IRAK1 IL1R1 IRAK2 TNFRSF13B IRAK1 TLR5 TIRAP TLR4 MYD88 TICAM2 TRAF6 IL1R1 IL1RL1 PELI1 TBPL1 IRAK2 PELI2 NCF1	Cytoplasm	Yes	https://www.bindingdb.org/uniprot/Q9NWZ3	✓
IRF4	HUMAN Interferon regulatory factor 4	LSIRF, MUM1	Q15306	ENSG0000(	Transcription factor,Cancer,Interleukin-Interferon	AL365272, AL589962	Lymphoid neoplasm, Neoplasm of mature B-cells, Chronic lymphocytic leukemia, Multiple myeloma, Adult T-cell Leukemia, Middle cerebral artery infarction, Melanoma, Hodgkin's disease, Chromosome aberration, Skin neoplasm	https://www.targetvalidation.org/target/ENSG00000137265/associations	FKBP4 SPI1 ROCK2 IRAK1 STAT6 MYD88 RIPK2 BCL6 IRF5 TRAF6 NFATC2 DEF6	Nucleus,	No		✓
IRF5	HUMAN Interferon regulatory factor 5	IRF5	Q13568	ENSG0000(	Transcription factor,Signalling,Interleukin-Interferon	AY693666	Autoimmune disease, Rheumatic disease, Systemic lupus erythematosus, Inflammatory bowel disease, Arthritis, Primary biliary cirrhosis, Systemic scleroderma, Pulmonary fibrosis, Ulcerative colitis	https://www.targetvalidation.org/target/ENSG00000128604/associations	TRAF6 TBK1 MYD88 MAVS IRF4 IRF3 IKBKE RELA TRIM21 ALPI TP53 RIPK2 IKBKB IRF6 IRF3 IRF7 MYD88 TRIM28 MAVS SETDB1 GRB2 IL10 IRF1 XPO1 GTPBP2 KPNA1 RGS3 HIVEP1 IL23A GPS1 CEP152 SGTA GTPBP1 KPNA6	Cytoplasm, Nucleus,	No		✓

ISG20	HUMAN Interferon-stimulated gene 20 kDa protein	CD25, HEM45	Q96AZ6	ENSG0000(	Signalling,Interleukin-Interferon	CR456942	Psoriasis, Adenocarcinoma, Autoimmune disease, Multiple sclerosis, Type I diabetes mellitus, Asthma, Rheumatoid arthritis, Systemic lupus erythematosus, Acute leukemia myelocytic, Systemic mastocytosis	https://www.targetvalidation.org/target/ENSG00000172183/associations	RELA NFKB1 IRF1	Nucleus, nucleolus, Cytoplasm, Cajal body,		Yes		✓
													https://www.drugbank.ca/biodb/polypeptides/Q96AZ6	
IST1	HUMAN IST1 homolog	KIAA0174	P53990	ENSG0000(	Others	AK293022	Astrocytoma, Glioblastoma multiforme, Atopic eczema, Pancreatic carcinoma, Congenital abnormality, Lung carcinoma, Breast carcinoma, Glioblastoma multiforme, Hereditary spastic paralegia, Colorectal adenocarcinoma	https://www.targetvalidation.org/target/ENSG00000182149/associations	VPS4A CHMP1B CAPN7	Cytoplasmic vesicle		No		✓
ITGB6	HUMAN Integrin beta-6	ITB6	P18564	ENSG0000(	Cancer	AC080166	Amelogenesis imperfecta, Prostate carcinoma, Lung carcinoma, Pulmonary emphysema, Defective enamel matrix, Hearing impairment, Primary hypogonadism, Mental deficiency, Delayed bone age, Melanoma	https://www.targetvalidation.org/target/ENSG00000115221/associations	FHL2 MAPK1 ITGAV VTN FN1 MAPK1 TGFB1	Membrane, Single-pass type I membrane protein, Cell junction, focal adhesion	Transmembrane	Yes		✓
													https://www.bindingdb.org/uniprot/P18564	
ITGBL1	HUMAN Integrin beta-like protein 1	OSCP, TIED	O95965	ENSG0000(	Others	AK298571	Breast carcinoma, Colorectal cancer, Non-small cell lung carcinoma, Pneumocystis jiroveci pneumonia, Colorectal cancer, Pancreatic ductal adenocarcinoma, Ovarian carcinoma, Gastric carcinoma, Psoriasis, Pituitary cancer	https://www.targetvalidation.org/target/ENSG00000198542/associations		Secreted		No		✓
ITK	HUMAN Tyrosine-protein kinase ITK/TSK	EMT, LYK, PSCTK2	Q08881	ENSG0000(	Kinase,Cancer	AK312846	Primary immunodeficiency, Lymphoproliferative syndrome, Autosomal recessive lymphoproliferative disease, Lymphoma, Splenomegaly, Aphthous stomatitis, Hepatomegaly, Pancytopenia, IgG Deficiency, Low hemoglobin count	https://www.targetvalidation.org/target/ENSG00000113263/associations	IKBKG BLNK LCP2 BTK GRB2 TEC ERBB3 CD28 FYN LCK ERBB2 LAT PLCG2 LCK PLCG1 SMAD4 KPNA2 KIF2C LAT SOCS1 HNRNPK GRB2 CBL BTK TGFBR1 CD28 KHDRBS1 TEC PPIA LCP2 BMX SIGLEC10 CSF2RB WAS G3BP1 SDC4	Cytoplasm		Yes		✓
													https://www.bindingdb.org/uniprot/Q08881	
ITPK1	HUMAN Inositol-tetrakisphosphate 1-kinase	ITPK1	Q13572	ENSG0000(	Kinase	BC007428	Thyroid stimulating hormone measurement, Migraine disorder, Neural tube defect, Neural tube closure defect, Spina bifida, Subarachnoid hemorrhage, Intracranial hemorrhage, Cerebral hemorrhage, Neoplasm invasiveness, Iniencephaly	https://www.targetvalidation.org/target/ENSG00000100605/associations				Yes		✓
													https://www.bindingdb.org/uniprot/Q13572	
ITPKB	HUMAN Inositol-trisphosphate 3-kinase B	IP3KB	P27987	ENSG0000(	Kinase,Cancer	AL365444	Li-Fraumeni syndrome, Severe combined immunodeficiency, Agammaglobulinemia, Isolated agammaglobulinemia, Acute myelocytic leukemia, Transcobalamin deficiency, Wiskott-Aldrich syndrome, Thymic aplasia, Medulloblastoma	https://www.targetvalidation.org/target/ENSG00000143772/associations	PRKACA PRKCA			No		✓
JADE1	HUMAN Protein Jade-1	KIAA1807, PHF17	Q6IE81	ENSG0000(	Transcription factor	AK074986	Glioblastoma multiforme, Lung carcinoma, Lung adenocarcinoma, Psoriasis, Kidney Neoplasm, Von Hippel-Lindau Syndrome, Renal cell carcinoma, Cystic kidney disease, Acute kidney failure, Asthma	https://www.targetvalidation.org/target/ENSG00000077684/associations	CSNK1A1 VHL CTNNB1 MAPK1 PRKACA PRKCB KAT7	Cytoplasm		No		✓

JAK3	HUMAN Tyrosine-protein kinase JAK3	JAK-3, JAK3_HUMAN, L-JAK	P52333	ENSG0000	Kinase,Cancer	U70065	Immune system disease, Severe combined immunodeficiency, T-B+ severe combined immunodeficiency, T-B+ severe combined immunodeficiency due to JAK3 deficiency, Juvenile myelomonocytic leukemia, Pneumonia, Intestinal obstruction, Diarrhea, Panhypogammaglobulinemia, Recurrent upper respiratory tract infection	https://www.targetvalidation.org/target/ENSG00000105639/associations	IKBKG ALK IL21R INPP5D STAM PTK2B KHDRBS1 PTPN11 IL9R CXCR4 STAT5A IRS1 IL2RG PTPN6 PIK3R1 IL4R JAK1 ERBB3 PTPN2 IL7R IL2RB VIL1 PTPRC FES LCK STAM2 JAK2 SOCS1 SOCS3 LCK STAT5A JAK2 JAK1 STAT6	Endomembrane system	Yes	https://www.bindingdb.org/uniprot/P52333	✓
JUNB	HUMAN Transcription factor jun-B	JUNB	P17275	ENSG0000	Kinase,Transcription factor	AY751746	Breast carcinoma, Lung carcinoma, Pituitary cancer, Myocardial ischemia, Breast carcinoma, Lymphoma, Status epilepticus, Middle cerebral artery infarction, Systemic lupus erythematosus, Pituitary cancer	https://www.targetvalidation.org/target/ENSG00000171223/associations	MAPK14 FOSB GSK3B MAPK9 MAPK8 STAT3 FOSL2 ABL1 EGFR SMAD4 NFE2L2 SMAD3 BCL2 FOSL1 NRF1 FOS BCL2L1 SMURF1	Nucleus,	No		✓
JUP	HUMAN Junction plakoglobin	CTNNG, DP3, PLAK	P14923	ENSG0000	Cancer	AY243535	Arrhythmogenic right ventricular dysplasia, Diffuse palmoplantar keratoderma, Naxos disease, Prostatic neoplasm, Palmoplantar keratosis, Fragile skin, Nikolsky sign, No-reflow phenomenon, Dystrophia unguium, Cardiomyopathy	https://www.targetvalidation.org/target/ENSG00000173801/associations	PKP2 DSC3 DSG2 DSC1 DSC2 DSG1 ARHGDIA CTNNB1 CTNNA1 CDH5 DSG3 APC CDH3 CDH2 FER DSP TCF4 SRC CDH1 FYN ERBB2 CTNND1 CTNNB1 EGFR CDH1 APC TCF7L2 ERBB2 BTRC CTNND1 CDH2 TCF4 ARHGDIA CTNNA1	Cell junction, adherens junction	No		✓
KAT2A	HUMAN Histone acetyltransferase KAT2A	GCN5, GCN5L2, PCAF-b	Q92830	ENSG0000	Transcription factor	BC039907	Inborn errors of metabolism, Spinocerebellar ataxia type 7, Breast neoplasm, Breast carcinoma, Gastric carcinoma, Precursor cell lymphoblastic leukemia lymphoma, Femoral-facial syndrome, Osteogenesis imperfecta type 3, Hepatocellular carcinoma, Waardenburg syndrome	https://www.targetvalidation.org/target/ENSG00000108773/associations	TRRAP CREBBP ZZZ3 EP300 H2AFX WDHD1 ERN1 CTNNB1 IRF7 SMAD3 NOTCH1 SMAD2 SOCS1 MYC HIST1H3A; HIST1H3B; HIST1H3C; HIST1H3D; HIST1H3E; HIST1H3F; HIST1H3G ; HIST1H3H; HIST1H3I; HIST1H3J SMAD7 SMURF1 ADA2 TAF9	Nucleus	Yes	https://www.bindingdb.org/uniprot/Q92830	✓
KAT7	HUMAN Histone acetyltransferase KAT7	HBO1, HBOA, MYST2	O95251	ENSG0000	Transcription factor	AK023890	Breast carcinoma, Leukemia, Lung carcinoma, Aortic valve insufficiency, Acute kidney failure, Androgen-Insensitivity syndrome, Central serous chorioretinopathy, T-cell leukemia, Diabetes mellitus, Psoriasis	https://www.targetvalidation.org/target/ENSG00000136504/associations	PLK1 CDK2 AR CDK1 AR HIST1H4A; HIST1H4B; HIST1H4C; HIST1H4D; HIST1H4E; HIST1H4F; HIST1H4H; HIST1H4I; HIST1H4J; HIST1H4K; HIST1H4L; HIST2H4A; HIST2H4B; HIST4H4 MCM2 HIST1H3A; HIST1H3B; HIST1H3C; HIST1H3D; HIST1H3E; HIST1H3F; HIST1H3G ; HIST1H3H; HIST1H3I; HIST1H3J	Nucleus, nucleoplasm	No		✓
KATNB1	HUMAN Katanin p80 WD40 repeat-containing subunit B1	KTNB1	Q9BVA0	ENSG0000	Cancer,Cancer-Antigen	AC092118	Lissencephaly, Jeune syndrome, Atelosteogenesis type I, Otopalatodigital syndrome, Infantile onset, Sloping forehead, Agyria, Cortical gyral simplification, Mental impairment, Greenberg dysplasia	https://www.targetvalidation.org/target/ENSG00000140854/associations	TGFBR1 KATNA1	Cytoplasm, Cytoplasm, cytoskeleton, microtubule organizing center, centrosome, spindle pole,	No		✓
KCMF1	HUMAN E3 ubiquitin-protein ligase KCMF1	DEBT91, DKFZP434L1021, PCMF, ZZZ1	Q9P0J7	ENSG0000	Cancer,Cancer-Antigen	AK314761	Lung carcinoma, Small cell lung carcinoma, Breast carcinoma, Sarcoma, Colon Carcinoma, Ewings sarcoma, Pancreatic carcinoma, Gastric carcinoma, Melanoma, Cystic fibrosis	https://www.targetvalidation.org/target/ENSG00000176407/associations			No		✓
KCNIP3	HUMAN Calsenilin	CSEN, DREAM, KCHIP3	Q9Y2W7	ENSG0000	Transcription factor	AF199599	Alzheimer's disease, Glioblastoma multiforme, Breast carcinoma, Stroke, Degenerative polyarthritis, Epilepsy, Breast carcinoma, Amyloidosis, Central neuroblastoma, Neuroblastoma	https://www.targetvalidation.org/target/ENSG00000115041/associations	KCND1 CLN3 CREM UBE2I CTBP2 PSEN1 CSNK1A1 CASP3 GRK2 TNFAIP3 CREB1 KCND2 KCND1	Cytoplasm	No		✓
KIF9	HUMAN Kinesin-like protein KIF9	MGC104186	Q9HAQ2	ENSG0000	Cancer,Cancer-Antigen	AK023894	Azoospermia, Teratozoospermia, Breast carcinoma, Central nervous system cancer, Medulloblastoma, Coronary heart disease	https://www.targetvalidation.org/target/ENSG00000088727/associations		Cytoplasm, cytoskeleton	No		✓

KIT	HUMAN Mast/stem cell growth factor receptor Kit	C-Kit, CD117, PBT, SCFR	P10721	ENSG0000(	Cancer,Cancer-Antigen	U63834	Piebaldism, Gastrointestinal stromal tumor, Leukemia, Acute myelocytic leukemia, Mastocytosis, Sarcoma, Small cell lung carcinoma, Gastrointestinal stromal tumor	https://www.targetvalidation.org/target/ENSG00000157404/associations	STAP1 CISH DOK1 SH3KBP1 INPP5D PTPRU SOCS4 SPRED1 GRB7 GRAP GRB10 CBLB BTK PTPN11 CLTC GRB2 BLK CRKL CRK TEC MATK STAT5A STAT1 CSF2RB PTPN6 PIK3R1 CBL KITLG RASA1 EPOR PRKCA SRC FGR PDGFRB HCK LYN	Cell membrane, Single-pass type I membrane protein, Cytoplasm,	Transmembrane	Yes	https://www.bindingdb.org/uniprot/P10721	✓
KIT_ext	HUMAN Mast/stem cell growth factor receptor Kit	C-Kit, CD117, PBT, SCFR	P10721	ENSG0000(	Cancer,Cancer-Antigen	U63834	Piebaldism, Gastrointestinal stromal tumor, Leukemia, Acute myelocytic leukemia, Mastocytosis, Sarcoma, Small cell lung carcinoma, Gastrointestinal stromal tumor	https://www.targetvalidation.org/target/ENSG00000157404/associations	STAP1 CISH DOK1 SH3KBP1 INPP5D PTPRU SOCS4 SPRED1 GRB7 GRAP GRB10 CBLB BTK PTPN11 CLTC GRB2 BLK CRKL CRK TEC MATK STAT5A STAT1 CSF2RB PTPN6 PIK3R1 CBL KITLG RASA1 EPOR PRKCA SRC FGR PDGFRB HCK LYN	Cell membrane, Single-pass type I membrane protein, Cytoplasm,	Transmembrane	Yes	https://www.bindingdb.org/uniprot/P10721	✓
KIT_int	HUMAN Mast/stem cell growth factor receptor Kit	C-Kit, CD117, PBT, SCFR	P10721	ENSG0000(	Cancer,Cancer-Antigen	U63834	Piebaldism, Gastrointestinal stromal tumor, Leukemia, Acute myelocytic leukemia, Mastocytosis, Sarcoma, Small cell lung carcinoma, Gastrointestinal stromal tumor	https://www.targetvalidation.org/target/ENSG00000157404/associations	STAP1 CISH DOK1 SH3KBP1 INPP5D PTPRU SOCS4 SPRED1 GRB7 GRAP GRB10 CBLB BTK PTPN11 CLTC GRB2 BLK CRKL CRK TEC MATK STAT5A STAT1 CSF2RB PTPN6 PIK3R1 CBL KITLG RASA1 EPOR PRKCA SRC FGR PDGFRB HCK LYN	Cell membrane, Single-pass type I membrane protein, Cytoplasm,	Transmembrane	Yes	https://www.bindingdb.org/uniprot/P10721	✓
KLF10	HUMAN Krueppel-like factor 10	TIEG, TIEG1	Q13118	ENSG0000(	Transcription factor	AF050110	Lung carcinoma, Mammary neoplasm, Leukemia, Type II diabetes mellitus, Chronic alcoholic intoxification, Ovarian neoplasm, Pancreatic carcinoma, Neoplasm metastasis, Breast carcinoma, Dent disease	https://www.targetvalidation.org/target/ENSG00000155090/associations	SP1 RAF1 SIN3A KDM5B	Nucleus		Yes	https://www.bindingdb.org/uniprot/Q13118	✓
KLF12	HUMAN Krueppel-like factor 12	AP-2rep, AP2REP, HSPC122	Q9Y4X4	ENSG0000(	Transcription factor	AF113122	Schizophrenia, Rheumatoid arthritis, Exanthema, Type II diabetes mellitus, Prostate carcinoma, Gastric carcinoma, Ovarian carcinoma, Sepsis, Osteosarcoma, Meningococcal infection	https://www.targetvalidation.org/target/ENSG00000118922/associations	EHMT2 CTBP1	Nucleus,		No		✓
KLF4	HUMAN Krueppel-like factor 4	EZF, GKLF	O43474	ENSG0000(	Transcription factor,Cancer	HF546202	Adenocarcinoma, Congenital abnormality, Myocardial ischemia, Juvenile arthritis, Thrombosis, Colonic Neoplasm, Type II diabetes mellitus, Secretory meningioma, Neoplasm of mature B-cells, Cutaneous melanoma	https://www.targetvalidation.org/target/ENSG00000136826/associations	CTBP1 FOXO1 STAT3 TP53 VHL EP300 HDAC2 SMAD2 KLF6 SP1 SRF KAT5 CREBBP	Nucleus,		No		✓
KLF6	HUMAN Krueppel-like factor 6	BCD1, COPEB, CPBP, ST12, Zf9	Q99612	ENSG0000(	Transcription factor,Cancer	AF284036	Prostatic neoplasm, Gastric carcinoma, Somatic mutation, Liver carcinoma, Colorectal neoplasm, Keratoconus, Lung carcinoma, Melanoma, Adenocarcinoma, Colonic neoplasm	https://www.targetvalidation.org/target/ENSG00000067082/associations	LCOR SP1 TP53 KLF4 HDAC3 RELA PTTG1 DAPK2	Nucleus,		No		✓
KLF7	HUMAN Krueppel-like factor 7	UKLF	O75840	ENSG0000(	Transcription factor	BX537890	Prostate carcinoma, Familial idiopathic steroid-resistant nephrotic syndrome, Breast carcinoma, Grebe type acromesomelic dysplasia, Type II diabetes mellitus, Obesity, Acheiropodia, Glioblastoma multiforme, MPI-CDG, Mucopolysaccharidosis type 3	https://www.targetvalidation.org/target/ENSG00000118263/associations	FBXO38	Nucleus		No		✓
KLHL12	HUMAN Kelch-like protein 12	C3IP1, hDKIR	Q53G59	ENSG0000(	Cancer,Cancer-Antigen	AK223072	Lung carcinoma, Pancreatic ductal adenocarcinoma, Juvenile dermatomyositis, Acute quadriplegic myopathy, Breast carcinoma, Hypertensive disease, Biliary liver cirrhosis, Hepatocellular carcinoma, Autoimmue hepatitis type 1, Prostate carcinoma	https://www.targetvalidation.org/target/ENSG00000117153/associations	DVL3 CUL3 DRD4 ARAF KLHL2 RIT1	Cytoplasmic vesicle, COPII-coated vesicle		No		✓

KLK3	HUMAN Prostate-specific antigen	APS, PSA	P07288	ENSG0000(	Cancer	X13941	Prostate carcinoma, Prostatic hyperplasia, Mammary neoplasm, Breast neoplasm, Vascular disease, Tuberculosis, Systemic lupus erythematosus, Rheumatic disease, Psychosis, Autoimmune disease	https://www.targetvalidation.org/target/ENSG00000142515/associations	AR SERPINA5 SLPI FN1 A2M SERPINA3 KDM1A IGFBP3 AR PTHLH PLG SEMG1	Secreted	Yes	https://www.bindingdb.org/uniprot/P07288	✓
KRAS	HUMAN GTPase KRas	KRAS2, RASK2, RASK	P01116	ENSG0000(	Kinase,Cancer	AF493917	Lymphedema, Juvenile myelomonocytic leukemia, Nevus sebaceous, Pancreatic neoplasm, Noonan syndrome, Gastric carcinoma, Adenocarcinoma, Cardio-facio-cutaneous syndrome, Colorectal neoplasm, Lung neoplasm	https://www.targetvalidation.org/target/ENSG00000133703/associations	SHOC2 RAPGEF5 RASSF5 RASGEF1A RASGEF1C RASGRP2 RIN1 RASGEF1B SOS2 SOS1 PRKCQ PPP2CB RAP1GDS1 FNTB FNTA PIK3CA LGALS3 PRKCA SRC BCL2 APP RAF1 PDE6D CALM1 PIK3R2 RAF1 TCF3 MAP3K1 APP SOS1	Cell membrane	Yes	https://www.bindingdb.org/uniprot/P01116	✓
KRT14	HUMAN Keratin, type I cytoskeletal 14	K1C14	P02533	ENSG0000(	Cancer,Cancer-Antigen	BC002690	Rare genetic skin disease, Inherited epidermolysis bullosa, Epidermolysis bullosa simplex, Dermatopathia pigmentosa reticularis, Naegeli syndrome, Weber-cockayne syndrome, Ectodermal dysplasia, Prostate carcinoma, Darier disease, Acral peeling skin syndrome	https://www.targetvalidation.org/target/ENSG00000186847/associations	KRT5 TRADD TNFRSF1A TCHP PKP2 KRT5	Cytoplasm, Nucleus,	No		✓
KRT15	HUMAN Keratin, type I cytoskeletal 15	CK15, K15, K1CO, K1C15, KRTB	P19012	ENSG0000(	Cancer,Cancer-Antigen	AK090604	Prostate carcinoma, Breast carcinoma, Neoplastic cell transformation, Adenoid cystic carcinoma, Salivary gland neoplasm, Bladder neoplasm, Aplasia cutis congenita, Acute GVH disease, Hyperplasia, Psoriasis	https://www.targetvalidation.org/target/ENSG00000171346/asso			No		✓
KRT19	HUMAN Keratin, type I cytoskeletal 19	CK19, K19, K1CS	P08727	ENSG0000(	Cancer,Cancer-Antigen	AK313261	Epithelial neoplasm, Asthma, Autoimmune disease, Cholangitis, Breast carcinoma, Copper-overload cirrhosis, Li-Fraumeni syndrome, Hepatocellular carcinoma, Deafness-infertility syndrome, HPV infection	https://www.targetvalidation.org/target/ENSG00000171345/associations	CSK SRC RAF1		No		✓
KRT4	HUMAN Keratin, type II cytoskeletal 4	CK4, CYK4, K4	P19013	ENSG0000(	Cancer	BC042174	White sponge nevus, Epithelial neoplasm, Autoimmune disease, Abnormality of metabolism/homeostasis, Oral leukoplakia, Leukokeratosis, Waisman syndrome, Squamous cell carcinoma, Intraepithelial neoplasia, Nasopharyngeal neoplasm, KID syndrome	https://www.targetvalidation.org/target/ENSG00000170477/asso			No		✓
KRT8	HUMAN Keratin, type II cytoskeletal 8	CK8, CYK8, K2C8, K8	P05787	ENSG0000(	Cancer,Cancer-Antigen	X74981	Fibrosis, Liver cirrhosis, Adenocarcinoma, Acute liver failure, Liver neoplasm, Precancerous condition, Gastric neoplasm, Renal cell carcinoma, Colorectal cancer, Ovarian carcinoma	https://www.targetvalidation.org/target/ENSG00000170421/asso	IKBKG PKP2 AURKB CAMK4 MAPK14 MAPK11 PKP1 PRKCD MAP2K1 PPP2CA GRB2 PPP2CB MAPK12 MAPK8 MAP2K2 MAPK1 MAPK3 FGFR3 DSP CDK1 KRT18 TP53 ANXA1 RAF1 ESR1 PLAT EGFR PTP4A3 MAPK13 KRT18	Cytoplasm	Yes	https://www.drugbank.ca/biodb/polypeptides/P05787	✓
KXD1	HUMAN KxDL motif-containing protein 1	C19orf50, KXDL, KXDL1	Q9BQD3	ENSG0000(	Others	NA	Retinitis pigmentosa, Reticular dystrophy of the retinal pigment epithelium, Breast carcinoma, Ovarian carcinoma, Diabetes mellitus, Prostate adenocarcinoma, Hepatocellular carcinoma, Glioblastoma multiforme, Ductal adenocarcinoma	https://www.targetvalidation.org/target/ENSG00000105700/associations		Lysosome membrane	No		✓

L3MBTL2	HUMAN Lethal(3)malignant brain tumor-like protein 2	L3MBTL2	Q969R5	ENSG0000(	Transcription factor	AK074091	Schizophrenia, Gastric carcinoma, Breast carcinoma, Medulloblastoma, Brain neoplasm, Acute myelocytic leukemia, Polycythemia vera, Neurotic disorder, Gastric carcinoma, Rheumatoid arthritis	https://www.targetvalidation.org/target/ENSG00000100395/associations	HIST1H4A; HIST1H4B; HIST1H4C; HIST1H4D; HIST1H4E; HIST1H4F; HIST1H4H; HIST1H4I; HIST1H4J; HIST1H4K; HIST1H4L; HIST2H4A; HIST2H4B; HIST4H4	Nucleus	No		✓
LAD1	HUMAN Ladinin-1	LAD1	O00515	ENSG0000(	Others	BC009742	Type II diabetes mellitus, Congenital leukocyte adherence deficiency, Leukocyte adhesion deficiency type 1, Persistent embryonic structure, Hypertensive disease, Linear IgA bullous dermatosis, Junctional epidermolysis bullosa, Metastatic prostate cancer, Autoimmune disease, Cardia troponin T measurement	https://www.targetvalidation.org/target/ENSG00000159166/associations	COL17A1	Secreted, extracellular space, extracellular matrix, basement membrane	No		✓
LAMTOR3	HUMAN Ragulator complex protein LAMTOR3	MAP2K1IP1, MAPBP, MAPKSP1, MP1, PRO2783	Q9UHA4	ENSG0000(	Signalling	AK311755	Ovarian carcinoma, Breast carcinoma, Pancreatic carcinoma, Gastric carcinoma, Lung carcinoma, Medulloblastoma, Thrombocytopenia, Acute myeloid leukemia, Inflammation, Melanoma	https://www.targetvalidation.org/target/ENSG00000109270/associations	KSR2 MAP2K1 MAP2K2 MAPK1 MAPK3 MAPK1 MAP2K1 MAPK3 MAP4K1 ZFYVE28 LAMTOR2	Late endosome membrane	No		✓
LASP1	HUMAN LIM and SH3 domain protein 1	Lasp-1, MLN50	Q14847	ENSG0000(	Cancer	AC110749	Adenocarcinoma, Lung carcinoma, Autistic disorder, Schizophrenia, Breast neoplasm, Colorectal carcinoma, Colorectal cancer, Bladder neoplasm, Liver carcinoma, Papillary renal cell carcinoma	https://www.targetvalidation.org/target/ENSG00000002834/associations	PRKG1 PRKG2 CXCR4 PRKX CDK7 CXCR3 PRKAR2B CXCR2 CXCR1 PRKACA CDK1 PRKCB ABL1 PRKY ACTB ZYX LPP	Cytoplasm, cell cortex	No		✓
LATS1	HUMAN Serine/threonine-protein kinase LATS1	WARTS	O95835	ENSG0000(	Kinase,Cancer,Cancer-Antigen	NA	Hypohidrotic ectodermal dysplasia, Hepatocellular carcinoma, Astrocytoma, Basal cell carcinoma, Sarcoma, Warts, Colorectal carcinoma, Colorectal cancer, Neurilemmoma, EEC syndrome	https://www.targetvalidation.org/target/ENSG00000131023/associations	MOB1A NUAK2 MOB1B PRKD1 STK3 STK4 NF2 CDK1 NUAK1 HTRA2 ITCH WWTR1 STK4 YAP1 LIMK1 CDK1 KIF23 STK3 ZYX LIMD1 FOXL2 PPP2R1A	Cytoplasm, cytoskeleton, microtubule organizing center, centrosome	Yes	https://www.bindingdb.org/uniprot/O95835	✓
LCK	HUMAN Tyrosine-protein kinase Lck	LCK, LSK, YT16, p56-LCK	P06239	ENSG0000(	Kinase,Cancer	X14055,X14053,X14054	Primary immunodeficiency due to a defect in adaptive immunity, Severe combined immunodeficiency, Diarrhea, Panniculitis, Infantile onset, Autosomal recessive predisposition, Recurrent respiratory infection, Autoimmune disease, Immunologic deficiency syndrome, HIV infection	https://www.targetvalidation.org/target/ENSG00000182866/associations	IKBKG PTPN22 GAB2 NLK PAG1 PTPRH MAPK15 PTCRA MAPK6 PTK2B CBLB MAPK7 DLG1 ITK PRKCD PTK2 PRKCQ MAPK10 JAK3 MAPK9 MAPK8 SYK ZAP70 CSK MAPK4 AXL PTPN6 MAPK1 PIK3R1 MAPK3 CBL	Cytoplasm	Yes	https://www.bindingdb.org/uniprot/P06239	✓
LCP1	HUMAN Plastin-2	L-PLASTIN, LC64P, PLS2, PLSL	P13796	ENSG0000(	Cancer	AL137141	Autoimmune disease, Lung carcinoma, Liver cirrhosis, Melanoma, Colonic neoplasm, Colorectal neoplasm, Type II diabetes mellitus, Prostate carcinoma, Ovarian carcinoma, Agammaglobulinemia	https://www.targetvalidation.org/target/ENSG00000136167/associations	PRKCD PRKX BLK PRKACA IL7R PRKY PRKCB CSF2RA CSF2RB GCA AIF1	Cytoplasm, cytoskeleton	No		✓
LDB1	HUMAN LIM domain-binding protein 1	CLIM2, NLI, hLdb1	Q86U70	ENSG0000(	Transcription factor	AB250384	Self-reported educational attainment, Melanoma, Nail-patella syndrome, Anaplasia, Squamous cell carcinoma, T-cell leukemia, Kidney Failure, Acute leukemia, Head and Neck malignant neoplasia, Diffuse large B-cell lymphoma	https://www.targetvalidation.org/target/ENSG00000198728/associations	LMO4 LMO1 LMO2 LMX1B LHX3 TRIM33 RLIM LHX2 LMX1A	Nucleus	No		✓
LDB2	HUMAN LIM domain-binding protein 2	CLIM1, LDB1	O43679	ENSG0000(	Transcription factor	AF064493	Rhegmatogenous retinal detachment, Melanoma, Lung adenocarcinoma, Arteriosclerosis, Atherosclerosis, Coronary arteriosclerosis, Lung adenocarcinoma, Astrocytoma, Glioblastoma multiforme, Psoriasis	https://www.targetvalidation.org/target/ENSG00000169744/associations	LHX9	Nucleus	No		✓

LDHA	HUMAN L-lactate dehydrogenase A chain	LDHA, PIG19, LDH-M	P00338	ENSG0000	Cancer	AK130587	Metabolic myopathy, Glycogen storage disease, Glycogen storage disease due to lactate dehydrogenase deficiency, Glycogen storage disease due to lactate dehydrogenase M-subunit deficiency, Myoglobinuria, Lactate dehydrogenase deficiency type A, Muscle rigidity, Rhabdomyolysis, Renal insufficiency, Impaired exercise tolerance, Kidney failure	https://www.targetvalidation.org/target/ENSG00000134333/associations	FGFR1 LDHB CDK1 ISG15 HSPA8 LDHB	Cytoplasm,		Yes	https://www.bindingdb.org/uniprot/P00338	✓
LDHB	HUMAN L-lactate dehydrogenase B chain	LDHB	P07195	ENSG0000	Cancer,Cancer-Antigen	BC015122	Glycogen storage disease due to lactate dehydrogenase deficiency, Glycogen storage disease due to lactate dehydrogenase H-subunit deficiency, Noninfiltrating intraductal carcinoma, Inborn errors of metabolism, Acute coronary syndrome, Renal cell carcinoma, Neoplasm invasiveness, Adenocarcinoma, LCAT deficiency, Breast carcinoma	https://www.targetvalidation.org/target/ENSG00000111716/associations	TBK1 IL7R LDHA LDHA	Cytoplasm,		Yes	https://www.bindingdb.org/uniprot/P07195	✓
LDHC	HUMAN L-lactate dehydrogenase C chain	CT32	P07864	ENSG0000	Others	AY286300	Azoospermia, Non-syndromic male infertility due to sperm motility disorder, Male infertility with teratozoospermia due to single gene mutation, Male infertility with azoospermia or oligozoospermia due to single gene mutation, Teratozoospermia, Tuberculosis, Non-small cell lung carcinoma, Oligospermia, Deafness-infertility syndrome, Primary ciliary dyskinesia	https://www.targetvalidation.org/target/ENSG00000166796/associations	CREB1 SP1	Cytoplasm,		Yes	https://www.drugbank.ca/biodb/polypeptides/P07864	✓
LEF1	HUMAN Lymphoid enhancer-binding factor 1	TCF10, TCF1ALPHA, TCF7L3	Q9UJU2	ENSG0000	Kinase,Transcription factor	AK294395	Adenocarcinoma, Lung carcinoma, Colonic neoplasm, Chronic lymphocytic leukemia, T-cell lymphoma, Neutropenia, Colon carcinoma, Peripheral T-cell lymphoma, Leukemia, Melanoma	https://www.targetvalidation.org/target/ENSG00000138795/associations	NARF NLK HIPK2 ALX4 PITX2 PIAS4 ALYREF SMAD2 SMAD4 EP300 TLE2 TLE1 SMAD3 CSNK2A1 CSNK1E CSNK1D CSNK1A1 CDX1 NOTCH1 TCF7 CTNNB1 APC PAX3 MYB MITF CTNNB1 MITF EP300 HIPK2 NOTCH1 SUMO2 PIAS4 RUNX2	Nucleus		No		✓
LEMD1	HUMAN LEM domain-containing protein 1	CT50, LEMP-1	Q68G75	ENSG0000	Others	AB096677	Colorectal neoplasm, Azoospermia, Renal cell carcinoma, Pancreatic adenocarcinoma, Colorectal cancer, Teratozoospermia, Pancreatic carcinoma, Melanoma, Lung carcinoma, Squamous cell carcinoma	https://www.targetvalidation.org/target/ENSG00000186007/associations		Membrane	Transmembrane	No		✓
LGALS1	HUMAN Galectin-1	HBL, HPL	P09382	ENSG0000	Others	EU363770	Melanoma, Non-small cell lung carcinoma, Keloid, Nephritis, Liver cirrhosis, Glioblastoma, Squamous cell carcinoma, Hodgkin's disease, Neoplasm metastasis, Hepatocellular carcinoma	https://www.targetvalidation.org/target/ENSG00000100097/associations	IL7R ITGB1 HRAS FAS PTPRB POU2AF1 SPN VPREB1 MUC16	Secreted, extracellular space, extracellular matrix		Yes	https://www.bindingdb.org/uniprot/P09382	✓
LGALS7	HUMAN Galectin-7	GAL7, LGALS7B, PIG1	P47929	ENSG0000	Others	CH471126	Tongue squamous cell carcinoma, Adenocarcinoma, Squamous cell carcinoma, Basal cell carcinoma, Breast Carcinoma, Hypopharyngeal neoplasm, Neoplasm metastasis, Oral neoplasm, Prostate carcinoma, Melanoma	https://www.targetvalidation.org/target/ENSG00000205076/associations	MAP3K3	Cytoplasm		Yes	https://www.bindingdb.org/uniprot/P47929	✓
LGMN	HUMAN Legumain	LGMN1, PRSC1	Q99538	ENSG0000	Cancer	BX161422	Dent disease, Congenital dyserythropoietic anemia, Nephronophthisis, Dent disease type 1, Ischemia, Ovarian neoplasm, Colorectal neoplasm, Enzyme inhibition disorder, Borderline ovarian tumor, Breast carcinoma	https://www.targetvalidation.org/target/ENSG00000100600/associations	HLA-DRB1 ISG15 CD74 CD74	Lysosome		Yes	https://www.bindingdb.org/uniprot/Q99538	✓
LHFP	HUMAN Lipoma HMGIC fusion partner	MGC22429	Q9Y693	ENSG0000	Cancer	AL158194, AL136358	Lung carcinoma, Prostate carcinoma, Colorectal adenocarcinoma, Glioma, Gliosarcoma, Familial breast cancer, Mesenchymal cell neoplasm, Lipoma, Melanoma, Basal cell carcinoma	https://www.targetvalidation.org/target/ENSG00000183722/asso		Membrane	Transmembrane	No		✓

LIMK2	HUMAN LIM domain kinase 2	LIMK2	P53671	ENSG0000(	Kinase,Cancer	CR456513	Schizophrenia, Non-syndromic congenital cataract, Azoospermia, Oligospermia, Male infertility, Central neuroblastoma, Fibrosarcoma metastatic, Colorectal cancer, Neurofibromatosis 2, Kallmann syndrome	https://www.targetvalidation.org/target/ENSG00000182541/associations	HIPK3 CDC42BPA ROCK1 PRKCD LIMK1 ROCK2 AURKA PRKCD CRK LIMK1 CFL1 GSN PARD3 PTK2B SSH1 DSTN	Cytoplasm, Nucleus,	Yes		✓
												https://www.bindingdb.org/uniprot/P53671	
LIMS1	HUMAN LIM and senescent cell antigen-like-containing domain protein 1	PINCH, PINCH1, NY-REN-48	P48059	ENSG0000(	Cancer,Cancer-Antigen	AK304260	Glioblastoma multiforme, Osteosarcoma, Neoplasm metastasis, Glioma, Breast carcinoma, Carcinoma breast stage IV, Clear cell adenocarcinoma, Renal fibrosis, Alzheimer's disease, Lung adenocarcinoma	https://www.targetvalidation.org/target/ENSG00000169756/associations	ILK TGFBR1 NCK2 RSU1	Cell junction, focal adhesion, Cell membrane, Peripheral membrane protein, Cytoplasmic side,	No		✓
LIN28A	HUMAN Protein lin-28 homolog A	CSDD1, LIN-28, LIN28, ZCCHC1	Q9H9Z2	ENSG0000(	Others	BC028566	Mesothelioma, Malignant mesothelioma, Neoplastic cell transformation, Nephroblastoma, Breast neoplasm, Liver carcinoma, Prostate neoplasm, Epithelial ovarian cancer, Embryonal neoplasm, Acrodermatitis enteropathica	https://www.targetvalidation.org/target/ENSG00000131914/associations		Cytoplasm	No		✓
LIPI	HUMAN Lipase member I	CT17, LPDL, mPA-PLA1beta, PRED5	Q6XZB0	ENSG0000(	Others	JN387912	Hyperlipoproteinemia type 4, Papillary thyroid carcinoma, Stroke, Ewings sarcoma, Sarcoma, Diffuse large B-cell lymphoma, Renal carcinoma	https://www.targetvalidation.org/target/ENSG00000188992/associations		Membrane, Secreted,	No		✓
LMNA	HUMAN Prelamin-A/C	CMD1A, HGPS, LGMD1B, LMN1	P02545	ENSG0000(	Cancer,Cancer-Antigen	M13452	Cardiomyopathy, Progeria, Polycystic ovary syndrome, Limb-girdle muscular dystrophy, Mandibuloacral dysplasia, Emery-Dreifuss muscular dystrophy, Familial cardiomyopathy, Charcot-Marie-Tooth disease type 2B1, Premature aging, Lipodystrophy	https://www.targetvalidation.org/target/ENSG00000160789/associations	CDK12 CDK19 CDK20 CDK10 CDK13 CDK18 PRKCE CDK17 CDK16 CDK5 CDK6 CDK3 CASP6 CDK9 CDK7 CDK8 AKT1 CASP1 CDK2 PRKCA SRC CDK4 CDK1 PRKCB ISG15 PRKCG FOS CHEK1 ACTB SREBF1	Nucleus, Nucleus envelope, Nucleus lamina, Nucleus, nucleoplasm,	No		✓
LMO2	HUMAN Rhombotin-2	RBTN2, RBTNL1, RHOM2, TTG2	P25791	ENSG0000(	Transcription factor,Cancer	BC042426	Lung carcinoma, Melanoma, T-cell leukemia, B-cell lymphoma, Hodgkin's disease, Follicular lymphoma, Precursor cell lymphoblastic leukemia lymphoma, Acute myelocytic leukemia, Cutaneous melanoma, Esophageal squamous cell carcinoma	https://www.targetvalidation.org/target/ENSG00000135363/associations	ERG KDM5A RELA AFDN TAL1 LDB1 BEX2 ELF2	Nucleus	No		✓
LNX1	HUMAN E3 ubiquitin-protein ligase LNX	LNX, MPDZ, PDZRN2	Q8TBB1	ENSG0000(	Others	AC058822	Esophageal squamous cell carcinoma, Central nervous system cancer, Glioma, Glioblastoma, Mucocutaneous lymph node syndrome, Nervous system neoplasm, Lung carcinoma, Teratozoospermia, Renal cell carcinoma, Adams-Oliver syndrome	https://www.targetvalidation.org/target/ENSG00000072201/associations	HSPB8 CXADR RHOC NUMB	Cytoplasm	No		✓
LRRFIP2	HUMAN Leucine-rich repeat flightless-interacting protein 2	LRRFIP2	Q9Y608	ENSG0000(	Transcription factor	AK302282	Psoriasis, Breast carcinoma, Lynch syndrome, Hereditary nonpolyposis colorectal cancer, Melanoma, Anaplastic large cell lymphoma, Infalmmation, Prostate carcinoma, Peste des petits ruminants iinfectious disease, Non-small cell lung carcinoma	https://www.targetvalidation.org/target/ENSG00000093167/associations	MYD88 DVL3		No		✓

LSM1	HUMAN U6 snRNA-associated Sm-like protein LSm1	CASM, YJL124C	O15116	ENSG0000(	Cancer,Cancer-Antigen	CH471080	Schizophrenia, Rare genetic vascular disease, Familial thoracic aortic aneurysm and aortic dissection, Non-syndromic congenital cataract, Early-onset non-syndromic cataract, Pancreatic neoplasm, Lung carcinoma, Prostate carcinoma, Breast carcinoma, Prostatic neoplasm	https://www.targetvalidation.org/target/ENSG00000175324/associations	LSM4 LSM5 XRN1 DCP2 LSM6 LSM3 LSM2 APPBP2	Cytoplasm	No		✓
LTA4H	HUMAN Leukotriene A-4 hydrolase	LKHA4, LTA4	P09960	ENSG0000(	Cancer	AK298017	Teratozoospermia, Atherosclerosis, Mental depression, Depressive disorder, Asthma, Myocardial infarction, Tuberculosis, Cerebrovascular accident, Leprosy, Carotid artery disease	https://www.targetvalidation.org/target/ENSG00000111144/associations	CAMK2A	Cytoplasm,	Yes		✓
												https://www.bindingdb.org/uniprot/P09960	
LTV1	HUMAN Protein LTV1 homolog	C6orf93, dJ468K18.4, FLJ14909	Q96GA3	ENSG0000(	Cancer,Cancer-Antigen	AK027815	Squamous cell carcinoma, Breast carcinoma, Colorectal cancer, Melanoma, Lung carcinoma, Acute lymphoblastic leukemia, Ductal adenocarcinoma, Anaplastic large cell lymphoma	https://www.targetvalidation.org/target/ENSG00000135521/associations	RPS3		No		✓
LUZP4	HUMAN Leucine zipper protein 4	CT-8, CT28, HOM-TES-85	Q9P127	ENSG0000(	Others	BC128134	Multiple myeloma, Li-Fraumeni syndrome, Azoospermia, Pancreatic carcinoma, Melanoma, Malignant neoplasm of testis, Severe cutaneous adverse reaction, Breast carcinoma, Urethritis	https://www.targetvalidation.org/target/ENSG00000102021/associations		Nucleus	No		✓
LY6K	HUMAN Lymphocyte antigen 6K	CO16	Q17RY6	ENSG0000(	Others	BC001291	Lung carcinoma, Breast carcinoma, Esophageal carcinoma, Gastric carcinoma, Malignant neoplasm of testis, Bladder carcinoma, Ovaraian carcinoma, Astrocytoma, Non-small cell lung carcinoma, Tongue squamous cell carcinoma	https://www.targetvalidation.org/target/ENSG00000160886/associations		Secreted	No		✓
LY86	HUMAN Lymphocyte antigen 86	dJ80N2.1, MD-1	O95711	ENSG0000(	Others	BC038846	Heart valve disease, Calcinosis, Asthma, Waist-hip ratio, Body mass index, Mammographic density measurement, Urate measurement, Overweight body mass index status, Unipolar depression, Gastritis	https://www.targetvalidation.org/target/ENSG00000112799/associations	TLR4 TLR4 LY96 CD180	Secreted, extracellular space	No		✓
LY96	HUMAN Lymphocyte antigen 96	MD-2	Q9Y6Y9	ENSG0000(	Others	AC022868	Breast carcinoma, Sepsis, Neoplasm metastasis, Rheumatoid arthritis, Colorectal neoplasm, Inflammatory bowel disease, Gastric neoplasm, Bacterial vaginosis, Infectious pregnancy complications, Cystic fibrosis	https://www.targetvalidation.org/target/ENSG00000154589/associations	CIRBP SFTPD LYN LY86 TLR2 TLR4 TLR4 TLR2 SFTPA2 MBL2 SFTPA1	Secreted, extracellular space	Yes		✓
												https://www.drugbank.ca/biodb/polypeptides/Q9Y6Y9	
LYL1	HUMAN Protein lyl-1	bHLHa18	P12980	ENSG0000(	Transcription factor,Cancer	AC005546	Prostate carcinoma, Hepatocellular carcinoma, Gastric adenocarcinoma, Lymphoid leukemia, Colorectal adenocarcinoma, Bile duct carcinoma, Malignant rhabdoid tumor, Wiskott-Aldrich syndrome, MYH9-related disease, Pseudo-von Willebrand disease	https://www.targetvalidation.org/target/ENSG00000104903/associations	MAPK1 MAPK3 TCF3 NFKB1 DRG1	Nucleus	No		✓
LYN	HUMAN Tyrosine-protein kinase Lyn	JTK8	P07948	ENSG0000(	Kinase,Cancer,Cancer-Antigen	BC075002	Acute lymphoblastic leukemia, Chronic myelogenous leukemia, HIV infection, Systemic lupus erythematosus, Lymphoma, B-Cell L\lymphoma, Mammary neoplasm, Breast carcinoma, Colorectal adenocarcinoma, Lung carcinoma	https://www.targetvalidation.org/target/ENSG00000254087/associations	PAG1 GP6 MAP3K3 DOK1 MAP4K1 INPP5D PTK2B PTPRJ BTK PRKCD PTK2 PRKCQ PLAUR MS4A2 PRKDC DLG4 CSNK2B PIK3CG SYK TEC MATK CSK CD79B CSF2RB FCGR2B FCER1G PTPN6 MAPK3 CDK2 CBL RASA1 EPOR PTPRA	Cell membrane, Nucleus, Cytoplasm, perinuclear region, Golgi apparatus, Membrane	Yes		✓
												https://www.bindingdb.org/uniprot/P07948	

LYPD6B	HUMAN Ly6/PLAUR domain-containing protein 6B	LYPD6B	Q8NI32	ENSG0000	Others	CH471058	Li-Fraumeni syndrome, Hepatocellular carcinoma, Atypical hemolytic-uremic syndrome, Maple syrup urine disease, Lymphoma, Breast carcinoma, Diffuse large B-cell lymphoma, Alport syndrome, Familial dysautonomia, Glioblastoma multiforme	https://www.targetvalidation.org/target/ENSG00000150556/associations		Cell membrane	No	✓
LZTR1	HUMAN Leucine-zipper-like transcriptional regulator 1	BTBD29, LZTR-1	Q8N653	ENSG0000	Cancer	D38496	Neurofibromatosis type 3, Noonan syndrome, Adenocarcinoma, Schwannomatosis, Thickened helices, Prominent eye, Prominent lower lip, Cardiac conduction abnormalities, Coagulation abnormalities, Midface retrusion	https://www.targetvalidation.org/target/ENSG00000099949/associations	BMPR1B		No	✓
MAEL	HUMAN Protein maelstrom homolog	CT128, FLJ14904, SPATA35	Q96JY0	ENSG0000	Others	AK302253	Colonic neoplasm, Colorectal adenocarcinoma, Li-Fraumeni syndrome, Hypertensive disease, Osteoporosis, Colorectal carcinoma, Ovarian carcinoma, Trichohepatoenteric syndrome, Astigmatism, Azoospermia	https://www.targetvalidation.org/target/ENSG00000143194/associations		Cytoplasm, Nucleus,	No	✓
MAFG	HUMAN Transcription factor MafG	MGC13090, MGC20149	O15525	ENSG0000	Transcription factor	BC012327	Hereditary thrombocytopenia with normal platelets, X-linked dyserythropoetic anemia with abnormal platelets and neutropenia, Autosomal dominant macrothrombocytopenia, Intrahepatic cholestasis, Lung neoplasm, Colorectal cancer, Chronic myeloid leukemia, Prostate carcinoma, MYH9-related disease, Pseudo-von Willebrand disease	https://www.targetvalidation.org/target/ENSG00000197063/associations	CREBBP NFE2L2 MAF BACH1 NFE2L2 JUN BRIP1 NFE2 NFE2L1	Nucleus	No	✓
MAGEA10	HUMAN Melanoma-associated antigen 10	CT1.10, MAGE10	P43363	ENSG0000	Others	NA	Li-Fraumeni syndrome, Tongue squamous cell carcinoma, Pancreatic ductal adenocarcinoma, Melanoma, Azoospermia, Pancreatic ductal adenocarcinoma, Monoclonal gammopathy, Plasmacytoma, Non-small cell lung carcinoma, Colorectal adenocarcinoma	https://www.targetvalidation.org/target/ENSG00000124260/associations		Nucleus	No	✓
MAGEA4	HUMAN Melanoma-associated antigen 4	CT1.4, MAGE-41, MAGE-X2, MAGE4	P43358	ENSG0000	Cancer,Cancer-Antigen	D32075	Li-Fraumeni syndrome, Colorectal adenocarcinoma, Prostatic hyperplasia, Non-small cell lung carcinoma, Squamous cell carcinoma, Transitional cell carcinoma, Lung neoplasm, Prostatic neoplasm, Malignant neoplasm of testis, Azoospermia	https://www.targetvalidation.org/target/ENSG00000147381/associations	HLA-A ZBTB17		No	✓
MAGEB1	HUMAN Melanoma-associated antigen B1	CT3.1, DAM10, MAGE-Xp, MAGEL1	P43366	ENSG0000	Others	AK314977	Li-Fraumeni syndrome, Colorectal adenocarcinoma, Medulloblastoma, Renal cell carcinoma, X-linked adrenal hypoplasia congenita, Tongue squamous cell carcinoma, Benign prostatic hyperplasia, Acute lymphoblastic leukemia, Renal cell cacinoma	https://www.targetvalidation.org/target/ENSG00000214107/associations			No	✓
MAGEB2	HUMAN Melanoma-associated antigen B2	CT3.2, DAM6, MAGE-XP-2	O15479	ENSG0000	Others	BC026071	Ovarian carcinoma, Medulloblastoma, Tongue squamous cell carcinoma, Melanoma, Renal cell carcinoma, Acute myelocytic leukemia, Systemic lupus erythematosus, Neurofibromatosis 1, Malignant peripheral nerve sheath tumor, Head and Neck squamous cell carcinoma	https://www.targetvalidation.org/target/ENSG00000099399/associations			No	✓
MAGEB3	HUMAN Melanoma-associated antigen B3	MAGEB3	O15480	ENSG0000	Others	BC069784	Li-Fraumeni syndrome	https://www.targetvalidation.org/target/ENSG00000198798/associations			No	✓

MAGEB4	HUMAN Melanoma-associated antigen B4	MAGEB4	O15481	ENSG0000(	Others	AC005185	Glioblastoma multiforme, Sjogren's syndrome	https://www.targetvalidation.org/target/ENSG00000120289/associations		Cytoplasm		No		✓
MAGEB5	HUMAN Melanoma-associated antigen B5	CT3.3, MAGE-B5	Q9BZ81	ENSG0000(	Cancer	NA	Colorectal cancer, MHPG measurement, HVA measurement, 5-HIAA measurement	https://www.targetvalidation.org/target/ENSG00000188408/associations				No		✓
MAGEB6	HUMAN Melanoma-associated antigen B6	CT3.4, MAGE-B6	Q8N7X4	ENSG0000(	Cancer	AF320514	Diabetes mellitus, Melanoma, Azoospermia, Esophagus adenocarcinoma, Schizophrenia, Multiple myeloma, Squamous cell carcinoma, Rectal carcinoma, Squamous cell carcinoma, Gastric neoplasm, Head and Neck squamous cell carcinoma	https://www.targetvalidation.org/target/ENSG00000176746/associations				No		✓
MAGEC1	HUMAN Melanoma-associated antigen C1	CT7, CT7.1, MAGE-C1	O60732	ENSG0000(	Cancer	BC127771	Tongue squamous cell carcinoma, Li-Fraumeni syndrome, Melanoma, Malignant neoplasm of testis, Non-small cell lung carcinoma, Oral cavity carcinoma, Precursor B-cell lymphoblastic leukemia, Glioblastoma multiforme	https://www.targetvalidation.org/target/ENSG00000155495/associations				No		✓
MAGEC2	HUMAN Melanoma-associated antigen C2	CT10, MAGE-C2, MAGEE1, HCA587	Q9UBF1	ENSG0000(	Cancer	AF196483	Hepatocellular carcinoma, Tongue squamous cell carcinoma, Melanoma, Liver carcinoma, Squamous cell carcinoma, Multiple myeloma, Sarcoma, Lung neoplasm, Glioblastoma multiforme, Li-Fraumeni syndrome	https://www.targetvalidation.org/target/ENSG00000046774/associations	TRIM28	Cytoplasm		No		✓
MAGEC3	HUMAN Melanoma-associated antigen C3	CT7.2, HCA2, MAGE-C3	Q8TD91	ENSG0000(	Cancer	AL049177, AL022152, AL023279	Li-Fraumeni syndrome, Papillary thyroid carcinoma, Osteopetrosis, Metabolic bone disorder, Autosomal recessive 3 osteopetrosis	https://www.targetvalidation.org/target/ENSG00000165509/associations				No		✓
MAK	HUMAN Serine/threonine-protein kinase MAK	MAK	P20794	ENSG0000(	Kinase	AL024498	Retinitis pigmentosa, Azoospermia, Non-syndromic male infertility due to sperm motility disorder, Deafness-infertility syndrome, Central nervous system cancer, Progressive night blindness, Lens opacities, Mental deficiency, Pallor of optic disc, Anteverted nostril	https://www.targetvalidation.org/target/ENSG00000111837/associations	AR CBL	Nucleus, Cytoplasm, cytoskeleton, microtubule organizing center, centrosome, spindle, Midbody, Cell projection, cilium, photoreceptor outer segment Photoreceptor inner segment,		Yes	https://www.bindingdb.org/uniprot/P20794	✓
MAL	HUMAN Myelin and lymphocyte protein	MAL	P21145	ENSG0000(	Others	X76680	Esophageal squamous cell carcinoma, Central nervous system cancer, Squamous cell carcinoma, Mammary neoplasm, Head and Neck neoplasm, Rheumatoid arthritis, Colorectal neoplasm, Adenoma, Epithelial ovarian cancer, Acute megakaryocytic leukemia	https://www.targetvalidation.org/target/ENSG00000172005/associations	TLR4 MYD88	Membrane, Multi-pass membrane protein,	Transmembrane	No		✓
MALL	HUMAN MAL-like protein	BENE	Q13021	ENSG0000(	Others	U17077	Li-Fraumeni syndrome, Lung carcinoma, Squamous cell carcinoma, Esophageal squamous cell carcinoma, Colorectal cancer, Adenoma, Neural tube defect, Nephronophthisis, Colon carcinoma, Azoospermia	https://www.targetvalidation.org/target/ENSG00000144063/associations	CAV1 CAV2	Membrane	Transmembrane	No		✓
MAP2K1	HUMAN Dual specificity mitogen-activated protein kinase kinase 1	MAPKK1, MEK1, PRKMK1, MP2K1	Q02750	ENSG0000(	Kinase,Cancer	NA	Cardiofaciocutaneous syndrome, Melanoma, Noonan syndrome, Mental retardation, Costello syndrome, Lung adenocarcinoma, Malignant peripheral nerve sheath tumor, Unipolar depression, Treatment-resistant depressive disorder, Hairy cell leukemia	https://www.targetvalidation.org/target/ENSG00000169032/associations	MAP3K2 LAMTOR3 CDK12 PLK3 CDK19 WDR83 TRIB1 TRIB3 CDK20 KSR1 BRAP MAPK6 MAP3K11 MAPK14 RPS6KA2 CDK10 PDK1 CDK13 ATR MAP3K1 PAK1 CDK18 PRKCD PRKCZ CDK17 CDK16 CDK5 CDK6 CDK3	Cytoplasm, cytoskeleton, microtubule organizing center, centrosome		Yes	https://www.bindingdb.org/uniprot/Q02750	✓

MAP2K3	HUMAN Dual specificity mitogen-activated protein kinase kinase 3	MAPKK3, MEK3, MKK3, PRKMK3, SKK2	P46734	ENSG00000	Kinase	U66840	Anthrax infection, Cervical carcinoma, Retinal disease, Myocardial ischemia, Mammary neoplasm, Rheumatoid arthritis, Van der Woude syndrome, Sepsis, Osteosarcoma, Lung carcinoma	https://www.targetvalidation.org/target/ENSG00000034152/associations	MAP3K4<br>DYRK1B<br>TAOK2<br>MAP3K20<br>MAP3K3<br>MAP3K5<br>TAOK1<br>MAP3K11<br>MAPK14<br>PKN1<br>PAK2<br>PAK1<br>PLCB2<br>MAPK12<br>MAP2K6<br>ARRB1<br>MAP3K8<br>MAPK1<br>MAPK3<br>MAP3K7<br>SMAD7<br>PLCB2<br>MAP3K4<br>SMAD4<br>MAPK1<br>SMAD3<br>MAPK14<br>MAPK13<br>SMAD1		Yes	https://www.bindingdb.org/uniprot/P46734	✓
MAP2K5	HUMAN Dual specificity mitogen-activated protein kinase kinase 5	MAPKK5, MEK5, PRKMK5, MKK5	Q13163	ENSG00000	Kinase	BT006780	Restless legs syndrome, Motion sickness, Gilles de la Tourette syndrome, Nocturnal myoclonus syndrome, Amphetamine-Related disorder, Depressive disorder, Prostate carcinoma, Obesity, Colonic neoplasm, Posterior cortical atrophy	https://www.targetvalidation.org/target/ENSG00000137764/associations	MAP3K2<br>MAP3K20<br>MAP3K3<br>MAPK7<br>PRKCZ<br>PRKCI<br>MAP3K8<br>PRKCZ<br>MAP3K2<br>PRKCI<br>MAP3K3<br>MAPK7<br>SQSTM1<br>BAD<br>STAMBP		Yes	https://www.bindingdb.org/uniprot/Q13163	✓
MAP2K6	HUMAN Dual specificity mitogen-activated protein kinase kinase 6	MAPKK6, MEK6, MKK6, PRKMK6, SAPKK3, SKK3	P52564	ENSG00000	Kinase	U39065	Anthrax infection, Li-Fraumeni syndrome, Hypersensitivity, Colonic neoplasm, Cardiomyopathy, Huntington disease, Rheumatoid arthritis, Lung neoplasm, Parkinson's disease, Melanoma	https://www.targetvalidation.org/target/ENSG00000108984/associations	MAP3K4<br>TRAF6<br>TAOK2<br>MAP3K20<br>MAP3K5<br>MAP3K11<br>MAPK14<br>PKN1<br>MAP3K1<br>MAP3K12<br>PLCB2<br>MAPK12<br>MAP2K3<br>MAP3K8<br>PPM1A<br>GADD45A<br>SRC<br>MAP3K7<br>MAPK13<br>SMAD7<br>PLCB2<br>AQP4<br>PRKAA2<br>MAP3K7<br>MAP3K4<br>MAPK8<br>PRKCD<br>BID<br>NFE2L2	Nucleus	Yes	https://www.bindingdb.org/uniprot/P52564	✓
MAP2K7	HUMAN Dual specificity mitogen-activated protein kinase kinase 7	Jnkk2, MKK7, PRKMK7, MEK7, SKK4	O14733	ENSG00000	Kinase	AF013589	Anthrax infection, Lung carcinoma, Hepatocellular carcinoma, Schizophrenia, Heart failure, Fibrosis, Precancerous condition, Melanoma, Colorectal carcinoma, Leukemia	https://www.targetvalidation.org/target/ENSG00000076984/associations	MAP3K2<br>MAPK8IP1<br>MAPK8IP3<br>TAOK2<br>MAP3K20<br>MAP3K5<br>TRIB1<br>TRIB3<br>SH3RF1<br>MAP3K11<br>MAPK8IP2<br>MAP3K1<br>FADD<br>MAP3K12<br>MAP3K10<br>MAPK10<br>NEK2<br>MAP2K4<br>MAPK9<br>MAPK8<br>BCL10<br>RIPK2<br>MAP3K7<br>MAP3K13<br>CDK5<br>MAP3K7<br>MAPK8<br>MAPK8IP1<br>MAPK9	Nucleus, Cytoplasm	Yes	https://www.bindingdb.org/uniprot/O14733	✓
MAP3K13	HUMAN Mitogen-activated protein kinase kinase kinase 13	LZK	O43283	ENSG00000	Kinase,Cancer	AK302951	Adenocarcinoma, Lung carcinoma, Melanoma, Parkinson's disease, Congestive heart failure, Cardiac fibrosis, Colorectal cancer, Bladder carcinoma, Kidney neoplasm, Bile duct carcinoma	https://www.targetvalidation.org/target/ENSG00000073803/associations	IKBKB<br>MAPK8IP1<br>MAP2K7<br>PRDX3	Cytoplasm	Yes	https://www.bindingdb.org/uniprot/O43283	✓
MAP3K14	HUMAN Mitogen-activated protein kinase kinase kinase 14	FTDCR1B, HSNIK, NIK	Q99558	ENSG00000	Kinase,Transcription factor,Signalling	CH471178	Primary immunodeficiency, Hyper-IgM syndrome without susceptibility to opportunistic infections, Sjogren's syndrome, Primary biliary cirrhosis, Rheumatoid arthritis, Down syndrome, Prostatic neoplasm, Peutz-Jeghers syndrome, Breast carcinoma, Tuberculosis	https://www.targetvalidation.org/target/ENSG00000006062/associations	IKBKG<br>TRAF6<br>TNFSF13B<br>NOD2<br>ZFP91<br>CASP10<br>WASF1<br>CDC37<br>CASP8<br>RIPK1<br>BIRC2<br>BIRC3<br>MAP3K1<br>TRAF3<br>TRAF1<br>TRAF2<br>REL RPL6<br>NFKB2<br>RPL8<br>RPL30<br>RPS11<br>RPS13<br>NLRP12<br>ARRB1<br>MAP2K4<br>MAP3K8<br>RPL3<br>LTBR<br>RPL4	Cytoplasm,	Yes	https://www.bindingdb.org/uniprot/Q99558	✓
MAP3K2	HUMAN Mitogen-activated protein kinase kinase kinase 2	MEKK2, MEKK2B, M3K2, MAPKKK2	Q9Y2U5	ENSG00000	Kinase	AC068282	Melanoma, Rheumatoid arthritis, Neoplasm metastasis, Adenocarcinoma, Hepatocarcinogenesis, Degenerative polyarthritis, Liver neoplasm, Lung carcinoma, Juvenile dermatomyositis, Small cell lung carcinoma	https://www.targetvalidation.org/target/ENSG00000169967/associations	IKBKG<br>SMURF1<br>WNK1<br>PKN2<br>STK38<br>MAP2K5<br>XIAP<br>MAP2K4<br>MAPK8<br>MAP2K7<br>YWHAG<br>MAPK8<br>MAPK1<br>PKN2<br>MAP2K1<br>YWHAZ<br>NFKBIA<br>MAP3K5<br>MAP2K5<br>IKBKG<br>MAP3K3<br>MAP2K4<br>MAP2K7<br>YWHAE<br>YWHAB<br>MAPKAP1<br>PROK2	Cytoplasm	Yes	https://www.bindingdb.org/uniprot/Q9Y2U5	✓
MAP3K20	HUMAN Mitogen-activated protein kinase kinase kinase 20	MLTK, ZAK,HCCS4	Q9NYL2	ENSG0000	Kinase,Signalling	AF325454	Split-foot malformation with mesoaxial polydactyly, B-cell lymphoma, Colon tumor, Acute myelocytic leukemia, Lung neoplasm, Mucosa-Associated lymphoid tissue lymphoma	https://www.targetvalidation.org/target/ENSG00000091436/associations	PKN1<br>STK11<br>YWHAZ<br>MAP2K6<br>MAP2K4<br>TGFBR1<br>RPS6KA5<br>MAP2K7<br>YWHAG<br>MAP2K3<br>MAP2K5<br>CHEK2<br>MAP2K4<br>MAP2K7<br>TGFBR1<br>HIST1H3A;<br>HIST1H3B;<br>HIST1H3C;<br>HIST1H3D;<br>HIST1H3E;<br>HIST1H3F;<br>HIST1H3G<br>;<br>HIST1H3H;<br>HIST1H3I;<br>HIST1H3J<br>MAP2K6<br>ZNF567<br>ZNF33A	Cytoplasm	Yes	https://www.bindingdb.org/uniprot/Q9NYL2	✓

MAP3K6	HUMAN Mitogen-activated protein kinase kinase kinase 6	ASK2, MAPKKK6, MEKK6, M3K6	O95382	ENSG0000(	Kinase	BC129950	Breast carcinoma, Psoriasis, Squamous cell lung carcinoma, Hereditary diffuse gastric cancer, Huntington disease, Influenza, Gastric carcinoma, Cutaneous lupus erythematosus, Familial gastric cancer, Glioma	https://www.targetvalidation.org/target/ENSG00000142733/associations	MAP3K5<br>YWHAG<br>MAPK8<br>SFN<br>YWHAQ<br>MAPK14<br>YWHAZ<br>MAP3K5<br>MAP2K4<br>YWHAH<br>YWHAE<br>YWHAB		Yes	https://www.bindingdb.org/uniprot/O95382	✓
MAP3K7	HUMAN Mitogen-activated protein kinase kinase kinase 7	TAK1, M3K7	O43318	ENSG0000(	Kinase,Signalling	AF218074	Cardiospondylocarpofacial syndrome, Frontometaphyseal dysplasia, Non-syndromic congenital cataract, Early-onset non-syndromic cataract, Nuclear cataract, Joint stiffness, Acquired scoliosis, Long fingers, Curvature of spine, Short thumb terminal phalanx	https://www.targetvalidation.org/target/ENSG00000135341/associations	TNFRSF11A IKBKG<br>TRAF6<br>TAOK2<br>MALT1<br>NLK TAB2<br>SMURF1<br>NOD2<br>HIPK2<br>STRADB<br>RBCK1<br>GTPBP2<br>MAP3K3<br>MAP3K5<br>MAP3K14<br>PELI1<br>WDR34<br>FBXW5<br>MAP4K1<br>TAB3<br>TAOK1<br>PPM1L<br>CDC37<br>MAPK14<br>TAB1<br>CAMK2D<br>CAMK2G<br>CAMK2B<br>RIPK1	Cytoplasm	Yes	https://www.bindingdb.org/uniprot/O43318	✓
MAP4	HUMAN Microtubule-associated protein 4	MAP4	P27816	ENSG0000(	Signalling	AK125245	Astrocytoma, Self-reported educational attainment, Ependymoma, B-Cell lymphoma, Schizophrenia, Adenovirus infection, Bladder carcinoma, Diffuse large B-Cell lymphoma, Peripheral neuropathy, Dwarfism	https://www.targetvalidation.org/target/ENSG00000047849/associations	MARK1<br>ERBIN<br>MARK4<br>MARK2<br>MARK3<br>CCNB1<br>TUBB<br>CDK1<br>ISG15<br>CHEK1	Cytoplasm, cytoskeleton,	Yes	https://www.drugbank.ca/biodb/polypeptides/P27816	✓
MAP4K3	HUMAN Mitogen-activated protein kinase kinase kinase kinase 3	GLK, MAPKKKK3, RAB8IPL1, M4K3	Q8IVH8	ENSG0000(	Kinase,Cancer,Cancer-Antigen	CH471053	Glioblastoma multiforme, Macroglobulinemia, Melanoma, Atopic eczema, Breast carcinoma, Adult-Onset still disease, Amyotrophic lateral sclerosis, Systemic lupus erythematosus, Temporomandibular joint disorder, Degenerative polyarthritis	https://www.targetvalidation.org/target/ENSG00000011566/associations	MAP3K4<br>MAP3K3<br>TRAF2<br>MAP3K1<br>MAPK14<br>TRAF2<br>PRKCQ<br>MAP3K3<br>BIN1<br>SH3GL2		Yes	https://www.bindingdb.org/uniprot/Q8IVH8	✓
MAP4K5	HUMAN Mitogen-activated protein kinase kinase kinase kinase 5	KHS	Q9Y4K4	ENSG0000(	Kinase	NA	Glutathione synthetase deficiency, Melanoma, Glutathione synthetase deficiency without 5-oxoprolinuria, Autosomal dominant severe congenital neutropenia, Psoriasis, Heart failure, Pancreatic carcinoma, Pancreatic ductal adenocarcinoma, Down syndrome, Atopic eczema	https://www.targetvalidation.org/target/ENSG00000012983/associations	MYLK2<br>TRAF2<br>BCR<br>STUB1<br>CRKL CRK<br>GSK3B<br>TRAF2<br>ABL1<br>GRB2<br>DOK1<br>TANK	Cytoplasm	Yes	https://www.bindingdb.org/uniprot/Q9Y4K4	✓
MAPK1	HUMAN Mitogen-activated protein kinase 1	ERK2, MAPK2, PRKM1, PRKM2	P28482	ENSG0000(	Kinase,Cancer	DQ399292	Adenocarcinoma, Lymphoid neoplasm, Neoplasm metastasis, Squamous cell carcinoma, Ovarian neoplasm, Lung neoplasm, Bipolar disorder, Sepsis, Schizophrenia, Cardiomyopathy	https://www.targetvalidation.org/target/ENSG00000100030/associations	DYRK1B<br>MAP3K2<br>HSF4<br>LAMTOR3<br>DUSP22<br>NOD2<br>MKNK2<br>WNK1<br>DUSP16<br>MKNK1<br>DUSP9<br>MAP3K5<br>CMTM3<br>CNKSR1<br>MAP4K1<br>STAB2<br>FRS2<br>MAPKAPK5 KSR1<br>DUPD1<br>DUSP7<br>DUSP6<br>DUSP5<br>MAPK14<br>RPS6KA1<br>RPS6KA2<br>PTPRR<br>PEA15<br>TNIP1	Cytoplasm, cytoskeleton, spindle	Yes	https://www.bindingdb.org/uniprot/P28482	✓
MAPK10	HUMAN Mitogen-activated protein kinase 10	JNK3, JNK3A, SAPK1B, p493F12, PRKM10	P53779	ENSG0000(	Kinase	AK057723	Migraine disorder, Autosomal dominant secondary polycythemia, Inborn errors of metabolism, Intellectual disability, Hyperactive behavior, Dull intelligence, Epileptic drop attack, Tonic-clonic seizures, Mental deficiency, Autistic behavior	https://www.targetvalidation.org/target/ENSG00000109339/associations	DUSP10<br>MAPK8IP3<br>MAD2L2<br>DUSP22<br>DUSP16<br>MAPK8IP2<br>CDK5<br>MAP2K6<br>ARRB1<br>MAPKAPK2 MAP2K4<br>ARRB2<br>DUSP1<br>IL1R1 JUN<br>APP<br>MAP2K7<br>STMN1<br>PPARA<br>SNTA1<br>LCK<br>STMN2<br>ARRB2<br>TCF3<br>MAPK8IP1<br>STUB1<br>TP53 APP<br>SFN<br>SH3BP5<br>RPS6KA1	Cytoplasm	Yes	https://www.bindingdb.org/uniprot/P53779	✓
MAPK11	HUMAN Mitogen-activated protein kinase 11	p38-2, p38Beta, PRKM11, SAPK2, SAPK2B	Q15759	ENSG0000(	Kinase	AF031135	Colorectal cancer, Metastatic colorectal cancer, Cardiomyopathy, HIV Infection, Burkitt lymphoma, Non-small cell lung carcinoma, Glioblastoma, Diffuse large B-Cell lymphoma, Human immunodeficiency virus II infection, Metabolic syndrome X	https://www.targetvalidation.org/target/ENSG00000185386/associations	DUSP10<br>DUSP16<br>ESR2<br>MAPKAPK5 MAPK14<br>TAB1<br>MAP2K6<br>MAP2K3<br>MAP2K4<br>DUSP1<br>RPS6KA5<br>SPAG9<br>EEF2K<br>EGFR<br>PRKCE<br>HIF1A<br>E2F1<br>TAB1<br>TCF3<br>NFE2L2<br>TP53<br>HBP1<br>KRT8<br>MAPK14<br>RPS6KA5<br>JUN<br>MAPKAPK3 BCL2<br>DUSP1	Cytoplasm	Yes	https://www.bindingdb.org/uniprot/Q15759	✓

MAPK12	HUMAN Mitogen-activated protein kinase 12	ERK6, p38gamma, SAPK-3, SAPK3	P53778	ENSG0000(	Kinase	CR456515	Breast carcinoma, Juvenile dermatomyositis, Inflammation, Breast neoplasm, Lung carcinoma, Lip and Oral cavity carcinoma, Melanoma, Squamous cell carcinoma, Tauopathy, Rectum cancer	https://www.targetvalidation.org/target/ENSG00000188130/associations	ERBIN MAPKAPK5 PKN1 SNTA1 DLG1 MAP2K6 MAP2K3 MAP2K4 PTPN4 DUSP1 PTPN3 PPM1D EEF2K CTNNB1 SNTA1 NFE2L2 KRT8 SH3BP5 MAP2K3 DUSP1 MTA1 MAPKAPK5 MAP2K4 HTRA2 GRB2 MAPT ATF2	Cytoplasm, Nucleus, Mitochondrion,	Yes	https://www.bindingdb.org/uniprot/P53778	✓
MAPK13	HUMAN Mitogen-activated protein kinase 13	p38delta, PRKM13, SAPK4	O15264	ENSG0000(	Kinase	AF004709	Li-Fraumeni syndrome, Familial hyperinsulinism, Familial glucocorticoid deficiency, Cholestasis, Neuronal ceroid-lipofuscinoses, Rheumatoid arthritis, HIV infection, Glioma, Cholangiocarcinoma, Peritoneal mesothelioma	https://www.targetvalidation.org/target/ENSG00000156711/associations	MAP2K6 CCND3 MAPKAPK5 MAPKAPK3 MAPK8IP2 MAP2K6 MAP2K3 APP EEF2K STMN1 PRKD1 MAP3K4 SMAD4 NFE2L2 TP53 KRT8 APP MAPKAPK3 MAPKAPK5 SQSTM1 CHEK1 MAPT ATF2 MAP2K6 CCND3 MAPK8IP2 EEF2K MBP RCSD1 DUSP10 MAP3K4		Yes	https://www.bindingdb.org/uniprot/O15264	✓
MAPK14	HUMAN Mitogen-activated protein kinase 14	CSBP, CSBP1, CSBP2, CSPB1, MXI2, SAPK2A, p38	Q16539	ENSG0000(	Kinase,Cancer	U19775	Acute coronary syndrome, Arthritis, Schizophrenia, Cardiomyopathy, Lung neoplasm, Chronic myeloid leukemia, Myocardial ischemia, Cholestasis, Liver neoplasm, Dysphoric mood	https://www.targetvalidation.org/target/ENSG00000112062/associations	TRAF6 DYRK1B NOD1 MAPK8IP1 STK39 DUSP22 NOD2 MKNK2 CARD9 DUSP16 DUSP26 MKNK1 MAPKAP1 DUSP9 MAP3K5 MINK1 MAPKAPK5 MAP4K3 DUPD1 CDON DUSP7 DUSP5 MAPKAPK3 PKN1 MAPK11	Cytoplasm	Yes	https://www.bindingdb.org/uniprot/Q16539	✓
MAPK3	HUMAN Mitogen-activated protein kinase 3	ERK1, p44erk1, p44mapk, PRKM3	P27361	ENSG0000(	Kinase,Cancer	EU332853	Phenotypic abnormality, Ischemia, Neoplasm metastasis, Squamous cell carcinoma, Prostatic neoplasm, Ovarian neoplasm, Cardiomyopathy, Schizophrenia, Lung neoplasm, Depressive disorder	https://www.targetvalidation.org/target/ENSG00000102882/associations	TAB1 PTPRR DUSP10 HSPB8 LAMTOR3 DUSP23 MKNK1 WDR83 DUSP9 MAP3K14 DKFZp686L02273 DUPD1 DUSP6 DUSP5 MAPK14 NCOA1 MYLK RPS6KA1 RPS6KA2 PTPRR PEA15 SQSTM1 GAB1 PPP2R5C MAP3K1 DUSP4 PTPRJ SOS1 PTPN11 DUSP2	Cytoplasm, Nucleus, Membrane, caveola	Yes	https://www.bindingdb.org/uniprot/P27361	✓
MAPK6	HUMAN Mitogen-activated protein kinase 6	ERK3, p97MAPK, PRKM6	Q16659	ENSG0000(	Kinase	AK313633	Congenital pulmonary alveolar proteinosis, Newborn respiratory distress syndrome, Congenital fiber-type disproportion myopathy, Acute infantile liver failure-multisystemic involvement syndrome, Renal-hepatic-pancreatic dysplasia, Hyperplasia, Squamous cell carcinoma, Melanoma, Lung neoplasm, Breast carcinoma	https://www.targetvalidation.org/target/ENSG00000069956/associations	CDC14A NLK MAPK15 DUSP6 PAK2 MAPK7 PAK1 MERTK MAP2K1 MAPK10 DAPK1 MAPK9 MAPK8 MAP2K2 MAPK4 MAPK1 MAPK3 CDK1 PRKCB CDC14B STMN1 PPARA SNTA1 LCK STMN2 MAP2K2 TCF3 IER3 TP53 MAP2K1	Cytoplasm	Yes	https://www.bindingdb.org/uniprot/Q16659	✓
MAPK7	HUMAN Mitogen-activated protein kinase 7	BMK1, ERK5, PRKM7	Q13164	ENSG0000(	Kinase,Cancer	U25278	Left ventricular noncompaction, Hyperalgesia, Neoplasm metastasis, Hodgkin's disease, Squamous cell carcinoma, Mammary neoplasm, Lung neoplasm, Multiple myeloma, Breast carcinoma, Colonic neoplasm	https://www.targetvalidation.org/target/ENSG00000166484/associations	RPS6KA1 PTPRR MAP2K5 PRKCZ CDK5 MAPK1 GJA1 APP SGK1 GJA1 STMN1 PPARA SNTA1 LCK STMN2 RAF1 TCF3 MAPK8 NFE2L2 TP53 APP RPS6KA1 TRIM28 BCL2 HSF1 NR3C1 LIFR MAP2K5 MYB SGK1 FOS CDKN1A	Cytoplasm, Nucleus, Nucleus, PML body,	Yes	https://www.bindingdb.org/uniprot/Q13164	✓
MAPK8_tv1	HUMAN Mitogen-activated protein kinase 8	JNK, JNK1, PRKM8, SAPK1	P45983	ENSG0000(	Kinase,Cancer	U35005	Familial partial lipodystrophy, Schizophrenia, Reperfusion Injury, Depressive disorder, Atopic dermatitis, Precancerous condition, Trigeminal neuralgia, Acute liver failure, Neoplastic cell transformation, Mental depression	https://www.targetvalidation.org/target/ENSG00000107643/associations	DUSP10 TRAF6 DYRK1B MAP3K2 NOD1 MAPK8IP1 MAPK8IP3 DUSP22 NOD2 CARD9 MYLK2 DUSP16 DUSP9 MAP3K5 MAP4K1 RND1 DUSP19 DUSP18 SH3RF1 DUSP7 NFE2 HSPB2 PRKD1 NFATC4 ROCK1 MAPK8IP2 MAP3K1 DUSP8 MAPK7	Cytoplasm	Yes	https://www.bindingdb.org/uniprot/P45983	✓
MAPK8_tv2	HUMAN Mitogen-activated protein kinase 8	JNK, JNK1, PRKM8, SAPK1	P45983	ENSG0000(	Kinase,Cancer	U35005	Familial partial lipodystrophy, Schizophrenia, Reperfusion Injury, Depressive disorder, Atopic dermatitis, Precancerous condition, Trigeminal neuralgia, Acute liver failure, Neoplastic cell transformation, Mental depression	https://www.targetvalidation.org/target/ENSG00000107643/associations	DUSP10 TRAF6 DYRK1B MAP3K2 NOD1 MAPK8IP1 MAPK8IP3 DUSP22 NOD2 CARD9 MYLK2 DUSP16 DUSP9 MAP3K5 MAP4K1 RND1 DUSP19 DUSP18 SH3RF1 DUSP7 NFE2 HSPB2 PRKD1 NFATC4 ROCK1 MAPK8IP2 MAP3K1 DUSP8 MAPK7	Cytoplasm	Yes	https://www.bindingdb.org/uniprot/P45983	✓

MAPK9	HUMAN Mitogen-activated protein kinase 9	JNK2, PRKM9, SAPK1A	P45984	ENSG0000(	Kinase	U35002	Progressive familial intrahepatic cholestasis, Focal palmoplantar keratoderma, Systemic lupus erythematosus, Reperfusion injury, Ischemia, Hyperalgesia, Trigeminal Neuralgia, Myocardial reperfusion injury, Colonic neoplasm	https://www.targetvalidation.org/target/ENSG00000050748/associations	DUSP10 MAPK8IP1 MAPK8IP3 DUSP16 DUSP9 DUSP19 MAPKAPK5 NFE2 PRKD1 NFATC4 MAPK8IP2 DUSP4 TRAF2 PPARA CSNK2A1 RAC1 CDC42 MAP2K6 DUSP3 ARRB1 MAPKAPK2 MAP2K4 MAPK8 CASP3 DUSP1 IL1R1 RET LCK APP TP53 DUSP14	Cytoplasm	Yes	https://www.bindingdb.org/uniprot/P45984	✓
MAPKAPK3	HUMAN MAP kinase-activated protein kinase 3	3pK	Q16644	ENSG0000(	Kinase,Signalling	CH471055	Retinal dystrophy, Patterned dystrophy of the retinal pigment, Chronic Hepatitis C, Schizophrenia, Retinal disease, Pancreatic ductal adenocarcinoma, Breast carcinoma, Influenza, Metastatic melanoma, Hepatocellular carcinoma, Psoriasis, Proteinuria	https://www.targetvalidation.org/target/ENSG00000114738/associations	MAPK14 MAPK11 MAPK13 EEF2K TCF3 HSPB2 MAPK14 MAPK13 BMI1 BECN1 HSPB1 TAB3 CBX4 EEF2 EEF2K RCSD1 PHC2 HLCS	Nucleus	Yes	https://www.bindingdb.org/uniprot/Q16644	✓
MAPKAPK5	HUMAN MAP kinase-activated protein kinase 5	PRAK, MK5	Q8IW41	ENSG0000(	Kinase	BC000833	Arthritis, Rheumatoid arthritis, Epidermodysplasia verruciformis, Alzheimer's disease, Hepatitis C infection, Hepatocellular carcinoma, Colorectal adenocarcinoma, Inflammation, Bronchoalveolar adenocarcinoma, Leukemia	https://www.targetvalidation.org/target/ENSG00000089022/associations	MAPK14 MAPK11 MAPK12 MAPK9 MAPK1 PRKACA ISG15 MAPK13 EEF2K TLR4 PRKACA NEUROD1 SMAD4 MAPK1 TP53 DNAJB1 MAPK9 NPM1 MAPK14 JUN MAPK13 DAXX EIF4E EIF4EBP1 EIF4B MAPK11 GYS1 FOXO3 RHEB CBL	Cytoplasm, Nucleus,	Yes	https://www.bindingdb.org/uniprot/Q8IW41	✓
MARK2	HUMAN Serine/threonine-protein kinase MARK2	EMK1, PAR-1, PAR-1B, Par1b	Q7KZI7	ENSG0000(	Kinase,Cancer,Cancer-Antigen	AP003780	Schizophrenia, Juvenile polyposis syndrome, Short stature due to GHSR deficiency, Atypical hemolytic-uremic syndrome, Generalized juvenile polyposis/juvenile polyposis coli, Tauopathies, Adenoid cystic carcinoma, Salivary gland neoplasm, Peutz-Jeghers syndrome, Prostate carcinoma	https://www.targetvalidation.org/target/ENSG00000072518/associations	PRKD2 TAOK1 STK11 PRKD1 CAMK1 PRKCZ DAPK1 GSK3B PRKCI YWHAQ PRKCA KIF13B HDAC4 YWHAZ MAP4 PINK1 GSK3B MAP3K3 STK11 CRTC2 MAPT DVL3 HDAC7 PARD3 HDAC5 YWHAB ARHGEF2 PARD6A DLG4	Cell membrane, Peripheral membrane protein, Cytoplasm, Lateral cell membrane, Cytoplasm, cytoskeleton,	Yes	https://www.bindingdb.org/uniprot/Q7KZI7	✓
MARK3	HUMAN MAP/microtubule affinity-regulating kinase 3	CTAK1, EMK2, KP78, PAR-1A	P27448	ENSG0000(	Kinase	AF465413	Phosphoenolpyruvate carboxykinase deficiency, Glycogen storage disease due to liver glycogen phosphorylase deficiency, Familial partial lipodystrophy, Köbberling type, Short stature - pituitary and cerebellar defects - small sella turcica, Phosphoenolpyruvate carboxykinase 2 deficiency, Osteoporosis, Hepatic steatosis, Alzheimer's disease, Renal hypoplasia, Acute monocytic leukemia	https://www.targetvalidation.org/target/ENSG00000075413/associations	STK11 PRKCZ PRKCQ PRKCI PRKACA PIM1 YWHAG PRKACA SFN MAP4 CDC25C DYNLL1 TCEA2 PPP2R5D CHEK2 HDAC7 YWHAB TTR PPP1R2 FANCF CPSF4 ASB13 PNPLA2 PMPCB VPS11 ATP6V0C PKP2 CHGB NOL11 ANKRD7	Cell membrane	Yes	https://www.bindingdb.org/uniprot/P27448	✓
MARK4	HUMAN MAP/microtubule affinity-regulating kinase 4	KIAA1860, MARKL1	Q96L34	ENSG0000(	Kinase,Cancer,Cancer-Antigen	AX305105	Lung carcinoma, Glioma, Glioblastoma, Amyloidosis, Brain neoplasm, Peutz-Jeghers syndrome, Low density lipoprotein cholesterol measurement, C-reactive protein measurement, Ischemia, Fatal familial insomnia	https://www.targetvalidation.org/target/ENSG00000007047/associations	ODF2 STK11 PRKCI PRKCG TUBB MAP2 YWHAZ MAP4 CDC42 MAPT HDAC7 YWHAH TUBB2A YWHAB TUBA4A PARD6A TUBG1 PNMA1 HPS6	Cytoplasm, cytoskeleton, microtubule organizing center, centrosome	Yes	https://www.bindingdb.org/uniprot/Q96L34	✓
MAST1	HUMAN Microtubule-associated serine/threonine-protein kinase 1	KIAA0973, SAST	Q9Y2H9	ENSG0000(	Kinase	BC027985	Prostate carcinoma, Astrocytoma, Ependymoma, Glioblastoma multiforme, Li-Fraumeni syndrome, Breast carcinoma, Acute lymphoblastic leukemia	https://www.targetvalidation.org/target/ENSG00000105613/associations	PTEN SNTA1 MTA1 GRK5 SNTB2	Cell membrane	Yes	https://www.bindingdb.org/uniprot/Q9Y2H9	✓
MASTL	HUMAN Serine/threonine-protein kinase greatwall	FLJ14813, GW, Gwl, THC2	Q96GX5	ENSG0000(	Kinase	AL160291	Hereditary thrombocytopenia with normal platelets, Autosomal thrombocytopenia with normal platelets, Neuroblastoma, Gray platelet syndrome, Autosomal dominant disorder, Asthma, Melanoma, Glioblastoma multiforme, Colorectal cancer, Hepatitis C infection	https://www.targetvalidation.org/target/ENSG00000120539/associations		Cytoplasm, cytoskeleton, microtubule organizing center, centrosome, Nucleus, Cleavage furrow,	No		✓

MATK	HUMAN Megakaryocyte-associated tyrosine-protein kinase	CHK, CTK, DKFZp434N1212, HHYLTK, HYL, HYLTK, Lsk, MGC1708, MGC2101	P42679	ENSG0000(	Kinase,Cancer	AK055395	Colorectal adenocarcinoma, Astrocytoma, Medulloblastoma, Brain neoplasm, Neuroblastoma, Hyperactive behavior, Colonic neoplasm, Breast carcinoma, Lung carcinoma, Ovarian carcinoma	https://www.targetvalidation.org/target/ENSG00000007264/associations	PTK2B SRC KIT LYN NTRK1 ERBB2 EWSR1 NTRK1 LYN PXN KIT FGR CD36 PTK2B SIRPA	Cytoplasm	Yes	https://www.bindingdb.org/uniprot/P42679	✓
MATN3	HUMAN Matrilin-3	EDM5, HOA	O15232	ENSG0000(	Others	CH471053	Multiple epiphyseal dysplasia, Multiple epiphyseal dysplasia type 5, Osteoarthritis, Degenerative polyarthritis, Spondyloepimetaphyseal disorder, Autosomal recessive predisposition, Platyspondyly, Carpal delayed ossification, Small capital femoral epiphyses, Metaphyseal irregularity	https://www.targetvalidation.org/target/ENSG00000132031/associations	COMP	Secreted	No		✓
MAX	HUMAN Protein max	bHLHd4	P61244	ENSG0000(	Transcription factor,Signalling,Cancer	X66867	Adrenal gland pheochromocytoma, Hereditary pheochromocytoma-paraganglioma, Inherited cancer-predisposing syndrome, Lung carcinoma, Pheochromocytoma, Paraganglioma, Metabolic syndrome X, Neuroblastoma, Colorectal cancer, Non-small cell lung carcinoma	https://www.targetvalidation.org/target/ENSG00000125952/associations	TRRAP MGA MAPK14 TFDP1 SMAD4 MXD1 SMAD3 CSNK2A1 CASP7 CASP5 MAPK1 TEAD1 CSNK2A2 E2F6 SPAG9 MAD1L1 EP300 CEBPA MNT MYC FTH1 MXD1 MXD3 MGA MXD4 MXI1	Nucleus, Cell projection, dendrite	Yes	https://www.bindingdb.org/uniprot/P61244	✓
MAZ	HUMAN Myc-associated zinc finger protein	Pur-1, ZF87, Zif87, ZNF801	P56270	ENSG0000(	Transcription factor	U33819	Multiple sclerosis, Epithelial neoplasm, Breast carcinoma, HIV infection, Degenerative polyarthritis, Cerebellar disease, Autistic disorder, Breast carcinoma, Amyloidosis, Inflammation	https://www.targetvalidation.org/target/ENSG00000103495/associations	CSNK2A1 DCC MAPK3 CSNK2A2 BPTF	Nucleus	No		✓
MCM5	HUMAN DNA replication licensing factor MCM5	CDC46	P33992	ENSG0000(	Signalling, Cancer	AY212028	Lung carcinoma, Large cell lung carcinoma, Melanoma, Chronic alcoholic intoxification, Adenocarcinoma, Dermatologic disorder, Colonic neoplasm, Chronic lymphocytic leukemia, Bladder carcinoma, Breast neoplasm	https://www.targetvalidation.org/target/ENSG00000100297/associations	ORC6 ORC3 CDC6 MCM6 ORC2 ORC1 MCM2 MCM7 MCM4 MCM3 RPA2 MYCN CDC45 ORC5 CHEK1 CDC7 MCM7 MCM6 CDC45 ORC2 ORC1 ORC5	Nucleus,	No		✓
MCMBP	HUMAN Mini-chromosome maintenance complex -binding protein	C10orf119, MCM-BP	Q9BTE3	ENSG0000(	Cancer,Cancer-Antigen	AC027672	Melanoma, Pancreatic carcinoma, Azoospermia, Atopic eczema, Glioblatoma multiforme, Colorectal neoplasm, Psoriasis, Cystic fibrosis, Coloretal cancer, Sjogren's syndrome	https://www.targetvalidation.org/target/ENSG00000197771/associations		Nucleus	No		✓
MDM2	HUMAN E3 ubiquitin-protein ligase Mdm2	HDM2	Q00987	ENSG0000(	Cancer	U33200	Adenocarcinoma, Squamous cell carcinoma, Mammary neoplasm, Breast carcinoma, Li-Fraumeni syndrome, Melanoma, Osteosarcoma, Lymphoma, Prostatic neoplasm, Dedifferentiated liposarcoma	https://www.targetvalidation.org/target/ENSG00000135679/associations	ALK TBK1 DAXX STK17A RASSF1 PAK6 UBE2Z TP63 HIPK2 TSG101 SRPK1 UBE2E2 MTBP USP7 COPS5 DDX17 KAT2B DYRK2 RASSF5 NEK9 CDKN2A PNCK TNNI3K HIF1A ELAVL1 RPS6KA2 ERBB4 STK38 PDK1 HDAC1	Nucleus, nucleoplasm, Cytoplasm, nucleolus,	Yes	https://www.bindingdb.org/uniprot/Q00987	✓
ME2	HUMAN NAD-dependent malic enzyme, mitochondrial	MAOM, NAD-ME	P23368	ENSG0000(	Cancer,Cancer-Antigen	AC015864	Melanoma, Breast carcinoma, Adenocarcinoma, Idiopathic generalized epilepsy, Bipolar disorder, Nonorganic psychosis, Manic, Schizophrenia, Psychotic disorder, Opioid-related disorder	https://www.targetvalidation.org/target/ENSG00000082212/associations		Mitochondrion matrix,	Yes	https://www.drugbank.ca/biodb/polypeptides/P23368	✓
MECP2	HUMAN Methyl-CpG-binding protein 2	MRX16, MRX79, RTT	P51608	ENSG0000(	Transcription factor,Cancer	AF158180	X-linked syndromic intellectual disability, Severe neonatal-onset encephalopathy with microcephaly, Rett syndrome, X-linked intellectual disability-psychosis-macroorchidism, Seizures, Myoclonus, Liver carcinoma, Autistic disorder, Intellectual disability	https://www.targetvalidation.org/target/ENSG00000169057/associations	HIPK2 SMARCA2 ATRX CBX5 YY1 SP1 CDKL5 RBPJ SIN3A SMARCB1 YBX1 PRMT6 LBR	Nucleus,	Yes	https://www.bindingdb.org/uniprot/P51608	✓

MED16	HUMAN Mediator of RNA polymerase II transcription subunit 16	DRIP92, THRAP5, TRAP95	Q9Y2X0	ENSG0000(	Transcription factor	BC007853	Ovarian carcinoma, Colorectal cancer, Melanoma, Breast carcinoma, Endometriosis, Schwannomatosis, Atopic eczema, Rheumatoid arthritis, Spina bifida, Hereditary spastic paraplegia	https://www.targetvalidation.org/target/ENSG00000175221/associations		Nucleus	No	✓
MED17	HUMAN Mediator of RNA polymerase II transcription subunit 17	CRSP6, CRSP77, DRIP80, TRAP80	Q9NVC6	ENSG0000(	Transcription factor,Cancer	AK022156	Infantile cerebral and cerebellar atrophy with postnatal progressive microcephaly, Central nervous system cancer, Glioblastoma multiforme, Lung carcinoma, Multiple myeloma, Non-small cell lung carcinoma, Melanoma, Clonus, Diffuse cerebral atrophy, Hypsarrhythmia, Postnatal microcephaly, Mental and motor retardation	https://www.targetvalidation.org/target/ENSG00000042429/associations		Nucleus	No	✓
MED21	HUMAN Mediator of RNA polymerase II transcription subunit 21	SRB7, SURB7	Q13503	ENSG0000(	Transcription factor	AK311838	Melanoma, Atopic eczema, Breast carcinoma, Small cell lung carcinoma, Prostate carcinoma, Non-seminomatous lesion, Acute myeloid leukemia, Adenocarcinoma, Psoriasis	https://www.targetvalidation.org/target/ENSG00000152944/associations	SKP1 CDK9 CDK8 MED6 MED14	Nucleus	No	✓
MED22	HUMAN Mediator of RNA polymerase II transcription subunit 22	SURF5	Q15528	ENSG0000(	Others	AJ224358	Brachyolmia, Achondrogenesis, Smith-McCort dysplasia, Autosomal recessive spondylocostal dysostosis, Mucopolysaccharidosis type 4, Kniest dysplasia, Hypochondrogenesis, Breast carcinoma, Lung carcinoma, Diaphanospondylodysostosis, Roifman syndrome	https://www.targetvalidation.org/target/ENSG00000148297/associations		Nucleus	No	✓
MED24	HUMAN Mediator of RNA polymerase II transcription subunit 24	CRSP100, CRSP4, DRIP100, KIAA0130, MED5, THRAP4, TRAP100	O75448	ENSG0000(	Transcription factor	AK022508	Multiple myeloma, X-linked lethal multiple pterygium syndrome, Medeira-Dennis-Donnai syndrome, Congenital heart disease, Asthma, Neuroblastoma, PAGOD syndrome, Acute myeloid leukemia, Hepatocellular carcinoma, Pancreatic ductal adenocarcinoma	https://www.targetvalidation.org/target/ENSG00000008838/associations		Nucleus	No	✓
MED30	HUMAN Mediator of RNA polymerase II transcription subunit 30	THRAP6, TRAP25	Q96HR3	ENSG0000(	Transcription factor	AC087361	Cardiomyopathy, Familial dilated cardiomyopathy, Familial isolated dilated cardiomyopathy, Congenital dyserythropoietic anemia, Gastric neoplasm, Shwachman-Diamond syndrome, Hyaline body myopathy, Barth syndrome, Atopic eczema, Histiocytoid cardiomyopathy	https://www.targetvalidation.org/target/ENSG00000164758/associations		Nucleus	No	✓
MED4	HUMAN Mediator of RNA polymerase II transcription subunit 4	DRIP36, HSPC126, TRAP36, VDRIP	Q9NPJ6	ENSG0000(	Transcription factor	AK001934	Glioblastoma multiforme, Breast carcinoma, Retinoblastoma, Medulloblastoma, Endometriosis, Ovarian carcinoma, Uterine fibroid, Cystic fibrosis, Dermatomyositis, Meningococcal infection	https://www.targetvalidation.org/target/ENSG00000136146/associations	MED18 MED1	Nucleus,	No	✓
MEF2A	HUMAN Myocyte-specific enhancer factor 2A	RSRFC4, RSRFC9	Q02078	ENSG0000(	Transcription factor,Signalling,Cancer	X68505	Cardiomyopathy, Nonorganic psychosis, Psychotic disorder, Myocardial infarction, Liver carcinoma, Alzheimer's disease, Diaphragmatic hernia, Coronary arteriosclerosis, Melanoma, Glioblastoma multiforme	https://www.targetvalidation.org/target/ENSG00000068305/associations	HDAC5 HDAC9 NLK SLC2A4RG ERBIN MAPK14 SMAD2 MEF2D SMAD4 NFATC2 MAPK7 EP300 CDK5 CSNK2A1 HDAC4 MAPK10 ASCL1 MAPK9 CASP3 MYOG CDK4 HAND1 SLC2A4 MYOD1 HDAC5 HDAC9	Nucleus	No	✓

MEF2C	HUMAN Myocyte-specific enhancer factor 2C	MEF2C	Q06413	ENSG0000(	Transcription factor,Signalling	AL833268	5q14.3 microdeletion syndrome, Self-reported educational attainment, Epilepsy, Stereotypic movement disorder, Autism spectrum disorder, Intellectual disability, Seizures, Upward slant of palpebral fissure, Hypoplasia of corpus callosum, Gastric carcinoma	https://www.targetvalidation.org/target/ENSG00000081189/associations	GRIP1<br>HDAC5<br>NLK<br>MYLK2<br>KAT2B<br>CREBBP<br>MAML1<br>MAPK15<br>MAPK6<br>MAPK14<br>SMAD2<br>RPS6KA2<br>MAPK7<br>EP300<br>CDK5<br>CSNK2A1<br>HDAC4<br>MAPK10<br>RPS6KA3<br>NOTCH1<br>MAPK9<br>MAPK8<br>GATA4<br>SOX18<br>AKT1<br>MAPK4<br>MAPK1<br>MAPK3<br>CSNK2A2	Nucleus,	No		✓
MEIS2	HUMAN Homeobox protein Meis2	MRG1	O14770	ENSG0000(	Transcription factor	AF179898	Cleft palate, Uranostaphyloschisis, Intellectual disability, Narrow forehead, Decreased width of the skull, Smooth philtrum, Mental deficiency, Pointed chin, Lymphoma, Tongue squamous cell carcinoma	https://www.targetvalidation.org/target/ENSG00000134138/associations	PAX6<br>PBX1	Nucleus	No		✓
MEIS3	HUMAN Homeobox protein Meis3	MRG2	Q99687	ENSG0000(	Transcription factor	CH471126	Tongue squamous cell carcinoma, Colorectal adenocarcinoma, Diffuse large B-cell lymphoma, Glioblastoma multiforme	https://www.targetvalidation.org/target/ENSG00000105419/associations		Nucleus	No		✓
MEOX2	HUMAN Homeobox protein MOX-2	GAX, MOX2	P50222	ENSG0000(	Transcription factor	AC005550	Renal cell carcinoma, Ovarian carcinoma, Myocardial ischemia, Alzheimer's disease, Amyloidosis, Meningomyelocele, Lung neoplasm, Oculopharyngodistal myopathy, Melanoma, Hyaline body myopathy	https://www.targetvalidation.org/target/ENSG00000106511/associations	RNF10	Nucleus	No		✓
METTL3	HUMAN N6-adenosine-methyltransferase 70 kDa subunit	MTA70	Q86U44	ENSG0000(	Cancer,Cancer-Antigen	BX247964	Lung carcinoma, Large cell lung carcinoma, Non-small cell lung carcinoma, Unipolar depression, Major depressive disorder, Melanoma, Breast carcinoma, Psoriasis, Dermatomyositis, Pancreatic carcinoma	https://www.targetvalidation.org/target/ENSG00000165819/associations	DYRK2<br>NEK3<br>WEE1<br>METTL14	Nucleus	No		✓
MIF	HUMAN Macrophage migration inhibitory factor	GIF, GLIF	P14174	ENSG0000(	Signalling, Cancer, Cytokine/Chemokine	L19686	Arthritis, Rheumatoid arthritis, Autoimmune disease, Sepsis, Arthralgia, Inflammation, Prostatic neoplasm, Mammary neoplasm, Autistic disorder, Dermatofibrosarcoma protuberans	https://www.targetvalidation.org/target/ENSG00000240972/associations	COPS5<br>CD74	Secreted	Yes	https://www.bindingdb.org/uniprot/P14174	✓
MITF	HUMAN Microphthalmia-associated transcription factor	bHLHe32	O75030	ENSG0000(	Transcription factor,Cancer	GU355676	Waardenburg syndrome, Tietz syndrome, Waardenburg syndrome type 2, Melanoma, Renal cell carcinoma, Hypopigmentation disorder, Melanocytic nevus, Ocular albinism, Cutaneous melanoma, Kidney neoplasm	https://www.targetvalidation.org/target/ENSG00000187098/associations	PIAS3<br>LEF1<br>CREBBP<br>MAPK14<br>RPS6KA1<br>EP300<br>UBE2I<br>GSK3B<br>GSK3A<br>MAPK8<br>CTNNB1<br>MAPK1<br>MAPK3<br>CDK2<br>TFEC<br>PSMD14<br>OTX2<br>YWHAZ<br>LEF1<br>PAX6<br>PATZ1<br>TFE3	Nucleus	No		✓
MKNK1	HUMAN MAP kinase-interacting serine/threonine-protein kinase 1	MNK1	Q9BUB5	ENSG0000(	Kinase	AL136373	Schizophrenia, Septic shock, Squamous cell lung carcinoma, Colorectal adenocarcinoma, Mucocutaneous lymph node syndrome, Rheumatoid arthritis, Glioblastoma multiforme, Breast carcinoma, Multiple myeloma, Chronic interstitial cystitis	https://www.targetvalidation.org/target/ENSG00000079277/associations	MAPK14<br>PAK2<br>EIF4G1<br>EIF4G2<br>PPP2CA<br>MAPK1<br>MAPK3<br>EIF4E<br>EIF4G3<br>MAPK1<br>EIF4G1<br>MAPK14<br>SFPQ<br>EIF4E<br>EIF4B<br>PAK2<br>EIF4G2<br>MAPK3<br>HNRNPA1<br>PLA2G4A<br>SPRY2<br>MBP<br>PNPLA8	Cytoplasm, Cytoplasm, Nucleus,	Yes	https://www.bindingdb.org/uniprot/Q9BUB5	✓
MKNK2	HUMAN MAP kinase-interacting serine/threonine-protein kinase 2	GPRK7, MNK2	Q9HBH9	ENSG0000(	Kinase,Cancer,Cancer-Antigen	BC073140	Lung carcinoma, Acute lymphoblastic leukemia, Hypertriglyceridemia, Breast carcinoma, T-cell acute lymphoblastic leukemia, Ulcerative colitis, Medulloblastoma, Diffuse large B-Cell lymphoma, Pancolitis, Pancreatic ductal adenocarcinoma	https://www.targetvalidation.org/target/ENSG00000099875/associations	MAPK14<br>VHL<br>MAPK1<br>MAPK3<br>MAPK1<br>EIF4G1<br>MAPK14<br>TRIM28<br>SFPQ<br>EIF4E<br>ESR1<br>CCR5<br>TGFBR1<br>ESR2<br>ADRB2<br>HNRNPA1<br>SPRY2<br>PLEC<br>MC4R<br>CHRM2	Nucleus, PML body, Cytoplasm,	Yes	https://www.bindingdb.org/uniprot/Q9HBH9	✓

MLANA	HUMAN Melanoma antigen recognized by T-cells 1	MART1	Q16655	ENSG0000(	Others	AK312149	Autoimmune disease, Multiple sclerosis, melanoma, Peritoneal neoplasm, Malignant mesothelioma, Contact dermatitis, Angiomyolipoma, Nevus, Sarcoma, Actinic keratosis	https://www.targetvalidation.org/target/ENSG00000120215/associations	GPR143	Endoplasmic reticulum membrane, Single-pass type III membrane protein, Golgi apparatus, Golgi apparatus, trans-Golgi network membrane, Melanosome,	Transmembrane	No		✓
MLF1	HUMAN Myeloid leukemia factor 1	MLF1	P58340	ENSG0000(	Cancer	AK056948	Lung carcinoma, Prostate carcinoma, Breast carcinoma, Melanoma, Brain ischemia, Leukemogenesis, Acute promyelocytic leukemia, Coronary heart disease, Gastric carcinoma, Colorectal carcinoma	https://www.targetvalidation.org/target/ENSG00000178053/associations	NRBP1 YWHAE COPS3 CENPU	Cytoplasm		No		✓
MLH1	HUMAN DNA mismatch repair protein Mlh1	COCA2, FCC2, HNPCC, HNPCC2	P40692	ENSG0000(	Cancer	U17857	Colonic neoplasm, Adenocarcinoma, Turcot syndrome, Hereditary non-polyposis colon cancer type 2, Torre -Muir syndrome, Colorectal neoplasm, Inherited cancer-predisposing syndrome, Prostate carcinoma, Lynch syndrome, Lung carcinoma	https://www.targetvalidation.org/target/ENSG00000076242/associations	BRIP1 ATR CASP3 TP53 MSH4 BLM PMS1 PMS2 POLH ATR EXO1 RAD9A MBD4 MLH3 TMSB4X	Nucleus		No		✓
MLKL	HUMAN Mixed lineage kinase domain-like protein	hMLKL	Q8NB16	ENSG0000(	Kinase	NA	Lung carcinoma, Breast carcinoma, Asthma, Amyloidosis, Gastric carcinoma, Melanoma, Laryngeal disease, Acute kidney failure, Inflammatory bowel disease, Fibrosarcoma	https://www.targetvalidation.org/target/ENSG00000168404/associations	RIPK3 IL15 PBX2	Cytoplasm		No		✓
MLLT11	HUMAN Protein AF1q	AF1Q	Q13015	ENSG0000(	Cancer	AL590133	Adenocarcinoma, Melanoma, Juvenile myelomonocytic leukemia, Breast Carcinoma, Acute myelocytic leukemia, Leukemogenesis, Mixed lineage leukemia, Myelodysplastic syndrome, Bladder transitional cell carcinoma, Prostate adenocarcinoma	https://www.targetvalidation.org/target/ENSG00000213190/associations		Nucleus		No		✓
MLLT3	HUMAN Protein AF-9	AF-9, AF9, YEATS3	P42568	ENSG0000(	Transcription factor,Cancer	AL512635, AL354879, AL513498	Adenocarcinoma, Colorectal adenocarcinoma, Bipolar disorder, Diabetic nephropathy, Acute myelocytic leukemia, Precursor cell lymphoblastic leukemia lymphoma, Lung carcinoma, Mixed lineage leukemia, melanoma, Head and Neck squamous cell carcinoma	https://www.targetvalidation.org/target/ENSG00000171843/associations	HSP90B1 TRIM24 RNF2 DOT1L BCOR AFF1 CBX8	Nucleus		No		✓
MLLT6	HUMAN Protein AF-17	AF17	P55198	ENSG0000(	Transcription factor,Cancer	AB209633	Leukemia, Acute Leukemia, Acute myeloid leukemia, Acute monocytic leukemia, Spastic syndrome, Colorectal cancer, Precursor cell lymphoblastic leukemia lymphoma, Multiple sclerosis, Myelodysplastic syndrome, Meningococcal infection	https://www.targetvalidation.org/target/ENSG00000275023/associations	DOT1L	Nucleus		No		✓
MLPH	HUMAN Melanophilin	exophilin-3, Slac-2a	Q9BV36	ENSG0000(	Cancer,Cancer-Antigen	AK225381	Griscelli disease, Griscelli disease type 3, Prostate carcinoma, Breast carcinoma, Hypopigmentation disorder, Autistic disorder, Hypopigmentation-immunodeficiency disease, Piebaldism, Hyperactive behavior, Prostate carcinoma	https://www.targetvalidation.org/target/ENSG00000115648/associations	RAB27A MYO5A	Cytoplasm,		No		✓
MLX	HUMAN Max-like protein X	bHLHd13, TCFL4	Q9UH92	ENSG0000(	Transcription factor	AF213668	Melanoma, Acute monocytic leukemia, Ovarian carcinoma, Hemorrhage, Breast carcinoma, Takayasu arteritis, Vasculitis, Migraine disorder, Myalgia, Subcutaneous nodule	https://www.targetvalidation.org/target/ENSG00000108788/associations	MLXIPL MLXIP	Cytoplasm		Yes		✓
													https://www.bindingdb.org/uniprot/Q9UH92	

MMP11	HUMAN Stromelysin-3	STMY3	P24347	ENSG0000(	Cancer	BC057788	Squamous cell carcinoma, Esophageal squamous cell carcinoma, Non-small cell lung carcinoma, Spontaneous abortion, Breast carcinoma, Colorectal neoplasm, Meningioma, Thyroid neoplasm, Gastric neoplasm, Adenocarcinoma	https://www.targetvalidation.org/target/ENSG00000099953/associations	SERPINA1	Secreted, extracellular space, extracellular matrix		Yes	https://www.bindingdb.org/uniprot/P24347	✓
MMP14	HUMAN Matrix metalloproteinase-14	MT1-MMP	P50281	ENSG0000(	Cancer	U41078	Multicentric osteolysis-nodulosis-arthropathy spectrum, Torg-Winchester syndrome, Nodulosis-arthropathy-osteolysis syndrome, Mammary neoplasm, Chronic obstructive pulmonary disease, Adenocarcinoma, Esophageal neoplasm, Arthropathy, Corneal opacity, Winchester syndrome	https://www.targetvalidation.org/target/ENSG00000157227/associations	CAV1 GNRHR ENG ACAN TIMP2 CCL2 SRC FURIN MMP2 ITGAV PRKCG ACTB LIMK1 MMP2 C1QBP SDC1 MMP13 CCL7 KISS1 TIMP4 LUM ADI1	Membrane Melanosome, Cytoplasm,	Transmembrane	Yes	https://www.bindingdb.org/uniprot/P50281	✓
MMP2	HUMAN 72 kDa type IV collagenase	CLG4, CLG4A, TBE-1	P08253	ENSG0000(	Signalling, Cancer	AK312711	Multicentric osteolysis-nodulosis-arthropathy spectrum, Torg-Winchester syndrome, Nodulosis-arthropathy-osteolysis syndrome, Myocardial infarction, Liver cirrhosis, Rheumatoid arthritis, Hypertensive disease, Glioblastoma, Colorectal neoplasm, Bone disease, Prostate carcinoma	https://www.targetvalidation.org/target/ENSG00000087245/associations	FOXM1 GNAI1 MMP14 TIMP3 PRKCA TIMP2 ATF2 FGFR1 AR THBS1 HSP90AA1 ITGAV TP53 FN1 COL1A1 IL1B TGFB1 TIMP1 CLDN1 FGFR1 EGFR BACE1 CXCL12 TGFB1 MMP14 CCL7 KISS1 COL7A1 PCSK9 LCN2 COL6A3	Secreted, extracellular space, extracellular matrix, Membrane, Nucleus,		Yes	https://www.bindingdb.org/uniprot/P08253	✓
MMP24	HUMAN Matrix metalloproteinase-24	MT5-MMP	Q9Y5R2	ENSG0000(	Others	NA	Myoclonus-dystonia syndrome, Prostate carcinoma, Atopic eczema, Liver cirrhosis, Chronic Hepatitis C, Thermal hyperalgesia, Impaired cognition, Kidney failure, Multiple sclerosis, Alzheimer's disease	https://www.targetvalidation.org/target/ENSG00000125966/associations	KISS1	Matrix metalloproteinase-24, Cell membrane	Transmembrane	Yes	https://www.bindingdb.org/uniprot/Q9Y5R2	✓
MNAT1	HUMAN CDK-activating kinase assembly factor MAT1	MAT1, RNF66	P51948	ENSG0000(	Kinase,Transcription factor	AY165512	HIV infection, Ovarian carcinoma, Lung neoplasm, Multiples sclerosis, Alzheimer's disease, Neurodegenerative disorder, Malaria, Systemic lupus erythematosus, Neuroblastoma, Huntington disease	https://www.targetvalidation.org/target/ENSG00000020426/associations	TSC1 CCNH CDK7 GTF2H1 POLR2A ERCC3 ERCC2 RB1 TRIM24 CCNH CDK7 POLR2A TSC1 ESR1 ERCC2 ERCC3 GTF2H1 GTF2E2	Nucleus,		Yes	https://www.bindingdb.org/uniprot/P51948	✓
MNDA	HUMAN Myeloid cell nuclear differentiation antigen	PYHIN3	P41218	ENSG0000(	Transcription factor	NA	Ovarian carcinoma, Lung carcinoma, inflammatory bowel disease, Crohn's disease, Atherosclerosis, Promyelocytic leukemia, Monocytoid B-cell lymphoma, Situs ambiguus, Neuropathy, Systemic lupus erythematosus	https://www.targetvalidation.org/target/ENSG00000163563/associations	CDK2 CDK1	Nucleus, Cytoplasm,		No		✓
MOB3A	HUMAN MOB kinase activator 3A	MOB-LAK, MOBKL2A, moblak	Q96BX8	ENSG0000(	Kinase,Cancer,Cancer-Antigen	AC005328	Glioblastoma multiforme, Colorectal cancer, Breast carcinoma, Colorectal adenocarcinoma, Pancreatic carcinoma, Mantle cell lymphoma, Acute myeloid leukemia, Chronic lymphocytic leukemia, Colorectal adenocarcinoma	https://www.targetvalidation.org/target/ENSG00000172081/associations				No		✓
MOK	HUMAN MAPK/MAK/MRK overlapping kinase	RAGE, RAGE1	Q9UQ07	ENSG0000(	Kinase	U46193	Malignant Mesothelioma, Colonic neoplasm, Colorectal adenocarcinoma, Diabetic retinopathy, Coronary artery disease, Type I diabetes mellitus, Diabetic nephropathy, Type II diabetes mellitus, Bladder carcinoma, Cystic fibrosis	https://www.targetvalidation.org/target/ENSG00000080823/associations	S100P PRKCA	Cytoplasm		No		✓

MORC1	HUMAN MORC family CW-type zinc finger protein 1	CT33, MORC, ZCW6	Q86VD1	ENSG0000(	Others	AC112138	Breast carcinoma, Azoospermia, Li-Fraumeni syndrome, Major depressive disorder, Unipolar depression, B-Cell lymphoma, Infertility, Glioblastoma multiforme, Mood disorder, Macroglobulinemia, Colorectal adenocarcinoma	https://www.targetvalidation.org/target/ENSG00000114487/associations		Nucleus		No	✓
MPP1	HUMAN 55 kDa erythrocyte membrane protein	DXS552E, EMP55	Q00013	ENSG0000(	Kinase	AY423731	Breast carcinoma, Malignant mesothelioma, Chronic granulomatous disease, Adenocarcinoma, Rheumatoid arthritis, Chronic Hepatitis C, Acute myeloid leukemia, Van der Woude syndrome, Sarcoidosis, Mycoplasma infection	https://www.targetvalidation.org/target/ENSG00000130830/associations	ERBIN GYPC	Membrane, Lipid-anchor, Cell projection, stereocilium		No	✓
MPP3	HUMAN MAGUK p55 subfamily member 3	DLG3	Q13368	ENSG0000(	Kinase	AK313045	Hepatocellular carcinoma, Retinal dystrophy, Colorectal adenocarcinoma, Melanoma, Lung adenocarcinoma, Retinopathy, Late-onset retinal degeneration, Astrocytoma, Lung carcinoma, Triglyceride measurement	https://www.targetvalidation.org/target/ENSG00000161647/associations	PLK2 ERBIN CADM1			No	✓
MPP5	HUMAN MAGUK p55 subfamily member 5	FLJ12615, PALS1	Q8N3R9	ENSG0000(	Kinase	BC053366	Melanoma, Osteosarcoma, Adenocarcinoma, Lung carcinoma, Colorectal adenocarcinoma, Leber congenital amaurosis, Overgrowth, Breast carcinoma, Metastatic prostate cancer, Down syndrome	https://www.targetvalidation.org/target/ENSG00000072415/associations	LIN7C PATJ PARD6B CRB1 CRB3 MPP4	Cell membrane, Peripheral membrane protein, Endomembrane system, Peripheral membrane protein, Cell junction, tight junction,		No	✓
MPZL2	HUMAN Myelin protein zero-like protein 2	EVA, EVA1	O60487	ENSG0000(	Others	AY359061	Breast carcinoma, Metastatic prostate cancer, Lung carcinoma, Pendred's syndrome, Paraganglioma, Congenital ear anomaly NOS, Dementia, Brain infarction, Impaired cognition, Pheochromocytoma	https://www.targetvalidation.org/target/ENSG00000149573/associations		Membrane	Transmembrane	No	✓
MRPL45	HUMAN 39S ribosomal protein L45, mitochondrial	RM45	Q9BRJ2	ENSG0000(	Cancer,Cancer-Antigen	BC130384	Lung carcinoma, Atopy	https://www.targetvalidation.org/target/ENSG00000278845/associations		Mitochondrion,		No	✓
MRPL55	HUMAN 39S ribosomal protein L55, mitochondrial	RM55, UNQ5835/PRO19675	Q7Z7F7	ENSG0000(	Others	AL136379	Hereditary spastic paraplegia, Glioblastoma multiforme	https://www.targetvalidation.org/target/ENSG00000162910/associations		Mitochondrion		No	✓
MRPS26	HUMAN 28S ribosomal protein S26, mitochondrial	C20orf193, MRP-S13, MRP-S26, RPMS13	Q9BYN8	ENSG0000(	Cancer,Cancer-Antigen	NA	Medulloblastoma, Adenocarcinoma	https://www.targetvalidation.org/target/ENSG00000125901/associations		Mitochondrion		No	✓
MRPS30	HUMAN 28S ribosomal protein S30, mitochondrial	PDCD9, BM047	Q9NP92	ENSG0000(	Others	AL136706	Breast carcinoma, Schizophrenia, Cystic fibrosis, Acute pyelonephritis, Liver carcinoma, HIV infection, MRSA, Melanoma, Glioblastoma multiforme, Chronic kidney disease	https://www.targetvalidation.org/target/ENSG00000112996/associations		Mitochondrion		No	✓
MRT4	HUMAN mRNA turnover protein 4 homolog	C1orf33, dJ657E11.4, MRT4	Q9UKD2	ENSG0000(	Cancer,Cancer-Antigen	AK027569	Breast carcinoma, Melanoma, Squamous cell carcinoma, Medulloblastoma, Adenocarcinoma, Ovarian carcinoma, Pancreatic carcinoma, Vulvar intraepithelial neoplasia, Multiple myeloma, Atopic eczema	https://www.targetvalidation.org/target/ENSG00000053372/associations	POLR3G	Nucleus, nucleolus		No	✓
MSC	HUMAN Musculin	ABF-1, bHLHa22	O60682	ENSG0000(	Transcription factor,Cancer,Cancer-Antigen	BC006313	Prostate carcinoma, Autism, Late-onset myasthenia gravis, Follicular lymphoma, B-Cell lymphoma, Burkitt lymphoma, Glioma, Multiple myeloma, Alzheimer's disease, Breast carcinoma	https://www.targetvalidation.org/target/ENSG00000178860/associations	ID2 TCF3	Nucleus,		No	✓

MSH2	HUMAN DNA mismatch repair protein Msh2	COCA1, HNPCC, HNPCC1	P43246	ENSG0000(	Cancer	L47583	Colonic neoplasm, Inherited cancer-predisposing syndrome, Lynch syndrome, Torre-Muir syndrome, Turcot syndrome, Colorectal cancer, Lymphoma, Gastric neoplasm, Hereditary nonpolyposis colorectal cancer, Non-polyposis Turcot syndrome	https://www.targetvalidation.org/target/ENSG00000095002/associations	ATR HMGB1 TP53 CHEK2 BLM MUTYH POLK ATR EXO1 MSH3 SLX4 MSH6	Nucleus		No		✓
MSLN	HUMAN Mesothelin	MPF	Q13421	ENSG0000(	Cancer	AE006464	Epithelial neoplasm, Mesothelioma, Adenocarcinoma, Ovarian neoplasm, Pancreatic neoplasm, Cholangiocarcinoma, Pleural disease, Lymphatic metastasis, Endometroid carcinoma, Lung adenocarcinoma	https://www.targetvalidation.org/target/ENSG00000102854/associations	MUC16	Cell membrane, Lipid-anchor, GPI-anchor, Golgi apparatus, Megakaryocyte-potentiating factor, Secreted,		No		✓
MSN	HUMAN Moesin	MSN	P26038	ENSG0000(	Cancer	NA	Adenocarcinoma, Colonic neoplasm, Degenerative polyarthritis, Myocardial ischemia, Juvenile arthritis, Neoplasm metastasis, Aplastic anemia, Pancreatic neoplasm, Mammary neoplasm, Squamous cell carcinoma	https://www.targetvalidation.org/target/ENSG00000147065/associations	LRRK2 ROCK1 PRKCQ PRKX SYK MAP3K8 PPM1A GRK2 PRKACA IL7R CD44 EZR NCF1 SRC ICAM1 ISG15 STK10 PPM1B ROCK2 PRKY EZR PRKCQ ISG15 IL2 VCAM1 ARHGDIA SYK SELPLG ICAM3 CD93	Cell membrane		No		✓
MTERF1	HUMAN Transcription termination factor 1, mitochondrial	MTERF	Q99551	ENSG0000(	Transcription factor	CH236949	Breast carcinoma, Ovarian carcinoma, MELAS Syndrome, Epithelial ovarian cancer, Mitochondrial Disease, Serum IgG glycosylation measurement, Low density lipoprotein cholesterol measurement, Nicotine dependence	https://www.targetvalidation.org/target/ENSG00000127989/associations		Mitochondrion,		No		✓
MTMR2	HUMAN Myotubularin-related protein 2	CMT4B, KIAA1073	Q13614	ENSG0000(	Others	AP001877	Charcot-Marie-Tooth disease, Hereditary motor and sensory neuropathy, Charcot-Marie-Tooth disease type 4, Charcot-Marie-Tooth disease type 4B1, Facial Paresis, Distal muscle weakness, Distal amyotrophy, Proximal neurogenic muscle weakness, Abnormal auditory evoked potential, Acquired scoliosis	https://www.targetvalidation.org/target/ENSG00000087053/associations	MAPK1 NEFL SBF1 SBF2	Cytoplasm		No		✓
MTO1	HUMAN Protein MTO1 homolog, mitochondrial	CGI-02	Q9Y2Z2	ENSG0000(	Cancer,Cancer-Antigen	AF319422	Mitochondrial disease, Mitochondrial hypertrophic cardiomyopathy with lactic acidosis due to MTO1 deficiency, Cardiomyopathy, Arrhythmogenic right ventricular dysplasia, Familial dilated cardiomyopathy, Lactic acidosis, EKG abnormalities, Cardiac conduction abnormalities, Hypertrophic cardiomyopathy, Combined oxidative phosphorylation deficiency 10	https://www.targetvalidation.org/target/ENSG00000135297/associations		Mitochondrion		No		✓
MUSK	HUMAN Muscle, skeletal receptor tyrosine-protein kinase	MUSK	O15146	ENSG0000(	Kinase	AL157881, AL513328	Ptosis, Congenital myasthenic syndrome, Fetal akinesia deformation sequence, Li-Fraumeni syndrome, Diabetes mellitus, Pena-Shokeir syndrome type I, Myasthenia gravis, Acquired scoliosis, Intrauterine retardation, Generalized amyotrophy	https://www.targetvalidation.org/target/ENSG00000030304/associations	DOK7 SRC WNT11 WNT9A DVL1 WNT11 RAPSN SYNE1 COLQ	Cell junction, synapse, postsynaptic cell membrane	Transmembrane	Yes		✓
													https://www.bindingdb.org/uniprot/O15146	
MUTYH	HUMAN Adenine DNA glycosylase	MYH, hMYH	Q9UIF7	ENSG0000(	Cancer	AB032922	Inherited cancer-predisposing syndrome, Gastric carcinoma, Pilomatrixoma, Adenomatous polyposis coli, Colonic neoplasm, Autosomal recessive predisposition, Multiple colonic adenomatous polyp, Colorectal cancer, Attenuated familial adenomatous polyposis, Oral squamous cell carcinoma	https://www.targetvalidation.org/target/ENSG00000132781/associations	HUWE1 MSH2 APEX1 ATR TOPBP1 MSH6	Nucleus		No		✓

MX1	HUMAN Interferon-induced GTP-binding protein Mx1	IFI-78K, MxA	P20591	ENSG0000(	Interleukin-Interferon	AK096355	Li-Fraumeni syndrome, Influenza, Acute myeloid leukemia, Fanconi anemia, Myocardial ischemia, Severe acute respiratory syndrome, Chronic Hepatitis C, Hepatitis B, Lung adenocarcinoma, Psoriasis	https://www.targetvalidation.org/target/ENSG00000157601/associations	ISG15 TRPC6 ISG15	Cytoplasm	No		✓
MX2	HUMAN Interferon-induced GTP-binding protein Mx2	MXB	P20592	ENSG0000(	Interleukin-Interferon	AL163285	Melanoma, Li-Fraumeni syndrome, Lung carcinoma, Narcolepsy, Cataplexy, Hepatitis C infection, Lung adenocarcinoma, Central nervous system cancer, Psoriasis, Malignant mesothelioma	https://www.targetvalidation.org/target/ENSG00000183486/associations		Cytoplasm	No		✓
MYBL2	HUMAN Myb-related protein B	B-MYB, BMYB	P10244	ENSG0000(	Transcription factor,Signalling,Cancer	AK223482	Central nervous system cancer, Hepatocellular carcinoma, Acute myeloid leukemia, Myelodysplastic syndrome, Mammary neoplasm, Bladder neoplasm, Breast carcinoma, Neuroblastoma, Chronic obstructive pulmonary disease, Colorectal neoplasm	https://www.targetvalidation.org/target/ENSG00000101057/associations	E2F4 E2F1 CSNK2A1 CDK9 RBL1 CDK2 CCNE1 PARP1 E2F3 EP300 CDK1 CCNB1 CCND1 CREBBP PLA2G4A ZNF622 SND1 BIN2 CCNF	Nucleus,	No		✓
MYC	HUMAN Myc proto-oncogene protein	bHLHe39, c-Myc	P01106	ENSG0000(	Transcription factor,Cancer	X00196,X00198	Lymphoid neoplasm, Neoplasm of mature B-cells, Lymphoma, Burkitt Lymphoma, Liver carcinoma, Colonic neoplasm, Adenocarcinoma, Medulloblastoma, Prostatic Neoplasm, Neuroblastoma	https://www.targetvalidation.org/target/ENSG00000136997/associations	DNMT3A TRRAP PUF60 MYCBP USP28 FUBP1 FBXW7 KAT5 COPS5 KAT2B KAT2A CREBBP HDAC2 DOT1L SIRT6 HUWE1 ATAD2 CDC73 PARP10 SMAD2 ELAVL1 AGER CHD4 HDAC1 PIN1 SKP2 NMI TRIM28 PAK2 MAPK7	Nucleus, nucleoplasm	Yes	https://www.bindingdb.org/uniprot/P01106	✓
MYCBP	HUMAN C-Myc-binding protein	AMY-1	Q99417	ENSG0000(	Transcription factor	AB451287	Breast carcinoma, Glioma, Colorectal adenocarcinoma, Adenoid cystic carcinoma, Obesity, Liver carcinoma, Colon carcinoma, Tongue carcinoma, Hyperamylasemia, Adenocarcinoma	https://www.targetvalidation.org/target/ENSG00000214114/associations	MIR22HG MYC	Cytoplasm, Nucleus, Mitochondrion,	No		✓
MYCBPAP	HUMAN MYCBP-associated protein	AMAP-1	Q8TBZ2	ENSG0000(	Cancer,Cancer-Antigen	AL136765	Azoospermia, Visceral adipose tissue measurement, Li-Fraumeni syndrome, Neoplasm metastasis, Breast carcinoma, Tongue squamous cell carcinoma, Head and Neck squamous cell carcinoma, Renal carcinoma, Teratozoospermia, Inflammation	https://www.targetvalidation.org/target/ENSG00000136449/associations		Cytoplasm	No		✓
MYCL	HUMAN Protein L-Myc	bHLHe38, LMYC, MYCL1	P12524	ENSG0000(	Transcription factor,Cancer	M19720	Adenocarcinoma, Prostate carcinoma, Small cell lung carcinoma, Medulloblastoma, Prostatic neoplasm, Lung neoplasm, Breast neoplasm, Colorectal cancer, Esophagus neoplasm, Gastric neoplasm	https://www.targetvalidation.org/target/ENSG00000116990/associations	GSK3B	Nucleus	No		✓
MYCN	HUMAN N-myc proto-oncogene protein	bHLHe37, MYCNOT, N-myc, NMYC	P04198	ENSG0000(	Transcription factor,Cancer	M13228	Feingold syndrome, Feingold syndrome type 1, Oculodigitoesophagoduodenal syndrome, Neuroblastoma, Medulloblastoma, Basal cell carcinoma, Esophageal atresia, Nervous system neoplasm, Hypoplastic toes, Everted lower lip vermilion	https://www.targetvalidation.org/target/ENSG00000134323/associations	CSNK2A1 MTOR CSNK2A2 SRC AURKA SIRT1 MCM7 MCM5 MCM3 TP53 MCM6 MCM4 ZBTB17 MCM8 MCM2 YAF2	Nucleus,	No		✓

MYD88	HUMAN Myeloid differentiation primary response protein MyD88	MYD88	Q99836	ENSG0000	Signalling, Cancer	BT007376	Primary immunodeficiency, Pyogenic bacterial infections due to MyD88 deficiency, Lymphoid neoplasm, MYD88 Deficiency, Waldenstrom macroglobulinemia, Diffuse large B-Cell lymphoma, Hematologic neoplasm, Pneumonia, Leukemia	https://www.targetvalidation.org/target/ENSG00000172936/associations	IRAK3 TRAF6 TLR6 STAP2 TLR7 IRAK4 TLR8 TLR9 TLR10 PELI1 IRF7 TICAM2 SIGIRR LRRFIP1 IRF4 IRF5 CBLB FADD FLII BTK TIRAP IRAK1 MAL IL1R1 TLR2 TLR5 IRAK2 TNFRSF13B TLR4 IRF3 TLR7 IRF7 TLR8 IRAK1	Cytoplasm	No		✓
MYF6	HUMAN Myogenic factor 6	bHLHc4, MRF4	P23409	ENSG0000	Transcription factor	AK313287	Centronuclear myopathy, Autosomal dominant centronuclear myopathy, Thanatophoric dysplasia, Jeune syndrome, Thoracolaryngopelvic dysplasia, Ring fibers, Muscle weakness, Muscle cramp, Duchenne muscular dystrophy, Becker Muscular Dystrophy	https://www.targetvalidation.org/target/ENSG00000111046/associations	ID3 ID2 ID1	Nucleus,	No		✓
MYNN	HUMAN Myoneurin	SBBIZ1, ZBTB31, ZNF902	Q9NPC7	ENSG0000	Transcription factor	AY514901	Multiple myeloma, Chronic lymphocytic leukemia, Bladder carcinoma, Celiac disease, Chronic obstructive pulmonary disease, Lung neoplasm, Multiple sclerosis, Prostate carcinoma, Acute lymphoblastic leukemia, Endometrial neoplasm	https://www.targetvalidation.org/target/ENSG00000085274/associations	ISG15	Nucleus	No		✓
MYOG	HUMAN Myogenin	bHLHc3, MYF4	P15173	ENSG0000	Transcription factor,Cancer	CH471067	Achondrogenesis, Lethal congenital contracture syndrome type 1, Hypophosphatasia, Autosomal recessive cutis laxa type 2, Autosomal recessive cutis laxa type 2A, Obesity, Rhabdomyosarcoma, Alveolar rhabdomyosarcoma, Neuroblastoma, Melanotic neuroectodermal tumor	https://www.targetvalidation.org/target/ENSG00000122180/associations	TBX2 ID3 ID2 ID1 POLR2C CDK4 UBC TCF3 EGLN3 SMARCA4 MEF2A	Nucleus,	No		✓
MYOZ2	HUMAN Myozenin-2	C4orf5, CS-1	Q9NPC6	ENSG0000	Cancer,Cancer-Antigen	AJ252149	Cardiomyopathy, Hypertrophic cardiomyopathy, Rare familial disorder with hypertrophic cardiomyopathy, Familial dilated cardiomyopathy, Familial restrictive cardiomyopathy, Left ventricular hypertrophy, Cardiomegaly, Cardiac arrhythmia, Cardiomyopathy, Becker muscular dystrophy	https://www.targetvalidation.org/target/ENSG00000172399/associations	PPP3CB	Cytoplasm, myofibril, sarcomere, Z line	No		✓
NAB1	HUMAN NGFI-A-binding protein 1	NAB1	Q13506	ENSG0000	Transcription factor	AC006460	Biliary liver cirrhosis, Primary biliary cirrhosis, Femoral neck bone mineral density, Melanoma, Leukemia, Peripheral neuropathy, Congenital hypomyelinating neuropathy, Ovarian carcinoma, Rheumatoid arthritis, Melanoma	https://www.targetvalidation.org/target/ENSG00000138386/associations	EGR1	Nucleus	No		✓
NAE1	HUMAN NEDD8-activating enzyme E1 regulatory subunit	APPBP1, ula-1, HPP1	Q13564	ENSG0000	Others	AK298159	Hereditary thrombocytopenia with normal platelets, Autosomal thrombocytopenia with normal platelets, Atypical hemolytic-uremic syndrome, Down syndrome, Alzheimer's disease, Amyloidosis, Oropharyngeal disorder, Dyskeratosis congenita, Essential thrombocythemia, Fanconi anemia	https://www.targetvalidation.org/target/ENSG00000159593/associations	TP53BP2 APC APP UBA3	Cell membrane	Yes	https://www.bindingdb.org/uniprot/Q13564	✓
NAGK	HUMAN N-acetyl-D-glucosamine kinase	GlcNAc kinase	Q9UJ70	ENSG0000	Kinase	CR457271	Diabetes mellitus, Blackfan-Diamond anemia, MODY, Glycogen storage disease due to hepatic glycogen synthase deficiency, Leigh syndrome, Neoplasm metastasis, Prostatic neoplasm, Leprechaunism, Rabson-Mendenhall syndrome, Acute monocytic leukemia	https://www.targetvalidation.org/target/ENSG00000124357/associations	AURKB GLB1 CDK1 STK16		Yes	https://www.drugbank.ca/biodb/polypeptides/Q9UJ70	✓

NAP1L1	HUMAN Nucleosome assembly protein 1-like 1	NAP1L, NRP	P55209	ENSG0000(	Cancer,Cancer-Antigen	AK122670	Breast carcinoma, Melanoma, Colorectal cancer, Hypoxia, Central diabetes insipidus, Clostridium difficile infection, Diarrhea, Prostate carcinoma, Breast carcinoma, Brain neoplasm	https://www.targetvalidation.org/target/ENSG00000187109/associations	COPS2 TP53 HIST1H2BJ TTLL10	Nucleus	No		✓
NAP1L3	HUMAN Nucleosome assembly protein 1-like 3	BNAP	Q99457	ENSG0000(	Cancer,Cancer-Antigen	AL009173	Li-Fraumeni syndrome, Prostate carcinoma, Ovarian carcinoma, Breast carcinoma, Psoriasis, Glioma, Astrocytoma, Lung carcinoma, Melanoma, Glioblastoma multiforme	https://www.targetvalidation.org/target/ENSG00000186310/associations		Nucleus	No		✓
NAP1L4	HUMAN Nucleosome assembly protein 1-like 4	NAP2	Q99733	ENSG0000(	Others	AC131971	Diffuse large B-cell lymphoma, Hepatocellular carcinoma, Gastric carcinoma, Chronic alcoholic intoxification, Hypoxia, Ascites, Chronic pancreatitis, Abscess, Systemic lupus erythematosus, Beckwith-Wiedemann syndrome	https://www.targetvalidation.org/target/ENSG00000205531/associations	CSNK2A1 CSNK2A2	Nucleus	No		✓
NCK1	HUMAN Cytoplasmic protein NCK1	NCK, NCKalpha	P16333	ENSG0000(	Signalling	CH471052	Glioblastoma multiforme, Lung carcinoma, Albuminuria, Proteinuria, Nephrotic syndrome, Wiskott-Aldrich syndrome, Osteopenia, Obesity, Hyperglycemia, Diabetic nephropathy, Metastatic melanoma	https://www.targetvalidation.org/target/ENSG00000158092/associations	TNIK TBK1 DOK1 MAP3K14 MAP4K1 BLNK MINK1 NTRK2 PKN2 DAG1 GAB1 CBLB PAK2 PAK1 LCP2 TNK2 SOS1 PTK2 EFNB1 CSNK1G2 RAC1 BCAR1 EPHB1 CRK DCC WAS FLT3 KDR IRS1 EPHB2 CBL ERBB3 FLT1	Cytoplasm, Endoplasmic reticulum, Nucleus,	Yes	https://www.bindingdb.org/uniprot/P16333	✓
NCOA4	HUMAN Nuclear receptor coactivator 4	ARA70, ELE1, RFG	Q13772	ENSG0000(	Cancer	AK304123	Endocrine system disease, Adenocarcinoma, Papillary thyroid carcinoma, Thyroid neoplasm, Nonmedullary thyroid carcinoma, Prostatic neoplasm, Thyroid carcinoma, Prostate carcinoma, Papillary carcinoma, Bile duct carcinoma	https://www.targetvalidation.org/target/ENSG00000266412/associations	AKT1 AR PPARG AR		No		✓
NCOA5	HUMAN Nuclear receptor coactivator 5	bA465L10.6, CIA	Q9HCD5	ENSG0000(	Others	AL162458, AL035662	Autoimmune disease, Multiple sclerosis, Glycogen storage disease due to glucose-6-phosphatase deficiency, Phosphoenolpyruvate carboxykinase deficiency, Rheumatoid Arthritis, Agranulocytosis, B16 melanoma, Tyep II diabetes mellitus, Hashimoto's encephalitis, Liver carcinoma	https://www.targetvalidation.org/target/ENSG00000124160/associations	HTATIP2	Nucleus,	No		✓
NDE1	HUMAN Nuclear distribution protein nudE homolog 1	NudE	Q9NXR1	ENSG0000(	Cancer,Cancer-Antigen	BC001421	Lissencephaly, Microlissencephaly, Breast carcinoma, Microcephaly, Hydranencephaly, Schizophrenia, Nonorganic psychosis, Psychotic disorder, Epilepsy, Large dysplastic ears	https://www.targetvalidation.org/target/ENSG00000072864/associations	ZDHHC2 CDK5 YWHAG PAFAH1B1 MAPK1 CDK2 PRKACA CDK1 TUBG1	Cytoplasm, cytoskeleton, microtubule organizing center, centrosome, Chromosome, centromere, kinetochore, spindle, Cleavage furrow,	No		✓
NDRG1	HUMAN Protein NDRG1	CAP43, DRG1, NDR1, RTP, TDD5	Q92597	ENSG0000(	Kinase,Cancer	AF186190	Charcot-Marie-Tooth disease, Hereditary motor and sensory neuropathy, Charcot-Marie-Tooth disease type 4D, Neoplasm metastasis, Prostatic Neoplasm, Mammary neoplasm, Glioblastoma, Head and Neck squamous cell carcinoma, Renal cell carcinoma, Non-small cell lung carcinoma	https://www.targetvalidation.org/target/ENSG00000104419/associations	SGK2 SGK3 GSK3A GRK2 PRKACA TP53 SGK1 BCL2L11 RAB4A	Cytoplasm, cytosol, cytoskeleton, microtubule organizing center, centrosome, Nucleus, Cell membrane,	No		✓
NDRG2	HUMAN Protein NDRG2	KIAA1248, SYLD	Q9UN36	ENSG0000(	Kinase	AB033074	Transient ischemic attack, Colonic neoplasm, Cataract, Neoplasm metastasis, Glioblastoma, Liver carcinoma, Liver neoplasm, Alzheimer's disease, Renal cell carcinoma, Glioma	https://www.targetvalidation.org/target/ENSG00000165795/associations	SGK2 SGK3 RPS6KA1 CSNK2A1 AKT1 RPS6KB1 ATP1B1 SGK1	Cytoplasm, Cytoplasm, perinuclear region, Cell projection, growth cone	No		✓

NDUFAB1	HUMAN Acyl carrier protein, mitochondrial	SDAP	O14561	ENSG0000(	Others	CH471145	Epithelial neoplasm, Rheumatoid arthritis, Coronary artery disease, Hypertensive disease, Diabetes mellitus, Crohn's disease, Tuberculosis, Obesity, Intracranial neoplasm, Hereditary nonpolyposis colorectal cancer	https://www.targetvalidation.org/target/ENSG00000004779/associations		Mitochondrion	Yes		✓
												https://www.drugbank.ca/biodb/polypeptides/O14561	
NDUFV3	HUMAN NADH dehydrogenase [ubiquinone] flavoprotein 3, mitochondrial	CI-9k, NY-REN-4, NDUV3	P56181	ENSG0000(	Cancer,Cancer-Antigen	CR542190	Bipolar disorder, Prostate neoplasm, Chronic obstructive pulmonary disease, Abnormal glucose tolerance, Hepatic steatosis, Acanthosis nigricans, Systemic lupus erythematosus, Obesity, Coronary artery disease, Polycystic ovary syndrome	https://www.targetvalidation.org/target/ENSG00000160194/associations	NDUFV2 NDUFS3 NDUFS3	Mitochondrion inner membrane	Yes		✓
												https://www.drugbank.ca/biodb/polypeptides/P56181	
NEDD9	HUMAN Enhancer of filamentation 1	CAS-L, CASS2, HEF1	Q14511	ENSG0000(	Cancer,Cancer-Antigen	AL022098	Prostate carcinoma, Central nervous system cancer, Alzheimer's disease, Parkinson's disease, Melanoma, Rheumatoid arthritis, Breast neoplasm, Gastric carcinoma, Glioma, Astrocytoma	https://www.targetvalidation.org/target/ENSG00000111859/associations	TRIP6 PTK2B RAPGEF1 PTK2 PTPN12 CRKL CRK LYN FYN LCK ABL1 BCAR1 PTK2 FZR1 TRIP6 ZYX BCAR3 DIMT1	Cytoplasm, cell cortex, Nucleus, Golgi apparatus, Cell projection, lamellipodium, Cytoplasm, Cell junction, focal adhesion,	No		✓
NEK11	HUMAN Serine/threonine-protein kinase Nek11	NimA-related protein kinase 11	Q8NG66	ENSG0000(	Kinase	AC055733	Central nervous system cancer, Ependymoma, Colonic neoplasm, Hepatocellular carcinoma, Colorectal carcinoma, Ovarian carcinoma, Serous cystadenocarcinoma, Breast carcinoma, Nasopharyngeal neoplasm, Malignant mesothelioma	https://www.targetvalidation.org/target/ENSG00000114670/associations	NEK2 CHEK1 BLM CDC25A	Nucleus, nucleolus,	Yes		✓
												https://www.bindingdb.org/uniprot/Q8NG66	
NEK3	HUMAN Serine/threonine-protein kinase Nek3	HSPK36, MGC29949	P51956	ENSG0000(	Kinase	CH471075	Li-Fraumeni syndrome, Breast carcinoma, Prostate carcinoma, Macroglobulinemia, Ovarian carcinoma, Pick disease, Glioblastoma multiforme, Progressive supraclear palsy, Melanoma, Acute promyelocytic leukemia	https://www.targetvalidation.org/target/ENSG00000136098/associations	MAP2K1 CSN2 ARID3A YWHAZ SMARCAD1 MDM2 PRLR HNRNPK HSPB1 KAT5 VAV2 VAV1 ZYX CC2D1A URI1 CDCA7L RNASEH2C LUZP1 METTL3 PRDM10 PAXBP1	Cytoplasm	Yes		✓
												https://www.bindingdb.org/uniprot/P51956	
NEK7	HUMAN Serine/threonine-protein kinase Nek7	NEK7	Q8TDX7	ENSG0000(	Kinase	AC093561	Glioblastoma multiforme, Melanoma, Lymphoma, Diffuse large B-cell lymphoma, Lung carcinoma, Breast neoplasm, Retinoblastoma, Hashimoto's encephalitis, Liver carcinoma, Head and Neck squamous cell carcinoma	https://www.targetvalidation.org/target/ENSG00000151414/associations	NEK9 NEK9	Nucleus, Cytoplasm, cytoskeleton, spindle pole, microtubule organizing center, centrosome,	Yes		✓
												https://www.bindingdb.org/uniprot/Q8TDX7	
NELFE	HUMAN Negative elongation factor E	NELF-E, RD, RDBP	P18615	ENSG0000(	Others	AK300717	HIV infection, Teratozoospermia, Hepatocellular carcinoma, Systemic lupus erythematosus, Breast carcinoma, Type I diabetes mellitus, Peripheral vascular disease, Acquired immunodeficiency syndrome, Amyotrophic lateral sclerosis, Frontotemporal lobar degeneration	https://www.targetvalidation.org/target/ENSG00000204356/associations	NELFA AURKB NELFB NELFCD HSP90AB1 NELFCD NELFB NELFA	Nucleus,	No		✓
NEUROD1	HUMAN Neurogenic differentiation factor 1	BHF-1, bHLHa3, MODY6, NEUROD	Q13562	ENSG0000(	Transcription factor	AB018693	Diabetes mellitus, MODY, Type II diabetes mellitus, Retinal dystrophy, Benign adult familial myoclonic epilepsy, Nervous system disorder, Impaired glucose tolerance, Progressive myoclonic epilepsy type 3, Cystic fibrosis, Bilateral polymicrogyria, Astrocytoma	https://www.targetvalidation.org/target/ENSG00000162992/associations	PDIK1L MAPKAPK5 ZFHX3 EP300 PITX1 CDK9 CDK7 GSK3B EPHB2 GATA3 CDK4 BMPR1B NTRK2 CDKN1A RREB1	Cytoplasm	No		✓

NEUROD4	HUMAN Neurogenic differentiation factor 4	ATH-3, Atoh3, bHLHa4	Q9HD90	ENSG0000(	Transcription factor	CH471054	Beauce type autosomal recessive ataxia, Autosomal recessive cerebellar ataxia - psychomotor retardation, Spinocerebellar ataxia type 31, Serum IgG glycosylation measurement, Type II diabetes mellitus, MODY, H1N1, Atherosclerosis, Small cell lung carcinoma, Chronic lymphocytic leukemia	https://www.targetvalidation.org/target/ENSG00000123307/associations		Nucleus	No		✓
NF2	HUMAN Merlin	MERL, SCH	P35240	ENSG0000(	Cancer	Y18000	Neurofibromatosis type 2, Meningioma, Schwannomatosis, Neurilemmoma, Acoustic Neuroma, Mesothelioma, Malignant mesothelioma, Renal cell carcinoma, Neuropathy, Cataract	https://www.targetvalidation.org/target/ENSG00000186575/associations	PAK2 PAK1 PPP1CB PRKX GRK6 AKT1 PRKACA CD44 ERBB2 PRKY XPO1 PXN LATS1 EZR RALGDS PAK1 LATS2 HGS SDCBP SPTBN1 TARBP2 SCHIP1 DCAF1 SGSM3 CCNB1IP1 GTPBP4	Cell projection, filopodium membrane, ruffle membrane, Peripheral membrane protein, Cytoplasmic side, Nucleus,	No		✓
NFATC4	HUMAN Nuclear factor of activated T-cells, cytoplasmic 4	NFAT3, NFAC4	Q14934	ENSG0000(	Transcription factor	EU887634	X-linked intellectual disability - epilepsy, Hereditary hyperekplexia, Alzheimer's disease, Mammary neoplasm, Cardiomegaly, Cardiomyopathy, Degenerative polyarthritis, Retinal disease, LEOPARD syndrome, Mild cognitive disorder	https://www.targetvalidation.org/target/ENSG00000100968/associations	DYRK1B RPS6KA6 NLK STK36 PAK6 WNK1 VRK1 SRPK1 SGK494 UHMK1 PXK CAMK4 MAPK14 RPS6KA1 RPS6KA2 STK38 ROCK1 MAPK7 PAK1 PPP3CA ACVR1 CDK5 CDK6 SRPK2 PPP3R1 EPHB1 PRKAA2 RPS6KA3 CDK7	Cytoplasm	No		✓
NFE2	HUMAN Transcription factor NF-E2 45 kDa subunit	NF-E2	Q16621	ENSG0000(	Transcription factor	CR450284	Rare hemorrhagic disorder due to a constitutional platelet anomaly, Congenital dyserythropoietic anemia, Hereditary thrombocytopenia with normal platelets, Polycythemia vera, Chronic myeloproliferative disorder, Acute erythroblastic leukemia, Thrombocytosis, Myeloproliferative disease, Primary myelofibrosis, Sepsis	https://www.targetvalidation.org/target/ENSG00000123405/associations	MAPK8 PEX14 MAFK MAFG ITCH MAPK8 MAPK9 WWP1 MAFF	Nucleus, PML body, Cytoplasm,	No		✓
NFE2L2	HUMAN Nuclear factor erythroid 2-related factor 2	NRF2	Q16236	ENSG0000(	Transcription factor,Cancer	AC019080	Adenocarcinoma, Diabetic nephropathy, Non-alcoholic fatty liver disease, Liver cirrhosis, Acute kidney injury, Pulmonary fibrosis, Non-small cell lung carcinoma, Liver carcinoma, Acute lung injury, Esophagus squamous cell carcinoma	https://www.targetvalidation.org/target/ENSG00000116044/associations	KLF2 NOX1 MAFF EIF2AK3 PAG1 PGAM5 SIRT1 CREBBP HDAC2 GADD45GIP1 MAPK14 MAPK11 KEAP1 CUL3 HDAC1 TRIM28 MAPK7 EP300 PRKCD PRKCE CDK5 CSNK2A1 RBX1 MAPK12 MAP2K6 SMARCA4 GSK3B PIK3CG	Cytoplasm, cytosol, Nucleus,	Yes	https://www.bindingdb.org/uniprot/Q16236	✓
NFIA	HUMAN Nuclear factor 1 A-type	KIAA1439	Q12857	ENSG0000(	Transcription factor	AC092784	Bipolar disorder, Celiac disease, Genetic renal or urinary tract malformation, Congenital neurologic anomalies, Amyloidosis, Alzheimer's disease, Asthma, Congenital hypoplasia of lung, Familial Alzheimer's disease (FAD), Impaired cognition	https://www.targetvalidation.org/target/ENSG00000162599/associations	TFAP4	Nucleus,	No		✓
NFIB	HUMAN Nuclear factor 1 B-type	NFI-RED, NFIB2, NFIB3	O00712	ENSG0000(	Transcription factor,Cancer	AK131233	Adenocarcinoma, Lung carcinoma, Adenoid cystic carcinoma, Agenesis of corpus callosum, Aplasia cutis congenita, Lipoma, Alzheimer's disease, Amyloidosis, Central neuroblastoma, Multiple lipomatosis	https://www.targetvalidation.org/target/ENSG00000147862/associations	FOXA1 NFIX NFIC	Nucleus,	No		✓
NFIL3	HUMAN Nuclear factor interleukin-3-regulated protein	E4BP4, IL3BP1, NF-IL3A, NFIL3A	Q16649	ENSG0000(	Transcription factor,Signalling	EF028070	Central nervous system cancer, Glioma, Glioblastoma multiforme, Circadian rhythm sleep disorder, Attention deficit hyperactivity disorder, Bipolar disorder, Schizophrenia, Mental depression, Breast carcinoma, Amyotrophic lateral sclerosis	https://www.targetvalidation.org/target/ENSG00000165030/associations	DR1 STAT6	Nucleus	No		✓

NFKBIA	HUMAN NF-kappa-B inhibitor alpha	IkappaBalpha, IKBA, MAD-3, NFKBI	P25963	ENSG0000(	Kinase,Transcription factor,Signalling,Cancer	AY496422	Primary immunodeficiency, Ectodermal dysplasia syndrome, Hodgkin's disease, Psoriasis, Liver carcinoma, Myocardial Ischemia, Brain ischemia, Hypohidrotic ectodermal dysplasia with immunodeficiency, Transcobalamin deficiency	https://www.targetvalidation.org/target/ENSG00000100906/associations	IKBKG NCOR2 TRAF6 MAP3K2 BTRC NOD1 HDAC5 FBXW11 TBK1 NKIRAS1 NKIRAS2 NOD2 MAP3K3 MAP3K14 DNAJA3 SIRT1 HDAC2 COMMD1 MAVS MAPK14 NFE2L2 RPS6KA1 RPS6KA2 IKBKE CUL1 HDAC1 ATM PTPN13 BTK	Cytoplasm, Nucleus,	Yes	https://www.bindingdb.org/uniprot/P25963	✓
NFX1	HUMAN Transcriptional repressor NF-X1	MGC20369, NFX2, TEG-42, Tex42	Q12986	ENSG0000(	Transcription factor	AL356472, AL356218	Glioblastoma multiforme, Lung carcinoma, Adenocarcinoma, Colorectal adenocarcinoma, HPV infection, Gastric carcinoma, Breast carcinoma, Specific language impairment, Cystic fibrosis, Sarcoidosis	https://www.targetvalidation.org/target/ENSG00000086102/associations	SIN3A PABPC1	Nucleus,	No		✓
NFYA	HUMAN Nuclear transcription factor Y subunit alpha	CBF-B, HAP2, NF-YA	P23511	ENSG0000(	Transcription factor	BC039244	Achondrogenesis, Brachyolmia, Autosomal dominant secondary polycythemia, Congenital enteropathy due to enteropeptidase deficiency, Autosomal recessive spondylocostal dysostosis, Adenocarcinoma, Essential hypertension, NOS hemophilia, Head and Neck squamous cell carcinoma, Fibrolamellar hepatocellular carcinoma	https://www.targetvalidation.org/target/ENSG00000001167/associations	RNF4 GLRX CDK2 JUN TP53 CEBPA NFYB PADI3 NR5A1 KPNB1 NFYC ZHX1	Nucleus,	No		✓
NFYB	HUMAN Nuclear transcription factor Y subunit beta	CBF-A, HAP3, NF-YB	P25208	ENSG0000(	Transcription factor	CH471054	Lung carcinoma, Breast carcinoma, Squamous cell lung carcinoma, Melanoma, Acute myelocytic leukemia, Essential hyertension, Psoriasis, Crohn's disease, Colorectal cancer, HIV infection	https://www.targetvalidation.org/target/ENSG00000120837/associations	RNF4 CIITA NFYA YBX3 TP53 MYC HAP1 YBX1 EP300 CEBPZ ELF1 NFYC CNTN2	Nucleus,	No		✓
NHLH1	HUMAN Helix-loop-helix protein 1	bHLHa35, HEN1, NSCL1	Q02575	ENSG0000(	Transcription factor	M97507	Arrhythmogenic right ventricular dysplasia, Familial isolated arrhythmogenic right ventricular dysplasia, Familial long QT syndrome, Romano-Ward syndrome, Familial dilated cardiomyopathy, Congenital abnormality, Cleft lip, Non-small cell lung carcinoma, Dysmorphism, Central neuroblastoma, Neonatal deformity	https://www.targetvalidation.org/target/ENSG00000171786/associations	LMO1 CD302	Nucleus	No		✓
NICN1	HUMAN Nicolin-1	MGC12936	Q9BSH3	ENSG0000(	Cancer,Cancer-Antigen	BC050653	Sepsis, Nasopharyngeal squamous cell carcinoma, Inflammatory bowel disease, Crohn's disease, Bladder neoplasm	https://www.targetvalidation.org/target/ENSG00000145029/associations		Nucleus	No		✓
NIM1K	HUMAN Serine/threonine-protein kinase NIM1	NIM1	Q8IY84	ENSG0000(	Kinase	CH471119	Breast carcinoma, Central nervous system cancer, Glioma, Papillary adenocarcinoma, Hypercalcemia, Thyroid carcinoma, Neutrophilia, Melanoma, Retinoblastoma, Glioma	https://www.targetvalidation.org/target/ENSG00000177453/associations	CRK		Yes	https://www.bindingdb.org/uniprot/Q8IY84	✓
NKX2-5	HUMAN Homeobox protein Nkx-2.5	CSX, NKX2.5, NKX2E	P52952	ENSG0000(	Transcription factor	AK290615	Atrial septal defect-atrioventricular conduction defects, Genetic cardiac anomaly, Congenital heart disease, Tetralogy of Fallot, Ventricular septal defect, Hypoplastic left heart syndrome, Atrial fibrillation, Ostium secundum atrial septal defect, Double outlet right ventricle, Myotonic dystrophy	https://www.targetvalidation.org/target/ENSG00000183072/associations	HIPK2 JARID2 HIPK1 SMAD4 CSNK2A1 SUMO1 GATA4 SMAD4 SRF HDAC1 FOXH1 TBX2 GATA4 TBX5	Nucleus	No		✓
NLK	HUMAN Serine/threonine-protein kinase NLK	NLK	Q9UBE8	ENSG0000(	Kinase	DQ316259	Gaucher disease, Purine nucleoside phosphorylase deficiency, Gaucher disease type 3, Colonic neoplasm, Liver carcinoma, Prostate carcinoma, Liver and Intrahepatic biliary tract carcinoma, Liver neoplasm, Ovarian carcinoma, Neoplasm metastasis	https://www.targetvalidation.org/target/ENSG00000087095/associations	LEF1 TAB2 FZD3 HIPK2 BMPR2 WNT5A MAP3K7 STMN1 CTNNB1 PPARA SNTA1 PRKACA LCK STMN2 MAP3K7 TCF3 FOXO1 SMAD4 TP53 HIPK2 RPS6KA1 BCL2 HSF1 NR3C1 NOTCH1 TCF7L2 LIFR MYB FOS CDKN1A	Nucleus	Yes	https://www.bindingdb.org/uniprot/Q9UBE8	✓

NLRP2	HUMAN NACHT, LRR and PYD domains-containing protein 2	NALP2, NBS1, PAN1, PYPAF2	Q9NX02	ENSG0000(	Transcription factor	AK000517	Steinert myotonic dystrophy, Pancreatic ductal adenocarcinoma, Li-Fraumeni syndrome, Breast carcinoma, Nijmegen breakage syndrome, Ataxia telangiectasia, Hematologic neoplasm, Chronic lymphocytic leukemia, Crohn's disease, Prostate carcinoma	https://www.targetvalidation.org/target/ENSG00000022556/associations	IKBKG SUGT1 SLC29A1 RFWD2 CARD17 PKN1 ACTR2 CARD18 CASP1 MEFV CHUK IKBKB IKBKB PYCARD CARD8 CHUK	Cytoplasm	No		✓
NLRP4	HUMAN NACHT, LRR and PYD domains-containing protein 4	NALP4, PAN2, PYPAF4, RNH2	Q96MN2	ENSG0000(	Transcription factor	AF482706	Diffuse large B-cell lymphoma, Breast carcinoma, Central nervous system cancer, Macroglobulinemia, Crohn's disease, Chronic lymphocytic leukemia, Adenocarcinoma, Colorectal carcinoma, Colorectal cancer, Polyp	https://www.targetvalidation.org/target/ENSG00000160505/associations	SUGT1 NLRC4 BECN1 CHUK SUGT1		No		✓
NME4	HUMAN Nucleoside diphosphate kinase, mitochondrial	NDPKD, nm23-H4, NM23D	O00746	ENSG0000(	Kinase	Z97634	Small cell lung carcinoma, Squamous cell carcinoma, Pancreatic ductal adenocarcinoma, Central neuroblastoma, Gastric carcinoma, Colon carcinoma, Lip and Oral cavity carcinoma, Oral neoplasm, Neuroblastoma, Leukemia	https://www.targetvalidation.org/target/ENSG00000103202/associations		Mitochondrion intermembrane space	No		✓
NME5	HUMAN Nucleoside diphosphate kinase homolog 5	nm23-H5	P56597	ENSG0000(	Kinase	AK312181	Azoospermia, Li-Fraumeni syndrome, Ciliopathy, Male infertility with azoospermia or oligozoospermia due to single gene mutation, Primary ciliary dyskinesia, Transitional cell carcinoma, Pancreatic carcinoma, Urothelial carcinoma, Acrofacial dysostosis, Frontonasal dysplasia	https://www.targetvalidation.org/target/ENSG00000112981/associations	RELA		No		✓
NME6	HUMAN Nucleoside diphosphate kinase 6	IPIA-ALPHA, NM23-H6	O75414	ENSG0000(	Kinase	AK297364	Chronic lymphocytic leukemia, Colonic neoplasm, Glioma, Lung carcinoma, Crohn's disease, Gastric carcinoma, Juvenile dermatomyositis, Breast carcinoma, Acute monocytic leukemia, Adenocarcinoma	https://www.targetvalidation.org/target/ENSG00000172113/associations			No		✓
NME7	HUMAN Nucleoside diphosphate kinase 7	NM23-H7	Q9Y5B8	ENSG0000(	Kinase	AL031726, AL356852, Z99758	Venous thromboembolism, Ovarian carcinoma, Breast carcinoma, Pancreatic carcinoma, Situs ambiguus, Hypertensive disease, Osteoporosis, Colon carcinoma, Astrocytoma, Melanoma	https://www.targetvalidation.org/target/ENSG00000143156/associations			No		✓
NME8	HUMAN Thioredoxin domain-containing protein 3	CILD6, NM23-H8, SPTRX2, TXNDC3	Q8N427	ENSG0000(	Kinase	BC036816	Ciliopathy, Primary ciliary dyskinesia, Azoospermia, Osteosarcoma, Sepsis, Ciliary motility disorder, Kartagener Syndrome, Sinusitis, Paranasal Sinus Disease, Autosomal recessive predisposition	https://www.targetvalidation.org/target/ENSG00000086288/associations		Cytoplasm	No		✓
NMRAL1	HUMAN NmrA-like family domain-containing protein 1	HSCARG	Q9HBL8	ENSG0000(	Others	BC002927	Ovarian carcinoma, Malignant peripheral nerve sheath tumor, Autoimmune disease, Schizopherenia, Rheumatoid arthritis	https://www.targetvalidation.org/target/ENSG00000153406/associations	IKBKE TRAF3	Cytoplasm, perinuclear region, Nucleus,	Yes	https://www.drugbank.ca/biodb/polypeptides/Q9HBL8	✓
NOL4	HUMAN Nucleolar protein 4	HRIHFB2255, NOLP	O94818	ENSG0000(	Others	AC010798	Schizophrenia, Circadian rhythm, Lung carcinoma, Cervix carcinoma, Astrocytoma, Ependymoma, Medulloblastoma, Glioblastoma multiforme, Azoospermia, Psoriasis	https://www.targetvalidation.org/target/ENSG00000101746/associations		Nucleus, nucleolus	No		✓

NPM1	HUMAN Nucleophosmin	B23, NPM	P06748	ENSG0000(	Cancer	U89321	HIV infection, Lymphoid neoplasm, Acute myelocytic leukemia, Acute promyelocytic leukemia, Lymphomatoid papulosis, Gastric neoplasm, Melanoma, Adenocarcinoma, Kidney neoplasm, Osteosarcoma	https://www.targetvalidation.org/target/ENSG00000181163/associations	DYRK1B ALK PADI4 PLK2 DYRK4 PLK3 MAP3K14 AURKB CDKN2A MAPKAPK5 TNNI3K ATR PRKCD PRKCE CDK6 CXCR4 PLK1 NEK2 GZMM CDK9 MAPK8 AKT1 TYK2 CDK2 PRKCH EIF2AK2 PRKCA SRC YES1 CDK1 PRKCB	Nucleus, nucleolus Cytoplasm, cytoskeleton, microtubule organizing center, centrosome,		Yes	https://www.bindingdb.org/uniprot/P06748	✓
NPY5R	HUMAN Neuropeptide Y receptor type 5	NPYR5	Q15761	ENSG0000(	Others	AY322538	Obesity, Familial hyperinsulinism, Congenital isolated hyperinsulinism, Obesity due to melanocortin 4 receptor deficiency, Absence epilepsy, Dyslipidemia, Liver disease, Type II diabetes mellitus, Left ventricular hypertrophy, MODY	https://www.targetvalidation.org/target/ENSG00000164129/associations	PYY NPY PPY	Cell membrane, Multi-pass membrane protein,	Transmembrane	Yes	https://www.bindingdb.org/uniprot/Q15761	✓
NR0B2	HUMAN Nuclear receptor subfamily 0 group B member 2	SHP	Q15466	ENSG0000(	Transcription factor	AL356390	Obesity, Obesity due to melanocortin 4 receptor deficiency, Liver carcinoma, Decreased resting energy expenditure, Phenotypic variability, Lipoidosis, Autosomal recessive predisposition, Burkitts lymphoma, Hyperplasia, LCAT deficiency	https://www.targetvalidation.org/target/ENSG00000131910/associations	ESR2 HDAC1 PRKCZ MDM2 HNF4A PPARG AR ESR1 NR1I2 PPARA SIRT1 NR1I3 AR RXRA ESR1 NR1I2 RARA RARB ESR2 THRB NR5A2 ESRRG THRA NR1H3 HNF4A RXRB NR1H2 ONECUT1	Nucleus		No		✓
NR1D2	HUMAN Nuclear receptor subfamily 1 group D member 2	BD73, EAR-1r, Hs.37288, HZF2, RVR	Q14995	ENSG0000(	Transcription factor	BC045613	Breast carcinoma, Sepsis, Melanoma, Endometriosis, Hepatitis C infection, Ulcerative colitis, Liver fibrosis, Pruritus, Liver cirrhosis, Pseudohyperkalemia cardiff	https://www.targetvalidation.org/target/ENSG00000174738/associations	ARNTL	Nucleus		Yes	https://www.bindingdb.org/uniprot/Q14995	✓
NR1H2	HUMAN Oxysterols receptor LXR-beta	LXR-b, NER, NER-I, RIP15, UNR	P55055	ENSG0000(	Transcription factor,Cancer	AC008655	Bladder carcinoma, Abnormality of lipid metabolism, Liver carcinoma, Cholestasis, Alzheimer's disease, Type II diabetes mellitus, Xeroderma pigmentosum, Cockayne syndrome, Obesity, Atherosclerosis	https://www.targetvalidation.org/target/ENSG00000131408/associations	CAMK2A NR0B2 PPARA PPARD RXRG PPARG ACVRL1 RXRB RXRA CIDEA PPARA HDAC4 PPARG RXRA PPARD ABCA1 RXRB	Nucleus		Yes	https://www.bindingdb.org/uniprot/P55055	✓
NR1I2	HUMAN Nuclear receptor subfamily 1 group I member 2	PXR, SXR	O75469	ENSG0000(	Transcription factor	AF084645	Inborn errors of metabolism, Osteosarcoma, Adenocarcinoma, Obesity, Fatty liver, Metabolic syndrome X, Diabetes mellitus, Neonatal jaundice, Barrett esophagus, Hypophosphatemia	https://www.targetvalidation.org/target/ENSG00000144852/associations	NR0B2 FOXO1 CDK2 RXRA NCOR2 FOXO1 RXRA NCOA1 HNF4A NR0B2	Nucleus		Yes	https://www.bindingdb.org/uniprot/O75469	✓
NR2C1	HUMAN Nuclear receptor subfamily 2 group C member 1	TR2, TR2-11	P13056	ENSG0000(	Transcription factor	AK291319	Hereditary elliptocytosis, Alzheimer's disease, Hereditary methemoglobinemia, Non-syndromic congenital cataract, Blackfan-Diamond anemia, Endometriosis, Parkinson's disease, Prostate carcinoma, Melanoma, Pick disease	https://www.targetvalidation.org/target/ENSG00000120798/associations	AR HDAC3 NR2C2 ESR1	Nucleus		No		✓
NR2C2	HUMAN Nuclear receptor subfamily 2 group C member 2	TAK1, TR4	P49116	ENSG0000(	Transcription factor	AK290590	Kallmann syndrome, Male infertility with azoospermia or oligozoospermia due to single gene mutation, Endometriosis, Prostate carcinoma, Breast carcinoma, Liver carcinoma, Melanoma, Osteoarthritis, Sickle cell anemia, Cockayne syndrome	https://www.targetvalidation.org/target/ENSG00000177463/associations	TRAF6 TAB2 TAB3 NR2C2AP JAZF1 TAB1 PRKAA1 TRAF2 HNF4A NR2C1 AR ESR1 SMAD6 CHUK IKBKB TRAF2 IKBKB AR CHUK PRKAA1	Nucleus		Yes	https://www.bindingdb.org/uniprot/P49116	✓

NR2E1	HUMAN Nuclear receptor subfamily 2 group E member 1	TLL, TLX, XTLL	Q9Y466	ENSG0000(	Transcription factor	AK131541	Retinal dystrophy, Retinitis pigmentosa, Cone rod dystrophy, Late infantile neuronal ceroid lipofuscinosis, Schizophrenia, Bipolar disorder, Precursor T-Cell lymphoblastic leukemia-lymphoma, Brain neoplasm, Central neuroblastoma	https://www.targetvalidation.org/target/ENSG00000112333/associations	ERBIN	Nucleus	No		✓
NR2E3	HUMAN Photoreceptor-specific nuclear receptor	PNR, rd7, RP37	Q9Y5X4	ENSG0000(	Transcription factor	AB307710	Retinal dystrophy, Vitreoretinal degeneration, Goldmann-Favre syndrome, Retinitis pigmentosa, Night blindness, Lens opacities, Mental deficiency, Enhanced S-Cone syndrome, Stickler syndrome, Rare isolated myopia	https://www.targetvalidation.org/target/ENSG00000278570/associations	CRX NR1D1	Nucleus	Yes	https://www.bindingdb.org/uniprot/Q9Y5X4	✓
NR3C1	HUMAN Glucocorticoid receptor	GR, GRL	P04150	ENSG0000(	Transcription factor,Signalling	AH005496	Glucocorticoid resistance, Hypertensive disease, Bipolar disorder, Depressive disorder, Hypokalemia, Mental depression, Schizophrenia, Fatigue, Infectious Meningitis, Dermatomyositis	https://www.targetvalidation.org/target/ENSG00000113580/associations	DAXX NLK SMARCD1 SMARCE1 SMARCC1 CREBBP MAPK15 PELP1 MAPK6 MAPK14 MAPK11 TRIP6 NCOA2 HES1 TDG FKBP5 TRIM28 MAPK7 SMARCB1 EP300 RELA SET CDK5 SMAD3 PRKDC SUMO1 MAPK10 PPP5C SMARCA4 GSK3B NRIP1 MAPK9	Cytoplasm	Yes	https://www.bindingdb.org/uniprot/P04150	✓
NR4A1	HUMAN Nuclear receptor subfamily 4 group A member 1	GFRP1, HMR, NAK1, NUR77, TR3	P22736	ENSG0000(	Transcription factor,Signalling	AK297526	Breast carcinoma, Hepatocellular carcinoma, Insulin resistance, Endometriosis, Kidney disease, Proteinuria, Myocardial ischemia, Gastric neoplasm, Acute myelocytic leukemia, Melanoma	https://www.targetvalidation.org/target/ENSG00000123358/associations	RPS6KA6 GADD45GIP1 RPS6KA1 PDK1 MAPK7 EP300 CSNK2A1 RPS6KA3 RXRG MAPK9 MAPK8 CTNNB1 GK AKT1 MAPK1 RXRA BCL2 RARA NR3C1 RPS6KA5 BNIP3L NCOR2 HIF1A TP53 BCL2 NR3C1 RPS6KA3 RXRA STAT3	Cytoplasm, Nucleus,	Yes	https://www.bindingdb.org/uniprot/P22736	✓
NR4A2	HUMAN Nuclear receptor subfamily 4 group A member 2	HZF-3, NOT, NURR1, RNR1, TINUR	P43354	ENSG0000(	Transcription factor,Signalling,Cancer	AK291456	Breast carcinoma, Schizophrenia, Bipolar disorder, Lewy body disease, Colorectal neoplasm, Psoriatic arthritis, Unipolar depression, Hyperphenylalaninemia, Crohn's disease, Pituitary cancer	https://www.targetvalidation.org/target/ENSG00000153234/associations	PIAS4 RPS6KA1 RXRB MAPK1 CDKN1C	Cytoplasm, Nucleus,	Yes	https://www.bindingdb.org/uniprot/P43354	✓
NR6A1	HUMAN Nuclear receptor subfamily 6 group A member 1	GCNF, RTR	Q15406	ENSG0000(	Transcription factor	AF004291	Brachyolmia, Achondrogenesis, Dyschondrosteosis-nephritis, Choriocarcinoma, Cleft lip, Cleft palate, Breast carcinoma, Prostate carcinoma, Restenosis, Arteriosclerosis	https://www.targetvalidation.org/target/ENSG00000148200/associations	NCOR1 UIMC1	Nucleus	No		✓
NRAS	HUMAN GTPase NRas	HRAS1	P01111	ENSG0000(	Kinase,Cancer	X00644	Noonan syndrome, Lymphoid neoplasm, Juvenile myelomonocytic leukemia, Melanoma, Nevus sebaceous, Melanocytic nevus, Follicular thyroid carcinoma, Adenocarcinoma, Epithelial neoplasm, Lung carcinoma	https://www.targetvalidation.org/target/ENSG00000213281/associations	ZDHHC9 SHOC2 SYNGAP1 RAPGEF5 RAPGEF6 RASGEF1A RASGEF1C RASGRP3 RASGRP2 RASGRF1 RAPGEF1 RIN1 RASGEF1B SOS1 ACVR1 PPP2CB CALM1 IQGAP1 PIK3CA NF1 RASA1 SRC BCL2 RAP2A RAF1 RASA4 RAF1 SOS1	Cell membrane	Yes	https://www.bindingdb.org/uniprot/P01111	✓
NRBF2	HUMAN Nuclear receptor-binding factor 2	COPR1, COPR2, DKFZp564C1664, FLJ30395	Q96F24	ENSG0000(	Others	AL136656	Glioblastoma multiforme, Schizophrenia, Breast carcinoma, Lung carcinoma, Sjogren's syndrome, Psoriasis, Atopic eczema, Pancreatic carcinoma, Melanoma, Multiple myeloma	https://www.targetvalidation.org/target/ENSG00000148572/associations	IKBKG ATG14 PIK3C3	Nucleus	No		✓
NRBP2	HUMAN Nuclear receptor-binding protein 2	DKFZp434P086	Q9NSY0	ENSG0000(	Kinase	AL137662	Hepatocellular carcinoma, Melanoma, Medulloblastoma	https://www.targetvalidation.org/target/ENSG00000185189/associations	TRIP10 CBL CAMKK1 SOX10	Cytoplasm	No		✓

NRF1	HUMAN Nuclear respiratory factor 1	ALPHA-PAL,	Q16656	ENSG0000(	Transcription factor	EU159452	Type II diabetes mellitus, Neuroblastoma, Bowen's Disease, Hepatitis B, Alzheimer's disease, Breast carcinoma, Parkinson's disease, Huntington disease, Fibrosis, Schizophrenia	https://www.targetvalidation.org/target/ENSG00000106459/associations	PPARGC1A CSNK2A1 DYNLL1 AKT1 JUND JUNB JUN EIF2S1 TFAM EIF2S2 PPRC1	Nucleus,	No		✓
NRIP1	HUMAN Nuclear receptor-interacting protein 1	RIP140	P48552	ENSG0000(	Transcription factor	AL163207	Breast carcinoma, Central nervous system cancer, Glioma, Mammary neoplasm, Heart failure, Myocardial Infarction, Endometrial neoplasm, Schizophrenia, Male infertility, Epithelial ovarian cancer	https://www.targetvalidation.org/target/ENSG00000180530/associations	HDAC5 PRMT1 KAT2B FHL1 CTBP1 SUMO1 NR0B1 AHR RXRB RXRA THRA RARA AR JUN ESR1 MAP3K7 E2F1 NR3C1 HDAC3 AR RXRA HDAC1 ESR1 NR5A1 RARA NR1H3 RXRB	Nucleus	Yes	https://www.drugbank.ca/biodb/polypeptides/P48552	✓
NRIP3	HUMAN Nuclear receptor-interacting protein 3	C11orf14	Q9NQ35	ENSG0000(	Transcription factor	AY211930	Prostate carcinoma, Medulloblastoma, Azoospermia, Breast carcinoma, Melanoma, Ependymoma, Astrocytoma, Tuberculosis, Cystic fibrosis, Ovarian carcinoma	https://www.targetvalidation.org/target/ENSG00000175352/associations			No		✓
NSF	HUMAN Vesicle-fusing ATPase	SKD2	P46459	ENSG0000(	Cancer,Cancer-Antigen	AK290204	Retinitis pigmentosa, LCAT deficiency, Astrocytoma, Glioblastoma multiforme, Hyperlipoproteinemia type 5, Schizophrenia, Cytomegalovirus infections, Progressive supranuclear palsy, Plasmacytoma anaplastic, Dyslexia	https://www.targetvalidation.org/target/ENSG00000073969/associations	PTPN9 PTPN1 FER SRC CD28 FES GABARAP ARRB1 NAPA VAPA STX4	Cytoplasm,	No		✓
NTPCR	HUMAN Cancer-related nucleoside-triphosphatase	C1orf57	Q9BSD7	ENSG0000(	Kinase,Cancer	NA	Melanoma, Lung adenocarcinoma, Western quine encephalitis, Osteoscarcoma, Glioblastoma multiforme, Hepatitis C infection, Hepatocellular carcinoma	https://www.targetvalidation.org/target/ENSG00000135778/associations			No		✓
NTRK3_ext	NT-3 growth factor receptor	TRKC	Q16288			AJ224521							✓
NUAK2	HUMAN NUAK family SNF1-like kinase 2	FLJ90349, SNARK	Q9H093	ENSG0000(	Kinase	NA	Breast carcinoma, Melanoma, Hepatitis C infection, Ovarian neoplasm, Neural tube defect, Glioma, Familial hypercholesterolemia, Cervical carcinoma, Central nervous system cancer, Hypertriglyceridemia	https://www.targetvalidation.org/target/ENSG00000163545/associations	STK11 SMAD4 LATS1 SMAD2 TGFBR1 ACVR1		Yes	https://www.bindingdb.org/uniprot/Q9H093	✓
NUBP2	HUMAN Cytosolic Fe-S cluster assembly factor NUBP2	CFD1	Q9Y5Y2	ENSG0000(	Cancer,Cancer-Antigen	CH471112	Neuroblastoma, Glioma, Melanoma, Inflammation, Chronic obstructive pulmonary disease, Serous adenocarcinoma, IGFBP-3 measurement	https://www.targetvalidation.org/target/ENSG00000095906/associations		Nucleus	No		✓
NUCB1	HUMAN Nucleobindin-1	Calnuc, NUC	Q02818	ENSG0000(	Others	AK315422	Large cell lung carcinoma, Hepatitis, Stevens-Johnson Syndrome, MRSA, Complete atrioventricular block, Liver cirrhosis, Fibrosis, Colorectal cancer, T-cell acute lymphoblastic leukemia,	https://www.targetvalidation.org/target/ENSG00000104805/associations	PTGS2 GNAI3	Golgi apparatus, cis-Golgi network membrane	No		✓
NUDT2	HUMAN Bis(5'-nucleosyl)-tetraphosphatase [asymmetrical]	APAH1	P50583	ENSG0000(	Signalling	CH471071	Psoriasis, Breast carcinoma, Leukemia, Mammary neoplasm, Noninfiltrating intraductal carcinoma, Familial idiopathic cardiomyopathy, Non-Neoplastic disorder, Chronic myelogenous leukemia, Ductal carcinoma in situ, Pustulosis palmaris et plantaris	https://www.targetvalidation.org/target/ENSG00000164978/associations	GAPDH		No		✓

NUF2	HUMAN Kinetochore protein Nuf2	CDCA1, CT106, NUF2R	Q9BZD4	ENSG0000(	Others	AK093348	Lung carcinoma, Large cell lung carcinoma, Melanoma, Hypertensive disease, Non-small cell lung carcinoma, Gastric carcinoma, Prostate carcinoma, Glioma, Azoospermia, Central nervous system cancer	https://www.targetvalidation.org/target/ENSG00000143228/associations	NDC80	Nucleus, Chromosome, centromere, kinetochore,	No		✓
NUP50	HUMAN Nuclear pore complex protein Nup50	NPAP60L	Q9UKX7	ENSG0000(	Others	AK314454	HIV infection, Neural tube closure defect, Isolated spina bifida, Atopic eczema, Psoriasis, Rheumatoid arthritis, Melanoma, Lung carcinoma, Multiple myeloma, Pancreatic ductal adenocarcinoma	https://www.targetvalidation.org/target/ENSG00000093000/associations	CDKN1B MAPK1 MAPK3 CDK1 XPO1 KPNA1 RAN KPNB1 NUP153	Nucleus, nuclear pore complex	No		✓
NXF2	HUMAN Nuclear RNA export factor 2	CT39, TAPL-2, NXF2B	Q9GZY0	ENSG0000(	Others	Z81367	Male infertility with azoospermia or oligozoospermia due to single gene mutation, Normosmic congenital hypogonadotropic hypogonadism, Kallmann syndrome, Non-syndromic male infertility due to sperm motility disorder, Deafness-infertility syndrome, Azoospermia, Cleft palate, Colorectal Carcinoma, Seminoma, Uranostaphyloschisis	https://www.targetvalidation.org/target/ENSG00000269437/associations		Nucleus, nucleoplasm, Cytoplasm,	No		✓
NXF2B	Nuclear RNA export factor 2	CT39, TAPL-2	Q9GZY0	ENSG00000269437-NXI		Z81367	Non-syndromic male infertility due to sperm motility disorder, Male infertility with azoospermia or oligozoospermia due to single gene mutation, Male infertility with teratozoospermia due to single gene mutation, azoospermia, language impairment, Partial chromosome Y deletion, Isolated follicle stimulating hormone deficiency, Isochromosomy Yp, spermatogenic failure 43, Isochromosomy Yq spermatogenic failure 42, Spinocerebellar ataxia type 32, Congenital bilateral absence of vas deferens, spermatogenic failure 44, Male infertility due to large-headed multiflagellar polyploid spermatozoa, schizophrenia, Monoamine oxidase A deficiency, Autosomal recessive non-syndromic sensorineural deafness type DFNB, spermatogenic failure 1, spermatogenic failure 31	https://platform.opentargets.org/target/ENSG00000269437/associations		Cytoplasm, Nucleus			✓
OAS2	HUMAN 2'-5'-oligoadenylate synthase 2	OAS2	P29728	ENSG0000(	Others	AC004551	Psoriasis, Li-Fraumeni syndrome, Lung carcinoma, Major Depressive Disorder, Unipolar Depression, Mental Depression, Tick-Borne Encephalitis, Chronic hepatitis B, Type I diabetes mellitus, Asthma	https://www.targetvalidation.org/target/ENSG00000111335/associations	NOD2	Cytoplasm, Cytoplasm, perinuclear region, Mitochondrion, Nucleus, Microsome, Endoplasmic reticulum,	No		✓
ODC1	HUMAN Ornithine decarboxylase	ODC	P11926	ENSG0000(	Signalling	M33764	Parasitic infection, Leishmaniasis, Trypanosomiasis, Papilloma, Adenoma, Neuroblastoma, HIV Infection, Atrichia with papular lesion, Squamous cell carcinoma, Prostate carcinoma	https://www.targetvalidation.org/target/ENSG00000115758/associations	AR TP53 OAZ1		Yes		✓
												https://www.bindingdb.org/uniprot/P11926	
ODF1	HUMAN Outer dense fiber protein 1	HSPB10, ODFP	Q14990	ENSG0000(	Others	BC104457	Azoospermia, IGA Glomerulonephritis, Chronic lymphocytic leukemia, Rheumatoid arthritis, Diabetes mellitus, Giant cell tumor of bone, Superficial ulcer, Skin erosion, Kallmann syndrome, Teratozoospermia	https://www.targetvalidation.org/target/ENSG00000155087/associations	CDK5R1 KLC1 SPAG5 TRIP6 ODF2 SPAG4		No		✓
ODF2	HUMAN Outer dense fiber protein 2	ODF84, ODFP2	Q5BJF6	ENSG0000(	Others	DQ444714	Azoospermia, Male infertility with teratozoospermia due to single gene mutation, Male infertility with azoospermia or oligozoospermia due to single gene mutation, Non-syndromic male infertility due to sperm motility disorder, Male infertility due to large-headed multiflagellar polyploid spermatozoa, Primary ciliary dyskinesia, Turner syndrome, Pancreatic carcinoma, Prostate carcinoma, Deafness-infertility syndrome	https://www.targetvalidation.org/target/ENSG00000136811/associations	ODF1 RAB8A CDK1 MARK4	Cytoplasm, cytoskeleton, microtubule organizing center, centrosome	No		✓
ODF3	HUMAN Outer dense fiber protein 3	TISP50, hSHIPPO, SHIPPO1	Q96PU9	ENSG0000(	Others	AC069287	Breast carcinoma, Prostate carcinoma, Benign prostatic hyperplasia, Prostate adenocarcinoma, Azoospermia	https://www.targetvalidation.org/target/ENSG00000177947/associations		Cytoplasm	No		✓

OGG1	HUMAN N-glycosylase/DNA lyase	HMMH, HOGG1, MUTM, OGH1	O15527	ENSG0000(	Signalling, Cancer	Y13277	Neurodegenerative disease, Cockayne syndrome, Renal cell carcinoma, Lung neoplasm, Adenocarcinoma, Chromosome aberration, Type II diabetes mellitus, Huntington Disease, Hyperplasia, Lymphoma	https://www.targetvalidation.org/target/ENSG00000114026/associations	PRKCA CAPN1 XRCC1 XPC	Nucleus, nucleoplasm	Yes		✓
												https://www.bindingdb.org/uniprot/O15527	
OIP5	HUMAN Protein Mis18-beta	CT86, hMIS18beta, MIS18B	O43482	ENSG0000(	Others	BC015050	Azoospermia, Lung carcinoma, Melanoma, Glioma, Medulloblastoma, Gastric carcinoma, Obesity, Esophageal neoplasm, Squamous cell carcinoma, Pancreatic adenocarcinoma	https://www.targetvalidation.org/target/ENSG00000104147/associations	BRAF RAF1 TMPO	Nucleus	No		✓
OLIG3	HUMAN Oligodendrocyte transcription factor 3	Bhlhb7, bHLHe20	Q7RTU3	ENSG0000(	Transcription factor	BK000141	Celiac disease, Inflammatory bowel disease, Primary biliary cirrhosis, Multiple sclerosis, Rheumatoid arthritis, Crohn's disease, Type I diabetes mellitus, Type II diabetes mellitus, Psoriasis, Coronary artery disease	https://www.targetvalidation.org/target/ENSG00000177468/associations		Nucleus	No		✓
OSBPL9	HUMAN Oxysterol-binding protein-related protein 9	OSBL9, OSBP4, ORP9	Q96SU4	ENSG0000(	Others	AK027535	Breast carcinoma, Breast adenocarcinoma, Inflammation, Diffuse large B-cell lymphoma, Adams-Oliver syndrome, Familial combined hyperlipidemia, Progressive supernuclear palsy, Diabetes mellitus, Dermatomyositis, Pancreatic ductal adenocarcinoma	https://www.targetvalidation.org/target/ENSG00000117859/associations		Late endosome membrane	No		✓
OTOA	HUMAN Otoancorin	DFNB22, OTOAN	Q7RTW8	ENSG0000(	Others	AC092719	Non-syndromic genetic deafness, Autosomal recessive non-syndromic sensorineural deafness type DFNB, Hearing loss, Deafness, Ovarian carcinoma	https://www.targetvalidation.org/target/ENSG00000155719/associations		Apical cell membrane	No		✓
OVOL1	HUMAN Putative transcription factor Ovo-like 1	HOVO1	O14753	ENSG0000(	Transcription factor	NA	Inflammatory skin disease, Atopic eczema, Pilomatrixoma, Renal cell carcinoma, Azoospermia, Psoriasis, Asthma, Tongue squamous cell carcinoma, Lung adenocarcinoma, Alopecia	https://www.targetvalidation.org/target/ENSG00000172818/associations	HDAC1	Nucleus	No		✓
OXSR1	HUMAN Serine/threonine-protein kinase OSR1	KIAA1101, OSR1	O95747	ENSG0000(	Kinase	BC008726	Corticosteroid-binding globulin deficiency, Hypertension, Congenital analbuminemia, Metachromatic leukodystrophy, Schizophrenia, Psoriasis, Rheumatoid arthritis, Breast carcinoma, Melanoma	https://www.targetvalidation.org/target/ENSG00000172939/associations	WNK1 PAK1 MTOR PAK1 TGFBR1 SMURF1 BMPR1B EEF1D SLC12A6 SLC12A2 SLC12A3 SLC12A1 TSC22D4	Cytoplasm	Yes		✓
												https://www.bindingdb.org/uniprot/O95747	
P3H4	HUMAN Synaptonemal complex protein SC65	LEPREL4, NO55, SC65	Q92791	ENSG0000(	Others	BC001047	Non-small cell lung carcinoma, Ovarian carcinoma, Breast carcinoma, Trypanosoma cruzi seropositivity, PR interval, Membranous glomerulonephritis, Interstitial cystitis, Prostate neoplasm, Chronic interstitial cystitis	https://www.targetvalidation.org/target/ENSG00000141696/associations		Nucleus, nucleolus	No		✓
P4HB	HUMAN Protein disulfide-isomerase	ERBA2L, PDI, PDIA1, PO4DB	P07237	ENSG0000(	Cancer,Cancer-Antigen	AK315631	Cole-Carpenter syndrome, Familial hypercholesterolemia, Inborn errors of metabolism, Cole Carpenter syndrome, Osteoporosis, Mandibular hypoplasia, Acrocephaly, Osteopenia, Osteochondrodysplasias, Muscle hypotonia	https://www.targetvalidation.org/target/ENSG00000185624/associations	CALR IL7R COL1A1 P4HA2 COL1A2 MAPT UBQLN1 P4HA3	Endoplasmic reticulum	Yes		✓
												https://www.bindingdb.org/uniprot/P07237	

PABPC1	HUMAN Polyadenylate-binding protein 1	PAB1, PABP1, PABPC2	P11940	ENSG0000(	Cancer,Cancer-Antigen	BC015958	Primary ovarian insufficiency, Teratozoospermia, Bladder neoplasm, Transitional cell carcinoma, Mammary neoplasm, Primary effusion lymphoma, Prosatate neoplasm, Breast carcinoma, Duchnne muscular dystrophy, Sarcoma	https://www.targetvalidation.org/target/ENSG00000070756/associations	RBM8A PAIP2 MAP3K14 SMG1 UPF1 GSPT2 CARM1 PAN3 PAN2 EIF4A2 NFX1 EIF4G1 EIF4G2 MAPKAPK2 RPL5 RPL4 RPL13 EIF4B RPL7 PARN XPO1 EIF4G3 PXN BRCA1 EIF4G1 SMG1 IRS1 UPF1 ATXN2 EIF4B	Cytoplasm, Nucleus,	Yes	https://www.bindingdb.org/uniprot/P11940	✓
PACSIN3	HUMAN Protein kinase C and casein kinase substrate in neurons protein 3	PACSIN3	Q9UKS6	ENSG0000(	Kinase	AC090589	Autosomal dominant secondary polycythemia, Jeune syndrome, Pyruvate kinase hyperactivity, Hereditary methemoglobinemia, Short rib-polydactyly syndrome (Verma-Naumoff type), LEOPARD syndrome, Cloacal exstrophy, Weaver syndrome, Saldino-Mainzer syndrome, Von Hippel-Lindau disease	https://www.targetvalidation.org/target/ENSG00000165912/associations	FASLG ADAM12 TRPV4	Cytoplasm	No		✓
PAGE1	HUMAN P antigen family member 1	CT16.3, GAGE-9, GAGEB1, PAGE-1	O75459	ENSG0000(	Others	BC004861	Li-Fraumeni syndrome, Azoospermia, Colorectal adenocarcinoma, Ovarian carcinoma, Melanoma, Germ cell tumor, Prostate carcinoma, Glioblastoma multiforme, Multiple myeloma, Acute lymphoblastic leukemia	https://www.targetvalidation.org/target/ENSG00000068985/associations			No		✓
PAGE2B	HUMAN Putative G antigen family E member 3	GAGEE3	Q5JRK9	ENSG0000(	Others	BC130359	Li-Fraumeni syndrome, Colorectal adenocarcinoma, Azoospermia, Breast carcinoma, Glioblastoma multiforme, Diabetes mellitus, Sepsis	https://www.targetvalidation.org/target/ENSG00000238269/associations			No		✓
PAGE5	HUMAN P antigen family member 5	CT16.1, CT16.2, PAGE-5	Q96GU1	ENSG0000(	Others	BC009230	Colorectal adenocarcinoma, Melanoma, Neoplasm metastasis, Malignant neoplasm of testis	https://www.targetvalidation.org/target/ENSG00000158639/associations			No		✓
PAK2	HUMAN Serine/threonine-protein kinase PAK 2	PAK65, PAKgamma	Q13177	ENSG0000(	Kinase,Cancer-Antigen	NA	HIV infection, Neural tube closure defect, Schizophrenia, Isolated spina bifida, Schizophrenia, Proteinuria, Acquired immunodeficiency syndrome, Prostatic neoplasm, Glioblastoma multiforme, Juvenile dermatomyositis	https://www.targetvalidation.org/target/ENSG00000180370/associations	MKNK1 MYLK ARHGEF6 CTTN RAC1 CDC42 PRKAA2 GZMM PPM1F SYK HTT CASP3 PPM1A MAPK1 MAPK3 NCK1 SRC HSPA5 YES1 PRL MYC ABL1 ARHGAP10 CTTN SRC SYN1 RAF1 PXN CASP3 EIF4G1 ABI1 CASP7 JUN MAP2K3 BRAF	Serine/threonine-protein kinase PAK 2, Cytoplasm,	Yes	https://www.bindingdb.org/uniprot/Q13177	✓
PAK4	HUMAN Serine/threonine-protein kinase PAK 4	KIAA1142	O96013	ENSG0000(	Kinase	AK074728	Lung carcinoma, Non-small cell lung carcinoma, Pancreatic Neoplasm, Parkinson's disease, Neoplasm metastasis, Gastric carcinoma, Liver carcinoma, Glioma, Papillary thyroid carcinoma, Cervical carcinoma	https://www.targetvalidation.org/target/ENSG00000130669/associations	PRKD1 CDC42 YWHAG CTNNB1 CTTN STMN2 PXN YWHAZ LIMK1 CDC42 GRB2 CBL BAD KHDRBS1 FGFR2 LUC7L2 RAN ARHGEF2 NFATC4 SSH1 ITGB5 WNK1 DCP1A ZHX1 ITGAV LARP1 ANKS1A DLST GATAD2A	Cytoplasm	Yes	https://www.bindingdb.org/uniprot/O96013	✓
PAK5	HUMAN Serine/threonine-protein kinase PAK 5	PAK7, KIAA1264	Q9P286	ENSG0000(	Kinase,Cancer-Antigen	AL031652, AL135935, AL353612	Cervical carcinoma, Glioma, Psoriasis, Nonorganic psychosis, Parkinson's disease, Neuropathy, Colon carcinoma, Lung carcinoma, Ependymoma, Astrocytoma	https://www.targetvalidation.org/target/ENSG00000101349/associations	RAC1 CDC42 TCF3 GATA1 CDC42 RAC1 CTNND1 BAD FHL3 RNF7 PDLIM7 TUBGCP4 LZTS2	Mitochondrion, Cytoplasm, Nucleus,	Yes	https://www.bindingdb.org/uniprot/Q9P286	✓
PAK6	HUMAN Serine/threonine-protein kinase PAK 6	PAK5	Q9NQU5	ENSG0000(	Kinase	AK290227	Prostate carcinoma, Hepatocellular carcinoma, Parkinson's disease, Epilepsy, Choriocarcinoma, Pneumonia, Colonic neoplasm, Renal cell carcinoma, Clear cell renal carcinoma, Metabolic disease	https://www.targetvalidation.org/target/ENSG00000137843/associations	AURKB MAPK14 RAC1 CDC42 MAP2K6 AR CDC42 RAC1 AR MDM2 CBL ESR1 PACSIN1 NFATC4	Cytoplasm, Nucleus,	Yes	https://www.bindingdb.org/uniprot/Q9NQU5	✓

PANK3	HUMAN Pantothenate kinase 3	FLJ12899	Q9H999	ENSG0000(	Kinase	CH471062	Glioblastoma multiforme, Lung carcinoma, Atopic eczema, Adenocarcinoma, Pancreatic ductal adenocarcinoma, Atopy, Dermatomyositis, Ulcerative colitis, Celiac disease, Hyperplasia	https://www.targetvalidation.org/target/ENSG00000120137/associations		Cytoplasm	Yes	https://www.bindingdb.org/uniprot/Q9H999	✓
PAPSS2	HUMAN Bifunctional 3'-phosphoadenosine 5'-phosphosulfate synthase 2	ATPSK2	O95340	ENSG0000(	Kinase	AF160509	Spondyloepiphyseal dysplasia and spondyloepimetaphyseal dysplasia, Spondyloepimetaphyseal dysplasia (Pakistani type), Ovarian carcinoma, Precocious pubarche, Genu varum, Platyspondyly, Kyphoscoliosis deformity of spine, Hypoplasia of lower limb, Autosomal recessive predisposition, Malignant mesothelioma	https://www.targetvalidation.org/target/ENSG00000198682/associations			No		✓
PASD1	HUMAN Circadian clock protein PASD1	CT63	Q8IV76	ENSG0000(	Transcription factor	AY623425	Li-Fraumeni syndrome, Medulloblastoma, Melanoma, Lymphoma, Multiple myeloma, Diffuse large B-Cell lymphoma, Neoplasm of mature B-cells, Ovarian carcinoma, Chronic lymphocytic leukemia, Hepatocellular carcinoma	https://www.targetvalidation.org/target/ENSG00000166049/associations		Nucleus	No		✓
PATZ1	HUMAN POZ-, AT hook-, and zinc finger-containing protein 1	PATZ, RIAZ, ZBTB19, ZNF278, ZSG	Q9HBE1	ENSG0000(	Transcription factor,Cancer	AF254084	Adenocarcinoma, Lung carcinoma, Sarcoma, Azoospermia, Seminoma, Testicular neoplasm, Anaplastic thyroid carcinoma, Thyroid carcinoma, Round cell carcinoma, Prostate adenocarcinoma	https://www.targetvalidation.org/target/ENSG00000100105/associations	BCL6 PRKAR1A AR TP53 MITF AR RNF4	Nucleus	No		✓
PAX3	HUMAN Paired box protein Pax-3	HUP2, WS1	P23760	ENSG0000(	Transcription factor,Cancer	AC010980	Waardenburg syndrome, Waardenburg syndrome type 1, Craniofacial-deafness-hand syndrome, Alveolar rhabdomyosarcoma, Craniofacial deafness hand syndrome, Klein's syndrome, Deafness, Neural tube defect, Spina bifida, Hirschsprung disease	https://www.targetvalidation.org/target/ENSG00000135903/associations	WWTR1 DACH2 CSNK2A1 TAF1 CIB1 DAXX LEF1 MDM2 DACH2 MSX1 SOX10	Nucleus	No		✓
PAX5	HUMAN Paired box protein Pax-5	BSAP	Q02548	ENSG0000(	Transcription factor,Cancer	AY463954	Hematological system disease, Lymphoid neoplasm, Acute lymphoblastic leukemia, Precursor B-Cell lymphoblastic leukemia-lymphoma, Hodgkin's disease, Multiple myeloma, Leukemogenesis, Lymphoma, Small cell lung carcinoma, Esophageal carcinoma	https://www.targetvalidation.org/target/ENSG00000196092/associations	ID3 ID2 ETS1 MYB PDCD1 CSF1R EP300 CD79A BLK CD19 TLE4 EBF1	Nucleus	No		✓
PAX8	HUMAN Paired box protein Pax-8	PAX8	Q06710	ENSG0000(	Transcription factor,Cancer	S77905	Thyroid disease, Congenital hypothyroidism, Primary congenital hypothyroidism, Thyroid hypoplasia, Thyroid ectopia, Hypothyroidism, Bradycardia, Macroglossia, Icterus, Athyreosis	https://www.targetvalidation.org/target/ENSG00000125618/associations	WWTR1 FOXI1 ID2 SMAD3 NOTCH1 NKX2-1 RB1 WNT1 TGFB1 EP300 WBP2	Nucleus	No		✓
PBX1	HUMAN Pre-B-cell leukemia transcription factor 1	PBX1, PRL	P40424	ENSG0000(	Transcription factor,Cancer	AL359255, AL357568, AL390119	Adenocarcinoma, Lymphoid neoplasm, Burkitt lymphoma, Acute lymphocytic leukemia, Precursor B-Cell lymphoblastic leukemia-lymphoma, Type II diabetes mellitus, Lymphoid leukemia, Melanoma, Tongue squamous cell carcinoma, Pancreatic ductal adenocarcinoma	https://www.targetvalidation.org/target/ENSG00000185630/associations	MYH10 HOXA5 HOXB8 HOXB1 NR3C1 MEIS2 MEIS1 MYOD1 PKNOX1 SESN1 PDX1	Nucleus	No		✓

PBX3	HUMAN Pre-B-cell leukemia transcription factor 3	PBX3	P40426	ENSG0000(	Transcription factor	AK289376	Congenital pulmonary alveolar proteinosis, Primary hyperoxaluria, Familial long QT syndrome, Cleft lip, Cleft palate, Mixed lineage leukemia, Liver carcinoma, Colorectal cancer, Prostate carcinoma, Cytogenetically normal acute myeloid leukemia	https://www.targetvalidation.org/target/ENSG00000167081/associations	HOXB8	Nucleus	No		✓
PC	HUMAN Pyruvate carboxylase, mitochondrial	PCB	P11498	ENSG0000(	Signalling	AK297705	Pyruvate carboxylase deficiency, Breast carcinoma, Clear cell renal carcinoma, Hereditary hyperekplexia, X-linked intellectual disability - epilepsy, Pyruvate carboxylase deficiency disease, Muscle hypotonia, Type II renal tubular acidosis, Hyper-beta-alaninemia, Lactic acidosis	https://www.targetvalidation.org/target/ENSG00000173599/associations		Mitochondrion matrix,	Yes		✓
												https://www.drugbank.ca/biodb/polypeptides/P11498	
PCBD1	Pterin-4-alpha-carbinolamine dehydratase	DCoH, Dimerization cofactor of HNF1, PCD	P61457	ENSG00000166228-PCI		AF082858	Hyperphenylalaninemia, Dehydratase deficiency, type II diabetes mellitus, smoking status measurement, red blood cell distribution width, lymphocyte count	https://platform.opentargets.org/target/ENSG00000166228/associations		Cytoplasm, Nucleus			✓
PCBP2	HUMAN Poly(rC)-binding protein 2	hnRNP-E2, HNRNPE2, HNRPE2	Q15366	ENSG0000(	Cancer,Cancer-Antigen	AK302067	Glioma, Adenocarcinoma, Oral neoplasm, Prostatic neoplasm, Blast phase, Metastatic prostate carcinoma, Rabies, Chronic myeloid leukemia, Melanoma, Liver cirrhosis	https://www.targetvalidation.org/target/ENSG00000197111/associations	STK17A ITCH MAVS SRSF3 CNBP IFNAR2 ARAF DDX58 IFIH1	Nucleus	No		✓
PCGF2	HUMAN Polycomb group RING finger protein 2	MEL-18, RNF110, ZNF144	P35227	ENSG0000(	Transcription factor	CH471152	Autosomal recessive spondylocostal dysostosis, Spondyloepiphyseal dysplasia tarda, Thanatophoric dysplasia, Smith-McCort dysplasia, Mucopolysaccharidosis type 4, Breast carcinoma, Breast neoplasm, Prostate carcinoma, Gastric carcinoma, Mucopolysaccharidosis type 4	https://www.targetvalidation.org/target/ENSG00000277258/associations	RYBP RING1 PHC1 CCND2 E2F6 RNF2 UBE2I HSF2 PHC2	Nucleus	No		✓
PCK2	HUMAN Phosphoenolpyruvate carboxykinase [GTP], mitochondrial	PEPCK, PEPCK2	Q16822	ENSG0000(	Kinase,Signalling	AL136295	Clear cell renal carcinoma, Adenocarcinoma, Steatohepatitis, Phosphoenolpyruvate carboxykinase deficiency, Hypoxia, Renal steatosis, Liver Failure, Hypoglycemia, Impaired gluconeogenesis, Autosomal recessive predisposition	https://www.targetvalidation.org/target/ENSG00000100889/associations		Mitochondrion,	No		✓
PCLAF	HUMAN PCNA-associated factor	NS5ATP9, OEATC-1, p15(PAF), PAF15, KIAA0101	Q15004	ENSG0000(	Cancer,Cancer-Antigen	JN245883	Liver carcinoma, Thyroid neoplasm, Psoriasis, Kaposi sarcoma, Dermatologic disorder, Breast carcinoma, Pancreatic carcinoma, Melanoma, Non-small cell lung carcinoma, Dyskeratosis congenita	https://www.targetvalidation.org/target/ENSG00000166803/associations	PCNA	Nucleus	No		✓
PDCD2L	HUMAN Programmed cell death protein 2-like	MGC13096	Q9BRP1	ENSG0000(	Transcription factor,Cancer	AC092073	Breast carcinoma, Colonic neoplasm, Colorectal adenocarcinoma, Medulloblastoma, Squamous cell carcinoma, Ovarian carcinoma, Sjogren's syndrome, Non-small cell lung carcinoma, Acute myeloid leukemia, Hepatocellular carcinoma	https://www.targetvalidation.org/target/ENSG00000126249/associations			No		✓
PDCD5	HUMAN Programmed cell death protein 5	MGC9294, TFAR19	O14737	ENSG0000(	Others	CR456934	Endometrial carcinoma, Lung carcinoma, Chronic myeloid leukemia, Gastric neoplasm, Rheumatoid arthritis, Astrocytoma, Degenerative polyarthritis, Chondrosarcoma, Endometrial carcinoma, Glioma	https://www.targetvalidation.org/target/ENSG00000105185/associations	CSNK2A1 KAT5		No		✓

PDCD6	HUMAN Programmed cell death protein 6	ALG-2, PEF1B	O75340	ENSG0000(	Others	BT020072	Melanoma, Ovarian carcinoma, Gastric carcinoma, Endogenous depression, Colon carcinoma, Pancreatic carcinoma, Sjogren's syndrome, Uterine fibrosis, Endometriosis, Autism	https://www.targetvalidation.org/target/ENSG00000249915/associations	MAP3K5 FAS SEC31A DAPK1 KDR PDCD6IP CHERP PEF1	Endoplasmic reticulum membrane		No		✓
PDCL3	HUMAN Phosducin-like protein 3	VIAF1	Q9H2J4	ENSG0000(	Others	AC068538	Self-reported educational attainment, Breast carcinoma, Melanoma, Alzheimer's disease, Glioblastoma multiforme	https://www.targetvalidation.org/target/ENSG00000115539/associations		Cytoplasm		Yes	https://www.bindingdb.org/uniprot/Q9H2J4	✓
PDE4A	HUMAN cAMP-specific 3',5'-cyclic phosphodiesterase 4A	DPDE2, PDE46	P27815	ENSG0000(	Signalling	U18088	Immune system disease, Psoriasis, Respiratory system disease, Bipolar disorder, Asthma, Schizophrenia, Chronic lymphocytic leukemia, Chronic obstructive pulmonary disease, Atopic eczema, Irritable bowel syndrome	https://www.targetvalidation.org/target/ENSG00000065989/associations	PRKACA LYN SQSTM1	Cytoplasm, perinuclear region, Cell projection, ruffle membrane, Membrane, Peripheral membrane protein,		Yes	https://www.bindingdb.org/uniprot/P27815	✓
PDGFB	HUMAN Platelet-derived growth factor subunit B	SIS, SSV	P01127	ENSG0000(	Kinase,Cancer, Cytokine/Chemokine	M12783	Neurodegenerative disease, Bilateral striopallidodentate calcinosis, Fahr's syndrome, Seizures, Dermatofibrosarcoma protuberans, Meningioma, Fibrosarcoma, Skin neoplasm, Epilepsy, Crohn's disease	https://www.targetvalidation.org/target/ENSG00000100311/associations	EGR1 PDGFRA PDGFRB SPARC PDGFA A2M NRP1 PDGFRB COL1A1 HRAS THBS1 PDGFA HNRNPC PDGFRA ART1	Secreted		Yes	https://www.bindingdb.org/uniprot/P01127	✓
PDGFRA_e	HUMAN Platelet-derived growth factor receptor alpha	CD140a, GAS9, PDGFR2	P16234	ENSG0000(	Cancer	AK316579	Gastrointestinal stromal tumor, Hypereosinophilic syndrome, Eosinophilia, Myeloproliferative disease, Chronic eosinophilic leukemia, Spina bifida, Medulloblastoma, Sarcoma, Gastric neoplasm, Breast neoplasm	https://www.targetvalidation.org/target/ENSG00000134853/associations	PDGFC FOXO1 CAV1 FOXO4 GRB2 JAK1 CBL PDGFRB PDGFA PDGFB EGFR FOXO3 CAV1 EGFR SRC SLC9A3R1 PLCG1 STAT5A JAK1 CRKL SHC1 PDGFRB PDGFB STAT1 CRK GRB14 PTPN11 PDGFA RAPGEF1 PIK3R1	Cell membrane	Transmembrane	Yes	https://www.bindingdb.org/uniprot/P16234	✓
PDGFRB	HUMAN Platelet-derived growth factor receptor beta	CD140b, JTK12, PDGFR, PDGFR1	P09619	ENSG0000(	Kinase,Cancer	AC005895	Infantile myofibromatosis, Penttinen-Aula syndrome, Myeloproliferative disease, Eosinophilia, Chronic myelomonocytic leukemia, Schizophrenia, Psychotic disorder, Schizoaffective disorder, Seizures, Glioblastoma multiforme	https://www.targetvalidation.org/target/ENSG00000113721/associations	ERRFI1 PDGFC PDGFD PRKG1 GAB1 PTPRJ TNK2 PTPN11 PTK2 CAV1 GRB2 PTEN DUSP3 ABL2 FLT3 GRK2 ACP1 JAK1 CBL S1PR1 EIF2AK2 PLCG1 PTPN1 PTPN2 NCK1 PDGFRA SRC KIT YES1 CSF1R FYN PDGFB	Cell membrane, Single-pass type I membrane protein, Cytoplasmic vesicle, Lysosome lumen,	Transmembrane	Yes	https://www.bindingdb.org/uniprot/P09619	✓
PDGFRB_e	HUMAN Platelet-derived growth factor receptor beta	PDGFR, PDGFR1	P09619			AC005895								✓
PDGFRL	HUMAN Platelet-derived growth factor receptor-like protein	PRLTS	Q15198	ENSG0000(	Kinase	CH471080	Colorectal adenocarcinoma, Colorectal neoplasm, Liver carcinoma, Colorectal Cancer, Micronodular cirrhosis, Subacute progressive viral hepatitis, Malignant peripheral nerve sheath tumor, Squamous cell carcinoma, Li-Fraumeni syndrome, Malignant mesothelioma	https://www.targetvalidation.org/target/ENSG00000104213/associations		Secreted		No		✓
PDIK1L	HUMAN Serine/threonine-protein kinase PDIK1L	CLIK1L	Q8N165	ENSG0000(	Kinase	AL391650	Breast carcinoma, Lung carcinoma, Endometriosis, Ovarian carcinoma, Schizophrenia, Prostate carcinoma, Colorectal carcinoma, Adenomatous polyposis coli, Esophageal carcinoma, Mucocutaneous lymph node syndrome	https://www.targetvalidation.org/target/ENSG00000175087/associations	NEUROD1 TNF HCLS1 ZHX1	Nucleus		No		✓

PDK1	HUMAN [Pyruvate dehydrogenase (acetyl-transferring)] kinase isozyme 1, mitochondrial	PDK1	Q15118	ENSG0000(	Kinase	AK312700	Clear cell renal carcinoma, Lung carcinoma, Glioma, Altitude sickness, Rectal neoplasm, Type II diabetes mellitus, Melanoma, Breast carcinoma, Glioblastoma multiforme, Osteosarcoma	https://www.targetvalidation.org/target/ENSG00000152256/associations	AKT3 PKN2 PDK2 USP4 PRKCD PRKCZ FLT3 AKT2 AKT1 PRKCA SRC FGFR1 ABL1 JAK2 NOS3 PRKCA PRKCE PTPN1 CDKN1B RAF1 IRAK1 MAP2K2 PRKCZ CASP9 FOXO1 PRKCD YAP1 GSK3A PRKCG BRCA1 EP300	Mitochondrion matrix	Yes	https://www.bindingdb.org/uniprot/Q15118	✓
PDK2	HUMAN [Pyruvate dehydrogenase (acetyl-transferring)] kinase isozyme 2, mitochondrial	PDHK2	Q15119	ENSG0000(	Kinase,Signalling	AC002401	Breast carcinoma, Inborn errors of metabolism, Pyruvate dehydrogenase deficiency, Cardiomyopathy, Type II diabetes mellitus, Obesity, Gout, Melanoma, Osteosarcoma, Ulcerative colitis	https://www.targetvalidation.org/target/ENSG00000005882/associations	PDK1 AKT1 SGK1 AKT1 SGK1 PDK1 HTT PDHA1 PDHA2 HtC2 DLAT PDHX ISOC2	Mitochondrion matrix,	Yes	https://www.bindingdb.org/uniprot/Q15119	✓
PDK3	HUMAN [Pyruvate dehydrogenase (acetyl-transferring)] kinase isozyme 3, mitochondrial	PDHK3	Q15120	ENSG0000(	Kinase	BC015948	Charcot-Marie-Tooth disease, X-linked Charcot-Marie-Tooth disease type 6, Breast carcinoma, Glioblastoma, Peripheral neuropathy, Metastatic melanoma, Pyruvate dehydrogenase deficiency, Type II diabetes mellitus, Obesity, Gout, Melanoma, adenocarcinoma, Renal cell carcinoma, Pancreatic adenocarcinoma	https://www.targetvalidation.org/target/ENSG00000067992/associations	PDHA1 PDHA2 DLAT PAXBP1	Mitochondrion matrix,	Yes	https://www.bindingdb.org/uniprot/Q15120	✓
PDK4	HUMAN [Pyruvate dehydrogenase (acetyl-transferring)] kinase isozyme 4, mitochondrial	PDK4	Q16654	ENSG0000(	Kinase	BC040239	Li-Fraumeni syndrome, Lung carcinoma, Myocardial ischemia, Obesity, Type II diabetes mellitus, Liver carcinoma, Van der Woude syndrome, Renal carnitine transport defect, Hypertriglyceridemia, Teratozoospermia	https://www.targetvalidation.org/target/ENSG00000004799/associations	PLEK PDHA1 PDHA2 DLAT	Mitochondrion matrix,	Yes	https://www.bindingdb.org/uniprot/Q16654	✓
PDPK1	HUMAN 3-phosphoinositide-dependent protein kinase 1	PDK1	O15530	ENSG0000(	Kinase	AB209835	Familial hyperinsulinism, Congenital isolated hyperinsulinism, Mammary neoplasm, Diffuse large B-Cell lymphoma, Heart failure, Rhabdomyosarcoma, Thyroid neoplasm, Papillary adenocarcinoma, Breast carcinoma, Colon carcinoma	https://www.targetvalidation.org/target/ENSG00000140992/associations	AKT3 RPS6KB2 CARD11 MAP3K5 PKN2 PKN1 RPS6KA1 RPS6KA2 PDK1 MELK PTK2B ILK PRKCZ PRKCQ PPP2R2B PTEN RPS6KA3 PIK3CG MTOR PRKCI AKT2 AKT1 PIK3R1 PRKCH RPS6KB1 SRC IGF1R RET HSP90AA1 INSR	Cytoplasm, Nucleus, Cell membrane, Peripheral membrane protein, Cell junction, focal adhesion,	Yes	https://www.bindingdb.org/uniprot/O15530	✓
PDXK	HUMAN Pyridoxal kinase	C21orf124, C21orf97, PKH, PNK, PRED79	O00764	ENSG0000(	Kinase	BC000123	Neoplasm, Azoospermia, Lung carcinoma, Teratozoospermia, Breast carcinoma, Malignant peripheral nerve sheath tumor, Lung adenocarcinoma, Ovarian carcinoma	https://www.targetvalidation.org/target/ENSG00000160209/associations		Cytoplasm,	Yes	https://www.bindingdb.org/uniprot/O00764	✓
PEBP1	HUMAN Phosphatidylethanolamine-binding protein 1	HCNP, PBP, PEBP, RKIP	P30086	ENSG0000(	Others	AK311927	Breast carcinoma, Kallmann syndrome, Heart failure, Renal cell carcinoma, Myocardial ischemia, Precancerous condition, Neoplasm metastasis, Colorectal neoplasm, Schizophrenia, Melanoma	https://www.targetvalidation.org/target/ENSG00000089220/associations	MAP3K14 PRKCD PRKCZ MAP2K1 MAPK1 GRK2 PRKCA IL7R PRKCB PRKCG RAF1 MAP3K7 CHUK IKBKB MAP3K7 RAF1 MAP2K1 BRAF IKBKB CHUK MAP4K4	Cytoplasm	No		✓
PELO	HUMAN Protein pelota homolog	PELO	Q9BRX2	ENSG0000(	Cancer,Cancer-Antigen	AF132951	Glioblastoma multiforme, Lung carcinoma, Ovarian carcinoma, Pancreatic ductal adenocarcinoma, Neoplasm metastasis, Meningococcal infection, Pulmonary tuberculosis, Melanoma, Mental Depression, Melanoma, Neuroblastoma, Dermatomyositis	https://www.targetvalidation.org/target/ENSG00000152684/associations		Nucleus	No		✓
PFKFB3	HUMAN 6-phosphofructo-2-kinase/fructose-2,6-bisphosphatase 3	F263	Q16875	ENSG0000(	Kinase	AF041831	Rheumatoid arthritis, Obesity, Unipolar depression, Dermatologic disorder, Myocardial ischemia, Mental Depression, Melanoma, Bipolar Disorder, Atrocytoma, Glioma	https://www.targetvalidation.org/target/ENSG00000170525/associations	PRKAA1 CSNK2A1 PRKAA2 AKT1 PRKACA PRKCA		Yes	https://www.bindingdb.org/uniprot/Q16875	✓

PFKFB4	HUMAN 6-phosphofructo-2-kinase/fructose-2,6-bisphosphatase 4	F264	Q16877	ENSG0000(	Kinase	AY714243	Central nervous system cancer, Glioma, Glioblastoma multiforme, Anoxia, Pancreatic Neoplasm, Gastric carcinoma, Medulloblastoma, Waardenburg syndrome, Postaxial acrofacial dysostosis, Breast carcinoma	https://www.targetvalidation.org/target/ENSG00000114268/associations			Yes	https://www.bindingdb.org/uniprot/Q16877	✓
PFKP	HUMAN ATP-dependent 6-phosphofructokinase, platelet type	PFK-C, PFKF	Q01813	ENSG0000(	Kinase	AL731533, AL451164	Azoospermia, Renal carcinoma, Lumbar disc degeneration, Obesity, Amyotrophic lateral sclerosis, Breast carcinoma, Renal tubular acidosis, Pancreatic carcinoma, Lung carcinoma, Ovarian carcinoma, Psoriasis	https://www.targetvalidation.org/target/ENSG00000067057/associations	PFKM PFKM	Cytoplasm	No		✓
PFN2	HUMAN Profilin-2	PROF2	P35080	ENSG0000(	Cancer,Cancer-Antigen	AK311782	Lung carcinoma, Astrocytoma, Glioblastoma multiforme, Amyotrophic lateral sclerosis, Breast carcinoma, Gastric neoplasm, Atopy, Ulcerative colitis, Crohn's disease, Psoriasis	https://www.targetvalidation.org/target/ENSG00000070087/associations	ROCK1 TRIM24 DNM1 ACTB VASP ACTG1	Cytoplasm, cytoskeleton,	Yes	https://www.drugbank.ca/biodb/polypeptides/P35080	✓
PHB2	HUMAN Prohibitin-2	Bap37, BCAP37, p22, REA	Q99623	ENSG0000(	Others	AF126021	Breast carcinoma, Hepatitis C infection, Autism spectrum disorder, Spinal tuberculosis, Pulmonary tuberculosis, Gastrointestinal carcinoid tumor, Rhabdomyosarcoma, Ulcerative colitis, hepatocellular carcinoma, Colorectal adenocarcinoma	https://www.targetvalidation.org/target/ENSG00000215021/associations	AURKB CAMK1D ARFGEF3 CAMK4 DDR1 CXCR4 BMPR1B TRPM6 ESR1	Mitochondrion inner membrane	No		✓
PHF1	HUMAN PHD finger protein 1	MTF2L2, TDRD19C	O43189	ENSG0000(	Transcription factor	AL662799	Sarcoma, Endometrial stromal sarcoma, Ossifying fibromyxoid tumor, Alzheimer's disease, Systemic lupus erythematosus, Scrapie, Sepsis, Uterine sarcoma, Uterine fibroid, Tauopathy	https://www.targetvalidation.org/target/ENSG00000112511/associations	SUZ12 EZH2	Nucleus, Cytoplasm, cytoskeleton, microtubule organizing center, centrosome,	Yes	https://www.bindingdb.org/uniprot/O43189	✓
PHF11	HUMAN PHD finger protein 11	BCAP, IGER, NY-REN-34	Q9UIL8	ENSG0000(	Cancer,Cancer-Antigen	AL139321	LCAT deficiency, Familial partial lipodystrophy, Coronary artery disease-hyperlipidemia-hypertension-diabetes-osteoporosis, Familial hemophagocytic lymphohistiocytosis, Asthma, Eczema, Atopic dermatitis, Bronchial hyperreactivity, Respiratory syncytial virus infection, Immediate hypersensitivity	https://www.targetvalidation.org/target/ENSG00000136147/associations	RELA PIAS4	Nucleus	No		✓
PHF5A	HUMAN PHD finger-like domain-containing protein 5A	bK223H9.2, INI, MGC1346, Rds3, SAP14b, SF3b14b, SF3B7	Q7RTV0	ENSG0000(	Transcription factor	BC075808	Breast carcinoma, Glioblastoma multiforme, Pancreatic ductal adenocarcinoma, Amyotrophic lateral sclerosis, Brain neoplasm, Medulloblastoma, Schizophrenia, Rhabdoid tumor, Primitive neuroectodermal tumor, Autosomal dominant retinitis pigmentosa	https://www.targetvalidation.org/target/ENSG00000100410/associations		Nucleus	No		✓
PHF7	HUMAN PHD finger protein 7	HSPC226, NYD-SP6	Q9BWX1	ENSG0000(	Cancer,Cancer-Antigen	CH471055	Azoospermia, Osteosarcoma, Breast carcinoma, Teratozoospermia, Colorectal adenocarcinoma	https://www.targetvalidation.org/target/ENSG00000010318/associations	IKBKG	Nucleus	No		✓
PHIP	HUMAN PH-interacting protein	BRWD2, DCAF14, FLJ20705, ndrp, WDR11	Q8WWQ0	ENSG0000(	Cancer,Cancer-Antigen	AL356776, AL450327	Lung carcinoma, Central nervous system cancer, Glioblastoma multiforme, Sjogren's Syndrome, Melanoma, Ulcer, Neoplasm metastasis, Ovarian carcinoma, Pancreatic carcinoma, Obesity	https://www.targetvalidation.org/target/ENSG00000146247/associations	IRS1	Nucleus	No		✓

PHKG1	HUMAN Phosphorylase b kinase gamma catalytic chain, skeletal muscle/heart isoform	PHKG	Q16816	ENSG0000(	Kinase,Cancer,Cancer-Antigen	AC092101	Breast carcinoma, Li-Fraumeni syndrome, Glycogen storage disease (Type IXD), Glioma, Astrocytoma, Lung carcinoma, Dermatomyositis, Fibroadenoma, Kabuki syndrome, Duchenne muscular dystrophy	https://www.targetvalidation.org/target/ENSG00000164776/associations	MAPK6 PHKG2 CALM1 MAPT PYGB PYGM PHKA1 PHKG2 CTRC		Yes	https://www.bindingdb.org/uniprot/Q16816	✓
PHKG2	HUMAN Phosphorylase b kinase gamma catalytic chain, liver/testis isoform	PHKG2	P15735	ENSG0000(	Kinase	AK293551	Glycogen storage disease IXC, Glycogen storage disease due to phosphorylase kinase deficiency, Glycogen storage disease due to liver phosphorylase kinase deficiency, Liver Cirrhosis, Lactic acidosis, MODY, Atopic eczema, Diffuse large B-cell lymphoma, Breast carcinoma, Fibrosis	https://www.targetvalidation.org/target/ENSG00000156873/associations	PHKG1 MAPT PYGB PYGM PHKG1 PHKA1		Yes	https://www.bindingdb.org/uniprot/P15735	✓
PHLDA1	HUMAN Pleckstrin homology-like domain family A member 1	PHRIP, TDAG51	Q8WV24	ENSG0000(	Cancer	BC110820	Central nervous system cancer, Melanoma, Epilepsy, Breast carcinoma, Osteosarcoma, Colon carcinoma, Colorectal cancer, Cervical carcinoma, Alpha-Thalassemia, Squamous cell carcinoma	https://www.targetvalidation.org/target/ENSG00000139289/associations	AURKA	Cytoplasm	No		✓
PHLDA2	HUMAN Pleckstrin homology-like domain family A member 2	BWR1C, HLDA2, IPL, TSSC3	Q53GA4	ENSG0000(	Cancer,Cancer-Antigen	CR407664	Squamous cell carcinoma, Lung carcinoma, Osteosarcoma, Anoxia, Premature infant, Autosomal dominant optic atrophy, Brain neoplasm, Non-small cell lung carcinoma, Adenocarcinoma, Ovarian carcinoma	https://www.targetvalidation.org/target/ENSG00000181649/associations		Cytoplasm	No		✓
PHTF1	HUMAN Putative homeodomain transcription factor 1	PHTF	Q9UMS5	ENSG0000(	Transcription factor	AL365321, AL133517	Autoimmune disease, Type I diabetes mellitus, Rheumatoid arthritis, Systemic lupus erythematosus, Crohn's disease, Psoriasis, Ankylosing spondylitis, Hypothyroidism, Ulcerative colitis, Celiac disease	https://www.targetvalidation.org/target/ENSG00000116793/associations		Nucleus	No		✓
PI42B	HUMAN Phosphatidylinositol 5-phosphate 4-kinase type-2 beta	PIP5K2B	P78356	ENSG0000(	Kinase,Cancer	NA	Breast carcinoma, Diabetes mellitus, Obesity, Alzheimer's disease, Neoplasm metastasis	https://www.targetvalidation.org/target/ENSG00000276293/associations	MAPK14 BTK TNFRSF1A SPOP	Endoplasmic reticulum membrane	Yes	https://www.bindingdb.org/uniprot/P78356	✓
PIAS2	HUMAN SUMO-protein ligase PIAS2	PIASX	O75928	ENSG0000(	Signalling	AC090241	Azoospermia, Familial exudative vitreoretinopathy, Mucopolysaccharidosis type 4, Achondrogenesis, Asperger syndrome, Central neuroblastoma, T-Cell lymphoma, Colon carcinoma, Diffuse large B-Cell lymphoma, Renal cell carcinoma	https://www.targetvalidation.org/target/ENSG00000078043/associations	SUMO1 AKT1 SRF AR MYC RACK1 SMAD4 STAT1 HIC1 PPARG AR STAT4 SUMO1 UXT ELK1 RUFY1 PARK7 CDKN2B	Nucleus speckle	No		✓
PIDD1	HUMAN p53-induced death domain-containing protein 1	LRDD, PIDD	Q9HB75	ENSG0000(	Signalling	AK074893	Breast carcinoma, Hepatocellular carcinoma, Primary immunodeficiency, Melanoma, Liver carcinoma, Renal cell carcinoma, Squamous cell carcinoma, Osteocarcinoma, Ovarian carcinoma, Acute promyelocytic leukemia	https://www.targetvalidation.org/target/ENSG00000177595/associations	TP53 CRADD PRKDC MADD IKBKG CASP2 FADD RIPK1	Cytoplasm	No		✓
PIK3C3	HUMAN Phosphatidylinositol 3-kinase catalytic subunit type 3	Vps34	Q8NEB9	ENSG0000(	Kinase,Signalling	Z46973	Obesity, Glioblastoma multiforme, Schizophrenia, Systemic lupus erythematosus, Esophageal neoplasm, Breast carcinoma, Ovarian carcinoma, Prostate carcinoma, Periodontitis	https://www.targetvalidation.org/target/ENSG00000078142/associations	SH3GLB1 UVRAG PIK3R4 CAMKK2 NRBF2 RUBCN ATG14 BECN1 CDK5 CALM1 CDK1 PTEN AKT1 PIK3R4 RPTOR MDM4 SRSF10 ANKS1A SRSF4	Midbody	Yes	https://www.bindingdb.org/uniprot/Q8NEB9	✓

PIK3R1	HUMAN Phosphatidylinositol 3-kinase regulatory subunit alpha	GRB1, p85, p85A	P27986	ENSG0000(	Kinase,Signalling,Cancer	AK094785	SHORT syndrome, Primary immunodeficiency, Primary immunodeficiency due to a defect in adaptive immunity, Lipodystrophy, Lipoatrophy, Rieger syndrome, Hyperglycemia, Agammaglobulinemia, Exanthema, Hypogammaglobulinemia	https://www.targetvalidation.org/target/ENSG00000145675/associations	ICOS IRS2 GAB2 WASF3 VAV3 IL1RAP MYD88 DOK1 AGAP2 INPP5D DKFZp686 L02273 PIK3AP1 TRAT1 SHB ERBB4 PTK2B GAB1 CBLB LCP2 DLG1 PTPRJ SOS2 SOS1 KHDRBS1 TYRO3 PTPN11 PRKCZ PTK2 DNM1		Yes	https://www.bindingdb.org/uniprot/P27986	✓
PIM1	HUMAN Serine/threonine-protein kinase pim-1	PIM	P11309	ENSG0000(	Kinase,Cancer	M24779	Lymphoid neoplasm, Neoplasm of mature B-cells, Prostatic Intraepithelial Neoplasia, Pyelonephritis, Glioblastoma, Congenital diaphragmatic hernia, Prostate carcinoma, B-Cell Lymphoma, Bladder carcinoma, Colonic neoplasm	https://www.targetvalidation.org/target/ENSG00000137193/associations	SND1 STAT3 ABCB1 CTTN CDKN1B HSP90AA1 RUVBL2 CDC25A NUMA1 RELA SKP2 MAP3K5 EIF2AK2 CBX1 SPI1 NKX3-1 CBX3 EIF4B RUNX3 CDKN1A AR FOXO3 CXCR4 MDM2 BAD AKT1S1 HIST1H3A; HIST1H3B; HIST1H3C; HIST1H3D; HIST1H3E;	Cytoplasm, Nucleus, Cell membrane,	Yes	https://www.bindingdb.org/uniprot/P11309	✓
PIM2	HUMAN Serine/threonine-protein kinase pim-2	PIM2	Q9P1W9	ENSG0000(	Kinase,Cancer	AF207550	Breast carcinoma, Leukemia, Acute myeloid leukemia, Multiple myeloma, Prostate carcinoma, Benign prostatic hyperplasia, Chronic lymphocytic leukemia, Periodontitis, Osteosarcoma, Melanoma	https://www.targetvalidation.org/target/ENSG00000102096/associations	IKBKG RPS6KA2 CRK SOCS1 CDKN1A PKM BAD EIF4ENIF1 ZYX ATXN1 ZHX1 URI1 NDUFB8 HNRNPH3 CLASRP FBXO18 RPL21 CUTC PUM1 R3HDM2 PAXBP1		Yes	https://www.bindingdb.org/uniprot/Q9P1W9	✓
PIN1	HUMAN Peptidyl-prolyl cis-trans isomerase NIMA-interacting 1	dod	Q13526	ENSG0000(	Cancer	AK291074	Normosmic congenital hypogonadotropic hypogonadism, 46-XX testicular disorder of sex development, Male infertility with azoospermia or oligozoospermia due to single gene mutation, Alzheimer's disease, Asthma, Secondary hyperparathyroidism, Squamous cell carcinoma, Breast carcinoma, Liver carcinoma, Hepatocellular carcinoma	https://www.targetvalidation.org/target/ENSG00000127445/associations	DAXX PKMYT1 AURKB MAP3K11 ELAVL1 STIL IRF3 NFATC2 SP140 MCL1 POU5F1 MYT1 FOXO4 SMAD3 CSNK2A1 TERF1 DAPK1 PLK1 PRKX CDK9 GSK3B MAP3K8 STAT3 SNCA PPARG IRS1 CTNNB1 CDC25C PML SHC1 POLR2A	Nucleus	Yes	https://www.bindingdb.org/uniprot/Q13526	✓
PIP	HUMAN Prolactin-inducible protein	GCDFP-15, GCDFP15, GPIP4	P12273	ENSG0000(	Others	AK312227	Breast carcinoma, Psoriasis, Sinusitis, Allergic rhinitis, Cystic disease, Breast fibrocystic disease, Teratozoospermia, Rosacea, Dermatomyositis, Glioma	https://www.targetvalidation.org/target/ENSG00000159763/associations	CD4 SPI1	Secreted	No		✓
PIP4K2C	HUMAN Phosphatidylinositol 5-phosphate 4-kinase type-2 gamma	PIP5K2C	Q8TBX8	ENSG0000(	Kinase	AK315588	Psoriasis, Nicotine dependence, Sarcoma, Schizophrenia, Bipolar disorder, Rheumatoid arthritis, Tardive dyskinesia	https://www.targetvalidation.org/target/ENSG00000166908/associations	BTK	Cytoplasm, Membrane,	Yes	https://www.bindingdb.org/uniprot/Q8TBX8	✓
PIP5K1B	HUMAN Phosphatidylinositol 4-phosphate 5-kinase type-1 beta	STM7, PI51B	O14986	ENSG0000(	Kinase	AK295587	Chronic kidney disease, Pancreatic carcinoma, Pancreatic adenocarcinoma, Chronic kidney failure, Schizophrenia, Friedreich ataxia, Asthma, Ependymoma, Medulloblastoma, Pancreatic carcinoma	https://www.targetvalidation.org/target/ENSG0000007242/associations	BTK SYK PRKCA	Endomembrane system	No		✓
PIR	HUMAN Pirin	PIR	O00625	ENSG0000(	Transcription factor	BT019583	Breast carcinoma, Melanoma, Lung carcinoma, Contact dermatitis, Acute mtelocytic leukemia, Osteosarcoma, Polycystic ovary syndrome, Adams-Oliver syndrome, Glioblastoma multiforme, Fibrosis	https://www.targetvalidation.org/target/ENSG0000087842/associations	BCL3 NFIX	Nucleus	Yes	https://www.bindingdb.org/uniprot/O00625	✓
PITRM1	HUMAN Presequence protease, mitochondrial	hMP1, KIAA1104, MP1	Q5JRX3	ENSG0000(	Cancer,Cancer-Antigen	AL451164	Subarachnoid hemorrhage, Cerebral hemorrhage, Kidney disease, Cerebrovascular accident, Intracranial hemorrhage, Amyloid plaque, Progressive cerebellar ataxia, Nonorganic psychosis, Alzheimer's disease, Psychotic disorder	https://www.targetvalidation.org/target/ENSG00000107959/associations		Mitochondrion matrix	Yes	https://www.bindingdb.org/uniprot/Q5JRX3	✓

PITX2	HUMAN Pituitary homeobox 2	ARP1, RGS, RIEG, RIEG1	Q99697	ENSG0000(	Transcription factor	AF048722	Iridogoniodysgenesis, Axenfeld-Rieger syndrome, Rieger anomaly, Glaucoma, Iris hypoplasia, Axenfeld anomaly, Atrial fibrillation, Peters anomaly, Central nervous system cancer, Cardic arrhythmia	https://www.targetvalidation.org/target/ENSG00000164093/associations	LEF1 KAT5 YBX1 CTNNB1 PROP1 LEF1 KAT5	Nucleus,	No		✓
PIWIL2	HUMAN Piwi-like protein 2	CT80, HILI	Q8TC59	ENSG0000(	Others	AK027497	Male infertility with azoospermia or oligozoospermia due to single gene mutation, Li-Fraumeni syndrome, Male infertility with teratozoospermia due to single gene mutation, Kallmann syndrome, Azoospermia, Bladder neoplasm, Young syndrome, Partial androgen insensitivity syndrome, Dyskeratosis congenita, Deafness-infertility syndrome	https://www.targetvalidation.org/target/ENSG00000197181/associations	DDX4	Cytoplasm	No		✓
PKDCC	HUMAN Extracellular tyrosine-protein kinase PKDCC	SgK493, Vlk	Q504Y2	ENSG0000(	Kinase	BC007901	Hypospadias, Acromesomelic dysplasia, Osteogenesis imperfecta, Osteogenesis imperfecta type 3, Atopy, Lung adenocarcinoma, Colorectal adenocarcinoma, Atelosteogenesis type I, Madelung deformity, Brachydactyly type A6	https://www.targetvalidation.org/target/ENSG00000162878/associations		Secreted	No		✓
PKLR	HUMAN Pyruvate kinase PKLR	KPYR, PK1, PKL	P30613	ENSG0000(	Kinase,Signalling	AY316591	Inborn errors of metabolism, Hemolytic anemia due to red cell pyruvate kinase deficiency, Pyruvate kinase hyperactivity, Icterus, Chronic hemolytic anemia, Unconjugated hyperbilirubinemia, Reticulocytosis, Abnormal amniotic fluid, Osteosarcoma, Sarcoma	https://www.targetvalidation.org/target/ENSG00000143627/associations			Yes	https://www.bindingdb.org/uniprot/P30613	✓
PKM	HUMAN Pyruvate kinase PKM	OIP3, PK2, PK3, PKM2, THBP1	P14618	ENSG0000(	Kinase,Signalling,Cancer	AK092369	Digestive system disease, Hepatocellular carcinoma, Osteoporosis, Squamous cell carcinoma, Lung neoplasm, Glioma, Astrocytoma, Clear cell renal carcinoma, Sepsis, Gastric carcinoma	https://www.targetvalidation.org/target/ENSG00000067225/associations	PIM2 SCYL2 HIF1A DAPK1 MAPK8 MAPK1 FGFR2 CDK4 ARAF ISG15 RAF1 STAT3 PML BUB3 TRIM35 HERC1	Cytoplasm Nucleus,	Yes	https://www.bindingdb.org/uniprot/P14618	✓
PKMYT1	HUMAN Membrane-associated tyrosine- and threonine-specific cdc2-inhibitory kinase	MYT1	Q99640	ENSG0000(	Kinase	AK098452	Breast carcinoma, Metabolic syndrome, Melanoma, Central neuroblastoma, Myeloblastosis, Neuroblastoma, Glioblastoma, Attention deficit hyperactivity disorder, Papillary thyroid carcinoma, Azoospermia	https://www.targetvalidation.org/target/ENSG00000127564/associations	PLK1 MAPK8 CDK1 BCL2 STEAP3 PLK1 CDK1 CCNB1 PIN1	Endoplasmic reticulum membrane	Yes	https://www.bindingdb.org/uniprot/Q99640	✓
PKNOX1	HUMAN Homeobox protein PKNOX1	PREP1	P55347	ENSG0000(	Transcription factor	AP001748	Blackfan-Diamond anemia, Congenital dyserythropoietic anemia, Alpha-Thalassemia, Familial partial lipodystrophy, Hereditary persistence of fetal hemoglobin - Beta-Thalassemia, Down Syndrome, Teratocarcinoma, Dementia, LCAT deficiency, Shwachman-Diamond syndrome	https://www.targetvalidation.org/target/ENSG00000160199/associations	PBX1 HOXD9 HOXB6 HOXB7 PAX6 HOXA1	Nucleus	No		✓
PLAC1	HUMAN Placenta-specific protein 1	CT92, OOSP2L	Q9HBJ0	ENSG0000(	Others	BC066327	Breast carcinoma, Adenocarcinoma, Breast adenocarcinoma, Pre-Eclampsia, Lung neoplasm, Pregnancy complication, Gastric carcinoma, Prostate carcinoma, Meckel syndrome, Medulloblastoma	https://www.targetvalidation.org/target/ENSG00000170965/associations		Secreted	No		✓

PLAGL2	HUMAN Zinc finger protein PLAGL2	KIAA0198	Q9UPG8	ENSG0000(	Transcription factor	AL121897	LCAT deficiency, Familial hypercholesterolemia, Azoospermia, Metabolic syndrome, Fish-eye disease, Acute myelocytic leukemia, Bronchogenic carcinoma, Hyperactive behavior, Centriacinar Emphysema, Lung adenocarcinoma	https://www.targetvalidation.org/target/ENSG00000126003/associations	RCHY1 HDAC7 EP300	Nucleus		No		✓
PLD2	HUMAN Phospholipase D2	PLD2	O14939	ENSG0000(	Signalling	AF038441	Systemic lupus erythematosus, Inflammatory bowel disease, Sepsis, Colitis, Cardiomegaly, Orthomyxoviridae Infection, Astrocytoma, Sarcoma, Colorectal cancer, Hypertensive disease	https://www.targetvalidation.org/target/ENSG00000129219/associations	RPTOR ACTN3 PRKCD CDK5 ARF1 GNAI1 LIMK1 JAK3 CSK PLCG1 PRKCA SRC ACTN1 FYN LCK EGFR DPYSL2 CAV1 OPRM1 RAC2 GAPDH ACTN3 GRB2 STXBP1 GM2A	Membrane		Yes	https://www.bindingdb.org/uniprot/O14939	✓
PLEKHA5	HUMAN Pleckstrin homology domain-containing family A member 5	KIAA1686, PEPP2, PKHA5	Q9HAU0	ENSG0000(	Others	AK001529	Pancreatic carcinoma, Renal cell carcinoma, Clear cell renal carcinoma, Melanoma, Leukemia, Hematologic neoplasm, Colorectal adenocarcinoma, Macroglobulinemia, Glioma, Ependymoma	https://www.targetvalidation.org/target/ENSG0000052126/associations		Cytoplasm		No		✓
PLK1	HUMAN Serine/threonine-protein kinase PLK1	PLK	P53350	ENSG0000(	Kinase,Cancer	X75932	Lung carcinoma, Liver disease, Glioma, Leukemia, Prostate carcinoma, Breast Carcinoma, Squamous cell carcinoma, Lung neoplasm, Dermatofibrosarcoma protuberans, Large cell lung carcinoma	https://www.targetvalidation.org/target/ENSG00000166851/associations	NINL NUDC INCENP ECT2 PKMYT1 CHFR GSG2 NEK9 MAVS BORA ATR PIN1 ATM PAK1 FOXM1 PPP2CA PPP2CB MAPKAPK2 MAP3K8 EIF2AK2 CCNB1 CHEK2 KIF20A STK10 BUB1 PRC1 FOXO3 PDPK1 AURKA KIF2A TUBB	Nucleus, Chromosome, centromere, kinetochore, Cytoplasm, cytoskeleton, microtubule organizing center, centrosome Midbody,		Yes	https://www.bindingdb.org/uniprot/P53350	✓
PLK2	HUMAN Serine/threonine-protein kinase PLK2	SNK	Q9NYY3	ENSG0000(	Kinase	AC008814	Breast carcinoma, Lung carcinoma, Juvenile arthritis, Multiple myeloma, Osteosarcoma, Acute myelocytic leukemia, Ovarian carcinoma, Astrocytoma, Glioblastoma multiforme, Ulcerative colitis	https://www.targetvalidation.org/target/ENSG00000145632/associations	TP53 FBXW7 WEE1 CIB1 NPM1 SNCA YWHAE IKBKAP SNCB MPP3 CENPJ CALU DLGAP4	Cytoplasm, cytoskeleton, microtubule organizing center, centrosome, centriole		Yes	https://www.bindingdb.org/uniprot/Q9NYY3	✓
PLK4	HUMAN Serine/threonine-protein kinase PLK4	Sak, STK18	O00444	ENSG0000(	Kinase	AK303399	Microcephalic primordial dwarfism, Seckel syndrome, Autosomal recessive chorioretinopathy-microcephaly, Dwarfism, Prenatal onset, Intrauterine retardation, Atrophy of cerebellum, Supratentorial atrophy, Normosmic congenital hypogonadotropic hypogonadism, Kallmann syndrome	https://www.targetvalidation.org/target/ENSG00000142731/associations	CENPJ CEP152 SMAD4 SFN TGFBR1 FBXW5 TEC RPS27A CENPJ MTUS2 FAM46C	Cytoplasm, cytoskeleton, microtubule organizing center, centrosome, centriole Cleavage furrow,		Yes	https://www.bindingdb.org/uniprot/O00444	✓
PMEL	HUMAN Melanocyte protein PMEL	D12S53E, gp100, Pmel17, SI, SIL, SILV	P40967	ENSG0000(	Others	U31799	Asthma, Griscelli disease, Melanoma, Oculocutaneous albinism, Melanoma B16, Cervix carcinoma, Vitiligo, High-Grade squamous intraepithelial lesion, Low grade squamous intraepithelial neoplasia, Hermansky-Pudlak syndrome, Retinal dystrophy	https://www.targetvalidation.org/target/ENSG00000185664/associations		Endoplasmic reticulum membrane, Single-pass type I membrane protein, Golgi apparatus, Melanosome, Endosome, multivesicular body,	Transmembrane	No		✓
PMF1	HUMAN Polyamine-modulated factor 1	PMF1	Q6P1K2	ENSG0000(	Transcription factor	AK289490	Bladder carcinoma, Chronic lymphocytic leukemia, Bladder tumor, Colorectal adenocarcinoma, Stroke, Breast carcinoma, Rheumatoid arthritis	https://www.targetvalidation.org/target/ENSG00000160783/associations	NFE2L2	Nucleus		No		✓
PMVK	HUMAN Phosphomevalonate kinase	HUMPMKI, PMK, PMKA	Q15126	ENSG0000(	Kinase	BC007694	Porokeratosis of Mibelli, Porokeratosis, Breast carcinoma, Multiple myeloma, Malignant mesothelioma, Hyperkeratosis, Chromosomal breakage, Skin photosensivity, Pruritus, Skin aplasia	https://www.targetvalidation.org/target/ENSG00000163344/associations		Peroxisome		No		✓

PNPT1	HUMAN Polyribonucleotide nucleotidyltransferase 1, mitochondrial	DFNB70, old-35, OLD35, PNPase	Q8TCS8	ENSG0000	Others	BC000862	Combined oxidative phosphorylation defect type 13, Autosomal recessive non-syndromic sensorineural deafness type DFNB, Inguinal hernia, Hearing loss, Liver carcinoma, Melanoma, Multiplem sclerosis, Myxoid cyst, Mitochondrial respiratory chain deficiency, Central neuroblastoma	https://www.targetvalidation.org/target/ENSG00000138035/associations	PRKDC	Cytoplasm, Mitochondrion, Mitochondrion intermembrane space, Peripheral membrane protein,		No		✓
PO2F2	HUMAN POU domain, class 2, transcription factor 2	OCT2, OTF2	P09086	ENSG0000	Transcription factor,Cancer	M36542	Hyper-IgM syndrome with susceptibility to opportunistic infections, Autosomal dominant severe congenital neutropenia, Agammaglobulinemia, Hodgkin's disease, Burkitt lymphoma, Colorectal carcinoma, Multiple myeloma, Cervical cancer, Primary effusion lymphoma, B-Cell lymphoma	https://www.targetvalidation.org/target/ENSG00000028277/associations	POU2AF1 AR TLE4	Cytoplasm Nucleus,		Yes	https://www.bindingdb.org/uniprot/P09086	✓
POLR2I	HUMAN DNA-directed RNA polymerase II subunit RPB9	RPB9	P36954	ENSG0000	Transcription factor	BC017112	HIV infection, Influenza infection, Teratozoospermia, Malignant peripheral nerve sheath tumor, Coronary artery disease, Multiple myeloma, Non-small cell lung carcinoma, Atherosclerosis, Sjogren's syndrome	https://www.targetvalidation.org/target/ENSG00000105258/associations	POLR2G POLR2F POLR2H POLR2B POLR2A POLR2E POLR2C POLR2D POLR2B POLR2A POLR2H POLR2E POLR2F POLR2C POLR2D POLR2G	Nucleus, nucleolus		No		✓
POMK	HUMAN Protein O-mannose kinase	SgK196	Q9H5K3	ENSG0000	Kinase	BC113703	Congenital muscular dystrophy, Qualitative or quantitative defects of alpha-dystroglycan, Walker-Warburg syndrome, Limb-girdle muscular dystrophy, Autosomal recessive limb-girdle muscular dystrophy, Impaired cognition, Prostate carcinoma, Mental retardation, Breast carcinoma, Lung adenocarcinoma	https://www.targetvalidation.org/target/ENSG00000185900/associations		Endoplasmic reticulum membrane	Transmembrane	No		✓
POTEE	HUMAN POTE ankyrin domain family member E	A26C1, A26C1A, CT104.2, POTE-2, POTE2, POTE2gamma	Q6S8J3	ENSG0000	Others	AY462872	Lung carcinoma, Non-small cell lung carcinoma, Prostate carcinoma, Breast carcinoma	https://www.targetvalidation.org/target/ENSG00000188219/associations				No		✓
POTEH	HUMAN POTE ankyrin domain family member H	A26C3, ACTBL1, CT104.7, POTE22	Q6S545	ENSG0000	Others	NA	Prostate carcinoma, Glioma	https://www.targetvalidation.org/target/ENSG00000198062/associations				No		✓
POU2AF1	HUMAN POU domain class 2-associating factor 1	OBF1	Q16633	ENSG0000	Cancer	AK313573	Lymphoid neoplasm, Melanoma, Bladder carcinoma, Primary biliary cirrhosis, Onychomycosis, Hyperpigmentation, Liver failure, Anti-nuclear factor positive, Dyslipidemias, Conjugated hyperbilirubinemia	https://www.targetvalidation.org/target/ENSG00000110777/associations	SUB1 GSK3B SYK LGALS1 SLC22A1 POU2F1 POU2F2	Nucleus,		No		✓
POU5F1	HUMAN POU domain, class 5, transcription factor 1	OCT3, Oct4, OTF3	Q01860	ENSG0000	Transcription factor,Cancer	AB088113	Adenocarcinoma, Colonic neoplasm, Gestational trophoblastic disease, Congenital heart defect, Amphetamine-Related disorder, Embryonal carcinoma, Bladder neoplasm, Psoriasis, Teratoma, Ovarian serous adenocarcinoma	https://www.targetvalidation.org/target/ENSG00000204531/associations	AKT3 EWSR1 WDR5 ZNF143 NR0B1 SOX2 AKT2 AKT1 MAPK1 MAPK3 PRKACA ETS2 PIM1 ESRRB OGT WWP2 ITCH PIN1 CDX2 FOXD3	Cytoplasm, Nucleus,		Yes	https://www.drugbank.ca/biodb/polypeptides/Q01860	✓
PPARD	HUMAN Peroxisome proliferator-activated receptor delta	NR1C2, NUC1, PPARB	Q03181	ENSG0000	Transcription factor,Signalling	AY919140	Cardiomegaly, Obesity, Fatty liver, Bipolar disorder, Cardiomyopathy, Peripheral neuropathy, Skin neoplasm, Type II diabetes mellitus, Angioma serpiginosum, Lamellar icthyosis	https://www.targetvalidation.org/target/ENSG00000112033/associations	NR1H3 NR1H2 RXRB RXRA SRC EP300 RELA RXRA HDAC1 NCOA1 NR1H3 RXRB NR1H2 RXRG	Nucleus,		Yes	https://www.bindingdb.org/uniprot/Q03181	✓
PPARG	HUMAN Peroxisome proliferator-activated receptor gamma	NR1C3	P37231	ENSG0000	Transcription factor,Cancer	L40904	Lipodystrophy, Primary lipodystrophy, Obesity, Hypertensive disease, Familial partial lipodystrophy type 3, Type II diabetes mellitus, Atherosclerosis, Colonic neoplasm, Crohn's disease, Chronic Hepatitis C	https://www.targetvalidation.org/target/ENSG00000132170/associations	NCOR2 PPARGC1A PRDM16 BRD8 WWTR1 MED1 NCOA2 NCOA4 NR1H3 FOXO1 MAP2K1 CDK5 UBE2I NR1H2 CDK9 MAPK8 SREBF1 MAP2K2 CTNNB1 RXRB MAPK1 MAPK3 NR2F2 SFPQ RXRA EGR1 PRKCA HMGA1 SRC VDR	Nucleus, Cytoplasm,		Yes	https://www.bindingdb.org/uniprot/P37231	✓

PPIC	HUMAN Peptidyl-prolyl cis-trans isomerase C	CYPC	P45877	ENSG0000	Others	BC002678	Breast carcinoma, Glioma, Small cell lung carcinoma, Astrocytoma, Gastric neoplasm, Lymphoma, Hyperglycemia, Multiple myeloma, Pulmonary ucoepidermoid carcinoma, Melanoma, Pituitary cancer	https://www.targetvalidation.org/target/ENSG00000168938/asso ciations	LGALS3BP	Cytoplasm,	Yes	https://www.bindingdb.org/uniprot/P45877	✓
PPID	HUMAN Peptidyl-prolyl cis-trans isomerase D	CYP-40	Q08752	ENSG0000	Signalling	AK313929	Bipolar disorder, Alzheimer's disease, Breast carcinoma, Hyperglycemia, Diabetes mellitus, Prostate carcinoma, Impaired cognition, Primary open angle glaucoma, Melanoma, Systemic lupus erythematous, Ischemia	https://www.targetvalidation.org/target/ENSG00000171497/asso ciations	SLC25A4 HSP90AB1 HSP90AA1 APP	Cytoplasm	Yes	https://www.bindingdb.org/uniprot/Q08752	✓
PPM1A	HUMAN Protein phosphatase 1A	PPPM1A, PP2C-alpha	P35813	ENSG0000	Kinase,Signalling	AB451247	Neurodegenerative disease, Seasonal allergic rhinitis, Familial partial lipodystrophy, Liver carcinoma, Bladder carcinoma, Prostate neoplasm, Cockayne syndrome, Jeuna syndrome, Ataxia-telangiectasia, Cushing syndrome	https://www.targetvalidation.org/target/ENSG00000100614/asso ciations	MAPK14 PRKCD PTEN MAP2K6 MAP2K4 AKT1 CACNA1A CACNA1C PRKCD CDK6 SMAD3 PRKAG1 MAPK14 SMAD1 IKBKB SMAD2 CDK2 CDC42BP A PAK2 PIK3R1 MAP2K4 PAK1 CDK9 BAD AXIN1 MAP2K6 EEF2 MSN CACNA1A CACNA1B CETN1	Nucleus	Yes	https://www.bindingdb.org/uniprot/P35813	✓
PPP1CB	HUMAN Serine/threonine-protein phosphatase PP1-beta catalytic subunit	PP-1B, PP1B	P62140	ENSG0000	Others	CR542263	Melanoma, Leukemia, Prostate carcinoma, Lung carcinoma, Chronic lymphocytic leukemia, Hereditary spastic paraplegia, Glioma, Astrocytoma, Noonan syndrome	https://www.targetvalidation.org/target/ENSG00000213639/asso ciations	RIPK3 PPP1R3C PPP1R12C PPP1R9B PPP1R10 PPP1R13B PPP1R16A BAD PPP1R3B PPP1R7 PPP1R8 PTK2 PPP1CA PPP1R2 PPP1R11 PPP1R12B AXIN1 PPP1R12A AURKA RYR1 TNNI3 NOS3 EIF2S1 PRKCA PRKD1 RAF1 STK4 PRKCD TP53	Cytoplasm	No		✓
PPP1R14A	HUMAN Protein phosphatase 1 regulatory subunit 14A	CPI-17, PPP1INL	Q96A00	ENSG0000	Others	AY050668	Li-Fraumeni syndrome, Prostate carcinoma, Breast carcinoma, Mesothelioma, Colorectal neoplasm, Bladder hypertrophy, Bartonella infection, Lymphoma, Premature obstetric labor, Cat-Scratch disease	https://www.targetvalidation.org/target/ENSG00000167641/asso ciations	CSNK1G3 CAMK2A CSNK1G1 PKN1 PRKD1 PRKG1 TP53BP2 CAMK2D ILK PRKCD PRKCZ PRKCE PKD1 CSNK1G2 PRKX CDK7 CSNK1E CSNK1D CSNK1A1 PRKACA PRKCA PRKCG ROCK2 PRKY DAPK3 PRKCA PRKCZ PPP1CA PPP1R12B	Cytoplasm	No		✓
PPP1R2P9	HUMAN Putative type-1 protein phosphatase inhibitor 4	IPP4	O14990	NA	Others	Z94277	Azoospermia, Amyotrophic lateral sclerosis, Sepsis, Colorectal cancer, Colorectal carcinoma, Glioma, Breast carcinoma, Astrocytoma, Melanoma, Huntington disease	https://www.targetvalidation.org/target/ENSG00000102055/asso ciations	PPP1CA		No		✓
PPP2CB	HUMAN Serine/threonine-protein phosphatase 2A catalytic subunit beta isoform	PPP2CB	P62714	ENSG0000	Signalling	CH471080	Acute lymphoblastic leukemia, Lung adenocarcinoma, Juvenile dermatomyositis, Prostatic neoplasm, Adenocarcinoma, Neoplasm metastasis, Rheumatoid arthritis, Hepatocellular carcinoma	https://www.targetvalidation.org/target/ENSG00000104695/asso ciations	PPP2R3B AXIN2 PPP2R2D SGO1 MAPK14 PPP2R5E PPP2R5A PPP2R5D PPP2R5C SET PPP2R2B PPP2R2A TGFBR2 TLX1 PPP2R1B PPP2R1A PRKCB AXIN1 PRKCB PRKG1 CAMK2B EGFR PRKCA PRKACA SRC PRKD1 PACS1 MAP3K7 VASP	Cytoplasm	Yes	https://www.drugbank.ca/biodb/polypeptides/P62714	✓
PPP2R1A	HUMAN Serine/threonine-protein phosphatase 2A 65 kDa regulatory subunit A alpha isoform	PPP2R1A	P30153	ENSG0000	Signalling, Cancer	BC001537	Autosomal dominant non-syndromic intellectual disability, Lung carcinoma, Mental deficiency, Facial asymmetry, Tented upper lip, Prominent metopic ridge, Kidney neoplasm, Ovarian adenocarcinoma, Breast carcinoma, Papillary renal cell carcinoma	https://www.targetvalidation.org/target/ENSG00000105568/asso ciations	MAP3K3 SGO1 PPP2CA PPP2R2A PPP2CB AKT1 SLC6A2 RPS3 HSP90AA1 CDK1 LATS1 MCM3 PPP2R1B AKT1 PPP2R3B SGO1 PPP2R5D PPP2R5C PPP2CA MAPT CXCR2 RPS3 PPP2R2C PPP2CB HSF2 PPP2R2B PPP2R2A FGFR1OP PPP2R5A	Cytoplasm	No		✓

PPP2R1B	HUMAN Serine/threonine-protein phosphatase 2A 65 kDa regulatory subunit A beta isoform	2AAB	P30154	ENSG0000(	Signalling	AK294716	Lung carcinoma, Azoospermia, Autosomal recessive predisposition, Bronchioloalveolar adenocarcinoma, Colorectal neoplasm, Colon carcinoma, Fanconi anemia, Hyper-IgM syndrome type 3, Agammaglobulinemia, Severe congenital neutropenia	https://www.targetvalidation.org/target/ENSG00000137713/associations	MYLK2 MAP3K3 TRIB3 SGO1 PPP2CA PPP2CB PPP2R1A JAK2 MCM3 RALA PPP2R3B PPP2R5D PPP2R5C PPP2CA PPP2R2C PPP2CB PPP2R5B PPP2R2A PPP2R5A PPP2R5E		No		✓
PPP2R2B	HUMAN Serine/threonine-protein phosphatase 2A 55 kDa regulatory subunit B beta isoform	PR55-BETA, SCA12	Q00005	ENSG0000(	Signalling	AK294659	Neurodegenerative disease, Autosomal dominant cerebellar ataxia, Spinocerebellar ataxia type 12, Mental or behavioural disorder, Schizophrenia, Action tremor, Cerebral atrophy, Dementia, Dysdiadochokinesis, Bipolar disorder	https://www.targetvalidation.org/target/ENSG00000156475/associations	PPP2CA PPP2CB TGFBR1 PPP2R1A RAF1 CHEK2 RAF1 PPP2CA PDPK1 PPP2CB	Cytoplasm	No		✓
PPP2R2C	HUMAN Serine/threonine-protein phosphatase 2A 55 kDa regulatory subunit B gamma isoform	IMYPNO, 2ABG	Q9Y2T4	ENSG0000(	Signalling	AK093115	Astrocytoma, Glioblastoma multiforme, Bipolar disorder, Type II diabetes mellitus, Lung carcinoma, Prostate carcinoma, Glioma, Psoriasis, Ovarian carcinoma, Squamous cell lung carcinoma	https://www.targetvalidation.org/target/ENSG00000074211/associations	PPP2CA PPP2R1B PPP2R1A RAF1 SRC PPP2CA		No		✓
PPP2R5A	HUMAN Serine/threonine-protein phosphatase 2A 56 kDa regulatory subunit alpha isoform	PPP2R5A	Q15172	ENSG0000(	Signalling	AL451060, AL360091	Hepatocellular carcinoma, Breast carcinoma, Liposarcoma, Pancreatic intraepithelial neoplasia, Pancreatic ductal adenocarcinoma, Melanoma, Fibroadenoma	https://www.targetvalidation.org/target/ENSG00000066027/associations	NEK1 SGO1 PPP2CA PPP2CB CSNK1A1 PPP2R1B PPP2R1A APC EIF2AK2 PRKCA CTLA4 BCL2 CDK1 JAK2 NOS3 APC TP63 GNL3 PPP2R5D PPP2CA MAPT AXIN1 TGFBR1 PPP2CB	Cytoplasm	Yes		✓
												https://www.bindingdb.org/uniprot/Q15172	
PPP2R5C	HUMAN Serine/threonine-protein phosphatase 2A 56 kDa regulatory subunit gamma isoform	KIAA0044	Q13362	ENSG0000(	Signalling	AK302470	Teratozoospermia, T-cell acute lymphoblastic leukemia, Chronic Lymphocytic Leukemia, Chromosomal instability, Chromosome-Defective micronuclei, Myeloid leukemia, Obesity, Gastric carcinoma, Melanoma, Hyperglycemia	https://www.targetvalidation.org/target/ENSG00000078304/associations	CAMK2A SGO1 ATM PRKAA1 EP300 PRKCD PPP2CA PPP2CB IER3 CASP3 PPP2R1B PPP2R1A MAPK1 MAPK3 CHEK2 CACNA1C GSK3A MAPK1 AKT1 BCL2 ATM RPS6KB1 CFL1 CREB1 BCAR1 PPP2CA BAD ATF1 AXIN1 MAPK3 CAMK1	Nucleus, Chromosome, centromere,	No		✓
PPP3CA	HUMAN Serine/threonine-protein phosphatase 2B catalytic subunit alpha isoform	CALNA, PPP2B, PP2BA	Q08209	ENSG0000(	Signalling	AY904364	Glioma, Muscular dystrophy, Testicular disease, Pancreatitis, Prostate neoplasm, Left ventricular hypertrophy, Alzheimer's disease, Schizophrenia, Parkinson's disease, Kenny-Caffey syndrome	https://www.targetvalidation.org/target/ENSG00000138814/associations	CABIN1 NFATC4 NFATC2 NFATC3 PRKCZ PPP3R1 FKBP1A CALM1 PSMA6 USP14 RCAN1 CASP3 ATP2B4 IRF2 BCL2 SOD1 NFATC1 PRKAR2A DNM1 AKAP5 CALM1 FLNA VASP CRYAA PPP1R1B JUN FKBP8 DNM2 GSK3B CSNK1E	Cell membrane	Yes		✓
												https://www.bindingdb.org/uniprot/Q08209	
PPP3CC	HUMAN Serine/threonine-protein phosphatase 2B catalytic subunit gamma isoform	CALNA3, CNA3	P48454	ENSG0000(	Signalling	AC037459	Glioblastoma multiforme, Mental or behavioural disorder, Schizophrenia, Nonorganic psychosis, Prostate neoplasm, Amphetamine-Related disorder, Parkinson's disease, Crohn's disease, Sjogren's syndrome, Psychosis	https://www.targetvalidation.org/target/ENSG00000120910/associations	NFATC2 CALM1 TGFBR1 NFATC1 BMPR1B PRKAR2A DNM1 CALM1 FLNA VASP CRYAA PPP1R1B JUN DNM2 GSK3B CSNK1E KSR2 BAD MAPT RCAN1 HSPB1 NFATC1 ELK1 NFATC2 NEFH PPP1R1A CANX SSH1 DNM1L GAD1 GAD2		No		✓
PPP3R1	HUMAN Calcineurin subunit B type 1	CNB, CNA2	P63098	ENSG0000(	Signalling	AC017083	Thyroid nodule, Lymphoma, Schizophrenia, Left ventricular hypertrophy, Myocardial Ischemia, Alzheimer's disease, Cornea plana, Dementia, Astrocytoma, Oligodendroglioma	https://www.targetvalidation.org/target/ENSG00000221823/associations	MAP3K5 NFATC4 NFATC2 NFATC3 PPP3CA CALM1 NFATC1 PSMA7 PPP3CA CALM1 MAP3K5 NFATC1 NFATC4 NFATC3	Cytoplasm, cytosol	Yes		✓
												https://www.drugbank.ca/biodb/polypeptides/P63098	
PPP4C	HUMAN Serine/threonine-protein phosphatase 4 catalytic subunit	PPP4, PPX	P60510	ENSG0000(	Others	X70218	Generalized pustular psoriasis, Psoriasis, Allergy, Autistic disorder, Pancreatic ductal adenocarcinoma, Glioma, Colorectal carcinoma, Breast carcinoma, Teratocarcinoma, Endometrial stromal sarcoma	https://www.targetvalidation.org/target/ENSG00000149923/associations	PPP4R3A RELA NDEL1 HDAC3 IKBKG H2AFX FOXO3 TRAF6 RPA2 MAP4K1 BANF1 IRS4 PPP4R2 PPP4R1	Cytoplasm, Nucleus, Cytoplasm, cytoskeleton, microtubule organizing center, centrosome,	No		✓

PPP4R1	HUMAN Serine/threonine-protein phosphatase 4 regulatory subunit 1	PP4R1, MEG1	Q8TF05	ENSG0000(	Others	AP001381	Hepatocellular carcinoma, Lung carcinoma, Juvenile dermatomyositis, Non-small cell lung carcinoma, Luminal B breast carcinoma, Lymphoma, Pancreatic carcinoma, Mucocutaneous lymph mode syndrome, Ulcerative colitis, Melanoma	https://www.targetvalidation.org/target/ENSG00000154845/associations	TRAF2 PPP4C		No		✓
PQBP1	HUMAN Polyglutamine-binding protein 1	MRX2, MRX55, MRXS8, RENS1, SHS	O60828	ENSG0000(	Transcription factor	AB041832	Renpenning syndrome, Breast carcinoma, Atrial septal defect, X-linked mental retardation, Microcephaly, Mental deficiency, Anxiety, Epilepsy, Sparse hair, Spinocerebellar ataxia type 1	https://www.targetvalidation.org/target/ENSG00000102103/associations	TXNL4A TRIM24 WBP11 POU3F2	Nucleus	No		✓
PRAME	HUMAN Melanoma antigen preferentially expressed in tumors	CT130, MAPE	P78395	ENSG0000(	Others	CH471095	Prostate carcinoma, Li-Fraumeni syndrome, Leukemia, Melanoma, Chronic myeloid leukemia, Medulloblastoma, Multiple myeloma, Sarcoma, Head and Neck neoplasm, Renal cell carcinoma	https://www.targetvalidation.org/target/ENSG00000185686/associations	STK19 RARA	Nucleus	No		✓
PRC1	HUMAN Protein regulator of cytokinesis 1	ASE1	O43663	ENSG0000(	Cancer,Cancer-Antigen	BC003138	Type II diabetes mellitus, Mammary neoplasm, IGA Glomerulonephritis, Hypertensive disease, Breast carcinoma, Biliary tract cancer, Bladder carcinoma, Colon carcinoma, Glioma, Ankylosing spondyltis	https://www.targetvalidation.org/target/ENSG00000198901/associations	PBK PLK1 TTK CDK2 CDK1 TP53 KIF4A RACGAP1 PLK1 ERN1 SYVN1 DERL1	Nucleus	No		✓
PRCC	HUMAN Proline-rich protein PRCC	RCCP1	Q92733	ENSG0000(	Cancer	AL590666	Lung carcinoma, Renal carcinoma, Kidney neoplasm, Clear-cell metastatic renal cell carcinoma, Melanoma, Breast carcinoma, Colorectal adenocarcinoma, Hepatocellular carcinoma, Osteosarcoma, Dedifferentiated chondrosarcoma	https://www.targetvalidation.org/target/ENSG00000143294/associations	SRSF2 MAD2L2	Nucleus	No		✓
PRDM4	HUMAN PR domain zinc finger protein 4	PFM1	Q9UKN5	ENSG0000(	Transcription factor	NA	Proteasome disability syndrome, Omodysplasia, Mucopolysaccharidosis type 3, Acromesomelic dysplasia (Grebe type), Brachydactyly type A6, Gastric carcinoma, Dubowitz syndrome, Hennekam syndrome, Madelung deformity, 4-hydroxybutyric aciduria	https://www.targetvalidation.org/target/ENSG00000110851/associations		Nucleus	No		✓
PRDX1	HUMAN Peroxiredoxin-1	NKEFA, PAGA	Q06830	ENSG0000(	Others	CR407652	Neurodegenerative disease, Alzheimer's disease, Li-Fraumeni syndrome, Lung neoplasm, Keloid, Asbestosis, Schistosomiasis, Down syndrome, Anoxia, Esophageal Neoplasm	https://www.targetvalidation.org/target/ENSG00000117450/associations	RIPK3 SRXN1 PBK NFE2L2 STK4 CDK6 PTEN PRDX2 CDK2 CDK4 AR CDK1 LCK ISG15 MYC AR PRDX4 PRDX2	Cytoplasm	No		✓
PRDX6	HUMAN Peroxiredoxin-6	1-Cys, aiPLA2, AOP2, KIAA0106, MGC46173, NSGPx, p29, PRX	P30041	ENSG0000(	Signalling	AF289352	Autoimmune disease, Arthritis, Rheumatoid arthritis, Systemic lupus erythematosus, Schizophrenia, Down syndrome, Contact dermatitis, Liver carcinoma, Myocardial ischemia, Oligodendroglioma	https://www.targetvalidation.org/target/ENSG00000117592/associations	MAPK1 NCF2 IL7R GSTP1 PSIP1 YWHAE	Cytoplasm	Yes	https://www.drugbank.ca/biodb/polypeptides/P30041	✓
PRELID1	HUMAN PRELI domain-containing protein 1, mitochondrial	CGI-106, PRELI, SSBI12	Q9Y255	ENSG0000(	Others	AF153607	Breast carcinoma, Colorectal adenocarcinoma, Malignant peripheral nerve sheath tumor, Glioblastoma multiforme, Hepatocellular carcinoma, Sjogren's syndrome, Thrombocytopenia, Lymphoma, Leukemia	https://www.targetvalidation.org/target/ENSG00000169230/associations		Mitochondrion, Mitochondrion intermembrane space	No		✓

PRKAA1	HUMAN 5'-AMP-activated protein kinase catalytic subunit alpha-1	AMPK1	Q13131	ENSG0000	Kinase	AK312947	Gastric carcinoma, Gastric cardia carcinoma, Gastric neoplasm, Obesity, Type II diabetes mellitus, Alzheimer's disease, Ovarian neoplasm, Sitosterolemia, Blackfan-Diamond anemia, Rh deficiency syndrome	https://www.targetvalidation.org/target/ENSG00000132356/associations	PRKAB1<br>CAB39<br>PRKAG2<br>PRKAG3<br>CAMKK2<br>BRSK1<br>CAMKK1<br>BRSK2<br>STK11<br>CAMK2B<br>PPP2CA<br>PPP2CB<br>PRKAG1<br>GSK3B<br>NR2C2<br>PRKACA<br>ULK1<br>PRKAB2<br>EEF2K<br>TNNI3<br>NOS3<br>EGFR<br>PRKACA<br>SYN1<br>VASP<br>CDKN1B<br>RAF1<br>SIRT2<br>CTBP1	Cytoplasm	Yes	https://www.bindingdb.org/uniprot/Q13131	✓
PRKAA2	HUMAN 5'-AMP-activated protein kinase catalytic subunit alpha-2	AMPK, AMPK2, PRKAA	P54646	ENSG0000	Kinase,Cancer,Cancer-Antigen	BC069740	Breast carcinoma, Familial hyperinsulinism, Congenital isolated hyperinsulinism, Cardiomyopathy, Cardiomegaly, Type II diabetes mellitus, Obesity, Glioma, MODY, Wolfram syndrome	https://www.targetvalidation.org/target/ENSG00000162409/associations	PRKAB1<br>PRKAG2<br>CAMKK2<br>CAMKK1<br>ULK2<br>STK11<br>TRIP6<br>PRKAG1<br>MAP2K6<br>LEP<br>PRKACA<br>ULK1<br>PRKAB2<br>MAP3K7<br>EEF2K<br>PRKACA<br>VASP<br>MAPK8<br>ARID3A<br>EP300<br>HAS2<br>PLD1<br>RPS6KA2<br>PRKAG1<br>SREBF1<br>PARP1<br>ULK1<br>PRKAB1<br>IKBKB	Cytoplasm	Yes	https://www.bindingdb.org/uniprot/P54646	✓
PRKACA	HUMAN cAMP-dependent protein kinase catalytic subunit alpha	PKACa	P17612	ENSG0000	Kinase	CH471106	Primary pigmented nodular adrenocortical disease, Colorectal adenocarcinoma, Ovarian serous adenocarcinoma, Fibrolamellar hepatocellular carcinoma, Adrenal Gland Neoplasm, Adrenal hyperplasia, Primary hypercorticolism, Neurogenic muscular atrophy, Muscle degeneration, Proximal muscle weakness	https://www.targetvalidation.org/target/ENSG00000072062/associations	DYRK1B<br>PPP1R1B<br>GNG12<br>NLK SGK2<br>AKAP9<br>SMO<br>TESK2<br>CAMKK2<br>AURKB<br>BAD<br>AKAP1<br>HIPK4<br>MAPKAPK<br>5 HIPK1<br>PXK KSR2<br>MAPK14<br>PDE3A<br>CAMK2D<br>CAMK2G<br>CAMK2B<br>ILK PDE3B<br>PAK1<br>PRKAA1<br>SRPK2<br>PPP2CA<br>PPP2CB<br>YWHAE<br>PKIA<br>PRKAA2	Cytoplasm, Cell membrane, Nucleus	Yes	https://www.bindingdb.org/uniprot/P17612	✓
PRKACB	HUMAN cAMP-dependent protein kinase catalytic subunit beta	PKACb	P22694	ENSG0000	Kinase	AB209189	Colonic neoplasm, Colorectal adenocarcinoma, Cholangiocarcinoma, Biliary Tract Neoplasm, Brain ischemia, Alzheimer's disease, Gastric carcinoma, Carney complex, Growth hormone-secreting pituitary adenoma, Adrenal hypertrophy	https://www.targetvalidation.org/target/ENSG00000142875/associations	DYRK1B<br>BAD<br>BMPR1A<br>PRKACA<br>PRKAR2A<br>PRKAR1A<br>NGFR<br>RPS6KA5<br>DCLK1<br>PRKAR2A<br>NOXA1<br>BAD<br>RCAN1<br>PRKAR1A<br>NGFR<br>PKIA<br>PRKAR2B<br>MYBPC3<br>PKIB	Cytoplasm	Yes	https://www.bindingdb.org/uniprot/P22694	✓
PRKACG	HUMAN cAMP-dependent protein kinase catalytic subunit gamma	PKACg	P22612	ENSG0000	Kinase	AL162730	Rare hemorrhagic disorder due to a constitutional platelet anomaly, Hepatocellular carcinoma, Hemorrhage, Macrothrombocytopenia, Anemia, Spontaneous haematoma, Autosomal recessive predisposition, Epistaxis, Menorrhagia, Gray platelet syndrome	https://www.targetvalidation.org/target/ENSG00000165059/associations	BAD NOS3<br>KCNQ4<br>BAD<br>MYBPC3		Yes	https://www.bindingdb.org/uniprot/P22612	✓
PRKAG3	HUMAN 5'-AMP-activated protein kinase subunit gamma-3	AAKG3, AMPKG3	Q9UGI9	ENSG0000	Kinase	BC098255	Type I diabetes mellitus, MODY, Inborn error carbohydrate metabolism, Inborn error lipid metabolism, Polycystic ovary syndrome, Alzheimer's disease, Atherosclerosis, Diffuse large B-Cell lymphoma, Hypertrophy, Obesity	https://www.targetvalidation.org/target/ENSG00000115592/associations	ULK2<br>PRKAA1<br>ULK1<br>PRKAA1		Yes	https://www.drugbank.ca/biodb/polypeptides/Q9UGI9	✓
PRKAR1A	HUMAN cAMP-dependent protein kinase type I-alpha regulatory subunit	CNC1, PRKAR1, TSE1, PKR1	P10644	ENSG0000	Kinase,Cancer	S54707	Acrodysostosis, Carney complex, Acrodysostosis with multiple hormone resistance, Acute promyelocytic leukemia, Papillary thyroid carcinoma, Familial atrial myxoma, Cushing syndrome, Acrodysostosis, Pituitary adenoma, Neurilemmoma	https://www.targetvalidation.org/target/ENSG00000108946/associations	CAMK2A<br>PDE4DIP<br>RPS6KA1<br>CDK16<br>GRB2<br>PRKX<br>MAPK9<br>MAPK1<br>CDK2<br>PRKACB<br>PRKACA<br>ITGA4<br>CDK4<br>EGFR<br>AKAP7<br>PRKACA<br>MAPK1<br>MYO7A<br>MTOR<br>ITGA4<br>PRKACB<br>PATZ1<br>PRKAR1B<br>HLA-A<br>HLA-A	Cell membrane	Yes	https://www.bindingdb.org/uniprot/P10644	✓
PRKCB	HUMAN Protein kinase C beta type	PKCB, PRKCB1, PRKCB2, KPCB	P05771	ENSG0000	Kinase,Cancer	FJ907246	Retinopathy, Diabetic retinopathy, Type II diabetes mellitus, Autistic disorder, Major depressive disorder, Hyperglycemia, Unipolar depression, Cardiomegaly, Gastric neoplasm, Colorectal neoplasm	https://www.targetvalidation.org/target/ENSG00000166501/associations	PLCB1<br>TRIM41<br>PXK<br>PHLPP2<br>STK11<br>ACVR2B<br>BTK<br>PPP2CA<br>PPP2CB<br>PPP1CA<br>DVL1P1<br>BLVRA<br>CSF2RB<br>ITGB7<br>GRK2<br>TNFRSF1<br>A LCP1<br>PHLPP1<br>PDPK1<br>DVL2<br>GRIN2B<br>EWSR1<br>GNAZ<br>YWHAG<br>MYH9<br>RACK1<br>GJA1<br>TRPV1<br>GRIN1	Cytoplasm	Yes	https://www.bindingdb.org/uniprot/P05771	✓

PRKCH	HUMAN Protein kinase C eta type	PKC-L, PKCL, PRKCL	P24723	ENSG0000(	Kinase,Cancer	AL138996	Rheumatoid arthritis, Obesity, Stroke, Cerebral infarction, Brain infarction, Cerebrovascular accident, Renal cell carcinoma, Myopathy, Atrophic gastritis, Hypertensive disease	https://www.targetvalidation.org/target/ENSG00000027075/associations	NOS3 ANXA1 CTTN SRC PRKD1 FYN GSK3A CCNE1 NPM1 GSK3B ITGB2 CDK2 PTPN11 PDPK1 GSTP1 PRKD2	Cytoplasm	Yes	https://www.bindingdb.org/uniprot/P24723	✓
PRKCI	HUMAN Protein kinase C iota type	DXS1179E	P41743	ENSG0000(	Kinase,Cancer	CH471052	Leukemia, Acute myeloid leukemia, Bipolar disorder, Depressive disorder, Obesity, Type II diabetes mellitus, Glioblastoma, Squamous cell carcinoma, Glioma, Psoriasis	https://www.targetvalidation.org/target/ENSG00000163558/associations	PARD6A MAP2K5 CDK5 PPP2CA PPP2CB IRAK1 SRC PDPK1 NUMB NOS3 ANXA1 CTTN SRC IRAK1 PRKCZ MARK2 FBXW7 SMG1 MAP2K1 GAPDH YWHAZ EZR HRAS IKBKB NFKBIA CDK7 MTA1 MAP2K5 CDC42 RHOQ IKBKG TRAF6 AKT3 PARD6A PARD6B PARD6G PAWR AJUBA PPP1R14A WWC1 PDK1 AGER GRB14 SQSTM1	Cytoplasm	Yes	https://www.bindingdb.org/uniprot/P41743	✓
PRKCZ	HUMAN Protein kinase C zeta type	PKC2	Q05513	ENSG0000(	Kinase	AK294649	Leukemia, Acute myeloid leukemia, Bipolar disorder, Type II diabetes mellitus, Degenerative polyarthritis, Renal cell carcinoma, Myopathy, Mammary neoplasm, Mast-cell leukemia, Glioma	https://www.targetvalidation.org/target/ENSG00000067606/associations	MAP2K5 C1QBP PRKCD PRKCQ CSNK2A1 CSNK2B YWHAZ RHOA CDC42 CASP9 IRAK1 PIK3CG PRKCI VHL YWHAB AKT1 PXK MYLK	Cytoplasm	Yes	https://www.bindingdb.org/uniprot/Q05513	✓
PRKD2	HUMAN Serine/threonine-protein kinase D2	HSPC187, PKD2	Q9BZL6	ENSG0000(	Kinase	AC008635	Chronic lymphocytic leukemia, Type I diabetes mellitus, Polycystic ovary syndrome, Chromosome aberration, Myeloid leukemia, Carcinoid tumor, Gastric neoplasm, Lymphocytosis, Polycystic kidney disease, Glioblastoma	https://www.targetvalidation.org/target/ENSG00000105287/associations	PKD2 PRKCE CSNK1E CSNK1D CSNK1A1 PRKCH PRKCA CDK1 ABL1 CASK PKD2 VASP MARK2 CIB1 RIN1 GORASP1 SET RXRA RARB HDAC7 HDAC5 HEY1 PTPN3 GRIP1 SSH1 PI4KB LNPEP DST PDLIM7 SNRNP70	Cytoplasm	Yes	https://www.bindingdb.org/uniprot/Q9BZL6	✓
PRKD3	HUMAN Serine/threonine-protein kinase D3	EPK2, PRKCN, KPCD3	O94806	ENSG0000(	Kinase	CH471053	Leukemia, Acute myeloid leukemia, Melanoma, Prostate carcinoma, Psoriasis, Ulcerative colitis, Mast-cell leukemia, Multiple myeloma, Uveal melanoma, Posterior uveitis	https://www.targetvalidation.org/target/ENSG00000115825/associations	IKBKG MAPK14 GSK3A PRKCA VAMP2 PRKD1 GSK3A KPNA2 HDAC5 KPNB1 GIT1	Cytoplasm	Yes	https://www.bindingdb.org/uniprot/O94806	✓
PRKRA	HUMAN Interferon-inducible double-stranded RNA-dependent protein kinase activator A	DYT16, HSD14, PACT, RAX	O75569	ENSG0000(	Kinase,Cancer,Cancer-Antigen,Interleukin-Interferon	AL136615	Combined dystonia, Dystonia 16, Atopic eczema, Psoriasis, Blackfan-Diamond anemia, Parkinson's disease, Speech impairment, Hyperreflexia, Bradykinesia, Anotia	https://www.targetvalidation.org/target/ENSG00000180228/associations	EIF2AK2 CHUK DDX58 EIF2AK2	Cytoplasm, perinuclear region, Cytoplasm,	No		✓
PRKX	HUMAN cAMP-dependent protein kinase catalytic subunit PRKX	PKX1	P51817	ENSG0000(	Kinase,Cancer,Cancer-Antigen	NA	Amyotrophic lateral sclerosis, Medulloblastoma, Astrocytoma, Autosomal dominant polycystic kidney, XX male, 46-XY female, Renal carcinoma, Primitive neuroctodermal tumor, Oligodendroglioma, Melanoma	https://www.targetvalidation.org/target/ENSG00000183943/associations	PDE3B PLCB3 PRKAR2A ATP2B1 AQP2 STMN1 NOS3 ANXA1 PKD1 RYR2 RHOA SRC MAP3K7 PLCG1 VASP APC RAF1 GSK3A FGF2 HNRNPD PPP1R14A CETN2 AURKA NOLC1 SOX9 GNA13 NFKB1 YWHAZ BRAF GSK3B	Cytoplasm, Nucleus,	Yes	https://www.bindingdb.org/uniprot/P51817	✓
PRL	HUMAN Prolactin	PRL	P01236	ENSG0000(	Cancer, Cytokine/Chemokine	X54393	Amyloidosis, Adenoma, Systemic lupus erythematosus, Prolactinoma, Pituitary neoplasm, Hyperprolactinemia, Autistic disorder, Female infertility, Schizophrenia, Amenorrhea	https://www.targetvalidation.org/target/ENSG00000172179/associations	PAK2 PPIB PAK2 PRLR	Secreted	No		✓
PRM1	HUMAN Sperm protamine P1	PRM1	P04553	ENSG0000(	Others	Y00443	Azoospermia, Male infertility, Oligospermia, Nephroblastoma, Varicocele, Teratozoospermia	https://www.targetvalidation.org/target/ENSG00000175646/associations	SRPK1 RPS6KA5	Nucleus, Chromosome,	No		✓

PRM2	HUMAN Protamine-2	hCG_15066	P04554	ENSG0000(	Others	AK292447	Azoospermia, Teratozoospermia, Male infertility, Polycythemia Vera, Oligospermia, Prostate carcinoma, Vein graft stenosis, Diffuse large B-cell lymphoma, Systemic lupus erythematosus, Inflammation	https://www.targetvalidation.org/target/ENSG00000122304/associations	STK4	Nucleus	No		✓
PRPF4	HUMAN U4/U6 small nuclear ribonucleoprotein Prp4	HPRP4, HPRP4P, PRP4, Prp4p, SNRNP60	O43172	ENSG0000(	Kinase	AL449305	Retinitis pigmentosa, Melanoma, Breast carcinoma, Breast adenocarcinoma, Psoriasis, Broad flat nasal bridge, Lens opacities, Progressive night blindness, Pallor of optic disc, Anteverted nostril	https://www.targetvalidation.org/target/ENSG00000136875/associations	CLK1 TGFBR1 ELK1 PRPF3 PPIH	Nucleus speckle	Yes	https://www.bindingdb.org/uniprot/O43172	✓
PRPS2	HUMAN Ribose-phosphate pyrophosphokinase 2	PRPS2	P11908	ENSG0000(	Kinase	BC110875	Hyper-IgM syndrome with susceptibility to opportunistic infections, Systemic lupus erythematosus, Hyper-IgM syndrome type 3, Teratozoospermia, Glioma, Colorectal adenocarcinoma, Sertoli cell-only syndrome, Psoriasis, Teratozoospermia, Astrocytoma	https://www.targetvalidation.org/target/ENSG00000101911/associations			No		✓
PRSS50	HUMAN Probable threonine protease PRSS50	CT20, TSP50	Q9UI38	ENSG0000(	Others	AF100707	Breast carcinoma, Gastric carcinoma, Mammary neoplasm, Mental depression, Colorectal carcinoma, Azoospermia, Cervical carcinoma, Testicular germ cell tumor, Embryonal carcinoma, Unipolar depression	https://www.targetvalidation.org/target/ENSG00000206549/associations		Endoplasmic reticulum	No		✓
PRSS54	HUMAN Inactive serine protease 54	CT67, KLKBL4	Q6PEW0	ENSG0000(	Others	AK058068	Azoospermia, Systemic lupus erythematosus, Melanoma, Chronic kidney failure	https://www.targetvalidation.org/target/ENSG00000103023/associations		Secreted	No		✓
PSCA	HUMAN Prostate stem cell antigen	PSCA	O43653	ENSG0000(	Cancer	BC023582	Duodenal ulcer, Bladder carcinoma, Gastric carcinoma, Linitis plastica, Prostate carcinoma, Adenocarcinoma, Schizophrenia, Hyperplasia, Tauopathy, Idiopathic pulmonary fibrosis	https://www.targetvalidation.org/target/ENSG00000167653/associations		Cell membrane, Lipid-anchor, GPI-anchor,	No		✓
PSG1	HUMAN Pregnancy-specific beta-1-glycoprotein 1	CD66f, PBG1, PSBG1, PSGGA	P11464	ENSG0000(	Others	M25385	Squamous cell carcinoma, Lung carcinoma, Squamous cell lung carcinoma, Breast carcinoma, Papillary thyroid carcinoma, Parkinson's disease, Dementia, Frontotemporal dementia, Malignant mesothelioma, Isolated scaphocephaly	https://www.targetvalidation.org/target/ENSG00000231924/associations		Secreted	No		✓
PSG11	HUMAN Pregnancy-specific beta-1-glycoprotein 11	PSG13, PSG14	Q9UQ72	ENSG0000(	Others	AC005947	Breast carcinoma, Cervical carcinoma, Glioblastoma multiforme	https://www.targetvalidation.org/target/ENSG00000243130/associations		Secreted	No		✓
PSIP1	HUMAN PC4 and SFRS1-interacting protein	LEDGF, p52, p75, PSIP2	O75475	ENSG0000(	Transcription factor,Cancer	AF199339	HIV infection, Bladder carcinoma, Acute myelocytic leukemia, Alopecia areata, Glioma, Neuroblastoma, Melanoma, Lung carcinoma, Colonic neoplasm, Cutaneous melanoma	https://www.targetvalidation.org/target/ENSG00000164985/associations	CASP3 TNF CDC7 MEN1 CRYAB PRDX6 ADH1A ALDH1A2 ADH4 GCLC HCFC1 SUMO1 HSPB1 SRSF1	Nucleus	Yes	https://www.bindingdb.org/uniprot/O75475	✓
PSMA6	HUMAN Proteasome subunit alpha type-6	p27K, PROS27	P60900	ENSG0000(	Others	AK302008	Multiple myeloma, Neoplasm of mature B-cells, Macroglobulinemia, Myocardial infarction, HIV infection, Type II diabetes mellitus, Cerebrovascular accident, Coronary artery disease, Atherosclerosis, Juvenile arthritis	https://www.targetvalidation.org/target/ENSG00000100902/associations	UCHL5 POMP PSMA8 ADRM1 PRKCZ PSMD4 PLK1 PSMA5 PSMA4 PSMA3 PSMA2 PSMA1 HSPB1 PSMD3 UBD PSMD14 PSMD11 CUL1 PPP3CA PSMD7 PSMA7 ADRM1 UCHL5 PSMC3 CDK2 PSMD4 PSMC2 PSMB5 PSME2	Cytoplasm	Yes	https://www.bindingdb.org/uniprot/P60900	✓

PSMB10	HUMAN Proteasome subunit beta type-10	beta2i, LMP10, MECL1	P40306	ENSG0000(	Others	BT019724	Neoplasm of mature B-cells, Macroglobulinemia, Mantle cell lymphoma, HIV infection, Autoimmune disease, Non-arthropod borne lymphocytic choriomeningitis, Inclusion body myositis, Multiple myeloma, Melanoma, Lung carcinoma	https://www.targetvalidation.org/target/ENSG00000205220/associations	PSMB7<br>PSMA6<br>PSMD4<br>PSMB3<br>PSMB5<br>PSMB6<br>PSMB4<br>PSMA5<br>PSMB9<br>PSMB8<br>PSMA4<br>PSMA3<br>PSMA2<br>PSMA1<br>IFNG<br>PSMD3<br>PSMA7<br>PSMD11<br>PSMD7<br>PSMC3<br>PSMD4<br>PSMC2<br>PSMB5<br>PSME2<br>PSMC1<br>PSME1<br>PSMD1<br>PSMD3<br>PSMD13	Cytoplasm	Yes	https://www.bindingdb.org/uniprot/P40306	✓
PSMB3	HUMAN Proteasome subunit beta type-3	PSMB3	P49720	ENSG0000(	Signalling	D26598	Neoplasm of mature B-cells, Multiple myeloma, Macroglobulinemia, Mantle cell lymphoma, Diffuse large B-cell lymphoma, AL amyloidosis, Bronchiolitis obiterans, Ovariann carcinoma, Glioblastoma multiforme	https://www.targetvalidation.org/target/ENSG00000277791/associations	UCHL5<br>POMP<br>PSMA8<br>PSMA6<br>PSMD4<br>PLK1<br>PSMB2<br>PSMA5<br>PSMA4<br>PSMA3<br>PSMA2<br>PSMA1<br>PSMB1<br>PSMD3<br>PSMA7<br>PSMD14<br>PSMD11<br>PSMD7<br>UCHL5<br>PSMC3<br>PSMD4<br>PSMC2<br>PSMB5<br>PSME2<br>PSMC1<br>PSME1<br>PSMD1<br>PSMD3<br>PSMD12	Cytoplasm	Yes	https://www.bindingdb.org/uniprot/P49720	✓
PSMB8	HUMAN Proteasome subunit beta type-8	beta5i, LMP7, PSMB5i, RING10, PSB8	P28062	ENSG0000(	Others	Z14982	Proteasome disability syndrome, Nakajo-Nishimura syndrome, CANDLE syndrome, JMP syndrome, Lipodystrophy, Panniculitis, Iron-refractory iron deficiency anemia, Macroglobulinemia, Non-Hodgkin's lymphoma	https://www.targetvalidation.org/target/ENSG00000204264/associations	POMP<br>PSMB7<br>PSMA6<br>PSMD4<br>PSMB2<br>PSMB3<br>PSMB5<br>PSMB6<br>PSMB4<br>PSMA5<br>PSMA4<br>PSMA3<br>PSMA2<br>PSMA1<br>PSMB1<br>IFNG<br>PSMD3<br>PSMA7<br>PSMD11<br>PSMD7<br>PSMC3<br>PSMD4<br>PSMC2<br>PSMB5<br>PSME2<br>PSMC1<br>PSME1<br>PSMD1<br>PSMD3	Cytoplasm	Yes	https://www.bindingdb.org/uniprot/P28062	✓
PSMB9	HUMAN Proteasome subunit beta type-9	beta1i, LMP2, PSMB6i, RING12	P28065	ENSG0000(	Others	X87344	Lymphoma, Neoplasm of mature B-cells, Non-Hodgkin's lymphoma, Mammary neoplasm, Type I diabetes mellitus, Psoriasis, Juvenile arthritis, Alzheimer's disease, Ankylosing spondylitis, Peptic esophagitis	https://www.targetvalidation.org/target/ENSG00000240065/associations	POMP<br>PSMD13<br>PSME2<br>PSMD1<br>PSMB7<br>PSMD5<br>PSMD6<br>PSMD2<br>PSMC6<br>PSMC5<br>PSMA6<br>PSMD4<br>PSMD7<br>PSMB2<br>PSMB3<br>PSMD8<br>PSMC4<br>PSMB10<br>PSMC2<br>PSMB5<br>PSMB6<br>PSMB4<br>PSMA5<br>PSMB8<br>PSMA4<br>PSMA3<br>PSMA2<br>PSMA1<br>PSMC3	Cytoplasm	Yes	https://www.bindingdb.org/uniprot/P28065	✓
PSMD4	HUMAN 26S proteasome non-ATPase regulatory subunit 4	AF, AF-1, Rpn10, S5A	P55036	ENSG0000(	Cancer,Cancer-Antigen	AL391069, AL592424	Neoplasm of mature B-cells, Multiple myeloma, Mantle cell lymphoma, Non-small cell lung carcinoma, Breast carcinoma, Brain lesion, Spastic syndrome, Cervix carcinoma, Myelodysplastic syndrome, Renal carcinoma	https://www.targetvalidation.org/target/ENSG00000159352/associations	UCHL5<br>UBQLN1<br>PSMD1<br>PSMB7<br>ADRM1<br>PSMD2<br>PSMC6<br>PSMC5<br>PSMC1<br>PSMA6<br>RAD23A<br>PSMB2<br>PSMB3<br>NEDD4<br>PSMC4<br>ID1<br>PSMB10<br>PSMC2<br>PSMB5<br>PSMB6<br>PSMB4<br>PSMA5<br>PSMB9<br>PSMB8<br>PSMA4<br>PSMA3<br>PSMA2<br>PSMA1<br>TGM2		No		✓
PSMD9	HUMAN 26S proteasome non-ATPase regulatory subunit 9	p27, Rpn4	O00233	ENSG0000(	Cancer,Cancer-Antigen	AC069503	Insomnia, Type II diabetes mellitus, Breast neoplasm, Glioma, Liver carcinoma, Mental depression, Gastrointestinal stromal tumor, Colorectal carcinoma, Carpel tunnel syndrome, MODY	https://www.targetvalidation.org/target/ENSG00000110801/associations	PSMC6<br>PSMC3<br>TCF3		No		✓
PSME1	HUMAN Proteasome activator complex subunit 1	IFI5111, PA28alpha	Q06323	ENSG0000(	Others	BT019337	Leiomyosarcoma, HIV infection, Esophagus squamous cell carcinoma, Colonic neoplasm, Ovarian neoplasm, Autoimmune disease, Inclusion body myositis, Tuberculosis, Myofibrillar myopathy	https://www.targetvalidation.org/target/ENSG00000092010/associations	PSME2<br>PSMB7<br>PSMC5<br>PSMA6<br>PSMB2<br>PSMB3<br>PSMB10<br>PSMB5<br>PSMB6<br>PSMB4<br>PSMA5<br>PSMB9<br>PSMB8<br>PSMA4<br>PSMA3<br>PSMA2<br>PSMA1<br>PSMB1<br>IL7R BRAF<br>IFNG<br>PSMA7<br>PSMD14<br>PSME2<br>PSMB6		No		✓
PSME2	HUMAN Proteasome activator complex subunit 2	PA28beta	Q9UL46	ENSG0000(	Signalling	BC004368	Chronic mucocutaneous candidosis, Asthma, Sepsis, HIV infection, Esophagus squamous cell carcinoma,<br>Colonic neoplasm, Adenocarcinoma, Gastric adenocarcinoma, Multiple myeloma, Non-neoplastic disorder	https://www.targetvalidation.org/target/ENSG00000100911/associations	PSMB7<br>PSME1<br>PSMA6<br>PSMB2<br>PSMB3<br>NAA10<br>PSMB10<br>PSMB5<br>PSMB6<br>PSMB4<br>PSMA5<br>PSMB9<br>PSMB8<br>PSMA4<br>PSMA3<br>PSMA2<br>PSMA1<br>PSMB1<br>IL7R IFNG<br>PSMA7<br>PSMB5<br>PSME1<br>PSMB9<br>PSMB6<br>PSMA4		No		✓

PSME3	HUMAN Proteasome activator complex subunit 3	Ki, PA28-gamma, PA28G, REG-GAMMA	P61289	ENSG0000(	Cancer,Cancer-Antigen	AK074999	Breast carcinoma, Colitis, HIV Infection, Hepatitis C infection, Steatohepatitis, Systemic lupus erythematosus, Discoid lupus erythematosus, Lupus vulgaris, Fatty liver, Liver cirrhosis	https://www.targetvalidation.org/target/ENSG00000131467/associations	PSMA5 PSMA1 CHEK2 SIRT1 NCOA3 KLF2 MAP3K3 CDKN2A CDKN1A PSMA5 PSMA1 MAFA AICDA	Nucleus		No		✓
PTEN	HUMAN Phosphatidylinositol 3,4,5-trisphosphate 3-phosphatase and dual-specificity protein phosphatase PTEN	MMAC1, TEP1	P60484	ENSG0000(	Cancer	AF143315	Inherited cancer-predisposing syndrome, PTEN hamartoma tumor syndrome, Cowden syndrome, Multiple hamartoma syndrome, Endometrial carcinoma, Head and Neck squamous cell carcinoma, Bannayan-Riley-Ruvalcaba syndrome, Autistic disorder, Meningioma, Ovarian neoplasm	https://www.targetvalidation.org/target/ENSG00000171862/associations	MAST1 PLK3 CDK19 USP7 PIK3C3 MAGI2 PREX2 MAST2 MAGI3 STK11 ROCK1 ATM PRKCZ PTK2 RELA XIAP CSNK2A1 PPP1CA CDC42 GSK3B NEDD4 FRK PPARG CTNNB1 AKT1 FGFR3 FGFR2 CSNK2A2 EGR1 SRC	Cytoplasm		Yes	https://www.bindingdb.org/uniprot/P60484	✓
PTGDR	HUMAN Prostaglandin D2 receptor	PTGDR	Q13258	ENSG0000(	Others	EF577397	Asthma, Moyamoya disease, Brooke-Spiegler syndrome, Atopic dermatitis, Bronchial hyperreactivity, Epithelial ovarian cancer, Hay fever, Neutrophilia, Ovarian carcinoma, Rosacea	https://www.targetvalidation.org/target/ENSG00000168229/associations	GNAS ANKRD13C PTGDR2	Cell membrane	Transmembrane	Yes	https://www.bindingdb.org/uniprot/Q13258	✓
PTGER3	HUMAN Prostaglandin E2 receptor EP3 subtype	EP3, PE2R3	P43115	ENSG0000(	Others	L27490	Hemorrhage, Obesity, Uterine fibroid, Stevens-Johnson syndrome, Schizophrenia, Thrombosis, Polycystic ovary syndrome, Asthma, Edema, Renovascular hypertension	https://www.targetvalidation.org/target/ENSG00000050628/associations	PTGER1 GNAI1 GNB3 GNG12 MKLN1	Cell membrane, Multi-pass membrane protein,	Transmembrane	Yes	https://www.bindingdb.org/uniprot/P43115	✓
PTK2_iso1	HUMAN Focal adhesion kinase 1	FADK, FAK, FAK1, PPP1R71	Q05397	ENSG0000(	Kinase,Cancer	AC067931	Lung carcinoma, Liver carcinoma, Squamous cell carcinoma, Glioblastoma, Rheumatoid arthritis, Nephritis, Glioma, Hepatocellular carcinoma, Meningioma, hepatoblastoma	https://www.targetvalidation.org/target/ENSG00000169398/associations	TLN1 CAMK2A MAPK8IP3 ARHGAP26 ASAP1 PTPRH PTPN23 SORBS1 PIK3R3 RB1CC1 NEDD9 PTK2B ROCK1 ACTN3 PTPN11 PTPN12 CDK5 PKD1 GRB2 PPP1CB PPP1CA CXCR4 PTEN BMX DNM2 GSK3B PXN CRKL CRK SYK DCC CASP3	Cell junction, focal adhesion, Cell membrane, Peripheral membrane protein, Cytoplasmic side, Cytoplasm, cell cortex, cytoskeleton, microtubule organizing center, centrosome Nucleus,		Yes	https://www.bindingdb.org/uniprot/Q05397	✓
PTK2_iso2	HUMAN Focal adhesion kinase 1	FADK, FAK, FAK1, PPP1R71	Q05397	ENSG0000(	Kinase,Cancer	AC067931	Lung carcinoma, Liver carcinoma, Squamous cell carcinoma, Glioblastoma, Rheumatoid arthritis, Nephritis, Glioma, Hepatocellular carcinoma, Meningioma, hepatoblastoma	https://www.targetvalidation.org/target/ENSG00000169398/associations	TLN1 CAMK2A MAPK8IP3 ARHGAP26 ASAP1 PTPRH PTPN23 SORBS1 PIK3R3 RB1CC1 NEDD9 PTK2B ROCK1 ACTN3 PTPN11 PTPN12 CDK5 PKD1 GRB2 PPP1CB PPP1CA CXCR4 PTEN BMX DNM2 GSK3B PXN CRKL CRK SYK DCC CASP3	Cell junction, focal adhesion, Cell membrane, Peripheral membrane protein, Cytoplasmic side, Cytoplasm, cell cortex, cytoskeleton, microtubule organizing center, centrosome Nucleus,		Yes	https://www.bindingdb.org/uniprot/Q05397	✓
PTK6	HUMAN Protein-tyrosine kinase 6	BRK	Q13882	ENSG0000(	Kinase	AK301364	Lung carcinoma, Thyroid carcinoma, Breast carcinoma, Lymphoma, Squamous cell carcinoma, Colon carcinoma, Epithelioma, Melanoma, Adenocarcinoma, Cutaneous melanoma	https://www.targetvalidation.org/target/ENSG00000101213/associations	KHDRBS1 AKT1 SFPQ ERBB2 EGFR CTNNB1 EGFR PXN RUVBL2 AKT1 ARHGAP35 ERBB3 BCAR1 STAT3 STAT5B KHDRBS1 STAP2 KLF5 CAMK1 ARHGAP5 ARAP1 PDE7A	Cytoplasm, Nucleus, Cell projection, ruffle, Membrane		Yes	https://www.bindingdb.org/uniprot/Q13882	✓
PTK7_ext	HUMAN Inactive tyrosine-protein kinase 7	CCK4	Q13308	ENSG0000(	Kinase	AF531868	Short rib-polydactyly syndrome, Short rib-polydactyly syndrome, Verma-Naumoff type, Jeune syndrome, Marfan syndrome, Acute myelocytic leukemia, Panic disorder, Esophagus squamous cell carcinoma, Prostate carcinoma, LCAT deficiency	https://www.targetvalidation.org/target/ENSG00000112655/associations	RACK1 CTNNB1	Membrane	Transmembrane	No		✓
PTK7_int	HUMAN Inactive tyrosine-protein kinase 7	CCK4	Q13308	ENSG0000(	Kinase	AF531868	Short rib-polydactyly syndrome, Short rib-polydactyly syndrome, Verma-Naumoff type, Jeune syndrome, Marfan syndrome, Acute myelocytic leukemia, Panic disorder, Esophagus squamous cell carcinoma, Prostate carcinoma, LCAT deficiency	https://www.targetvalidation.org/target/ENSG00000112655/associations	RACK1 CTNNB1	Membrane	Transmembrane	No		✓

PTP4A1	HUMAN Protein tyrosine phosphatase type IVA 1	PRL-1, PTPCAAX 1	Q93096	ENSG0000(	Others	CR749458	Breast carcinoma, Melanoma, Adenocarcinoma, Squamous cell carcinoma, T-Cell Lymphoma, B-Cell Lymphoma, Lung neoplasm, Multiple myeloma, Duchenne muscular dystrophy, Atopic eczema	https://www.targetvalidation.org/target/ENSG00000112245/associations	CD302 TUBA1B ATF7	Cell membrane, Early endosome, Endoplasmic reticulum, Cytoplasm, cytoskeleton, spindle,	Yes	https://www.bindingdb.org/uniprot/Q93096	✓
PTP4A2	HUMAN Protein tyrosine phosphatase type IVA 2	HU-PP-1, OV-1, PRL-2, ptp-IV1a, PTP4A, PTPCAAX 2, BM-008	Q12974	ENSG0000(	Others	L48723	Breast carcinoma, Melanoma, Adenocarcinoma, Hodgkin's Lymphoma, Precursor B-cell lymphoblastic leukemia, Epithelial ovarian cancer, Prostate carcinoma, Psoriasis, Pick disease, Onchocerciasis	https://www.targetvalidation.org/target/ENSG00000184007/associations	RABGGTB CDK2	Cell membrane, Early endosome, Cytoplasm,	Yes	https://www.bindingdb.org/uniprot/Q12974	✓
PTP4A3	HUMAN Protein tyrosine phosphatase type IVA 3	PRL-3, PRL-R, PRL3, TP4A3	O75365	ENSG0000(	Kinase	BC003105	Li-Fraumeni syndrome, Gastric neoplasm, Colorectal neoplasm, Liver carcinoma, Mammary neoplasm, Neuroblastoma, Malignant mesothelioma, Endometriosis, Astrocytoma, Periodontitis	https://www.targetvalidation.org/target/ENSG00000184489/associations	STMN1 KRT8 EZR ITGB1 NCL BCAR1	Cell membrane	Yes	https://www.bindingdb.org/uniprot/O75365	✓
PTPA	HUMAN Serine/threonine-protein phosphatase 2A activator	PR53, PTPA, PPP2R4	Q15257	ENSG0000(	Signalling	X86428	Rheumatoid arthritis, Bipolar disorder, Gastric neoplasm, Alzheimer's disease, Breast carcinoma, Chronic Hepatitis C infection, Ovarian neoplasm, Prostate neoplasm, Melanoma, Hypersomnia	https://www.targetvalidation.org/target/ENSG00000119383/associations	CSNK2A1 PPP2CA SRC MID1 CCNG1 AKT1 PPP2CA	Cytoplasm	Yes	https://www.bindingdb.org/uniprot/Q15257	✓
PTPN1	HUMAN Tyrosine-protein phosphatase non-receptor type 1	PTP1B	P18031	ENSG0000(	Signalling	BT006752	Familial hyperinsulinism, Congenital isolated hyperinsulinism, Type II diabetes mellitus, Obesity, B-Cell Lymphoma, Schizophrenia, Hodgkin's disease, Rett Syndrome, Inborn errors of metabolism, Alzheimer's disease	https://www.targetvalidation.org/target/ENSG00000196396/associations	CDK12 CDK19 CDK20 NTRK2 NTRK3 CDK10 PDK1 CDK13 GRAP CDK18 PRKCE CDK17 CDK16 CDK5 CDK6 CDK3 CSNK2A1 GRB2 BCAR1 EPHB3 PLK1 CDK9 CDK7 GSK3B CLK2 CLK1 CDK8 AKT2 AKT1	Endoplasmic reticulum membrane	Yes	https://www.bindingdb.org/uniprot/P18031	✓
PTPN11	HUMAN Tyrosine-protein phosphatase non-receptor type 11	BPTP3, NS1, PTP2C, SH-PTP2, SHP-2, SHP2, PTN11	Q06124	ENSG0000(	Cancer	L08807	Noonan Syndrome, LEOPARD Syndrome, Juvenile myelomonocytic leukemia, Metachondromatosis, Enchondromatosis, Fetal growth retardation, Leukemia, Hypertrophic Cardiomyopathy, Atrial septal defect, Cafe-au-Lait spots	https://www.targetvalidation.org/target/ENSG00000179295/associations	MAP3K4 SIT1 SIGLEC7 GAB2 ALK PILRB MAP3K5 SIGLEC10 INPP5D FRS2 IL22RA1 KIR2DL5A BTLA NTRK2 ERBB4 PTK2B GAB1 SLAMF1 DDR1 SOS1 PTK2 TEK IRF8 MS4A2 SIRPA GRB2 JAK3 PXN PIK3CG LEPR CRKL KIR3DL1	Cytoplasm	Yes	https://www.bindingdb.org/uniprot/Q06124	✓
PTPN2	HUMAN Tyrosine-protein phosphatase non-receptor type 2	PTPT, TC-PTP, TCPTP	P17706	ENSG0000(	Others	EF445017	Rheumatoid arthritis, Crohn's disease, Ulcerative colitis, Juvenile rheumatoid arthritis, Iridocyclitis, Type I diabetes mellitus, Esophageal carcinoma, Celiac disease, Polycythemia vera, Gaucher disease type 1	https://www.targetvalidation.org/target/ENSG00000175354/associations	TRAF2 CDK5 ITGA1 JAK3 CDK2 JAK1 SRC PDGFRB CDK1 INSR EGFR GJA1 FKBP4 CAV1 EGFR SRC FYN STAT5A JAK1 STAT6 SHC1 PDGFRB STAT1 MET INSR WASL STAT3 KDR JAK3 STAT5B KPNB1	Endoplasmic reticulum	Yes	https://www.bindingdb.org/uniprot/P17706	✓
PTPN20	HUMAN Tyrosine-protein phosphatase non-receptor type 20	PTPN20A, PTPN20B, PTN20	Q4JDL3	ENSG0000(	Others	AY704144	Tongue squamous cell carcinoma, Astrocytoma, Glioblastoma multiforme, Li-Fraumeni syndrome, Ependymoma, Isolated scaphocephaly, Medulloblastoma, Sjogren's syndrome, Primitive neuroectodermal tumor, Rosacea	https://www.targetvalidation.org/target/ENSG00000204179/associations		Nucleus	No		✓

PTPN22	HUMAN Tyrosine-protein phosphatase non-receptor type 22	Lyp, Lyp1, Lyp2, PTPN8, PTN22	Q9Y2R2	ENSG0000(	Others	AF077031	Arthritis, Rheumatoid arthritis, Type I diabetes mellitus, Autoimmune disease, Systemic lupus erythematosus, Graves disease, Vitiligo, Inflammatory bowel disease, Granulomatosis with polyangiitis, Giant cell arteritis	https://www.targetvalidation.org/target/ENSG0000134242/associations	TRAF3 PRKCD CSK PSTPIP1 LCK CBL ZAP70 CD247	Cytoplasm		Yes	https://www.bindingdb.org/uniprot/Q9Y2R2	✓
PTPN4	HUMAN Tyrosine-protein phosphatase non-receptor type 4	PTPN4	P29074	ENSG0000(	Cancer	M68941	Breast carcinoma, Septic shock, Acute megakaryocytic leukemia, Autism spectrum disorder, Hematologic neoplasm, Rett syndrome, Pituitary disease, Myelofibrosis, Acromegaly, Acute myelocytic leukemia	https://www.targetvalidation.org/target/ENSG00000088179/associations	CRK PRSS1 TRIM24 MAPK12 CD247 GRIN2A GRID2	Cell membrane		Yes	https://www.bindingdb.org/uniprot/P29074	✓
PTPN6	HUMAN Tyrosine-protein phosphatase non-receptor type 6	HCP, HCPH, PTP1C, SHP-1, SHP1, PTN6	P29350	ENSG0000(	Cancer	U15528	Autoimmune lymphoproliferative syndrome, Hyper-IgM syndrome with susceptibility to opportunistic infection, Schizophrenia, Calcinosis, Heart valve disease, Multiple sclerosis, Multiple myeloma, Sezary syndrome, Spondyloenchondrodysplasia, Li-Fraumeni syndrome	https://www.targetvalidation.org/target/ENSG00000111679/associations	MAP3K4 SIGLEC9 SIGLEC7 GAB2 PILRA PILRB TLR10 DOK1 SIGLEC10 BLNK LILRB1 LILRB4 LILRB2 KIR2DL5A LAIR1 PDCD1 PTK2B LCP2 KHDRBS1 PRKCD IRF8 SMAD3 GRB2 JAK3 KIR3DL1 KIR2DL3 KIR2DL1 SYK ZAP70	Cytoplasm, Nucleus,		Yes	https://www.bindingdb.org/uniprot/P29350	✓
PTRH1	HUMAN Probable peptidyl-tRNA hydrolase	C9orf115	Q86Y79	ENSG0000(	Others	BC047012	Ovarian carcinoma, Glioblastoma multiforme, Pancreatic carcinoma, Osteosarcoma, Hypoparathyroidism, Familial hypophosphatemic ricket, Paroxysmal kinesigenic choreoathetosis, X-linked Vitamin D-Resistant rickets, X-Linked hypophosphatemic rickets, Type I Thanatophoric dysplasia	https://www.targetvalidation.org/target/ENSG00000187024/associations				No		✓
PTTG1IP	HUMAN Pituitary tumor-transforming gene 1 protein-interacting protein	C21orf1, C21orf3, PBF	P53801	ENSG0000(	Others	AL163300	Breast carcinoma, Lung carcinoma, Non-small cell lung carcinoma, Thyroid neoplasm, Pituitary neoplasm, Differentiated thyroid gland carcinoma, Sarcoma, Osteosarcoma, Glioma, Astrocytoma	https://www.targetvalidation.org/target/ENSG00000183255/associations	SRC TP53 PTTG1	Membrane	Transmembrane	No		✓
PXK	HUMAN PX domain-containing protein kinase-like protein	FLJ20335	Q7Z7A4	ENSG0000(	Kinase	AY847222	Autoimmune disease, Systemic lupus erythematosus, Breast carcinoma, Ovarian carcinoma, Rheumatoid arthritis, Systemic scleroderma, IGA Glomerulonephritis, Pick disease, Endometrial neoplasm, Astrocytoma	https://www.targetvalidation.org/target/ENSG00000168297/associations	PRKCB PRKACA TRIP10 MTA1 NFATC4 PRKD2	Cytoplasm		No		✓
PXN	HUMAN Paxillin	PAXI	P49023	ENSG0000(	Others	D86863	Glioma, Hepatocellular carcinoma, Non-small cell lung carcinoma, Liver carcinoma, Osteosarcoma, Ovarian neoplasm, Renal cell carcinoma, Hermansky-Pudlak syndrome, Hyperplasia, Adenoid cystic carcinoma	https://www.targetvalidation.org/target/ENSG00000089159/associations	TLN1 GIT1 ASAP1 TUBA8 PARVA SORBS1 PTPRO MAPK14 PTPRR PTK2B PTK6 ITGA9 GAB1 ILK PAK2 PAK1 PTPN11 PTK2 PTPN12 CDK5 TUBA1B PPP2CA PPP2CB BCAR1 EPHB1 LIMK1 GSK3B GSK3A CRKL CRK MAPK8 SYK TEC	Cytoplasm, cytoskeleton		No		✓
PYCR1	HUMAN Pyrroline-5-carboxylate reductase 1, mitochondrial	P5CR1	P32322	ENSG0000(	Cancer,Cancer-Antigen	CR541769	Cutis laxa, Inborn errors of metabolism, Autosomal recessive cutis laxa type 2, Autosomal recessive cutis laxa type 2B, Premature aging, Geroderma osteodysplastica, Osteopenia, De barsy syndrome, PYCR1-related De Barsy syndrome, Prostate carcinoma	https://www.targetvalidation.org/target/ENSG00000183010/associations	CDK2 PARK7	Mitochondrion		Yes	https://www.drugbank.ca/biodb/polypeptides/P32322	✓

PYGB	HUMAN Glycogen phosphorylase, brain form	PYGB	P11216	ENSG0000(	Signalling	BC017045	Myocardial ischemia, Adenoid cystic carcinoma, Salivary gland neoplasm, Adenocarcinoma, Non-small cell lung carcinoma, Intracranial hemorrhage, Subarachnoid hemorrhage, Cerebrovascular accident, Cerebral hemorrhage, Asthma	https://www.targetvalidation.org/target/ENSG00000100994/associations	LMTK2 PHKG1 PHKG2 JAK2 ACTN1		Yes		✓
												https://www.bindingdb.org/uniprot/P11216	
PYGO2	HUMAN Pygopus homolog 2	PYGO2	Q9BRQ0	ENSG0000(	Transcription factor	BC032099	Isolated anophthalmia - microphthalmia, Colobomatous microphthalmia, Non-syndromic congenital cataract, Microphthalmia-cataract, Cockayne syndrome, Ovarian neoplasm, Glioma, Breast carcinoma, Glioblastoma, Adams-Oliver syndrome	https://www.targetvalidation.org/target/ENSG00000163348/associations	CREBBP AKT1	Nucleus	No		✓
RAB38	HUMAN Ras-related protein Rab-38	NY-MEL-1	P57729	ENSG0000(	Others	BC015808	Prostate carcinoma, Frontotemporal dementia, Griscelli disease, Hermanski-Pudlak Syndrome, Oculocutaneous albinism, Frontotemporal dementia, Segmental vitiligo, Melanoma, Alzheimer's disease, Hypopigmentation disorder	https://www.targetvalidation.org/target/ENSG00000163348/associations	TGFBR1 BMPR1B	Cell membrane	No		✓
RABEP1	HUMAN Rab GTPase-binding effector protein 1	RAB5EP, rabaptin-5, RABPT5, RABPT5A	Q15276	ENSG0000(	Cancer	BC041700	Melanoma, Breast carcinoma, Chronic myelomonocytic leukemia, Gastric neoplasm, Myeloproliferative disease, Mammary neoplasm, Myelodysplastic syndrome, Cutaneous melanoma, Rheumatoid arthritis, Basal cell carcinoma	https://www.targetvalidation.org/target/ENSG00000029725/associations	PRKD1 HUNK CASP3 RAB5A RAB4A GAP43 KCNH1 RABGEF1 RAB5A GGA1 GGA3	Cytoplasm, Early endosome, Recycling endosome, Cytoplasmic vesicle,	No		✓
RABL6	HUMAN Rab-like protein 6	C9orf86, Parf, RBEL1	Q3YEC7	ENSG0000(	Others	GQ169126	Melanoma, Osteocarcinoma, Breast carcinoma, T-cell acute lymphoblastic leukemia, Retinoblastoma, Hepatocellular carcinoma, Vulvar intraepithelial neoplasia, Pancreatic adenocarcinoma	https://www.targetvalidation.org/target/ENSG00000196642/associations		Cytoplasm	No		✓
RAC1	HUMAN Ras-related C3 botulinum toxin substrate 1	p21-Rac1, Rac-1, TC-25	P63000	ENSG0000(	Signalling, Cancer	AJ132695	Melanoma, Liver carcinoma, Depressive disorder, Diabetes mellitus, Heart failure, Dilated cardiomyopathy, HIV infection, Osteosarcoma, Inflammatory bowel disease, Lymphoid leukemia,	https://www.targetvalidation.org/target/ENSG00000136238/associations	MAP3K4 DAAM1 VAV3 PAK5 HACD3 ARHGEF4 PAK6 PARD6A NOD2 DEF6 PARD6B PARD6G MYD88 DVL3 ARHGEF2 DOCK2 NGEF NOXA1 CDC42BPG MAP3K11 RALBP1 ARHGEF6 DOCK1 ARHGEF7 KEAP1 RASGRF1 PAK2 PAK1	Cell membrane	Yes		✓
												https://www.bindingdb.org/uniprot/P63000	
RAC2	HUMAN Ras-related C3 botulinum toxin substrate 2	EN-7	P15153	ENSG0000(	Signalling	BT006919	Neutrophil immunodeficiency syndrome, Ovarian carcinoma, Juvenile myelomonocytic leukemia, Melanoma, Immunologic deficiency syndrome, Hodgkin's lymphoma, Glioma, Astrocytoma, Autoimmune lymphoproliferative syndrome, Autosomal agammaglobulinemia	https://www.targetvalidation.org/target/ENSG00000128340/associations	DOCK2 SH3RF1 PAK1 ARHGDIA RAP1GDS1 S100A9 S100A8 PLD2 CUL1 CDC42 NOXA1 PAK1 NCF2 RTKN CYBB ARHGDIB	Cytoplasm,	Yes		✓
												https://www.drugbank.ca/biodb/polypeptides/P15153	
RAD21	HUMAN Double-strand-break repair protein rad21 homolog	hHR21, KIAA0078, SCC1	O60216	ENSG0000(	Cancer	AK289505	Cornelia de Lange syndrome, Lymphoid neoplasm, Broad flat nasal bridge, Anteverted nostril, Absent external auditory canals, Hypoplastic feet, Generalized hirsutism, Papillary renal cell carcinoma, Myelodysplastic syndrome, Lung carcinoma	https://www.targetvalidation.org/target/ENSG00000164754/associations	PRSS21 SMC3 STAG1 STAG2 WAPL ESCO1 SMC1A ESPL1 PLK1 CAPN1 CTCF SMC1A SMC3 IL7R STAG2 FLNB STAG1	Nucleus	No		✓

RAD23A	HUMAN UV excision repair protein RAD23 homolog A	HHR23A	P54725	ENSG0000(	Signalling, Cancer	AB209713	Psoriasis, Drug-induced agranulocytosis, Squamous cell carcinoma, Xeroderma pigmentosum, DNA Damage, Melanoma, Multiple sclerosis, Dermatomyositis, Ovarian carcinoma, Duchenne muscular dystrophy	https://www.targetvalidation.org/target/ENSG00000179262/associations	UBE3A XPC UBC RPN1 UBE4B TP53 RAD23B TRAF2 PSMD4 NGLY1 DDI1	Nucleus,	No		✓
RAD23B	HUMAN UV excision repair protein RAD23 homolog B	HHR23B, HR23B, RD23B	P54727	ENSG0000(	Signalling, Cancer	AK125226	Ankyloblepharon - ectodermal defects - cleft lip/palate, EEC syndrome, Focal facial dermal dysplasia, Dyskeratosis congenita, Craniofacial abnormalities, Male infertility, Liver carcinoma, Multiple myeloma, Follicular lymphoma, Peutz-Jeghers syndrome	https://www.targetvalidation.org/target/ENSG00000119318/associations	UBQLN1 DYRK4 PSMD1 PSMB7 ADRM1 STK38 PSMD6 TDG PSMD2 UBE3A XPC PSMC6 PSMC5 PSMD4 RAD23A PSMC2 MAPK1 ERCC3 UBC CETN2 TDG XPC DBI PSMD1 PSMD6 ERCC3 UBE3A	Nucleus, Cytoplasm,	No		✓
RAD51	HUMAN DNA repair protein RAD51 homolog 1	FANCR, HsRad51, RAD51A, RECA	Q06609	ENSG0000(	Signalling, Cancer	AF233744	Breast carcinoma, Familial congenital mirror movements, Breast neoplasm, Mammary neoplasm, Fanconi anemia, Mental retardation, Hypopigmented skin patch, Hereditary breast cancer, Astrocytoma, Large cell lung carcinoma	https://www.targetvalidation.org/target/ENSG00000051180/associations	FANCD2 BARD1 PALB2 MDC1 DMC1 ATM PRKAA1 BTK E2F1 CSNK2A1 UBE2I SUMO1 CASP7 BLM PLK1 BRCA2 ATRX RAD52 CASP3 BRCA1 IRS1 RPA3 RPA1 ERCC2 H2AFX RPA2 TP53 ABL1 RECQL5 POLQ RAD51C CHEK1 RPA1	Nucleus	Yes	https://www.bindingdb.org/uniprot/Q06609	✓
RAD51B	HUMAN DNA repair protein RAD51 homolog 2	R51H2, RAD51L1, REC2	O15315	ENSG0000(	Cancer	BX161515	Mammary neoplasm, Breast carcinoma, Hamartoma, Glioma, Multiple sclerosis, Squamous cell carcinoma, Adenocarcinoma, Primary biliary cirrhosis, Ovarian endometroid carcinoma, Colonic neoplasm	https://www.targetvalidation.org/target/ENSG00000182185/associations	H1F0 XRCC2	Nucleus	No		✓
RAD51D	HUMAN DNA repair protein RAD51 homolog 4	R51H3, RAD51L3, Trad	O75771	ENSG0000(	Kinase,Cancer	AB016223	Ovarian carcinoma, Breast carcinoma, Familial ovarian cancer, Hereditary Breast and Ovarian cancer syndrome, Bloom syndrome, Cervical carcinoma, Fanconi anemia, Juvenile dermatomyositis, Rheumatoid arthritis, Chronic lymphocytic leukemia	https://www.targetvalidation.org/target/ENSG00000185379/associations	XRCC3 XRCC2	Nucleus Cytoplasm, cytoskeleton, microtubule organizing center, centrosome, Chromosome, telomere,	No		✓
RAF1	HUMAN RAF proto-oncogene serine/threonine-protein kinase	c-Raf, CRAF, Raf-1	P04049	ENSG0000(	Kinase,Cancer	EU332868	Rare familial disorder with hypertrophic cardiomyopathy, Noonan syndrome, Dilated cardiomyopathy, Auditory system disease, LEOPARD syndrome, Glioma, Cardiac arrhythmia, Pilocytic astrocytoma, Auditory system disease, Kidney neoplasm	https://www.targetvalidation.org/target/ENSG00000132155/associations	SHOC2 SPRY4 WDR83 BAG1 MAP3K5 TSC22D3 ERBIN PBK CNKSR1 BAD CNKSR2 CERK KSR1 KSR2 PKN1 RHEB PDK1 PPP2R5D PRKG1 PIN1 GRB10 MAP3K1 STK3 PAK2 MAPK7 PAK1 PRKAA1 DMPK PRKCD	Cytoplasm, Cell membrane, Mitochondrion, Nucleus,	Yes	https://www.bindingdb.org/uniprot/P04049	✓
RAG1	HUMAN V(D)J recombination-activating protein 1	RNF74	P15918	ENSG0000(	Others	AC139427	Primary immunodeficiency, Combined T and B cell immunodeficiency, Severe combined immunodeficiency, T-B-severe combined immunodeficiency, Omenn syndrome, Septicemia, Chronic diarrhea, Exfoliative dermatitis, Sarcoma, Hyper-IgM syndrome type 5	https://www.targetvalidation.org/target/ENSG00000166349/associations	RAG2 HMGB2 UBC KPNA2 HIST3H3 MTOR KPNA1 RAG2	Nucleus	No		✓
RALBP1	HUMAN RalA-binding protein 1	RLIP1, RLIP76	Q15311	ENSG0000(	Cancer,Cancer-Antigen	CH471113	Pancreatic carcinoma, Lung carcinoma, Myocardial ischemia, Epilepsy, Glioblastoma, Prostate neoplasm, Atherosclerosis, Obesity, Neuroblastoma, Melanoma	https://www.targetvalidation.org/target/ENSG00000017797/associations	REPS2 CASP8 RAC1 TNFRSF1A PRKCA RALB RALA TLR3 RALA CDC42 RAC1 SYNJ2BP AP2M1 REPS1 EPN1	Membrane	Yes	https://www.drugbank.ca/biodb/polypeptides/Q15311	✓

RAN	HUMAN GTP-binding nuclear protein Ran	ARA24, Gsp1, TC4	P62826	ENSG0000(	Cancer,Cancer-Antigen	AF501887	HIV infection, Pancreatic carcinoma, Degenerative polyarthritis, Keloid, Sciatic neuropathy, Renal cell carcinoma, Kidney neoplasm, Nasopharyngeal neoplasm, Amyotrophic lateral sclerosis	https://www.targetvalidation.org/target/ENSG00000132341/associations	NUP50 XPO5 RANBP9 NEK9 KPNB1 NUTF2 PLK1 RANBP2 NUP153 RANGAP1 RANBP1 WAS TGFBR1 RCC1 AR ISG15 ABL1 PAK4 IPO13 XPOT XPO1 BMPR1B NFKBIA AR MAD2L2 XPO1 KPNB1 RANBP3 XPO5 NUTF2	Nucleus	Yes	https://www.drugbank.ca/biodb/polypeptides/P62826	✓
RAP1GDS1	HUMAN Rap1 GTPase-GDP dissociation stimulator 1	SmgGDS	P52306	ENSG0000(	Cancer	AC019077	Melanoma, Esophageal carcinoma, Lung carcinoma, Breast Carcinoma, Non-small cell lung carcinoma, Acute lymphocytic leukemia, Prostate carcinoma, Cutaneous melanoma, Gastric intestinal type adenocarcinoma, Osteosarcoma	https://www.targetvalidation.org/target/ENSG00000138698/associations	KIFAP3 RHOA RAC2 CDC42 RAC1 RAP1A RAP1B KRAS RHOB RHOC		No		✓
RAPGEF4	HUMAN Rap guanine nucleotide exchange factor 4	cAMP-GEFII, CGEF2, EPAC2	Q8WZA2	ENSG0000(	Kinase,Signalling	AK296340	Central nervous system cancer, Glioma, Astrocytoma, Autistic disorder, Mental depression, Depressive disorder, Left ventricular hypertrophy, Brugada syndrome, Naxos disease, Familial atrial fibrillation	https://www.targetvalidation.org/target/ENSG00000091428/associations	RIMS1 RIMS1 RIMS2	Cytoplasm, Membrane	Yes	https://www.bindingdb.org/uniprot/Q8WZA2	✓
RARA	HUMAN Retinoic acid receptor alpha	NR1B1, RAR	P10276	ENSG0000(	Transcription factor,Cancer	FJ487576	Lymphoid neoplasm, Acute promyelocytic leukemia, Liver carcinoma, Fibroadenoma, Schizophrenia, Phyllodes tumor, Somatic mutation, Sciatic neuropathy, Bipolar disorder, Psoriasis	https://www.targetvalidation.org/target/ENSG00000131759/associations	CREBBP ESR2 NCOA2 NR0B2 EP300 SUMO2 MAPK10 CCNH CDK7 NRIP1 MAPK9 MAPK8 CSK AKT1 RXRB MAPK1 RXRA PRKACA PRKCA SRC THRA SP1 ESR1 RPS6KA5 NCOR1 ACTN4 TRIM24 CTNNB1 EGFR GNAQ NCOR2 E2F1	Nucleus, Cytoplasm,	Yes	https://www.bindingdb.org/uniprot/P10276	✓
RARB	HUMAN Retinoic acid receptor beta	HAP, NR1B2, RRB2	P10826	ENSG0000(	Transcription factor,Cancer	BC060794	Syndromic microphthalmia, Matthew-Wood syndrome, Psoriasis, Eczema, Anophthalmos, Adenocarcinoma, Congenital diaphragmatic hernia, Microphthalmos, Rosacea, Atopic eczema	https://www.targetvalidation.org/target/ENSG00000077092/associations	PRKD2 NR0B2 RXRB RXRA THRA NCOR2 NCOA3 RXRA THRA RXRB RXRG	Nucleus, Cytoplasm,	Yes	https://www.bindingdb.org/uniprot/P10826	✓
RB1	HUMAN Retinoblastoma-associated protein	OSRC, RB	P06400	ENSG0000(	Transcription factor,Cancer	L41870	Retinoblastoma, Osteosarcoma, Small celll lung carcinoma, Liver carcinoma, Bladder neoplasm, Lymphoma, Fallopian tube serous adenocarcinoma, Retinal cancer, Pancreatic neoplasm, Prostate carcinoma	https://www.targetvalidation.org/target/ENSG00000139687/associations	MORF4L1 CDK12 SMYD2 CDK19 RBBP8 AURKB SIRT1 HDAC2 CDK20 PPP1R26 MAPK14 E2F4 CDK10 IRF3 E2F2 TFDP2 TFDP1 CDK13 HDAC1 SKP2 PRKAA1 EP300 CDK18 ID2 E2F1 MDM2 CDK17 CDK16 CDK5 CDK6 CDK3	Nucleus	Yes	https://www.bindingdb.org/uniprot/P06400	✓
RBKS	HUMAN Ribokinase	RBSK	Q9H477	ENSG0000(	Kinase	AC021171	Schizophrenia, Pancreatic ductal adenocarcinoma, Tuberculosis, Chronic lymphocytic leukemia, Hepatocellular carcinoma, Spina bifida, Glioblastoma multiforme, Sepsis, Contagious pustular dermatitis, Nasopharyngeal neoplasm	https://www.targetvalidation.org/target/ENSG00000171174/associations		Cytoplasm	No		✓
RBM28	HUMAN RNA-binding protein 28	FLJ10377	Q9NW13	ENSG0000(	Cancer,Cancer-Antigen	AC010655	Alopecia, ANE syndrome, Breast carcinoma, Skeletal muscle atrophy, Kyphoscoliosis deformity of spine, Pyramidal tract syndrome, Upper motor neuron dysfunction, Ovarian carcinoma, Multiple myeloma, Breast carcinoma	https://www.targetvalidation.org/target/ENSG00000106344/asso		Nucleus, nucleolus	No		✓
RBM46	HUMAN Probable RNA-binding protein 46	CT68, MGC27016	Q8TBY0	ENSG0000(	Others	CH471056	Li-Fraumeni syndrome, Prostate carcinoma, Azoospermia, Steinert myotonic dystrophy, Melanoma, Tuberculosis, Breast carcinoma	https://www.targetvalidation.org/target/ENSG00000151962/associations			No		✓

RBM6	HUMAN RNA-binding protein 6	DEF3, g16, NY-LU-12	P78332	ENSG0000(	Cancer	U50839	Leukemia, Obesity, Small cell lung carcinom, Prostate carcinoma, Acute Megakaryocytic Leukemia, Paraneoplastic Encephalomyelitis, Self-reported educational attainment, Astrocytoma, Lung carcinoma, Pancreatic ductal adenocarcinoma	https://www.targetvalidation.org/target/ENSG00000004534/associations		Nucleus	No		✓
RBM7	HUMAN RNA-binding protein 7	RBM7	Q9Y580	ENSG0000(	Others	CH471065	Asthma, Glioblastoma multiforme, Melanoma, Allergy, Lung carcinoma, Non-small cell lung carcinoma, Atopic eczema, Neuropathy, Meningeal tuberculosis, Sjogren's syndrome	https://www.targetvalidation.org/target/ENSG00000076053/associations	ZCCHC8		No		✓
RBMS1	HUMAN RNA-binding motif, single-stranded-interacting protein 1	C2orf12, MSSP, MSSP1, SCR2	P29558	ENSG0000(	Cancer,Cancer-Antigen	AC104598	Diabetes mellitus, Type II diabetes mellitus, Self-reported educational attainment, Mayer-Rokitansky-Küster-Hauser syndrome, Amyotrophic lateral sclerosis, Ataxia telangiectasia, Obesity, Hypogammaglobulinemia, X-linked agammaglobulinemia, Thyroid carcinoma	https://www.targetvalidation.org/target/ENSG00000153250/associations	POLA1	Nucleus,	No		✓
RBPJ	HUMAN Recombining binding protein suppressor of hairless	CBF1, IGKJRB, IGKJRB1, RBPJK, RBPSUH	Q06330	ENSG0000(	Transcription factor	L07876	Adams-Oliver syndrome, Rheumatoid arthritis, Inflammatory bowel disease, Cutis marmorata, Congenital scar, Aplasia cutis congenita, Microphthalmos, Talipes, Strabismus, Polyp	https://www.targetvalidation.org/target/ENSG00000168214/associations	NCOR2<br>HEY1<br>MAPK8IP1<br>NOTCH3<br>NOTCH4<br>SPEN<br>RITA1<br>MAML3<br>KAT2B<br>MAML1<br>MAML2<br>CIR1<br>MAPK14<br>HES1<br>FHL1<br>SNW1<br>HDAC1<br>FOXC1<br>NOTCH2<br>MECP2<br>NOTCH1<br>KDM5A<br>NCOR1<br>NCOR2<br>HES1<br>MAML1<br>NOTCH1<br>NCOR1<br>MAML3	Nucleus, Cytoplasm,	Yes	https://www.bindingdb.org/uniprot/Q06330	✓
RCAN1	HUMAN Calcipressin-1	DSCR1	P53805	ENSG0000(	Signalling	U85267	Hyper-IgM syndrome with susceptibility to opportunistic infection, Congenital left-sided heart lesion,<br>Down syndrome, Congenital heart defect, Huntington disease, Rheumatoid arthritis, Craniofacial abnormalities, Cardiomegaly, Alzheimer's disease, Chronic lymphocytic leukemia	https://www.targetvalidation.org/target/ENSG00000159200/associations	TRAF6<br>NLK<br>MAP3K3<br>MAP3K14<br>MAPK15<br>MAPK6<br>DYRK1A<br>MAPK7<br>PPP3CA<br>MAPK10<br>MAP2K6<br>GSK3B<br>GSK3A<br>PPP3CC<br>MAPK9<br>MAPK8<br>MAPK4<br>MAPK1<br>MAPK3<br>PRKACB<br>PRKACA<br>PPP3CB<br>PPP3CA<br>IRAK1<br>USP22<br>BTRC UXT<br>TOLLIP		No		✓
RCC2	HUMAN Protein RCC2	TD-60	Q9P258	ENSG0000(	Others	BC042141	Familial idiopathic steroid-resistant nephrotic syndrome, Blackfan-Diamond anemia, Familial hemophagocytic lymphohistiocytosis, Juvenile polyposis syndrome, Colobomatous microphthalmia, Melanoma, Basal cell carcinoma, Rectal carcinoma, Marinesco-Sjogren's syndrome, Colorectal cancer	https://www.targetvalidation.org/target/ENSG00000179051/associations	RAC1	Nucleus, nucleolus, Chromosome, centromere<br>,<br>Cytoplasm, cytoskeleton, spindle,	No		✓
REG3A	HUMAN Regenerating islet-derived protein 3-alpha	HIP, PAP, PAP1, PBCGF, REG-III, REG3	Q06141	ENSG0000(	Others	X68641	Inflammatory bowel disease, Crohn's disease, Chronic pancreatitis, Type II diabetes mellitus, Seizures,<br>Sciatic neuropathy, Liver failure, Alzheimer's disease, Liver carcinoma, Cystic fibrosis	https://www.targetvalidation.org/target/ENSG00000172016/associations	SIPA1<br>FIP1L1<br>SSU72	Secreted	No		✓
RERG	HUMAN Ras-related and estrogen-regulated growth inhibitor	MGC15754	Q96A58	ENSG0000(	Others	AC007543	Chronic obstructive pulmonary disease, Breast carcinoma, Marinesco-Sjogren's syndrome, Mammary neoplasm, Lung adenocarcinoma, Ovarian carcinoma, Astrocytoma, Pituitary cancer, Glioblastoma multiforme, Medulloblastoma	https://www.targetvalidation.org/target/ENSG00000134533/associations		Cytoplasm	No		✓

RET	HUMAN Proto-oncogene tyrosine-protein kinase receptor Ret	CDHF12, CDHR16, HSCR1, MEN2B, PTC, RET51	P07949	ENSG0000(	Kinase,Cancer	X15262	Multiple endocrine neoplasia, Hirschsprung disease, Pheochromocytoma, Multiple endocrine neoplasia type 2a, Multiple endocrine neoplasia type 2b, Familial medullary thyroid carcinoma, Congenital central hypoventilation, Thyroid neoplasm, Multiple endocrine neoplasia, Hirschsprung disease	https://www.targetvalidation.org/target/ENSG00000165731/associations	IKBKG CBLC NRTN DOK1 SHC3 DOK4 DOK6 DDR2 MAPK14 GAB1 CBLB PTPRJ TNK2 PTK2 GRB2 GFRA1 MAPK9 MAPK8 GDNF MAPK1 MAPK3 CBL PRKACA PRKCA SRC PTPRF AIP PRKAR2A AKAP5	Cell membrane	Transmembrane	Yes		✓
													https://www.bindingdb.org/uniprot/P07949	
RET_ext	Proto-oncogene tyrosine-protein kinase receptor Ret	CDHF12, CDHR16, PTC, RET51	P07949			AC010864								✓
RET_int	Proto-oncogene tyrosine-protein kinase receptor Ret	CDHF12, CDHR16, PTC, RET51	P07949			AC010864								✓
REXO4	HUMAN RNA exonuclease 4	hPMC2, XPMC2H	Q9GZR2	ENSG0000(	Transcription factor	AK025493	Breast carcinoma, Melanoma, Medulloblastoma, Tuberous sclerosis, Congenital heart defect	https://www.targetvalidation.org/target/ENSG00000148300/associations		Nucleus, nucleolus		No		✓
RFK	HUMAN Riboflavin kinase	RIFK	Q969G6	ENSG0000(	Kinase	AL391868	Lung carcinoma, Small cell lung carcinoma, Non-small cell lung carcinoma, Breast carcinoma, Stroke, Melanoma, Glioblastoma multiforme, Renal cell carcinoma, Alzheimer's disease, Astrocytoma	https://www.targetvalidation.org/target/ENSG00000135002/associations		Cytoplasm		Yes		✓
													https://www.drugbank.ca/biodb/polypeptides/Q969G6	
RFX3	HUMAN Transcription factor RFX3	RFX3	P48380	ENSG0000(	Transcription factor	AL365202, AL133549, AL354941	Gout, Hypertrophic cardiomyopathy, Arrhythmogenic right ventricular dysplasia, Familial restrictive cardiomyopathy, Familial isolated arrhythmogenic right ventricular dysplasia, Ependymoma, Ciliopathies, Agenesis of corpus callosum, Ventricular septal defect, COG7-CDG	https://www.targetvalidation.org/target/ENSG00000080298/associations		Nucleus		No		✓
RGS5	HUMAN Regulator of G-protein signaling 5	RGS5	O15539	ENSG0000(	Others	AJ891044	Hypertensive disease, Schizophrenia, Atherosclerosis, Chronic obstructive pulmonary disease, Renal carcinoma, Clear cell renal carcinoma, Osteosarcoma, Colorectal adenocarcinoma, Inflammatory skin disease, Lung adenocarcinoma	https://www.targetvalidation.org/target/ENSG00000143248/associations		Cytoplasm		No		✓
RHOA	HUMAN Transforming protein RhoA	ARH12, ARHA, Rho12, RhoA, RHOH12	P61586	ENSG0000(	Signalling, Cancer	BT019870	Lymphoid neoplasm, Adenocarcinoma, Gastric neoplasm, Angioimmunoblastic lymphadenopathy, Peripheral T-Cell Lymphoma, Proteinuria, Bladder neoplasm, Congenital diaphragmatic hernia, Burkitt lymphoma, Melanoma	https://www.targetvalidation.org/target/ENSG00000067560/associations	RIPOR2 DAAM1 VAV3 RABAC1 ARHGEF12 ARHGAP35 ARHGEF3 ARHGEF4 FZD3 SMURF1 ECT2 DEF6 RTKN ARHGDIG ARHGEF2 ARHGEF1 NGEF ARHGEF25 SRGAP1 NET1 ARHGEF18 PKN2 PKN1 GNA13 PRKG1 RASGRF1 ROCK1	Cell membrane, Lipid-anchor, Cytoplasmic side, Cytoplasm, cytoskeleton, Cleavage furrow, Cytoplasm, cell cortex		Yes		✓
													https://www.bindingdb.org/uniprot/P61586	
RHOB	HUMAN Rho-related GTP-binding protein RhoB	ARH6, ARHB, MST081, RhoB, RHOH6	P62745	ENSG0000(	Cancer	AK124398	Lung carcinoma, Breast carcinoma, Renal cell carcinoma, Systemic scleroderma, Degenerative polyarthritis, Glioblastoma, Osteoarthritis, Glioma, Psoriasis, Dermatomyositis	https://www.targetvalidation.org/target/ENSG00000143878/associations	ARHGEF3 ARHGDIG ARHGDIA RAP1GDS1 CSNK1A1 PDE6D DIAPH1 RHPN2	Late endosome membrane, Lipid-anchor, Cell membrane, Lipid-anchor, Nucleus, Cleavage furrow,		Yes		✓
													https://www.drugbank.ca/biodb/polypeptides/P62745	
RHOH	HUMAN Rho-related GTP-binding protein RhoH	ARHH, RhoH, TTF	Q15669	ENSG0000(	Cancer	NA	Adenocarcinoma, Squamous cell carcinoma, B-Cell lymphoma, Follicular leukemia, Epidermodysplasia verruciformis, Primary cutaneous follicle center lymphoma, Primary cutaneous marginal zone B-cell lymphoma, Thyroid lymphoma, Crohn's disease, Glioblastoma multiforme	https://www.targetvalidation.org/target/ENSG00000168421/associations	TGFBR1 SRC SYK	Cytoplasm		No		✓

RHOT2	HUMAN Mitochondrial Rho GTPase 2	ARHT2, C16orf39, MIRO-2	Q8IXI1	ENSG0000(	Others	AK090426	Rare hereditary hemochromatosis, Hemochromatosis type 2, Beta-Thalassemia, African iron overload, Familial hemophagocytic lymphohistiocytosis, Syndromic diarrhea, Parkinson's disease, Hereditary hemochromatosis, Familial benign copper deficiency, Glioblastoma multiforme	https://www.targetvalidation.org/target/ENSG00000140983/associations		Mitochondrion outer membrane	Transmembrane	No		✓
RHOXF2	HUMAN Rhox homeobox family member 2	PEPP-2, PEPP2, THG1	Q9BQY4	ENSG0000(	Transcription factor	BC021719	Li-Fraumeni syndrome, Breast carcinoma, Leukemia, Gastric neoplasm, Hematologic neoplasm, Azoospermia, Lung carcinoma, Chronic myelogenous leukemia, Gastric carcinoma, Male indertility	https://www.targetvalidation.org/target/ENSG00000131721/associations	ORC2	Nucleus		No		✓
RIDA	HUMAN 2-iminobutanoate/2-iminopropanoate deaminase	P14.5, UK114, HRSP12	P52758	ENSG0000(	Cancer,Cancer-Antigen	CR541652	Prion disease, Breast carcinoma, Pick disease, Acute myeloid leukemia, Breast carcinoma, Hepatitis C infection, Prostate adenocarcinoma, Progressive supranuclear palsy, Chronic calcifying pancreatitis, Pancreatic ductal adenocarcinoma	https://www.targetvalidation.org/target/ENSG00000132541/associations		Cytoplasm		Yes	https://www.drugbank.ca/biodb/polypeptides/P52758	✓
RING1	HUMAN E3 ubiquitin-protein ligase RING1	RNF1	Q06587	ENSG0000(	Transcription factor	AL031228	Autosomal recessive spondylocostal dysostosis, Spondyloepiphyseal dysplasia tarda, Orofaciodigital syndrome type 11, Smith-McCort dysplasia, Renal cell carcinoma, Systemic lupus erythematosus, Type I diabetes mellitus, Prostate carcinoma, Pseudoachondroplasia, Cervical adenocarcinoma	https://www.targetvalidation.org/target/ENSG00000204227/associations	FHL1 UBE2D1 BMI1 CBX7 E2F6 H2AFZ RYBP FHL1 PCGF2 PHC1 CBX2	Nucleus		No		✓
RIOK2	HUMAN Serine/threonine-protein kinase RIO2	FLJ11159	Q9BVS4	ENSG0000(	Kinase	AC008883	Lung carcinoma, Acute lymphoblastic leukemia, Pregnancy complication, Glioblastoma, Hypertensive disease, Pre-Eclampsia, Gastric carcinoma, Trypanosoma cruzi seropositivity, Multiple sclerosis, Chronic myelogenous leukemia	https://www.targetvalidation.org/target/ENSG00000058729/associations	PLK1	Cytoplasm		Yes	https://www.bindingdb.org/uniprot/Q9BVS4	✓
RIOK3	HUMAN Serine/threonine-protein kinase RIO3	SUDD	O14730	ENSG0000(	Kinase	NA	Melanoma, Ovarian carcinoma, Lung carcinoma, Measles, Pancreatic neoplasm, Gaucher disease type 3, Progressive supranuclear palsy, Diverticulitis, Psoriasis, Dermatomyositis	https://www.targetvalidation.org/target/ENSG00000101782/associations	CASP10 IRF3 IFIH1 TBK1	Cytoplasm		Yes	https://www.bindingdb.org/uniprot/O14730	✓
RIPK1	HUMAN Receptor-interacting serine/threonine-protein kinase 1	RIP	Q13546	ENSG0000(	Kinase	AY682848	Colorectal cancer, Diffuse large B-Cell lymphoma, Follicular lymphoma, Rheumatoid arthritis, Non-Hodgkin's lymphoma, Renal cell carcinoma, Non-small cell lung carcinoma, Hepatocellular carcinoma, Pancreatitis, Hypertrophy	https://www.targetvalidation.org/target/ENSG00000137275/associations	IKBKG RIPK3 TRAF6 RNF11 DAXX TAB2 CYLD PIDD1 ZBP1 RBCK1 MAP3K3 MAP3K14 ITCH PELI1 OPTN IRF7 CASP10 TANK RFFL TRPC4AP CCDC50 TICAM1 TAX1BP1 MAVS TRADD CASP8 SQSTM1 BIRC2 BIRC3	Cytoplasm, Cell membrane		Yes	https://www.bindingdb.org/uniprot/Q13546	✓
RIPK2	HUMAN Receptor-interacting serine/threonine-protein kinase 2	CARD3, CARDIAK, RICK, RIP2	O43353	ENSG0000(	Kinase	AY358813	Leprosy, Primary immunodeficiency, Common variable immunodeficiency, Hyper-IgM syndrome with susceptibility to opportunistic infection, Hyper-IgM syndrome without susceptibility to opportunistic infection, Ulcerative colitis, Asthma, Peritonitis, Huntington disease, Multibacillary leprosy	https://www.targetvalidation.org/target/ENSG00000104312/associations	IKBKG TRAF6 NOD1 PYCARD NLRC4 NOD2 CARD9 CARD6 ERBIN PELI3 TICAM1 MAVS CARD16 TRIP6 IRF4 BIRC2 BIRC3 UBE2V1 TRAF3 TRAF2 XIAP UBE2N IRAK1 CASP1 NGFR MAP3K7 TLR4 TUBB MYH9	Cytoplasm		Yes	https://www.bindingdb.org/uniprot/O43353	✓

RNASEL	HUMAN 2-5A-dependent ribonuclease	PRCA1, RNS4	Q05823	ENSG0000(	Others	CH471067	Familial prostate cancer, Circadian rhythm, Chronic Fatigue Syndrome, Hepatitis C infection, West Nile viral infection, Lung carcinoma, Ovarian carcinoma, Cervical carcinoma, Colonic neoplasm, Li-Fraumeni syndrome	https://www.targetvalidation.org/target/ENSG00000135828/associations	IQGAP1 AR ABCE1	Cytoplasm	Yes	https://www.bindingdb.org/uniprot/Q05823	✓
RNF38	HUMAN E3 ubiquitin-protein ligase RNF38	RNF38	Q9H0F5	ENSG0000(	Others	AK093480	Azoospermia, Cone rod dystrophy, Lung carcinoma, Breast carcinoma, Gastric carcinoma,Small cell lung carcinoma, Diffuse large B-cell lymphoma, Autosomal dominany Emery-Dreifuss muscular dystrophy, Pancreatic carcinoma	https://www.targetvalidation.org/target/ENSG00000137075/associations		Nucleus	No		✓
RNF40	HUMAN E3 ubiquitin-protein ligase BRE1B	BRE1B, KIAA0661, RBP95	O75150	ENSG0000(	Cancer,Cancer-Antigen	BX640763	Prostate carcinoma, Breast carcinoma, Ovarian carcinoma, Retinoblastoma, Autistic disorder, Nephroblastoma, Schizophrenia, Astrocytoma, Ulcerative colitis, Autism	https://www.targetvalidation.org/target/ENSG00000103549/associations	RNF20 ATM RNF20 RB1	Nucleus	No		✓
RNF6	HUMAN E3 ubiquitin-protein ligase RNF6	DKFZp686P0776	Q9Y252	ENSG0000(	Others	AY009109	Esophageal squamous cell carcinoma, Melanoma, Acute lymphoblastic leukemia, Squamous cell carcinoma, Chest pain, Colorectal adenocarcinoma, Pelizaeus-Merzbacher disease, Scoliosis, Teratozoospermia, Glioblastoma multiforme, Multiple sclerosis	https://www.targetvalidation.org/target/ENSG00000127870/associations	LIMK1	Nucleus	No		✓
RNF7	HUMAN RING-box protein 2	RBX2, CKBBP1, ROC2, SAG	Q9UBF6	ENSG0000(	Others	AF312226	Pseudohypoaldosteronism type 2, Bartter syndrome, Infantile Bartter syndrome with sensorineural deafness, Coronary artery calcification, HIV infection, LCAT deficiency, Cystic fibrosis, Fish-eye disease, Hypobetelipoproteinemia, Liver cirrhosis	https://www.targetvalidation.org/target/ENSG00000114125/associations	IKBKG PAK5 CUL5 CUL1 CSNK2A1 CSNK2B CSNK2A2 SOCS3 CASP3	Cytoplasm	No		✓
RNGTT	HUMAN mRNA-capping enzyme	CAP1A	O60942	ENSG0000(	Others	AB009024	HIV infection, Colorectal cancer, Endometrial neoplasm, Alpha-Thalassemia, Beta-Thalassemia, Liver carcinoma, Colorectal cancer, Tuberculosis, Dysentery, Uveitis	https://www.targetvalidation.org/target/ENSG00000111880/associations	RNMT RNMT	Nucleus	Yes	https://www.drugbank.ca/biodb/polypeptides/O60942	✓
ROPN1	HUMAN Ropporin-1A	CT91, ROPN1A, ropporin	Q9HAT0	ENSG0000(	Others	CH471052	Azoospermia, Breast carcinoma, Non-syndromic male infertility due to sperm motility disorder, Male infertility with teratozoospermia due to single gene mutation, Deafness-infertility syndrome, Brain ischemia, Ischemic stroke, Ovarian carcinoma, Psoriasis, Li-Fraumeni syndrome, Colorectal adenocarcinoma	https://www.targetvalidation.org/target/ENSG00000065371/associations	SPA17		No		✓
RORC	HUMAN Nuclear receptor ROR-gamma	NR1F3, RORG, RZRG	P51449	ENSG0000(	Transcription factor	BC031554	Autoimmune disease, Inflammatory bowel disease, Primary immunodeficiency due to a defect in adaptive immunity, Ulcerative colitis, Lymphedema, Obesity, Wiskott-Aldrich syndrome, Severe congenital neutropenia, Lymphoma, Transcobalamin deficiency	https://www.targetvalidation.org/target/ENSG00000143365/associations	FOXP3 CHD4 EIF4EBP1 ARNTL	Nucleus	Yes	https://www.bindingdb.org/uniprot/P51449	✓

RPA2	HUMAN Replication protein A 32 kDa subunit	RFA2, REPA2, RPA32, RPA34	P15927	ENSG0000(	Cancer	AL109927	Stroke, Breast carcinoma, Multiple endocrine neoplasia, Multiple sclerosis, Ductal breast carcinoma, Colorectal cancer, Melanoma, Ataxia-telangiectasia, Glioblastoma multiforme, Hepatocellular carcinoma	https://www.targetvalidation.org/target/ENSG00000117748/associations	STK17A<br>PIAS4 ATR<br>ATM<br>RAD51<br>E2F1<br>CDK5<br>CDK6<br>PRKDC<br>PPP4C<br>CDK7<br>MAPK9<br>MAPK8<br>PRKCI<br>RPA3<br>WEE1<br>RPA1 XPA<br>UNG<br>CDK4<br>CDK1<br>PIAS1<br>HUS1<br>EIF4EBP3<br>MAP3K7<br>MCM5<br>BLM RPA1<br>HUS1<br>MCM3<br>RAD52<br>XPA	Nucleus	No		✓
RPL10	HUMAN 60S ribosomal protein L10	DXS648E, QM	P27635	ENSG0000(	Cancer,Cancer-Antigen	S64169,S64168	Autism spectrum disorder, Lymphoid neoplasm, Penile hypospadias, Facial deformity, Congenital camptodactyly, Single transverse palmar crease, Melanoma, Oral squamouc cell carcinoma, Osteosarcoma, Atopic eczema	https://www.targetvalidation.org/target/ENSG00000147403/associations	RPL36<br>MAP3K14<br>MAP3K1<br>RPL18<br>RPL6<br>RPL18A<br>RPL19<br>RPL24<br>RPS21<br>RPL38<br>RPL8<br>RPL11<br>RPL10A<br>RPL31<br>RPL30<br>RPS26<br>RPS25<br>RPS24<br>RPL23<br>RPS6<br>RPL23A<br>RPS4X<br>RPL7A<br>RPS11<br>RPS13<br>RPS18<br>RPS23<br>RPS14<br>RPS16		No		✓
RPL10A	HUMAN 60S ribosomal protein L10a	Csa-19, L10A, NEDD6	P62906	ENSG0000(	Cancer,Cancer-Antigen	BT020041	Diabetes mellitus, Chronic myelogenous leukemia, Adenocarcinoma, Adenoma, Focal glomerulosclerosis, Liver carcinoma	https://www.targetvalidation.org/target/ENSG00000198755/associations	RPL26L1<br>MAP3K14<br>MAP3K1<br>RPL18<br>RPL6<br>RPL18A<br>RPL19<br>RPL24<br>RPL8<br>RPL11<br>RPL31<br>RPL30<br>RPS26<br>RPS25<br>RPL23<br>RPS6<br>RPL23A<br>RPS4X<br>RPL7A<br>RPS11<br>RPS13<br>RPS23<br>RPS16<br>RPS15A<br>RPS8<br>RPL37A<br>RPL15<br>RPS3A<br>RPS20		No		✓
RPL11	HUMAN 60S ribosomal protein L11	L11	P62913	ENSG0000(	Others	BC018970	Blackfan-Diamond anemia, Breast carcinoma, Teratozoospermia, Migraine disorder, Triphalangeal thumb, Electrocardiogram abnormal, Diabetes mellitus, Psoriasis, Melanoma, Hypospadias	https://www.targetvalidation.org/target/ENSG00000142676/associations	RPL36<br>TBK1<br>MAP3K14<br>RPL10L<br>RPL18<br>RPL6<br>RPL18A<br>RPL19<br>RPL24<br>RPL38<br>RPL8<br>RPL10A<br>RPL31<br>RPL30<br>RPS26<br>RPS25<br>RPL23<br>RPS6<br>RPL23A<br>RPS4X<br>RPL7A<br>RPS11<br>RPS13<br>RPS23<br>RPS14<br>RPS16<br>RPS15A<br>RPS8<br>RPS7	Nucleus, nucleolus	Yes	https://www.drugbank.ca/biodb/polypeptides/P62913	✓
RPL13A	HUMAN 60S ribosomal protein L13a	L13A	P40429	ENSG0000(	Cancer,Cancer-Antigen	CH471177	Simpson-Golabi-Behmel syndrome, Ovarian carcinoma, Chronic lymphocytic leukemia, Cerebral ischemia, Cerebral infarction, Glioma, Cataract, Uveal melanoma, Breast carcinoma, Schizophrenia	https://www.targetvalidation.org/target/ENSG00000142541/associations	RPL8<br>RPL30<br>DAPK1<br>RPL14<br>RPL10<br>DAPK3<br>RPL10<br>RPL8<br>RPL30<br>RPL14	Cytoplasm	Yes	https://www.drugbank.ca/biodb/polypeptides/P40429	✓
RPL15	HUMAN 60S ribosomal protein L15	EC45, L15, RPL10, TCBAP0781	P61313	ENSG0000(	Others	AY347528	Blackfan-Diamond anemia, Adenocarcinoma, Pancreatic ductal adenocarcinoma, Macrocytic anemia, Electrocardiogram abnormal, Intermittent migraine headache, Reticulocytopenia, Cystic fibrosis, Lung carcinoma, Macroglobulinemia	https://www.targetvalidation.org/target/ENSG00000174748/associations	RPL36<br>RPL18<br>RPL6<br>RPL18A<br>RPL19<br>RPL24<br>RPL8<br>RPL11<br>RPL10A<br>RPL31<br>RPL30<br>RPS26<br>RPS25<br>RPL23<br>RPS6<br>RPL23A<br>RPS4X<br>RPL7A<br>RPS11<br>RPS13<br>RPS23<br>RPS14<br>RPS16<br>RPS15A<br>RPS8<br>RPS7<br>RPL37A<br>RPL26<br>RPS3A	Membrane	Yes	https://www.drugbank.ca/biodb/polypeptides/P61313	✓
RPL17	HUMAN 60S ribosomal protein L17	RPL17	P18621	ENSG0000(	Others	AK304659	Epithelial neoplasm, Lymphoid neoplasm, Myocardial ischemia, Systemic lupus erythematosus, Tuberculosis, Autoimmune disease, Type I diabetes mellitus, Melanoma, Breast carcinoma, Adenocarcinoma	https://www.targetvalidation.org/target/ENSG00000265681/associations	RPL18<br>RPL19<br>RPL24<br>RPL8<br>RPL11<br>RPL32<br>RPL23<br>RPS6<br>RPL23A<br>RPS4X<br>RPS11<br>RPS13<br>RPS23<br>RPS15A<br>RPS8<br>RPL15<br>RPL26<br>RPS3A<br>RPS20<br>RPS5<br>RPL21<br>RPL5<br>RPL35<br>RPL3<br>RPL4<br>RPL9;<br>RPL9P7;<br>RPL9P8;<br>RPL9P9		No		✓
RPL18	HUMAN 60S ribosomal protein L18	L18	Q07020	ENSG0000(	Cancer,Cancer-Antigen	BC000374	Melanoma, Teratozoospermia, Pancreatic carcinoma, Breast carcinoma, Diabetes mellitus, Colorectal carcinoma, Glioma, Multiple myeloma, Macroglobulinemia, Lung carcinoma	https://www.targetvalidation.org/target/ENSG00000063177/associations	RPL36<br>MAP3K14<br>RPL10L<br>MAP3K1<br>RPL6<br>RPL18A<br>RPL19<br>RPL24<br>RPL38<br>RPL8<br>RPL11<br>RPL32<br>RPL10A<br>RPL31<br>RPL30<br>RPS26<br>RPS25<br>RPL23<br>RPS6<br>RPL23A<br>RPS4X<br>RPL7A<br>RPS11<br>RPS13<br>RPS23<br>RPS14<br>RPS16<br>RPS15A<br>RPS8	Cytoplasm,	No		✓

RPL18A	HUMAN 60S ribosomal protein L18a	L18A	Q02543	ENSG0000(	Cancer,Cancer-Antigen	BC007512	Schizophrenia	https://www.targetvalidation.org/target/ENSG00000105640/associations	RPL36<br>RPL10L<br>MAP3K12<br>TNK2<br>RPL18<br>RPL6<br>RPL19<br>RPL24<br>RPL8<br>RPL11<br>RPL32<br>RPL10A<br>RPL31<br>RPL30<br>RPS26<br>RPL23<br>RPS6<br>RPL23A<br>RPS4X<br>RPL7A<br>RPS11<br>RPS13<br>RPS23<br>RPS14<br>RPS16<br>RPS15A<br>RPS8<br>RPS7<br>RPL37A	No	✓
RPL22	HUMAN 60S ribosomal protein L22	L22	P35268	ENSG0000(	Cancer	CR456873	Melanoma, Cutaneous melanoma, Myelodysplasia, Uterine corpus cancer, Colorectal cancer, Thymic lymphoma, Endometrial carcinoma, Streptococcal pneumonia, Lobar pneumonia, Wiskitt-Aldrich syndrome	https://www.targetvalidation.org/target/ENSG00000116251/associations	MAP3K14<br>RPL18<br>RPL6<br>RPL18A<br>RPL19<br>RPL24<br>RPL11<br>RPL10A<br>RPL31<br>RPL30<br>RPS26<br>RPL23<br>RPS6<br>RPL23A<br>RPS4X<br>RPL7A<br>RPS11<br>RPS13<br>RPS23<br>RPS16<br>RPS8<br>RPL37A<br>RPL15<br>RPS3A<br>RPL14<br>RPL21<br>RPL5<br>RPL27A<br>RPS19	No	✓
RPL27A	HUMAN 60S ribosomal protein L27a	L27A	P46776	ENSG0000(	Cancer,Cancer-Antigen	CH471064	Primary immunodeficiency, Dyskeratosis congenita, Gaucher disease, Griscelli disease, Hoyeraal-Hreidarsson syndrome, Cerebellar ataxia, Pancytopenia, Hyperpigmentation, Fanconi anemia, Isovaleric acidemia	https://www.targetvalidation.org/target/ENSG00000166441/associations	RPL36<br>MAP3K14<br>RPL10L<br>RPL18<br>RPL6<br>RPL19<br>RPL24<br>RPL8<br>RPL11<br>RPL30<br>RPS25<br>RPS6<br>RPL23A<br>RPS4X<br>RPL7A<br>RPS16<br>RPS8<br>RPL37A<br>RPL15<br>RPS3A<br>RPS20<br>RPL14<br>RPL5<br>RPL35<br>RPL3<br>RPL22<br>RPL12<br>RPL10<br>RPL13	No	✓
RPL30	HUMAN 60S ribosomal protein L30	L30	P62888	ENSG0000(	Others	AK312102	Teratozoospermia, Breast carcinoma, Medulloblastoma, Liver carcinoma, Gaucher disease, Lung carcinoma, Metachromatic leukodystrophy, Colonic neoplasm, Hepatocellular carcinoma, Acute lymphoblastic leukemia	https://www.targetvalidation.org/target/ENSG00000156482/associations	RPL36<br>MAP3K14<br>MAP3K1<br>RPL18<br>RPL6<br>RPL18A<br>NFKB2<br>RPL19<br>RPL38<br>RPL8<br>RPL11<br>RPL10A<br>RPL31<br>RPS26<br>RPL23<br>RPS6<br>RPL23A<br>RPS4X<br>RPL7A<br>RPS11<br>RPS13<br>RPS23<br>RPS14<br>RPS16<br>RPS15A<br>RPS8<br>RPS7<br>RPL37A<br>RPL27	No	✓
RPL31	HUMAN 60S ribosomal protein L31	L31	P62899	ENSG0000(	Others	AC016738	Teratozoospermia, Lung carcinoma, Mammary neoplasm, Prostate carcinoma, Blackfan-Diamond anemia, Colorectal adenocarcinoma, Psoriasis, Central nervous system cancer, Multiple myeloma, Melanoma	https://www.targetvalidation.org/target/ENSG00000071082/associations	RPL18<br>RPL6<br>RPL18A<br>RPL19<br>RPL24<br>RPL8<br>RPL11<br>RPL10A<br>RPL30<br>RPS26<br>RPL23<br>RPS6<br>RPL23A<br>RPS4X<br>RPL7A<br>RPS11<br>RPS13<br>RPS23<br>RPS14<br>RPS16<br>RPS15A<br>RPS8<br>RPS7<br>RPL37A<br>RPL27<br>RPL15<br>RPS3A<br>EIF6<br>RPL14	No	✓
RPL32	HUMAN 60S ribosomal protein L32	L32, PP9932	P62910	ENSG0000(	Signalling	AK311909	Periodontitis, Sarcoidosis, Congestive heart failure, Cardiomyopathy, Asthma, Obesity, Osteosarcoma, Inflammatory bowel disease, Interstitial lung disease, Acute lymphoblastic leukemia	https://www.targetvalidation.org/target/ENSG00000144713/associations	MAP3K14<br>RPL18<br>RPL18A<br>RPL19<br>RPL8<br>RPL23<br>RPS6<br>RPL23A<br>RPS4X<br>RPL7A<br>RPS16<br>RPS15A<br>RPS8<br>RPS3A<br>RPL14<br>RPL21<br>RPL5<br>RPL3<br>RPL4<br>RPL9;<br>RPL9P7;<br>RPL9P8;<br>RPL9P9<br>RPS3<br>RPL17<br>RPL7<br>RPS2<br>RPS6<br>RPS3	No	✓
RPL34	HUMAN 60S ribosomal protein L34	L34	P49207	ENSG0000(	Cancer,Cancer-Antigen	CR542242	Non-small cell lung carcinoma, Esophageal squamous cell carcinoma, Retinoblastoma, Synovial sarcoma, Osteosarcoma, Gastric carcinoma, Sepsis, Pancreatic carcinoma, Breast carcinoma, Diabetes mellitus	https://www.targetvalidation.org/target/ENSG00000109475/associations	EIF6	No	✓
RPL37A	HUMAN 60S ribosomal protein L37a	L37A	P61513	ENSG0000(	Cancer,Cancer-Antigen	AK289472	Lung carcinoma, Large cell lung carcinoma, Type I diabetes nephropathy, Polycystic ovary syndrome, Breast carcinoma, Chronic kidney disease, Teratozoospermia, Rheumatoid arthritis, Pancreatic adenocarcinoma, Glioblastoma multiforme	https://www.targetvalidation.org/target/ENSG00000197756/associations	MAP3K1<br>RPL18<br>RPL6<br>RPL18A<br>RPL19<br>RPL24<br>RPL8<br>RPL11<br>RPL10A<br>RPL31<br>RPL30<br>RPS26<br>RPS25<br>RPL23<br>RPS6<br>RPL23A<br>RPS4X<br>RPL7A<br>RPS11<br>RPS13<br>RPS23<br>RPS14<br>RPS16<br>RPS15A<br>RPS8<br>RPS7<br>RPL15<br>RPS3A<br>RPS20	No	✓

RPL5	HUMAN 60S ribosomal protein L5	L5, MSTP030	P46777	ENSG0000(	Cancer	AL162740	Blackfan-Diamond anemia, Melanoma, Acute lymphoblastic leukemia, Aase Smith syndrome 2, Cleft palate, Precursor T-Cell lymphoblastic leukemia-lymphoma, Uranostaphyloschisis, Intermittent migraine headache, Electrocardiogram abnormal, Melanoma	https://www.targetvalidation.org/target/ENSG00000122406/associations	RPL36<br>UPF2<br>MAP3K14<br>RPL10L<br>RPL18<br>RPL6<br>RPL18A<br>RPL19<br>RPL24<br>CSNK2A1<br>RPL38<br>RPL8<br>RPL11<br>RPL32<br>RPL10A<br>RPL31<br>RPL30<br>RPS26<br>RPS25<br>RPL23<br>RPS6<br>RPL23A<br>RPS4X<br>RPL7A<br>RPS11<br>RPS13<br>RPS23<br>RPS14<br>RPS16	Cytoplasm	No		✓
RPL7A	HUMAN 60S ribosomal protein L7a	L7A, SURF3	P62424	ENSG0000(	Others	M36072	Osteosarcoma, Mammary neoplasm, Breast neoplasm, Colorectal carcinoma	https://www.targetvalidation.org/target/ENSG00000148303/associations	RPL36<br>MAP3K14<br>RPL10L<br>RPL18<br>RPL6<br>RPL18A<br>RPL19<br>RPL24<br>RPL38<br>RPL8<br>RPL11<br>RPL32<br>RPL10A<br>RPL31<br>RPL30<br>RPS26<br>RPS25<br>RPL23<br>RPS6<br>RPL23A<br>RPS4X<br>RPS11<br>RPS13<br>RPS23<br>RPS14<br>RPS16<br>RPS15A<br>RPS8<br>RPL37A		No		✓
RPLP1	HUMAN 60S acidic ribosomal protein P1	RRP1	P05386	ENSG0000(	Cancer,Cancer-Antigen	CH471082	Chronic lymphocytic leukemia, Rheumatoid arthritis, Atrial fibrillation, Colon tumor, Cholangiocarcinoma, Systemic lupus erythematosus, Hepatocellular carcinoma, Melanoma, Embryonal neoplasm, Non-small cell lung adenocarcinoma	https://www.targetvalidation.org/target/ENSG00000137818/associations	RPL6<br>RPL19<br>RPL11<br>RPS26<br>RPS25<br>RPL23<br>RPS6<br>RPS4X<br>RPL7A<br>RPS13<br>RPS23<br>RPS16<br>RPS15A<br>RPS8<br>RPS7<br>RPS3A<br>RPS10<br>RPS5<br>RPS19<br>RPL4<br>RPL12<br>RPS3<br>RPS2<br>RPSA<br>RPLP0<br>RPLP2<br>TRIM24<br>EEF1A1<br>RPS6		No		✓
RPS15A	HUMAN 40S ribosomal protein S15a	S15A, OK/SW-cl.82	P62244	ENSG0000(	Others	AB062400	Lung carcinoma, Glioma, Glioblastoma multiforme, Colorectal carcinoma, Hepatitis B, Uveal melanoma, Anaplastic oligodendroglioma, Cystic fibrosis, Prostate adenocarcinoma, Sepsis	https://www.targetvalidation.org/target/ENSG00000134419/associations	MAP3K14<br>RPL18<br>RPL6<br>RPL18A<br>RPL19<br>RPL24<br>RPS21<br>RPL38<br>RPL11<br>RPL32<br>RPL10A<br>RPL31<br>RPL30<br>FAU<br>RPS26<br>RPS25<br>RPS24<br>RPS15<br>RPL23<br>RPS6<br>RPL23A<br>RPS4X<br>RPL7A<br>RPS11<br>RPS13<br>RPS18<br>RPS23<br>RPS14<br>RPS16		No		✓
RPS2	HUMAN 40S ribosomal protein S2	LLREP3, RPS4	P15880	ENSG0000(	Signalling, Cancer	CH471112	Prostate carcinoma, Breast carcinoma, Benign prostatic hyperplasia, Hypoxia, Hodgkin's disease, Turner syndrome, 45-X Gonadal dysgenesis, Colorectal cancer, Hypertrophy, Li-Fraumeni syndrome	https://www.targetvalidation.org/target/ENSG00000140988/associations	RPL10L<br>RPL18<br>RPL6<br>RPL18A<br>RPL19<br>RPL24<br>RPS21<br>RPL38<br>RPS27A<br>RPL8<br>RPL11<br>RPL32<br>RPL10A<br>RPL31<br>RPL30<br>RPS28<br>RPS26<br>RPS25<br>RPS24<br>RPS15<br>RPL23<br>RPS6<br>RPL23A<br>RPS4X<br>RPL7A<br>RPS11<br>RPS13<br>RPS29<br>RPS18		No		✓
RPS27A	HUMAN Ubiquitin-40S ribosomal protein S27a	S27A, Uba80, UBCEP80	P62979	ENSG0000(	Others	BC001392	T-cell leukemia, Listeriosis, Cystic fibrosis, HIV infection, Parkinson's disease, Sandhoff disease, Seminoma, Rous sarcoma, Sudden infant death syndrome, Melanoma	https://www.targetvalidation.org/target/ENSG00000143947/associations	RIPK3<br>MAP3K3<br>MAST2<br>MAP3K1<br>ACVR1<br>TGFBR1<br>RPS2<br>RPSA<br>ISG15<br>PLK4<br>BMPR1B<br>RPSA<br>RPS2	Ubiquitin, Cytoplasm	No		✓
RPS6KA1	HUMAN Ribosomal protein S6 kinase alpha-1	p90Rsk, RSK, RSK1, MAPKAPK1A	Q15418	ENSG0000(	Kinase,Cancer	AK315730	Ovarian carcinoma, Lung carcinoma, Osteosarcoma, Sezary syndrome, Rectal neoplasm, Breast carcinoma, Prostate carcinoma, Colonic neoplasm, Lewy body dementia, Melanoma	https://www.targetvalidation.org/target/ENSG00000117676/associations	RPS6KA6<br>NLK<br>SGK494<br>MAPK15<br>MAPK6<br>MAPK14<br>RPS6KA2<br>PDK1<br>MAPK7<br>PPP2CA<br>PPP2CB<br>MAPK10<br>RPS6KA3<br>GSK3B<br>MAPK9<br>MAPK8<br>MAP3K8<br>MAPK4<br>MAPK1<br>MAPK3<br>CREB1<br>TP53<br>RPS6KA4<br>RPS6KA5<br>AKAP8<br>PDPK1<br>FGFR1<br>FLNA<br>PACS1	Nucleus, Cytoplasm,	Yes	https://www.bindingdb.org/uniprot/Q15418	✓
RPS6KA2	HUMAN Ribosomal protein S6 kinase alpha-2	RSK, RSK3, MAPKAPK1C	Q15349	ENSG0000(	Kinase	AL022069, Z98049	Tongue squamous cell carcinoma, Dental caries, Sclerosis, Colonic neoplasm, Rectal neoplasm, Breast carcinoma, Colon carcinoma, Prostate carcinoma, Inflammatory bowel disease, Ulcerative colitis, Malignant mesothelioma	https://www.targetvalidation.org/target/ENSG00000071242/associations	PIM2<br>WNK1<br>MAP3K14<br>TESK2<br>HIPK4<br>HIPK1<br>PRKG1<br>PRPF4B<br>PRKCZ<br>ACVR1<br>MAP2K1<br>CDK5<br>SRPK2<br>PRKAA2<br>PLK1<br>NEK2<br>GSK3B<br>SYK<br>MTOR<br>TGFBR2<br>MAP2K2<br>MAPK1<br>MAPK3<br>CDKL5<br>PDPK1<br>CTTN<br>FLNA<br>CDKN1B<br>TRIP10	Nucleus	Yes	https://www.bindingdb.org/uniprot/Q15349	✓

RPS6KA3	HUMAN Ribosomal protein S6 kinase alpha-3	MRX19, RSK, RSK2, ISPK1, MAPKAPK 1B	P51812	ENSG0000(	Kinase,Ca ncer	AL732366, AL807772	Coffin-Lowry syndrome, X-linked non-syndromic intellectual disability, Osteogenesis imperfecta, Male infertility with teratozoospermia due to single gene mutation, Liver carcinoma, Seizures, X-linked 19 mental retardation, Pectus excavatum, Orofaciodigital syndrome type I, Bruck syndrome	https://www.targetvalidation.org/ target/ENSG00000177189/asso ciations	PEA15 PPP2CA PPP2CB GSK3B MAPKAPK 2 FLT4 MAPK1 MAPK3 NR4A1 FGFR3 FGFR4 FGFR2 SRC FGFR1 FYN EGFR PDPK1 PPM1D GRIN1 FGFR1 EIF2S1 FLNA CDKN1B HMGN2 TINF2 GSK3A MAPK1 YBX1 ATF4 FGF2	Nucleus	Yes	https://www.bindingd b.org/uniprot/P51812	✓
RPS6KA4	HUMAN Ribosomal protein S6 kinase alpha-4	MSK2, RSKB, KS6A4	O75676	ENSG0000(	Kinase	CH471076	Inflammatory bowel disease, Biliary liver cirrhosis, Crohn's disease, Ulcerative colitis, Leprosy, Rheumatoid arthritis, Atopic eczema, Diffuse large B-cell lymphoma, Breast carcinoma	https://www.targetvalidation.org/ target/ENSG00000162302/asso ciations	RNF2 MAPK14 MAP2K1 CSNK2A1 GSK3B MAPK1 MAPK3 FLNA CDKN1B MAPK1 MAP2K1 RPS6KA1 MAPK14 RELA GSK3B RPS6 MTA1 TSC2 FOS ENO1 CREB1 SRF HMGN1 STK11 SUFU MDM2 DAPK1 PPP1R13L RPTOR BAD ATF1	Nucleus	Yes	https://www.bindingd b.org/uniprot/O75676	✓
RPS6KA5	HUMAN Ribosomal protein S6 kinase alpha-5	MSK1, RLPK	O75582	ENSG0000(	Kinase	AK295266	Colorectal adenocarcinoma, Malignant mesothelioma, Breast carcinoma, Melanoma, Psoriasis, Rheumatoid arthritis, Spinocerebellar Ataxia Type 1, Colon carcinoma, Bladder neoplasm, Atopic eczema	https://www.targetvalidation.org/ target/ENSG00000100784/asso ciations	MAPK14 MAPK11 CSNK2A1 GSK3B MAPK1 MAPK3 PRKACA ATF2 PRKACA FLNA CDKN1B PRKCD MAPK1 EP300 HSPB2 STAT1 HIST3H3 RPS6KA1 H2AFZ NFKB1 RELA NR3C1 GSK3B RPS6 EIF4EBP1 TSC2 PPP2R5D FOS TBC1D4	Nucleus, Cytoplasm,	Yes	https://www.bindingd b.org/uniprot/O75582	✓
RPS6KA6	HUMAN Ribosomal protein S6 kinase alpha-6	RSK4	Q9UK32	ENSG0000(	Kinase	AL389887, AL354653	Li-Fraumeni syndrome, Breast carcinoma, Malignant mesothelioma, Acquired Immunodeficiency Syndrome (AIDS), HIV infection, Renal cell carcinoma, Autism spectrum disorder, Renal carcinoma, Colon carcinoma, Endometrial carcinoma	https://www.targetvalidation.org/ target/ENSG00000072133/asso ciations	MAPK14 GSK3B MAPK3 FLNA CDKN1B RPS6KA1 GSK3B RPS6 TSC2 FOS CREB1 SRF STK11 DAPK1 RPTOR BAD ESR1 NR4A1 SLC9A1 NFATC4 GMFB	Cytoplasm, cytosol	Yes	https://www.bindingd b.org/uniprot/Q9UK3 2	✓
RPS6KB1	HUMAN Ribosomal protein S6 kinase beta -1	p70(S6K)-alpha, S6K1, STK14A	P23443	ENSG0000(	Kinase	AK300202	Multiple sclerosis, Familial hyperinsulinism, Congenital isolated hyperinsulinism, Left ventricular hypertrophy, Atrophic, Tuberous sclerosis, Alzheimer's disease, Ovarian neoplasm, MODY, DEND syndrome	https://www.targetvalidation.org/ target/ENSG00000108443/asso ciations	NEK6 RPTOR PHLPP2 PDK1 PIN1 PPP2R5C PRKCZ CREM PPP2CA PPP2R2A RAC1 RBX1 PPP2CB CDC42 MTOR PIK3CA IRS1 AKT1 MAPK1 MAPK3 EIF4B PRKACA YES1 CDK1 APP PHLPP1 PDPK1 CTTN PRKACA IRF3 MAPK1 TP63 APP	Cell junction, synapse, synaptoso me Mitochondr ion outer membrane, Mitochondr ion,	Yes	https://www.bindingd b.org/uniprot/P23443	✓
RPS6KB2	HUMAN Ribosomal protein S6 kinase beta -2	P70-BETA, STK14B	Q9UBS0	ENSG0000(	Kinase	AF099739	Breast carcinoma, Adenocarcinoma, Lung carcinoma, Rectal neoplasm, Head and Neck neoplasm, Non-small cell lung carcinoma, Rectum cancer, Psoriasis, Tuberous sclerosis, Prostate carcinoma	https://www.targetvalidation.org/ target/ENSG00000175634/asso ciations	IKBKG RPTOR PDK1 PRKCD PRKCZ PRKCE MTOR PRKCI PRKCA SRC PRKCB PRKCG PDPK1 HMGN2 RICTOR JUN KRT18 RPS6 PDPK1 RPTOR MTOR HSPB1 PDCD4 MXD1 EEF2K CRYBA1 COASY	Cytoplasm, Nucleus,	Yes	https://www.bindingd b.org/uniprot/Q9UBS 0	✓
RPS6KL1	HUMAN Ribosomal protein S6 kinase-like 1	RPS6KL1	Q9Y6S9	ENSG0000(	Kinase	AL390152	Li-Fraumeni syndrome, Squamous cell carcinoma, Breast carcinoma, Ovarian carcinoma	https://www.targetvalidation.org/ target/ENSG00000198208/asso ciations			No		✓
RPS7	HUMAN 40S ribosomal protein S7	S7	P62081	ENSG0000(	Signalling	AC108488	Blackfan-Diamond anemia, Waardenburg syndrome, Autosomal recessive spondylocostal dysostosis, Electrocardiogram abnormal, Intermittent migraine headache, Robinow syndrome, Brachydactyly type B, Colorectal cancer, Lateral meningocele syndrome, Aicardi syndrome	https://www.targetvalidation.org/ target/ENSG00000171863/asso ciations	RPL10L RPL18 RPL6 RPL18A RPL19 RPL11 RPL31 RPL30 RPS26 RPS25 RPS24 RPS15 RPL23 RPS6 RPL23A RPS4X RPS11 RPS13 RPS18 RPS23 RPS14 RPS16 RPS15A RPS8 RPL37A RPL15 RPS3A RPS20 RPL14	Cytoplasm, cytoskeleto n, microtubul e organizing center, centrosom e	No		✓
RRAGC	HUMAN Ras-related GTP-binding protein C	FLJ13311, GTR2	Q9HB90	ENSG0000(	Others	BC016668	Neoplasm of mature B-cells, Dilated cardiomyopathy, Lymphoma, Follicular lymphoma, Tuberous sclerosis, Sporadiac amyotrophic lateral sclerosis, Sepsis, Heart failure	https://www.targetvalidation.org/ target/ENSG00000116954/asso ciations	RPTOR RRAGA RRAGB PRPF4B RPTOR MTOR	Cytoplasm	No		✓

RRAS	HUMAN Ras-related protein R-Ras	RRAS	P10301	ENSG0000(	Signalling	CR541944	Noonan syndrome, Autosomal dominant severe congenital neutropenia, Breast carcinoma, Supravalvular aortic stenosis, Combined immunodeficiency with skin granulomas, Juvenile myelomonocytic leukemia, Amyloidosis, Alzheimer's disease, Gemistocytic astrocytoma, Tuberculosis	https://www.targetvalidation.org/target/ENSG00000126458/associations	SOS1 PPP2CA NOTCH1 EPHB2 PLCG1 SRC ARAF FYN RGL2 RAF1 BCL2 RALGDS PIK3R1 NCK1 ARAF RASSF5 AFDN	Cell membrane	No		✓
RRAS2	HUMAN Ras-related protein R-Ras2	TC21	P62070	ENSG0000(	Signalling	AK300103	Ovarian neoplasm, Ovarian carcinoma, Juvenile myelomonocytic leukemia, Parkinson's disease, Breast carcinoma, Leiomyosarcoma, Stickler syndrome, MRCS syndrome, Progressive cone dystrophy, Hypobetalipoproteinemia	https://www.targetvalidation.org/target/ENSG00000133818/associations	RASGRF1 RALGDS ACVR1 ARAF RAF1 RAPGEF3 RGL2 BMPR1B RAF1 RIN1 RALGDS PIK3R1 ARAF RASSF5 AFDN RGL2	Cell membrane	No		✓
RTFDC1	HUMAN Protein RTF2 homolog	C20orf43, CDAO5, HSPC164, RTF2, AD007, HSPC169	Q9BY42	ENSG0000(	Cancer,Cancer-Antigen	AF212244	Pancreatic carcinoma, Acute myeloid leukemia, Melanoma, Gastric carcinoma, Breast carcinoma	https://www.targetvalidation.org/target/ENSG00000022277/associations			No		✓
RUFY1	HUMAN RUN and FYVE domain-containing protein 1	RABIP4, ZFYVE12	Q96T51	ENSG0000(	Kinase	BC032571	Mental or behavioural disorder, Schizophrenia, Ovarian carcinoma, Pancreatic carcinoma, Astrocytoma, Gastric carcinoma, Oancreatic carcinoma, Epilepsy	https://www.targetvalidation.org/target/ENSG00000176783/associations	BMX EPHA3 RAB4A PIAS2	Cytoplasm, Early endosome membrane, Peripheral membrane protein,	No		✓
RUNX1T1	HUMAN Protein CBFA2T1	AML1T1, CBFA2T1, CDR, ETO, MTG8, ZMYND2	Q06455	ENSG0000(	Cancer	AF018282	Lymphoid neoplasm, Lung carcinoma, Esophageal carcinoma, Acute myeloid leukemia, Alzheimer's disease, Prostate carcinoma, Bladder carcinoma, Melanoma, Breast carcinoma, Gastric adenocarcinoma	https://www.targetvalidation.org/target/ENSG00000079102/associations	PAK1 ZBTB16 ATN1 BCL6 AKT1 PRKACA PRKAR2A SP1 HSP90AA1 NCOR1 SIN3A HDAC1 RUNX1 TCF12	Nucleus	No		✓
RUVBL1	HUMAN RuvB-like 1	ECP54, INO80H, NMP238, Pontin52, RVB1, TIP49, TIP49a	Q9Y265	ENSG0000(	Signalling	AF099084	Colonic neoplasm, Prostate neoplasm, Colon carcinoma, Aplastic anemia, Breast carcinoma, Cervix carcinoma, Medulloblastoma, Glioblastoma multiforme, Lung carcinoma, Dermatomyositis	https://www.targetvalidation.org/target/ENSG00000175792/associations	RIPK3 TBK1 MORF4L1 ING3 MRGBP MAP3K3 MAP3K14 TESK2 IKBKE PLK1 MAP3K8 TGFBR2 BMPR1A CTNNB1 TBP H2AFZ ACTL6A STK16 OFD1 MAP3K7 ACTB EP400 RUVBL2 CAD TRRAP TERT TBP ACTG1 VPS72 PLG DKC1	Nucleus matrix, Nucleus, nucleoplasm, Cytoplasm, Membrane, cytoskeleton, microtubule organizing center, centrosome,	Yes	https://www.bindingdb.org/uniprot/Q9Y265	✓
RXRB	HUMAN Retinoic acid receptor RXR-beta	H-2RIIBP, NR2B2, RCoR-1	P28702	ENSG0000(	Transcription factor,Cancer	AF120161	Psoriasis, Psoriasis vulgaris, Non-Hodgkin's lymphoma, Kallmann syndrome, Atopic eczema, Schizophrenia, Thyroid carcinoma, Primary ciliary dyskinesia, Breast carcinoma, Deafness-infertility syndrome	https://www.targetvalidation.org/target/ENSG00000204231/associations	NR0B2 NR1H3 PPARA PPARD NR1H2 NRIP1 PPARG NR2F2 RARG VDR THRB THRA RARB RARA TACC2 PPARA NRIP1 PPARG VDR RARA RARB THRB RARG PPARD THRA NR1H3 NR2F2 NR1H2 NR4A2 NR2F1	Nucleus	Yes	https://www.bindingdb.org/uniprot/P28702	✓
S100A6	HUMAN Protein S100-A6	2A9, CACY	P06703	ENSG0000(	Signalling, Cancer	AY034480	Epithelial neoplasm, Medulloblastoma, Type II diabetes mellitus, Intracranial vasospasm, Osteosarcoma, Adenocarcinoma, Stricture, Lung adenocarcinoma, Duchenne muscular dystrophy, Myocardial ischemia	https://www.targetvalidation.org/target/ENSG00000197956/associations	TP53 CACYBP ANXA11	Nucleus envelope, Cytoplasm, Cell membrane, Peripheral membrane protein, Cytoplasmic side,	No		✓
S100A7	HUMAN Protein S100-A7	PSOR1, S100A7c	P31151	ENSG0000(	Cancer	BC034687	Psoriasis, Head and Neck neoplasia, Atopic dermatitis, Rhinitis, Systemic scleroderma, Breast carcinoma, Tongue squamous cell carcinoma, Inflammatory skin disease, Prostate carcinoma, Tuberous sclerosis	https://www.targetvalidation.org/target/ENSG00000143556/associations	MAP3K3 TGM2 EGFR COPS5 RANBP9 FABP5	Cytoplasm	Yes	https://www.drugbank.ca/biodb/polypeptides/P31151	✓

S100A8	HUMAN Protein S100-A8	CAGA, CFAG, MRP8, P8	P05109	ENSG0000(	Cancer	AK291328	Prostate carcinoma, Squamous cell carcinoma, Atopic dermatitis, Acute myelocytic leukemia, Keloid, Gastric carcinoma, Peptic ulcer, Juvenile periodontitis, Asthma, Psoriasis	https://www.targetvalidation.org/target/ENSG00000143546/associations	MAP3K3 RAC2 RAC1 S100A9	Secreted, Cytoplasm, cytoskeleton, Cell membrane, Peripheral membrane protein,	No		✓
S100A9	HUMAN Protein S100-A9	CAGB, CFAG, MRP14, P14	P06702	ENSG0000(	Cancer	AF237581	Psoriasis, Colonic neoplasm, Schizophrenia, Keloid, Squamous cell carcinoma, Vasculitis, Rheumatoid arthritis, Alzheimer's disease, Chronic granulomatous disease, Tuberculosis	https://www.targetvalidation.org/target/ENSG00000163220/associations	MAPK14 WEE1 S100A8 EGFR RAC2 RAC1	Secreted, Cytoplasm, cytoskeleton, Cell membrane, Peripheral membrane protein,	No		✓
S100P	HUMAN Protein S100-P	S100P	P25815	ENSG0000(	Others	AY423724	Breast carcinoma, Tongue squamous cell carcinoma, Polycystic ovary syndrome, Adenocarcinoma, Ovarian neoplasm, Colon carcinoma, Ulcerative colitis, Leukemia, Lung carcinoma	https://www.targetvalidation.org/target/ENSG00000163993/associations	EZR CACYBP AGER MOK S100Z	Nucleus, Cytoplasm, Cell projection, microvillus membrane,	Yes	https://www.drugbank.ca/biodb/polypeptides/P25815	✓
SAGE1	HUMAN Sarcoma antigen 1	CT14, SAGE	Q9NXZ1	ENSG0000(	Others	NA	Breast carcinoma, Ovarian carcinoma, Lung neoplasm, Medulloblastoma, Noninfiltrating intraductal carcinoma, Melanoma, Spermatocytic seminoma, Li-Fraumeni syndrome, Azoospermia, Germ cell tumor	https://www.targetvalidation.org/target/ENSG00000181433/associations			No		✓
SAV1	HUMAN Protein salvador homolog 1	salvador, WW45, WWP4	Q9H4B6	ENSG0000(	Kinase,Cancer	AK290883	Glioblastoma multiforme, Syndromic diarrhea, Generalized pustular psoriasis, Liver carcinoma, Pancreatic carcinoma, Adenomatous polyposis coli, Congestive heart failure, Malignant mesothelioma, Werner syndrome, White sponge nevus	https://www.targetvalidation.org/target/ENSG00000151748/associations	STK3 STK4 STK4 STK3 RASSF1	Nucleus	No		✓
SBDS	HUMAN Ribosome maturation protein SBDS	CGI-97, FLJ10917, SDS, SWDS	Q9Y3A5	ENSG0000(	Transcription factor,Cancer	AK289609	Shwachman-Diamond syndrome, Myelodysplastic syndrome, Exocrine pancreatic insufficiency, Osteopenia, Respiratory Distress Syndrome, Myocardial necrosis, Hepatocellular carcinoma, Gastric adenocarcinoma, Melanoma, Bladder carcinoma	https://www.targetvalidation.org/target/ENSG00000126524/associations	EFL1	Cytoplasm, Nucleus, nucleolus, nucleoplasm, cytoskeleton, spindle,	No		✓
SCAND1	HUMAN SCAN domain-containing protein 1	SDP1	P57086	ENSG0000(	Transcription factor	CH471077	Ovarian carcinoma, Psoriasis, Malignant peripheral nerve sheath tumor, Melanoma, Atopic eczema, Prostate carcinoma, Gastric carcinoma, Li-Fraumeni syndrome, Breast carcinoma	https://www.targetvalidation.org/target/ENSG00000171222/associations	MZF1 ZNF202 ZSCAN21	Nucleus	No		✓
SCEL	HUMAN Sciellin	SCEL	O95171	ENSG0000(	Others	AL137140	Lung carcinoma, Squamous cell carcinoma, Lung adenocarcinoma, Chronic kidney failure, Papillary thyroid carcinoma, Prostate carcinoma, Cystic fibrosis, Atopic eczema, Ovarian carcinoma, Gastric carcinoma	https://www.targetvalidation.org/target/ENSG00000136155/associations	TXNIP	Cytoplasm, Membrane,	No		✓
SCFD1	HUMAN Sec1 family domain-containing protein 1	C14orf163, KIAA0917, RA410, SLY1, STXBP1L2	Q8WVM8	ENSG0000(	Cancer,Cancer-Antigen	AF319958	Breast carcinoma, Chronic gastritis, Glioblastoma multiforme, Gastric carcinoma, Amyotrophic lateral sclerosis	https://www.targetvalidation.org/target/ENSG00000092108/associations	STX5	Cytoplasm	No		✓
SCGB1D2	HUMAN Secretoglobin family 1D member 2	LIPHB, LPNB	O95969	ENSG0000(	Cancer	BC104840	Rosacea, Breast carcinoma, Psoriasis, Pituitary neoplasm, Fibroadenoma, Lung carcinoma, Prostate carcinoma, Atopic eczema, Melanoma, Hepatocellular carcinoma	https://www.targetvalidation.org/target/ENSG00000124935/associations	SCGB2A2	Secreted	No		✓

SCMH1	HUMAN Polycomb protein SCMH1	Scml3	Q96GD3	ENSG00000	Transcripti on factor	CR457161	Male infertility with azoospermia or oligozoospermia due to single gene mutation, Kallmann syndrome, Isolated Klippel-Feil syndrome, Mayer-Rokitansky-Küster-Hauser syndrome, Frontometaphyseal dysplasia, Waardenburg syndrome type I, Okihiro syndrome, MURCS association, Gorlin syndrome, Ovarian carcinoma	https://www.targetvalidation.org/target/ENSG00000010803/associations	PHC1	Nucleus		Yes		✓
													https://www.drugbank.ca/biodb/polypeptides/Q96GD3	
SCP2	HUMAN Non-specific lipid-transfer protein	NLTP	P22307	ENSG0000(	Others	M55421	Leukoencephalopathy-dystonia-motor neuropathy, Hyperlipoproteinemia type 1, Homozygous familial hypercholesterolemia, Neonatal intrahepatic cholestasis due to citrin deficiency, Action Tremor, Spasmodic torticollis, Head tremor, Impaired saccades, Neuropathy, LCAT deficiency	https://www.targetvalidation.org/target/ENSG00000116171/associations	FOXO3 CAV1	Cytoplasm		Yes		✓
													https://www.bindingdb.org/uniprot/P22307	
SCYL3	HUMAN Protein-associating with the carboxyl-terminal domain of ezrin	PACE-1, PACE1	Q8IZE3	ENSG0000(	Kinase	AK292507	Glioblastoma multiforme, Ovarian carcinoma, Lung carcinoma, Chronic lymphocytic leukemia, Breast carcinoma, Osteoporosis, Endometrial neoplasm, Prostate carcinoma, Glioblastoma multiforme	https://www.targetvalidation.org/target/ENSG00000000457/associations	EZR CBL	Cytoplasm		No		✓
SDCCAG3	HUMAN Serologically defined colon cancer antigen 3	NY-CO-3	Q96C92	ENSG0000(	Cancer	AL592301	Colonic neoplasm, Colorectal adenocarcinoma, Lung carcinoma, Parkinson's disease, Medulloblastoma, Anaplastic large cell lymphoma, Glioblastoma multiforme, Interstitial lung disease	https://www.targetvalidation.org/target/ENSG00000165689/associations		Cytoplasm		No		✓
SDCCAG8	HUMAN Serologically defined colon cancer antigen 8	BBS16, CCCAP, NPHP10, NY-CO-8, SLSN7, SDCG8, hSPC085	Q86SQ7	ENSG0000(	Cancer	AF161348	Ciliopathy, Senior-Loken syndrome, Retinal dystrophy, Bardet-Biedl syndrome, Obesity, Renal cyst, Schizophrenia, Meckel syndrome, Brachydactyly type A5, Syndactyly type 2	https://www.targetvalidation.org/target/ENSG0000054282/associations	TSC1 CEP164 TSC1	Cytoplasm, cytoskeleton, microtubule organizing center, centrosome, centriole, Cell junction,		No		✓
SDHB	HUMAN Succinate dehydrogenase [ubiquinone] iron-sulfur subunit, mitochondrial	SDH, SDH1	P21912	ENSG0000(	Cancer	AL049569	Adrenal gland pheochromocytoma, Hereditary pheochromocytoma-paraganglioma, Carney-Stratakis syndrome, Paraganglioma, Gastrointestinal stromal tumor, Renal cell carcinoma, Multiple hamartoma syndrome, Mitochondrial complex II deficiency, Epithelial neoplasm, Squamous cell lung carcinoma	https://www.targetvalidation.org/target/ENSG00000117118/associations	SDHAF2 SDHA SDHA	Mitochondrion inner membrane, Peripheral membrane protein, Matrix side,		Yes		✓
													https://www.drugbank.ca/biodb/polypeptides/P21912	
SDHC	HUMAN Succinate dehydrogenase cytochrome b560 subunit, mitochondrial	CYB560, PGL3	Q99643	ENSG00000	Kinase,Cancer	AF081495	Carney-Stratakis syndrome, Hereditary pheochromocytoma-paraganglioma, Inherited cancer-predisposing syndrome, Gastrointestinal stromal tumor, Breast carcinoma, Carotid body paraganglioma, Glomus jugulare tumor, Multiple hamartoma syndrome, Extra-adrenal pheochromocytoma, Cowden syndrome	https://www.targetvalidation.org/target/ENSG00000143252/associations	SDHD SDHD	Mitochondrion inner membrane, Multi-pass membrane protein,	Transmembrane	Yes		✓
													https://www.drugbank.ca/biodb/polypeptides/Q99643	
SEC14L2	HUMAN SEC14-like protein 2	C22orf6, KIAA1186, KIAA1658, S14L2	O76054	ENSG0000(	Others	AK303751	Cholesterol-ester transfer protein deficiency, Hyperlipoproteinemia type 5, Breast carcinoma, Type I diabetes mellitus, Ankylosing spondylitis, Hepatitis C inection, Prostate carcinoma, LCAT deficiency, Epithelial neoplasm, Hypobetalipoproteinemia	https://www.targetvalidation.org/target/ENSG00000100003/associations	PRKACA PIK3CG	Cytoplasm, Nucleus,		Yes		✓
													https://www.drugbank.ca/biodb/polypeptides/O76054	

SECISBP2	HUMAN Selenocysteine insertion sequence-binding protein 2	SEBP2, SBP2	Q96T21	ENSG0000(	Cancer,Cancer-Antigen	BX000356, AL160054, AL929575	Short stature-delayed bone age due to thyroid hormone metabolism deficiency, Vitamin B12-responsive methylmalonic acidemia, Methylmalonic acidemia with homocystinuria, Homocystinuria without methylmalonic aciduria, Trichomegaly - retina pigmentary degeneration - dwarfism, Colorectal cancer, Inborn errors amino acid metabolism, Myopathy, Liver fibrosis, Cohen syndrome	https://www.targetvalidation.org/target/ENSG00000187742/associations		Nucleus	No	✓
SEMG1	HUMAN Semenogelin-1	CT103, SEMG	P04279	ENSG0000(	Others	AY256465	Amyloidosis, AL amyloidosis, Teratozoospermia, Colorectal neoplasm, Ovarian carcinoma, Multiple myeloma, Non-small cell lung carcinoma, Epithelial ovarian cancer, Sarcoma, Neoplasm of mature B-cells	https://www.targetvalidation.org/target/ENSG00000124233/associations	PRKCA KLK3	Secreted	No	✓
SEPTIN4	Septin-4		O43236	ENSG00000108387-SEI		AF176379	refractive error measurement, glomerular filtration rate, body height, Non-syndromic male infertility due to sperm motility disorder, azoospermia, Male infertility with azoospermia or oligozoospermia due to single gene mutation, Male infertility with teratozoospermia due to single gene mutation, spermatogenic failure 43, spermatogenic failure 42, spermatogenic failure 44, Autosomal recessive non-syndromic sensorineural deafness type DFNB, spermatogenic failure 31, Partial chromosome Y deletion, Male infertility due to globozoospermia, Male infertility due to large-headed multiflagellar polyploid spermatozoa, spermatogenic failure 1, Congenital bilateral absence of vas deferens, Isolated follicle stimulating hormone deficiency, Female infertility due to zona pellucida defect, Isochromosomy Yp	https://platform.opentargets.org/target/ENSG00000108387/associations		Cell projection, Cilium, Cytoplasm, Cytoplasmic vesicle, Cytoskeleton, Flagellum, Mitochondrion, Nucleus		✓
SEPTIN5	Septin-5	Cell division control-related protein 1, CDCrel-1, Peanut-like protein 1	Q99719	ENSG00000184702-SEI		CR456545	heel bone mineral density, bone quantitative ultrasound measurement, platelet count, bone density, mean platelet volume, platelet component distribution width, acute myeloid leukemia, esophageal squamous cell carcinoma, prostate adenocarcinoma, hepatocellular carcinoma, brain glioblastoma, small cell lung carcinoma, papillary renal cell carcinoma, breast ductal adenocarcinoma, thyroid carcinoma, metaplastic breast carcinoma, squamous cell lung carcinoma, cecum adenocarcinoma, gastric intestinal type adenocarcinoma, Ovarian Endometrioid Adenocarcinoma with Squamous Differentiation	https://platform.opentargets.org/target/ENSG00000184702/associations		Cytoplasm, Cytoskeleton		✓
SEPTIN6	Septin-6	KIAA0128, SEP2, SEPT6	Q14141	ENSG00000125354-SEI		AF403061	acute myeloid leukemia, pancreatic ductal adenocarcinoma, hepatocellular carcinoma, pancreatic carcinoma, cutaneous melanoma, carcinoma of liver and intrahepatic biliary tract, cervical squamous cell carcinoma, colon adenocarcinoma, bile duct adenocarcinoma, brain glioblastoma, basal cell carcinoma, clear cell renal carcinoma, glioma, breast carcinoma, Uterine Carcinosarcoma, oral cavity carcinoma, skin squamous cell carcinoma, gastric carcinoma, bladder carcinoma, lymphoid neoplasm	https://platform.opentargets.org/target/ENSG00000125354/associations		Cell projection, Centromere, Chromosome, Cilium, Cytoplasm, Cytoskeleton, Flagellum, Kinetochore		✓
SEPTIN9	Septin-9	MLL septin-like fusion protein, Ov/Br septin, Septin D1	Q9UHD8	ENSG00000184640-SEI		AJ312321	neuralgic amyotrophy, refractive error measurement, heel bone mineral density, reticulocyte count systolic blood pressure, reticulocyte measurement, intraocular pressure measurement, neutrophil count, body height, platelet component distribution width, lymphocyte percentage of leukocytes, acute myeloid leukemia, bone quantitative ultrasound measurement, drug use measurement, blood pressure, hypertension, cardiovascular disease, Diuretic use measurement, type II diabetes mellitus, Immature Reticulocyte Fraction Measurement	https://platform.opentargets.org/target/ENSG00000184640/associations		Cytoplasm, Cytoskeleton		✓
SERPINB5	HUMAN Serpin B5	maspin, PI5, SPB5	P36952	ENSG0000(	Signalling	BC020713	Epithelial neoplasm, Lung carcinoma, Mammary neoplasm, Keloid, Colorectal neoplasm, Adenocarcinoma, Lung neoplasm, Sarcoma, Gastric cardia carcinoma, SHORT syndrome	https://www.targetvalidation.org/target/ENSG00000206075/associations	HDAC1 ISG15 TP53 IRF6 PRSS21	Secreted, extracellular space	No	✓
SERPINE2	HUMAN Glia-derived nexin	GDN, PI7, PN1	P07093	ENSG0000(	Others	AC073641	Breast carcinoma, Chronic obstructive pulmonary disease, Cerebral infarction, Pulmonary emphysema, Hypercapnia, Hepatopulmonary syndrome, Pulmonary hypertension, Anoxia, Alpha 1-Antitrypsin deficiency, Hepatocellular carcinoma	https://www.targetvalidation.org/target/ENSG00000135919/associations	PLAU F2	Secreted, extracellular space	No	✓

SERPINF2	HUMAN Alpha-2-antiplasmin	A2AP, AAP, ALPHA-2-PI, PLI	P08697	ENSG0000(	Others	AC130343	Rare hemorrhagic disorder due to a constitutional coagulation factors defect, Congenital alpha2 antiplasmin deficiency, Colorectal cancer, Autosomal recessive predisposition, Hemarthrosis, Ecchymosis, Hemothorax, Hemothorax, Blood Coagulation Disorder, Streptococcal Infection	https://www.targetvalidation.org/target/ENSG00000167711/associations	PRSS1 F11 FGA PLG CFD PLG DEFA1; DEFA1B KLK13 SERPINB12 CTRB1	Secreted	Yes	https://www.drugbank.ca/biodb/polypeptides/P08697	✓
SET	HUMAN Protein SET	2PP2A, IPP2A2, PHAPII, IGAAD	Q01105	ENSG0000(	Cancer	U51924	Colorectal adenocarcinoma, Breast ductal adenocarcinoma, Bladder carcinoma, Necrosis, Acute myelocytic leukemia, Blast phase, Prostate carcinoma, Cutaneous melanoma, Angiosarcoma, Lung adenocarcinoma	https://www.targetvalidation.org/target/ENSG00000119335/associations	SETBP1 TREX1 PRKD2 CDK5 HIST1H3A; HIST1H3B; HIST1H3C; HIST1H3D; HIST1H3E; HIST1H3F; HIST1H3G ; HIST1H3H; HIST1H3I; HIST1H3J CSNK2A1 CSNK2B RAC1 PIK3CG ANP32A CDKN1A HMGB2 HNRNPA2 B1 CHRM3 CSNK2A2 NME1 GZMA PTMA APP TP53	Cytoplasm, cytosol, Endoplasmic reticulum, Nucleus, nucleoplasm,	No		✓
SFN	HUMAN 14-3-3 protein sigma	HME1	P31947	ENSG0000(	Cancer	AF029082	Hepatocellular carcinoma, Li-Fraumeni syndrome, Squamous cell carcinoma, Lymphatic metastasis, Lung adenocarcinoma, Liver carcinoma, Keloid, Adenocarcinoma, Baller-Gerold syndrome, EEC syndrome	https://www.targetvalidation.org/target/ENSG00000175793/associations	MAP3K5 TRIM25 MST1R YWHAZ YWHAG MAPK10 MAPK9 MAPK8 AKT1 CDC25B MARK3 YWHAQ ANPEP BRAF PRKAR2A BCR CDK1 TP53 RAF1 EGFR ABL1 MAP3K6 PLK4 YWHAG CDKN1B RAF1 FOXO4 RICTOR YWHAQ YWHAZ	Cytoplasm, Nucleus, Secreted,	Yes	https://www.bindingdb.org/uniprot/P31947	✓
SGK1	HUMAN Serine/threonine-protein kinase Sgk1	SGK	O00141	ENSG0000(	Kinase,Cancer	FM205707	Breast carcinoma, Unipolar depression, Uremia, Diabetic Neuropathy, Type II diabetes mellitus, Alzheimer's disease, Melanoma, Lung carcinoma, Prostate carcinoma, Colonic neoplasm	https://www.targetvalidation.org/target/ENSG00000118515/associations	IKBKG NEK6 RICTOR PDPK2P SLC9A3R2 PDK2 PDK1 MAPK7 PRKX MTOR PRKACA PRKY PDPK1 IKBKB CTNNB1 WNK4 SLC2A4 SLC9A3R2 CDKN1B FOXO1 GSK3A MAPK1 NEDD4 STUB1 KPNA2 FBXW7 RICTOR KCNQ4 BRAF	Cytoplasm, Nucleus, Endoplasmic reticulum membrane, Cell membrane, Mitochondrion,	Yes	https://www.bindingdb.org/uniprot/O00141	✓
SGK2	HUMAN Serine/threonine-protein kinase Sgk2	SGK2	Q9HBY8	ENSG0000(	Kinase	BT020098	Inflammatory bowel disease, Ulcerative colitis, Clear cell renal carcinoma, Cervix carcinoma, Rosacea, Crohn's disease, Atopic eczema, Shwachman-Diamond syndrome, Vulvar intraepithelial neoplasia, Pancreatic ductal adenocarcinoma	https://www.targetvalidation.org/target/ENSG00000101049/associations	PDK1 PDPK1 PRKACA FOXO1 BRAF GSK3B MAP3K3 CREB1 FOXO3 NDRG1 HTT CARHSP1 NEDD4L KCNJ1 NDRG2	Cytoplasm	Yes	https://www.bindingdb.org/uniprot/Q9HBY8	✓
SGK3	HUMAN Serine/threonine-protein kinase Sgk3	SGK2, SGKL	Q96BR1	ENSG0000(	Kinase	AK291428	Marie Unna hereditary hypotrichosis, Pure hair and nail ectodermal dysplasia, Woolly hair, Skin fragility-woolly hair-palmoplantar keratoderma syndrome, Hypotrichosis simplex, Breast carcinoma, Melanoma, Glioblastoma multiforme, Prostate carcinoma, Multiple myeloma	https://www.targetvalidation.org/target/ENSG00000104205/associations	PDK1 PRKCA PDPK1 ITCH SLC9A3R2 FOXO1 GSK3A BRAF GSK3B MAP3K3 CREB1 FOXO3 NDRG1 HTT CARHSP1 NEDD4L KCNJ1 NDRG2 FLII FIP1L1	Cytoplasmic vesicle Early endosome, Recycling endosome,	Yes	https://www.bindingdb.org/uniprot/Q96BR1	✓
SGO1	HUMAN Shugoshin 1	SGOL1	Q5FBB7	ENSG0000(	Others	AB193058	Central nervous system cancer, Cardiac arrhythmia, Chronobiology disorder, Intestinal disease, Colorectal cancer, Liver carcinoma, Bipolar disorder, Psoriasis, Pancreatic adenocarcinoma, Lung carcinoma	https://www.targetvalidation.org/target/ENSG00000129810/associations	PPP2CA NEK2 PPP2R1A CDK1 PPP2R1B PPP2R5D PPP2R5C PPP2CA PPP2CB PPP2R5B PPP2R1A PPP2R5A PPP2R5E	Nucleus	No		✓
SGSM3	HUMAN Small G protein signaling modulator 3	DJ1042K10.2, RabGAP-5, RABGAP5, RUSC3, RUTBC3	Q96HU1	ENSG0000(	Cancer,Cancer-Antigen	CR456441	Hepatocellular carcinoma, Breast carcinoma, Neurofibromatosis type 2, Melanoma, Prostate carcinoma, Thyroid carcinoma, Colon carcinoma, Colorectal carcinoma	https://www.targetvalidation.org/target/ENSG00000100359/associations	RAB5C NF2 GJA1	Cytoplasm	No		✓

SH2B1	HUMAN SH2B adapter protein 1	KIAA1299, SH2B	Q9NRF2	ENSG0000(	Signalling	AB037720	Obesity, Inflammatory bowel disease, Hyperinsulinism, Autism spectrum disorder, Attention deficit hyperactivity disorder, WAGR syndrome, MODY, Type I diabetes mellitus, Androgen insensitivity syndrome, LEOPARD syndrome	https://www.targetvalidation.org/target/ENSG00000178188/associations	NTRK2 FLT3 MAPK1 MAPK3 JAK1 FGFR3 PRKCA PDGFRA KIT PDGFRB CSF1R INSR NTRK1 EGFR JAK2 SH2B2 NTRK1 JAK2 GRB2 STAT3	Cytoplasm	No		✓
SH2D1A	HUMAN SH2 domain-containing protein 1A	DSHP, IMD5, SAP, XLP, XLPD	O60880	ENSG0000(	Others	AB586694	Primary immunodeficiency, Lymphoproliferative syndrome, X-linked lymphoproliferative disease, Lymphoma, Hypogammaglobulinemia, Meningitis, Pancytopenia, Splenomegaly, Thrombocytopenia, Multiple myeloma	https://www.targetvalidation.org/target/ENSG00000183918/associations	CD244 NTRK2 NTRK3 SLAMF1 ERBB3 FYN LCK TP53 NTRK1 EGFR FYN CD247 SLAMF1	Cytoplasm	Yes		✓
												https://www.bindingdb.org/uniprot/O60880	
SH3BGRL3	HUMAN SH3 domain-binding glutamic acid-rich-like protein 3	SH3L3, P1725	Q9H299	ENSG0000(	Others	AL451139	Colorectal cancer, Pancreatic carcinoma, Bladder neoplasm, Transitional cell carcinoma, Urothelial carcinoma, Liver carcinoma, Psoriasis, Melanoma, Rheumatoid arthritis, Medulloblastoma	https://www.targetvalidation.org/target/ENSG00000142669/associations	ERBB2	Cytoplasm, Nucleus,	No		✓
SH3GL1	HUMAN Endophilin-A2	CNSA1, EEN, SH3D2B	Q99961	ENSG0000(	Cancer	AK299166	Listeriosis, Melanoma, Lymphoid neoplasm, Acute myelocytic leukemia, Breast neoplasm, Prostate adenocarcinoma, Hepatocellular carcinoma, Squamous cell lung carcinoma, Gastric adenocarcinoma, Bile duct carcinoma	https://www.targetvalidation.org/target/ENSG00000141985/associations	LRRK2 PTK2 SRC	Cytoplasm	No		✓
SH3GLB1	HUMAN Endophilin-B1	Bif-1, CGI-61, KIAA0491	Q9Y371	ENSG0000(	Cancer,Cancer-Antigen	AB007960	Obesity, Ovarian carcinoma, Adenocarcinoma, Merkel cell carcinoma, Lymphoid leukemia, Lung neoplasm, Mammary neoplasm, Benign prostatic hyperplasia, Cerebral ishemia, Alzheimer's disease	https://www.targetvalidation.org/target/ENSG00000097033/associations	UVRAG CDK5 SRC BAK1 BECN1 PIK3C3 CBL BAX UVRAG	Cytoplasm	No		✓
SHPK	HUMAN Sedoheptulokinase	CARKL, SHK	Q9UHJ6	ENSG0000(	Kinase	AL832420	Gastric carcinoma, Lung carcinoma, Diffuse large B-cell lymphoma, Arthrogryposis, Postprandial hyperglycemia, Abnormal CNS myelination, Subcortical cerebral atrophy, Glioma, Ovarian carcinoma, Cystinosis	https://www.targetvalidation.org/target/ENSG00000197417/associations		Cytoplasm	No		✓
SIAH1	HUMAN E3 ubiquitin-protein ligase SIAH1	Siah-1, Siah-1a	Q8IUQ4	ENSG0000(	Others	EF026094	Kallmann syndrome, Breast carcinoma, Normosmic congenital hypogonadotropic hypogonadism, Liver carcinoma, Parkinson's disease, Adenomatous polyposis coli, Cervical cancer, Colon carcinoma, Astrocytoma, Prostate carcinoma	https://www.targetvalidation.org/target/ENSG00000196470/associations	HIPK3 MYD88 SH3RF1 TERF2 ATR ATM PKD1 UBE2D3 GRM5 EEF1D TP53 GAPDH DAB1 SIAH2 NUMB CTNNB1 HIPK2 NOTCH1 BAG1 RBBP8 SNCAIP SNCA TRIB3 CACYBP AFF4 PHC2	Cytoplasm, Nucleus,	No		✓
SIK2	HUMAN Serine/threonine-protein kinase SIK2	DKFZp434K1115, KIAA0781, LOH11CR1I, QIK, SNF1LK2	Q9H0K1	ENSG0000(	Kinase	EF445030	Griscelli disease, Griscelli disease type 3, Central nervous system cancer, Ovarian carcinoma, Colorectal cancer, Colon carcinoma, Prostate carcinoma, Obesity, Diffuse large B-Cell lymphoma, Glioma	https://www.targetvalidation.org/target/ENSG00000170145/associations	STK11 AKT2 PRKACA IRS1 CEP250 CRTC2 CDK5R1 CRTC1	Cytoplasm	Yes		✓
												https://www.bindingdb.org/uniprot/Q9H0K1	
SIVA1	HUMAN Apoptosis regulatory protein Siva	CD27BP, SIVA, Siva-1, Siva-2	O15304	ENSG0000(	Others	AF401214	Lung carcinoma, Non-small cell lung carcinoma, Coxsackievirus infection, Breast carcinoma, Colon carcinoma, Melanoma, Acute promyelocytic leukemia, Hepatocellular carcinoma, Sjogren's syndrome, Juvenile dermatomyositis	https://www.targetvalidation.org/target/ENSG00000184990/associations	BCL2L1 ABL2 TYK2 TP53 MAP3K7 MEFV TCAP CD27 BCL2L1 FOXP3 CDKN2A	Cytoplasm	No		✓

SIX1	HUMAN Homeobox protein SIX1	DFNA23	Q15475	ENSG0000(	Transcription factor	BC008874	Branchio-otic syndrome, Autosomal dominant non-syndromic sensorineural deafness type DFNA, BOR syndrome, Glaucoma, Open-angle glaucoma, Congenital abnormality, Congenital deafness, Gustatory lacrimation, Lacrimoauriculodentodigital syndrome, Prostate carcinoma	https://www.targetvalidation.org/target/ENSG00000126778/associations	FOXI1 CDH1 EYA2 DLX5 PAX2 GATA3 OTX2 EYA1 DACH1 LFNG HMX3 BMP4 FGF10 DACH2 OTX1 FGF3	Nucleus Cytoplasm,		No		✓
SKAP1	HUMAN Src kinase-associated phosphoprotein 1	SCAP1, SKAP55	Q86WV1	ENSG0000(	Kinase	AC036222	Ovarian carcinoma, Malignant epithelial tumor of ovary, Hairy cell leukemia, Peripheral T-Cell lymphoma, Hodgkin's lymphoma, Lung adenocarcinoma, Hepatocellular carcinoma, Schizophrenia, Arthritis, Neoplasm of mature B-cells	https://www.targetvalidation.org/target/ENSG00000141293/associations	GRB2 SRC PTPRC LYN FYN LCK FYB SRC	Cytoplasm, Nucleus, Cell membrane,		No		✓
SLA	HUMAN Src-like-adapter	SLAP, SLAP1	Q13239	ENSG0000(	Kinase,Signalling	CR536537	Lung carcinoma, Acute myeloid leukemia, Clear cell renal carcinoma, Acute monocytic leukemia, Vitiligo, Duodenal ulcer, Sjogren's syndrome, Septic shock, Glioblastoma multiforme, Pancreatitis	https://www.targetvalidation.org/target/ENSG00000155926/associations	SYK ZAP70 FLT3 EPHA2 CBL VAV1 FGFR1 PDGFRB ERBB2 EGFR LAT ZAP70 SYK CD247	Cytoplasm		No		✓
SLA2	HUMAN Src-like-adapter 2	C20orf156, SLAP-2	Q9H6Q3	ENSG0000(	Kinase	AK025645	Osteosarcoma, Breast carcinoma, Acute lymphoblastic leukemia, Stroke, Non-small cell lung carcinoma, Shwachman-Diamond syndrome, Meningococcal infection, Cerebral ishemia, Cytomegalovirus infection	https://www.targetvalidation.org/target/ENSG00000101082/associations	PKN1 ZAP70 ERBB2 AURKA CBL CD247	Cytoplasm, Cell membrane, Cytoplasmic vesicle,		No		✓
SLC25A6	HUMAN ADP/ATP translocase 3	ANT3	P12236	ENSG0000(	Cancer,Cancer-Antigen	BC008737	Influenza infection, HIV infection, Melanoma, Bipolar disorder, Familial idiopathic cardiomyopathy, Refractory cancer, Complete atrioventricular block, Enterovirus Infection, Turner syndrome, Duchenne muscular dystrophy	https://www.targetvalidation.org/target/ENSG00000169100/associations	MAP3K3 MAP3K1 FGFR3	Mitochondrion inner membrane, Multi-pass membrane protein,	Transmembrane	Yes	https://www.drugbank.ca/biodb/polypeptides/P12236	✓
SLCO6A1	HUMAN Solute carrier organic anion transporter family member 6A1	CT48, SLC21A19, OATP6A1	Q86UG4	ENSG0000(	Others	AC094108	Schizophrenia, Azoospermia, Autoimmune disease, Breast carcinoma, Lung carcinoma, Liver carcinoma, Prostate carcinoma, Colorectal carcinoma, Colorectal cancer, Crohn's disease	https://www.targetvalidation.org/target/ENSG00000205359/associations		Cell membrane, Multi-pass membrane protein,	Transmembrane	No		✓
SMAD2	HUMAN Mothers against decapentaplegic homolog 2	JV18-1, MADH2, MADR2	Q15796	ENSG0000(	Kinase,Transcription factor,Signalling,Cancer	AF027964	Adenocarcinoma, Squamous cell carcinoma, Craniofacial abnormality, Acromicric dysplasia, Chronic hepatitis, Copper-overload cirrhosis, Colorectal cancer, Squamous cell carcinoma, Liver carcinoma, Holoprosencephaly	https://www.targetvalidation.org/target/ENSG00000175387/associations	PIAS3 UCHL5 STRAP CAMK2A TRIM33 MTMR4 SMURF1 SMURF2 RANBP3 HIPK2 WWP1 NUAK2 WWTR1 CTDSP1 ERBIN NEDD4L ITCH IRF7 KAT2B KAT2A CREBBP HDAC2 UHMK1 SNIP1 EID2 RNF111 MAPK14 MAPK11 TGIF1 SNW1	Cytoplasm		Yes	https://www.drugbank.ca/biodb/polypeptides/Q15796	✓
SMAD4	HUMAN Mothers against decapentaplegic homolog 4	DPC4, MADH4	Q13485	ENSG0000(	Kinase,Transcription factor,Cancer	CH471096	Myhre syndrome, Inherited cancer-predisposing syndrome, Juvenile polyposis syndrome, Brachydactyly, Deafness, Colorectal neoplasm, Hereditary hemorrhagic telangiectasia, Pancreatic carcinoma, Hyperplasia, Renal carcinoma	https://www.targetvalidation.org/target/ENSG00000141646/associations	PIAS3 BTRC TRIM33 FBXW11 DAXX NLK EIF2AK4 WWOX SMURF1 NEK6 NUAK2 CITED1 SMAD5 TSSK3 PBK USP9X IRF7 COPS5 CREBBP STK35 UHMK1 SNIP1 EID2 PIAS4 STK11IP MAPKAPK5 STRADA E2F4 STK11 SMAD1	Cytoplasm		No		✓

SMAD5	HUMAN Mothers against decapentaplegic homolog 5	Dwfc, JV5-1, MADH5	Q99717	ENSG00000	Kinase,Transcription factor,Signalling	U73825	Osteosarcoma, Colorectal cancer, Glioma, Schizophrenia, Type II diabetes mellitus, Liver carcinoma, Lung neoplasm, Type I diabetes mellitus, Hemochromatosis, Pulmonary hypertension	https://www.targetvalidation.org/target/ENSG00000113658/associations	STUB1<br>SMURF1<br>SMURF2<br>COPS5<br>SMAD1<br>RUNX2<br>SMAD4<br>ACVR1<br>BMPR1A<br>BMP6<br>FLNA<br>PRKCA<br>SKIL SKI<br>BMP2<br>BMPR1B<br>SMAD4	Cytoplasm, Nucleus,	No		✓
SMAD9	HUMAN Mothers against decapentaplegic homolog 9	MADH6	O15198	ENSG00000	Kinase,Transcription factor,Signalling	AL138706	Pulmonary hypertension, Idiopathic familial pulmonary arterial hypertension, Breast carcinoma, Primary ciliary dyskinesia, Familial primary pulmonary hypertension, Progressive disorder, Myocardial infarction, Duchenne muscular dystrophy, Glioma, Aprosencephaly cerebellar dysgenesis	https://www.targetvalidation.org/target/ENSG00000120693/associations	SMURF1<br>SMURF2<br>SMG1<br>MAPK14<br>SMAD4<br>ACVR1<br>BMPR1A<br>BMP2<br>BMPR1B<br>SMAD4<br>SMAD2	Cytoplasm	No		✓
SMARCD1	HUMAN SWI/SNF-related matrix-associated actin-dependent regulator of chromatin subfamily D member 1	BAF60A	Q96GM5	ENSG00000	Cancer	BC009368	Breast carcinoma, Colorectal adenocarcinoma, Lung carcinoma, Hyperlipidemia, Non-small cell lung carcinoma, Gastric carcinoma, Melanoma, Basal cell carcinoma, Esophageal squamous cell carcinoma, Hepatocellular carcinoma	https://www.targetvalidation.org/target/ENSG00000066117/associations	SMARCE1<br>SMARCC1<br>PHF10<br>SMARCC2<br>PBRM1<br>SMARCA4<br>NR3C1<br>ACTL6A<br>ARID1A<br>SMARCB1<br>NR3C1<br>SMARCA4<br>BRD7	Nucleus	No		✓
SMN2	HUMAN Survival motor neuron protein	GEMIN1, SMA, SMA1, SMA2, SMA3, SMNT, SMN, SMNC, SMN2	Q16637	ENSG00000	Signalling	AK289669	Proximal spinal muscular atrophy, Proximal spinal muscular atrophy type 3, Juvenile spinal muscular atrophy, Respiratory failure, Muscle cramp, Ventricular septal defect, Genetic neuromuscular disease, Genetic motor neuron disease, Kallmann syndrome, Distal hereditary motor neuropathy type 7	https://www.targetvalidation.org/target/ENSG00000205571/associations	DDX20<br>USP9X<br>GEMIN6<br>GEMIN5<br>MIB1<br>TIAL1<br>COPA<br>COIL<br>UCHL1<br>TP53<br>POP7<br>ZEB2<br>HNRNPR<br>PPM1G<br>GEMIN2<br>FUBP1<br>MAP3K5<br>OSTF1<br>GEMIN7	Nucleus, gem	No		✓
SNCA	HUMAN Alpha-synuclein	NACP, PARK1, PARK4	P37840	ENSG00000	Others	D31839	Young adult-onset Parkinsonism, Dementia, Lewy body dementia, Parkinson's disease, Neurodegenerative disorder, AL amyloidosis, Glioma, Amyloidosis, Astrocytoma, Li-Fraumeni syndrome	https://www.targetvalidation.org/target/ENSG00000145335/associations	CAMK2A<br>STUB1<br>PLK2<br>PLK3<br>PINK1<br>PARK7<br>KLK6<br>SIAH1<br>LRRK2<br>SNCB<br>PTK2B<br>DYRK1A<br>PIN1<br>PRKCE<br>SLC6A3<br>CSNK2A1<br>PPP2CA<br>PPP2CB<br>PLK1<br>CSNK1D<br>CSNK1A1<br>MAPK8<br>SYK GRK6<br>GRK5<br>HSPA4<br>AKT1<br>SLC6A4<br>MAPK1<br>MAPK3	Cytoplasm, cytosol	Yes	https://www.bindingdb.org/uniprot/P37840	✓
SNX6	HUMAN Sorting nexin-6	SNX6	Q9UNH7	ENSG00000	Cancer,Cancer-Antigen	AL445883	Rheumatoid arthritis, Asthma, Juvenile dermatomyositis, Pulmonary mucoepidermoid carcinoma, Alzheimer's disease, Ependymoma, Holoprosencephaly, Rheumatoid arthritis, Systemic lupus erythematosus, Leukemia	https://www.targetvalidation.org/target/ENSG00000129515/associations	BRMS1<br>ACVR2B<br>SNX1<br>TGFBR2<br>ACVRL1<br>TGFBR1<br>PDGFRA<br>PIM1 INSR<br>EGFR<br>SNX4<br>SNX2<br>BMPR1B<br>TGFBR2<br>TGFBR1<br>GIT1 SNX1<br>SNX2<br>SNX4	Early endosome	No		✓
SOCS2	HUMAN Suppressor of cytokine signaling 2	CIS2, Cish2, SOCS-2, SSI-2, SSI2, STATI2	O14508	ENSG00000	Kinase,Signalling	AF037989	Obesity, Narcolepsy, Schizophrenia, Myeloproliferative disease, Polycythemia vera, Respiratory syncytial virus infection, Asthma, Mammary neoplasm, MODY, Diabetes mellitus	https://www.targetvalidation.org/target/ENSG00000120833/associations	PTK2 GHR<br>IGF1R<br>INSR<br>SOCS3<br>EPOR<br>IGF1R<br>LEPR<br>INSR<br>PRLR<br>SOCS3<br>GHR<br>PTK2B<br>CUL5		No		✓
SOCS3	HUMAN Suppressor of cytokine signaling 3	CIS3, Cish3, SOCS-3, SSI-3	O14543	ENSG00000	Kinase	BC060858	Leukemia, Breast carcinoma, Liver carcinoma, Atherosclerosis, Myocardial ischemia, Endometrial neoplasm, Mammary neoplasm, Obesity, Prostatic neoplasm, Atopic dermatitis	https://www.targetvalidation.org/target/ENSG00000184557/associations	TRAF6<br>CSF3R<br>MAPK11<br>ELAVL1<br>STAT4<br>PTPN11<br>CXCR4<br>JAK3<br>CSNK1E<br>LEPR<br>STAT5A<br>IL6ST AXL<br>JAK1<br>RASA1<br>EPOR<br>IL2RB<br>SRC IL6R<br>IGF1R<br>CSF1R<br>LCK INSR<br>APP EGFR<br>JAK2<br>MAP3K7<br>SOCS2<br>EPOR LCK<br>MAP3K7<br>STAT5A<br>JAK2 JAK1<br>MAPK8IP1		No		✓
SOCS5	HUMAN Suppressor of cytokine signaling 5	CIS6, Cish5, CISH6, KIAA0671, SOCS-5	O75159	ENSG00000	Kinase,Signalling	AK290194	Allergic Conjunctivitis, Type I diabetes mellitus, Autoimmune disease, Atopic dermatitis, Eczema, Filarial elephantiases, Uveitis, Anaplastic thyroid carcinoma, Asthma, Cervial carcinoma	https://www.targetvalidation.org/target/ENSG00000171150/associations	KIT APP<br>EGFR<br>EGFR APP<br>IL4R ELOC		No		✓

SOCS6	HUMAN Suppressor of cytokine signaling 6	CIS4, Cish4, HSPC060, SOCS4, SSI4, STAI4, STATI4	O14544	ENSG0000	Kinase,Signalling	NA	Central nervous system cancer, Astrocytoma, Gastric carcinoma, Colorectal carcinoma, Colorectal cancer, Alzheimer's disease, Liver carcinoma, Glioblastoma multiforme, Lung carcinoma, Melanoma	https://www.targetvalidation.org/target/ENSG00000170677/associations	ACVR1 TGFBR1 KIT INSR BMPR1B LCK YAP1 FLT3 KIT		No		✓
SOD1	HUMAN Superoxide dismutase [Cu-Zn]	ALS, ALS1	P00441	ENSG0000	Signalling, Cancer	X01780	Neurodegenerative disease, Motor neuron disease, Amyotrophic lateral sclerosis, Familial amyotrophic lateral sclerosis, Sporadic amyotrophic lateral sclerosis, Depressive disorder, Nerve Degeneration, Down syndrome, Type II diabetes mellitus, Parkinson's disease	https://www.targetvalidation.org/target/ENSG00000142168/associations	AMFR IL7R CCS HSPA8 PPP3CA BCL2 RAC1 SQSTM1 HSPA4 COMMD1 KARS PDIA2 RNF19A HOXB2	Cytoplasm	Yes		✓
												https://www.bindingdb.org/uniprot/P00441	
SOD2	HUMAN Superoxide dismutase [Mn], mitochondrial	SODM	P04179	ENSG0000	Signalling, Cancer	X07834	Diabetic nephropathy, Mammary neoplasm, Type II diabetes mellitus, Schizophrenia, Parkinson's disease, Esophageal neoplasm, Squamous cell carcinoma, Acute monocytic leukemia, PGM-CDG, Barth syndrome	https://www.targetvalidation.org/target/ENSG00000112096/associations	IL7R CDK4 RAB4A USP36	Mitochondrion matrix,	Yes		✓
												https://www.drugbank.ca/biodb/polypeptides/P04179	
SORD	HUMAN Sorbitol dehydrogenase	DHSO	Q00796	ENSG0000	Signalling	U67243	Non-syndromic congenital cataract, Early-onset non-syndromic cataract, Nuclear cataract, Zonular cataract, Total congenital cataract, Necrotizing enterocolitis, Diabetic retinopathy, Hepatic necrosis, Isolated ectopia lentis, Prostate carcinoma	https://www.targetvalidation.org/target/ENSG00000140263/associations		Mitochondrion membrane	Yes		✓
												https://www.bindingdb.org/uniprot/Q00796	
SOX10	HUMAN Transcription factor SOX-10	DOM, WS2E, WS4	P56693	ENSG0000	Transcription factor	AK300945	Waardenburg syndrome, Waardenburg syndrome type 2, Waardenburg-Shah syndrome, Leukodystrophy, Neurologic Waardenburg-Shah syndrome, Kallmann syndrome, Hirschsprung disease, Schizophrenia, Yemenite deaf-blind hypopigmentation syndrome, Sensorineural Hearing Loss, Hermansky-Pudlak syndrome	https://www.targetvalidation.org/target/ENSG00000100146/associations	NRBP2 AURKB AGER NMI PAK1 CDK5 NEK4 CDK7 MAPK9 MAPK1 PAX3 PIM1 RAF1 CHEK2	Cytoplasm	No		✓
SOX15	HUMAN Protein SOX-15	SOX20, SOX26, SOX27	O60248	ENSG0000	Transcription factor	CH471108	Prostate carcinoma, Becker muscular dystrophy, Central serous chorioretinopathy, Polycystic ovary syndrome, Lung neoplasm, Malignant mesothelioma, Pancreatic adenocarcinoma, Barrett's esophagus, Gastric carcinoma, Squamous cell carcinoma	https://www.targetvalidation.org/target/ENSG00000129194/associations		Nucleus	No		✓
SOX2	HUMAN Transcription factor SOX-2	SOX2	P48431	ENSG0000	Transcription factor,Cancer	NA	Syndromic microphthalmia, Esophageal malformation, Anophthalmos, Septo-Optic Dysplasia, Microphthalmos, Microphthalmos, Hypogonadotropic hypogonadism, Waardenburg syndrome type 2, Schizophrenia, Bile duct carcinoma	https://www.targetvalidation.org/target/ENSG00000181449/associations	NOTCH1 PRKCI BRCA1 CTNNB1 TLX1 POU2F1 PARP1 HDAC1 ATG10 SUMO1 ATOH1 TBX2 POU5F1	Nucleus,	No		✓
SP1	HUMAN Transcription factor Sp1	TSFP1	P08047	ENSG0000	Transcription factor	CH471054	Gas trapping measurement, Isolated anophthalmia - microphthalmia, Blackfan-Diamond anemia, Colobomatous microphthalmia, Congenital primary aphakia, Oculotrichoanal syndrome	https://www.targetvalidation.org/target/ENSG00000185591/associations	E2F7 CREBBP HDAC2 HIF1A SMAD2 ATR SMAD4 ATM EP300 FOXM1 PRKCZ RELA SP3 TFAP4 SMAD3 PRKDC CSNK2A1 PPP2CA PTEN MAPK8 AKT1 PML MAPK1 MAPK3 CSNK2A2 ETS1 TXN AR LYN HSP90AA1 CDK1 RB1 TFAP2A JUN	Nucleus, Cytoplasm,	No		✓

SP110	HUMAN Sp110 nuclear body protein	IFI41, IFI75	Q9HB58	ENSG0000(	Transcription factor	AF280095	Primary immunodeficiency, Hepatic veno-occlusive disease - immunodeficiency, Colorectal adenocarcinoma, Autosomal recessive predisposition, Endomyocardial Fibrosis, IgG deficiency, Chronic interstitial cystitis, Juvenile dermatomyositis, Psoriasis, Tuberculosis	https://www.targetvalidation.org/target/ENSG00000135899/associations		Nucleus		No	✓
SP2	HUMAN Transcription factor Sp2	KIAA0048	Q02086	ENSG0000(	Transcription factor	BC016680	Squamous cell carcinoma, Chronic Hepatitis B infection, Chronic lymphocytic leukemia, Melanoma, Migraine disorder, Myopia, Mandibuloacral dysplasia, Amyotrophic lateral sclerosis, Neuroblastoma, Irritable bowel syndrome	https://www.targetvalidation.org/target/ENSG00000167182/associations	IRF1	Nucleus,		No	✓
SPA17	HUMAN Sperm surface protein Sp17	CT22, SP17	Q15506	ENSG0000(	Others	AK311806	Azoospermia, Lung carcinoma, Rheumatoid arthritis, Multiple myeloma, Esophageal neoplasm, Non-small cell lung carcinoma, Epithelial ovarian cancer, Ovarian carcinoma, Breast carcinoma, Ependymoma	https://www.targetvalidation.org/target/ENSG00000064199/associations	AKAP3 ROPN1	Membrane		No	✓
SPAG4	HUMAN Sperm-associated antigen 4 protein	CT127, SUN4	Q9NPE6	ENSG0000(	Others	AF043344	Renal cell carcinoma, Clear cell renal carcinoma, Breast carcinoma, Glioblastoma multiforme, Ulcerative colitis, Lung carcinoma, Coronary heart disease, Idiopathic pulmonary fibrosis, Medulloblastoma, Pancreatic carcinoma	https://www.targetvalidation.org/target/ENSG00000061656/associations	TBCD ODF1	Membrane	Transmembrane	No	✓
SPAG9	HUMAN C-Jun-amino-terminal kinase-interacting protein 4	CT89, HLC6, HSS, JLP, KIAA0516, PHET, SYD1, JIP4	O60271	ENSG0000(	Kinase	AK024068	Male infertility with azoospermia or oligozoospermia due to single gene mutation, Non-syndromic male infertility due to sperm motility disorder, Renal cell carcinoma, Systemic scleroderma, Breast carcinoma,<br>Liver carcinoma, Thyroid carcinoma, Kallmann syndrome, Oculocutaneous albinism type 1A, Griscelli disease	https://www.targetvalidation.org/target/ENSG00000008294/associations	MAP3K3 CDON MAPK14 KLC1 ARF6 MAP2K4 MAPK8 CDK2 MAX MAPK14 MAP3K3 MAPK11 MAP2K4 EXOC4 KLC1 PIKFYVE	Cytoplasm, Cytoplasm, perinuclear region,		No	✓
SPANXN1	HUMAN Sperm protein associated with the nucleus on the X chromosome N1	SPANX-N1	Q5VSR9	ENSG0000(	Others	DQ336119	Li-Fraumeni syndrome, Glioblastoma multiforme, Mental or behavioural disorder, Response to simvastatin, PCKS9 protein measurement	https://www.targetvalidation.org/target/ENSG00000203923/associations				No	✓
SPANXN2	HUMAN Sperm protein associated with the nucleus on the X chromosome N2	SPANX-N2	Q5MJ10	ENSG0000(	Others	DQ336113	Tuberculosis, Glioblastoma multiforme, Influenza, Response to reverse transcriptase inhibitor, Peripheral neuropathy, HIV-1 infection	https://www.targetvalidation.org/target/ENSG00000268988/associations				No	✓
SPANXN4	HUMAN Sperm protein associated with the nucleus on the X chromosome N4	SPANX-N4	Q5MJ08	ENSG0000(	Others	AL137840	Glioblastoma multiforme, Li-Fraumeni syndrome, Smoking behavior, 3-hydroxypropylmercapturic acid measurement	https://www.targetvalidation.org/target/ENSG00000189326/associations				No	✓
SPARC	HUMAN SPARC	ON	P09486	ENSG0000(	Signalling, Cancer	CR456799	Osteogenesis imperfecta, Osteoporosis, Ovarian neoplasm, Colonic neoplasm, Acute myelocytic leukemia, Intervertebral disc degeneration, Multiple myeloma, Adenocarcinoma, Mucopolysaccharidosis type 4B, Brachyolmia	https://www.targetvalidation.org/target/ENSG00000113140/associations	ILK CTSK MMP3 THBS1 FGA COL3A1 TGFB1 PLG TGM2 PDGFB PDGFA VEGFA CASP8 VCAM1 STAB1	Secreted, extracellular space, extracellular matrix, basement membrane		No	✓

SPARCL1	HUMAN SPARC-like protein 1	MAST9	Q14515	ENSG0000(	Cancer	AC093906	Lung carcinoma, Lung adenocarcinoma, Breast carcinoma, Endometriosis, Epilepsy, Schizophrenia, Multiple sclerosis, Prostate carcinoma, Ovarian carcinoma, Glioma	https://www.targetvalidation.org/target/ENSG00000152583/associations		Secreted, extracellular space, extracellular matrix		No		✓
SPATA19	HUMAN Spermatogenesis-associated protein 19, mitochondrial	SPAS1, spergen1	Q7Z5L4	ENSG0000(	Others	BC058039	Azoospermia, Teratozoospermia, Prostatic neoplasm, Benign prostatic hyperplasia, Prostate carcinoma, Basal cell carcinoma, Invasive ductal carcinoma	https://www.targetvalidation.org/target/ENSG00000166118/associations		Mitochondrion outer membrane		No		✓
SPATA25	HUMAN Spermatogenesis-associated protein 25	C20orf165, TSG23	Q9BR10	ENSG0000(	Others	AL008726	Brachyolmia, Achondrogenesis, Azoospermia, Cervical carcinoma, Thoracomelic dysplasia, Smith-Lemli-Optiz syndroome, Braddock syndrome, CHILD syndrome, Focal dermal hypoplasia, Hurler syndrome	https://www.targetvalidation.org/target/ENSG00000149634/associations		Membrane	Transmembrane	No		✓
SPDEF	HUMAN SAM pointed domain-containing Ets transcription factor	bA375E1.3, PDEF	O95238	ENSG0000(	Transcription factor	BX255971, AL157372	Inflammatory bowel disease, Ovarian neoplasm, Mammary neoplasm, Prostatic neoplasm, Breast carcinoma, Prostate carcinoma, Gastric carcinoma, Rotor syndrome, Inflammatory bowel disease, Dubin-Johnson syndrome	https://www.targetvalidation.org/target/ENSG00000124664/associations	AR NKX3-1 AR	Nucleus		No		✓
SPEG	HUMAN Striated muscle preferentially expressed protein kinase	APEG1, KIAA1297	Q15772	ENSG0000(	Kinase	CR542201	Centronuclear myopath, Autosomal recessive centronuclear myopathy, Autosomal recessive predisposition, Facial paresis, Severe hypotonia, Axial muscle weakness, Infantile onset, Hypoplastic mandible condyle, Retrognathia, Prostate carcinoma	https://www.targetvalidation.org/target/ENSG00000072195/associations		Nucleus,		No		✓
SPG20	HUMAN Spartin	KIAA0610, TAHCCP1	Q8N0X7	ENSG0000(	Others	AY038359	Hereditary spastic paraplegia, Complex hereditary spastic paraplegia, Autosomal recessive spastic paraplegia type 20, Microcephaly, Troyer syndrome, Distal amyotrophy, Congenital pes cavus, Congenital camptodactyly, Patellar clonus, Short stature	https://www.targetvalidation.org/target/ENSG00000133104/associations	TUBA1A EPS15 PLIN3 ITCH	Cytoplasm		No		✓
SPIB	HUMAN Transcription factor Spi-B	SPI-B	Q01892	ENSG0000(	Transcription factor,Signalling	AK300639	Biliary liver cirrhosis, Primary biliary cirrhosis, Hyperpigmentation, Liver fibrosis, Dyslipidemia, Conjugated hyperbilirubinemia, Symptomatic dermographism, Hanot's cirrhosis, Colorectal cancer, Psoriasis	https://www.targetvalidation.org/target/ENSG00000269404/associations	CSNK2A1 MAPKAPK2 MAPK8 MAPK3 CSNK2A2	Nucleus		No		✓
SPIC	HUMAN Transcription factor Spi-C	MGC40611, SPI-C	Q8N5J4	ENSG0000(	Transcription factor	NA	Familial hemophagocytic lymphohistiocytosis, Beta-Thalassemia associated with another hemoglobin anomaly, X-linked lymphoproliferative disease, Psoriasis, Hepatocellular carcinoma, Colorectal carcinoma, Colorectal cancer, Macroglobulinemia, Cardiomyopathy	https://www.targetvalidation.org/target/ENSG00000166211/associations		Nucleus		No		✓
SPO11	HUMAN Meiotic recombination protein SPO11	CT35, SPATA43, TOPVIA	Q9Y5K1	ENSG0000(	Others	AL135939	Male infertility with teratozoospermia due to single gene mutation, Male infertility with azoospermia or oligozoospermia due to single gene mutation, Normosmic congenital hypogonadotropic hypogonadism, Kallmann syndrome, Primary ovarian insufficiency, Azoospermia, Premature menopause, WARG syndrome	https://www.targetvalidation.org/target/ENSG00000054796/associations		Nucleus		No		✓

SQSTM1	HUMAN Sequestosome-1	A170, OSIL, p60, p62, PDB3, ORCA	Q13501	ENSG0000(	Others	AK312451	Neurodegenerative diseas, Frontotemporal dementia with motor neuron disease, Osteitis deformans, Congenital disorder of glycosylation, Amyotrophic lateral sclerosis, Osteitis deformans, Nonaka myopathy, Skeletal muscle atrophy, Bone disease, Type I diabetes mellitus	https://www.targetvalidation.org/target/ENSG00000161011/associations	IKBKG TRAF6 TBK1 MAP1LC3A GABARAPL1 MAP1LC3B MYO3A WDFY3 SPRED2 NTRK2 NTRK3 NFE2L2 TRADD NBR1 RIPK1 MAP2K5 TARDBP PRKCZ ACVR1 GABARAPL2 ATXN3 IRAK1 PRKCI SNCA TGFBR1 IRS1 PDE4A	Cytoplasm, Late endosome, Lysosome, Cytoplasmic vesicle, autophagosome, Nucleus, Endoplasmic reticulum, Cytoplasm, P-body,	No		✓
SRM	HUMAN Spermidine synthase	SPS1, SRML1	P19623	ENSG0000(	Kinase	CH471130	Squamous cell carcinoma, Lung carcinoma, Liver carcinoma, Prostate carcinoma, Breast carcinoma, Liver neoplasm, Hypertensive disease, Glioma, Adenocarcinoma, Hypercholesterolemia	https://www.targetvalidation.org/target/ENSG00000116649/associations			Yes	https://www.bindingdb.org/uniprot/P19623	✓
SRP19	HUMAN Signal recognition particle 19 kDa protein	SRP19	P09132	ENSG0000(	Cancer,Cancer-Antigen	AC008536	Myopathy, Breast carcinoma, Adenomatous polyposis coli	https://www.targetvalidation.org/target/ENSG00000153037/associations	SRP54 SRP54	Cytoplasm,	No		✓
SRPK1	HUMAN SRSF protein kinase 1	SFRSK1	Q96SB4	ENSG0000(	Kinase	Z99128	Psoriasis, Breast carcinoma, Melanoma, Ovarian carcinoma, Glioma, Germ cell tumor, Gastrointestinal carcinoid tumor, Sepsis, Glioblastoma multiforme, CINCA syndrome	https://www.targetvalidation.org/target/ENSG00000096063/associations	IKBKG STK11 CSNK2A1 CSNK2B CLK1 CSNK2A2 YWHAG MDM2 HNRNPK PPP1R13L MDM4 SRSF1 YWHAB NFATC4 MBP RBM4 SRSF3 RBM8A LBR U2AF2 PRM1 SNRNP70 SRRM1 SCAF11 SAFB SREK1 SRSF6 SRSF5 SRSF8	Cytoplasm, Nucleus, Nucleus matrix, Microsome,	Yes	https://www.bindingdb.org/uniprot/Q96SB4	✓
SRPK2	HUMAN protein kinase 2	SRS protein kinase 2, SFRS protein kinase 2	P78362	ENSG0000(	Kinase	AC004884	Schizophrenia, Melanoma, Alzheimer's disease, Hepatitis B infection, Lung adenocarcinoma, Lung squamous cell carcinoma, Systemic scleroderma, Breast adenocarcinoma, Pathological myopia, Hypertriglyceridemia	https://www.targetvalidation.org/target/ENSG00000135250/associations	YWHAB AKT1 PRKACA PRKCD SMAD4 RPS6KA2 MTA1 EIF4B BCL3 MDM2 CAMKK1 ACIN1 SRSF1 NFATC4 ZRANB2 SRSF2 U2AF2 ASAP2 PRPF40A	Cytoplasm, Nucleus,	Yes	https://www.bindingdb.org/uniprot/P78362	✓
SRPX2	HUMAN Sushi repeat-containing protein SRPX2	SRPUL	O60687	ENSG0000(	Others	AL035608	Rolandic epilepsy - speech dyspraxia, Polymicrogyria, Bilateral perisylvian polymicrogyria, Glioblastoma multiforme, Malignant mesothelioma, Ovarian carcinoma, Cystic fibrosis, Pancreatic ductal adenocarcinoma, Medulloblastoma, Amyotrophic lateral sclerosis	https://www.targetvalidation.org/target/ENSG00000102359/associations	HGF	Secreted, Cytoplasm, Cell surface, Cell junction, synapse	No		✓
SRSF5	HUMAN Serine/arginine-rich splicing factor 5	HRS, SFRS5, SRP40	Q13243	ENSG0000(	Cancer,Cancer-Antigen	BT007089	Lung carcinoma, Large cell lung carcinoma, Melanoma, Breast carcinoma, Neurotoxicity Syndrome, Hodgkin's disease, Dementia, Brugada syndrome, Pick disease, Bipolar disorder	https://www.targetvalidation.org/target/ENSG00000100650/associations	SRPK1 TNK2	Nucleus,	No		✓
SS18L1	HUMAN Calcium-responsive transactivator	KIAA0693, CREST	O75177	ENSG0000(	Cancer	AK125656	Melanoma, Lung carcinoma, Prostate carcinoma, Synovial sarcoma, Cerebral hemorrhage, Intracranial hemorrhage, Subarachnoid hemorrhage, Systemic scleroderma, Scleroderma, Telangiectasis	https://www.targetvalidation.org/target/ENSG00000184402/associations	CREBBP	Nucleus	No		✓
SSB	HUMAN Lupus La protein	La, La/SSB	P05455	ENSG0000(	Others	AB451228	Arthritis, Hepatitis C infection, Systemic lupus erythematosus, Sjogren's syndrome, Hepatitis B infection, Congenital heart block, Complete atrioventricular block, Autoimmune disease, Myocardial ischemia, Neonatal hemochromatosis	https://www.targetvalidation.org/target/ENSG00000138385/associations	VRK1 MAP3K14 CSNK2A1 CSNK2A2 GZMB NCL TROVE2	Nucleus	No		✓
SSBP4	HUMAN Single-stranded DNA-binding protein 4	SSBP4	Q9BWG4	ENSG0000(	Transcription factor	NA	Tuberculosis, Neuroblastoma, Acute monocytic leukemia, Pancreatic ductal adenocarcinoma	https://www.targetvalidation.org/target/ENSG00000130511/associations	CDKL5	Nucleus	No		✓

SSNA1	HUMAN Sjoegren syndrome nuclear autoantigen 1	N14, NA14	O43805	ENSG0000(	Cancer,Cancer-Antigen	AL929554	Autoimmune disease, Vasculitis, Rheumatic disease, Diabetes mellitus, Peeling skin syndrome, Systemic scleroderma, Dermatomyositis, Melanoma, Rheumatoid arthritis, Polymyositis	https://www.targetvalidation.org/target/ENSG00000176101/associations	SPAST	Nucleus, Cytoplasm, cytoskeleton, microtubule organizing center, centrosome,	No	✓
SSX1	HUMAN Protein SSX1	CT5.1	Q16384	ENSG0000(	Transcription factor,Cancer	BC125151	Sarcoma, Melanoma, Synovial sarcoma, Alzheimer's Disease, Rhabdomyosarcoma, Liver carcinoma, Leukemia, Somatic mutation, Squamous cell lung carcinoma, Cutaneous melanoma	https://www.targetvalidation.org/target/ENSG00000126752/associations			No	✓
SSX2	HUMAN Protein SSX2	HOM-MEL-40, SSX2A, SSX2B	Q16385	ENSG0000(	Transcription factor,Cancer	AL445236, AL450023	Prostate carcinoma, Sarcoma, Synovial sarcoma, Somatic mutation, Prostatic neoplasm, Liver carcinoma, Melanoma, Breast carcinoma, Ewings sarcoma, Multiple myeloma	https://www.targetvalidation.org/target/ENSG00000268447/associations		Nucleus,	No	✓
SSX2b	HUMAN Protein SSX2	HOM-MEL-40, SSX2A, SSX2B	Q16385	ENSG0000(	Transcription factor,Cancer	AL445236, AL450023	Prostate carcinoma, Sarcoma, Synovial sarcoma, Somatic mutation, Prostatic neoplasm, Liver carcinoma, Melanoma, Breast carcinoma, Ewings sarcoma, Multiple myeloma	https://www.targetvalidation.org/target/ENSG00000268447/associations		Nucleus,	No	✓
SSX3	HUMAN Protein SSX3	CT5.3	Q99909	ENSG0000(	Transcription factor,Cancer,Cancer-Antigen	CH471164	Li-Fraumeni syndrome, Azoospermia, Melanoma, Synovial sarcoma, Diffuse large B-cell lymphoma, Central nervous system cancer, Breast carcinoma	https://www.targetvalidation.org/target/ENSG00000165584/associations			No	✓
SSX4	HUMAN Protein SSX4	CT5.4, SSX4A, SSX4B	O60224	ENSG0000(	Transcription factor,Cancer	BC005325	Synovial sarcoma, Monophasic synovial sarcoma, Melanoma, Brain neoplasm, Multiple myeloma, Intrahepatic cholangiocarcinoma	https://www.targetvalidation.org/target/ENSG00000268009/associations			No	✓
SSX4B	HUMAN Protein SSX4	CT5.4, SSX4A, SSX4B	O60224	ENSG0000(	Transcription factor,Cancer	BC005325	Synovial sarcoma, Monophasic synovial sarcoma, Melanoma, Brain neoplasm, Multiple myeloma, Intrahepatic cholangiocarcinoma	https://www.targetvalidation.org/target/ENSG00000268009/associations			No	✓
SSX5	HUMAN Protein SSX5	SSX5	O60225	ENSG0000(	Transcription factor	AL356464, AL683817	Melanoma, Papillary thyroid carcinoma, Monoclonal gammopathy, Liver carcinoma	https://www.targetvalidation.org/target/ENSG00000165583/associations			No	✓
SSX7	HUMAN Protein SSX7	SSX7	Q7RTT5	ENSG0000(	Transcription factor	NA	Diabetes mellitus, Glioblastoma multiforme	https://www.targetvalidation.org/target/ENSG00000187754/associations			No	✓
STAM	HUMAN Signal transducing adapter molecule 1	STAM1	Q92783	ENSG0000(	Signalling	CH471072	Listeriosis, Fibrosis, Non-alcoholic fatty liver disease, Liver Cirrhosis, Hepatocarcinogenesis, Steatohepatitis, Ependymoma, Juvenile dermatomyositis, Lymphangioleiomyomatosis, Colorectal adenocarcinoma	https://www.targetvalidation.org/target/ENSG00000136738/associations	CAMK2A JAK3 ARRB1 JAK1 UBC STAMBP JAK2 TIMM8A JAK2 JAK1 JAK3 HGS	Cytoplasm	No	✓
STAM2	HUMAN Signal transducing adapter molecule 2	Hbp	O75886	ENSG0000(	Signalling	AK292265	Listeriosis, Azoospermia, Chemotherapy-induced alopecia, Breast carcinoma, Abscess, Coinfection, Chemotherapy-induced-alopecia, Melanoma, Breast carcinoma, Medulloblastoma	https://www.targetvalidation.org/target/ENSG00000115145/associations	JAK3 TYK2 JAK1 PTPN1 SRC FYN EGFR JAK2 UBC JAK2 JAK1 TYK2 JAK3 HGS	Cytoplasm	No	✓
STAP1	HUMAN Signal-transducing adaptor protein 1	BRDG1, STAP-1	Q9ULZ2	ENSG0000(	Kinase	BC014958	Familial hypercholesterolemia, Breast carcinoma, Clear cell renal carcinom, Osteosarcoma, Ovarian carcinoma, Hyperlipoproteinemia type 2A, Atypical ductal breast hyperplasia, Acute monocytic leukemia, Azoospermia, Multiple myeloma	https://www.targetvalidation.org/target/ENSG00000035720/associations	PTK2B TEC KIT KIT	Nucleus	No	✓

STARD7	HUMAN StAR-related lipid transfer protein 7, mitochondrial	GTT1	Q9NQZ5	ENSG0000(	Cancer,Cancer-Antigen	CH471207	Diffuse large B-cell lymphoma, Breast carcinoma, Malignant peripheral nerve sheath tumor, Ovarian carcinoma, Psoriasis, Choriocarcinoma, Liver carcinoma, Hydatidiform mole, Type II diabetes mellitus, Hepatocellular carcinoma	https://www.targetvalidation.org/target/ENSG00000084090/associations		Mitochondrion	No		✓
STAT1	HUMAN Signal transducer and activator of transcription 1-alpha/beta	ISGF-3, STAT91	P42224	ENSG0000(	Kinase,Transcription factor	AY865620	Primary immunodeficiency, Chronic mucocutaneous candidosis, Mendelian susceptibility to mycobacterial diseases, Mendelian susceptibility to mycobacterial diseases due to partial STAT1 deficiency, Liver carcinoma, Osteoporosis, Rheumatoid arthritis, Mycobacterium Infection, Liver cirrhosis, Wiskott-Aldrich syndrome	https://www.targetvalidation.org/target/ENSG00000115415/associations	ALK MBD1 CNOT8 PRMT1 CREBBP PIAS4 MAVS IL27RA MAPK14 DDB1 IL11RA IKBKE CAMK2D CAMK2G CAMK2B HDAC1 ATM NMI TRIM28 EP300 PTPN11 PRKCD PTK2 ACVR1 RELA IRF9 FANCC CSNK2A1 SUMO1 CXCR4 STAT2 JAK3	Cytoplasm	Yes	https://www.bindingdb.org/uniprot/P42224	✓
STAT3	HUMAN Signal transducer and activator of transcription 3	APRF	P40763	ENSG0000(	Transcription factor,Cancer	AK291933	Autoimmune disease, Job's syndrome, Primary immunodeficiency, Squamous cell carcinoma, Prostate carcinoma, Inflammatory bowel disease, Lung carcinoma, Pancreatic ductal adenocarcinoma, Hyper-IgE syndrome, Head and Neck neoplasia	https://www.targetvalidation.org/target/ENSG00000168610/associations	PIAS3 ALK OGDHL IL20RA DAXX NLK NDUFA13 DUSP22 SH2B1 IL21R NFKBIZ GNL3 MAP3K14 CSF3R BICD1 KAT5 CREBBP HDAC2 MRPS31 DYRK2 MORC4 IL22RA1 PELP1 IL20RB ELP2 IL23R HES5 HIF1A MAPK14 NCOA1 PTPRR	Cytoplasm, Nucleus	Yes	https://www.bindingdb.org/uniprot/P40763	✓
STAT4	HUMAN Signal transducer and activator of transcription 4	STAT4	Q14765	ENSG0000(	Transcription factor,Cancer	NA	Systemic lupus erythematosus, Arthritis, Behcet Syndrome, Sjogren's syndrome, Systemic scleroderma, Liver carcinoma, Multiple sclerosis, Juvenile rheumatoid arthritis, Crohn's disease, Ulcerative colitis	https://www.targetvalidation.org/target/ENSG00000138378/associations	IL12RB2 CREBBP IL23R MAPK14 CSNK2A1 MAP2K6 LEPR IL12RB1 STAT3 TYK2 JUN PIAS2 JAK2 LAMTOR5 SOCS3 STAT3 SOCS3 IL12RB2	Cytoplasm, Nucleus,	No		✓
STAT5A	HUMAN Signal transducer and activator of transcription 5A	STAT5	P42229	ENSG0000(	Transcription factor,Cancer	AK301457	Li-Fraumeni syndrome, Hematological system disease, Adenocarcinoma, Noninfiltrating Intraductal carcinoma, Myocardial ischemia, Ductal carcinoma, Lobular carcinoma, Wiskott-Aldrich syndrome, Primary immunodeficiency, Familial prostate cancer	https://www.targetvalidation.org/target/ENSG00000126561/associations	CISH CRLF2 TSLP CREBBP NCOA1 ERBB4 IL15RA EP300 BTK PTPN11 TEK IL5RA IL9R PTEN JAK3 BMX STAT5B DUSP3 CRKL MAPK8 SYK STAT1 STAT3 MPL PPARG IL2RG TYK2 MAPK1 MAPK3 IL3RA ACP1 IL4R JAK1 PPIB	Cytoplasm	Yes	https://www.bindingdb.org/uniprot/P42229	✓
STAU1	HUMAN Double-stranded RNA-binding protein Staufen homolog 1	PPP1R150, STAU	O95793	ENSG0000(	Others	AF061940	Teratozoospermia, Melanoma, Ovarian carcinoma, Gastric carcinoma, Dermatomyositis, Lung adenocarcinoma, Multiple myeloma, Psoriasis, Colorectal cancer, Malignant peripheral nerve sheath tumor	https://www.targetvalidation.org/target/ENSG00000124214/associations	MAP3K14 DHX58 ROCK2 CASC3 TUBB ROCK2 AKT1 RPS6 UPF1 CDC42 PPP1CA	Cytoplasm	No		✓
STIP1	HUMAN Stress-induced-phosphoprotein 1	HOP, STI1	P31948	ENSG0000(	Cancer,Cancer-Antigen	CR536512	Melanoma, Psoriasis, Glioma, Asthma, Ovarian carcinoma, Lung carcinoma, Non-small cell lung carcinoma, Spinal cord ischemia, Papillary thyroid carcinoma, Colorectal cancer	https://www.targetvalidation.org/target/ENSG00000168439/associations	DYRK4 TNNI3K MAP4K2 IRAK1 IL15 HSPA8 HSPA1A PRNP CDC37	Cytoplasm	No		✓
STK10	HUMAN Serine/threonine-protein kinase 10	LOK, PRO2729	O94804	ENSG0000(	Kinase,Cancer,Cancer-Antigen	AC024561	Testicular germ cell tumor, Migraine disorder, Severe combined immunodeficiency, Dermatitis, Dermatologic disorder, Inflammatory dermatosis, Asthma, Chronic mucocutaneous candidosis, Melanoma, Ewings sarcoma	https://www.targetvalidation.org/target/ENSG00000072786/associations	BLM EZR PLK1 MSN RDX SUB1	Cell membrane	Yes	https://www.bindingdb.org/uniprot/O94804	✓

STK11	HUMAN Serine/threonine-protein kinase STK11	LKB1, PJS	Q15831	ENSG0000(	Kinase,Cancer	AF055327	Inherited cancer-predisposing syndrome, Peutz-Jeghers syndrome, Testicular germ cell tumor, Adenocarcinoma, Breast carcinoma, Squamous cell carcinoma, Hamartomatous polyp, Lung adenocarcinoma, Ductal adenocarcinoma	https://www.targetvalidation.org/target/ENSG00000118046/associations	CAB39 APPL1 RPS6KA6 STRADB SGK494 SIRT1 STRADA MARK2 RPS6KA1 RPS6KA2 ATM PRKCZ PRKAA2 RPS6KA3 MAPK1 MAPK3 RPS6KB1 PRKACA RPS6KA4 RPS6KA5 PRKCB PTEN PRKAA2 SMAD4 MARK2 TP53 SOX9 PRKAG1 STK38	Nucleus, Cytoplasm, Membrane Mitochondrion,	Yes	https://www.bindingdb.org/uniprot/Q15831	✓
STK16	HUMAN Serine/threonine-protein kinase 16	MPSK, PKL12	O75716	ENSG0000(	Kinase	AJ010872	Melanoma, Acute lymphoblastic leukemia, Albuminuria, Glioblastoma multiforme, Pancreatic carcinoma, Alzheimer's disease, Presenile dementia, Senile plaque, Dementia, Gastric carcinoma	https://www.targetvalidation.org/target/ENSG00000115661/associations	PRKACA RUVBL1 VHL RUVBL2 NPM1 TRIM28 CHAF1B SRF CBL ELK1 MBP EEF1D DRG1 ADAMTSL4 EIF4H KCTD17 NAGK RPIA	Cytoplasm, perinuclear region, Membrane	Yes	https://www.bindingdb.org/uniprot/O75716	✓
STK17B	HUMAN Serine/threonine-protein kinase 17B	DRAK2	O94768	ENSG0000(	Kinase	AB011421	Lung carcinoma, Breast carcinoma, Autoimmune disease, Idiopathic pulmonary fibrosis, Colorectal cancer, Breast carcinoma, Thyroid carcinoma, Papillary thyroid carcinoma, Multiple sclerosis, Azoospermia	https://www.targetvalidation.org/target/ENSG00000081320/associations	TGFBR1 STX1A CHP1 DAPK1 CAMKK2	Nucleus	Yes	https://www.bindingdb.org/uniprot/O94768	✓
STK24	HUMAN Serine/threonine-protein kinase 24	MST3, MST3/N, STK3	Q9Y6E0	ENSG0000(	Kinase	AL137249, AL356423	Self-reported educational attainment, Breast carcinoma, Glioblastoma multiforme, Bipolar Disorder,<br>Prostate carcinoma, Type II Pseudohypoaldosteronism, Thymoma, Inflammatory bowel disease, Pancreatic carcinoma, Hypertensive disease	https://www.targetvalidation.org/target/ENSG00000102572/associations	STK38L MAPK14 PRKX CASP3 PRKACA PRKY TFAM CASP3 CASP7 STK38 CASP8 STK38L PDCD10 HNRNPH3 HLCS	Cytoplasm, Nucleus, Membrane,	Yes	https://www.bindingdb.org/uniprot/Q9Y6E0	✓
STK25	HUMAN Serine/threonine-protein kinase 25	SOK1, YSK1	O00506	ENSG0000(	Kinase	AK291947	Non-alcoholic steatohepatitis, Breast carcinoma, Benign prostatic hyperplasia, Neuroblastoma, Prostate carcinoma, Central neuroblastoma, Albright's hereditary osteodystrophy, Pseudohypoparathyroidism, Epilepsy, Myopathy	https://www.targetvalidation.org/target/ENSG00000115694/associations	IKBKG PTPN13 PRKCD YWHAZ PDCD10 GOLGA2 CCM2	Cytoplasm	Yes	https://www.bindingdb.org/uniprot/O00506	✓
STK26	HUMAN Serine/threonine-protein kinase 26	MASK, MST4	Q9P289	ENSG0000(	Kinase	AB040057	Ovarian carcinoma, Central nervous system cancer, Glioma, Prostatic neoplasm, Liver carcinoma, Pituitary disease, Prostate carcinoma, Metastatic hepatocellular carcinoma, Glioblastome multiforme, Medulloblastoma	https://www.targetvalidation.org/target/ENSG00000134602/associations	CAB39 PRKACA EZR TRAF6 PDCD10	Cytoplasm	Yes	https://www.bindingdb.org/uniprot/Q9P289	✓
STK3	HUMAN Serine/threonine-protein kinase 3	KRS1, MST2	Q13188	ENSG0000(	Kinase	AK291837	Familial pancreatic carcinoma, Metastatic prostate cancer, Sarcoma, Colorectal carcinoma, Colorectal cancer, Breast carcinoma, Colon carcinoma, Sickle cell anemia, Idiopathic pulmonary fibrosis, Liver carcinoma	https://www.targetvalidation.org/target/ENSG00000104375/associations	AKT3 TAOK2 RASSF1 LATS2 SAV1 RASSF4 TAOK3 TAOK1 RASSF6 PPP2CA PPP2CB PPP1CB PPP1CA CASP3 PPP1CC AKT2 AKT1 SRC RAF1 ABL1 LATS1 WWTR1 PRKCA RAF1 YAP1 LATS1 CASP3 ABL1 NEK2 LATS2	Cytoplasm	Yes	https://www.bindingdb.org/uniprot/Q13188	✓
STK32A	HUMAN Serine/threonine-protein kinase 32A	YANK1, ST32A	Q8WU08	ENSG0000(	Kinase	NA	Breast carcinoma, Lung carcinoma, Melanoma, Central nervous system cancer, Glioma, Celiac disease, Oligodendroglioma, Autism, Prostate carcinoma, Papillary thyroid carcinoma	https://www.targetvalidation.org/target/ENSG00000169302/asso		Cell membrane, Lipid-anchor	Yes	https://www.bindingdb.org/uniprot/Q8WU08	✓

STK32B	HUMAN Serine/threonine-protein kinase 32B	STK32, YANK2, UNQ3003/PRO9744	Q9NY57	ENSG0000(	Kinase	AJ250839	Li-Fraumeni syndrome, Essential tremor, Cleft lip, Cleft palate, Ellis-Van Creveld syndrome, Mild mental retardation, Neoplasm metastasis, Astrocytoma, Glioblastoma multiforme, Celiac disease	https://www.targetvalidation.org/target/ENSG00000152953/associations				Yes	https://www.bindingdb.org/uniprot/Q9NY57	✓
STK32C	HUMAN Serine/threonine-protein kinase 32C	PKE, YANK3	Q86UX6	ENSG0000(	Kinase	AL590105, AL512622	Lung adenocarcinoma, Squamous cell carcinoma, Diarrhea, Type II diabetes mellitus, Mental depression, Dysentery, Macroglobulinemia, Unipolar depression, Hepatocellular carcinoma, Colorectal adenocarcinoma	https://www.targetvalidation.org/target/ENSG00000165752/associations				Yes	https://www.bindingdb.org/uniprot/Q86UX6	✓
STK33	HUMAN Serine/threonine-protein kinase 33	STK33	Q9BYT3	ENSG0000(	Kinase	AL833011	Li-Fraumeni syndrome, Obesity, Asthma, Type II diabetes mellitus, Breast carcinoma, Hepatocellular carcinoma, Multiple sclerosis, Colorectal adenocarcinoma, Ependymoma, Acute myeloid leukemia	https://www.targetvalidation.org/target/ENSG00000130413/associations	CRK	Cytoplasm, perinuclear region		Yes	https://www.bindingdb.org/uniprot/Q9BYT3	✓
STK38	HUMAN Serine/threonine-protein kinase 38	NDR	Q15208	ENSG0000(	Kinase	BC095413	Neoplasm of mature B-cells, Adenocarcinoma, Lymphoma, Febrile convulsions, Sepsis, Septicemia, Seizures, Gastric carcinoma, Spina bifida, Thrombocytopenia	https://www.targetvalidation.org/target/ENSG00000112079/associations	STK24<br>STK11<br>STK4<br>GSK3B<br>PRKACA<br>CTTN<br>YAP1<br>MAP3K1<br>MAP3K2<br>RAD23B<br>YWHAZ<br>MTA1<br>MDM2<br>PPP1R13L<br>YWHAB<br>NFATC4<br>CTDP1<br>CDCA5<br>RAB3IP<br>S100B<br>MOB2<br>HLCS	Nucleus, Cytoplasm,		Yes	https://www.bindingdb.org/uniprot/Q15208	✓
STK38L	HUMAN Serine/threonine-protein kinase 38-like	KIAA0965, NDR2	Q9Y2H1	ENSG0000(	Kinase	AC092827	Malignant mesothelioma, Colorectal adenocarcinoma, Lung carcinoma, Lung adenocarcinoma, Tuberculosis, Neoplasm metastasis, Tuberculosis, Medulloblastoma, Retinal dystrophy, Glioblastoma multiforme	https://www.targetvalidation.org/target/ENSG00000211455/associations	STK24<br>CTTN<br>PRKCD<br>EIF4B<br>NUAK1<br>YWHAB<br>STK24<br>RAB3IP	Cytoplasm, cytoskeleton, Membrane,		Yes	https://www.bindingdb.org/uniprot/Q9Y2H1	✓
STK4	HUMAN Serine/threonine-protein kinase 4	KRS2, MST1, YSK3	Q13043	ENSG0000(	Kinase	Z93016,AL109839	Primary immunodeficiency, Combined immunodeficiency due to STK4 deficiency, Schizophrenia, Recurrent viral infection, Recurrent bacterial infection, Recurrent fungal infection, Lymphopenia, Warts, Immunologic deficiency syndrome, Chronic mucocutaneous candidosis	https://www.targetvalidation.org/target/ENSG00000101109/associations	AKT3<br>TAOK2<br>RASSF1<br>SAV1<br>TAOK3<br>RASSF5<br>RASSF3<br>TAOK1<br>PHLPP2<br>PPP2CA<br>PPP2CB<br>PPP1CB<br>PPP1CA<br>MAPK8<br>CASP3<br>PPP1CC<br>AKT2<br>AKT1<br>MST1<br>RAF1<br>ABL1<br>LATS1<br>PHLPP1<br>THAP12<br>CHEK1<br>TNNI3<br>MDFI<br>FOXO1<br>LATS1	Cytoplasm, Nucleus,		Yes	https://www.bindingdb.org/uniprot/Q13043	✓
STKLD1	HUMAN Serine/threonine kinase-like domain-containing protein STKLD1	C9orf96, SGK071	Q8NE28	ENSG0000(	Kinase,Cancer,Cancer-Antigen	BC036504	Melanoma, Breast carcinoma	https://www.targetvalidation.org/target/ENSG00000198870/associations				No		✓
STMN1	HUMAN Stathmin	C1orf215, FLJ32206, Lag, LAP18, OP18, PP17, PP19, PR22, SMN	P16949	ENSG0000(	Signalling, Cancer	Z11566	Lung carcinoma, Neurodegenerative disease, Glioma, Prostatic neoplasm, Depressive disorder, Spinal muscular atrophy, Leber plus disease, Leigh syndrome, Epilepsy, Metachromatic leukodystrophy	https://www.targetvalidation.org/target/ENSG00000117632/associations	CAMK2A<br>NLK<br>CDK12<br>CDK19<br>AURKB<br>MAPK15<br>UHMK1<br>CDK20<br>MAPK6<br>CAMK4<br>CDK10<br>CDK13<br>CAMK2B<br>ATR<br>MAPK7<br>PAK1<br>CDK18<br>CDK17<br>CDK16<br>CDK5<br>CDK6<br>CDK3<br>CSNK2A1<br>MAPK10<br>PRKX<br>CDK9<br>CDK7<br>CDK8<br>MAPK9	Cytoplasm, cytoskeleton,		No		✓
STRADA	HUMAN STE20-related kinase adapter protein alpha	LYK5, NY-BR-96, STRAD	Q7RTN6	ENSG0000(	Kinase	AK075005	Alzheimer's disease, Peutz-Jeghers syndrome, Epilepsy, Congenital abnormality, Adenocarcinoma, Myeloproliferative disease, Medulloblastoma, Colorectal cancer, Melanoma, Tuberous sclerosis	https://www.targetvalidation.org/target/ENSG00000266173/associations	CAB39<br>STK11<br>SMAD4<br>STK11<br>CAB39	Nucleus		No		✓
STRADB	HUMAN STE20-related kinase adapter protein beta	ALS2CR2, CALS-21, ILPIP, PRO1038	Q9C0K7	ENSG0000(	Kinase	AK027637	Juvenile amyotrophic lateral sclerosis, Sepsis	https://www.targetvalidation.org/target/ENSG00000082146/associations	TRAF6<br>CAB39<br>STK11<br>MAP3K7<br>MAP3K7<br>STK11<br>TRAF6<br>XIAP<br>CAB39	Nucleus		No		✓

STUB1	HUMAN E3 ubiquitin-protein ligase CHIP	STUB1, CHIP, NY-CO-7	Q9UNE7	ENSG0000(	Signalling	AF217968	Neurodegenerative disease, Autosomal recessive cerebellar ataxia, Spinocerebellar ataxia type 29, Cerebellar ataxia-hypogonadism, Autosomal recessive predisposition, Cerebellar Hypoplasia, Dysarthria, Appendicular ataxia, Truncal ataxia, Spastic ataxia	https://www.targetvalidation.org/target/ENSG00000103266/associations	MAP4K5 CAMK2A TBK1 MAP3K3 MAP3K5 MAP3K14 YME1L1 KIAA1524 NHLRC1 RPS6KA1 PTK2B RUNX2 PRKCZ RUNX1 CDK5 CDK6 UBXN2A EIF5A UBE2D3 BACE1 ATXN1 MAPK10 HSPA7 MAPK8 TGFBR1 AHR HSPA4 NOS1 PRKACA	Cytoplasm	No		✓
STYX	HUMAN Serine/threonine/tyrosine-interacting protein	STYX	Q8WUJ0	ENSG0000(	Others	CH471061	Male infertility with teratozoospermia due to single gene mutation, Non-syndromic male infertility due to sperm motility disorder, Male infertility due to globozoospermia, Male infertility with azoospermia or oligozoospermia due to single gene mutation, Deafness-infertility syndrome, Azoospermia, Oligospermia, Autoimmune Disease, Type I diabetes melitus, Primary ciliary dyskinesia	https://www.targetvalidation.org/target/ENSG00000198252/associations	FBXW7 CARHSP1	Nucleus	No		✓
STYXL1	HUMAN Serine/threonine/tyrosine-interacting-like protein 1	DUSP24, MK-STYX	Q9Y6J8	ENSG0000(	Others	AF188201	Small cell lung carcinoma, Melanoma, Epilepsy, Mental retardation, Prostate adenocarcinoma, Precursor B-cell lymphoblastic leukemia, Mucocutaneous lymph node syndrome, Glioblastoma multiforme	https://www.targetvalidation.org/target/ENSG00000127952/associations	TGFBR1		No		✓
SUB1	HUMAN Activated RNA polymerase II transcriptional coactivator p15	PC4, RPO2TC1	P53999	ENSG0000(	Transcription factor	BC010537	Prostate carcinoma, Teratozoospermia, Acute myelocytic leukemia, Liver carcinoma, Multiple myeloma, Melanoma, Glioma, Acute leukemia, Gastric carcinoma, Non-small cell lung carcinoma	https://www.targetvalidation.org/target/ENSG00000113387/associations	CSNK2A1 CLK2 HNF4A CSTF2 TAF1 CSNK2A2 STK10 SUPT5H EP300 POU2AF1	Nucleus,	No		✓
SUCLA2	HUMAN Succinate--CoA ligase [ADP-forming] subunit beta, mitochondrial	SUCB1	Q9P2R7	ENSG0000(	Cancer,Cancer-Antigen	AK315513	Mitochondrial DNA depletion syndrome, Mitochondrial DNA depletion syndrome (encephalomyopathic form), Mitochondrial DNA depletion syndrome (encephalomyopathic form with methylmalonic aciduria), Colorectal cancer, Hereditary spastic paraplegia, Retinoblastoma, Multiple myeloma, Malonic aciduria, Leukopenia, Deafness	https://www.targetvalidation.org/target/ENSG00000136143/associations	ALAS2	Mitochondrion	Yes	https://www.drugbank.ca/biodb/polypeptides/Q9P2R7	✓
SUFU	HUMAN Suppressor of fused homolog	PRO1280, SUFUH, SUFUXL	Q9UMX1	ENSG0000(	Transcription factor,Cancer	AF175770	Medulloblastoma, Esophageal carcinoma, Basal cell carcinoma, Basal cell nevus syndrome, Desmoplastic medulloblastoma, Keratocystic odontogenic tumor, Coarse facial feature, Gorlin syndrome, Isolated Kippel-Feil syndrome, Neural tube defect	https://www.targetvalidation.org/target/ENSG00000107882/associations	BTRC FBXW11 STK36 WNK1 SMO MAP3K19 GSK3B PRKACA GLI3 GLI2 GLI1 RPS6KA4 MTSS1 SAP18 GLI2 GLI3 SMO GLI1 SAP18 GLIS3 STK36 GLIS2	Cytoplasm	No		✓
SUMO2	HUMAN Small ubiquitin-related modifier 2	SMT3B, SMT3H2	P61956	ENSG0000(	Signalling	AC022211	Ductal adenocarcinoma, Hepatocellular carcinoma, Melanoma, Gastric carcinoma, Endometrial neoplasm, Bloom syndrome, Promyelocytic leukemia, Prostate carcinoma, Breast carcinoma, Spinocerebellar Ataxia Type 7, Cardiomyopathy, Degenerative polyarthritis, Chronic Hepatitis B	https://www.targetvalidation.org/target/ENSG00000188612/associations	LEF1 RANGAP1 GLI2 KALRN KDM1A RNF168 JUN SENP3 TRIM63 FOS ELF4 RCOR1 UIMC1 PML ATF7IP RARA RNF4 SOX6 SENP6 CENPC	Nucleus, Nucleus, PML body,	No		✓
SUPT20H	HUMAN Transcription factor SPT20 homolog	C13orf19, FAM48A, P38IP, SPT20, FP757	Q8NEM7	ENSG0000(	Transcription factor,Cancer,Cancer-Antigen	AL138706, AL391383	Melanoma, Teratozoospermia, Breast carcinoma, Juvenile dermatomyositis, Colorectal adenocarcinoma, Clear cell renal carcinoma, Head and Neck squamous cell carcinoma	https://www.targetvalidation.org/target/ENSG00000102710/associations	MAPK14 ATG9A	Nucleus	No		✓
SUPT4H1	HUMAN Transcription elongation factor SPT4	Spt4, SPT4H, SUPT4H	P63272	ENSG0000(	Transcription factor	AK129758	HIV infection, Breast carcinoma, Huntington disease, Spastic syndrome, Lung adenocarcinoma, Colorectal cancer, Colorectal adenocarcinoma, Small cell lung carcinoma	https://www.targetvalidation.org/target/ENSG00000213246/associations	SUPT5H	Nucleus	No		✓

SYCE1	HUMAN Synaptonemal complex central element protein 1	bA108K14.6, C10orf94, CT76	Q8N0S2	ENSG0000(	Others	AK314978	Primary ovarian insufficiency, Male infertility with azoospermia or oligozoospermia due to single gene mutation, Normosmic congenital hypogonadotropic hypogonadism, Kallmann syndrome, Azoospermia, Turner syndrome, Premature ovarian failure, Trichohepatoenteric Syndrome, Young syndrome, Deafness-infertility syndrome	https://www.targetvalidation.org/target/ENSG00000171772/associations		Nucleus		No		✓
SYCP1	HUMAN Synaptonemal complex protein 1	CT8, HOM-TES-14, SCP1	Q15431	ENSG0000(	Others	AL358372, AL645502	Male infertility with teratozoospermia due to single gene mutation, Male infertility with azoospermia or oligozoospermia due to single gene mutation, Primary ovarian insufficiency, Teratozoospermia, Kallmann syndrome, Adenocarcinoma, Pancreatic neoplasm, Xeroderma pigmentosum, Synovial sarcoma, Liver and intrahepatic biliary tract carcinoma	https://www.targetvalidation.org/target/ENSG00000198765/associations		Nucleus		No		✓
SYK	HUMAN Tyrosine-protein kinase SYK	KSYK	P43405	ENSG0000(	Kinase,Cancer	BC001645	Autoimmune disease, Rheumatoid arthritis, Melanoma, Mesothelioma, Anti-Glomerular basement membrane disease, Lymphoma, Peripheral T-Cell lymphoma, Breast carcinoma, B-Cell lymphoma, Bile duct carcinoma	https://www.targetvalidation.org/target/ENSG00000165025/associations	TRAF6 GAB2 DBNL CLEC1B CLEC7A MYD88 MAP4K1 BLNK NFAM1 RHOH RPS6KA1 ERBB4 PRKD1 PTK2B SLA CBLB PRKAA1 BTK PTK2 MS4A2 SH3BP2 GRB2 DUSP3 CRKL STAT3 CD79B CEACAM3 FLT3 FCGR2C FCER1G PTPN6	Cell membrane		Yes	https://www.bindingdb.org/uniprot/P43405	✓
TACC1	HUMAN Transforming acidic coiled-coil-containing protein 1	KIAA1103	O75410	ENSG0000(	Others	AK314620	Breast carcinoma, Colorectal adenocarcinoma, Gliosarcoma, Giant cell glioblastoma, Gastric neoplasm, Glioblastoma multiforme, Malignant mesothelioma, Gastrointestinal stromal tumor, Dermatomyositis, Prostate carcinoma	https://www.targetvalidation.org/target/ENSG00000147526/associations	AURKC AURKB AURKA	Cytoplasm		No		✓
TACSTD2	HUMAN Tumor-associated calcium signal transducer 2	GA733-1, M1S1, TROP2	P09758	ENSG0000(	Others	X77754	Corneal dystrophy, Gelatinous drop-like corneal dystrophy, Lung carcinoma, Cystic fibrosis, Photophobia, Endometriosis, Blurred vision, Childhood onset, Photodysphoria, Autosomal recessive predisposition	https://www.targetvalidation.org/target/ENSG00000184292/associations	PRKCA	Membrane, Single-pass type I membrane protein,	Transmembrane	No		✓
TADA2A	HUMAN Transcriptional adapter 2-alpha	TADA2L, KL04P	O75478	ENSG0000(	Transcription factor	AC068400	Azoospermia, Acute lymphoblastic leukemia, VACTERL/VATER association, Premature infant, Type II diabetes mellitus, Obesity, Vasculitis	https://www.targetvalidation.org/target/ENSG00000276234/associations		Nucleus, Chromosome,		No		✓
TADA3	HUMAN Transcriptional adapter 3	ADA3, hADA3, NGG1, TADA3L	O75528	ENSG0000(	Transcription factor	CR533543	Breast carcinoma, Malignant peripheral nerve sheath tumor, Cervical carcinoma, Medulloblastoma, Acute monocytic leukemia, HIV infection	https://www.targetvalidation.org/target/ENSG00000171148/associations	ANKRD12 TP53 ESR1	Nucleus		No		✓
TAF5L	HUMAN TAF5-like RNA polymerase II p300/CBP-associated factor-associated factor 65 kDa subunit 5L	PAF65B	O75529	ENSG0000(	Others	CH471098	Breast carcinoma, Diabetes mellitus, Type I diabetes mellitus, Breast adenocarcinoma, Melanoma, Central nervous system cancer, Pancreatic carcinoma, Diffuse large B-cell lymphoma, Chronic lymphocytic leukemia, Colorectal adenocarcinoma	https://www.targetvalidation.org/target/ENSG00000135801/associations		Nucleus		No		✓
TAF6L	HUMAN TAF6-like RNA polymerase II p300/CBP-associated factor-associated factor 65 kDa subunit 6L	PAF65A	Q9Y6J9	ENSG0000(	Transcription factor	NA	Breast carcinoma, Melanoma	https://www.targetvalidation.org/target/ENSG00000162227/associations		Nucleus		No		✓

TAF7L	HUMAN Transcription initiation factor TFIID subunit 7-like	CT40, TAF2Q	Q5H9L4	ENSG0000(	Transcription factor	Z68331,AL109801G	HIV infection, Li-Fraumeni syndrome, Male infertility with azoospermia or oligozoospermia due to single gene mutation, Kallmann syndrome, Normosmic congenital hypogonadotropic hypogonadism, Male infertility, Primary ciliary dyskinesia, Myelodysplastic syndrome, Colorectal adenocarcinoma, Breast carcinoma	https://www.targetvalidation.org/target/ENSG00000102387/associations		Nucleus	No		✓
TAF9	HUMAN Transcription initiation factor TFIID subunit 9	TAF2G, TAFII31	Q16594	ENSG0000(	Transcription factor	BT019652	HIV infection, neoplasm, cancer,sarcoma, multiple myeloma, non-small cell lung carcinoma, non-small cell lung adenocarcinoma, acute quadriplegic myopathy	https://www.targetvalidation.org/target/ENSG00000273841/associations	KAT2A TAF12 GTF2B TAF1 TBP TAF1 TAF10 TAF4 TAF6 TAF13 TAF11 TAF5	Nucleus	No		✓
TAGLN	HUMAN Transgelin	DKFZp686P11128, SM22, WS3-10	Q01995	ENSG0000(	Cancer	D17409	Lung carcinoma, Lung adenocarcinoma, Colonic neoplasm, Endometriosis, Keloid, Lipoidosis, Asthma, Adenocarcinoma, Carotid stenosis, Esophagus squamous cell carcinoma	https://www.targetvalidation.org/target/ENSG00000149591/associations	PRKG2 PRKCD PRKCE PRKCA IGF1R PRKCB ISG15 PRKCG PIK3CD TRAF6 RNF14	Cytoplasm	No		✓
TAOK3	HUMAN Serine/threonine-protein kinase TAO3	DPK, JIK, MAP3K18	Q9H2K8	ENSG0000(	Kinase	AF181985	Chronic obstructive pulmonary disease, Obesity, Postoperative pain, Prostate carcinoma, Breast carcinoma, Congenital hypothyroidism, Metastatic prostate cancer, Erectile dysfunction, Ischemia, Seizures	https://www.targetvalidation.org/target/ENSG00000135090/associations	SLK ATM ERN1 STK4 TRAF2 STK3	Cytoplasm	Yes	https://www.bindingdb.org/uniprot/Q9H2K8	✓
TARDBP	HUMAN TAR DNA-binding protein 43	ALS10, TDP-43	Q13148	ENSG0000(	Transcription factor	EF434183	Amyotrophic lateral sclerosis, Brain disease, Familial amyotrophic lateral sclerosis, Muscle degeneration, Respiratory failure, Nerve degeneration, Muscular dystrophy, Kennedy disease, Cystic fibrosis, Alexander disease	https://www.targetvalidation.org/target/ENSG00000120948/associations	TIAL1 CSNK2A1 VCP IRAK1 CSNK1A1 FUS HSPA4 UBC HDAC6 SQSTM1 UBQLN1 UBQLN2	Nucleus	No		✓
TBC1D2	HUMAN TBC1 domain family member 2A	Armus, PARIS1, TBC1D2A, TBD2A	Q9BYX2	ENSG0000(	Others	AK026105	Prostate carcinoma, Breast carcinoma, Post-traumatic stress disorder, Cleft lip, Cleft palate, Breast carcinoma, Glioblastoma multiforme, Ovarian carcinoma, Meningococcal infection, Adams-Oliver syndrome	https://www.targetvalidation.org/target/ENSG00000095383/associations		Cytoplasm	No		✓
TBC1D5	HUMAN TBC1 domain family member 5	KIAA0210	Q92609	ENSG0000(	Others	AC090960	Schizophrenia, Tuberculosis, Adenocarcinoma, Rheumatoid arthritis, Tuberculosis, Gliblastoma multiforme, Gaucher disease type 1, Osteosarcoma, Emphysema, Melanoma	https://www.targetvalidation.org/target/ENSG00000131374/associations	VPS29	Endosome membrane	No		✓
TBCB	HUMAN Tubulin-folding cofactor B	CG22, CKAP1, CKAPI	Q99426	ENSG0000(	Cancer,Cancer-Antigen	AD001527	Ovarian carcinoma, Pancreatic carcinoma, Multiple myeloma, Rheumatoid arthritis, Breast carcinoma, Pachygyria, Schizophrenia, Prostate carcinoma, Giant axonal neuropathy, Macroglobulinemia	https://www.targetvalidation.org/target/ENSG00000105254/associations	PAK1 PRKCE GAN	Cytoplasm	No		✓
TBK1	HUMAN Serine/threonine-protein kinase TBK1	NAK	Q9UHD2	ENSG0000(	Kinase	AK291039	Neurodegenerative disease, Frontotemporal dementia with motor neuron disease, Glaucoma, Open-angle glaucoma, Amyotrophic Lateral Sclerosis, Herpes encephalitis, Apathy, Skeletal muscle atrophy, Paralysed, Low tension glaucoma	https://www.targetvalidation.org/target/ENSG00000183735/associations	IKBKG TRAF6 DTX4 CYLD AZI2 ZBP1 IFIH1 ELF4 MAP3K14 EXOC2 OPTN DHX58 EXOC4 ARHGEF2 TANK DYRK2 TICAM1 MIB1 TMEM173 MAVS CDC37 CASP8 IRF3 IKBKE TRIM14 CALCOCO2 TRAF3 XIAP YWHAE TXLNA AKT1	Cytoplasm	Yes	https://www.bindingdb.org/uniprot/Q9UHD2	✓

TBL1X	HUMAN F-box-like/WD repeat-containing protein TBL1X	EBI, TBL1	O60907	ENSG0000(	Signalling	AC003036	Breast carcinoma, Tongue squamous cell carcinoma, Adenocarcinoma, Pancreatic ductal adenocarcinoma, Autism spectrum disorder, Steatohepatitis, Colorectal cancer, Breast adenocarcinoma, Metabolic syndrome X, Rheumatoid arthritis	https://www.targetvalidation.org/target/ENSG00000101849/associations	CTNNB1 GPS2	Nucleus	No	✓
TBPL1	HUMAN TATA box-binding protein-like protein 1	STUD, TLF, TLP, TRF2	P62380	ENSG0000(	Transcription factor	AK292320	Teratozoospermia, Male infertility with azoospermia or oligozoospermia due to single gene mutation, Male infertility with teratozoospermia due to single gene mutation, Kallmann syndrome, Normosmic congenital hypogonadotropic hypogonadism, Breast carcinoma, Colon carcinoma, Gastric carcinoma, Glioma, Hereditary hemochromatosis	https://www.targetvalidation.org/target/ENSG00000028839/associations	IRAK4 GTF2A1 NF1 GTF2B	Cytoplasm	No	✓
TBX5	HUMAN T-box transcription factor TBX5	HOS	Q99593	ENSG0000(	Transcription factor	AF221714	Heart-hand syndrome, Holt-Oram syndrome, Ventricular septal defect, Atrial septal defect, Paroxysmal atrial fibrillation, Triphalangeal thumb, Hypoplastic left heart syndrome, Radial aplasia, Schizophrenia, Adenocarcinoma	https://www.targetvalidation.org/target/ENSG00000089225/associations	NKX2-5	Nucleus	No	✓
TBX6	HUMAN T-box transcription factor TBX6	TBX6	O95947	ENSG0000(	Transcription factor	AC093512	Autosomal dominant spondylocostal dysostosis, Autism, Colorectal adenocarcinoma, Scoliosis, Congenital hemivertebra, Spinal fusion, Anteverted nostril, Posterior rib fusion, Lung adenocarcinoma, Li-Fraumeni syndrome	https://www.targetvalidation.org/target/ENSG00000149922/associations		Nucleus	No	✓
TCEA2	HUMAN Transcription elongation factor A protein 2	TFIIS	Q15560	ENSG0000(	Transcription factor	AL590548	Renal cell carcinoma, Acute promyelocytic leukemia, Colorectal adenocarcinoma, Pancreatic ductal adenocarcinoma, Breast carcinoma, Crohn's disease, Psoriasis, Hepatocellular carcinoma, Melanoma, Meningococcal infection	https://www.targetvalidation.org/target/ENSG00000171703/associations	CAMKV ERCC8 GTF2B MARK3 CDK11B AKT1S1 GRIPAP1	Nucleus,	No	✓
TCEAL1	HUMAN Transcription elongation factor A protein-like 1	SIIR, TCAL1	Q15170	ENSG0000(	Transcription factor	BC000809	Gastric carcinoma, Breast carcinoma, Breast adenocarcinoma, Sarcoma, Colorectal cancer, Cervix carcinoma, Bladder neoplasm, Chronic obstructive pulmonary disease, Lyme disease, Adenomatous Polyposis coli	https://www.targetvalidation.org/target/ENSG00000172465/associations	GSK3B GSK3A CDK1	Nucleus,	No	✓
TCF12	HUMAN Transcription factor 12	bHLHb20, HEB, HTF4	Q99081	ENSG0000(	Transcription factor,Cancer	AK304007	Craniosynostosis, Isolated plagiocephaly, Isolated brachycephaly, Prostate adenocarcinoma, Glioma, Bile duct carcinoma, Brain stem glioblastoma, Astrocytoma, Pancreatic ductal adenocarcinoma, Colorectal adenocarcinoma	https://www.targetvalidation.org/target/ENSG00000140262/associations	PTF1A RUNX1T1 ID3 ID2 ID1 TCF21 TCF3 EP300 CREBBP	Nucleus,	No	✓
TCF21	HUMAN Transcription factor 21	bHLHa23, POD1	O43680	ENSG0000(	Transcription factor	CR450293	Stroke, Coronary heart disease, Vascular disease, Coronary artery disease, Adrenocortical carcinoma, Atherosclerosis, Arteriosclerosis, Head and Neck squamous cell carcinoma, Ovarian carcinoma, Jeune syndrome	https://www.targetvalidation.org/target/ENSG00000118526/associations	HDAC1 TCF4 TCF12	Nucleus,	No	✓

TCF4	HUMAN Transcription factor 4	bHLHb19, ITF2, SEF2-1B	P15884	ENSG0000(	Transcription factor,Signalling	FR748210	Pitt-Hopkins syndrome, Corneal dystrophy, Fuchs endothelial corneal dystrophy, Mental retardation, Schizophrenia, Bipolar disorder, Primary sclerosing cholangitis, Nonorganic psychosis, Peripheral neuropathy, Seizures	https://www.targetvalidation.org/target/ENSG00000196628/associations	NLK PIAS4 CTBP1 EP300 AES ID3 ID2 CSNK2A1 ID1 CTNNB1 JUP JUN HBP1 TCF21 BCL9 ID2 CTBP1 EP300 HIPK2 AES AR BCL9 TLE1 JUP FERMT2	Nucleus		No		✓
TCL1A	HUMAN T-cell leukemia/lymphoma protein 1A	TCL1	P56279	ENSG0000(	Cancer	BC005831	Lymphoid neoplasm, Melanoma, Cutaneous melanoma, Colorectal adenocarcinoma, Leukemia, Multiple myeloma, Squamous cell lung carcinoma, Prostate adenocarcinoma, Oral cavity cancer, Gastric adenocarcinoma	https://www.targetvalidation.org/target/ENSG00000100721/associations	AKT3 AES AKT2 AKT1 FOS EP300 AKT1 JUN ATM AKT2 AKT3	Cytoplasm		No		✓
TDP2	HUMAN Tyrosyl-DNA phosphodiesterase 2	TTRAP, EAP2, AD-022	O95551	ENSG0000(	Transcription factor	AK002168	Autosomal recessive cerebellar ataxia-epilepsy-intellectual disability syndrome due to TUD deficiency, Colorectal cancer, Narcolepsy, Dyslexia, Osteosarcoma, Lung carcinoma, neurodegenerative disease, Endometrial neoplasm, Ulcerative colitis	https://www.targetvalidation.org/target/ENSG00000111802/associations	TRAF6 ACVR1B CD40	Nucleus, PML body, nucleolus, Cytoplasm,		Yes	https://www.bindingdb.org/uniprot/O95551	✓
TDRD1	HUMAN Tudor domain-containing protein 1	CT41.1	Q9BXT4	ENSG0000(	Others	CH471066	Li-Fraumeni syndrome, Colorectal adenocarcinoma, Prostate carcinoma, Male infertility, Seminoma, Testicular germ cell tumor, Benign prostatic hyperplasia, Sertoli cell-only syndrome, Hepatocellular carcinoma, Autism	https://www.targetvalidation.org/target/ENSG00000095627/asso		Cytoplasm		No		✓
TDRD3	HUMAN Tudor domain-containing protein 3	TDRD3	Q9H7E2	ENSG0000(	Others	AL354764, AL512666	Atopic eczema, Psoriasis, Fragile X syndrome, Leukemia, Melanoma, Breast adenocarcinoma, Pancreatic carcinoma	https://www.targetvalidation.org/target/ENSG00000083544/associations		Cytoplasm		No		✓
TDRKH	HUMAN Tudor and KH domain-containing protein	TDRD2	Q9Y2W6	ENSG0000(	Others	AL589765	Azoospermia, Breast carcinoma, Malignant mesothelioma, Non-small cell lung carcinoma, Macroglobulinemia, Sarcoma, Multiple myeloma	https://www.targetvalidation.org/target/ENSG00000182134/associations		Cytoplasm		No		✓
TEAD2	HUMAN Transcriptional enhancer factor TEF-4	ETF, TEF-4, TEF4	Q15562	ENSG0000(	Transcription factor	AC010524	Breast carcinoma, Lung carcinoma, Small cell lung carcinoma, Medulloblastoma, Polycystic ovary syndrome, Neurofibromatosis 2, Multiple acyl coenzyme A dehydrogenase deficiency, Wrinkly skin syndrome	https://www.targetvalidation.org/target/ENSG00000074219/associations	WWTR1 YAP1 NCOA1	Nucleus,		No		✓
TEAD3	HUMAN Transcriptional enhancer factor TEF-5	ETFR-1, TEAD5, TEF-5	Q99594	ENSG0000(	Transcription factor	BC091488	Small cell lung carcinoma, Diffuse large B-cell lymphoma, Preeclampsia, Cardiac hypertrophy, CINCA syndrome, Eclampsia, Heart disease	https://www.targetvalidation.org/target/ENSG00000007866/associations	WWTR1 YAP1	Nucleus,		No		✓
TEK	HUMAN Angiopoietin-1 receptor	CD202b, TIE-2, TIE2, VMCM, VMCM1	Q02763	ENSG0000(	Kinase,Cancer	AK295043	Mucocutaneous venous malformation, Lung carcinoma, Hemangiosarcoma, Hydrophthalmos, Blue rubber bleb nevus syndrome, Intestinal volvulus, Cavernous hemangioma, Colonic neoplasm, Thyroid carcinoma, Colorectal cancer	https://www.targetvalidation.org/target/ENSG00000120156/associations	IKBKG ANGPT4 ANGPT1 TXK TIE1 SHC1 PTPRB FYN LCK ANGPTL1 ANGPT2 FYN STAT5A SHC1 GRB14 SOCS1 GRB7 PTPN11 PIK3R1 GRB2 STAT5B PTPRB ANGPT2 TNIP2 GRAP ANGPT1 ANGPT4 DOK4 TIE1 ANGPTL1 DOK2	Cell membrane	Transmembrane	Yes	https://www.bindingdb.org/uniprot/Q02763	✓

TERT	HUMAN Telomerase reverse transcriptase	EST2, hEST2, TCS1, TP2, TRT	O14746	ENSG0000(	Cancer	AB085628	Aplastic anemia, Dyskeratosis congenita, Melanoma, Idiopathic pulmonary fibrosis, Hoyeraal-Hreidarsson syndrome, Liver carcinoma, Pulmonary hypertension, Glioma, Astrocytoma, Primary immunodeficiency	https://www.targetvalidation.org/target/ENSG00000164362/associations	RUVBL1 RUVBL2 POT1 TEP1 ACD PTGES3 PDK1 PTPN11 SMARCA4 MTOR AKT1 RPS6KB1 PRKCA XRCC6 SRC HSP90AA1 LCK MYC ABL1 MEN1 XRCC5 XRCC6 HSP90AA1 AKT1 RELA NCL UBE2D3 PML MTOR PTGES3 PINX1 DCAF1	Nucleus, nucleolus, nucleoplasm, Chromosome, telomere, Cytoplasm, PML body,	Yes	https://www.bindingdb.org/uniprot/O14746	✓
TEX101	HUMAN Testis-expressed protein 101	SGRG, TX101	Q9BY14	ENSG0000(	Others	AY359077	Male infertility with teratozoospermia due to single gene mutation, Azoospermia, Psoriasis, Male infertility with azoospermia or oligozoospermia due to single gene mutation, Non-syndromic male infertility due to sperm motility disorder, Adenocarcinoma, Breast carcinoma, Ductal carcinoma	https://www.targetvalidation.org/target/ENSG00000131126/associations		Cell membrane	No		✓
TEX14	HUMAN Inactive serine/threonine-protein kinase TEX14	CT113	Q8IWB6	ENSG0000(	Kinase	AF285601	Azoospermia, Normosmic congenital hypogonadotropic hypogonadism, Deafness-infertility syndrome, Non-syndromic male infertility due to sperm motility disorder, Male infertility with azoospermia or oligozoospermia due to single gene mutation, Breast neoplasm, TesticulargGerm cell tumor	https://www.targetvalidation.org/target/ENSG00000121101/associations	PLK1 CDK1	Cytoplasm	No		✓
TFAP4	HUMAN Transcription factor AP-4	AP-4, bHLHc41	Q01664	ENSG0000(	Transcription factor	X57435	Breast carcinoma, Familial hemophagocytic lymphohistiocytosis, Proteasome disability syndrome, Colorectal carcinoma, Liver carcinoma, LCAT deficiency, Mkajo-Nishimura syndrome, CANDLE syndrome, JMP syndrome	https://www.targetvalidation.org/target/ENSG00000090447/associations	SP1 HDAC1 NFIA	Nucleus,	No		✓
TFCP2	HUMAN Alpha-globin transcription factor CP2	CP2, LBP-1C, LSF, TFCP2C	Q12800	ENSG0000(	Transcription factor	AK291264	Hepatocellular carcinoma, Breast carcinoma, Melanoma, Unipolar depression, Alzheimer's disease	https://www.targetvalidation.org/target/ENSG00000135457/associations	CDK3 PPP2CA MAPK1 MAPK3 YY1 CDK2 APBB1 RNF2 NFE4	Nucleus	No		✓
TFCP2L1	HUMAN Transcription factor CP2-like protein 1	CRTR1, LBP-9	Q9NZI6	ENSG0000(	Transcription factor	BC064698	Clear cell renal carcinoma, Bartter syndrome, Nephronophthisis, Ataxia telangiectasia, Renal Cell carcinoma	https://www.targetvalidation.org/target/ENSG00000115112/associations	UBP1	Nucleus	No		✓
TFDP1	HUMAN Transcription factor Dp-1	Dp-1, DP1, DRTF1	Q14186	ENSG0000(	Transcription factor,Signalling,Cancer	CH471085	Lung carcinoma, Large cell lung carcinoma, Pancreatic carcinoma, Breast carcinoma, Juvenile arthritis, Non-Hodgkin's lymphoma, Liver carcinoma	https://www.targetvalidation.org/target/ENSG00000198176/associations	CREBBP RYBP E2F4 E2F5 E2F2 SMAD4 RBL2 E2F1 CDK6 CDK3 RBL1 CDK2 RB1 E2F6 CHEK1 SOCS3 E2F3 E2F1 MAX E2F5 RB1 RBL1 E2F2	Nucleus	No		✓
TFDP2	HUMAN Transcription factor Dp-2	DP2	Q14188	ENSG0000(	Transcription factor,Cancer	AK303181	Malignant mesothelioma, Chronic kidney failure, Asthma, Liver carcinoma, Colorectal cancer, Cervix carcinoma, Retinoblastoma, Testicular germ cell tumor	https://www.targetvalidation.org/target/ENSG00000114126/associations	E2F4 E2F2 E2F1 YWHAE RBL1 RB1 E2F6 E2F3 E2F1 RB1 GMCL1 RBL1 E2F2	Nucleus,	No		✓
TFDP3	HUMAN Transcription factor Dp family member 3	CT30, E2F-like, HCA661	Q5H9I0	ENSG0000(	Transcription factor	NA	Li-Fraumeni syndrome, Colorectal adenocarcinoma, Liver neoplasm, Colitis, Asthma, Prostate carcinoma	https://www.targetvalidation.org/target/ENSG00000183434/associations	SOCS3	Nucleus, Cytoplasm,	No		✓
TFE3	HUMAN Transcription factor E3	bHLHe33, TFEA	P19532	ENSG0000(	Transcription factor,Cancer	AF196779	Adenocarcinoma, Renal carcinoma, Alveolar soft part sarcoma, Epithelioid hemangioendothelioma, Perivascular epithelioid cell neoplasm	https://www.targetvalidation.org/target/ENSG00000068323/associations	SMAD4 SMAD3 MITF TFEC E2F3	Nucleus,	No		✓
TFEB	HUMAN Transcription factor EB	bHLHe35, TCFEB	P19484	ENSG0000(	Transcription factor,Cancer	BC006225	Adenocarcinoma, Lung carcinoma, Kidney neoplasm, Microphthalmos, Renal carcinoma, Glioblastoma	https://www.targetvalidation.org/target/ENSG00000112561/associations	MTOR MAPK1 MAP1LC3B SQSTM1 UVRAG	Cytoplasm	No		✓

TFEC	HUMAN Transcription factor EC	BHLHE34, TCFEC, TFECL	O14948	ENSG0000	Transcription factor	CH236947	Li-Fraumeni syndrome, Griscelli disease, Familial hyperinsulinism, Multiple sclerosis, Microphthalmos, Hereditary hyperkplexia	https://www.targetvalidation.org/target/ENSG00000105967/associations	MITF TFE3	Nucleus		No		✓
TFG	HUMAN Protein TFG	FLJ36137, SPG57, TF6	Q92734	ENSG0000	Cancer	AK093456	Hereditary spastic paraplegia, Charcot-Marie-Tooth disease, Hereditary motor and sensory neuropathy, Complex hereditary spastic paraplegia, Papillary thyroid carcinoma, Neuropathy, Colonic neoplasm, Melanoma	https://www.targetvalidation.org/target/ENSG00000114354/associations	MAP3K3 TSSK2 TRIM68 TRAF3 PTPN6 SRC IKBKG SEC16A SEC16B	Endoplasmic reticulum		No		✓
TFPT	HUMAN TCF3 fusion partner	amida, FB1, INO80F	P0C1Z6	ENSG0000	Cancer	CH471135	Squamous cell carcinoma, Colorectal adenocarcinoma, Esophageal squamous cell carcinoma, Rectal adenocarcinoma, Retinal disease	https://www.targetvalidation.org/target/ENSG00000105619/associations	CDK1 NOL3 TCF3 PAWR	Nucleus		No		✓
TFRC	HUMAN Transferrin receptor protein 1	CD71, p90, TFR1	P02786	ENSG0000	Cancer	AB209254	Primary immunodeficiency, Adenocarcinoma, Iron deficiency anemia, Beta Thalassemia, Glioma, Obesity, Non-Hodgkin's lymphoma	https://www.targetvalidation.org/target/ENSG00000072274/associations	SH3BP4 IKBKE PRKCE MAPK1 PRKCA SRC PRKCB PRKCG TF CD4 GABARAP TF CD247 RAB5B	Cell membrane	Transmembrane	Yes	https://www.drugbank.ca/biodb/polypeptides/P02786	✓
TGFBR2	HUMAN TGF-beta receptor type-2	MFS2	P37173	ENSG0000	Kinase,Cancer	U69152	Rare disease with thoracic aortic aneurysm and aortic dissection, Loeys-Dietz syndrome, Aortic Aneurysm, Migraine disorder, Esophageal carcinoma, Fatigue, Marfan syndrome, Squamous cell carcinoma	https://www.targetvalidation.org/target/ENSG00000163513/associations	STRAP SNX6 DAXX SMURF1 SMURF2 TSSK1B SMAD2 EIF3I PAK1 TGFBR3 DAB2 SMAD3 AP2B1 GRB2 PPP1CA TGFB2 ACVRL1 TGFBR1 CTNNB1 PML PIK3R1 ENG SRC TGFB3 HSP90AA1 CDK1 APP TGFB1 ZFYVE9 MAP3K7 SMAD7	Cell membrane	Transmembrane	Yes	https://www.bindingdb.org/uniprot/P37173	✓
TGIF1	HUMAN Homeobox protein TGIF1	HPE4, TGIF	Q15583	ENSG0000	Transcription factor,Cancer	AK291112	Holoprosencephaly, Hypotrichosis simplex, Primary ciliary dyskinesia, L1 syndrome, Glioma	https://www.targetvalidation.org/target/ENSG00000177426/associations	SIN3A SMAD2 HDAC1 CTBP1 MAPK1 MAPK3 AR JUN TRIM24 SMAD2 WWP1	Nucleus		No		✓
TGIF2	HUMAN Homeobox protein TGIF2	TGIF2	Q9GZN2	ENSG0000	Transcription factor	AK313919	Familial isolated hypoparathyroidism, Familial hypocalciuric hypercalcemia, Autosomal dominant hypocalcemia, Rectal carcinoma, Medulloblastoma, Holoprosencephaly, Non-small cell lung carcinoma	https://www.targetvalidation.org/target/ENSG00000118707/associations		Nucleus,		No		✓
THEG	HUMAN Testicular haploid expressed gene protein	CT56, THEG1	Q9P2T0	ENSG0000	Others	CH471242	Male infertility with teratozoospermia due to single gene mutation, Kallmann syndrome, Male infertility with azoospermia or oligozoospermia due to single gene mutation, Normosmic congenital hypogonadotropic hypogonadism, Central nervous system cancer, Gonadoblastoma, Breast carcinoma, Gastric carcinoma, Central neuroblastoma, Testicular infection	https://www.targetvalidation.org/target/ENSG00000105549/associations	CCT5	Nucleus		No		✓
THRA	HUMAN Thyroid hormone receptor alpha	EAR7, ERBA1, NR1A1, THRA1, THRA2	P10827	ENSG0000	Transcription factor,Cancer	M24899	Congenital hypothyroidism, Xerosis, Dyschezia, Thyroid carcinoma, Euthyroid sick syndrome, Unipolar depression, Distal hereditay motor neuropathy type 2, Nodular goiter, Systolic heart failure	https://www.targetvalidation.org/target/ENSG00000126351/associations	NCOR2 TRIP11 NR0B2 CDK7 RXRB NR2F2 RXRA RARG RARB RARA NCOR1 TRIM24 NCOR2 NRIP1 TRIM63 CREB1 RXRA RARA RARB RARG RXRB NR2F2 GTF2B RXRG ITGB3BP	Nucleus		Yes	https://www.bindingdb.org/uniprot/P10827	✓
THRB	HUMAN Thyroid hormone receptor beta	ERBA-BETA, ERBA2, GRTH, NR1A2, PRTH, THR1	P10828	ENSG0000	Transcription factor,Cancer	AC012087	Generalized resistance to thyroid hormone, Selective pituitary resistance to thyroid hormone, Follicular thyroid carcinoma, Hyperthyroidism, Hypothyroidism, Systemic lupus erythematosus, Unipolar depression, Nodular goiter, Glycogen storage disease due to glycogen branching enzyme deficiency	https://www.targetvalidation.org/target/ENSG00000151090/associations	TRIP11 NR0B2 ACVR1 RXRB MAPK1 MAPK3 RXRA SRC TP53 NCOR1 STAT5A CTCF NCOA3 PPARGC1A PPARG RXRA PIK3R1 RXRB RXRG	Nucleus		Yes	https://www.bindingdb.org/uniprot/P10828	✓

THUMPD1	HUMAN THUMP domain-containing protein 1	FLJ20274, Tan1	Q9NXG2	ENSG0000(	Cancer,Cancer-Antigen	AK000281	Glioblastoma multiforme, Atopic eczema, Breast carcinoma, Lung carcinoma, Endometrial carcinoma, Uterine corpus cancer, Colorectal cancer, Melanoma	https://www.targetvalidation.org/target/ENSG00000066654/associations	NAT10			No		✓
TIE1_ext	HUMAN Tyrosine-protein kinase receptor Tie-1	TIE	P35590	ENSG0000(	Kinase,Cancer	AC093420	Von Willebrand disease, P2Y12 defect, Hereditary angioedema, Gastrointestinal stromal tumor, Neurilemmoma, Fibroid tumor, Hepatopulmonary syndrome, Liver cirrhosis, Melanoma, Hemangiosarcoma	https://www.targetvalidation.org/target/ENSG00000066056/associations	ANGPT1 TEK PTPN11 PIK3R1 TEK	Cell membrane	Transmembrane	Yes	https://www.bindingdb.org/uniprot/P35590	✓
TIE1_int	HUMAN Tyrosine-protein kinase receptor Tie-1	TIE	P35590	ENSG0000(	Kinase,Cancer	AC093420	Von Willebrand disease, P2Y12 defect, Hereditary angioedema, Gastrointestinal stromal tumor, Neurilemmoma, Fibroid tumor, Hepatopulmonary syndrome, Liver cirrhosis, Melanoma, Hemangiosarcoma	https://www.targetvalidation.org/target/ENSG00000066056/associations	ANGPT1 TEK PTPN11 PIK3R1 TEK	Cell membrane	Transmembrane	Yes	https://www.bindingdb.org/uniprot/P35590	✓
TIMP3	HUMAN Metalloproteinase inhibitor 3	SFD	P35625	ENSG0000(	Cancer,Cancer-Antigen	U02571	Retinal dystrophy, Sorsby's fundus dystrophy, Asthma, Colitis, Neurotoxicity syndrome, Retinitis pigmentosa, Neoplasm invasiveness, Gastric neoplasm	https://www.targetvalidation.org/target/ENSG00000100234/associations	MMP13 KDR GNRHR MMP9 ADAM17 MMP2 AGTR2 ADAMTS4 EFEMP1 ADAMTS5	Secreted, extracellular space, extracellular matrix,		Yes	https://www.drugbank.ca/biodb/polypeptides/P35625	✓
TIRAP	HUMAN Toll/interleukin-1 receptor domain-containing adapter protein	Mal, wyatt	P58753	ENSG0000(	Signalling	AY576785	Sepsis, Cytomegalovirus infection, Malaria, Tuberculosis, Systemic lupus erythematosus, Pulmonary tuberculosis, Leprosy	https://www.targetvalidation.org/target/ENSG00000150455/associations	TRAF6 IRAK4 RNF216 MYD88 CCDC47 BTK PRKCD IRAK1 CASP1 EIF2AK2 CAPN1 TLR2 IRAK2 SOCS1 TLR4 CASP1 IRF3 IRF7 IRAK1 TAB2 JAK1 TLR4 UBR2 MYD88 NF1 EIF2AK2 SOCS1 TLR2 UBR1 RPTOR STAT3	Cytoplasm		No		✓
TK1	HUMAN Thymidine kinase, cytosolic	KITH	P04183	ENSG0000(	Kinase,Cancer	BT006941	Lung carcinoma, Ovarian neoplasm, Lung neoplasm, Papillary thyroid carcinoma, Liver and Intrahepatic Biliary tract carcinoma, Familial mediterranean fever, Acute lymphoblastic leukemia, Pancreatic adenocarcinoma	https://www.targetvalidation.org/target/ENSG00000167900/associations	CDK2 CDK1 CDKN1A	Cytoplasm,		Yes	https://www.bindingdb.org/uniprot/P04183	✓
TKT	HUMAN Transketolase	TKT	P29401	ENSG0000(	Others	AK289454	Wernicke-Korsakoff syndrome, Wernicke encephalopathy, Hypoxia, Schizophrenia, Multiple sclerosis, Thiamine deficiency, Hepatocellular carcinoma, Large celll lung carcinoma, Melanoma, Lung carcinoma	https://www.targetvalidation.org/target/ENSG00000163931/associations	AKT1 ISG15			Yes	https://www.bindingdb.org/uniprot/P29401	✓
TLK1	HUMAN Serine/threonine-protein kinase tousled-like 1	KIAA0137, PKU-BETA	Q9UKI8	ENSG0000(	Kinase	D50927	Glioblastoma multiforme, Lung carcinoma, Squamous cell lung carcinoma, Adenoid cystic carcinoma, Peutz-Jeghers syndrome, Herpes simplex infection, Myotonic dystrophy, Tuberculosis, Melanoma	https://www.targetvalidation.org/target/ENSG00000163931/associations	CHEK1 SNAP23 ASF1B RAD9A ASF1A HIST1H1A HIST1H3A; HIST1H3B; HIST1H3C; HIST1H3D; HIST1H3E; HIST1H3F; HIST1H3G ; HIST1H3H; HIST1H3I; HIST1H3J TLK2 MBP CSN1S1	Nucleus		Yes	https://www.bindingdb.org/uniprot/Q9UKI8	✓
TLK2	HUMAN Serine/threonine-protein kinase tousled-like 2	MGC44450, PKU-ALPHA	Q86UE8	ENSG0000(	Kinase	CH471109	Breast carcinoma, Lung carcinoma, Hepatocellular carcinoma, Central neuroblastoma, Prostate carcinoma, Neurofibromatosis 1, Liver carcinoma, Systemic lupus erythematosus	https://www.targetvalidation.org/target/ENSG00000146872/associations	TLK1 IRF7 CHEK1 ASF1B YWHAZ ASF1A MBP FEZ1	Nucleus, Cytoplasm, perinuclear region, cytoskeleton,		Yes	https://www.bindingdb.org/uniprot/Q86UE8	✓

TLX2	HUMAN T-cell leukemia homeobox protein 2	Enx, HOX11L1, NCX, Tlx2	O43763	ENSG0000(	Transcription factor	BC006356	Genetic intestinal disease, Hirschsprung disease, Waardenburg-Shah syndrome, Multiple endocrine neoplasia type 2, Congestive heart failure, Ovarian carcinoma, Central neuroblastoma, Colonic atresia, Microbillus inclusion disease	https://www.targetvalidation.org/target/ENSG00000115297/associations	YWHAH	Nucleus		No		✓
TMEFF1	HUMAN Tomoregulin-1	C9orf2, H7365	Q8IYR6	ENSG0000(	Others	AL353805, AL354917	Cardiac hypertrophy, Leukemia, Brain neoplasm, Cleft lip, Cleft palate, Malignant mesothelioma	https://www.targetvalidation.org/target/ENSG00000241697/associations		Cell membrane	Transmembrane	No		✓
TMEFF2	HUMAN Tomoregulin-2	HPP1, TENB2, TPEF	Q9UIK5	ENSG0000(	Others	AF179274	Li-Fraumeni syndrome, Schizophrenia, Self-reported educational attainment, Barrett esophagus, Prostate carcinoma, Polycystic ovary syndrome, Adenocarcinoma, Bladder neoplasm, Gastric neoplasm, Colorectal neoplasm	https://www.targetvalidation.org/target/ENSG00000144339/associations		Membrane, Secreted,	Transmembrane	No		✓
TMEM108	HUMAN Transmembrane protein 108	KIAA1690, RTLN, UNQ1875/PRO4318	Q6UXF1	ENSG0000(	Others	CH471052	Seasonal allergic rhinitis, Azoospermia, Breast carcinoma, Obesity, Central nervous system cancer, Astrocytoma, Psoriasis, Schizophrenia	https://www.targetvalidation.org/target/ENSG00000144868/associations		Membrane	Transmembrane	No		✓
TNFAIP6	HUMAN Tumor necrosis factor-inducible gene 6 protein	TSG-6, TSG6	P98066	ENSG0000(	Others	AC009311	Renal carcinoma, Renal cell carcinoma, Clear cell renal carcinoma, Juvenile arthritis, Dermatologic disorder, Degenerative polyarthritis, Peritoneal fibrosis, Epstein-Barr virus infection, Tuberculosis, Li-Fraumeni syndrome	https://www.targetvalidation.org/target/ENSG00000123610/associations	ACAN THBS1 FN1			Yes	https://www.drugbank.ca/biodb/polypeptides/P98066	✓
TNFAIP8	HUMAN Tumor necrosis factor alpha-induced protein 8	GG2-1, MDC-3.13, SCC-S2	O95379	ENSG0000(	Others	CR457137	Esophagus squamous cell carcinoma, Juvenile arthritis, Breast carcinoma, Non-Hodgkin's lymphoma, Osteosarcoma, Central nervous system cancer, Glioblastoma multiforme, Ovarian carcinoma, Pancreatic carcinoma, Astrocytoma	https://www.targetvalidation.org/target/ENSG00000145779/associations		Cytoplasm		No		✓
TNFRSF11B	HUMAN Tumor necrosis factor receptor superfamily member 11B	OCIF, OPG	O00300	ENSG0000(	Cancer, Cytokine/Chemokine	AK313710	Juvenile paget disease, Osteoporosis, Rheumatoid arthritis, Osteosclerosis, Hypercalcemia, Angioid Streak, Uremia, Hyperalgesia, Ghosal hematodiaphyseal dysplasia, Ependymoma	https://www.targetvalidation.org/target/ENSG00000164761/associations	TNFSF10 TNFSF11 TNFSF11	Secreted		No		✓
TNIP1	HUMAN TNFAIP3-interacting protein 1	ABIN-1, KIAA0113, NAF1, VAN	Q15025	ENSG0000(	Others	AB177543	Systemic lupus erythematosus, Psoriasis, Psoriasis vulgaris, Systemic Scleroderma, Lupus nephritis, Chronic obstructive pulmonary disease, Sjogren's syndrome, Autoimmune lymphoproliferative syndrome	https://www.targetvalidation.org/target/ENSG00000145901/associations	MAPK1 TNFAIP3 NFKB1 S100B MAPK1 NFKB1 IKBKG	Cytoplasm, Nucleus,		No		✓
TNK2	HUMAN Activated CDC42 kinase 1	ACK, ACK1	Q07912	ENSG0000(	Kinase,Cancer	BC008884	Parkinson's disease, Inflammatory bowel disease, Colitis, Liver neoplasm, Gastric neoplasm, Mammary neoplasm, Prostate carcinoma	https://www.targetvalidation.org/target/ENSG00000061938/associations	CDC42 BMX SRC PDGFRB FYN EGFR CLTC EGFR CTTN SRC FYN KDM3A PDGFRB AKT1 WASL CDC42 RET AR BCAR1 GRB2 DDR1 PPP1R13L NCK1 SNX9 WWOX MERTK WAS RASGRF1 SYNJ1 RASGEF1C CACNB2 GOLGA2 MCF2	Cell membrane, Nucleus, Endosome, Cell junction, adherens junction Cytoplasmic vesicle membrane, Peripheral membrane protein, Cytoplasmic side, Cytoplasmic vesicle, clathrin-coated vesicle, Membrane, clathrin-coated pit,		Yes	https://www.bindingdb.org/uniprot/Q07912	✓

TOLLIP	HUMAN Toll-interacting protein	TOLLIP	Q9H0E2	ENSG0000(	Signalling	AK127123	Idiopathic pulmonary fibrosis, Amyotrophic lateral sclerosis, Cerebrovascular accident, Subarachnoid hemorrhage, Intracranial hemorrhage, Sepsis, Tuberculosis, Leprosy, Atopic eczema, Cutaneous leishmaniasis	https://www.targetvalidation.org/target/ENSG00000078902/associations	TRAF6 IL1RAP PEG10 MAPK14 RCAN1 IRAK1 EIF2AK2 IL1R1 UBC TOM1L1 TLR2 IRAK2 TLR3 TLR4 UBC ITCH CAV1 IRAK1 TLR4 DIABLO WRNIP1 TLR2 MAGED1 TLR3 DZIP3 CSTF1 TRAF6 IL1R1 XIAP BIRC2 NT5DC2	Cytoplasm	No		✓
TOM1	HUMAN Target of Myb protein 1	TOM1	O60784	ENSG0000(	Cancer	AK297827	Multiple myeloma, Monoclonal gammopathy, Sepsis, Breast carcinoma, Bipolar disorder, Acute lymphocytic leukemia, Cystic fibrosis, Precursor cell lymphoblastic leukemia lymphoma, Hereditary spastic paraplegia, Colonic neoplasm	https://www.targetvalidation.org/target/ENSG00000100284/associations	UBC CLTC	Cytoplasm	No		✓
TP53	HUMAN Cellular tumor antigen p53	p53	P04637	ENSG0000(	Kinase,Transcription factor,Cancer	M14694	Inherited cancer-predisposing syndrome, Liver carcinoma, Sarcoma, Adrenocortical carcinoma, Osteosarcoma, Li-Fraumeni syndrome, Gastric neoplasm, Squamous cell carcinoma, Pancreatic neoplasm, Choroid plexus papilloma	https://www.targetvalidation.org/target/ENSG00000141510/associations	CSNK1G3 TRRAP CDC14A ALK PLAGL1 ING1 DAXX STK17A NLK GTSE1 CDK12 CDKN2AIP TOPORS LATS2 SMYD2 SIRT7 KMT5A NLRC4 CSNK1G1 MSL2 EHMT1 KAT8 PLK3 HIPK3 HIPK2 ING2 BBC3 CDK19 GNL3	Cytoplasm, Nucleus, PML body, Endoplasmic reticulum, Mitochondrion matrix,	Yes	https://www.bindingdb.org/uniprot/P04637	✓
TP53RK	HUMAN TP53-regulating kinase	C20orf64, prpk	Q96S44	ENSG0000(	Kinase	AL031055, AL133520	Glioblastoma multiforme, Lung carcinoma, Breast carcinoma, Narcolepsy, Colorectal carcinoma, Colorectal cancer, Chronic lymphocytic leukemia, Anaplastic large cell lymphoma, Multiple myeloma	https://www.targetvalidation.org/target/ENSG00000172315/associations	AKT1 TP53 ERBB2 CALCOCO2 TPRKB	Nucleus	No		✓
TP63	HUMAN Tumor protein 63	EEC3, KET, OFC8, p51, p63, p73H, p73L, SHFM4, TP73L	Q9H3D4	ENSG0000(	Transcription factor	AF075429	Ectodermal dysplasia syndrome, EEC syndrome, ADULT syndrome, Ankyloblepharon ectodermal defects -cleft lip/palate, Rapp-Hodgkin's syndrome, Hay-Wells syndrome, Bladder exstrophy, Rudiger syndrome 1, Extodactyly	https://www.targetvalidation.org/target/ENSG00000073282/associations	BTRC HIPK2 WWP1 RCHY1 ITCH FBXW7 PPP2R5A ATM MDM2 UBE2I RACK1 BRCA1 TGFBR1 PML CDK2 RPS6KB1 TP53 ABL1 TP73 IKBKB PRKCD YAP1 ATG4A ULK1 ATM BECN1 ATG3 ATG7 MDM2 ATG10 ATG9A ATG5	Nucleus	No		✓
TP73	HUMAN Tumor protein p73	P73	O15350	ENSG0000(	Kinase,Transcription factor	AF079094	Li-Fraumeni syndrome, Alveolar rhabdomyosarcoma, Small cell lung carcinoma, Congenital neurologic anomalies, Squamous cell carcinoma, Lung neoplasm, Cowden syndrome, Primary ciliary dyskinesia	https://www.targetvalidation.org/target/ENSG00000078900/associations	WWOX HIPK2 RCHY1 ITCH SIRT1 KAT2B SMAD2 CASP8 PIN1 PRKAA1 EP300 PRKCD E2F1 RPL11 PMS2 PLK1 YAP1 RPL5 CASP3 STAT1 CDK2 CCNA2 PRKCA SRC FBXO45 HCK CDK1 PRKCB TP53 ABL1 CHUK	Nucleus Cytoplasm,	No		✓
TPI1	HUMAN Triosephosphate isomerase	TPI	P60174	ENSG0000(	Cancer,Cancer-Antigen	U47924	Disorder of glycolysis, Triose phosphate-isomerase deficiency, Hereditary elliptocytosis, Myopathy, Schizophrenia, Congestive heart failure, Alzheimer's disease, Hemolytic anemia, Neurogenic muscular atrophy	https://www.targetvalidation.org/target/ENSG00000111669/associations	CASP1 CFL1		Yes	https://www.bindingdb.org/uniprot/P60174	✓
TPM1	HUMAN Tropomyosin alpha-1 chain	C15orf13, CMH3, TMSA	P09493	ENSG0000(	Cancer,Cancer-Antigen	M19715	Cardiomyopathy, Hypertrophic cardiomyopathy, Left ventricular noncompaction, Congestive heart failure, Familial dilated cardiomyopathy, Qualitative or quantitative defects of dystrophin	https://www.targetvalidation.org/target/ENSG00000140416/associations	RIPK3 DAPK1 PIK3CA PRKCA TPM2 PKD2 TNNT2 TNNT1	Cytoplasm, cytoskeleton,	No		✓

TPM3	HUMAN Tropomyosin alpha-3 chain	hTM5	P06753	ENSG0000(	Cancer	AL590431	Congenital myopathy, Nemaline myopathy, Congenital fiber-type disproportion myopathy, Cap myopathy, Congenital fiber type disproportion, Thyroid neoplasm, Colonic neoplasm, Head and Neck squamous cell carcinoma, Bladder carcinoma	https://www.targetvalidation.org/target/ENSG00000143549/associations	RIPK3<br>IKBKE<br>IL7R<br>CHUK<br>ACTA1<br>TMOD1	Cytoplasm, cytoskeleton,		No		✓
TPM4	HUMAN Tropomyosin alpha-4 chain	TPM4	P67936	ENSG0000(	Cancer	BT019634	Prostate carcinoma, Melanoma, Prostate adenocarcinoma, Asbestosis, Acute coronary syndrome, Osteoporosis, Esophageal neoplasm, Hepatocellular carcinoma, Gastric adenocarcinoma, Rectal adenocarcinoma	https://www.targetvalidation.org/target/ENSG00000167460/associations	IL7R	Cytoplasm, cytoskeleton,		No		✓
TPTE	HUMAN putative tyrosine-protein phosphatase TPTE	CT44	P56180	ENSG0000(	Others	AY219888	Lung carcinoma, Down syndrome, Autistic disorder, Colorectal carcinoma, Macrocephaly, Tongue squamous cell carcinoma, Autism, Hepatocellular carcinoma	https://www.targetvalidation.org/target/ENSG00000274391/associations		Membrane, Multi-pass membrane protein,	Transmembrane	No		✓
TRAF1	HUMAN TNF receptor-associated factor 1	EBI6	Q13077	ENSG0000(	Kinase,Signalling,Cancer	AK315476	Autoimmune disease, Rheumatic disease, Arthritis, Rheumatoid arthritis, Li-Fraumeni syndrome, Patent ductus arteriosus, Systemic lupus erythematosus, Glioma	https://www.targetvalidation.org/target/ENSG00000056558/associations	MAP3K14<br>CASP10<br>TANK<br>TICAM1<br>TRADD<br>CASP8<br>RIPK1<br>BIRC2<br>BIRC3<br>TRAF2<br>CD40<br>TNFRSF1B<br>TNFRSF1A SRC<br>RIPK2<br>CHUK<br>IKBKB<br>SRC<br>TRAF3<br>MAP3K14<br>TICAM1<br>TRAF2<br>CD40<br>BIRC3<br>TRAF6<br>TRADD<br>TNFRSF1B			No		✓
TRAF2	HUMAN TNF receptor-associated factor 2	TRAP3	Q12933	ENSG0000(	Kinase,Cancer	AK298370	Hyper-IgM syndrome with susceptibility to opportunistic infections, Hyper-IgM syndrome without susceptibility to opportunistic infection, Hyper-IgM syndrome type 3, X-linked hyper-IgM syndrome, Hepatic veno-occlusive disease-immunodeficiency, Alzheimer's disease, Glioma, Multiple myeloma, Non-Hodgkin's lymphoma	https://www.targetvalidation.org/target/ENSG00000127191/associations	TNFRSF11A<br>TNFRSF18<br>RIPK3<br>MAP4K5<br>TNIK<br>IP6K2<br>TBK1<br>TAB2<br>DIABLO<br>CYLD<br>TAOK3<br>MYLK2<br>PEG3<br>MAP3K5<br>MAP3K14<br>RNF2<br>COPS5<br>TANK<br>TRPC4AP<br>TAB3<br>MAP4K3<br>TICAM1<br>MAVS<br>MAST2<br>DAB2IP<br>PKN1<br>TAB1<br>TRIP6	Cytoplasm		No		✓
TRAF3IP1	HUMAN TRAF3-interacting protein 1	IFT54, MIP-T3, MIPT3	Q8TDR0	ENSG0000(	Others	CH471063	Ciliopathy, Senior-Loken syndrome, Brachydactyly type C, Pelvis-shoulder dysplasia, Nephronophthisis,<br>Retinal dystrophy, Cognitive delay	https://www.targetvalidation.org/target/ENSG00000204104/associations	TRAF3<br>IL13RA1	Cytoplasm, cytoskeleton		No		✓
TRAF4	HUMAN TNF receptor-associated factor 4	CART1, MLN62, RNF83	Q9BUZ4	ENSG0000(	Kinase,Signalling,Cancer	DQ323999	Breast carcinoma, Autosomal recessive spondylocostal dysostosis, Alzheimer's disease, HIV Infection, Crohn's disease, Anastomosis, Osteosarcoma	https://www.targetvalidation.org/target/ENSG00000076604/associations	MAP3K4<br>TRAF6<br>TICAM1<br>TRAF2<br>IRAK1<br>RPS6KB1<br>NCF1<br>NGFR<br>TP53<br>CHUK<br>BCKDK<br>MAP3K4<br>IRAK1<br>TICAM1<br>TRAF6<br>SMURF1	Cytoplasm, Nucleus, perinuclear region, Cell junction, tight junction, Cell membrane, Peripheral membrane protein, Cytoplasmic side, cytoskeleton		No		✓
TRAF5	HUMAN TNF receptor-associated factor 5	RNF84	O00463	ENSG0000(	Kinase,Signalling	CR536557	Primary immunodeficiency, Hyper-IgM syndrome with susceptibility to opportunistic infections, Common variable immunodeficiency, Alzheimer's disease, Multiple myeloma, HIV infection, Hepatitis C infection, Non-Hodgkin's lymphoma, Uveomeningoencephalitic syndrome, Ankylosing spondylitis	https://www.targetvalidation.org/target/ENSG00000082512/associations	TRAF6<br>MAP3K5<br>MAP3K14<br>MAVS<br>TRADD<br>RIPK1<br>TRAF3<br>CD40<br>RIPK2<br>MAP3K7<br>LTBR<br>IL6ST<br>TRAF2<br>CD40<br>MAP3K11	Cytoplasm		No		✓
TRAF6	HUMAN TNF receptor-associated factor 6	RNF85	Q9Y4K3	ENSG0000(	Kinase,Cancer	AC009656	Autoimmune disease, Arthritis, Rheumatoid arthritis, Multiple myeloma, Osteoporosis, Hepatitis C infection, Non-Hodgkin's lymphoma, Ectodermal dysplasia	https://www.targetvalidation.org/target/ENSG00000175104/associations	NUMBL<br>TNFRSF11A IKBKG<br>IRAK3<br>PSMD13<br>STUB1<br>MALT1<br>STK26<br>TAB2<br>SPHK1<br>IRAK4<br>CYLD<br>EDA2R<br>PELI2<br>TOLLIP<br>STRADB<br>AMBRA1<br>RBCK1<br>CARD11<br>TRAF4<br>ECSIT<br>MYD88<br>MAP3K3<br>KIR2DL4<br>MAP3K5<br>MAP3K14<br>PSMD1<br>RANBP9<br>AJUBA	Cytoplasm		Yes	https://www.bindingdb.org/uniprot/Q9Y4K3	✓

TRAIP	HUMAN E3 ubiquitin-protein ligase TRAIP	RNF206, TRIP	Q9BWF2	ENSG0000(	Others	BC000310	Microcephalic primordial dwarfism, Seckel syndrome, Breast carcinoma, Dwarfism, Cone-shaped epiphyses, Hypoplastic mandible condyle, Bilateral fifth finger clinodactyly	https://www.targetvalidation.org/target/ENSG00000183763/associations	TRAF3 SYK PCNA CYLD POLH	Cytoplasm, perinuclear region, Nucleus	No	✓
TRIB2	HUMAN Tribbles homolog 2	GS3955, TRB2	Q92519	ENSG0000(	Kinase	AC009486	Ovarian carcinoma, Uveitis, Acute erythroblastic leukemia, Liver and intrahepatic biliary tract carcinoma, Narcolepsy, Melanoma, Prostate carcinoma, Multiple scelerosis	https://www.targetvalidation.org/target/ENSG00000071575/associations	RPS6KB1 BTRC UBQLN4 HLCS	Cytoplasm	No	✓
TRIB3	HUMAN Tribbles homolog 3	C20orf97, dJ1103G7.3, TRB3	Q96RU7	ENSG0000(	Kinase	AK026945	Breast carcinoma, Adenocarcinoma, Metabolic syndrome X, Anoxia, Hyperglycemia, Alzheimer's disease, Myocardial ischemia, Type II diabetes mellitus, Melanoma, Glioblastoma multiforme	https://www.targetvalidation.org/target/ENSG00000101255/associations	SIAH1 ELAVL1 AKT2 AKT1 ATF4 PRKCD EP400 ATF4 COPS6 MAP2K1 PPP2R1B AKT1 RELA AKT2 DDIT3 SETDB1 MAP2K7 KAT5 EEF1G GIT1 GDF9 LUZP1 PBX2 ATF5	Nucleus	No	✓
TRIM32	HUMAN E3 ubiquitin-protein ligase TRIM32	BBS11, HT2A, LGMD2H	Q13049	ENSG0000(	Transcription factor	AL133284	Ciliopathy, Bardet-Biedl syndrome, Muscular dystrophy, Myopathy, Obesity, Hypoplastic ovary, Limb-girdle muscular dystrophy type 2H	https://www.targetvalidation.org/target/ENSG00000119401/associations	XIAP UBE2N IRAK1 TMEM173 ABI2 GLIS2	Cytoplasm,	No	✓
TRIM37	HUMAN E3 ubiquitin-protein ligase TRIM37	KIAA0898, MUL, POB1	O94972	ENSG0000(	Cancer,Cancer-Antigen	AK294850	Mulibrey nanism, Breast carcinoma, Azoospermia, Hepatomegaly, Nephroblastoma, Congestive heart failure, Hypoplastic frontal sinuses, Hepatocellular carcinoma, Osteosarcoma	https://www.targetvalidation.org/target/ENSG00000108395/associations	PBK TRAF6 UBB	Cytoplasm, perinuclear region	No	✓
TRIP11	HUMAN Thyroid receptor-interacting protein 11	CEV14, GMAP-210, GMAP210, Trip230	Q15643	ENSG0000(	Cancer	BC146845	Achondrogenesis, Achondrogenesis type 1A, Lethal skeletal dysplasia, Long philtrum, Hypoplastic ischia, Hypoplastic mandible condyle, Lung carcinoma, Kidney neoplasm, Esophageal carcinoma	https://www.targetvalidation.org/target/ENSG00000100815/associations	ARNT RB1 THRB THRA	Golgi apparatus, cis-Golgi network membrane	No	✓
TRMT10C	HUMAN Mitochondrial ribonuclease P protein 1	MRPP1, RG9MTD1	Q7L0Y3	ENSG0000(	Others	BC035967	Breast carcinoma, Glioblastoma multiforme, Lung carcinoma, Diffuse large B-cell lymphoma, Ovarian carcinoma, Colorectal cancer, Ductal adenocarcinoma, Anaplastic large cell lymphoma, Gastric carcinoma, HSD10 disease	https://www.targetvalidation.org/target/ENSG00000174173/associations	GRSF1 HSD17B10	Mitochondrion matrix, mitochondrion nucleoid	No	✓
TROVE2	HUMAN 60 kDa SS-A/Ro ribonucleoprotein	Ro60, SSA2	P10155	ENSG0000(	Cancer,Cancer-Antigen	U44388	Primary immunodeficiency, Immunodeficiency due to a complement cascade protein anomaly, Autoimmune lymphoproliferative syndrome, Hyper-IgM syndrome with susceptibility to opportunistic infections, Immunodeficiency due to an early component of complement deficiency, Systemic lupus erythematosus, Sjogren's syndrome, Autoimmune disease, Lupus vulgaris, Congenital heart block	https://www.targetvalidation.org/target/ENSG00000116747/associations	SSB RAB11FIP5 TRIM21	Cytoplasm	No	✓
TSC22D1	HUMAN TSC22 domain family protein 1	TGFB1I4, TSC22, T22D1, KIAA1994	Q15714	ENSG0000(	Transcription factor	D87061	Psoriasis, Anti-neutrophil antibody associated vasculitis, Ovarian carcinoma, Diabetic nephropathy, Type II diabetes mellitus, Prostate carcinoma, Neoplastic cell transformation	https://www.targetvalidation.org/target/ENSG00000102804/associations	MAP3K12 TPT1 TP53 TSC22D4	Cytoplasm	No	✓
TSC22D4	HUMAN TSC22 domain family protein 4	THG-1, TILZ2	Q9Y3Q8	ENSG0000(	Transcription factor	CH236956	Self-reported educational attainment, Medulloblastoma, Cachexia, Hypertriglyceridemia, Wasting syndrome, Ovarian carcinoma, Turner syndrome	https://www.targetvalidation.org/target/ENSG00000166925/associations	NRBP1 STK17A TSC22D1 PRKCD CDK5 WEE1 OXSR1	Nucleus	No	✓

TSG101	HUMAN Tumor susceptibility gene 101 protein	VPS23	Q99816	ENSG0000(	Transcription factor,Cancer	U82130	HIV infection, Breast carcinoma, Ovarian neoplasm, Hepatitis C infection, Prostate neoplasm, Burkitts lymphoma, Wilms tumor, Hepatocellular carcinoma	https://www.targetvalidation.org/target/ENSG00000074319/associations	VPS28 GMCL1 VPS37A VPS37D LRSAM1 EP300 BCR UBC HCK NR3C2 NR3C1 TOM1L1 MGRN1 HGS VPS37C DAXX CDKN1A MDM2 AKAP9 AATF VPS37B VPS28 MVB12A	Cytoplasm	Yes	https://www.bindingdb.org/uniprot/Q99816	✓
TSGA10	HUMAN Testis-specific gene 10 protein	CEP4L	Q9BZW7	ENSG0000(	Kinase	AC019097	Azoospermia, Osteosarcoma, Acute lymphoblastic leukemia, Bladder neoplasm, Ovarian neoplasm, Liver carcinoma, Melanoma, Hematopoietic neoplasm	https://www.targetvalidation.org/target/ENSG00000135951/associations	HIF1A FLNA BIRC2	Cytoplasm	No		✓
TSPY1	HUMAN Testis-specific Y-encoded protein 1	CT78, TSPY	Q01534	ENSG0000(	Others	BC121114	Prostate carcinoma, Hepatocellular carcinoma, Male infertility, Gonadoblastoma, Hypogonadism, Seminoma, Hyperplasia, Pituitary gland adenoma	https://www.targetvalidation.org/target/ENSG00000258992/associations	CSNK2A1 AR	Cytoplasm	No		✓
TSPY2	HUMAN Testis-specific Y-encoded protein 2	TSPYQ1	A6NKD2	ENSG0000(	Others	NA	NA	NA		Cytoplasm	No		✓
TSPY3	HUMAN Testis-specific Y-encoded protein 3	CT78	P0CV98	ENSG0000(	Others	AC006158	Gonadoblastoma, Prostate carcinoma, Triploidy syndrome, Ovarian small cell carcinoma	NA		Cytoplasm	No		✓
TSSK1B	HUMAN Testis-specific serine/threonine-protein kinase 1	FKSG81, SPOGA1, SPOGA4, STK22A, STK22D, TSSK1	Q9BXA7	ENSG0000(	Kinase	AC010431	Azoospermia, Fibrosis, Endothelial dysfunction, Systemic scleroderma, Chronic graft-versus-host disease, Unipolar depression	https://www.targetvalidation.org/target/ENSG00000212122/associations	CHEK2 TGFBR2 TGFBR1	Cytoplasm	Yes	https://www.bindingdb.org/uniprot/Q9BXA7	✓
TSSK2	HUMAN Testis-specific serine/threonine-protein kinase 2	FLJ38613, SPOGA2, STK22B	Q96PF2	ENSG0000(	Kinase	AC004471	Azoospermia, Male infertility, Autoimmune disease, Systemic scleroderma, Connective tissue disease	https://www.targetvalidation.org/target/ENSG00000206203/associations	TFG TSKS	Cytoplasm	Yes	https://www.bindingdb.org/uniprot/Q96PF2	✓
TSSK3	HUMAN Testis-specific serine/threonine-protein kinase 3	SPOGA3, STK22C	Q96PN8	ENSG0000(	Kinase	CH471059	Azoospermia, Acute myeloid leukemia	https://www.targetvalidation.org/target/ENSG00000162526/associations	SMAD4		Yes	https://www.bindingdb.org/uniprot/Q96PN8	✓
TSSK6	HUMAN Testis-specific serine/threonine-protein kinase 6	CT72, FLJ24002, SSTK	Q9BXA6	ENSG0000(	Kinase	AK172841	Azoospermia, Male infertility with teratozoospermia due to single gene mutation, Male infertility with azoospermia or oligozoospermia due to single gene mutation, Partial chromosome Y deletion, Congenital bilateral absence of vas deferens, Primary ovarian insufficiency, Deafness-infertility syndrome, 46-XX testicular disorder of sex development	https://www.targetvalidation.org/target/ENSG00000178093/associations			No		✓
TTBK2	HUMAN Tau-tubulin kinase 2	KIAA0847, SCA11	Q6IQ55	ENSG0000(	Kinase	BC041876	Autosomal dominant cerebellar ataxia, Spinocerebellar ataxia type 11, Renal carcinoma, Frontotemporal dementia, Amyotrophic lateral sclerosis, Movement disorder, Renal cell carcinoma, Spinocerebellar degeneration	https://www.targetvalidation.org/target/ENSG00000128881/associations	CTNNB1 CDK5 APC BID TP53 YWHAQ CSNK1E PSEN2 SV2A NFATC3 KIF2A SLC18A2	Cell projection, cilium Cytoplasm, cytoskeleton, microtubule organizing center, centrosome, centriole, cytosol, Nucleus,	No		✓
TTF2	HUMAN Transcription termination factor 2	HuF2	Q9UNY4	ENSG0000(	Transcription factor	AL445231, AL391476	Breast carcinoma, Acute lymphoblastic leukemia, Lung carcinoma, Small cell lung carcinoma, Ovarian carcinoma, Hypothyroidism, Thyroid agenesis, Choanal atresia, Uranostaphyloschisis, Bifid epiglottis	https://www.targetvalidation.org/target/ENSG00000116830/associations	PRKACA CDC5L	Cytoplasm	No		✓
TTK	HUMAN Dual specificity protein kinase TTK	CT96, MPH1, MPS1, MPS1L1	P33981	ENSG0000(	Kinase	AL133475	Lung carcinoma, Central nervous system cancer, Hepatocellular carcinoma, Polycystic ovary syndrome, Melanoma, Lymphoma, Colorectal cancer, Breast carcinoma, Gastric carcinoma, Mucopolysaccharidoses	https://www.targetvalidation.org/target/ENSG00000112742/associations	CDK5 HSPA9 CDC27 MAPK1 MAPK3 CDK2 BRAF CHEK2 TACC2 BCAS2 NDC80 TUBB EIF2S1 EGFR BUB1B BLM SMAD4 TP53 TACC2 FAS ABL1 PRC1 E2F4 CHEK2 CDCA8 RUNX2 TUBB1 RBBP5 BANF1 HSPA9		Yes	https://www.bindingdb.org/uniprot/P33981	✓

TUBA4A	HUMAN Tubulin alpha-4A chain	H2-ALPHA, TUBA1	P68366	ENSG0000(	Signalling	AK299958	Neurodegenerative disease, Amyotrophic lateral sclerosis, Mammary neoplasm, Non-small cell lung carcinoma, Multiple sclerosis, Neural tube defect, Prostate carcinoma, Melanoma, Gout, Sarcoma	https://www.targetvalidation.org/target/ENSG00000127824/associations	TAOK2 HDAC6 MARK4 TTL SIRT2 NUMA1 RAC1 PLK1 SYK ZAP70 VDAC1 IL7R EPB41 FYN MGRN1 TUBB YWHAG MAP1A GPHN TUBB4B TUBA1A TUBB3	Cytoplasm, cytoskeleton,	Yes		✓
												https://www.drugbank.ca/biodb/polypeptides/P68366	
TUBB	HUMAN Tubulin beta chain	OK/SW-cl.56, Tubb5	P07437	ENSG0000(	Others	AF070593	Genetic central nervous system malformation, Multiple benign circumferential skin creases on limbs, Michelin tire baby syndrome, Necrotizing enterocolitis, Heart failure, Dilated cardiomyopathy, Schizophrenia, Narcolepsy, Systemic lupus erythematosus, Lung neoplasm	https://www.targetvalidation.org/target/ENSG00000196230/associations	RIPK3 TAOK2 TBK1 CRIPT MAP3K3 MAP3K14 MARK4 RICTOR TTBK1 DPYSL2 NUMA1 IKBKE BMPR2 RIPK1 MAP3K1 MAP3K10 MAP1A TUBA4A DYNLL1 YWHAE PLK1 ZAP70 TTK IL7R MAPT ALDOA STAU1 RIPK2 CHUK IKBKB	Cytoplasm, cytoskeleton	Yes		✓
												https://www.drugbank.ca/biodb/polypeptides/P07437	
TUBGCP3	HUMAN Gamma-tubulin complex component 3	GCP3, Spc98p	Q96CW5	ENSG0000(	Cancer,Cancer-Antigen	BT009772	Pancreatic carcinoma, Breast carcinoma, Astrocytoma, Mucinous adenocarcinoma, Glioblastoma, Liver neoplasm, Melanoma, Visna, Osteosarcoma, Prostate adenocarcinoma	https://www.targetvalidation.org/target/ENSG00000126216/associations	TUBGCP2	Cytoplasm, cytoskeleton, microtubule organizing center, centrosome,	No		✓
TULP2	HUMAN Tubby-related protein 2	CT65, TUBL2	O00295	ENSG0000(	Others	NA	Azoospermi, Breast carcinoma, Asthma, Lung carcinoma, Colorectal adenocarcinoma, Retinitis pigmentosa, Retinitis pigmentosa 14, Cone-Rod dystrophy, Prostate carcinoma, Thrombocytopenia	https://www.targetvalidation.org/target/ENSG00000104804/associations		Cytoplasm	No		✓
TWF1	HUMAN Twinfilin-1	A6, PTK9	Q12792	ENSG0000(	Kinase	AK297206	Inflammatory bowel disease, Melanoma, Primary Fanconi syndrome, Inclusion body myopathy with Paget disease of bone and frontotemporal dementia, Multiple myeloma, Lymphoma, Breast carcinoma, Mammary neoplasm, Anaplastic large cell lymphoma, Lung carcinoma	https://www.targetvalidation.org/target/ENSG00000151239/associations	CAP2 ACTC1 BMPR2	Cytoplasm, cytoskeleton	No		✓
TWF2	HUMAN Twinfilin-2	A6r, A6RP, PTK9L	Q6IBS0	ENSG0000(	Others	CR456732	Li-Fraumeni syndrome, Breast carcinoma, Hypertriglyceridemia, Breast adenocarcinoma, Acute quadriplegic myopathy, Juvenile dermatomyositis	https://www.targetvalidation.org/target/ENSG00000247596/associations	CHGB	Cytoplasm, cytoskeleton	No		✓
TWIST2	HUMAN Twist-related protein 2	bHLHa39, Dermo-1, DERMO1	Q8WVJ9	ENSG0000(	Transcription factor	BC017907	Ablepharon macrostomia syndrome, Barber-Say syndrome, Focal facial dermal dysplasia, Focal facial dermal dysplasia type III, Cervix carcinoma, Breast carcinoma, Colorectal cancer, Cervical cancer, Androgenetic alopecia	https://www.targetvalidation.org/target/ENSG00000233608/associations	MEF2C SREBF1	Nucleus	No		✓
TXN2	HUMAN Thioredoxin, mitochondrial	MT-TRX, TRX2	Q99757	ENSG0000(	Cancer,Cancer-Antigen	CR456601	Hypertension, Psoriasis, Spina bifida, Neural tube defect, Head and Neck neoplasm, Osteosarcoma, Breast carcinoma, Hypertension, Malaria, Glaucoma	https://www.targetvalidation.org/target/ENSG00000100348/associations	LY6E	Mitochondrion	Yes		✓
												https://www.bindingdb.org/uniprot/Q99757	
TXNRD1	HUMAN Thioredoxin reductase 1, cytoplasmic	GRIM-12, Trxr1, KDRF	Q16881	ENSG0000(	Cancer	AF077367	Leukemia, Acute myeloid leukemia, Diphtheria, Adenoma, Epilepsy, Colorectal Neoplasm, Myocardial ischemia, Degenerative polyarthritis, Mesothelioma, Amyotrophic lateral sclerosis	https://www.targetvalidation.org/target/ENSG00000198431/associations	GABPA CAV1 ISG15 GLRX2	Cytoplasm, Nucleus,	Yes		✓
												https://www.bindingdb.org/uniprot/Q16881	

TYMS	HUMAN Thymidylate synthase	TS, Tsase, OK/SW-cl.29	P04818	ENSG0000(	Cancer	AB077208	Lung carcinoma, Pancreatic neoplasm, Colorectal neoplasm, Gastric neoplasm, Mammary neoplasm, Rectal neoplasm, Squamous cell carcinoma, Liver carcinoma, Non-Hodgkin's lymphoma, keloid	https://www.targetvalidation.org/target/ENSG00000176890/associations	CSNK2A1 CSNK2A2	Nucleus		Yes		✓
													https://www.bindingdb.org/uniprot/P04818	
TYRO3	HUMAN Tyrosine-protein kinase receptor TYRO3	Brt, Dtk, Etk-2, Rek, RSE, Sky, Tif	Q06418	ENSG0000(	Kinase,Cancer	D50479	Autosomal dominant nocturnal frontal lobe epilepsy, Familial mesial temporal lobe epilepsy with febrile seizures, Generalized epilepsy with febrile seizures-plus, Familial temporal epilepsy, Thrombosis, Atherosclerosis, Neoplasm metastasis, Lung neoplasm, Autoimmune disease, Melanoma	https://www.targetvalidation.org/target/ENSG00000092445/associations	IKBKG GAS6 SRC PROS1 FYN YES1 PRKCD PROS1 PIK3R1 CBL FGR ZAP70 RANBP9 GAS6	Cell membrane, Single-pass type I membrane protein,	Transmembrane	Yes		✓
													https://www.bindingdb.org/uniprot/Q06418	
TYRO3_int	HUMAN Tyrosine-protein kinase receptor TYRO3	Brt, Dtk, Etk-2, Rek, RSE, Sky, Tif	Q06418	ENSG0000(	Kinase,Cancer	D50479	Autosomal dominant nocturnal frontal lobe epilepsy, Familial mesial temporal lobe epilepsy with febrile seizures, Generalized epilepsy with febrile seizures-plus, Familial temporal epilepsy, Thrombosis, Atherosclerosis, Neoplasm metastasis, Lung neoplasm, Autoimmune disease, Melanoma	https://www.targetvalidation.org/target/ENSG00000092445/associations	IKBKG GAS6 SRC PROS1 FYN YES1 PRKCD PROS1 PIK3R1 CBL FGR ZAP70 RANBP9 GAS6	Cell membrane, Single-pass type I membrane protein,	Transmembrane	Yes		✓
													https://www.bindingdb.org/uniprot/Q06418	
TYRP1	HUMAN 5,6-dihydroxyindole-2-carboxylic acid oxidase	b-PROTEIN, CAS2, CATB, GP75, OCA3, TRP, TYRP	P17643	ENSG0000(	Others	D83059	Oculocutaneous albinism, Oculocutaneous albinism type 3, Melanoma, Nystagmus, Freckles, Strabismus, Achromasia, Adenocarcinoma, Lung neoplasm, Rieger anomaly	https://www.targetvalidation.org/target/ENSG00000107165/associations	TP53 GIPC1 TYR	Melanosome membrane	Transmembrane	No		✓
UBA1	HUMAN Ubiquitin-like modifier-activating enzyme 1	A1S9T, CFAP124, GXP1, POC20, UBE1, UBE1X	P22314	ENSG0000(	Cancer,Cancer-Antigen	CH471164	X-linked distal arthrogryposis multiplex congenita, Ovarian carcinoma, Acute lymphoblastic leukemia, Spinal muscular atrophy, Hyperkyphosis, Severe hypotonia, Acquired scoliosis, Gait abnormality, Wiskott-Aldrich syndrome, Steinert myotonic dystrophy	https://www.targetvalidation.org/target/ENSG00000130985/associations	CSNK2A1 CSNK2A2 MGMT UBC CDK1 CSNK2A1 UBE2A UBE2E1 UBE2B GRB2 CDC34 UBE2D2 UBE2R2 UBE2K UBB GAN	Cytoplasm		Yes		✓
													https://www.bindingdb.org/uniprot/P22314	
UBA3	HUMAN NEDD8-activating enzyme E1 catalytic subunit	hUba3, NAE2, UBE1C	Q8TBC4	ENSG0000(	Others	AC092060	Melanoma, Metastatic melanoma, Breast carcinoma, Breast adenocarcinoma, Down syndrome, Juvenile dermatomyositis, Schizophrenia, Colorectal adenocarcinoma, Amyotrophic lateral sclerosis	https://www.targetvalidation.org/target/ENSG00000144744/associations	NEDD8 NAE1 UBE2M ESR1 NEDD8 TBATA			No		✓
UBE2I	HUMAN SUMO-conjugating enzyme UBC9	UBC9, UBCE9	P63279	ENSG0000(	Cancer,Cancer-Antigen	U29092	Ovarian carcinoma, Glioma, Breast neoplasm, Melanoma, Mammary neoplasm, Lung neoplasm, Alzheimer's disease, Lymphatic metastasis, Ovarian carcinoma, Psoriasis	https://www.targetvalidation.org/target/ENSG00000103275/associations	HIPK2 MAPKAPK5 MAP3K1 UBE2K PTEN BLM BCAM RANBP2 FHIT RANGAP1 HNF4A ETV6 BRCA1 CBS PCGF2 PML APEX1 FAS KCNA5 WT1 XBP1 TCF3 ATF2 ETS1 TOP1 EGR2 AR PARP1 CDK1 JUN ESR1 PIAS1 RIPK2	Nucleus, Cytoplasm,		Yes		✓
													https://www.bindingdb.org/uniprot/P63279	
UBE2Q1	HUMAN Ubiquitin-conjugating enzyme E2 Q1	NICE-5, PRO3094, UBE2Q	Q7Z7E8	ENSG0000(	Others	AK293986	Breast carcinoma, Acute lymphoblastic leukemia, Colorectal cancer, Sjogren's syndrome, Hepatocellular carcinoma, Multiple myeloma, Prostate adenocarcinoma, Squamous cell lung carcinoma	https://www.targetvalidation.org/target/ENSG00000160714/associations	B4GALT1 TP53	Nucleus		No		✓
UBE2V1	HUMAN Ubiquitin-conjugating enzyme E2 variant 1	CROC-1, CROC1, UBE2V, UEV-1, UEV1A, P/OKcl.19	Q13404	ENSG0000(	Cancer,Cancer-Antigen	U97279	Breast carcinoma, Lung carcinoma, Prostate carcinoma, Stroke, Merkel cell carcinoma, Myeloid leukemia, Xeroderma pigmentosum, Rett syndrome, Autism spectrum disorder, Cytomegalovirus infection	https://www.targetvalidation.org/target/ENSG00000244687/associations	TRAF6 UBE2N NPR2 RIPK2 IKBKG TRAF6	Nucleus		No		✓

UBP1	HUMAN Upstream-binding protein 1	LBP-1a, LBP1	Q9NZI7	ENSG0000(	Transcription factor	CR749798	Juvenile dermatomyositis, Breast adenocarcinoma, Lhermitte-Duclos disease, Neuropathic pain, Alpha-Thalassemia, Melanoma, Fanconi anemia, Neurofibromatosis type 1, Lung adenocarcinoma	https://www.targetvalidation.org/target/ENSG00000153560/associations	TFCP2L1	Nucleus	No		✓
UCHL3	HUMAN Ubiquitin carboxyl-terminal hydrolase isozyme L3	UCHL3	P15374	ENSG0000(	Others	AK313665	Retinal dystrophy, Retinitis pigmentosa, Progressive cone dystrophy, Late-onset retinal degeneration, Bietti crystalline dystrophy, Parkinson's disease, Lewy body disease, Uterine cervical neoplasm, Wagner disease, Goldmann-Favre syndrome	https://www.targetvalidation.org/target/ENSG00000118939/associations	UBC SMAD1	Cytoplasm,	Yes	https://www.bindingdb.org/uniprot/P15374	✓
UCKL1	HUMAN Uridine-cytidine kinase-like 1	URKL1	Q9NWZ5	ENSG0000(	Kinase	AK223220	Ovarian carcinoma, Acute monocytic leukemia, Pick disease, Breast carcinoma, Acute erythroblastic leukemia, Glioblastoma multiforme, Adenocarcinoma, Progressive supranuclear palsy, Multiple myeloma	https://www.targetvalidation.org/target/ENSG00000198276/associations		Cytoplasm	Yes	https://www.drugbank.ca/biodb/polypeptides/Q9NWZ5	✓
UHMK1	HUMAN Serine/threonine-protein kinase Kist	KIS, Kist	Q8TAS1	ENSG0000(	Kinase	AL359699	Rare genetic intellectual disability with developmental anomaly, Lung carcinoma, Small cell lung carcinoma, Melanoma, Schizophrenia, Bipolar disorder, Plexiform Neurofibroma, Myelodysplastic syndrome, Acute myelocytic leukemia, Nerve sheath tumor	https://www.targetvalidation.org/target/ENSG00000152332/associations	STMN1 SYN1 CDKN1B SMAD4 SMAD1 SMAD2 PPP1R13L CEBPB TGFBR1 XPO1 SMURF1 ACVR1 ZYX NFATC4 IQGAP1 MBP PAM SF1 PIMREG	Nucleus	No		✓
ULK2	HUMAN Serine/threonine-protein kinase ULK2	ATG1B, KIAA0623, Unc51.2	Q8IYT8	ENSG0000(	Kinase	NA	Li-Fraumeni syndrome, Breast carcinoma, Lymphoma, Prostate carcinoma, Malignant mesothelioma, Glioblastoma, Glioma, Tuberculosis, Ependymoma, Colonic neoplasm	https://www.targetvalidation.org/target/ENSG00000083290/associations	RB1CC1 SQSTM1 MTOR PRKACA TP53 ATG13 PRKAA2 PRKAB1 RB1CC1 SETDB1 ATG13 LUC7L2 SYNGAP1 PRKAG3 EEF1B2 POLA2 NASP	Cytoplasmic vesicle membrane	Yes	https://www.bindingdb.org/uniprot/Q8IYT8	✓
ULK4	HUMAN Serine/threonine-protein kinase ULK4	FAM7C1, FLJ20574, REC01035	Q96C45	ENSG0000(	Kinase	AC104305	Multiple myeloma, Bipolar disorder, Hypertensive disease, Agenesis of corpus callosum, Azoospermia, Monoclonal gammopathy, Ciliopathy, Marshall-Smith syndrome, Cutis gyrata-acanthosis nigricans-craniosynostosis	https://www.targetvalidation.org/target/ENSG00000168038/associations	SMURF1		No		✓
UQCRC1	HUMAN Cytochrome b-c1 complex subunit 1, mitochondrial	QCR1, UQCR1	P31930	ENSG0000(	Cancer,Cancer-Antigen	CH471055	Psoriasis, Ovarian carcinoma, Multiple myeloma, Schizophrenia, Alzheimer's disease, Cystic fibrosis, Ovarian neoplasm, Obesity, Type II diabetes mellitus, Rheumatoid arthritis	https://www.targetvalidation.org/target/ENSG00000010256/associations	UQCRC2 CYCS	Mitochondrion inner membrane,	Yes	https://www.drugbank.ca/biodb/polypeptides/P31930	✓
USF1	HUMAN Upstream stimulatory factor 1	bHLHb11, MLTFI, UEF	P22415	ENSG0000(	Transcription factor,Signalling	AK314876	Hyperlipidemia, Bilateral polymicrogyria, Hereditary palmoplantar keratoderma, Gamborg-Nielsen type, Autosomal dominant nocturnal frontal lobe epilepsy, Bilateral parasagittal parieto-occipital polymicrogyria, Type II diabetes mellitus, Obesity, Atherosclerosis, Metabolic syndrome X, Mixed hyperlipidemia	https://www.targetvalidation.org/target/ENSG00000158773/associations	MAPK14 PRKDC GSK3A ESR1 SUPT5H TNFAIP3 GTF2I USF2 TCFL5	Nucleus	No		✓
USF2	HUMAN Upstream stimulatory factor 2	bHLHb12, FIP	Q15853	ENSG0000(	Transcription factor	Y07661	Endometriosis, Inflammation, Alzheimer's disease, Chronic myeloid leukemia, Anoxia, Choledochal cyst, Biliary atresia, Breast neoplasm, Breast carcinoma, Mycoses	https://www.targetvalidation.org/target/ENSG00000105698/associations	EP300 GSK3B BRCA1 USF1 MAF PPARGC1A FOS	Nucleus	No		✓

USH1C	HUMAN Harmonin	AIE75, DFNB18, NY-CO-37, NY-CO-38, , PDZ73	Q9Y6N9	ENSG0000(	Cancer	AB018687	Retinal dystrophy, Usher syndrome type I, Non-syndromic genetic deafness, Autosomal recessive non-syndromic sensorineural deafness type DFNB, Usher syndrome type 1C, Retinitis pigmentosa, Congenital sensorineural hearing loss, Cognitive delay	https://www.targetvalidation.org/target/ENSG00000006611/associations	CDH23 ANKS4B USH1G USHBP1	Cytoplasm, cytosol	No		✓
USP10	HUMAN Ubiquitin carboxyl-terminal hydrolase 10	KIAA0190, UBPO	Q14694	ENSG0000(	Cancer,Cancer-Antigen	AL162049	Breast carcinoma, Mucocutaneous lymph node syndrome, Breast adenocarcinoma, Juvenile dermatomyositis, Glioblastoma, Colon tumor, Pancreatic carcinoma	https://www.targetvalidation.org/target/ENSG00000103194/associations	ATM G3BP1 CFTR AR	Cytoplasm	Yes	https://www.bindingdb.org/uniprot/Q14694	✓
UTP14A	HUMAN U3 small nucleolar RNA-associated protein 14 homolog A	NY-CO-16, SDCCAG16	Q9BVJ6	ENSG0000(	Cancer,Cancer-Antigen	AL034405	Colorectal cancer, Breast carcinoma, Ovarian carcinoma, Breast adenocarcinoma, Metastatic prostate cancer, Multiple myeloma, Psoriasis, Steinert myotonic dystrophy	https://www.targetvalidation.org/target/ENSG00000156697/associations	TP53 MPHOSPH10	Nucleus, nucleolus	No		✓
UXT	HUMAN Protein UXT	ART-27, STAP1	Q9UBK9	ENSG0000(	Transcription factor	AL009172	Teratozoospermia, Breast carcinoma, Prostate carcinoma, Aortic valve insufficiency, Androgen-insensitivity syndrome, X-linked mental retardation	https://www.targetvalidation.org/target/ENSG00000126756/associations	CECR2 MAP3K3 SARM1 RELA RCAN1 AR PIAS2 E2F6	Cytoplasm, cytoskeleton, microtubule organizing center, centrosome, Nucleus,	No		✓
VAV1	HUMAN Proto-oncogene vav	VAV	P15498	ENSG0000(	Cancer	AC020954	Primary immunodeficiency, Hyper-IgM syndrome with susceptibility to opportunistic infections, Severe combined immunodeficiency, Medulloblastoma, Lymphopenia, Adenocarcinoma, Neuroblastoma, Lung adenocarcinoma	https://www.targetvalidation.org/target/ENSG00000141968/associations	BLNK SHB PTK2B CBLB LCP2 MERTK BTK PRKCQ SH3BP2 RAC1 GRB2 HNRNPK CDC42 ARHGDIB NEK3 DNM2 CRK SYK ZAP70 TEC IL6ST TYK2 SHC1 PTPN6 MAPK1 PIK3R1 CBL ERBB3 PLCG1 SPI1 CD19 XRCC6 SRC BCR		Yes	https://www.bindingdb.org/uniprot/P15498	✓
VAX2	HUMAN Ventral anterior homeobox 2	DRES93	Q9UIW0	ENSG0000(	Transcription factor	BC006336	Atopic eczema, Astigmatism, Isolated anophthalmia-microphthalmia, Colobomatous microphthalmia, Congenital primary aphakia, Anophthalmos, Tongue squamous cell carcinoma	https://www.targetvalidation.org/target/ENSG00000116035/associations		Nucleus	No		✓
VCL	HUMAN Vinculin	VCL, MV	P18206	ENSG0000(	Signalling	AL731576, AL596247	Cardiomyopathy, Familial dilated cardiomyopathy, Hypertrophic cardiomyopathy, Osteoporosis, Dyspnea, Neuroendocrine tumor, Left ventricular noncompaction 10, Left ventricular noncompaction 10, Acute coronary syndrome	https://www.targetvalidation.org/target/ENSG00000035403/associations	TLN1 SORBS1 RAVER1 PTK2 PKD1 ACTA1 VASP PXN CTNNB1 CTNNA1 PRKCA IL7R SRC CDH1 ACTN1 ARPC2 ACTN1 CLTC SRC PXN EIF4E SORBS1 TLN1 PTK2 ACTA1	Cell membrane	No		✓
VDR	HUMAN Vitamin D3 receptor	NR1I1	P11473	ENSG0000(	Transcription factor	AB002168	Rickets, Hypocalcemic rickets, Osteoporosis, Alopecia, Secondary hyperparathyroidism, Vitamin D deficiency, Mammary neoplasm, Hypertensive disease	https://www.targetvalidation.org/target/ENSG00000111424/associations	CREBBP NCOA1 SNW1 EP300 ACVR1 SMAD3 CSNK2A1 PRKX CDK7 RXRB RXRA CSNK2A2 PRKACA PRKCA FGR PRKCB TP53 NCOR1 PRKY HR TRIM24 CTNNB1 SRC CYP3A4 CASP3 PLD1 STAT1 CYP2B6 JUN BAG1 PPARG	Nucleus	Yes	https://www.bindingdb.org/uniprot/P11473	✓
VEGFB	HUMAN Vascular endothelial growth factor B	VEGFL, VRF	P49765	ENSG0000(	Transcription factor,Cancer,Cytokine/Chemokine	U48801	Hemorrhage, Retinopathy, Diabetic retinopathy, Retinal vein occlusion, Pancreatic neoplasm, Atherosclerosis, Liver carcinoma, Chorioamnionitis, Familial long QT syndrome	https://www.targetvalidation.org/target/ENSG00000173511/associations	FLT1 VEGFA FLT1	Secreted	Yes	https://www.drugbank.ca/biodb/polypeptides/P49765	✓

VEGFD	HUMAN Vascular endothelial growth factor D	VEGF-D, VEGFD, FIGF	O43915	ENSG0000(	Transcription factor,Cancer, Cytokine/Chemokine	AJ000185	Lung carcinoma, Breast carcinoma, Lymphangioleiomyomatosis, Lung adenocarcinoma, Melanoma, Colorectal cancer, Non-small cell lung carcinoma, Gastric carcinoma, Renal glucosuria, Lymphedema	https://www.targetvalidation.org/target/ENSG00000165197/associations	KDR FLT4 KDR FLT4 ITGA9	Secreted	No		✓
VGLL1	HUMAN Transcription cofactor vestigial-like protein 1	TDU, TONDU	Q99990	ENSG0000(	Transcription factor	CH471150	Prostate carcinoma, Autoimmune disease, Trigeminal neuralgia, Breast carcinoma, Ductal carcinoma, Luminal A breast carcinoma, Psoriasis, Crohn's disease	https://www.targetvalidation.org/target/ENSG00000102243/associations		Nucleus	No		✓
VHL	HUMAN Von Hippel-Lindau disease tumor suppressor	pVHL	P40337	ENSG0000(	Cancer	AC034193	Kidney neoplasm, Von Hippel-Lindau Syndrome, Pheochromocytoma, Paraganglioma, Polycythemia, Adenoma, Kidney neoplasm, Chuvash erythrocytosis, Renal cell carcinoma	https://www.targetvalidation.org/target/ENSG00000134086/associations	CARD9 NEK1 ELOB ELOC CUL2 CSNK2A1 RACK1 RBX1 GSK3B CSNK1A1 POLR2A HNRNPA2B1 TGM2 CLU GLI1 SP1 FN1 CHEK2 STK16 KLF4 HIF1A PRKCZ PRKCD PIAS4 EPAS1 TUBA1A MKNK2 CDKN2A USP20 JADE1 CERKL	Cytoplasm, Membrane, Peripheral membrane protein, Nucleus,	Yes	https://www.bindingdb.org/uniprot/P40337	✓
VIM	HUMAN Vimentin	VIME	P08670	ENSG0000(	Cancer,Cancer-Antigen	Z19554	Non-syndromic congenital cataract, Pulverulent cataract, Nephrosis, Mammary neoplasm, Prostatic neoplasm, Synovial sarcoma, Malignant mesothelioma, Liver cirrhosis, Colonic neoplasm, Epithelial neoplasm	https://www.targetvalidation.org/target/ENSG00000026025/associations	RIPK3 CAMK2A PKP2 MAP3K3 AURKB PKN1 PLEC CASP8 PKP1 CAMK2D ROCK1 TRIM28 MAP3K1 PAK2 PAK1 CDK5 PRKDC PPP2CA PPP2CB CASP6 CASP9 CASP7 PLK1 MAPKAPK2 CASP3 AKT1 CDK2 PRKACA PRKCA	Cytoplasm	No		✓
VPS45	HUMAN Vacuolar protein sorting-associated protein 45	h-vps45, H1, VPS45A, VPS45B	Q9NRW7	ENSG0000(	Cancer,Cancer-Antigen	AK023170	Recurrent infections-myelofibrosis-nephromegaly syndrome, Severe congenital neutropenia, Schizophrenia, Amyotrophic lateral sclerosis, Hyperglobulinemia, Autosomal recessive predisposition, Tuberculosis, Medulloblastoma, Malignant mesothelioma, Primary myelofibrosis	https://www.targetvalidation.org/target/ENSG00000136631/associations	RBSN STX16	Golgi apparatus membrane	No		✓
VPS4A	HUMAN Vacuolar protein sorting-associated protein 4A	FLJ22197, SKD1, SKD1A, SKD2, VPS4, VPS4-1	Q9UN37	ENSG0000(	Others	AF159063	HIV infection, Familial partial lipodystrophy, Mandibuloacral dysplasia, Wolfram syndrome, Ataxia-telangiectasia, Hepatitis A infection, Hepatitis C infection, Mumps, Vesicular stomatitis, Congestive heart failure	https://www.targetvalidation.org/target/ENSG00000132612/associations	CHMP1A IST1 RND2 VPS4B NPC1 CHMP3 VTA1 CHMP6 CHMP1B	Prevacuolar compartment membrane, Peripheral membrane protein, Late endosome membrane Midbody,	No		✓
VPS54	HUMAN Vacuolar protein sorting-associated protein 54	HCC8	Q9P1Q0	ENSG0000(	Others	AL359939	Amyotrophic lateral sclerosis type 4, Spinal muscular atrophy with respiratory distress type 1, Proximal spinal muscular atrophy, Hereditary motor and sensory neuropathy, Okinawa type, Post-traumatic stress disorder, HMN proximal type I, Cholera, Motor neuron atrophy, Sepsis, Ovarian carcinoma	https://www.targetvalidation.org/target/ENSG00000143952/associations	VPS52	Golgi apparatus, trans-Golgi network	No		✓
VPS72	HUMAN Vacuolar protein sorting-associated protein 72 homolog	Swc2, TCFL1, YL-1, YL1	Q15906	ENSG0000(	Transcription factor	AK222935	Malignant Mesothelioma, Bardet-Biedl syndrome, Melanoma, Sarcoma, Luminal B breast carcinoma	https://www.targetvalidation.org/target/ENSG00000163159/associations	RUVBL1 CAMK2A DMAP1 CAMK4 CAMK2D PRKCE CSNK2A1 MAPK1 CSNK2A2 PRKCA PRKCB PRKCG YEATS4	Nucleus	No		✓
VRK3	HUMAN Inactive serine/threonine-protein kinase VRK3	VRK3	Q8IV63	ENSG0000(	Kinase	AK303010	Neuroblastoma, Lung carcinoma, Breast adenocarcinoma, Psoriasis, Parkinson's disease, Colorectal adenocarcinoma, Schizophrenia, Neurodegenerative disease	https://www.targetvalidation.org/target/ENSG00000105053/associations	CTNNB1 CDK5 APC BID TP53 YWHAQ CSNK1E TLR9 PSEN2 NFATC3 SLC18A2 NOL7 EZH1	Nucleus	No		✓

WAS	HUMAN Wiskott-Aldrich syndrome protein	IMD2, WASP	P42768	ENSG0000(	Cancer	AF115549	Primary immunodeficiency, Wiskott-Aldrich syndrome, Hereditary thrombocytopenia with normal platelets, X-linked thrombocytopenia with normal platelets, Eczema, Inflammatory bowel disease, Autoimmune disease	https://www.targetvalidation.org/target/ENSG00000015285/associations	LCP2 ITK TNK2 BTK PTPN12 CSNK2A1 RAC1 GRB2 CDC42 VASP CRKL TEC MTOR C5AR1 CSNK2A2 PLCG2 NCK1 HCLS1 SRC FGR HCK LYN FYN LCK ABL1 DAB1 FYB SRC RANBP2 KPNA2 RANGAP1 PSTPIP1 ARPC1B CDC42 GRB2 NCK1	Cytoplasm, cytoskeleton,		Yes	https://www.drugbank.ca/biodb/polypeptides/P42768	✓
WDR45B	HUMAN WD repeat domain phosphoinositide-interacting protein 3	WDR45L, WIPI3	Q5MNZ6	ENSG0000(	Cancer,Cancer-Antigen	AY691427	Breast carcinoma, Glioblastoma multiforme, Pancreatic carcinoma	https://www.targetvalidation.org/target/ENSG00000141580/associations				No		✓
WDR46	HUMAN WD repeat-containing protein 46	BING4, C6orf11, UTP7	O15213	ENSG0000(	Cancer,Cancer-Antigen	AL844527, BX000343	Diffuse large B-cell lymphoma, Respiratory system disease, Breast carcinoma, Gastric neoplasm, Systemic lupus erythematosus, Melanoma	https://www.targetvalidation.org/target/ENSG00000227057/associations		Nucleus, nucleolus		No		✓
WISP3	HUMAN WNT1-inducible-signaling pathway protein 3	CCN6, UNQ462	O95389	ENSG0000(	Others	Z99289,AL512299	Progressive pseudorheumatoid arthropathy of childhood, Arthropathy, Degenerative polyarthritis, Joint stiffness, Platyspondyly, Prostate carcinoma, Tongue squamous cell carcinoma, Osteosarcoma, Breast carcinoma	https://www.targetvalidation.org/target/ENSG00000112761/associations		Secreted		No		✓
WT1	HUMAN Wilms tumor protein	WT33	P19544	ENSG0000(	Transcription factor,Cancer	AK093168	Denys-Drash syndrome, Frasier syndrome, Nephroblastoma, Congenital diaphragmatic hernia, WAGR syndrome, Malaignant mesothelioma, Meacham syndrome, Nephrotic syndrome type 4, Acute myelocytic leukemia	https://www.targetvalidation.org/target/ENSG00000184937/associations	PAWR AMER1 PRKX PRKACA AREG EGFR PRKY TP53 PAX2 CREBBP UBE2I FHL2 SRY RBM4 U2AF2 WTAP	Nucleus		No		✓
WWOX	HUMAN WW domain-containing oxidoreductase	FOR, SDR41C1, WOX1	Q9NZC7	ENSG0000(	Cancer	AF227526	Autosomal recessive cerebellar ataxia, Autosomal recessive cerebellar ataxia-epilepsy-intellectual disability syndrome due to WWOX deficiency, Early infantile epileptic encephalopathy, Squamous cell carcinoma, Epilepsy, Seizures, Epileptic encephalopathy, Gastric neoplasm	https://www.targetvalidation.org/target/ENSG00000186153/associations	SAMD5 ERBB4 ATM TNK2 MAP2K6 MAPK8 TGFBR1 SRC TP53 SMAD4 TP73	Cytoplasm		No		✓
WWTR1	HUMAN WW domain-containing transcription regulator protein 1	DKFZp586I1419, TAZ	Q9GZV5	ENSG0000(	Kinase,Transcription factor,Cancer	CH471052	Adenocarcinoma, Colonic neoplasm, Melanoma, Epithelioid hemangioendothelioma, Muscular atrophy, Atherosclerosis, Breast carcinoma, Gastric adenocarcinoma, Bile duct carcinoma, Bladder carcinoma	https://www.targetvalidation.org/target/ENSG00000018408/associations	TJP2 LATS2 NEK1 CCAR1 AMOT STK3 BTK YWHAE CSNK1E CSNK1D CDK1 LATS1 MYOD1 PAX3 SMAD3 PAX8 SMAD2 PPARG NKX2-1 DVL1 RUNX2 TEAD3 TEAD1 TTF1 TEAD2 TEAD4	Nucleus, Cytoplasm,		No		✓
XAGE1A	HUMAN X antigen family member 1	GAGED2, XAGE-1, XAGE1A, XAGE1B, XAGE1C, XAGE1D, XAGE1E	Q9HD64	ENSG0000(	Others	AJ318878	Melanoma, Lung neoplasm, Adenoid cystic carcinoma, Ewings sarcoma, Lung adenocarcinoma, Prostate neoplasm, Alveolar rhabdomyosarcoma	https://www.targetvalidation.org/target/ENSG00000204382/associations				No		✓
XAGE2	HUMAN X antigen family member 2	CT12.2, GAGED3, XAGE-2, XAGE2B	Q96GT9	ENSG0000(	Others	AL953874	Ovarian carcinoma, Breast carcinoma, Tongue squamous cell carcinoma, Hepatocellular carcinoma, Endometrial neoplasm, Rhabdomyosarcoma, Germ cell tumor	https://www.targetvalidation.org/target/ENSG00000155622/associations				No		✓
XAGE4	HUMAN XAGE-4 protein	XAGE4	Q8WWM0	ENSG0000(	Others	AL159987.19	NA	NA	NA			NA		✓
XBP1	HUMAN X-box-binding protein 1	XBP2	P17861	ENSG0000(	Transcription factor,Cancer	AB076383	Inborn errors of metabolism, Congenital dyserythropoietic anemia, Hereditary persistence of fetal hemoglobin-Beta-Thalassemia, Schzophrenia, Bipolat disorderm, Glioma, Fatty liver	https://www.targetvalidation.org/target/ENSG00000100219/associations	MAPK1 MAPK3 ESR1 FOS ERN1 BECN1 UBE2I	Endoplasmic reticulum	Transmembrane	Yes	https://www.bindingdb.org/uniprot/P17861	✓

XIAP	HUMAN E3 ubiquitin-protein ligase XIAP	API3, BIRC4, hILP	P98170	ENSG0000(	Cancer	AL121601	Primary immunodeficiency, Lymphoproliferative syndrome, X-linked lymphoproliferative disease, Lymphoma, Hypogammaglobulinemia, Meningitis, Ototoxicity, Osteosarcoma, Desminopathy	https://www.targetvalidation.org/target/ENSG00000101966/associations	TAB2<br>DIABLO<br>TOLLIP<br>STRADB<br>CAMKK1<br>XAF1<br>TAB1<br>PDK1<br>IKBKE<br>BIRC2<br>BIRC3<br>EIF4G2<br>RAC1<br>CASP9<br>ARHGDIA<br>CASP3<br>ACVRL1<br>TGFBR1<br>ACVR1B<br>AKT2<br>AKT1 UBC<br>HNRNPA1<br>APP<br>CAPNS1<br>RAF1<br>HTRA2<br>RIPK2<br>MAP3K7<br>SEPT4	Cytoplasm, Nucleus,	Yes	https://www.bindingdb.org/uniprot/P98170	✓
XYLB	HUMAN Xylulose kinase	FLJ10343, FLJ12539	O75191	ENSG0000(	Kinase	AP006193	Schizophrenia, Gaucher disease, Autosomal recessive hypophosphatemic rickets, Breast carcinoma, Ischemic stroke, Ischemic cerebrovascular accident, Kenney-Caffey syndrome, Pycnodysostosis, CHARGE syndrome, Sanjad-Sakati syndrome	https://www.targetvalidation.org/target/ENSG00000093217/associations			No		✓
YARS	HUMAN Tyrosine--tRNA ligase, cytoplasmic	tyrRS	P54577	ENSG0000(	Cancer,Cancer-Antigen	AK223608	Charcot-Marie-Tooth disease, Autosomal dominant intermediate Charcot-Marie-Tooth disease type C cancer, Lung carcinoma, Large cell lung carcinoma, Distal amyotrophy, Axonal regeneration, Neuropathy, Dermatomyositis, Adenocarcinoma, Neuroblastoma	https://www.targetvalidation.org/target/ENSG00000134684/associations	ELANE	Cytoplasm	Yes	https://www.bindingdb.org/uniprot/P54577	✓
YEATS4	HUMAN YEATS domain-containing protein 4	GAS41, NuBI-1	O95619	ENSG0000(	Transcription factor	BC000994	Glioma, Astrocytoma, Venous thromboembolism, Liposarcoma, Glioblastoma multiforme, Posterior cortical atrophy, Ovarian carcinoma, Colorectal cancer	https://www.targetvalidation.org/target/ENSG00000127337/associations	TRRAP<br>RUVBL1<br>RUVBL2<br>MORF4L1<br>ING3<br>MRGBP<br>DMAP1<br>BRD8<br>VPS72<br>EP400<br>NUMA1<br>TFAP2B<br>EPC1	Nucleus	No		✓
YTHDF2	HUMAN YTH domain-containing family protein 2	HGRG8, NY-REN-2	Q9Y5A9	ENSG0000(	Cancer,Cancer-Antigen	AK292581	Hepatocellular carcinoma, HIV-1 infection	https://www.targetvalidation.org/target/ENSG00000198492/associations		Cytoplasm, cytosol	No		✓
YWHAE	HUMAN 14-3-3 protein epsilon	14-3-3E	P62258	ENSG0000(	Cancer,Cancer-Antigen	U43430	Melanoma, Kidney neoplasm, Colorectal adenocarcinoma, Miller Dieker syndrome, Schizophrenia, Endometrial stromal sarcoma, Lissencephaly, Bipolar disorder, Cerebral atrophy, Ovarian serous adenocarcinoma	https://www.targetvalidation.org/target/ENSG00000108953/associations	RIPK3<br>MAP3K2<br>TBK1<br>PLK2<br>PLK3<br>MAP3K3<br>MAP3K5<br>TSC1<br>DYRK1A<br>MAP3K1<br>YWHAH<br>MST1R<br>MAP3K10<br>CDK16<br>YWHAZ<br>CALM1<br>YWHAG<br>MLF1<br>PAPOLA<br>TSC2<br>CASP3<br>USP8<br>YWHAB<br>CDC25B<br>PRDX6<br>YWHAQ<br>RPS6KB1<br>CDK11B<br>PRKACA	Nucleus	Yes	https://www.drugbank.ca/biodb/polypeptides/P62258	✓
YWHAG	HUMAN 14-3-3 protein gamma	KCIP-1	P61981	ENSG0000(	Others	CR541925	Multiple sclerosis, Breast carcinoma, Pancreatic ductal adenocarcinoma, Glioblastoma multiforme, Schizophrenia, Down syndrome, Fibroid tumor, Morbid obesity, Lung adenocarcinoma	https://www.targetvalidation.org/target/ENSG00000170027/associations	MAP3K2<br>MAP3K20<br>LATS2<br>CENPJ<br>WNK1<br>MAP3K3<br>MAP3K5<br>SRPK1<br>TSC1<br>CAMKK1<br>RPTOR<br>CLASP1<br>DYRK1A<br>PRPF4B<br>PAK1<br>FOXO1<br>PRKCD<br>YWHAH<br>PRKCQ<br>CDK17<br>CDK16<br>PRKDC<br>TUBA4A<br>YWHAZ<br>YWHAE<br>GSK3A<br>TSC2<br>CLK3<br>CLK2	Cytoplasm	Yes	https://www.bindingdb.org/uniprot/P61981	✓
ZAP70	HUMAN Tyrosine-protein kinase ZAP-70	SRK, ZAP-70	P43403	ENSG0000(	Kinase	BC039039	Primary immunodeficiency, Combined immunodeficiency due to ZAP70 deficiency autoimmune disease, Severe combined immunodeficiency, X-Linked combined immunodeficiency disease, ZAP70 deficiency, Eczema, Splenomegaly, Autosomal recessive predisposition, Panhypogammaglobulinemia	https://www.targetvalidation.org/target/ENSG00000115085/associations	PTPN22<br>DBNL<br>DEF6<br>NFAM1<br>PTCRA<br>MAPK14<br>SHB SLA<br>CBLB<br>SOS1<br>TYRO3<br>SH3BP2<br>GRB2<br>CXCR4<br>DUSP3<br>PIK3CG<br>CRKL CRK<br>SYK<br>BMPR1A<br>SHC1<br>PTPN6<br>EPHA3<br>ACP1 CBL<br>ERBB3<br>CD247<br>RASA1<br>PLCG1<br>MUC1<br>SRC<br>CD3G	Cytoplasm	Yes	https://www.bindingdb.org/uniprot/P43403	✓
ZBTB16	HUMAN Zinc finger and BTB domain-containing protein 16	PLZF, ZNF145	Q05516	ENSG0000(	Kinase,Transcription factor,Cancer	BC029812	Adenocarcinoma, Lung carcinoma, Acute promyelocytic leukemia, Strabismus, Unipolar depression, Fibular hypoplasia, Autosomal recessive predisposition, Prostate carcinoma	https://www.targetvalidation.org/target/ENSG00000109906/associations	TAB2<br>MAP3K3<br>LMTK3<br>EP300<br>SUMO1<br>AGTR2<br>MAPK8<br>ATP7B<br>BMI1 PML<br>CDK2<br>RXRA<br>GATA1<br>GNAO1<br>CDK1<br>TIMP1<br>ATP6AP2<br>ID2<br>RUNX1T1<br>IL3RA<br>CEBPA<br>CCNA2<br>GFI1<br>DUSP6<br>MYC<br>HBEGF<br>RNF112	Nucleus	No		✓

ZBTB25	HUMAN Zinc finger and BTB domain-containing protein 25	C14orf51, KUP, ZNF46	P24278	ENSG0000(	Transcription factor	BC035804	Breast carcinoma,Squamous cell carcinoma, Nijmegen breakage syndrome, Ovarian carcinoma, Pancreatic carcinoma, Multiple myeloma, Glioblastoma multiforme	https://www.targetvalidation.org/target/ENSG00000089775/associations	MAPK9	Nucleus	No	✓
ZBTB32	HUMAN Zinc finger and BTB domain-containing protein 32	FAZF, TZFP, ZNF538	Q9Y2Y4	ENSG0000(	Transcription factor	AF130255	Azoospermia, Cervical carcinoma, Fanconi anemia, Neoplasm of mature B-cells, Breast carcinoma, Oropharyngeal carcinoma, Promyelocytic leukemia	https://www.targetvalidation.org/target/ENSG00000011590/associations	FANCC	Nucleus	No	✓
ZBTB7B	HUMAN Zinc finger and BTB domain-containing protein 7B	c-Krox, hcKrox, ZBTB15, ZFP67, Znf857B, ZBT7B	O15156	ENSG0000(	Transcription factor	AL451085	X-linked immunodeficiency with magnesium defect, Epstein-Barr virus infection and neoplasia, Schizophrenia, Lung fibrosis - immunodeficiency - 46-XX gonadal dysgenesis, Severe combined immunodeficiency, Wiskott-Aldrich syndrome, Melanoma, Crohn's disease	https://www.targetvalidation.org/target/ENSG00000160685/associations	EP300	Nucleus	No	✓
ZC4H2	HUMAN Zinc finger C4H2 domain-containing protein	HCA127, KIAA1166, WWS	Q9NQZ6	ENSG0000(	Cancer,Cancer-Antigen	AK022918	Arthrogryposis multiplex congenita, Intellectual disability-developmental delay-contractures syndrome, Li-Fraumeni syndrome, Tongue squamous cell carcinoma, Wieacker-Wolff syndrome, Arthrogryposis, Walker-Warburg congenital muscular dystrophy, Cerebral Palsy, Paresis	https://www.targetvalidation.org/target/ENSG00000126970/associations		Cytoplasm	No	✓
ZFP36L1	HUMAN mRNA decay activator protein ZFP36L1	Berg36, BRF1, ERF1, RNF162B, TIS11B	Q07352	ENSG0000(	Transcription factor,Cancer	BC018340	Autoimmune disease, Type I diabetes mellitus, Inflammatory bowel disease, Multiple sclerosis, Breast carcinoma, Adenocarcinoma, Epithelial ovarian cancer, Varicose ulcer, Acute lymphocytic leukemia, Psoriasis	https://www.targetvalidation.org/target/ENSG00000185650/associations	MAPK14 PDK1 MAPKAPK2 AKT2 AKT1 MAPK3 MAPK14	Nucleus	No	✓
ZHX2	HUMAN Zinc fingers and homeoboxes protein 2	KIAA0854	Q9Y6X8	ENSG0000(	Transcription factor	NA	Atherosclerosis, Type I diabetes mellitus, Tuberculosis, Melanoma, Colorectal cancer, Liver carcinoma, Thyroid carcinoma, Hepatocellular carcinoma	https://www.targetvalidation.org/target/ENSG00000178764/associations	ZHX3 CDC123	Nucleus	No	✓
ZKSCAN1	HUMAN Zinc finger protein with KRAB and SCAN domains 1	KOX18, ZNF139, ZNF36	P17029	ENSG0000(	Transcription factor	CH471091	Melanoma, Gastric carcinoma, Lung carcinoma, Lung adenocarcinoma, Juvenile dermatomyositis, Psoriasis, Liver cirrhosis	https://www.targetvalidation.org/target/ENSG00000106261/associations		Nucleus	No	✓
ZKSCAN3	HUMAN Zinc finger protein with KRAB and SCAN domains 3	ZPF47, Zfp47, ZNF306, ZNF309, ZSCAN13	Q9BRR0	ENSG0000(	Transcription factor	AL358785, AL021997	Bladder carcinoma, Colorectal cancer, Spinobulbar muscular atrophy, Prostate carcinoma, Colon tumor, Multiple myeloma	https://www.targetvalidation.org/target/ENSG00000189298/associations	ULK1 MAP1LC3B WIPI2	Nucleus, Cytoplasm,	No	✓
ZKSCAN4	HUMAN Zinc finger protein with KRAB and SCAN domains 4	p373c6.1, ZNF307, ZNF427	Q969J2	ENSG0000(	Transcription factor	CH471081	Breast carcinoma, Cardiac hypertrophy, Hepatocellular carcinoma, Fibrosis, Schizophrenia, Leukemia, Dilated cardiaomyopathy	https://www.targetvalidation.org/target/ENSG00000187626/associations		Nucleus	No	✓
ZKSCAN5	HUMAN Zinc finger protein with KRAB and SCAN domains 5	KIAA1015, ZFP95	Q9Y2L8	ENSG0000(	Transcription factor	CH471091	Medulloblastoma, Psoriasis, Lung carcinoma	https://www.targetvalidation.org/target/ENSG00000196652/associations		Nucleus	No	✓
ZMAT2	HUMAN Zinc finger matrin-type protein 2	FLJ31121, hSNU23, Snu23	Q96NC0	ENSG0000(	Transcription factor	NA	Breast carcinoma, Prostate carcinoma	https://www.targetvalidation.org/target/ENSG00000146007/associations		Nucleus	No	✓
ZMAT3	HUMAN Zinc finger matrin-type protein 3	FLJ12296, MGC10613, PAG608, WIG-1, WIG1	Q9HA38	ENSG0000(	Transcription factor,Signalling,Cancer	AK122768	Post-traumatic stress disorder, Melanoma, Acute lymphoblastic leukemia, Nerve degeneration, Adenocarcinoma, Colon carcinoma, Cervix carcinoma, Esophageal carcinoma, Lung carcinoma	https://www.targetvalidation.org/target/ENSG00000172667/associations	AGO2	Nucleus	No	✓

ZMYM2	HUMAN Zinc finger MYM-type protein 2	FIM, MYM, RAMP, ZNF198	Q9UBW7	ENSG0000(	Kinase,Transcription factor,Cancer	AL136621	Lymphoid neoplasm, Leukemia, Acute lymphoblastic leukemia, Myeloproliferative disease, Trichohepatoenteric syndrome, Hepatitis B infection, Gastric carcinoma, X-linked mental retardation, Chronic myeloproliferative disorder	https://www.targetvalidation.org/target/ENSG00000121741/associations	PLK1 PML FGFR1	Nucleus	No	✓
ZMYND11	HUMAN Zinc finger MYND domain-containing protein 11	BS69, ZMY11	Q15326	ENSG0000(	Others	DQ335454	Autosomal dominant non-syndromic intellectual disability, Mental retardation, Azoospermia, Autistic disorder, Dysmorphic facies, Atopic eczema, Cervical carcinoma, Psoriasis	https://www.targetvalidation.org/target/ENSG00000015171/associations	TRAF6 TICAM1 TAB1 H3F3A; H3F3B BMPR1A ETS2 MYB NCOR1 HIST3H3 TRAF6 MGA PDLIM7	Nucleus	No	✓
ZMYND8	HUMAN Protein kinase C-binding protein 1	PRKCBP1, RACK7, KIAA1125	Q9ULU4	ENSG0000(	Kinase,Cancer	AF273045	Bartter syndrome, Familial hypocalciuric hypercalcemia, Familial idiopathic steroid-resistant nephrotic syndrome, Acute erythroblastic leukemia, Prostate carcinoma, Kenny-Caffey syndrome	https://www.targetvalidation.org/target/ENSG00000101040/associations	PRKCB FHOD1	Nucleus	No	✓
ZNF136	HUMAN Zinc finger protein 136	ZNF136	P52737	ENSG0000(	Transcription factor	U09367	Breast carcinoma, Adenocarcinoma, Breast adenocarcinoma, Melanoma, Pancreatic carcinoma, Psoriasis	https://www.targetvalidation.org/target/ENSG00000196646/associations		Nucleus	No	✓
ZNF165	HUMAN Zinc finger protein 165	CT53, ZSCAN7, ZPF165	P49910	ENSG0000(	Transcription factor	AL121944	Azoospermia, Lung carcinoma, Bladder neoplasm, Transitional cell carcinoma, Benign prostatic hyperplasia, Tarsal-Carpal coalition syndrome, Metastatic prostate cancer	https://www.targetvalidation.org/target/ENSG00000197279/associations		Nucleus	No	✓
ZNF169	HUMAN Zinc finger protein 169	ZNF169	Q14929	ENSG0000(	Transcription factor	BC047702	Breast carcinoma, Multiple keratoacanthoma, Ferguson-Smith type, Lung carcinoma, Multiple self-healing squamous epithelioma	https://www.targetvalidation.org/target/ENSG00000175787/associations		Nucleus	No	✓
ZNF174	HUMAN Zinc finger protein 174	ZSCAN8, ZN174	Q15697	ENSG0000(	Transcription factor	BC001161	Breast carcinoma, Melanoma, Lung adenocarcinoma, Small cell lung carcinoma, Wiskott-Aldrich syndrome, Glioblastoma multiforme	https://www.targetvalidation.org/target/ENSG00000103343/associations		Nucleus	No	✓
ZNF19	HUMAN Zinc finger protein 19	KOX12, MGC51021	P17023	ENSG0000(	Transcription factor	AC010547	Breast carcinoma, Melanoma	https://www.targetvalidation.org/target/ENSG00000157429/associations		Nucleus	No	✓
ZNF202	HUMAN Zinc finger protein 202	ZKSCAN10	O95125	ENSG0000(	Transcription factor	DQ093962	Metabolic disease, Autosomal recessive hypophosphatemic rickets, Achondrogenesis, Spondylo-megaepiphyseal-metaphyseal dysplasia, Atherosclerosis, Myocardial infarction, Myocardial ischemia, Arteriosclerosis, Dyslipidemia, Diaphanospondylodysostosis	https://www.targetvalidation.org/target/ENSG00000166261/associations	SCAND1	Nucleus	No	✓
ZNF207	HUMAN BUB3-interacting and GLEBS motif-containing protein ZNF207	ZN207, BUGZ	O43670	ENSG0000(	Transcription factor	AK226172	Lung carcinoma, Large cell lung carcinoma, Maple syrup urine disease, Brain neoplasm, Glioblastoma multiforme, Pick disease, Colorectal adenocarcinoma, Jeune syndrome	https://www.targetvalidation.org/target/ENSG00000010244/associations		Nucleus	No	✓
ZNF256	HUMAN Zinc finger protein 256	BMZF-3	Q9Y2P7	ENSG0000(	Transcription factor	AC008969	Ovarian carcinoma, Melanoma, Psoriasis, Hepatocellular carcinoma, Rosacea, Glioblastoma multiforme, Endometrial neoplasm	https://www.targetvalidation.org/target/ENSG00000152454/associations		Nucleus	No	✓

ZNF274	HUMAN Neurotroph in receptor-interacting factor homolog	ZKSCAN1 9, SP2114	Q96GC6	ENSG0000(	Transcripti on factor	AF275681	Normosmic congenital hypogonadotropic hypogonadism, Kallmann syndrome, Glioblastoma multiforme, Breast carcinoma, Nerve degeneration, Prader-Willi Syndrome, Pancreatic carcinoma, Melanoma	https://www.targetvalidation.org/target/ENSG00000171606/associations	TRIM28 NGFR NGFR	Cytoplasm	No		✓
ZNF276	HUMAN Zinc finger protein 276	CENP-Z, CENPZ, ZFP276, ZNF477	Q8N554	ENSG0000(	Transcripti on factor	AC010538	Breast carcinoma, Hepatitis C infection	https://www.targetvalidation.org/target/ENSG00000158805/associations		Nucleus	No		✓
ZNF277	HUMAN Zinc finger protein 277	NRIF4, ZNF277P	Q9NRM2	ENSG0000(	Transcripti on factor	AC004111	Parental longevity, Glioblastoma multiforme, T-cell acute lymphoblastic leukemia, Autistic disorder, Colon carcinoma, Melanoma, Atopic eczema, Medulloblastoma	https://www.targetvalidation.org/target/ENSG00000198839/associations		Nucleus	No		✓
ZNF282	HUMAN Zinc finger protein 282	HUB1	Q9UDV7	ENSG0000(	Transcripti on factor	AC004890	Breast carcinoma, Squamous cell carcinoma, Esophageal squamous cell carcinoma, T-cell leukemia, Nijmegen breakage syndrome	https://www.targetvalidation.org/target/ENSG00000170265/associations		Nucleus	No		✓
ZNF3	HUMAN Zinc finger protein 3	A8-51, FLJ20216, HF.12, KOX25, PP838, Zfp113	P17036	ENSG0000(	Transcripti on factor	AK297625	Osteosarcoma, Breast carcinoma, Atopic eczema, Medulloblastoma, Melanoma, Empheysema	https://www.targetvalidation.org/target/ENSG00000166526/associations		Nucleus	No		✓
ZNF323	HUMAN Zinc finger and SCAN domain-containing protein 31	ZNF310P, ZSCAN31	Q96LW9	ENSG0000(	Transcripti on factor	CH471081	Schizophrenia, Psychotic disorder, Nonorganic psychosis, Breast carcinoma, Lung adenocarcinoma, Unipolar depression	https://www.targetvalidation.org/target/ENSG00000235109/associations		Nucleus	No		✓
ZNF331	HUMAN Zinc finger protein 331	RITA, ZNF361, ZNF463	Q9NQX6	ENSG0000(	Transcripti on factor,Canc er	AF272148	Melanoma, Lung carcinoma, Dermatologic disorder, Thyroid neoplasm, Breast carcinoma, Liver carcinoma, Sarcoma, Basal cell carcinoma, Oral cavity cancer, Cutaneous melanoma	https://www.targetvalidation.org/target/ENSG00000130844/associations		Nucleus	No		✓
ZNF346	HUMAN Zinc finger protein 346	JAZ	Q9UL40	ENSG0000(	Transcripti on factor	CR749692	Small cell lung carcinoma, Benign prostatic hyperplasia, Colorectal cancer, Melanoma, Meningococcal infection, Glioblastoma multiforme, Potassium deficiency	https://www.targetvalidation.org/target/ENSG00000113761/associations	XPO5	Nucleus, nucleolus	No		✓
ZNF35	HUMAN Zinc finger protein 35	HF.10, HF10, Zfp105	P13682	ENSG0000(	Transcripti on factor	AC099669	Squamous cell carcinoma, Pancreatic carcinoma, Renal carcinoma, Non-small cell lung carcinoma, Cervical adenocarcinoma, Herpes simplex infection, Breast carcinoma, Pancreatic carcinoma	https://www.targetvalidation.org/target/ENSG00000169981/associations		Nucleus	No		✓
ZNF354A	HUMAN Zinc finger protein 354A	EZNF, HKL1, KID-1, KID1, TCF17	O60765	ENSG0000(	Transcripti on factor	AF116030	Cystic fibrosis, Germ cell tumor, Breast carcinoma, Kidney neoplasm, Seminoma, Testicular neoplasm, Embryonal carcinoma, Lung carcinoma	https://www.targetvalidation.org/target/ENSG00000169131/associations	TRIM28 HSPA7	Nucleus	No		✓
ZNF37A	HUMAN Zinc finger protein 37A	KOX21, ZNF37	P17032	ENSG0000(	Transcripti on factor	CH471072	Acute lymphoblastic leukemia, Prostate carcinoma, T-cell acute lymphoblastic leukemia, Ovarian carcinoma, Gastric carcinoma, Gaucher disease type 1, Steinert myotonic dystrophy	https://www.targetvalidation.org/target/ENSG00000075407/associations		Nucleus	No		✓
ZNF384	HUMAN Zinc finger protein 384	CAGH1, CIZ, NMP4, TNRC1	Q8TF68	ENSG0000(	Transcripti on factor,Canc er	CH471116	Melanoma, Lymphoid neoplasm, Head and Neck squamous cell carcinoma, Acute leukemia, Osteoporosis, Osteopenia, Prostate carcinoma, Basal cell carcinoma, Bladder carcinoma, Oral squamous cell carcinoma	https://www.targetvalidation.org/target/ENSG00000126746/associations	TAF15	Nucleus	No		✓

ZNF394	HUMAN Zinc finger protein 394	ZKSCAN14	Q53GI3	ENSG0000	Transcription factor	AC073063	Melanoma, Lung carcinoma, Acute myeloid leukemia, Pancreatic carcinoma, Breast carcinoma, Meningococcal infection	https://www.targetvalidation.org/target/ENSG00000160908/associations		Nucleus	No	✓
ZNF398	HUMAN Zinc finger protein 398	KIAA1339, ZER6	Q8TD17	ENSG0000	Transcription factor	AK304603	Breast carcinoma, Lung carcinoma, Thrombocytopenia, Adenocarcinoma, Spina bifida, Liver carcinoma, Ovarian carcinoma, Hepatocarcinogenesis, Meningococcal infection	https://www.targetvalidation.org/target/ENSG00000197024/associations	ESR1	Nucleus	No	✓
ZNF41	HUMAN Zinc finger protein 41	ZNF41	P51814	ENSG0000	Transcription factor	AJ010019	Mental retardation, Mental or behavioural disorder, Cognitive disorder, Lung adenocarcinoma, Synovial sarcoma, Colorectal adenocarcinoma, Diffuse large B-cell lymphoma	https://www.targetvalidation.org/target/ENSG00000147124/associations		Nucleus	No	✓
ZNF444	HUMAN Zinc finger protein 444	EZF2, ZSCAN17	Q8N0Y2	ENSG0000	Transcription factor	AK074063	Breast carcinoma, polyp, Peutz-Jeghers syndrome, Juvenile dermatomyositis, Malignant myoepithelioma	https://www.targetvalidation.org/target/ENSG00000167685/associations		Nucleus	No	✓
ZNF449	HUMAN Zinc finger protein 449	ZSCAN19	Q6P9G9	ENSG0000	Transcription factor	AL450472	Breast carcinoma, Large cell lung carcinoma, Colorectal cancer, Breast adenocarcinoma	https://www.targetvalidation.org/target/ENSG00000173275/associations		Nucleus	No	✓
ZNF496	HUMAN Zinc finger protein 496	ZKSCAN17	Q96IT1	ENSG0000	Transcription factor	AC104335	Multiple sclerosis, Squamous cell lung carcinoma, Sepsis, Melanoma, Thrombocytopenia	https://www.targetvalidation.org/target/ENSG00000162714/associations	NEK4 NSD1	Nucleus	No	✓
ZNF593	HUMAN Zinc finger protein 593	ZT86	O00488	ENSG0000	Transcription factor	BC002580	Medulloblastoma, Breast carcinoma, Glioblastoma multiforme, Steinert myotonic dystrophy, Colorectal adenocarcinoma, Gastric neoplasm	https://www.targetvalidation.org/target/ENSG00000142684/associations		Nucleus, nucleolus	No	✓
ZNF645	HUMAN E3 ubiquitin-protein ligase ZNF645	CBLL2	Q8N7E2	ENSG0000	Others	BC074910	Azoospermia, Cervical carcinoma, Parkinson's disease, Fanconi anemia, Von Hippel-Lindau syndrome, Breast carcinoma, Prostate carcinoma	https://www.targetvalidation.org/target/ENSG00000175809/associations		Cytoplasm		✓
ZNF70	HUMAN Zinc finger protein 70	Cos17, MGC48959	Q9UC06	ENSG0000	Transcription factor	X60077	Squamous cell lung carcinoma, Lung adenocarcinoma, Colorectal adenocarcinom, Prostate adenocarcinoma	https://www.targetvalidation.org/target/ENSG00000187792/associations		Nucleus	No	✓
ZNHIT3	HUMAN Zinc finger HIT domain-containing protein 3	TRIP3	Q15649	ENSG0000	Others	CH471199	Melanoma, Acute lymphoblastic leukemia, Glioma, Ovarian carcinoma, Precursor cell lymphoblastic leukemia lymphoma, Juvenile dermatomyositis, MODY, Acute myeloid leukemia	https://www.targetvalidation.org/target/ENSG00000273611/associations	HNF4A NUFIP1		No	✓
ZNRD1	HUMAN DNA-directed RNA polymerase I subunit RPA12	RPA12	Q9P1U0	ENSG0000	Transcription factor	CR457109	Graves disease, HIV infection, HIV-1 infection, Gastric neoplasm, Systemic lupus erythematosus, Spontaneous abortion, Esophageal neoplasm, Asthma, Leukemia, Lung carcinoma	https://www.targetvalidation.org/target/ENSG00000066379/associations	RPA2	Nucleus, nucleolus	No	✓
ZSCAN12	HUMAN Zinc finger and SCAN domain-containing protein 12	KIAA0426, ZNF305, ZNF96	O43309	ENSG0000	Transcription factor	Z98745	Sepsis, Prostate carcinoma, Melanoma	https://www.targetvalidation.org/target/ENSG00000158691/associations		Nucleus	No	✓
ZSCAN16	HUMAN Zinc finger and SCAN domain-containing protein 16	ZNF392, ZNF435	Q9H4T2	ENSG0000	Transcription factor	AL358933	Li-Fraumeni syndrome, Breast carcinoma, Colorectal cancer, Osteosarcoma, Psoriasis, Melanoma, Prostate carcinoma, Medulloblastoma, Acute lymphoblastic leukemia, Small cell lung carcinoma	https://www.targetvalidation.org/target/ENSG00000196812/associations		Nucleus	No	✓

ZSCAN18	HUMAN Zinc finger and SCAN domain-containing protein 18	ZNF447, ZSC18	Q8TBC5	ENSG0000(	Transcription factor	AC008751	Tongue squamous cell carcinoma, Breast carcinoma, Li-Fraumeni syndrome, Psoriasis, Renal cell carcinoma, Colorectal carcinoma, Congenital contractural arachnodactyly, Colorectal cancer, Non-neoplastic disorder, Medulloblastoma	https://www.targetvalidation.org/target/ENSG00000121413/associations		Nucleus	No	✓
ZSCAN21	HUMAN Zinc finger and SCAN domain-containing protein 21	NY-REN-21, ZNF38	Q9Y5A6	ENSG0000(	Transcription factor	CH471091	Breast carcinoma, Lung carcinoma, Benign prostatic hyperplasia, Chronic lymphocytic leukemia, Parkinson's disease, Pancreatic ductal adenocarcinoma	https://www.targetvalidation.org/target/ENSG00000166529/associations	SCAND1	Nucleus	No	✓
ZSCAN26	HUMAN Zinc finger and SCAN domain-containing protein 26	SRE-ZBP, ZNF187	Q16670	ENSG0000(	Transcription factor	BC013951	Breast carcinoma, T-cell acute lymphoblastic leukemia, Colorectal adenocarcinoma, Hepatocellular carcinoma, Ovarian carcinoma, Anaplstic large cell lymphoma, Psoriasis	https://www.targetvalidation.org/target/ENSG00000197062/associations		Nucleus	No	✓
ZSCAN9	HUMAN Zinc finger and SCAN domain-containing protein 9	PRD51, ZNF193, PIG12	O15535	ENSG0000(	Transcription factor	AY550973	Hepatitis C infection, Benign prostatic hyperplasia	https://www.targetvalidation.org/target/ENSG00000137185/associations		Nucleus	No	✓
