# Supplementary material for: High-throughput autoantibody screening identifies differentially abundant autoantibodies in autism spectrum disorder
Source: Front Mol Neurosci. 2023 Oct 16;16:1222506. doi: 10.3389/fnmol.2023.1222506 (PMC10613655; doi:10.3389/fnmol.2023.1222506)
Supplement: Supplementary file 1 [file Data_Sheet_1.ZIP › Supplmentary_Material_Updated/Supplementary_Material_Figures_V3.docx]

Supplementary material – Figures

High-throughput Autoantibody Screening Identifies Differential Autoantibodies and Exploratory Pathways Involved in Autism Spectrum Disorder Pathology

Areej Mesleh^1,2^, Hanan Ehtewish^1,2^, Katie Lennard^3^, Houari B. Abdesselem ^1,4^, Fouad Al-Shaban ^1,2^, Julie Decock^1,5^, Nehad M. Alajez^1,5^, Abdelilah Arredouani^1,6^, Mohamed M. Emara^7^, Omar Albagha^1^, Lawrence W. Stanton^1,2^, Sara A. Abdulla^2^, Jonathan M. Blackburnand^3,8,9^, and Omar M.A. El-Agnaf ^1,2,*^

^1^ College of Health and Life Sciences (CHLS), Hamad Bin Khalifa University (HBKU), Qatar Foundation (QF), Doha, Qatar.

^2^ Neurological Disorders Research Center, Qatar Biomedical Research Institute (QBRI), Hamad Bin Khalifa University (HBKU), Qatar Foundation (QF), Doha, Qatar.

^3^ Sengenics Corporation, Level M, Plaza Zurich, Damansara Heights, Kuala Lumpur 50490, Malaysia.

^4^ Proteomics Core Facility, Qatar Biomedical Research Institute (QBRI), Hamad Bin Khalifa University (HBKU), Qatar Foundation (QF), Doha, Qatar.

^5^ Translational Cancer and Immunity Center, Qatar Biomedical Research Institute (QBRI), Hamad Bin Khalifa University (HBKU), Qatar Foundation (QF), Doha, Qatar.

^6^ Diabetes Research Center, Qatar Biomedical Research Institute (QBRI), Hamad Bin Khalifa University (HBKU), Doha, Qatar.

^7^ Basic Medical Sciences Department, College of Medicine, QU Health, Qatar University (QU), Doha, Qatar

^8^ Department of Integrative Biomedical Sciences, Faculty of Health Sciences, University of Cape Town, South Africa.

^9^ Institute of Infectious Disease and Molecular Medicine, Faculty of Health Sciences, University of Cape Town, South Africa.

*** Correspondence:**Omar M.A. El-Agnaf
[oelagnaf@hbku.edu.qa](mailto:oelagnaf@hbku.edu.qa)

**Supplementary Figure S1.**t-SNE plots of the samples **(A)** before, and **(B)** after combat batch correction.

**A**

**B**

Supplementary Figure S2. Unsupervised heatmap of the (A) 534 autoantibodies selected for downstream analysis after quality control assessment and (B) the differentially expressed autoantibodies (n=29).

**
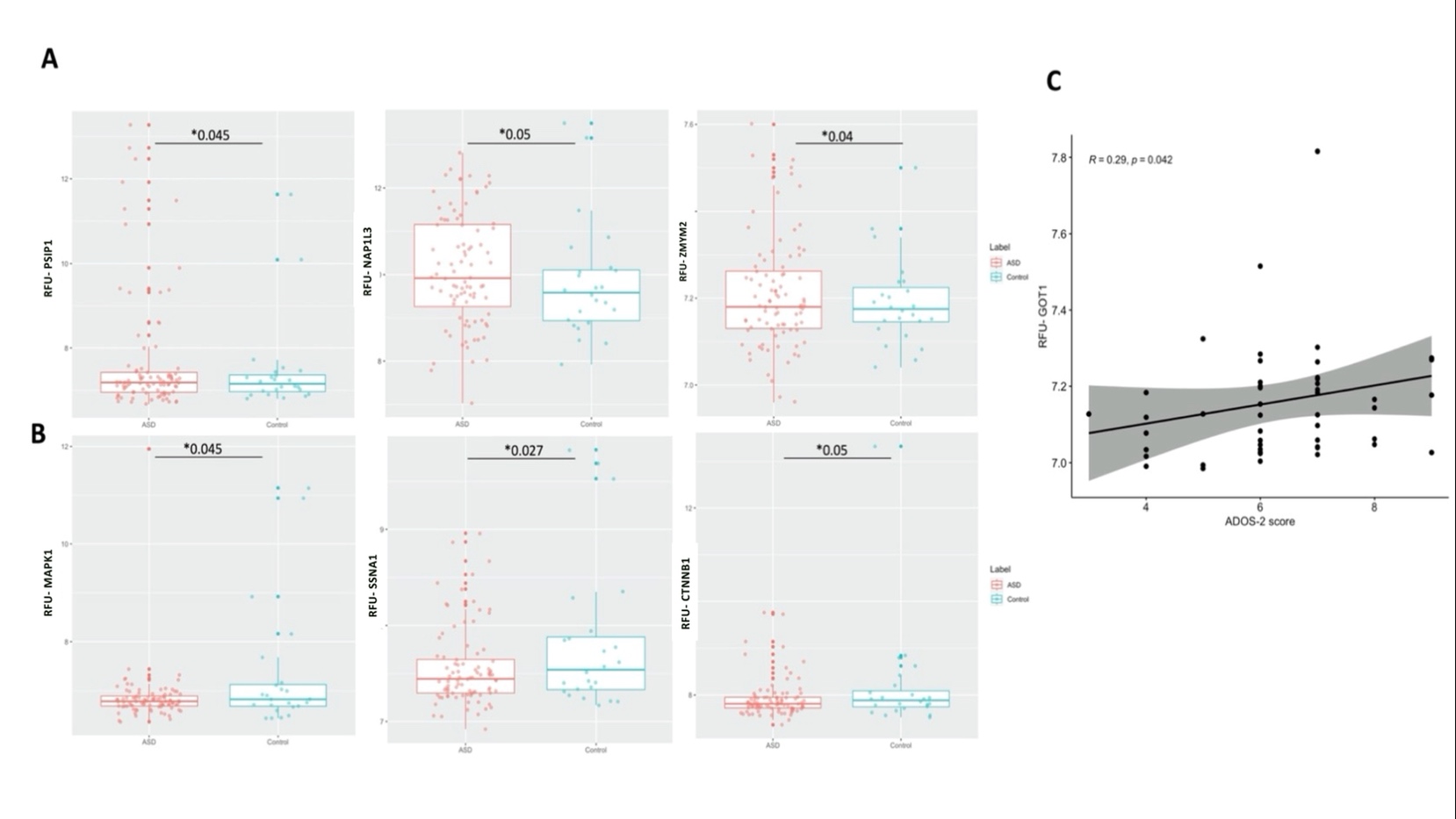
**

**Supplementary Figure S3. Boxplots and scatterplots of the top autoantibodies. (A)** Boxplots of the top upregulated and **(B)** downregulated autoantibodies. **(C)** A scatterplot of GOT1, the only differentially expressed autoantibody that significantly correlated with the ADOS-2 score. All the *p-*values on the top of the boxplots are *Limma’s* unadjusted *p-*value.


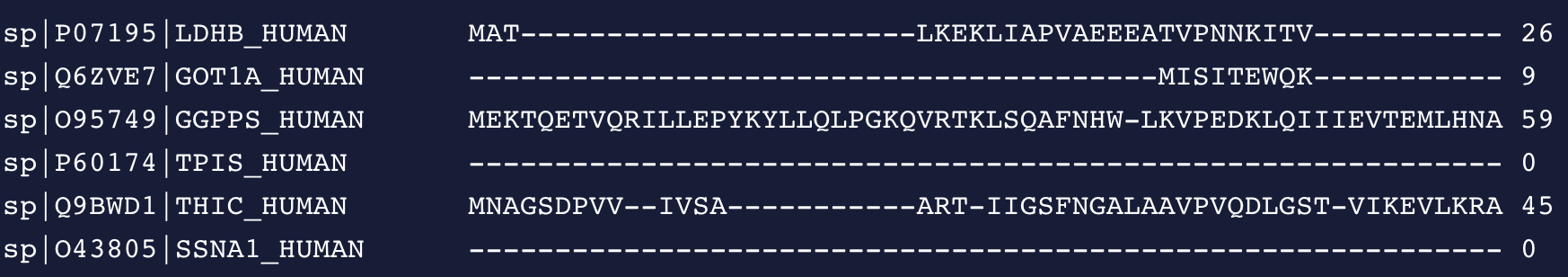

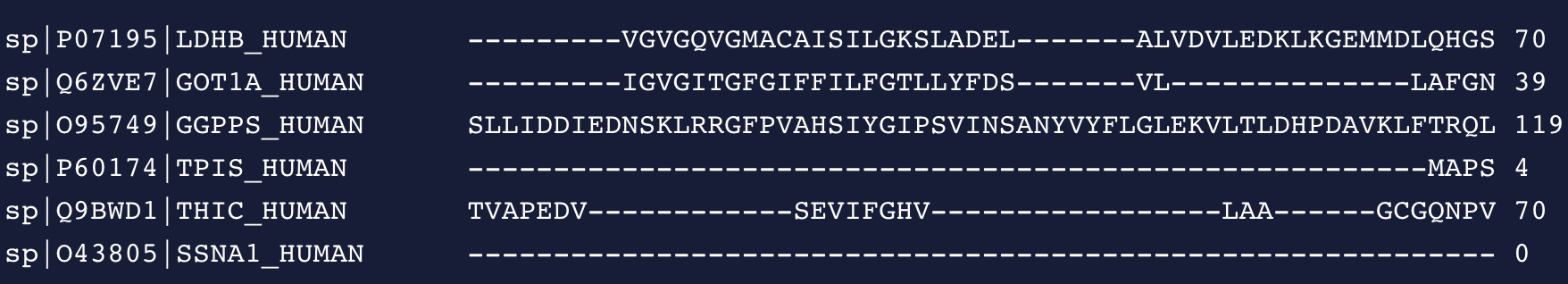

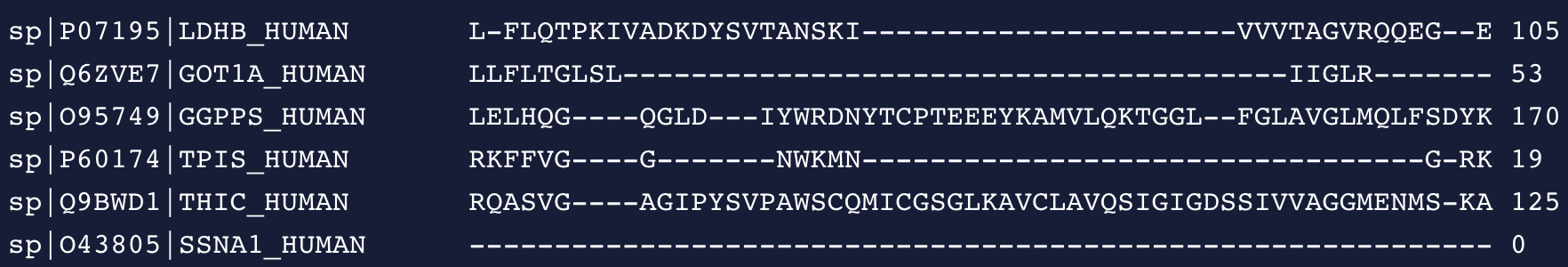

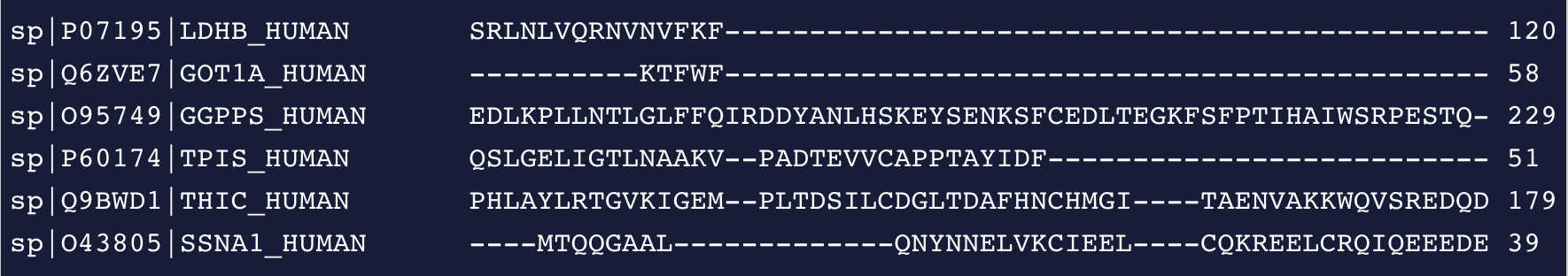

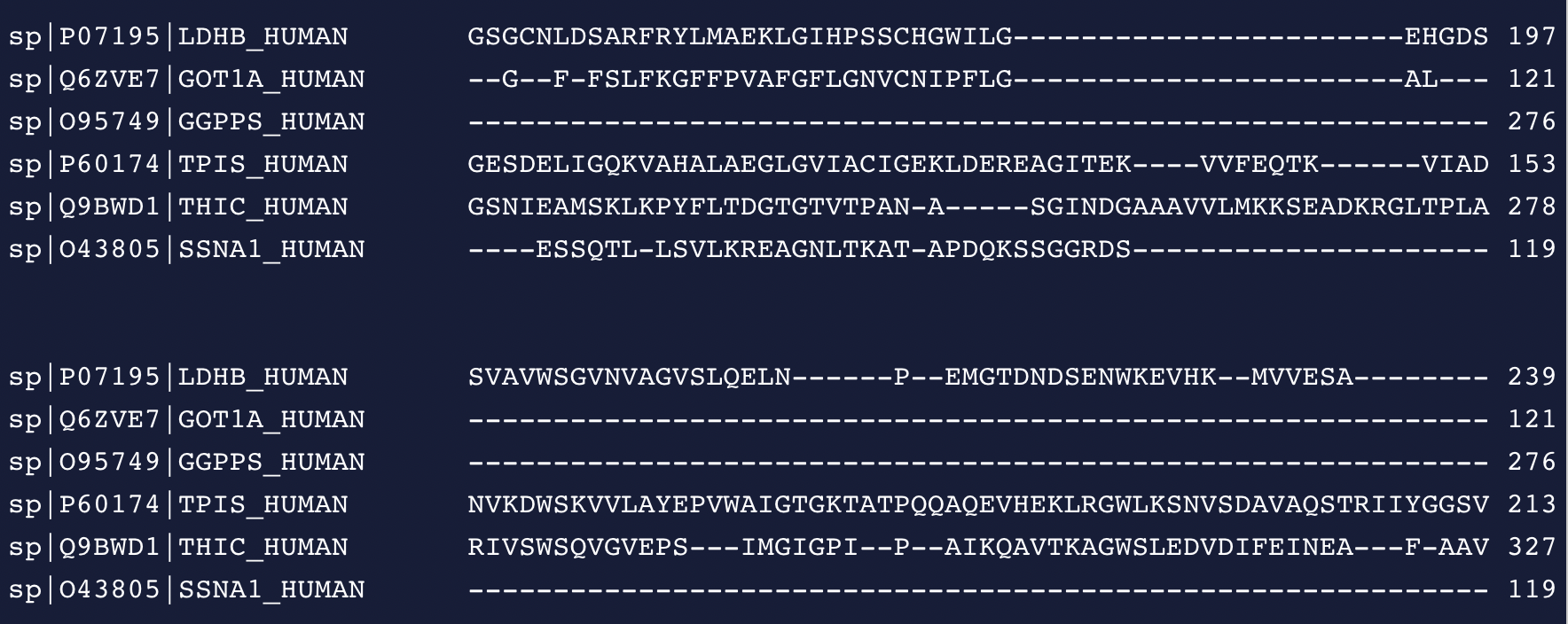

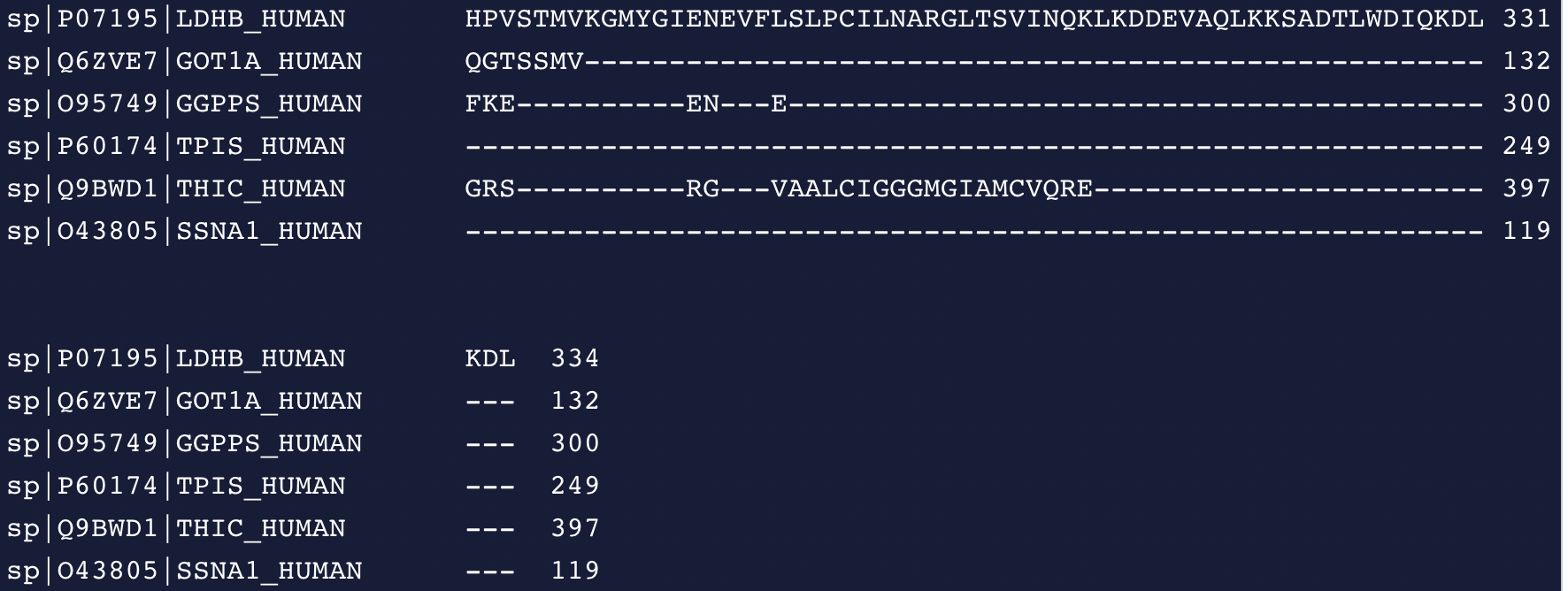


**Supplementary Figure S4.** Protein sequence alignment output of the highly correlated autoantibodies using Uniprot, THIC corresponds to ACAT2.
